# Experimental evolution of recombination and crossover interference in *Drosophila* caused by directional selection for stress-related traits

**DOI:** 10.1186/s12915-015-0206-5

**Published:** 2015-11-27

**Authors:** Dau Dayal Aggarwal, Eugenia Rashkovetsky, Pawel Michalak, Irit Cohen, Yefim Ronin, Dan Zhou, Gabriel G. Haddad, Abraham B. Korol

**Affiliations:** Institute of Evolution, University of Haifa, Haifa, 3498838 Israel; Virginia Bioinformatics Institute, Virginia Tech, Washington Street, MC 0477, Blacksburg, VA 24061-0477 USA; University of California, San Diego, USA; Rady Children’s Hospital, San Diego, USA

**Keywords:** *D. melanogaster*, Directional selection, Evolution of interference, Negative interference, Positive interference

## Abstract

**Background:**

Population genetics predicts that tight linkage between new and/or pre-existing beneficial and deleterious alleles should decrease the efficiency of natural selection in finite populations. By decoupling beneficial and deleterious alleles and facilitating the combination of beneficial alleles, recombination accelerates the formation of high-fitness genotypes. This may impose indirect selection for increased recombination. Despite the progress in theoretical understanding, interplay between recombination and selection remains a controversial issue in evolutionary biology. Even less satisfactory is the situation with crossover interference, which is a deviation of double-crossover frequency in a pair of adjacent intervals from the product of recombination rates in the two intervals expected on the assumption of crossover independence. Here, we report substantial changes in recombination and interference in three long-term directional selection experiments with *Drosophila melanogaster*: for desiccation (~50 generations), hypoxia, and hyperoxia tolerance (>200 generations each).

**Results:**

For all three experiments, we found a high interval-specific increase of recombination frequencies in selection lines (up to 40–50 % per interval) compared to the control lines. We also discovered a profound effect of selection on interference as expressed by an increased frequency of double crossovers in selection lines. Our results show that changes in interference are not necessarily coupled with increased recombination.

**Conclusions:**

Our results support the theoretical predictions that adaptation to a new environment can promote evolution toward higher recombination. Moreover, this is the first evidence of selection for different recombination-unrelated traits potentially leading, not only to evolution toward increased crossover rates, but also to changes in crossover interference, one of the fundamental features of recombination.

**Electronic supplementary material:**

The online version of this article (doi:10.1186/s12915-015-0206-5) contains supplementary material, which is available to authorized users.

## Background

Unraveling the forces responsible for the nearly universal distribution of sex and recombination among eukaryotes is one of the central problems in evolutionary biology. Several classes of models based on the combinatorial consequences of recombination (initially suggested by Weismann [1]), have been developed to explain the maintenance of sex and recombination, including selection against deleterious mutations and combination of advantageous mutations [2–7], and genetic adaptation to varying environments, both biotic and abiotic [8–12]. Tight linkage between new and/or pre-existing beneficial and deleterious alleles should decrease the efficiency of natural selection, as a consequence of the Hill-Robertson effect [13], which includes various forms of interference in finite populations [14–17]. Recombination accelerates the formation of high-fitness genotypes, which in turn can indirectly select for higher recombination rates. The shared condition for such situations is negative linkage disequilibrium (LD; <0) between fitness loci, as a result of weak negative epistasis, spatially and temporally varying selection (biotic or abiotic), or genetic drift [14, 15, 18–20]. Despite the considerable progress in theoretical analyses over the last decade, the interplay between mutation, recombination and selection remains a controversial issue in evolutionary biology, partly due to a lack of robust empirical evidence. As noted by Barton [21], *“…although the basic theoretical framework is clear, we still do not know whether selection is generally strong enough, and has the right form, to give a general advantage to sex and recombination*”. In this respect, it is worth mentioning the important and debated assumption of insufficient recombination as a limit to selection. Numerous studies support this hypothesis [20, 22–30], while opposite conclusions have also been reached [31–35] based on the idea that a low level of recombination should be sufficient to achieve most of the benefits associated with this process [36].

The existence of significant genetic variation for recombination is a precondition for efficient indirect selection for recombination. Such variation has indeed been demonstrated in many organisms [12, 37]. Experiments showing responses to direct selection for altered recombination frequency (*rf*) provide further evidence for genetic polymorphism at recombination-controlling loci [38–45]. A question arises as to whether selection for fitness-related traits can utilize this variation and lead to directional changes in *rf*. Theoretical models indicate that directional or variable selection for multilocus traits may promote evolution towards increased recombination [18, 46]. A considerable increase in *rf* as a result of selection for various traits unrelated to recombination has indeed been observed in a few studies with *Drosophila melanogaster* [47–52] (Additional file 1: Table S1). Simulation analysis suggests that interaction between drift and selection could be the source of LD <0 in most of the studies where increased recombination was caused by selection for unrelated quantitative traits [53].

Substantial evidence indicates that the observed frequency of double crossovers in adjacent intervals usually differs from the product of recombination rates in the two intervals, which is expected on the assumption of independence, a phenomenon termed crossover interference [54, 55]. The degree of interference is measured by the coefficient of coincidence (*c*), the ratio of observed to expected rates of double crossovers in target intervals: positive interference (*c* <1) corresponds to situations in which the occurrence of a crossover in one segment reduces the probability of exchange in the second segment, whereas negative interference (*c* >1) refers to situations in which the observed rate of double crossovers is higher than the expected rate with independence. Positive crossover interference is a common characteristic of meiotic organisms, with only a very few known exceptions (some fungi) where recombination proceeds with no interference ([56] and references therein). It is generally assumed that negative crossover interference is mainly associated with intragenic recombination (gene conversion). Nevertheless, cases are known of a higher than expected frequency of double crossovers in adjacent segments of small genetic but large physical length. In *Drosophila melanogaster*, a strong excess of double exchanges was reported within a 4 cM segment of chromosome 3 spanning the centromere and accounting for 25 % of its cytological length [57]. Similar results have been obtained in other *Drosophila* studies with autosomes [12, 58, 59], but not with the X chromosome [60]. Negative interference in *Drosophila* has also been shown to be associated with the interchromosomal effect of translocations on recombination and in situations with temperature-induced recombination [59]. It has been suggested that negative interference could be a characteristic of genomic regions with a low density of recombination events [57]. Despite numerous physical and formal models of interference and corresponding statistical tools to analyze experimental data on interference, only one attempt has been undertaken to explain interference as an evolvable feature [61]. As emphasized by Wang et al. [62], interference remains a mystery, an evolutionary conundrum. To our knowledge, this aspect has generally been overlooked, despite the interesting models aimed at understanding the mechanism.

Herein, we report new results showing a substantial increase in recombination frequency and changes in crossover interference in directional selection experiments with *D. melanogaster* for desiccation, hypoxia, and hyperoxia tolerance. Novel elements include the facts that (1) the effect of long-term selection (50–200 generations) for three traits unrelated to recombination was evaluated over 16 marked intervals, with independent replicates and (2) in addition to increased recombination, relaxation of positive interference and the occurrence of significant negative interference were observed, which may be considered as first evidence of experimental evolution of crossover interference.

## Results

For each of the three selection experiments, we estimated the recombination frequency and coefficient of crossover coincidence in backcrossed progeny (scheme in Additional file 2: Figure S1).

### Effect of selection for desiccation tolerance on recombination

**Fig. 1:**
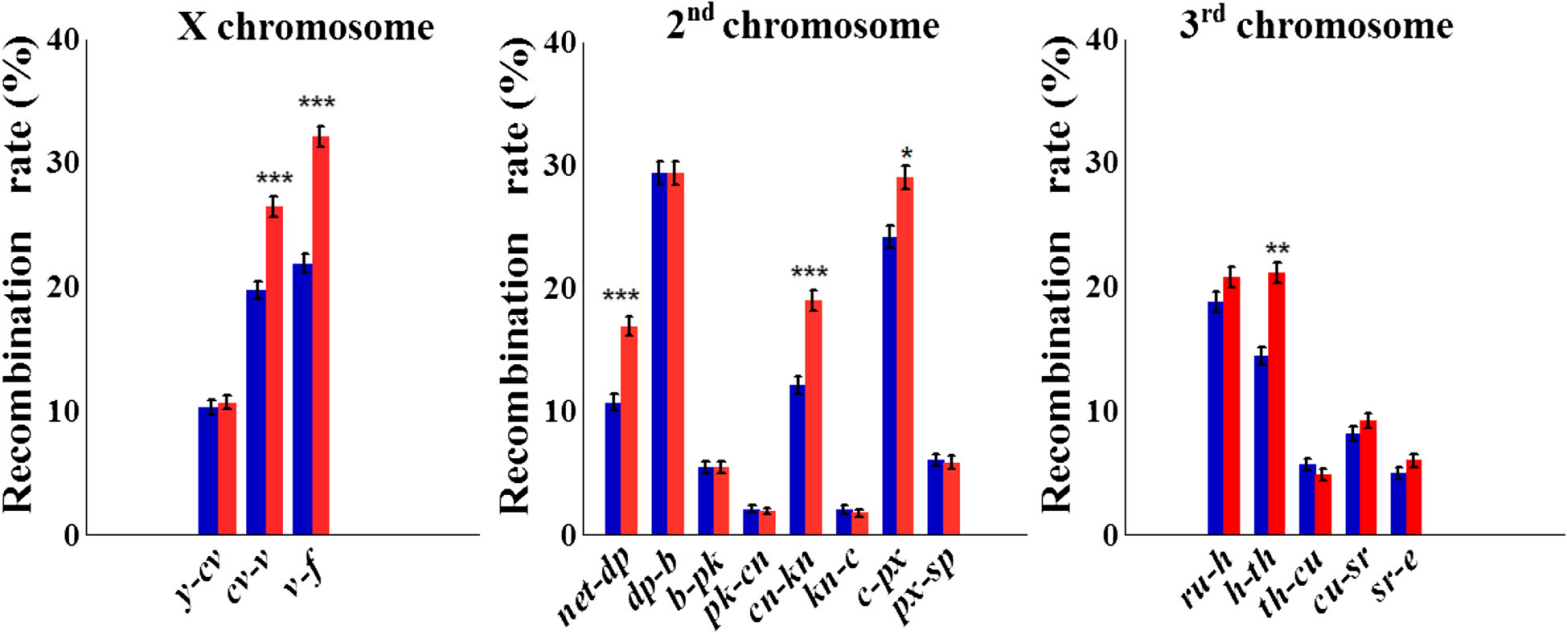
Change in recombination rates (± SE) in *D. melanogaster* caused by directional selection for desiccation tolerance. Significant increases in recombination rates were observed in selection lines (*red*) compared to control (*blue*) in intervals *cv-v* and *v-f* of chromosome X; *net-dp*, *cn-kn,* and *c-px* of chromosome 2; and *h-th* of chromosome 3. *Asterisks* indicate significant differences between selection and control at 0.01 and 0.001 levels using false discovery rate adjusted *P* values

A highly significant interval-specific increase in *rf* was observed in each of the three large chromosomes in the selection lines compared to controls (Fig. 1, Table 1; Additional file 3). In the X chromosome, we observed a maximal relative increase in *rf* (δ_*rf*_) in the proximal interval *v-f* (from 21.9 to 32.1 %, δ_*rf*_ = 46.7 %, *P* = 3.1 × 10^–18^) and a moderate increase in the interval *cv-v* (from 19.7 to 26.4 %, δ_*rf*_ = 34.3 %, *P* = 4.1× 10^–9^). In chromosome 2, increased *rf* in selection versus control lines was found in distal region *net-dp* of the 2 L arm (from 10.7 to 16.9 %, δ_*rf*_ = 58.1 %, *P* = 2.5 × 10^–8^), proximal region *cn-kn* of the 2R arm (from 12.1 to 19.0 %, δ_*rf*_ = 56.9 % *P* = 4.1 × 10^–9^), and *c-px* region of the 2R arm (from 24.1 to 29.0 %, δ_*rf*_ = 20.0 %, *P* = 1.0 × 10^–3^). In chromosome 3, an increase in *rf* was only detected in the interval *h-th*: from 14.4 to 21.1 (δ_*rf*_ = 46.6 %, *P* = 5.1 × 10^–08^). Altogether, in six out of the 16 intervals, significantly higher *rf* values were obtained in selection lines, and an opposite significant effect was not observed in any of the intervals (Table 1). The sum of *rf* estimates across the tested intervals in chromosome X has changed from 51.9 to 69.2 (δ = 33.5 %), in chromosome 2 from 91.9 to 109.3 (δ = 18.9 %), in chromosome 3 from 52.0 to 61.9 (δ = 19.1 %), and for all 16 scored intervals, from 195.8 to 240.4 (22.8 %).

**Table 1:** Effect of desiccation selection on recombination rates in 16 regions of the *D. melanogaster* genome

	Control		Desiccation		Control versus desiccation		
Interval	*rf* (%)	χ^2a^	*rf* (%)	χ^2a^	δ_*rf*_ (%)^b^	χ^2c^	*P* ^*d*^
X chromosome							
*y-cv*	10.28 ± 0.54	0.38	10.68 ± 0.55	8.52^e^	3.89	0.27	1
*cv-v*	19.69 ± 0.71	1.34	26.44 ± 0.79	2.43	34.28	40.74	4.1 × 10^–9^
*v-f*	21.89 ± 0.74	0.85	32.12 ± 0.83	3.23	46.73	84.86	3.1 × 10^–18^
2L chromosome							
*net-dp*	10.70 ± 0.65	1.58	16.92 ± 0.79	0.53	58.13	36.85	2.5 × 10^–8^
*dp-b*	29.36 ± 0.96	0.72	29.37 ± 0.96	0.21	0.03	0.00	1
*b-pk*	5.44 ± 0.48	1.92	5.49 ± 0.48	0.49	0.91	0.01	1
*pk-cn*	2.08 ± 0.30	0.31	1.91 ± 0.29	1.04	−8.17	0.16	1
2R chromosome							
*cn-kn*	12.11 ± 0.69	3.37	19.01 ± 0.82	3.63	56.98	41.26	4.1 × 10^–9^
*kn-c*	2.03 ± 0.30	5.60	1.74 ± 0.28	0.94	−14.29	0.52	1
*c-px*	24.13 ± 0.90	0.13	28.95 ± 0.95	6.59^e^	19.98	13.44	1.0 × 10^–3^
*px-sp*	6.07 ± 0.50	1.72	5.88 ± 0.50	0.75	−3.13	0.07	1
3rd chromosome							
*ru-h*	18.80 ± 0.82	1.75	20.75 ± 0.85	0.36	10.37	2.70	0.43
*h-th*	14.40 ± 0.74	2.85	21.11 ± 0.86	3.32	46.60	35.05	5.1 × 10^–8^
*th-cu*	5.64 ± 0.49	0.17	4.86 ± 0.45	1.71	−13.83	1.37	0.89
*cu-sr*	8.20 ± 0.58	0.63	9.18 ± 0.61	6.76^e^	11.95	1.37	0.89
*sr-e*	4.94 ± 0.46	0.95	5.99 ± 0.50	1.42	21.26	2.40	0.50

We scored 1,050 individuals of each line (three lines in control and three in selection variant); the total sample size was 6,300 for estimation of recombination frequency (*rf*) in X chromosome intervals. For each of the other crosses (2L, 2R, and 3), we scored 750 individuals per line, i.e. 4,500 per cross. Thus, the total sample for recombination analysis in desiccation selection experiment was 19,800 flies

^a^χ^2^ test for between-lines heterogeneity within either control of selection variants (df = 2)

^b^δ_*rf*_ (%), relative change of *rf* in selection variant compared to control variant

^c^χ^2^ test for significance between *rf* values in selection versus control (df = 1), see Methods

^d^False discovery rate corrected *P* values (two-tailed test) based on *χ* ^2 c^ are present to take into account the effect of multiple comparisons

^e^ *P* <0.05; for more details of this table, see Methods and Additional file 3

The increase in *rf* in the selection lines was accompanied by changes in crossover interference in adjacent and non-adjacent intervals (Table 2; Additional file 1: Table S2; Additional files 4 and 5). Thus, significant positive interference in the region *y-cv-v* of chromosome X in the control was replaced by no interference in the selection lines: the coefficient of coincidence *c* increased from 0.56 to 0.95 (*P* = 8.4 × 10^–3^). Moreover, in the *cv-v-f* region, significant positive interference in the control (*ĉ* = 0.70) changed to significant negative interference (*ĉ* = 1.40) in the selection lines; the difference between the two estimates was highly significant (*P* = 1.1 × 10^–16^). We did not find negative interference in chromosome 2, but the tendency towards significant relaxation of positive interference in the selection lines was expressed in both arms, e.g. in region *net-dp-b* in 2 L (from 0.35 to 0.81, *P* = 2.3 × 10^–5^) and in *cn-kn-px* in 2R (from 0.38 to 0.91, *P* = 1.3 × 10^–6^). n arm 2R, this tendency was also observed for pairs of non-adjacent intervals, e.g. for *cn*-*kn*_*c*-*px*, with *ĉ* = 0.41 in the control and *ĉ* = 0.95 in the selection lines (*P* = 3.1 × 10^–6^; Additional file 1: Table S2). As in chromosomes X and 2, selection caused a consistent and, in certain cases, highly significant tendency toward relaxation of positive interference in adjacent and non-adjacent intervals in chromosome 3. Moreover, in some pairs of intervals, significant positive interference was replaced by significant negative interference, e.g. in the *h*-*cu-sr* region, with *ĉ* = 0.41 in control and *ĉ* = 1.32 in selection lines (*P* = 5.1 × 10^–7^). Notably, we also observed a tendency toward relaxation of positive interference for intervals separated by the centromere. For example, segments *ru-h* and *h-th* are located in the 3 L arm, while *cu-sr* and *sr-e* are in the 3R arm (Additional file 2: Figure S2). A significant relaxation of positive interference was observed for several pairs of these intervals: for *ru-h*_*cu-sr*, coefficient *ĉ* changed from 0.52 to 1.14 (*P* = 2.3 × 10^–3^), for *ru-h_cu-e* from 0.41 to 0.92 (*P* = 4.0 × 10^–4^), and for *ru-th_cu-sr* from 0.36 to 0.73 (*P* = 5.8 × 10^–3^; Additional file 1: Table S3 and Additional file 5). As with adjacent intervals, replacement of significant positive interference in the control by significant negative interference in the selection lines was also found for non-adjacent intervals, e.g. for pair *h-th*_*cu-sr*, coefficient *ĉ* changed from 0.49 to 1.56 (*P* = 1.9 × 10^–6^).

**Table 2:** Effect of desiccation selection on the coefficient of coincidence in adjacent intervals of the major chromosomes of *D. melanogaster*

Intervals	Control	Desiccation	χ^2^_ML_	*P*
*y-cv-v*	0.564 ± 0.086	0.947 ± 0.084	9.39	8.4 × 10^–3^
*cv-v-f*	0.698 ± 0.061	1.395 ± 0.045	72.75	1.1 × 10^–16^
*net*-*dp*-*b*	0.352 ± 0.065	0.813 ± 0.069	21.13	2.3 × 10^–5^
*dp*-*b*-*pk*	0.110 ± 0.054	0.220 ± 0.075	1.44	0.66
*c*-*px*-*sp*	0.060 ± 0.042	0.052 ± 0.036	0.02	1
*ru*-*h*-*th*	0.814 ± 0.099	1.101 ± 0.081	0.41	0.09
*h*-*th*-*cu*	0.109 ± 0.076	0.680 ± 0.154	10.20	5.4 × 10^–3^
*th*-*cu*-*sr*	0.192 ± 0.134	0.478 ± 0.206	1.34	0.71
*cu*-*sr*-*e*	0.109 ± 0.108	0.234 ± 0.133	0.50	1

*P* values (two-tailed test) are based on unweighted likelihood tests for the difference between control and selection estimates, corrected for false discovery rate

For more details, see Additional file 1: Table S2

ML, Maximum likelihood

### Effect of two-way selection for hypoxia/hyperoxia tolerance on recombination

**Fig. 2:**
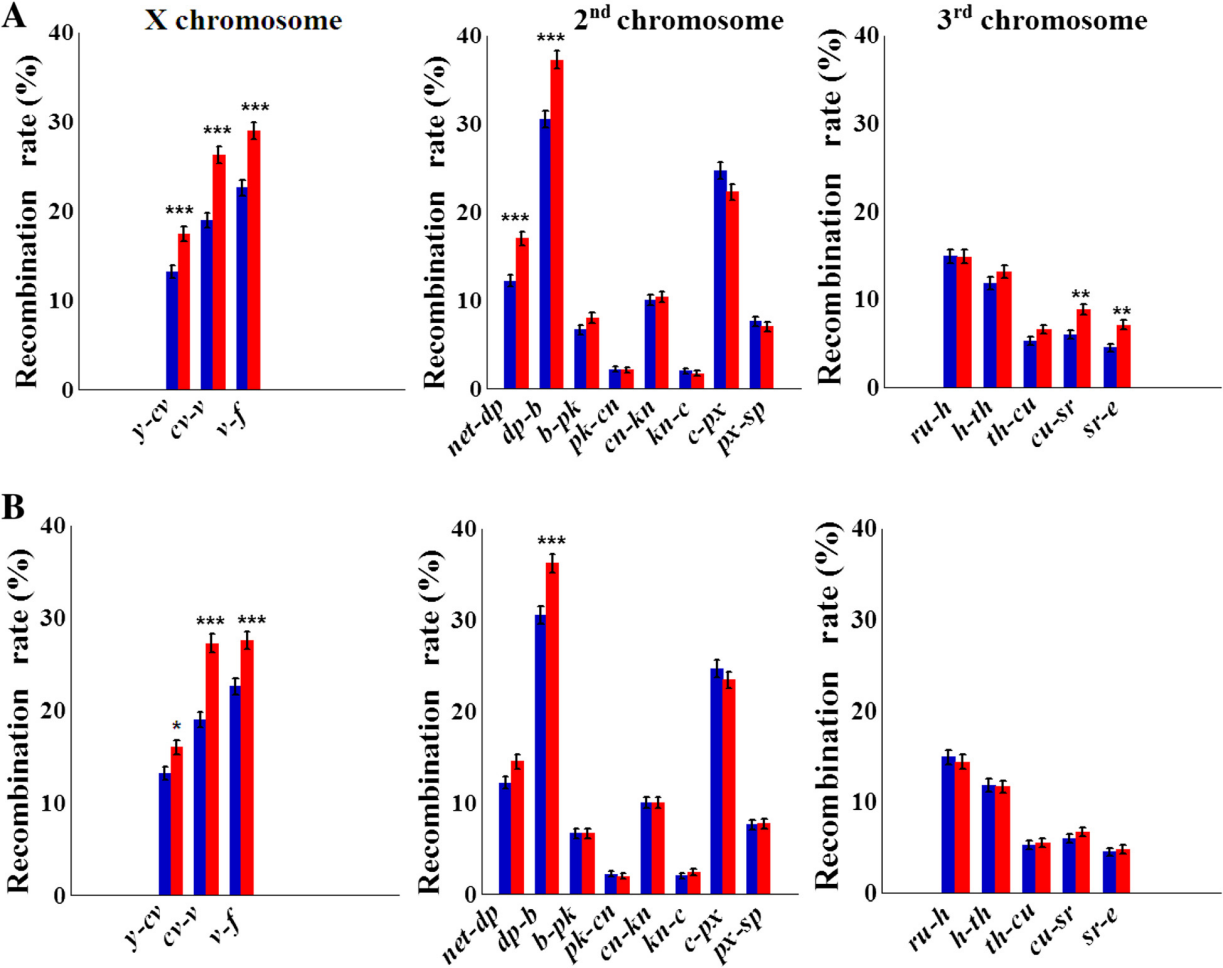
Change in recombination rates (±SE) in *D. melanogaster* caused by directional selection for (**a**) hypoxia and (**b**) hyperoxia tolerance. Significant increases in recombination rates were observed in hypoxia selection variant (*red*) compared to control (*blue*) in intervals *y-cv*, *cv-v*, and *v-f* of chromosome X; *net-dp* and *dp-b* of chromosome 2; and *cu-sr* and *sr-e* of chromosome 3. In hyperoxia selection variant, a significant increase in recombination rate was observed in all tested intervals of chromosome X, while only *dp-b* interval in chromosome 2 and in no interval of chromosome 3. *Asterisks* indicate significant differences between selection and control variants at 0.05 and 0.001 levels using false discovery rate adjusted *P* values

As with selection for desiccation tolerance, selection for both hypoxia and hyperoxia tolerance resulted in highly significant interval-specific increases in *rf* (Fig. 2, Table 3; Additional file 3)*.* No significant decrease in *rf* was observed in any of the 16 marker intervals in either direction of selection. In total, indirect selection for increased recombination had a significant effect on more intervals in hypoxia lines than in hyperoxia lines (7 vs. 4). Fisher’s exact test for the 2 × 2 contingency table of the outcomes of these two experiments across 16 intervals indicated their significant association (*P* = 0.019). The observed changes in *rf* were more pronounced in the lines selected for hypoxia tolerance (Table 3), excluding the reaction of the *cv-v* interval, with δ_*rf*_ = 38.7 % (*P* = 5.1 × 10^–8^) and 43.7 % (*P* = 2.1 × 10^–9^) in hypoxia and hyperoxia lines, respectively. This interval was among the most reactive with respect to δ_*rf*_ in the entire hypoxia/hyperoxia experiment. Other hyper-reactive intervals (all in hypoxia-tolerant lines) included *net-dp* in the 2 L arm, with δ_*rf*_ = 39.4 % (*P* = 4.2 × 10^–5^), and *cu-sr* and *sr-e* in chromosome 3, with δ_*rf*_ = 47.0 % (*P* = 1.8 × 10^–3^) and 56.9 % (*P* = 1.6 × 10^–3^), respectively_*.*_ No change in *rf* was observed in the 2R arm. The sum of *rf* values across all 16 tested intervals changed from 192.9 to 228.7 (δ = 18.6 %) in hypoxia-selected lines and to 216.5 (δ = 12.2 %) in hyperoxia-selected lines.

**Table 3:** Effect of hypoxia and hyperoxia selection on recombination rates in 16 regions of the *D. melanogaster* genome

	Control		Hypoxia		Control vs. Hypoxia			Hyperoxia		Control vs. Hyperoxia		
Interval	*rf* (%)	*χ* ^2a^	*rf* (%)	*χ* ^2a^	δ_*rf*_ (%)^b^	*χ* ^2 c^	*P* ^*d*^	*rf* (%)	*χ* ^2a^	δ_*rf*_ (%)^b^	*χ* ^2c^	*P* ^d^
X chromosome												
*y-cv*	13.15 ± 0.71	0.10	17.46 ± 0.80	6.31^e^	32.78	16.21	4.6 × 10^–4^	15.97 ± 0.77	2.37	21.44	7.21	4.1 × 10^–2^
*cv-v*	18.96 ± 0.83	0.57	26.29 ± 0.93	3.40	38.66	34.77	5.1 × 10^–8^	27.25 ± 0.94	1.99	43.72	43.43	2.1 × 10^–9^
*v-f*	22.57 ± 0.88	0.14	28.98 ± 0.96	2.04	28.40	24.30	9.8 × 10^–6^	27.52 ± 0.94	1.94	21.93	14.69	9.6 × 10^–4^
2 L chromosome												
*net-dp*	12.20 ± 0.69	3.41	17.01 ± 0.79	1.79	39.43	21.07	4.2 × 10^–5^	14.50 ± 0.74	0.91	18.85	5.19	0.12
*dp-b*	30.48 ± 0.97	0.42	37.20 ± 1.02	0.08	22.85	22.05	2.8 × 10^–5^	36.16 ± 1.01	1.04	18.64	16.42	4.5 × 10^–4^
*b-pk*	6.65 ± 0.53	2.63	7.99 ± 0.57	0.14	20.15	2.98	0.38	6.65 ± 0.52	2.73	0.00	0.00	1
*pk-cn*	2.19 ± 0.31	0.71	2.12 ± 0.30	0.41	−3.20	0.03	1	1.95 ± 0.29	2.20	−10.96	0.32	1
2R chromosome												
*cn-kn*	9.99 ± 0.63	0.37	10.38 ± 0.64	0.46	3.90	0.29	1	10.01 ± 0.63	0.89	0.20	0.02	1
*kn-c*	2.02 ± 0.30	1.69	1.73 ± 0.27	3.84	14.36	0.52	1	2.40 ± 0.32	1.02	18.81	0.77	1
*c-px*	24.67 ± 0.91	5.88	22.21 ± 0.88	0.54	−9.97	3.79	0.26	23.42 ± 0.89	3.76	−5.07	0.95	1
*px-sp*	7.58 ± 0.56	0.51	7.01 ± 0.54	2.73	−7.52	0.55	1	7.71 ± 0.56	1.82	1.72	0.03	1
3rd chromosome												
*ru-h*	14.89 ± 0.75	2.94	14.84 ± 0.75	2.89	−0.34	0.01	1	14.39 ± 0.74	0.40	−3.36	0.22	1
*h-th*	11.82 ± 0.68	5.27	13.09 ± 0.71	3.34	10.74	1.67	0.80	11.66 ± 0.68	0.79	−1.35	0.03	1
*th-cu*	5.24 ± 0.47	4.52	6.58 ± 0.52	4.72	25.57	3.59	0.28	5.48 ± 0.48	1.82	4.58	0.13	1
*cu-sr*	5.98 ± 0.50	1.69	8.79 ± 0.60	1.40	46.99	13.08	1.8 × 10^–3^	6.70 ± 0.53	0.38	12.04	1.23	0.96
*sr-e*	4.50 ± 0.44	0.84	7.06 ± 0.54	0.28	56.89	13.55	1.6 × 10^–3^	4.75 ± 0.45	0.14	5.56	0.16	1

For each cross, we scored 750 individuals of each line (3 lines each in control, hypoxia and hyperoxia variant) i.e., 6750 per cross. Thus, a total 27,000 flies were scored for recombination analysis in X chromosome, 2L arm, 2R arm and 3 chromosome in hypxoxia-hyperoxia experiment.

^a^ *χ* ^2^test for between-lines heterogeneity within either control of selection variants (df = 2)

^b^δ_*rf*_ (%), relative change of *rf* in selection variant compared to control variant

^c^ *χ* ^2^ test for significance between *rf* values in selection versus control (df = 1), see Methods

^d^False discovery rate corrected *P* values (two-tailed test) based on *χ* ^2c^ are present to take into account the effect of multiple comparisons

^e^ *P* <0.05; for more details of this table, see Methods and Additional file 3

Selection for hypoxia and hyperoxia tolerance also caused relaxation of positive interference and appearance of negative interference. In the X chromosome, the latter effect was expressed particularly strongly, in both directions of selection, in pairs of adjacent and non-adjacent intervals (Table 4; Additional file 1: Table S3 and Additional files 4 and 5). Remarkably, for the *y-cv-f* region, no interference in the control (*ĉ* = 1.05) changed to highly significant negative interference in the selection lines: *ĉ* = 2.12 in hypoxia (*P* = 9.5 × 10^–28^) and *ĉ* = 2.09 in hyperoxia (*P* = 6.8 × 10^–25^). A similar pattern, for either adjacent or non-adjacent pairs of intervals (*net*-*dp*-*b, dp*-*b*-*pk, net*-*dp*_*b*-*pk,* and *net*-*dp*_*b*-*cn*), was observed in the 2 L arm for both directions of selection (Additional file 1: Table S3). The difference between control and selection lines was more pronounced for hypoxia selection and for adjacent pairs of intervals. Although selection had no significant effect on *rf* in arm 2R, changes in crossover interference in adjacent and non-adjacent intervals of 2R were observed in the hyperoxia selection lines. Thus, for adjacent intervals, the coefficient of coincidence increased from *ĉ* = 0.43 to 0.72 (*P =* 2.9 × 10^–2^) for *cn*-*kn*-*sp*, from 0.42 to 0.70 (*P =* 1.8 × 10^–2^) for *cn*-*c*-*sp*, and from 0.37 to 0.66 (*P =* 4.6 × 10^–2^) for *cn*-*px*-*sp*. In chromosome 3, no changes in *rf* or interference were found in hyperoxia-tolerant lines. Although no increase in *rf* in the lines selected for hypoxia tolerance was detected in the 3 L arm, we observed significant changes in interference in this arm: either considerable relaxation of strong positive interference (e.g. in *h*-*th*-*sr* region) or replacement of significant positive interference with no interference (e.g. in *ru*-*h*-*th* region). Relaxation of interference was also noted for non-adjacent intervals, including across-centromere effects: for the pair of intervals *ru*-*th*_*cu*-*e*, coefficient *ĉ* changed from 0.15 to 0.49 (*P =* 4.8 × 10^–4^).

**Table 4:** Effect of hypoxia and hyperoxia selection on the coefficient of coincidence in adjacent intervals of the major chromosomes of *D. melanogaster*

Intervals	Control	Hypoxia	χ^2^_ML_	*P* (control vs. hypoxia)	Hyperoxia	χ^2^_ML_	*P* (control vs. hyperoxia)
*y-cv-v*	0.712 ± 0.099	1.108 ± 0.078	8.88	1.1 × 10^–2^	0.883 ± 0.076	1.72	0.55
*cv-v-f*	0.737 ± 0.074	1.118 ± 0.056	16.07	2.8 × 10^–4^	1.200 ± 0.058	22.83	9.7 × 10^–6^
*net*-*dp*-*b*	0.332 ± 0.058	1.295 ± 0.062	96.20	7.9 × 10^–22^	1.014 ± 0.068	49.46	1.4 × 10^–11^
*dp*-*b*-*pk*	0.540 ± 0.097	1.195 ± 0.095	20.45	3.2 × 10^–5^	1.146 ± 0.107	16.42	2.3 × 10^–4^
*b*-*pk*-*cn*	0.301 ± 0.295	0.262 ± 0.258	0.01	1	0.643 ± 0.442	0.41	1
*kn*-*c*-*sp*	0.272 ± 0.129	0.179 ± 0.124	0.01	1	0.422 ± 0.148	0.84	0.97
*c*-*px*-*sp*	0.283 ± 0.078	0.253 ± 0.081	0.08	1	0.316 ± 0.083	0.08	1
*ru*-*h*-*th*	0.495 ± 0.103	0.995 ± 0.129	8.97	1.0 × 10^–2^	0.396 ± 0.097	0.44	1
*h*-*th*-*cu*	0.137 ± 0.095	0.598 ± 0.162	5.50	0.06	0.342 ± 0.148	1.42	0.60

*P* values (two-tailed test) are based on unweighted likelihood tests for the difference between control and selection estimates, corrected for false discovery rate

For more details, see Additional file 1: Table S3

ML, Maximum likelihood

### Between-replicate heterogeneity in recombination rates and changes in interference

Analysis of 16 genomic intervals showed segment-specific increases in recombination rate and relaxation of positive interference, or even its replacement by negative interference in all three selection experiments compared to the corresponding controls. The question is whether the changes in interference, deduced using the estimates of coefficient of coincidence, represent a ‘true’ cytogenetic effect, or an alternative process? Säll and Bengtsson [63] demonstrated that, even in the absence of negative interference, heterogeneity of recombination rates within a sample, with positive covariation of *rf* values in two intervals, may lead to biased upward *ĉ* and even to *ĉ* values highly significantly exceeding *c* = 1, i.e. the false discovery of negative interference. To reduce the risk of such outcomes when combining potentially heterogeneous data from the replicated lines, we used a weighted maximum likelihood (ML) approach in addition to the standard ML approach (see Methods). A special analysis of data heterogeneity and correlation between *rf* values was performed to assess the possible effect on *ĉ* estimates.

As explained in Methods, the segregating progeny of each of the three replicate control and selection lines were obtained in three bottles each with approximately 250 flies (Additional file 2: Figure S1). Although each such trio of sub-samples represents the same selection or control line, analyzing them separately enables taking into account one additional source of variation on post-meiotic stages (differential survival of the progeny) that might affect the *rf* estimates. The small size of these sub-samples (n = 250) precludes the possibility of interference analysis for a considerable portion of the interval pairs on such a sub-replicate level, but linkage analysis is still possible. Thus, based on nine data points (three replicate lines × three bottles of backcross segregants per line), we could calculate the correlation between *rf* values for pairs of intervals, either adjacent and non-adjacent (Additional file 1: Tables S2 and S3 for desiccation and hypoxia/hyperoxia selection experiments, respectively). For each of the three experiments, the following question can be addressed: is there any association between a significant change of *c* in selection material for certain interval pairs and a significant positive correlation between *rf* values for the same interval pairs? The analysis (Additional file 1: Table S4a) suggests that this factor does not explain the cases of significant increase of *c* values in selection lines in any of the three selection experiments. Likewise, cases with significant increase of *c* in selection lines do not show strong associations with significant increase in *rf* in one or both segments (Additional file 1: Table S4b,c and Additional file 6: Text S1).

### Additional observations on negative interference

In all three experiments, the most pronounced changes toward negative interference were observed in the X chromosome. In the 2 L arm, negative interference appeared in a number of cases in hypoxia- and hyperoxia-tolerant lines, while desiccation-tolerant lines manifested only a reduction in positive interference. In the 2R arm, desiccation- and hyperoxia-tolerant lines showed relaxation of positive interference, while no such effect was observed in the hypoxia-tolerant lines. In some intervals of chromosome 3, negative interference appeared in the desiccation-tolerant lines, while only relaxation of positive interference was observed in hypoxia-tolerant lines, and there was no effect in hyperoxia-tolerant lines. We also found that negative interference caused by selection can be accompanied by a decrease in *rf* over long intervals compared to controls. Thus, *rf* values along the X chromosome in hypoxia-tolerance selection lines significantly exceeded corresponding control values (Table 3): 17.46 vs. 13.15 in *y-cv*, 26.29 vs. 18.96 in *cv-v*, and 28.98 vs. 22.57 in *v-f*. Nevertheless, the *rf*_*y-f*_ value in control lines was significantly higher than that in selection lines: 38.58 vs. 26.66 (Additional file 3). Similarly, a higher *rf* value for *y-f* in control lines compared to selection lines was observed in the hyperoxia experiment (38.58 in control vs. 27.77 in selection) and desiccation experiment (39.26 in control vs. 31.46 in selection), as well as for *net-cn* of the 2L arm in hypoxia and hyperoxia experiments (45.64 in control vs. 35.84 in hypoxia and 39.68 in hyperoxia; Additional file 3). This non-monotonicity can be explained by the increased chance of double-recombination events observed among the component shorter intervals (see the corresponding estimates of *c* in Additional file 1: Tables S2 and S3). Our results of non-monotonicity raises an important point about marker spacing in experimental recombination-evolution studies: by choosing intervals that are too long, one might actually observe *rf* values that remain the same or appear to decrease even if the map length has truly increased (due to the increased chance of double crossovers). This comment may be relevant for some of the long intervals used in the previous studies and the current data.

## Discussion 

We estimated genomic changes in recombination in *D. melanogaster* caused by long-term selection (50–200 generations) for tolerance to desiccation, hypoxia, and hyperoxia. Using the same sets of markers, we provide robust evidence of indirect selection for recombination in all three experiments. We found that long-term selection has resulted in a dramatic increase in recombination rates in different genomic regions (up to 40–50 % per interval) relative to control levels. A higher response was displayed by the X chromosome compared to autosomes in all the three experiments. No significant reduction in *rf* was observed in any of the 16 genomic intervals analyzed, for any of the three experiments. Remarkably, in addition to the unidirectional changes in *rf*, we observed a highly significant increase in the rate of double crossovers, expressed as relaxation of positive interference and occurrence of negative interference. Relaxation of positive interference was evident for all tested chromosomes in all three experiments, whereas the intervals with selection-induced negative interference differed between the selection regimes.

### Crossover interference as an evolving phenotype

A comparison of meiotic mutants and normal genotypes leads to the conclusion that the genomic distribution of crossover exchanges in normal meiosis is more restricted and less proportional to physical distances than in meiosis altered by mutations [64–66]. Thus, these restrictions may be largely a result of evolutionary adjustments of crossover distribution along the chromosome. Relaxation of (positive) interference in meiotic mutants has also been observed, despite a general tendency toward linkage tightening [67–69]. Such effects (crossover re-distribution along the chromosome and relaxation of interference) were also displayed by mei-mutants with increased recombination rates [70]. Such observations suggest that the direction and level of interference are evolvable phenotypes. A first, formal analysis of interference-modifier evolution was conducted by Goldstein et al. [61]. Using numerical analysis, they showed that in an overdominance selection model, interference modifiers evolve to reduce the overall recombination rate, whereas in a mutation-selection balance model interference can evolve toward an overall increase in recombination if fitness effects of the selected loci are super-multiplicative. However, there has been no evidence available to date showing changes in interference in evolution experiments. Our results indicate that long-term directional selection for recombination-unrelated traits may lead not only to an increase in recombination rates, but also to relaxation of positive interference and appearance of negative interference.

### Alternative explanations for the obtained results

The repeatable observation of association between directional selection and increased recombination implies selection for rec modifiers [12, 71, 72] or changes in the respective genomic regions’ ability to recombine. These two mutually non-exclusive scenarios can be considered as changes in regulating and reacting systems of the hierarchical control of recombination [12, 72]. The fixation of polymorphic recombination hotspot motifs can serve as an example of changes in the reacting system. Selection pressure may also strengthen the ability to recombine if the initial material was heterozygous for small inversions and evolved toward structural homozygosity due to selection and drift. However, despite our growing understanding of the importance of structural heterozygosity in population-genetic experiments with *D. melanogaster* [73], this assumption cannot explain the reproducibility of the observed patterns among replicates and the similarity of *rf* values between the controls for desiccation and hypoxia/hyperoxia experiments, as well as their good correspondence with the standard *D. melanogaster* genetic map. More importantly, this assumption is also incompatible with exclusively upward changes in *rf* in all selection lines (Tables 1 and 3). Another assumption, that the increase in *rf* was caused by initial positive LDs between the advantageous alleles conferring resistance (to desiccation, hypoxia, or hyperoxia) and recombination alleles increasing *rf*, is also improbable. Indeed, this assumption implies a prevalence of cis-regulation of recombination for all intervals that showed a selection-induced increase in *rf*; it also requires a further assumption of uniformity of signs of such LDs. Moreover, this explanation contradicts our findings of unidirectional changes of *rf* in both hypoxia and hyperoxia selection experiments, similar to earlier findings of unidirectional changes of *rf* in two-way selection for geotaxis [51].

## Conclusions

Theoretical analysis shows that the fitness epistasis caused by truncation selection with a steadily moving optimum can have a powerful effect on selection for increased recombination in large populations [18]. An alternative mechanism is fluctuation of LD in small populations combined with directional selection, which may also lead to higher recombination [19]. In the present study, we observed increased recombination in three independent replicates of each selection experiment – for desiccation, hypoxia, and hyperoxia tolerance. Presumably, both abovementioned mechanisms could play a role in the observed changes in recombination. However, although selection × drift interaction may be an important factor contributing to the evolutionary advantage of increased recombination, the high uniformity of the replicates enables us to suggest that directional selection with a steadily moving optimum has played a leading role in the observed recombination response. As shown by Charlesworth [18], selection pressure on a rec-modifier when a trait is subject to selection with a steadily moving optimum should be sufficient to account for observed increases in *rf* in artificial selection experiments, especially for organisms with small chromosome number, like *D. melanogaster*.

The observed pattern of recombination changes across the genome induced by selection for traits unrelated to recombination does not necessarily adequately reflect the distribution of loci affecting those traits. Flexon and Rodell [48] did find such a correspondence in their pioneering study of the effect of selection for resistance to DDT on recombination in *D. melanogaster* and revealed a positive correlation between the chromosome contribution to resistance and the extent of change in *rf* relative to the control level. It is worth noting that experiments involving direct selection for changed recombination have shown that selection for *rf* in one region can result in a spectrum of correlated changes in other regions with different chromosomes being involved in this changed control of recombination [39, 41, 45]. Concerning our results, out of 188 genes residing in hypoxia-tolerance selected regions [74], 44 are located on the 3R arm and 144 on the X chromosome; 10 of these genes from 3R and 52 from X belong to the intervals with observed significant increases in *rf* (*y*-*f* for X and *th*-*e* for 3R). To evaluate whether the increase in *rf* is coordinated with the selection of new combinations of alleles of relevant tolerance genes, these results should be complemented with fine-scale assays of recombination landscapes and genome scanning for footprints of selection. This would enable testing whether alterations in the recombination system caused by long-term selection include a change in the ‘spectrum of recombinants’, i.e. involvement in crossover exchanges in genomic regions that were excluded from crossing-over in controls [12, 68], or simply reflect a quantitative increase in *rf*.

Presumably, episodes of novel intensive selection pressures are not uncommon in nature [14, 15, 75]. As noted by Barton [14], “*…it remains possible that local populations experience far more directional selection, and that it is this which sustains widespread sex and recombination*”. *D. melanogaster* is one of the organisms that, at least outside of its native habitats in Africa, seems to undergo boom-bust cycles, dramatically reducing the long-term effective population size and allowing adaptation in the boom years to occur in populations of large short-term effective population size, enabling short-term evolution to act primarily on pre-existing intermediate-frequency genetic variants that are driven the rest of the way to fixation via soft sweeps [76, 77]. The results of the current study indicate that selection for stress tolerance can lead to a considerable increase in the level of recombination and also deeply modify such basic features of recombination as crossover interference, displayed by relaxation of positive interference, and even evolution of negative interference. Till now, theoretical studies of recombination evolution have been concentrated on the central question of ‘why sex and recombination?’, ignoring the fact that several important features of recombination also remain unexplained, including its environmental dependence, widespread occurrence of crossover interference, sex differences in *rf*, and its species-specificity, to name just a few ([12, 71, 78]; but see [12, 79, 80]). Comparative analysis of recombination in ecologically divergent populations and assessment of changes in recombination in selection experiments may serve as an important source of evidence for better understanding of the mechanisms of maintenance of sexual recombination and explaining why recombination is so variable within and between species.

## Methods

Three sets of *D. melanogaster* lines resulting from long-term directional selection for stress tolerance were employed in our experiments: (1) three desiccation-resistant lines established by selection over 48 generations; (2) three lines tolerant to severe hypoxic stress generated through long-term experimental selection (for more than 200 generations), and (3) three hyperoxia-tolerant lines. Details of the experimental scheme for hypoxia-tolerance selection were provided elsewhere [81, 82]. Peculiarities of the selection for hyperoxia tolerance are described by Zhao et al. [83]. Selection for desiccation tolerance was performed by DDA. 

### Selection for desiccation tolerance

Wild individuals of *D. melanogaster* (n = 120) were collected in March 2009 from Madhya Pradesh, Jabalpur, India (23°30’N; 80°01’E; alt. 393 m). Before the start of the selection experiment, mass culture was maintained for five generations under standard laboratory conditions at low density (on yeast-cornmeal-agar medium at 21 °C, and ~70 % relative humidity) to eliminate environmental effects. For laboratory selection, virgin flies were sexed under CO_2_ anesthesia at least 48 h prior to the experiment. Then, virgin flies (3–4 days old) were placed in groups of 25 into plastic vials containing 2 g of silica gel and covered with foam discs. Experiments were conducted for males and females separately. Flies were subjected to desiccation stress until approximately LT_70_–LT_85_ level of mortality was reached. Control groups were established in the same manner, excluding water stress. In each generation, we examined approximately 1,000 virgin flies of each sex per replicate, of which at least 100 males and 100 females survived the LT_70–85_ cut-off to become the parents of the next generation. For each group (selection and control), survivors were randomly allocated into three sub-groups (three replicates). The same protocol was repeated for 48 generations (each next generation was subjected to analogous treatment), and then selection was relaxed for 8–10 generations before initiating the recombination tests. The control lines were not subjected to any treatment and were maintained in comparable densities to the selection lines on standard media. In the present study, we used three control and three desiccation-resistant lines for recombination tests. Average desiccation tolerance of the initial population was 14.8 h and 23.2 h (with SD = 2.88 and 3.44), for males and females, respectively. After 48 generations of selection, these tolerance characteristics increased to 25.3 h and 43.6 h for males and females, respectively, i.e. 3.65 SDs and 5.93 SDs compared to the starting population.

### Hypoxia- and hyperoxia-tolerant lines

Selection for hypoxia/hyperoxia tolerance was initiated after crossing 27 isofemale *D. melanogaster* lines (kindly provided by Dr. Andrew Davis), that varied considerably in acute anoxia test as well as for eclosion rates when cultured under hypoxic or hyperoxic conditions. Males and virgin females (n = 20) were collected and pooled from each isofemale line. This parental population was reared at room temperature with standard food medium. F_1_ embryos from the pooled population were separated and maintained in nine separate chambers, three each for control, hypoxia- and hyperoxia-selection experiments. Trial experiments were run to determine the starting O_2_ concentrations for hypoxia- and hyperoxia-tolerance selection. We analyzed the feasibility and tolerance capacity of the F_1_ progeny of the parental cross to different O_2_ concentrations (i.e. 8, 6, or 4 % O_2_ for hypoxia selection and 60 %, 70 %, 80 % and 90 % O_2_ for hyperoxia selection). In addition, the tolerance levels of each parental line to hypoxia or hyperoxia were measured by testing survival of each individual line in the hypoxic or hyperoxic environments. In the pilot study, the selection for hypoxia tolerance was therefore started at 8 % O_2_ and for hyperoxia tolerance at 60 % O_2_. The low O_2_ concentration was gradually decreased by 1 % and the high O_2_ was increased by 10 % every 3 to 5 generations to maintain the selection pressure. The population size was kept at around 2,000 flies in each generation. Eggs of the first egg laying for each generation were removed to limit genetic drift induced by the ‘early-bird’ effect. After seven generations of selection, hyperoxia tolerance was increased to 80 % O_2_, and after 13 generations the hypoxia tolerance in the hypoxia-selected flies reached 5 %, a level that is lethal for most of the control flies (Additional file 2: Figure S3). The hyperoxia-selected flies broke through the lethal hyperoxic level (90 % O_2_) after 13 generations of selection, and the hypoxia-selected flies exhibited tolerance to a severe level of hypoxia (4 % O_2_, embryonic lethal to control flies) following 32 generations of selection. The lethality in these selection experiments was defined as the level of oxygen in which *D. melanogaster* cannot complete development and reproduce.

### Genetic crosses

Virgin females (3 days post-eclosion) of each control and selection lines (three replicate lines each for control and selection groups) were allowed to mate with males of marker stocks (Additional file 2: Figure S1). Four marker stocks were employed (Additional file 2: Figure S2): *y cv v f* for the X chromosome; *net dp b pk cn* for the 2 L arm, *cn kn c px sp* for the 2R arm, and *ru h th cu sr e* for chromosome 3. F_1_ heterozygous virgin females were collected for each replicate line, and thereafter test-crossed with marker males. Because maternal age may also influence *rf* in *D. melanogaster*, we reduced this effect by allowing the 50- to 60-hour old (post-eclosion) F_1_ virgin females to mate with marker males for approximately 48 hours. To obtain a sufficient number of flies per replicate for scoring recombination, each replicate line was divided into three sub-replicates before the start of recombination experimentation. In this panel, we scored recombination in nine sub-replicates of three replicate lines each for control and selection. In the desiccation experiment, we scored 1,050 individuals of each replicate line (or 350 individuals per sub-replicate), i.e. a total 6,300 flies were counted for estimation of *rf* at the X chromosome. We scored 750 individuals of each replicate line (or 250 individuals per sub-replicate), i.e. 4,500 individuals each were scored for arms 2 L and 2R and chromosome 3. A total of 19,800 flies were counted for estimation of *rf* in the desiccation-selection experiment. Similarly, 750 flies per line, or a total 27,000 flies, were scored for *rf* in the hypoxia/hyperoxia experiments. In the three experiments, we scored a total of 46,800 individuals.

### Statistical analysis

For each pair of intervals and each of the three control or selection lines, ML analysis was performed to estimate the recombination frequencies *r*_1*k*_ and *r*_2k_ together with the coefficient of coincidence *c*_*k*_ (*k* = 1,2,3). For a pair of intervals, either adjacent or non-adjacent, the log-likelihood function had the following form:

$$ \log \left(L\left(r{1}_k,r{2}_k,{c}_k\right)\right)=\sum_{ij,k}{n}_{ij,k} \log \left({p}_{ij,k}\left(\kern0.10em r{1}_k,r{2}_k,{c}_k\right)\right) $$

where *i*, *j ϵ* {0, 1} define whether the recombination event occurred in the first or second interval, respectively (0 – no recombination, 1 – recombination), *k* denotes the replicate line, and *p*_*ijk*_ and *n*_*ijk*_ represent the probability and the observed number of individuals of the genotype class *ij* in replicate line *k* in the backcross progeny (within control or selection). The frequencies for the four genotype classes were defined as:

$$ \begin{array}{l}{p}_{11,k}=\left(r{1}_kr{2}_k{c}_k\right),\kern1em \\ {}{p}_{01,k}=r{2}_k\left(1-r{1}_k{c}_k\right),\kern1em \\ {}{p}_{01,k}=r{1}_k\left(1-r{2}_k{c}_k\right),\kern1em \\ {}{p}_{00,k}=\left(1-r{2}_k-r{1}_k+r{1}_kr{2}_k{c}_k\right).\kern1em \end{array} $$

The ML estimate $\widehat{{\boldsymbol{\uptheta}}_k}$ of the vector **θ**_***k***_ = (*r*1_*k*_, *r*2_*k*_, *c*_*k*_) for *k* = 1,2,3 was obtained by numerical optimization of the log-likelihood function *L* (**θ**_***k***_), using the gradient-descent procedure in which all three parameters *r*1_*k*_, *r*2_*k*_ and *c*_*k*_ are evaluated simultaneously in every iteration:

$$ \begin{array}{l}r{1}_{n+1,k}=r{1}_{n,k}-{\alpha}_{n+1}\frac{\partial \mathrm{L}\left({\boldsymbol{\uptheta}}_{\boldsymbol{\kappa}}\right)}{\partial r{1}_k}\\ {}r{2}_{n+1,k}=r{2}_{n,k}-{\alpha}_{n+1}\frac{\partial \mathrm{L}\left({\boldsymbol{\uptheta}}_{\boldsymbol{\kappa}}\right)}{\partial r{2}_k}\\ {}{c}_{n+1,k}={c}_{n,k}-{\alpha}_{n+1}\frac{\partial \mathrm{L}\left({\boldsymbol{\uptheta}}_{\boldsymbol{\kappa}}\right)}{\partial {c}_k}\end{array} $$

where *n* refers to iteration number, *k* to the line (within control or selection), and α to the step size. The variances of the estimated parameters *r*_1*k*_, *r*_2*k*_, *c*_*k*_ were calculated as corresponding diagonal elements of the covariance matrix **V**_*k*_ **= I**^**−**1^(${\widehat{\boldsymbol{\uptheta}}}_k$ ) = **I**_***k***_^**−**1^, where **I** is the Fisher’s information matrix [54]. The estimates of the parameter vector Θ = (*r*_1_, *r*_2_, *c*) for the entire group (control or selection) together with the vector **V**_**Θ**_ of their variances, were obtained as:

$$ \widehat{\boldsymbol{\Theta}}=\frac{{{\displaystyle {\sum}_i\boldsymbol{I}}}_i\widehat{{\boldsymbol{\uptheta}}_{\boldsymbol{\upiota}}}}{{{\displaystyle {\sum}_i\boldsymbol{I}}}_i}\kern0.24em \mathrm{and}\;{\mathbf{V}}_{\varTheta }={\left({\displaystyle {\sum}_i{\mathbf{I}}_i}\right)}^{-1} $$

This approach enables tests of the heterogeneity of the lines within selection and control groups, across the entire set of selection and control lines, and between selection and control groups, with respect to the estimated parameters. To assess the heterogeneity of $\widehat{{\boldsymbol{\uptheta}}_k}$ estimates of all three parameters (*r*1_*k*_, *r*2_*k*_*c*_*k*_) in *k* lines we can use the following statistics that is asymptotically distributed as *χ*^2^ with 3(*k*-1) degrees of freedom:

$$ {X}_3^2\left(k-1\right)={\displaystyle \sum_m{\left(\widehat{\varTheta}-\widehat{{\boldsymbol{\uptheta}}_m}\right)}^T{I}_m}\left(\widehat{\varTheta}-\widehat{{\boldsymbol{\uptheta}}_m}\right) $$

To assess heterogeneity of a single parameter *p* in *k* lines the following statistics asymptotically distributed as *χ*^2^ with df = *k*-1 can be used:

$$ {X}_{k-1}^2 = {\displaystyle \sum_m}\frac{{\left(\widehat{\varTheta}-\widehat{{\boldsymbol{\uptheta}}_m}\ \right)}^2}{\upsigma_{pm}^2} $$

where $\widehat{{\boldsymbol{\uptheta}}_k}$ is the ML-estimate of **θ**_*k*_, *σ*_*pk*_^2^ is the squared standard error of parameter *p* in the *k*^th^ line, and $\widehat{\varTheta}$ is the weighted mean of $\widehat{{\boldsymbol{\uptheta}}_k}$. Using this weighted likelihood approach, we can present the total heterogeneity of $\widehat{{\boldsymbol{\uptheta}}_k}$ across all lines of control and selection groups as:

$$ {X^2}_{\mathrm{total}\ \left(\mathrm{control}+\mathrm{selection}\right)} = {X^2}_{\mathrm{within}\ \left(\mathrm{control}\right)}+{X^2}_{\mathrm{within}\ \left(\mathrm{selection}\right)}+{X^2}_{\mathrm{between}\ \left(\mathrm{control}\ \mathrm{v}\mathrm{s}.\ \mathrm{s}\mathrm{election}\right).} $$

Thus, the significance of the difference between selection and control lines can be tested using the statistics:

$$ {X^2}_{\mathrm{between}\ \left(\mathrm{control}\ \mathrm{v}\mathrm{s}.\ \mathrm{s}\mathrm{election}\right)} = {X^2}_{\mathrm{total}\ \left(\mathrm{control}+\mathrm{selection}\right)}\mathit{\hbox{-}}{X^2}_{\mathrm{within}\ \left(\mathrm{control}\right)}\hbox{-} {X^2}_{\mathrm{within}\ \left(\mathrm{selection}\right)} $$

which is distributed approximately as *χ*^2^ with df = 1 upon H_0_{no difference between the compared groups (selection vs. control) for the parameter *p*}.

The importance of using this approach in testing the differences in interference derives from the fact that heterogeneity of recombination rates within the sample (e.g. between replicate lines of the selection group), with positive co-variation of recombination rates in two intervals, may lead to biased upward estimates of *c* and even *c* >1 [63]. Therefore, to reduce the danger of such outcomes while testing for significance between control and selection lines in each of the three experiments, we employed, wherever possible, the weighted ML estimates of recombination (Additional file 3) and interference (Additional files 4 and 5) parameters in weighted likelihood approach, in addition to the standard ML approach (see below). However, where $\widehat{\theta_c}$ , the estimate of *c*, was zero in one or more of the three control or selection lines, its standard error was also zero, thereby overweighting the estimates of *c* from the other two lines and leading to zero weighted average per selection or control. Thus, for all the data we also employed the standard and more direct ML approach allowing for each line, in both selection and control, to have its own *r*1_*k*_ and *r*2_*k*_. Namely, to test for significance of the differences of *c* values in selection and control, we performed log-likelihood ratio test of H_0_ {one global *c* for all selected and control lines} versus H_1_ {two c’s, one for all selected lines and one for all control lines}:

H_1_ : {**Θ**_control_ = (**r**_**1**c_, **r**_2c_, *c*_c_), **Θ**_selection_ = (**r**_1s_, **r**_2s_, *c*_s_)} vs. H_0_ : {**Θ**_control_ = (**r**_1c_, **r**_2c_, *c*), **Θ**_selection_ = (**r**_1c_, **r**_2c_, *c*)}, where pairs of vectors **r**_**1**c_ and **r**_**2**c_ represent the unknown *rf* values for the analyzed pair of intervals for the three control lines, **r**_**1**s_ and **r**_**2**s_ – the vectors of *rf* values for the three selection lines, *c*_*c*_ and *c*_*s*_ – the line-independent values of coefficients of coincidence for control and selection groups, and *c*_*g*_ – the global *c* under the H_0_ assumption that *c*_*s*_ = *c*_*c*_. Therefore, the H_0_ and H_1_ hypotheses are specified by 14 and 13 parameters and the log-likelihood ratio test of H_1_ versus H_0_ is asymptotically distributed as *χ*^2^ with df = 1.

The obtained *P* values (for two-tailed test) were subjected to false discovery rate correction for multiple comparisons before demonstrations in tables, figures and text. For false discovery rate correction, we used a total 48 comparisons across three experiments (with 16 intervals in each) for the recombination rates, while 189 comparisons for the interference estimates.

## Additional files

Additional file 1: Table S1. A review of previous reports on indirect selection for recombination in *Drosophila melanogaster*. **Table S2.** Effect of desiccation selection on the coefficient of coincidence in adjacent and non-adjacent intervals of *D. melanogaster*. **Table S3.** Effect of hypoxia and hyperoxia selection on the coefficient of coincidence in adjacent and non-adjacent intervals of *D. melanogaster*. **Table S4.** Coincidence of interval-pair cases of changes in interference with significant positive correlation between *rf* values in the interval-pairs (a) and with significant increases in *rf* in desiccation, hypoxia, and hyperoxia selection variants (b). (PDF 486 kb) Additional file 2: Figure S1. Effects of selection for tolerance to desiccation, hypoxia, and hyperoxia stresses on recombination rates in *Drosophila melanogaster*: (a) general scheme; (b) a fragment of the flowchart for selected line D1 of the desiccation experiment. **Figure S2.** Marker lines employed in the study. For each chromosome, a separate line was employed, excluding chromosome 2, where two lines (a and b) were used. **Figure S3.** The schematic presentation of hypoxia/hyperoxia selection experiment (PDF 497 kb) Additional file 3:
**Estimates of recombination rates per replicate and entire variants (selection and control).** (PDF 1054 kb) Additional file 4:
**Estimates of the coefficients of coincidence for adjacent intervals per replicate and entire variants (selection and control).** (PDF 2958 kb) Additional file 5:
**Estimates of the coefficients of coincidence for non-adjacent intervals per replicate and entire variants (selection and control).** (PDF 2103 kb) Additional file 6:
**Text S1.** Changes in recombination and interference are not necessarily directly coupled.(PDF 199 kb)

## Abbreviations

*c*: Coefficient of coincidence
LD: Linkage disequilibrium
ML: Maximum likelihood
*rf*: Recombination frequency
