# Supplementary material for: Experimental evolution of recombination and crossover interference in Drosophila caused by directional selection for stress-related traits
Source: BMC Biol. 2015 Nov 27;13:101. doi: 10.1186/s12915-015-0206-5 (PMC4661966; doi:10.1186/s12915-015-0206-5)
Supplement: Additional file 1: Table S1. — A review of previous reports on indirect selection for recombination in Drosophila melanogaster. Table S2. Effect of desiccation selection on the coefficient of coincidence in adjacent and non-adjacent intervals of D. melanogaster. Table S3. Effect of hypoxia and hyperoxia selection on the coefficient of coincidence in adjacent and non-adjacent intervals of D. melanogaster. Table S4. Coincidence of interval-pair cases of changes in interference with significant positive correlation between rf values in the interval-pairs (a) and with significant increases in rf in desiccation, hypoxia, and hyperoxia selection variants (b). (PDF 486 kb) [file 12915_2015_206_MOESM1_ESM.pdf]

Additional file 1

Table S1. A review of previous reports on indirect selection for recombination in *Drosophila melanogaster*

References	Selected trait	Duration (generations)	Markers	Genetic length, cM (no. of intervals)	Changes in *rf* (%)	Changes in Interference
Flexon and Rodell [48]	Resistance to DDT	22-38	X: *w*, *m*, *f*<br>2: *dp*, *cn*, *bw*<br>3: *Gl*, *Sb*	X: 55 (2)<br>2: 92 (2)<br>3: 17 (1)<br>Σ=164 cM (5)	X: –0.7<br>2: 18.9<br>3: 67.4	NA
Korol et al. [12]	Daily temperature fluctuations	~80	X: *ct*, *v*, *f*, *car*<br>2: *al*, *b*, *pr*, *c*, *px*, *sp*<br>3: *rh*, *h*, *th*, *st*, *cu*, *sr*, *e*, *ca*	X: 41 (3)<br>2: 106 (5)<br>3: 101 (7)<br>Σ=248 (15)	X: 19.6<br>2: 25.1<br>3: 32.5	Negative interference for small regions of 2nd and 3rd chromosomes
Korol and Iliadi [51]	Positive geotaxis	~40	X: *y*, *cv*, *v*, *ct*, *car*<br>2: *al*, *b*, *vg*, *bw*<br>3: *ru*, *h*, *cu*, *sr*, *e*	X: 62 (4)<br>2: 104 (3)<br>3: 71 (4)<br>Σ=237 (11)	X: 51.8<br>2: 33.9<br>3: 21.3	NA
--"--	Negative geotaxis	--"--	--"--	--"--	X: 71.7<br>2: –1.5<br>3: 25.7	NA
Rodell et al. [52]	Increased sternopleural bristle number	25	2: *dp*, *cn*, *bw*<br>3: *se*, *ss*, *ro*	2: 92 (2)<br>3: 65 (2)<br>Σ=157 (4)	2: 8.3<br>3: 14.2	NA
--"--	Decreased sternopleural bristle-number	25	--"--	--"--	2: 3. 6<br>3: 0.1	NA
This study	Desiccation tolerance	50	X: *y*, *cv*, *v*, *f*<br>2L: *net*, *dp*, *b*, *pk*, *cn*<br>2R: *cn*, *kn*, *c*, *px*, *sp*<br>3rd: *rh*, *h*, *th*, *cu*, *sr*, *e*	X: 57 (4)<br>2L: 58 (5)<br>2R: 50 (5)<br>3: 71 (6)<br>Σ=234 (16)	X: 33.5<br>2L:12.8<br>2R:25.3<br>3:19.1	Relaxation in positive interference for all the large chromosomes and appearance of negative interference for chromosomes X and 3
This study	Hypoxia tolerance	200	--"--	--"--	X: 33.0<br>2L:24.8<br>2R: –6.6<br>3: 18.7	Relaxation in positive interference for all the large chromosomes and appearance of negative interference for chromosomes X and 2
This study	Hyperoxia tolerance	200	--"--	--"--	X: 29.4<br>2L: 15.0<br>2R: –1.6<br>3: 1.3	Relaxation in positive interference for all the large chromosomes and appearance of negative interference for chromosomes X and 2

NA = not analyzed

Table 2. Effect of desiccation selection on the coefficient of coincidence in adjacent and non-adjacent intervals of *D. melanogaster*

Intervals [a]	Control	*R*	Desiccation	*R*	χ^2_{ML}	p^a	p^b
**X chromosome**							
Adjacent intervals							
y-cv-v	0.564 ± 0.086	-0.35	0.947 ± 0.084	**0.74**	9.39	4.4E-03	8.4E-03
y-cv-f	0.902 ± 0.069	0.01	1.880 ±0.073	0.34	90.27	4.2E-21	1.6E-20
y-v-f	0.852 ± 0.052	**0.71**	1.587 ± 0.041	0.07	115.20	1.4E-26	6.0E-26
cv-v-f	0.698 ± 0.061	0.58	1.395 ± 0.045	0.21	72.75	3.0E-17	1.1E-16
Non-adjacent intervals							
y-cv, v-f	0.986 ± 0.099	-0.02	1.278 ± 0.078	**0.70**	5.25	0.05	0.07
**2L chromosome**							
Adjacent intervals							
net-dp-b	0.352 ± 0.065	**0.71**	0.813 ± 0.069	0.07	21.13	8.6E-06	2.3E-05
net-dp-pk	0.359 ± 0.059	0.40	0.737 ± 0.060	-0.25	17.93	4.6E-05	1.1E-04
net-dp-cn	0.350 ± 0.057	0.25	0.706 ± 0.058	-0.39	17.54	5.6E-05	1.3E-04
net-b-pk	0.192 ± 0.061	-0.70	0.274 ± 0.071	0.03	0.77	0.77	1
net-b-cn	0.154 ± 0.047	-0.68	0.234 ± 0.057	-0.23	1.15	0.56	0.79
dp-b-pk	0.110 ± 0.054	-0.85	0.220 ± 0.075	0.21	1.44	0.46	0.66
dp-b-cn	0.080 ± 0.039	-0.77	0.182 ± 0.059	0.09	2.16	0.25	0.41
net-pk-cn	0.050 ± 0.049	-0.12	0.107 ± 0.074	-0.46	0.43	1	1
Non-adjacent intervals							
net-dp, b-pk	0.376 ± 0.163	-0.58	0.333 ± 0.121	-0.57	0.05	1	1
net-dp, b-cn	0.326 ± 0.129	-0.58	0.281 ± 0.096	-0.64	0.08	1	1
net-dp, pk-cn	0.199 ± 0.196	-0.23	0.134 ± 0.132	-0.42	0.08	1	1
net-b, pk-cn	0.056 ± 0.055	-0.25	0.119 ± 0.082	-0.57	0.41	1	1
**2R chromosome**							
Adjacent intervals							
cn-kn-px	0.377 ± 0.067	0.37	0.905 ± 0.064	**0.85**	26.93	4.2E-07	1.3E-06
cn-kn-sp	0.364 ± 0.059	0.38	0.814 ± 0.055	**0.93**	26.72	4.8E-07	1.5E-06
cn-c-px	0.394 ± 0.065	0.33	0.875 ± 0.062	0.57	24.04	1.9E-06	5.2E-06
cn-c-sp	0.367 ± 0.056	**0.95**	0.780 ± 0.054	**0.69**	24.84	1.2E-06	3.5E-06
cn-px-sp	0.141 ± 0.052	-0.05	0.191 ± 0.058	0.01	0.43	1	1
kn-px-sp	0.056 ± 0.039	-0.18	0.049 ± 0.034	-0.39	0.16	1	1
c-px-sp	0.060 ± 0.042	-0.37	0.052 ± 0.036	-0.22	0.02	1	1
Non-adjacent intervals							
cn-kn, c-px	0.407 ± 0.072	0.35	0.952 ± 0.067	**0.82**	25.10	1.0E-06	3.1E-06
cn-kn, c-sp	0.387 ± 0.062	**0.97**	0.849 ± 0.057	**0.91**	25.19	1.0E-06	3.0E-06
cn-kn, px-sp	0.297 ± 0.129	0.09	0.393 ± 0.118	0.03	0.33	1	1

cn-c, px-sp	0.250 ± 0.109	0.25	0.362 ± 0.109	0.17	0.57	1	1
**<u>3rd chromosome</u>**							
Adjacent intervals							
ru-h-th	0.814 ± 0.099	-0.30	1.101 ± 0.081	0.41	4.68	0.06	0.09
ru-h-cu	0.603 ± 0.074	-0.48	0.997 ± 0.072	0.65	13.63	4.4E-04	9.3E-04
ru-h-sr	0.501 ± 0.058	-0.19	0.757 ± 0.062	-0.12	8.86	5.8E-03	1.1E-02
ru-h-e	0.408 ± 0.049	-0.20	0.669 ± 0.054	-0.54	12.23	9.4E-04	1.9E-03
ru-th-cu	0.082 ± 0.046	-0.34	0.614 ± 0.115	-0.66	18.99	2.6E-05	6.6E-05
ru-th-sr	0.247 ± 0.049	-0.19	0.693 ± 0.069	0.09	26.24	6.0E-07	1.8E-06
ru-th-e	0.200 ± 0.039	-0.00	0.633 ± 0.055	-0.85	38.46	1.1E-09	3.8E-09
h-th-cu	0.109 ± 0.076	0.15	0.680 ± 0.154	-0.38	10.20	2.8E-03	5.4E-03
h-th-sr	0.336 ± 0.083	0.30	1.121 ± 0.107	-0.03	35.27	5.8E-09	1.9E-08
h-th-e	0.332 ± 0.070	0.08	1.059 ± 0.084	-0.62	37.62	1.7E-09	5.8E-09
ru-cu-sr	0.331 ± 0.067	-0.22	0.709 ± 0.081	-0.71	12.48	8.2E-04	1.7E-03
ru-cu-e	0.237 ± 0.046	-0.13	0.614 ± 0.060	-0.63	23.83	2.2E-06	6.1E-06
h-cu-sr	0.406 ± 0.099	0.27	1.317 ± 0.123	-0.21	28.79	1.6E-07	5.1E-07
h-cu-e	0.357 ± 0.073	0.05	1.053 ± 0.088	-0.59	32.23	2.8E-08	8.9E-08
th-cu-sr	0.192 ± 0.134	0.02	0.478 ± 0.206	0.19	1.34	0.50	0.71
th-cu-e	0.180 ± 0.102	0.25	0.356 ± 0.139	0.28	1.01	0.61	0.85
ru-sr-e	0.088 ± 0.043	0.13	0.442 ± 0.080	0.23	14.22	3.2E-04	6.9E-04
h-sr-e	0.233 ± 0.085	-0.60	0.604 ± 0.112	0.10	6.47	2.2E-02	3.7E-02
th-sr-e	0.131 ± 0.091	-0.68	0.211 ± 0.103	0.05	0.33	1	1
cu-sr-e	0.109 ± 0.108	-0.53	0.234 ± 0.133	0.02	0.50	1	1
Non-adjacent intervals							
ru-h, ***th-cu***	0.125 ± 0.071	-0.38	0.694 ± 0.158	0.26	11.34	1.5E-03	3.0E-03
ru-h, th-sr	0.362 ± 0.074	-0.05	0.954 ± 0.101	0.05	21.56	6.8E-06	1.8E-05
ru-h, th-e	0.332 ± 0.061	0.30	0.819 ± 0.079	-0.37	23.02	3.2E-06	8.7E-06
ru-h, cu-sr	0.516 ± 0.113	0.19	1.136 ± 0.133	0.12	11.83	1.2E-03	2.3E-03
ru-h, cu-e	0.414 ± 0.080	0.51	0.916 ± 0.095	-0.32	15.35	1.8E-04	4.0E-04
ru-h, sr-e	0.237 ± 0.103	0.21	0.531 ± 0.127	-0.75	3.04	0.16	0.25
ru-th, cu-sr	0.356 ± 0.076	-0.08	0.731 ± 0.087	-0.46	10.09	3.0E-03	5.8E-03
ru-th, cu-e	0.270 ± 0.053	0.09	0.653 ± 0.064	-0.72	19.61	1.9E-05	4.9E-05
ru-th, sr-e	0.124 ± 0.061	0.25	0.592 ± 0.102	-0.59	13.75	4.2E-04	8.9E-04
ru-cu, sr-e	0.078 ± 0.044	0.19	0.522 ± 0.092	-0.09	17.21	6.6E-05	1.6E-04
h-th, cu-sr	0.485 ± 0.127	0.50	1.558 ± 0.143	-0.18	26.05	6.6E-07	1.9E-06
h-th, cu-e	0.468 ± 0.098	0.40	1.228 ± 0.102	-0.49	24.77	1.3E-06	3.7E-06
h-th, sr-e	0.431 ± 0.156	-0.20	0.798 ± 0.148	-0.61	2.64	0.22	0.30
h-cu, sr-e	0.268 ± 0.106	-0.33	0.714 ± 0.129	-0.76	6.35	2.4E-02	4.0E-02
th-cu, sr-e	0.158 ± 0.157	-0.27	0.149 ± 0.148	0.16	0.00	1	1

[a] - the table includes pairs of "atomic" intervals (**bold font**), "derivative" intervals and their combinations;
R - coefficient of correlation between recombination rates for the considered pair of intervals, adjacent or non-adjacent; *R* values are underlined for cases with significant (at least at p<0.05 level) positive correlation;
χ^2_{ML} –test to assess the significance of difference between coefficients of coincidence in control and selection groups, based on unweighted log-likelihood ratio test. See Material and Methods for the description of the procedure, and Additional files 4 and 5 with calculation details for adjacent and non-adjacent pairs of intervals, receptively.
p^a and p^b are pre-and post-FDR corrected *p*-values for the difference between control and selection estimates.

Table 4. Effect of hypoxia and hyperoxia selection on the coefficient of coincidence in adjacent and non-adjacent intervals of *D. melanogaster*

Intervals [a]	Control	*R*	Hypoxia	*R*	Control versus hypoxia			Hyperoxia	*R*	Control versus hyperoxia		
					χ^2_{ML}	*p* [a]	*p* [b]			χ^2_{ML}	*p* [a]	*p* [b]
**<u>X chromosome</u>**												
**Adjacent intervals**												
y-cv-v	0.712 ± 0.099	0.63	1.108 ± 0.078	0.56	8.88	5.8E-03	1.1E-02	0.883 ± 0.076	-0.30	1.72	0.38	0.55
y-cv-f	1.054 ± 0.074	0.03	2.116 ± 0.062	-0.27	123.52	2.2E-28	9.5E-28	2.093 ± 0.067	-0.43	110.33	1.7E-25	6.8E-25
y-v-f	0.971 ± 0.061	-0.22	1.808 ± 0.052	-0.66	111.15	1.1E-25	4.6E-25	1.785 ± 0.051	0.14	104.15	3.8E-24	1.5E-23
cv-v-f	0.737 ± 0.074	0.14	1.118 ± 0.056	-0.20	16.07	1.2E-04	2.8E-04	1.200 ± 0.058	0.12	22.83	3.6E-06	9.7E-06
**Non-adjacent intervals**												
y-cv, v-f	1.077 ± 0.103	0.23	1.886 ± 0.079	-0.23	36.29	3.4E-09	1.1E-08	1.920 ± 0.088	0.50	37.02	2.4E-09	8.0E-09
**<u>2L chromosome</u>**												
**Adjacent intervals**												
net-dp-b	0.332 ± 0.058	0.14	1.295 ± 0.062	0.04	96.20	2.0E-22	7.9E-22	1.014 ± 0.068	-0.93	49.46	4.0E-12	1.4E-11
net-dp-pk	0.381 ± 0.057	0.09	1.497 ± 0.061	-0.13	139.85	5.8E-32	2.6E-31	1.204 ± 0.068	-0.84	74.97	9.6E-18	3.6E-17
net-dp-cn	0.383 ± 0.055	-0.10	1.510 ± 0.060	0.06	150.22	3.0E-34	1.4E-33	1.207 ± 0.066	-0.73	80.50	5.8E-19	2.2E-18
net-b-pk	0.555 ± 0.082	-0.87	1.675 ± 0.093	-0.32	71.64	5.2E-17	1.9E-16	1.478 ± 0.097	-0.65	48.20	7.6E-12	2.6E-11
net-b-cn	0.496 ± 0.068	-0.81	1.579 ± 0.083	-0.24	89.82	5.2E-21	2.0E-20	1.300 ± 0.086	-0.78	50.72	2.2E-12	7.8E-12
dp-b-pk	0.540 ± 0.097	-0.44	1.195 ± 0.095	-0.51	20.45	1.2E-05	3.2E-05	1.146 ± 0.107	-0.75	16.42	1.0E-04	2.3E-04
dp-b-cn	0.476 ± 0.080	-0.80	1.130 ± 0.084	-0.19	28.19	2.2E-07	6.8E-07	0.969 ± 0.091	-0.73	15.75	1.4E-04	3.2E-04
net-pk-cn	0.318 ± 0.111	0.35	1.285 ± 0.199	-0.01	17.66	5.2E-05	1.3E-04	0.730 ± 0.167	-0.40	4.41	0.07	0.11
dp-pk-cn	0.285 ± 0.121	-0.18	0.878 ± 0.177	**<u>0.82</u>**	7.16	1.5E-02	2.6E-02	0.408 ± 0.142	-0.32	0.43	1	1
b-pk-cn	0.301 ± 0.295	-0.16	0.262 ± 0.258	-0.44	0.01	1	1	0.643 ± 0.442	0.28	0.41	1	1
**Non-Adjacent intervals**												
net-dp, b-pk	0.588 ± 0.167	-0.19	1.563 ± 0.182	-0.21	13.52	4.8E-04	1.0E-03	1.403 ± 0.215	**<u>0.67</u>**	8.81	6.0E-03	1.1E-02
net-dp, b-cn	0.609 ± 0.147	-0.26	1.556 ± 0.160	0.04	16.50	9.8E-05	2.3E-04	1.455 ± 0.190	0.62	11.95	1.1E-03	2.2E-03
net-dp, pk-cn	0.647 ± 0.309	-0.18	1.470 ± 0.362	0.04	2.59	0.22	0.33	1.798 ± 0.439	0.31	4.26	7.8E-02	0.12
net-b, pk-cn	0.398 ± 0.128	-0.03	1.563 ± 0.187	-0.10	10.72	2.2E-03	4.3E-03	0.599 ± 0.156	-0.63	1.06	0.60	0.83
dp-b, pk-cn	0.394 ± 0.150	-0.69	0.837 ± 0.188	0.49	3.42	0.13	0.20	0.421 ± 0.156	-0.42	0.19	1	1
**<u>2R chromosome</u>**												
**Adjacent intervals**												
cn-kn-px	0.387 ± 0.075	-0.14	0.342 ± 0.074	0.50	0.20	1	1	0.487 ± 0.084	0.28	0.75	0.78	1
cn-kn-sp	0.434 ± 0.069	0.16	0.440 ± 0.072	0.43	0.00	1	1	0.720 ± 0.084	0.35	6.94	1.7E-02	2.9E-02
cn-c-px	0.376 ± 0.070	0.66	0.332 ± 0.070	0.18	0.20	1	1	0.515 ± 0.079	0.48	1.67	0.40	0.58
cn-c-sp	0.417 ± 0.064	0.29	0.414 ± 0.067	0.30	0.00	1	1	0.697 ± 0.077	0.54	7.92	9.8E-03	1.8E-02
kn-c-px	0.257 ± 0.143	0.66	0.235 ± 0.159	-0.28	0.01	1	1	0.466 ± 0.178	0.21	0.84	0.72	0.97
kn-c-sp	0.272 ± 0.129	0.29	0.179 ± 0.124	0.22	0.24	1	1	0.422 ± 0.148	0.02	0.61	0.86	1
cn-px-sp	0.373 ± 0.073	0.03	0.404 ± 0.081	-0.01	0.08	1	1	0.659 ± 0.092	-0.13	6.05	2.8E-02	4.6E-02
kn-px-sp	0.286 ± 0.075	0.17	0.237 ± 0.076	0.06	0.22	1	1	0.315 ± 0.080	0.10	0.07	1	1
c-px-sp	0.283 ± 0.078	-0.27	0.253 ± 0.081	-0.05	0.08	1	1	0.316 ± 0.083	-0.35	0.08	1	1
**Non-Adjacent intervals**												

cn-kn, c-px	0.398 ± 0.079	0.15	0.346 ± 0.077	0.35	0.23	1	1	0.527 ± 0.090	0.44	1.11	0.58	0.81
cn-kn, c-sp	0.444 ± 0.072	0.11	0.449 ± 0.075	0.43	0.00	1	1	0.764 ± 0.089	0.55	7.81	1.0E-02	1.9E-02
cn-kn, px-sp	0.531 ± 0.169	-0.57	0.721 ± 0.195	0.20	0.52	0.94	1	1.365 ± 0.245	-0.38	7.78	1.1E-02	1.9E-02
cn-c, px-sp	0.491 ± 0.148	-0.53	0.623 ± 0.169	0.16	0.33	1	1	1.144 ± 0.202	-0.27	6.70	1.9E-02	3.3E-02
**3rd chromosome**												
**Adjacent intervals**												
ru-h-th	0.495 ± 0.103	0.54	0.995 ± 0.129	0.65	8.97	5.6E-03	1.0E-02	0.396 ± 0.097	-0.15	0.44	1	1
ru-h-cu	0.396 ± 0.077	0.59	0.695 ± 0.092	0.58	6.04	2.8E-02	4.6E-02	0.331 ± 0.074	-0.28	0.35	1	1
ru-h-sr	0.334 ± 0.061	**0.69**	0.717 ± 0.077	0.48	14.59	2.6E-04	5.7E-04	0.278 ± 0.057	-0.50	0.42	1	1
ru-h-e	0.313 ± 0.054	**0.80**	0.639 ±0.065	0.49	14.31	3.2E-04	6.9E-04	0.265 ± 0.051	-0.59	0.39	1	1
ru-th-cu	0.163 ± 0.071	0.36	0.327 ± 0.089	**0.74**	1.97	3.2E-01	0.47	0.259 ± 0.088	0.35	0.73	0.78	1
ru-th-sr	0.140 ± 0.045	0.31	0.531 ± 0.071	**0.75**	19.57	1.9E-05	4.9E-05	0.203 ± 0.052	0.17	0.87	0.70	0.95
ru-th-e	0.155 ± 0.040	0.33	0.453 ± 0.055	0.48	17.61	5.4E-05	1.3E-04	0.190 ± 0.043	0.02	0.37	1	1
h-th-cu	0.137 ± 0.095	0.51	0.598 ± 0.162	**0.84**	5.50	3.8E-02	0.06	0.342 ± 0.148	**0.76**	1.42	0.46	0.60
h-th-sr	0.098 ± 0.056	0.39	0.552 ± 0.102	**0.95**	13.45	5.0E-04	1.0E-03	0.246 ± 0.084	**0.67**	2.24	0.26	0.39
h-th-e	0.116 ± 0.051	0.37	0.438 ± 0.076	**0.78**	10.92	1.9E-03	3.7E-03	0.201 ± 0.065	0.26	1.09	0.60	0.83
ru-cu-sr	0.122 ± 0.053	0.32	0.606 ±0.089	0.57	18.85	2.8E-05	7.0E-05	0.133 ± 0.053	-0.32	0.02	1	1
ru-cu-e	0.139 ± 0.043	0.46	0.458 ± 0.059	0.38	17.05	7.2E-05	1.7E-04	0.131 ± 0.040	0.58	0.02	1	1
h-cu-sr	0.085 ± 0.059	0.39	0.452 ± 0.103	**0.72**	8.16	8.6E-03	1.6E-02	0.117 ± 0.066	**0.78**	0.14	1	1
h-cu-e	0.098 ± 0.048	0.48	0.339 ± 0.068	0.64	7.46	1.2E-02	2.2E-02	0.092 ± 0.046	0.50	0.00	1	1
ru-sr-e	0.137 ± 0.060	0.56	0.319 ± 0.070	0.22	3.68	0.11	0.17	0.130 ± 0.057	0.06	0.00	1	1
h-sr-e	0.085 ± 0.059	0.56	0.288 ± 0.079	0.38	3.63	0.12	0.18	0.080 ± 0.056	**0.78**	0.00	1	1
**Non-Adjacent intervals**												
ru-h, th-cu	0.164 ± 0.093	0.62	0.088 ± 0.062	**0.80**	0.49	0.96	1	0.167 ± 0.095	-0.34	0.00	1	1
ru-h, th-sr	0.157 ± 0.063	0.66	0.411 ± 0.084	**0.71**	5.48	3.8E-02	0.06	0.200 ± 0.068	-0.47	0.23	1	1
ru-h, th-e	0.168 ± 0.054	**0.75**	0.402 ± 0.069	0.61	6.65	1.9E-02	3.4E-02	0.199 ± 0.058	-0.65	0.17	1	1
ru-h, cu-sr	0.147 ± 0.084	0.50	0.641 ± 0.136	0.54	8.42	7.4E-03	1.4E-02	0.227 ± 0.099	-0.35	0.39	1	1
ru-h, cu-e	0.168 ± 0.067	0.00	0.527 ± 0.093	0.44	8.82	6.0E-03	1.1E-02	0.215 ± 0.074	-0.87	0.24	1	1
ru-h, sr-e	0.196 ± 0.111	0.78	0.338 ± 0.115	0.25	0.76	0.76	1	0.195 ± 0.110	-0.76	0.00	1	1
ru-th, cu-sr	0.117 ± 0.057	0.07	0.665 ± 0.104	0.54	18.49	3.4E-05	8.4E-05	0.159 ± 0.063	-0.41	0.25	1	1
ru-th, cu-e	0.150 ± 0.048	0.21	0.493 ± 0.069	0.23	14.99	2.2E-04	4.8E-04	0.157 ± 0.048	-0.41	0.01	1	1
ru-th, sr-e	0.195 ± 0.084	0.30	0.338 ± 0.089	-0.23	1.36	0.48	0.68	0.227 ± 0.089	-0.26	0.08	1	1
ru-cu, sr-e	0.163 ± 0.071	0.48	0.316 ± 0.077	0.03	2.09	0.32	0.45	0.190 ± 0.075	0.09	0.08	1	1
h-th, cu-sr	0.061 ± 0.061	0.09	0.495 ± 0.130	**0.82**	7.72	1.1E-02	1.9E-02	0.168 ± 0.095	-0.28	0.91	0.68	0.92
h-th, cu-e	0.105 ± 0.059	0.19	0.352 ± 0.084	0.59	5.12	0.05	0.08	0.133 ± 0.065	0.36	0.11	1	1
h-th, sr-e	0.163 ± 0.114	0.25	0.333 ± 0.122	0.11	0.97	0.64	0.87	0.240 ± 0.136	0.53	0.19	1	1
h-cu, sr-e	0.114 ± 0.079	0.45	0.300 ± 0.096	0.34	2.0	0.32	0.47	0.167 ± 0.095	**0.70**	0.19	1	1

[a] - the table includes pairs of “atomic” intervals (bold font), “derivative” intervals and their combinations;
R - coefficient of correlation between recombination rates for the considered pair of intervals, adjacent or non-adjacent;
R values are underlined for cases with significant (at least at p<0.05 level) positive correlation;
χ^2_{ML} –test to assess the significance of difference between coefficients of coincidence in control and selection variants, based on unweighted log-likelihood ratio test. See Material and Methods for the description of the procedure, and Additional files 4 and 5 with calculation details for adjacent and non-adjacent pairs of intervals, receptively.
p^a and p^b are pre-and post-FDR corrected *p*-values for the difference between control and selection estimates.

Table S4. Coincidence of interval-pair cases of changes in interference with significant positive correlation between *rf* values in the interval-pairs (a); and with significant increase in *rf* in one interval (b); or both the intervals (c); in selection groups.

			Variant with significant relaxation/negative interference compared to control								
			Desiccation			Hypoxia			Hyperoxia		
**(a)**	Significant positive		Yes	No	Σ	Yes	No	Σ	Yes	No	Σ
	correlation between *rf*	Yes	6	1	7	6	4	10	1	5	6
	values in the considered	No	34	22	56	29	24	53	18	39	57
	interval pairs	Σ	40	23	63	35	28	63	19	44	63
	p (two-tailed test)				0.41			1.00			0.66
**(b)**	Significant increase	Yes	19	11	30	23	9	32	4	1	5
	of *rf* in only one	No	21	22	43	12	19	31	15	43	58
	interval	Σ	40	23	63	35	28	63	19	44	63
	p (two-tailed test)				0.24			0.011			0.025
**(c)**	Significant increase	Yes	8	0	8	5	1	6	3	1	4
	of *rf* in both	No	32	23	55	30	27	57	16	43	59
	intervals	Σ	40	23	63	35	28	63	19	44	63
	p (two-tailed test)				0.023			0.21			0.08

The contingency 2×2 tables represent the distribution of interval pairs with respect to two alternatives: significant (Yes) vs. non-significant (No) changes in interference (the columns) and (a) significant positive correlation (Yes vs. No) between *rf* values in the considered interval pairs (rows); significant (Yes) vs. non-significant (No) increase in *rf* in either one (b); or both of the considered intervals (c).
