# Supplementary material for: Experimental evolution of recombination and crossover interference in Drosophila caused by directional selection for stress-related traits
Source: BMC Biol. 2015 Nov 27;13:101. doi: 10.1186/s12915-015-0206-5 (PMC4661966; doi:10.1186/s12915-015-0206-5)
Supplement: Additional file 2: Figure S1. — Effects of selection for tolerance to desiccation, hypoxia, and hyperoxia stresses on recombination rates in Drosophila melanogaster: (a) general scheme; (b) a fragment of the flowchart for selected line D1 of the desiccation experiment. Figure S2. Marker lines employed in the study. For each chromosome, a separate line was employed, excluding chromosome 2, where two lines (a and b) were used. Figure S3. The schematic presentation of hypoxia/hyperoxia selection experiment (PDF 497 kb) [file 12915_2015_206_MOESM2_ESM.pdf]

Additional file 2

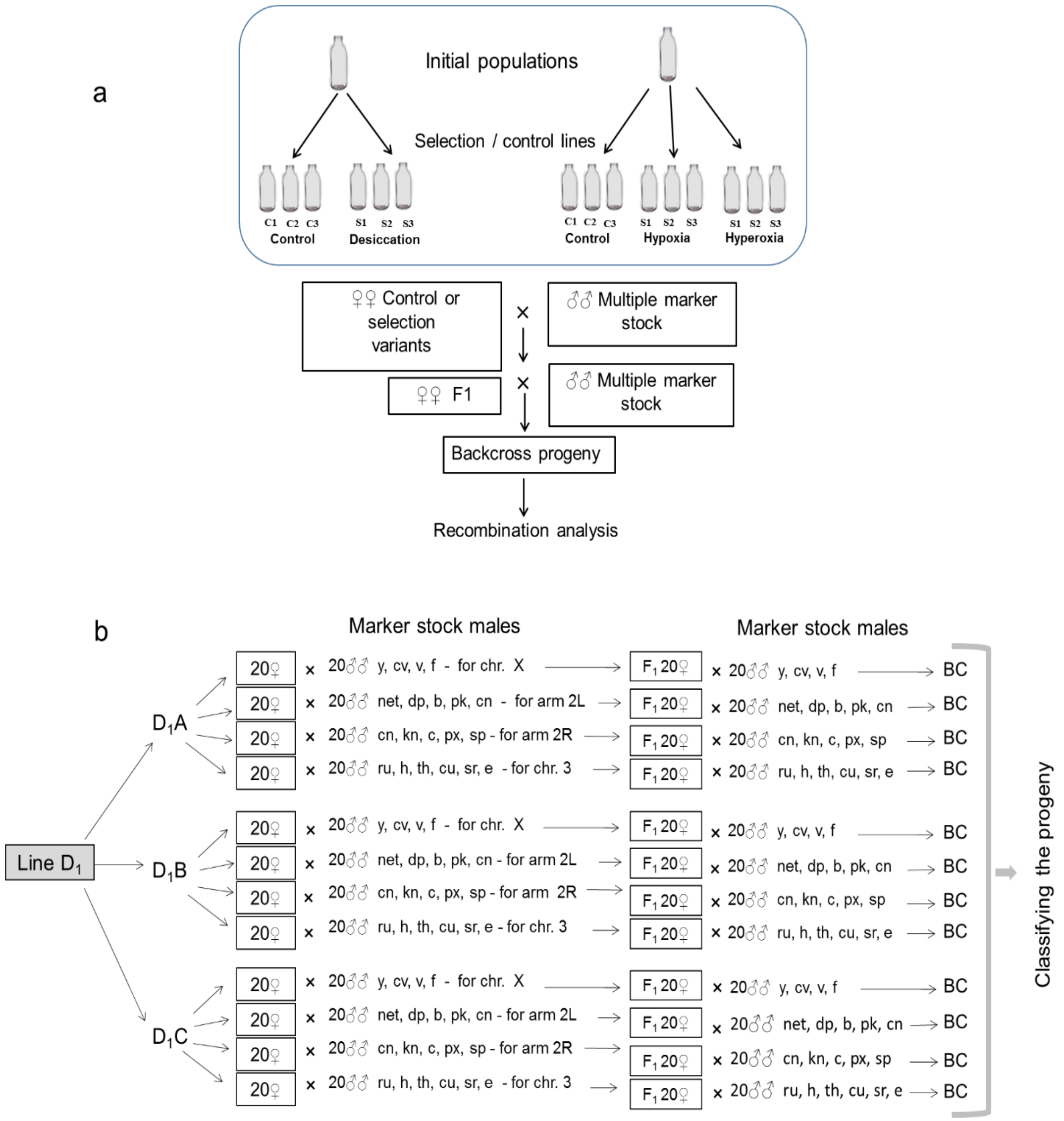

Figure S1

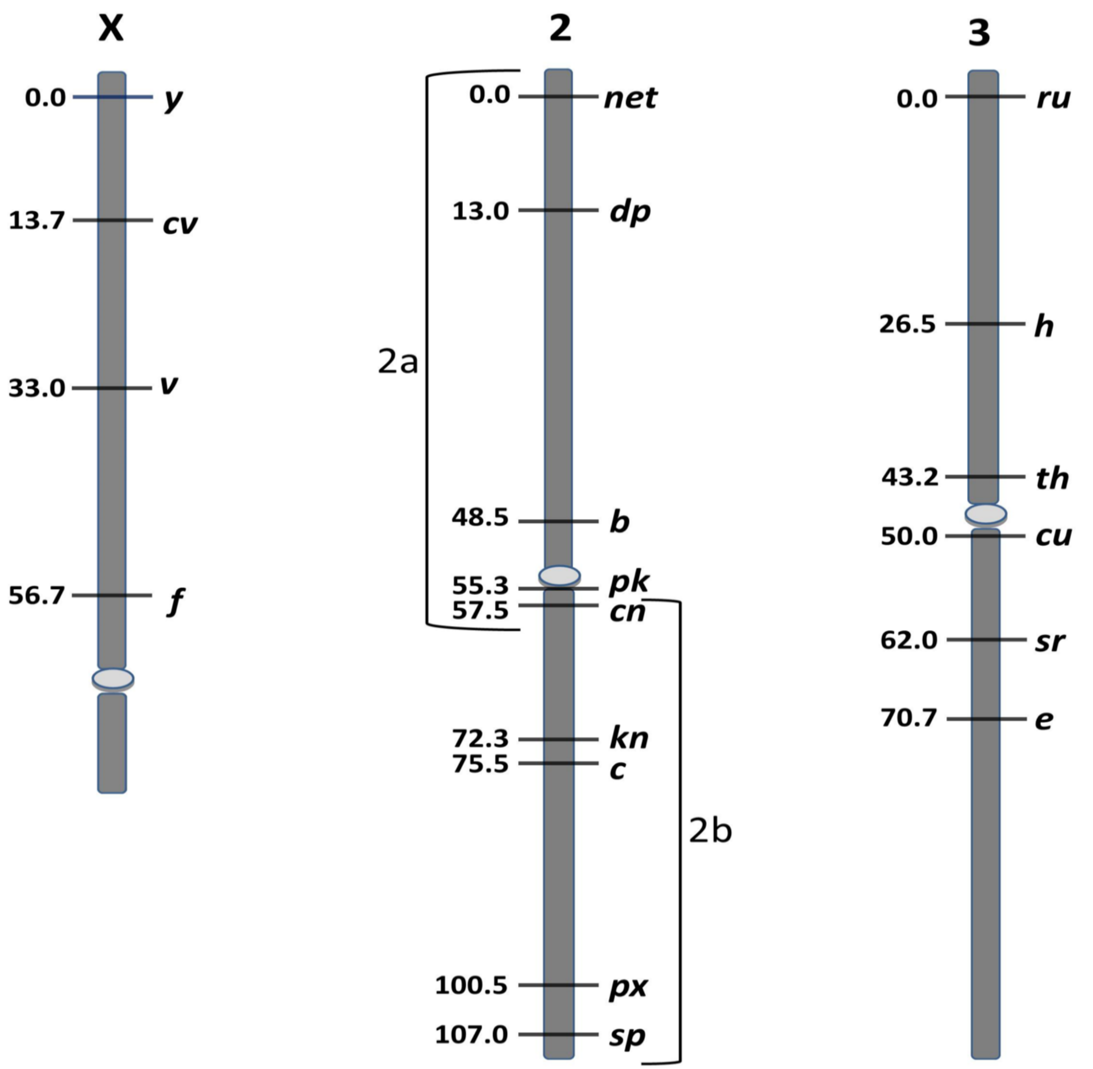
X
0.0 y
13.7 cv
33.0 v
56.7 f
2
0.0 net
13.0 dp
48.5 b
55.3 pk
57.5 cn
72.3 kn
75.5 c
100.5 px
107.0 sp
2a
2b
3
0.0 ru
26.5 h
43.2 th
50.0 cu
62.0 sr
70.7 e

Figure S2

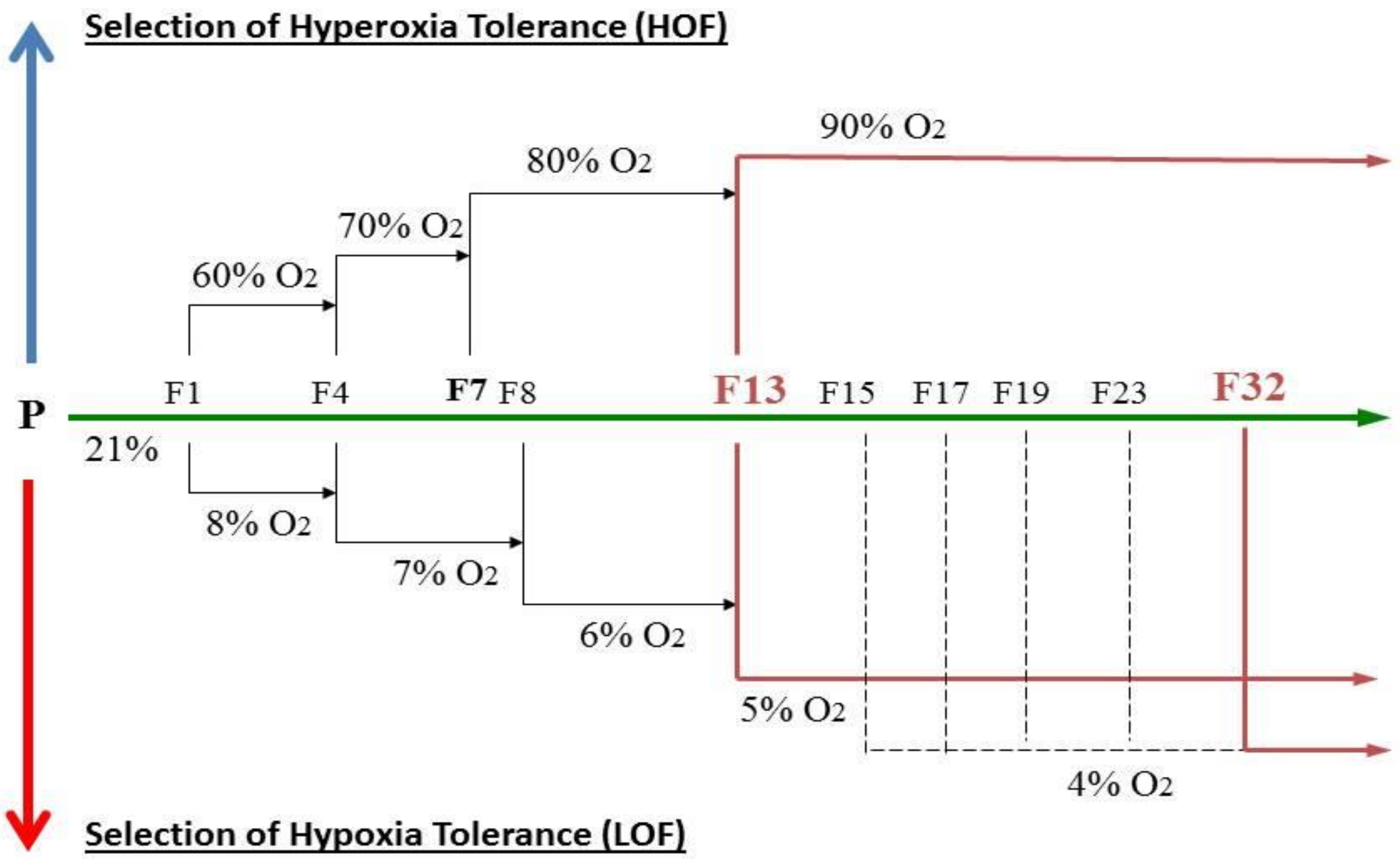
Selection of Hyperoxia Tolerance (HOF)
90% O2
80% O2
70% O2
60% O2
P
F1
F4
F7
F8
F13
F15
F17
F19
F23
F32
21%
8% O2
7% O2
6% O2
5% O2
4% O2
Selection of Hypoxia Tolerance (LOF)

Figure S3

Legends to supplementary Figures

Figure S1. Analyzing the effect of selection for tolerance to desiccation, hypoxia and hyperoxia stresses on recombination in *Drosophila melanogaster*: (a) general scheme; (b) a fragment of the flowchart for selected line D1 of the desiccation experiment.

Figure S2. Marker lines employed in the study. For each chromosome a separate line was employed, excluding chromosome 2, where two lines (a and b) were used.

Figure S3. Selection for hypoxia/hyperoxia tolerance was initiated after crossing 27 isofemale *D. melanogaster* lines. The feasibility and tolerance capacity of the F_1 progeny of the parental cross to different O_2 concentrations was analyzed in the trial experiments. After 7 generations of selection, hyperoxia tolerance was increased to 80% O_2, and after 13 generations of selection, the hypoxia tolerance in the hypoxia-selected flies (i.e., low oxygen flies (LOF)) reached 5%, a level of hypoxia that is lethal for most of the control flies. The hyperoxia-selected flies (i.e., high oxygen flies (HOF)) broke through the lethal hyperoxic level (90% O_2) after 13 generations of selection, and the LOF flies exhibited tolerance to a severe level of hypoxia (i.e., 4% O_2, embryonic lethal to control flies) following 32 generations of selection.
