# Supplementary material for: Experimental evolution of recombination and crossover interference in Drosophila caused by directional selection for stress-related traits
Source: BMC Biol. 2015 Nov 27;13:101. doi: 10.1186/s12915-015-0206-5 (PMC4661966; doi:10.1186/s12915-015-0206-5)
Supplement: Additional file 3: — Estimates of recombination rates per replicate and entire variants (selection and control). (PDF 1054 kb) [file 12915_2015_206_MOESM3_ESM.pdf]

Additional file 3

Estimates of recombination frequencies and their heterogeneity for replicate lines of control and selection variants

For each experiment (desiccation, hypoxia, hyperoxia), corresponding unweighted ML-estimates of recombination frequency (teta) for each replicate line and its standard error (SE) are shown. For the entire Control and Selection variants, weighted estimates and corresponding SE and chi-square for between-replicates heterogeneity are provided as described in Materials and Methods. In addition, chi-square for Control vs. Selection difference is calculated as chi^2(ctrl+sel)-chi^2(sel)-chi^2(ctrl) (see SI text Materials and Methods).

…………………………………………………………………………………………..

Desiccation experiment

X chromosome

markers 1-2 (*y-cv*)

	Control	Desiccation
**Line 1**		
Teta	0.1008	0.1223
SE	0.0093	0.0101
**Line 2**		
Teta	0.1060	0.1139
SE	0.0095	0.0098
**Line 3**		
Teta	0.0999	0.0849
SE	0.0093	0.0086
Teta 3×1050	0.1028	0.1068
SE	0.0054	0.0055
chi^2:	0.3848	8.5158

Teta Sel+Ctrl 6×1050 0.1047
chi^2: 9.1687
chi^2(ctrl+sel)-chi^2(sel)-chi^2(ctrl): 0.2681

---

markers 1-3 (*y-v*)

	Control	Desiccation
**Line 1**		
Teta	0.2640	0.3257
SE	0.0136	0.0145
**Line 2**		
Teta	0.2765	0.3373
SE	0.0138	0.0146
**Line 3**		
Teta	0.2894	0.2921
SE	0.0140	0.0140

	Control	Desiccation
Teta 3×1050	0.2769	0.3184
SE	0.0080	0.0083
chi^2:	1.6085	5.2586

Teta Sel+Ctrl 6×1050 0.2968
chi^2: 19.8910
chi^2(ctrl+sel)-chi^2(sel)-chi^2(ctrl): 13.0240

---

markers 1-4 (*y-f*)

	**Control**	**Desiccation**
**Line 1**		
Teta	0.3836	0.2983
SE	0.0150	0.0140
**Line 2**		
Teta	0.3854	0.3322
SE	0.0150	0.0145
**Line 3**		
Teta	0.4068	0.3064
SE	0.0151	0.0142
Teta 3X1050	0.3926	0.3146
SE	0.0087	0.0083
chi^2:	1.4635	2.5228

Teta Sel+Ctrl 6X1050 0.3517
chi^2: 46.1850
chi^2(ctrl+sel)-chi^2(sel)-chi^2(ctrl): 42.1987

---

markers 2-3 (*cv-v*)

	**Control**	**Desiccation**
**Line 1**		
Teta	0.1859	0.2719
SE	0.0120	0.0137
**Line 2**		
Teta	0.1958	0.2722
SE	0.0122	0.0137
**Line 3**		
Teta	0.2056	0.2469
SE	0.0125	0.0133
Teta 3×1050	0.1969	0.2644
SE	0.0071	0.0079
chi^2:	1.3446	2.4308

Teta Sel+Ctrl 6×1050 0.2272
chi^2: 44.5137
chi^2(ctrl+sel)-chi^2(sel)-chi^2(ctrl): 40.7382

---

markers 2-4 (*cv-f*)

	Control	Desiccation
**Line 1**		
Teta	0.3482	0.3647
SE	0.0146	0.0149
**Line 2**		
Teta	0.3520	0.3597
SE	0.0147	0.0148
**Line 3**		
Teta	0.3645	0.3214
SE	0.0148	0.0144
Teta 3×1050	0.3558	0.3484
SE	0.0085	0.0085
chi^2:	0.5485	5.2032

Teta Sel+Ctrl 6×1050 0.3521
chi^2: 6.1357
chi^2(ctrl+sel)-chi^2(sel)-chi^2(ctrl): 0.3841
----

markers 3-4 (*v-f*)

	Control	Desiccation
**Line 1**		
Teta	0.2041	0.3422
SE	0.0124	0.0146
**Line 2**		
Teta	0.2217	0.3095
SE	0.0128	0.0143
**Line 3**		
Teta	0.2228	0.3122
SE	0.0128	0.0143
Teta 3×1050	0.2189	0.3212
SE	0.0074	0.0083
chi^2:	0.8593	3.2358

Teta Sel+Ctrl 6×1050 0.2639
chi^2: 88.9578
chi^2(ctrl+sel)-chi^2(sel)-chi^2(ctrl): 84.8626

2L chromosome

markers 1-2 (*net-dp*)

	Control	Desiccation
**Line 1**		
teta	0.1148	0.1584
SE	0.0116	0.0133
**Line 2**		
teta	0.1049	0.1737
SE	0.0112	0.0138
**Line 3**		
teta	0.0974	0.1629
SE	0.0108	0.0134

teta 3×750	0.1070	0.1692
SE	0.0065	0.0079
chi^2:	1.5838	0.5226

teta ctrl+sel 6×750 0.1322
chi^2: 38.9603
chi^2(ctrl+sel)-chi^2(sel)-chi^2(ctrl): 36.8538

<u>markers 1-3 *(net-b)*</u>

	Control	Desiccation
**Line 1**		
Teta	0.3898	0.3932
SE	0.0178	0.0178
**Line 2**		
Teta	0.3794	0.3650
SE	0.0177	0.0175
**Line 3**		
Teta	0.3666	0.3865
SE	0.0176	0.0178
Teta 3×750	0.3790	0.3821
SE	0.0102	0.0102
chi^2:	0.9233	1.2318

Teta Sel+Ctrl 6×750 0.3805
chi^2: 2.2004
chi^2(ctrl+sel)-chi^2(sel)-chi^2(ctrl): 0.0453

---

markers 1-4 (*net-pk*)

	Control	Desiccation
**Line 1**	0.4291	0.4278
Teta	0.0181	0.0181
SE		
**Line 2**		
Teta	0.4341	0.4108
SE	0.0181	0.0179
**Line 3**		
Teta	0.4147	0.4372
SE	0.0180	0.0181
Teta 3×750	0.4262	0.4257
SE	0.0104	0.0104
chi^2:	0.6599	1.0098

Teta Sel+Ctrl 6×750 0.4259
chi^2: 1.6706
chi^2(ctrl+sel)-chi^2(sel)-chi^2(ctrl): 0.0010

---

markers 1-5 (*net-cn*)

	Control	Desiccation
Line 1		
Teta	0.4479	0.4466
SE	0.0182	0.0181
Line 2		
Teta	0.4568	0.4287
SE	0.0181	0.0180
Line 3		
Teta	0.4333	0.4545
SE	0.0181	0.0182
Teta 3X750	0.4462	0.4435
SE	0.0105	0.0105
chi^2:	0.8900	1.0220

Teta Sel+Ctrl 6X750 0.4448
chi^2: 1.9447
chi^2(ctrl+sel)-chi^2(sel)-chi^2(ctrl): 0.0326
----

markers 2-3 (*dp-b*)

	Control	Desiccation
**Line 1**		
Teta	0.3029	0.2981
SE	0.0168	0.0167
**Line 2**		
Teta	0.2921	0.2864
SE	0.0166	0.0165
**Line 3**		
Teta	0.2839	0.2943
SE	0.0165	0.0166
Teta 3×750	0.2936	0.2937
SE	0.0096	0.0096
chi^2:	0.7235	0.2105

Teta Sel+Ctrl 6×750 0.2936
chi^2: 0.9341
chi^2(ctrl+sel)-chi^2(sel)-chi^2(ctrl): 0.0000
----

markers 2-4 (*dp-pk*)

	Control	Desiccation
**Line 1**		
Teta	0.3452	0.3411
SE	0.0174	0.0173
**Line 2**		
Teta	0.3529	0.3368
SE	0.0174	0.0172

Line 3
Teta 0.3373 0.3450
SE 0.0173 0.0173

Teta 3×750 0.3453 0.3418
SE 0.0100 0.0100
chi^2: 0.4248 0.0750

Teta Sel+Ctrl 6×750 0.3435
chi^2: 0.5612
chi^2(ctrl+sel)-chi^2(sel)-chi^2(ctrl): 0.0614
----

markers 2-5 (*dp-cn*)

Control **Desiccation**
Line 1 0.3639 0.3625
Teta 0.0176 0.0175
SE
Line 2
Teta 0.3754 0.3545
SE 0.0177 0.0174
Line 3
Teta 0.3587 0.3622
SE 0.0175 0.0175

Teta 3×750 0.3662 0.3604
SE 0.0102 0.0101
chi^2: 0.5077 0.0967

Teta Sel+Ctrl 6×750 0.3633
chi^2: 0.7641
chi^2(ctrl+sel)-chi^2(sel)-chi^2(ctrl): 0.1597
----

markers 3-4 (*b-pk*)

Control **Desiccation**
Line 1
Teta 0.0422 0.0553
SE 0.0073 0.0083
Line 2
Teta 0.0578 0.0480
SE 0.0085 0.0078
Line 3
Teta 0.0558 0.0545
SE 0.0084 0.0083

Teta 3×750 0.0544 0.0549
SE 0.0048 0.0048
chi^2: 1.9170 0.4893

Teta Sel+Ctrl 6×750 0.0546
chi^2: 2.4132
chi^2(ctrl+sel)-chi^2(sel)-chi^2(ctrl): 0.0070

----

markers 3-5 (*b-cn*)

	Control	Desiccation
**Line 1**		
Teta	0.0573	0.0799
SE	0.0084	0.0099
**Line 2**		
Teta	0.0840	0.0648
SE	0.0101	0.0090
**Line 3**		
Teta	0.0761	0.0706
SE	0.0097	0.0093
Teta 3×750	0.0752	0.0744
SE	0.0056	0.0055
chi^2:	2.2301	0.8316

Teta Sel+Ctrl 6×750 0.0748
chi^2: 3.0723
chi^2(ctrl+sel)-chi^2(sel)-chi^2(ctrl): 0.0106
----

markers 4-5 (*pk-cn*)

	Control	Desiccation
**Line 1**		
Teta	0.0143	0.0229
SE	0.0043	0.0055
**Line 2**		
Teta	0.0209	0.0149
SE	0.0052	0.0044
**Line 3**		
Teta	0.0192	0.0160
SE	0.0050	0.0046
Teta 3×750	0.0208	0.0191
SE	0.0030	0.0029
chi^2:	0.3143	1.0436

Teta Sel+Ctrl 6×750 0.0199
chi^2: 1.5136
chi^2(ctrl+sel)-chi^2(sel)-chi^2(ctrl): 0.1557

2R chromosome

markers 1-2 (*cn-kn*)

	Control	Desiccation
**Line 1**		
Teta	0.1292	0.2133

SE 0.0122 0.0150
Line 2
Teta 0.1027 0.1750
SE 0.0111 0.0139
Line 3
Teta 0.1265 0.1834
SE 0.0121 0.0141

Teta 3×750 0.1211 0.1901
SE 0.0069 0.0083
chi^2: 3.3731 3.6263

Teta Sel+Ctrl 6×750 0.1493
chi^2: 48.2613
chi^2(ctrl+sel)-chi^2(sel)-chi^2(ctrl): 41.2619
----

markers 1-3 (*cn-c*)

	Control	Desiccation
**Line 1**		
Teta	0.1597	0.2280
SE	0.0133	0.0153
**Line 2**		
Teta	0.1174	0.1960
SE	0.0117	0.0145
**Line 3**		
Teta	0.1464	0.1982
SE	0.0129	0.0146

Teta 3×750 0.1426 0.2073
SE 0.0074 0.0085
chi^2: 7.1884 2.6473

Teta Sel+Ctrl 6×750 0.1702
chi^2: 42.7817
chi^2(ctrl+sel)-chi^2(sel)-chi^2(ctrl): 32.9460
----

markers 1-4 (*cn-px)*

	Control	Desiccation
**Line 1**		
Teta	0.3571	0.4218
SE	0.0174	0.0180
**Line 2**		
Teta	0.3492	0.3719
SE	0.0173	0.0176
**Line 3**		
Teta	0.3635	0.3840
SE	0.0175	0.0178

Teta 3×750 0.3586 0.3925
SE 0.0101 0.0103
chi^2: 0.2441 4.3752

Teta Sel+Ctrl 6×750 0.3753
chi^2: 10.1315
chi^2(ctrl+sel)-chi^2(sel)-chi^2(ctrl): 5.5122
----

markers 1-5 (*cn-sp*)

	Control	Desiccation
Line 1		
Teta	0.4293	0.4650
SE	0.0180	0.0181
Line 2		
Teta	0.4014	0.4266
SE	0.0178	0.0181
Line 3		
Teta	0.4073	0.4372
SE	0.0179	0.0181

Teta 3X750	0.4137	0.4430
SE	0.0104	0.0105
chi^2:	1.3610	2.4194

Teta Sel+Ctrl 6X750 0.4282
chi^2: 7.7366
chi^2(ctrl+sel)-chi^2(sel)-chi^2(ctrl): 3.9562
----

markers 2-3 (*kn-c*)

	Control	Desiccation
**Line 1**		
Teta	0.0274	0.0144
SE	0.0059	0.0044
**Line 2**		
Teta	0.0144	0.0157
SE	0.0044	0.0045
**Line 3**		
Teta	0.0180	0.0160
SE	0.0049	0.0046

Teta 3×750	0.0203	0.0174
SE	0.0030	0.0028
chi^2:	5.6042	0.9436

Teta Sel+Ctrl 6×750 0.0187
chi^2: 7.0685
chi^2(ctrl+sel)-chi^2(sel)-chi^2(ctrl): 0.5207
----

markers 2-4 (*kn-px*)

	Control	Desiccation
**Line 1**		
Teta	0.2566	0.3371

SE	0.0159	0.0173
**Line 2**		
Teta	0.2548	0.2839
SE	0.0158	0.0165
**Line 3**		
Teta	0.2609	0.3036
SE	0.0160	0.0168
Teta 3×750	0.2604	0.3076
SE	0.0093	0.0097
chi^2:	0.0046	5.0663

Teta Sel+Ctrl 6×750 0.2829
chi^2: 17.4193
chi^2(ctrl+sel)-chi^2(sel)-chi^2(ctrl): 12.3483

<u>markers 2-5 (*kn-sp*)</u>

	**Control**	**Desiccation**
**Line 1**		
Teta	0.3288	0.3879
SE	0.0171	0.0178
**Line 2**		
Teta	0.3155	0.3490
SE	0.0169	0.0174
**Line 3**		
Teta	0.3105	0.3598
SE	0.0169	0.0175
Teta 3×750	0.3199	0.3655
SE	0.0098	0.0101
chi^2:	0.6966	2.5598

Teta Sel+Ctrl 6×750 0.3420
chi^2: 13.6698
chi^2(ctrl+sel)-chi^2(sel)-chi^2(ctrl): 10.4133

<u>markers 3-4 (*c-px*)</u>

	**Control**	**Desiccation**
**Line 1**		
Teta	0.2333	0.3224
SE	0.0154	0.0171
**Line 2**		
Teta	0.2401	0.2623
SE	0.0155	0.0161
**Line 3**		
Teta	0.2404	0.2859
SE	0.0156	0.0165
Teta 3×750	0.2413	0.2895
SE	0.0090	0.0095
chi^2:	0.1311	6.5927

Teta Sel+Ctrl 6×750 0.2640
chi^2: 20.1649
chi^2(ctrl+sel)-chi^2(sel)-chi^2(ctrl): 13.4411
----

markers 3-5 (*c-sp*)

	Control	Desiccation
**Line 1**		
Teta	0.3061	0.3731
SE	0.0168	0.0177
**Line 2**		
Teta	0.3007	0.3274
SE	0.0167	0.0171
**Line 3**		
Teta	0.2903	0.3426
SE	0.0166	0.0173
Teta 3×750	0.3008	0.3475
SE	0.0097	0.0100
chi^2:	0.5922	3.5110

Teta Sel+Ctrl 6×750 0.3233
chi^2: 15.3670
chi^2(ctrl+sel)-chi^2(sel)-chi^2(ctrl): 11.2637
----

markers 4-5 (*px-sp*)

	Control	Desiccation
**Line 1**		
Teta	0.0698	0.0558
SE	0.0093	0.0084
**Line 2**		
Teta	0.0586	0.0639
SE	0.0086	0.0089
**Line 3**		
Teta	0.0534	0.0499
SE	0.0082	0.0079
Teta 3×750	0.0607	0.0588
SE	0.0050	0.0050
chi^2:	1.7188	0.7467

Teta Sel+Ctrl 6×750 0.0597
chi^2: 2.5338
chi^2(ctrl+sel)-chi^2(sel)-chi^2(ctrl): 0.0683

3rd Chromosome

markers 1-2 (*ru-h*)

	Control	Desiccation
**Line 1**		
Teta	0.1972	0.2025
SE	0.0145	0.0147
**Line 2**		
Teta	0.1940	0.2123
SE	0.0144	0.0149
**Line 3**		
Teta	0.1722	0.2052
SE	0.0138	0.0147
Teta 3×750	0.1880	0.2075
SE	0.0082	0.0085
chi^2:	1.7510	0.3585

Teta Sel+Ctrl 6×750 0.1973
chi^2: 4.8117
chi^2(ctrl+sel)-chi^2(sel)-chi^2(ctrl): 2.7022
----

markers 1-3 (*ru-th*)

	Control	Desiccation
**Line 1**		
Teta	0.2880	0.3252
SE	0.0165	0.0171
**Line 2**		
Teta	0.2868	0.3085
SE	0.0165	0.0168
**Line 3**		
Teta	0.2887	0.3332
SE	0.0165	0.0172
Teta 3×750	0.2889	0.3225
SE	0.0096	0.0099
chi^2:	0.0043	1.0321

Teta Sel+Ctrl 6×750 0.3052
chi^2: 7.0349
chi^2(ctrl+sel)-chi^2(sel)-chi^2(ctrl): 5.9984
----

markers 1-4 (*ru-cu*)

	Control	Desiccation
**Line 1**		
Teta	0.3436	0.3565
SE	0.0173	0.0175
**Line 2**		
Teta	0.3390	0.3370
SE	0.0173	0.0173
**Line 3**		
Teta	0.3437	0.3625
SE	0.0173	0.0175

Teta 3×750	0.3427	0.3523
SE	0.0100	0.0101
chi^2:	0.0356	1.1830

Sel+Ctrl 6×750 0.3474
chi^2: 1.6783
chi^2(ctrl+sel)-chi^2(sel)-chi^2(ctrl): 0.4598

<u>markers 1-5 (*ru-sr*)</u>

	**Control**	**Desiccation**
**Line 1**		
Teta	0.4066	0.3997
SE	0.0179	0.0178
**Line 2**		
Teta	0.4047	0.3766
SE	0.0179	0.0177
**Line 3**		
Teta	0.4060	0.4197
SE	0.0179	0.0180
Teta 3×750	0.4062	0.3993
SE	0.0104	0.0103
chi^2:	0.0037	2.8725

Teta Sel+Ctrl 6×750 0.4027
chi^2: 3.1007
chi^2(ctrl+sel)-chi^2(sel)-chi^2(ctrl): 0.2245

<u>markers 1-6 (*ru-e*)</u>

	**Control**	**Desiccation**
Line 1		
Teta	0.4587	0.4380
SE	0.0182	0.0181
Line 2		
Teta	0.4435	0.4217
SE	0.0181	0.0179
Line 3		
Teta	0.4543	0.4528
SE	0.0181	0.0181
Teta 3X750	0.4524	0.4386
SE	0.0105	0.0105
chi^2:	0.3485	1.3116

Teta Sel+Ctrl 6X750 0.4455
chi^2: 2.5321
chi^2(ctrl+sel)-chi^2(sel)-chi^2(ctrl): 0.8721
----

markers 2-3 (*h-th*)

	Control	Desiccation
**Line 1**		
Teta	0.1306	0.2258
SE	0.0123	0.0153
**Line 2**		
Teta	0.1423	0.1840
SE	0128	0.0141
**Line 3**		
Teta	0.1603	0.2182
SE	0.0134	0.0151
Teta 3×750	0.1440	0.2111
SE	0.0074	0.0086
chi^2:	2.8501	3.3174

Teta Sel+Ctrl 6×750 0.1726
chi^2: 41.2171
chi^2(ctrl+sel)-chi^2(sel)-chi^2(ctrl): 35.0496
----

markers 2-4 (*h-cu*)

	Control	Desiccation
**Line 1**		
Teta	0.1853	0.2559
SE	0.0142	0.0159
**Line 2**		
Teta	0.1983	0.2336
SE	0.0146	0.0154
**Line 3**		
Teta	0.2130	0.2475
SE	0.0149	0.0157
Teta 3×750	0.1991	0.2470
SE	0.0084	0.0091
chi^2:	1.6699	0.7101

Teta Sel+Ctrl 6×750 0.2212
chi^2: 17.2955
chi^2(ctrl+sel)-chi^2(sel)-chi^2(ctrl): 14.9155
----

markers 2-5 (*h-sr*)

	Control	Desiccation
**Line 1**		
Teta	0.2567	0.2775
SE	0.0159	0.0163
**Line 2**		
Teta	0.2772	0.2715
SE	0.0163	0.0162
**Line 3**		
Teta	0.2704	0.2888

SE	0.0162	0.0165

Teta 3×750	0.2683	0.2799
SE	0.0093	0.0095
chi^2:	0.7986	0.5666

Teta Sel+Ctrl 6×750 0.2740
chi^2: 2.1279
chi^2(ctrl+sel)-chi^2(sel)-chi^2(ctrl): 0.7626
----

markers 2-6 (*h-e*)

	Control	Desiccation
**Line 1**		
Teta	0.3119	0.3118
SE	0.0169	0.0169
**Line 2**		
Teta	0.3051	0.3153
SE	0.0170	0.0171
**Line 3**		
Teta	0.3184	0.3265
SE	0.0170	0.0171

Teta 3×750	0.3119	0.3199
SE	0.0098	0.0098
chi^2:	0.3107	0.3788

Teta Sel+Ctrl 6×750 0.3159
chi^2: 1.0220
chi^2(ctrl+sel)-chi^2(sel)-chi^2(ctrl): 0.3325
----

markers 3-4 (*th-cu*)

	Control	Desiccation
**Line 1**		
Teta	0.0525	0.0474
SE	0.0081	0.0078
**Line 2**		
Teta	0.0554	0.0557
SE	0.0084	0.0084
**Line 3**		
Teta	0.0557	0.0426
SE	0.0084	0.0074

Teta 3×750	0.0564	0.0486
SE	0.0049	0.0045
chi^2:	0.0166	1.7106

Teta Sel+Ctrl 6×750 0.0523
chi^2: 3.0989
chi^2(ctrl+sel)-chi^2(sel)-chi^2(ctrl): 1.3718
----

markers 3-5 (*th-sr*)

	Control	Desiccation
**Line 1**		
Teta	0.1300	0.1204
SE	0.0123	0.0119
**Line 2**		
Teta	0.1426	0.1665
SE	0.0128	0.0136
**Line 3**		
Teta	0.1347	0.1278
SE	0.0125	0.0122
Teta 3×750	0.1368	0.1369
SE	0.0072	0.0072
chi^2:	0.3659	6.8707

Teta Sel+Ctrl 6×750 0.1369
chi^2: 7.2369
chi^2(ctrl+sel)-chi^2(sel)-chi^2(ctrl): 0.0003
----

markers 3-6 (*th-e*)

	Control	Desiccation
**Line 1**		
Teta	0.1861	0.1702
SE	0.0142	0.0137
**Line 2**		
Teta	0.1865	0.2182
SE	0.0142	0.0150
**Line 3**		
Teta	0.1809	0.1931
SE	0.0141	0.0144
Teta 3×750	0.1849	0.1944
SE	0.0082	0.0083
chi^2:	0.0951	5.7995

Teta Sel+Ctrl 6×750 0.1896
chi^2: 6.5665
chi^2(ctrl+sel)-chi^2(sel)-chi^2(ctrl): 0.6720
----

markers 4-5 (*cu-sr*)

	Control	Desiccation
**Line 1**		
Teta	0.0746	0.0756
SE	0.0096	0.0096
**Line 2**		
Teta	0.0864	0.1168
SE	0.0103	0.0117
**Line 3**		
Teta	0.0811	0.0844

SE	0.0100	0.0101
Teta 3×750	0.0820	0.0918
SE	0.0058	0.0061
chi^2:	0.6298	6.7558

Teta Sel+Ctrl 6×750 0.0867
chi^2: 8.7572
chi^2(ctrl+sel)-chi^2(sel)-chi^2(ctrl): 1.3716

----

markers 4-6 (*cu-e*)

	**Control**	**Desiccation**
**Line 1**		
Teta	0.1305	0.1259
SE	0.0123	0.0121
**Line 2**		
Teta	0.1303	0.1735
SE	0.0123	0.0138
**Line 3**		
Teta	0.1295	0.1500
SE	0.0122	0.0130
Teta 3×750	0.1311	0.1501
SE	0.0071	0.0075
chi^2:	0.0078	6.0332

Teta Sel+Ctrl 6×750 0.1401
chi^2: 9.4170
chi^2(ctrl+sel)-chi^2(sel)-chi^2(ctrl): 3.3760

----

markers 5-6 (*sr-e*)

	**Control**	**Desiccation**
**Line 1**		
Teta	0.0534	0.0501
SE	0.0082	0.0080
**Line 2**		
Teta	0.0438	0.0485
SE	0.0075	0.0078
**Line 3**		
Teta	0.0504	0.0671
SE	0.0080	0.0091
Teta 3×750	0.0494	0.0599
SE	0.0046	0.0050
chi^2:	0.9475	1.4171

Teta Sel+Ctrl 6×750 0.0542
chi^2: 4.7650
chi^2(ctrl+sel)-chi^2(sel)-chi^2(ctrl): 2.4004

Hypoxia-hyperoxia experiment

X chromosome

markers 1–2 (*y-cv*)

	Control	Hypoxia
**Line 1**		
Teta	0.1292	0.1758
SE	0.0122	0.0139
**Line 2**		
Teta	0.1334	0.1515
SE	0.0124	0.0131
**Line 3**		
Teta	0.1299	0.2001
SE	0.0123	0.0146
Teta 3×750	0.1315	0.1746
SE	0.0071	0.0080
chi^2:	0.1004	6.3154

Teta Sel+Ctrl 6×750 0.1506
chi^2: 22.6227
chi^2(ctrl+sel)-chi^2(sel)-chi^2(ctrl): 16.2068
----

markers 1–3 (*y-v*)

	Control	Hypoxia
**Line 1**		
Teta	0.2901	0.3080
SE	0.0166	0.0168
**Line 2**		
Teta	0.2805	0.3415
SE	0.0164	0.0173
**Line 3**		
Teta	0.2842	0.3586
SE	0.0164	0.0175
Teta 3×750	0.2857	0.3362
SE	0.0095	0.0100
chi^2:	0.1609	4.3189

Teta Sel+Ctrl 6×750 0.3099
chi^2: 17.9227
chi^2(ctrl+sel)-chi^2(sel)-chi^2(ctrl): 13.4428
----

markers 1–4 (*y-f*)

	Control	Hypoxia
Line 1		
Teta	0.3917	0.2703

SE	0.0178	0.0162
Line 2		
Teta	0.3580	0.3266
SE	0.0175	0.0169
Line 3		
Teta	0.4079	0.2095
SE	0.0179	0.0148
Teta 3X750	0.3858	0.2666
SE	0.0103	0.0093
chi^2:	4.0517	29.0523

Teta Sel+Ctrl 6X750 0.3201
chi^2: 107.5704
chi^2(ctrl+sel)-chi^2(sel)-chi^2(ctrl): 74.4664

---

markers 2–3 (*cv-v*)

	**Control**	**Hypoxia**
**Line 1**		
Teta	0.1958	0.2533
SE	0.0145	0.0159
**Line 2**		
Teta	0.1899	0.2434
SE	0.0143	0.0156
**Line 3**		
Teta	0.1803	0.2878
SE	0.0140	0.0165
Teta 3×750	0.1896	0.2629
SE	0.0083	0.0093
chi^2:	0.5680	3.4047

Teta Sel+Ctrl 6×750 0.2220
chi^2: 38.7422
chi^2(ctrl+sel)-chi^2(sel)-chi^2(ctrl): 34.7695

---

markers 2–4 (*cv-f*)

	**Control**	**Hypoxia**
**Line 1**		
Teta	0.3667	0.3959
SE	0.0176	0.0179
**Line 2**		
Teta	0.3380	0.3851
SE	0.0173	0.0178
**Line 3**		
Teta	0.3518	0.3650
SE	0.0174	0.0176
Teta 3×750	0.3523	0.3821
SE	0.0101	0.0102
chi^2:	1.2893	1.5483

Teta Sel+Ctrl 6×750 0.3669
chi^2: 7.1386
chi^2(ctrl+sel)-chi^2(sel)-chi^2(ctrl): 4.3011
----

markers 3–4 (*v-f*)

	Control	Hypoxia
**Line 1**		
Teta	0.2265	0.3068
SE	0.0153	0.0168
**Line 2**		
Teta	0.2287	0.2873
SE	0.0153	0.0165
**Line 3**		
Teta	0.2205	0.2746
SE	0.0151	0.0163
Teta 3×750	0.2257	0.2898
SE	0.0088	0.0096
chi^2:	0.1430	2.0396

Teta Sel+Ctrl 6×750 0.2552
chi^2: 26.4856
chi^2(ctrl+sel)-chi^2(sel)-chi^2(ctrl): 24.3030
----

markers 1–2 (*y-cv*)

	Control	Hyperoxia
**Line 1**		
Teta	0.1292	0.1769
SE	0.0122	0.0139
**Line 2**		
Teta	0.1334	0.1455
SE	0.0124	0.0128
**Line 3**		
Teta	0.1299	0.1523
SE	0.0123	0.0131
Teta 3×750	0.1315	0.1597
SE	0.0071	0.0077
chi^2:	0.1004	2.3783

Teta Sel+Ctrl 6×750 0.1445
chi^2: 9.6908
chi^2(ctrl+sel)-chi^2(sel)-chi^2(ctrl): 7.2122
----

markers 1–3 (*y-v*)

	Control	Hyperoxia

**Line 1**		
Teta	0.2901	0.3790
SE	0.0166	0.0177
**Line 2**		
Teta	0.2805	0.3451
SE	0.0164	0.0173
**Line 3**		
Teta	0.2842	0.3412
SE	0.0164	0.0173

Teta 3×750	0.2857	0.3557
SE	0.0095	0.0101
chi^2:	0.1609	2.8385

Teta Sel+Ctrl 6×750 0.3187
chi^2: 28.3938
chi^2(ctrl+sel)-chi^2(sel)-chi^2(ctrl): 25.3944
----

markers 1–4 (*y-f*)

	**Control**	**Hyperoxia**
Line 1		
Teta	0.3917	0.2312
SE	0.0178	0.0154
Line 2		
Teta	0.3580	0.3266
SE	0.0175	0.0169
Line 3		
Teta	0.4079	0.2697
SE	0.0179	0.0161

Teta 3X750	0.3858	0.2777
SE	0.0103	0.0094
chi^2:	4.0517	19.1942

Teta Sel+Ctrl 6X750 0.3270
chi^2: 83.6810
chi^2(ctrl+sel)-chi^2(sel)-chi^2(ctrl): 60.4350
----

markers 2–3 (*cv-v*)

	**Control**	**Hyperoxia**
**Line 1**		
Teta	0.1958	0.2900
SE	0.0145	0.0166
**Line 2**		
Teta	0.1899	0.2543
SE	0.0143	0.0158
**Line 3**		
Teta	0.1803	0.2670
SE	0.0140	0.0161

Teta 3×750	0.1896	0.2725

SE	0.0083	0.0094
chi^2:	0.5680	1.9872

Teta Sel+Ctrl 6×750 0.2258
chi^2: 46.4926
chi^2(ctrl+sel)-chi^2(sel)-chi^2(ctrl): 43.9374

----

markers 2–4 (*cv-f*)

	**Control**	**Hyperoxia**
**Line 1**		
Teta	0.3667	0.3674
SE	0.0176	0.0176
**Line 2**		
Teta	0.3380	0.3789
SE	0.0173	0.0177
**Line 3**		
Teta	0.3518	0.3559
SE	0.0174	0.0175
Teta 3×750	0.3523	0.3679
SE	0.0101	0.0102
chi^2:	1.2893	0.9294

Teta Sel+Ctrl 6×750 0.3600
chi^2: 3.4101
chi^2(ctrl+sel)-chi^2(sel)-chi^2(ctrl): 1.1914

----

markers 3–4 (*v-f*)

	**Control**	**Hyperoxia**
**Line 1**		
Teta	0.2265	0.2827
SE	0.0153	0.0164
**Line 2**		
Teta	0.2287	0.2480
SE	0.0153	0.0156
**Line 3**		
Teta	0.2205	0.2855
SE	0.0151	0.0165
Teta 3×750	0.2257	0.2752
SE	0.0088	0.0094
chi^2:	0.1430	1.9412

Teta Sel+Ctrl 6×750 0.2488
chi^2: 16.7743
chi^2(ctrl+sel)-chi^2(sel)-chi^2(ctrl): 14.6902

2L Chromosome

markers 1-2 (*net-dp*)

	Control	Hypoxia
**Line 1**		
Teta	0.1253	0.1637
SE	0.0121	0.0135
**Line 2**		
Teta	0.1043	0.1595
SE	0.0111	0.0134
**Line 3**		
Teta	0.1368	0.1821
SE	0.0069	0.0079
Teta 3×750	0.1220	0.1701
SE	0.0069	0.0079
chi^2:	3.4149	1.7856

Teta Sel+Ctrl 6×750 0.1427
chi^2: 26.2658
chi^2(ctrl+sel)-chi^2(sel)-chi^2(ctrl): 21.0653

----

markers 1-3 (*net-b*)

	Control	Hypoxia
**Line 1**		
Teta	0.4000	0.3600
SE	0.0179	0.0175
**Line 2**		
Teta	0.4040	0.3813
SE	0.0179	0.0177
**Line 3**		
Teta	0.4037	0.3928
SE	0.0103	0.0102
Teta 3×750	0.4031	0.3780
SE	0.0103	0.0102
chi^2:	0.0481	1.8290

Teta Sel+Ctrl 6×750 0.3904
chi^2: 4.8547
chi^2(ctrl+sel)-chi^2(sel)-chi^2(ctrl): 2.9777

----

markers 1-4 (*net-pk*)

	Control	Hypoxia
**Line 1**		
Teta	0.4506	0.3455
SE	0.0182	0.0173
**Line 2**		
Teta	0.4394	0.3530
SE	0.0181	0.0174
**Line 3**		
Teta	0.4299	0.3689

SE	0.0180	0.0176
Teta 3×750	0.4404	0.3568
SE	0.0105	0.0101
chi^2:	0.6096	0.8607

Teta Sel+Ctrl 6×750 0.3971
chi^2: 34.5466
chi^2(ctrl+sel)-chi^2(sel)-chi^2(ctrl): 33.0762
----

markers 1-5 (*net-cn*)

	**Control**	**Hypoxia**
Line 1		
Teta	0.4666	0.3465
SE	0.0182	0.0173
Line 2		
Teta	0.4637	0.3466
SE	0.0182	0.0173
Line 3		
Teta	0.4379	0.3781
SE	0.0181	0.0177
Teta 3X750	0.4564	0.3584
SE	0.0105	0.0101
chi^2:	1.4496	1.9381

Teta Sel+Ctrl 6X750 0.4055
chi^2: 48.5782
chi^2(ctrl+sel)-chi^2(sel)-chi^2(ctrl): 45.1905
----

markers 2-3 (*dp-b*)

	**Control**	**Hypoxia**
**Line 1**		
Teta	0.2959	0.3683
SE	0.0167	0.0176
**Line 2**		
Teta	0.3077	0.3701
SE	0.0168	0.0176
**Line 3**		
Teta	0.3074	0.3752
SE	0.0168	0.0177
Teta 3×750	0.3048	0.3720
SE	0.0097	0.0102
chi^2:	0.4230	0.0798

Teta Sel+Ctrl 6×750 0.3368
chi^2: 23.2956
chi^2(ctrl+sel)-chi^2(sel)-chi^2(ctrl): 22.7928

----

markers 2-4 (*dp-pk*)

	Control	Hypoxia
**Line 1**		
Teta	0.3569	0.3839
SE	0.0175	0.0177
**Line 2**		
Teta	0.3515	0.3757
SE	0.0174	0.0177
**Line 3**		
Teta	0.3413	0.3811
SE	0.0173	0.0177
Teta 3×750	0.3502	0.3809
SE	0.0101	0.0102
chi^2:	0.4391	0.1396

Teta Sel+Ctrl 6×750 0.3652
chi^2: 5.1591
chi^2(ctrl+sel)-chi^2(sel)-chi^2(ctrl): 4.5804
----

markers 2-5 (*dp-cn*)

	Control	Hypoxia
**Line 1**		
Teta	0.3756	0.3881
SE	0.0177	0.0177
**Line 2**		
Teta	0.3731	0.3757
SE	0.0177	0.0177
**Line 3**		
Teta	0.3546	0.3983
SE	0.0102	0.0103
Teta 3×750	0.3679	0.3879
SE	0.0102	0.0103
chi^2:	0.8782	0.8218

Teta Sel+Ctrl 6×750 0.3778
chi^2: 3.6201
chi^2(ctrl+sel)-chi^2(sel)-chi^2(ctrl): 1.9201
----

markers 3-4 (*b-pk*)

	Control	Hypoxia
**Line 1**		
Teta	0.0741	0.0826
SE	0.0096	0.0101
**Line 2**		

Teta	0.0506	0.0765
SE	0.0080	0.0097
**Line 3**		
Teta	0.0675	0.0799
SE	0.0091	0.0099
Teta 3×750	0.0665	0.0799
SE	0.0053	0.0057
chi^2:	2.6276	0.1450

Teta Sel+Ctrl 6×750 0.0727
chi^2: 5.7553
chi^2(ctrl+sel)-chi^2(sel)-chi^2(ctrl): 2.9827
----

markers 3-5 (*b-cn*)

	**Control**	**Hypoxia**
**Line 1**		
Teta	0.0985	0.1023
SE	0.0109	0.0111
**Line 2**		
Teta	0.0734	0.0957
SE	0.0095	0.0107
**Line 3**		
Teta	0.0867	0.1026
SE	0.0103	0.0111
Teta 3×750	0.0883	0.1004
SE	0.0060	0.0063
chi^2:	1.6092	0.2509

Teta Sel+Ctrl 6×750 0.0940
chi^2: 3.7749
chi^2(ctrl+sel)-chi^2(sel)-chi^2(ctrl): 1.9148
----

markers 4-5 (*pk-cn*)

	**Control**	**Hypoxia**
**Line 1**		
Teta	0.0236	0.0209
SE	0.0055	0.0052
**Line 2**		
Teta	0.0222	0.0172
SE	0.0054	0.0047
**Line 3**		
Teta	0.0093	0.0221
SE	0.0035	0.0054
Teta 3×750	0.0219	0.0212
SE	0.0031	0.0030
chi^2:	0.7104	0.4082

Teta Sel+Ctrl 6×750 0.0215

chi^2: 1.1495
chi^2(ctrl+sel)-chi^2(sel)-chi^2(ctrl): 0.0309
----

markers 1-2 (*net-dp*)

	Control	Hyperoxia
**Line 1**		
Teta	0.1253	0.1509
SE	0.0121	0.0130
**Line 2**		
Teta	0.1043	0.1415
SE	0.0111	0.0127
**Line 3**		
Teta	0.1368	0.1370
SE	0.0125	0.0126
Teta 3×750	0.1220	0.1450
SE	0.0069	0.0074
chi^2:	3.4149	0.9142

Teta Sel+Ctrl 6×750 0.1327
chi^2: 9.5222
chi^2(ctrl+sel)-chi^2(sel)-chi^2(ctrl): 5.1932
----

markers 1-3 (*net-b*)

	Control	Hyperoxia
**Line 1**		
Teta	0.4000	0.3891
SE	0.0179	0.0178
**Line 2**		
Teta	0.4040	0.4227
SE	0.0179	0.0180
**Line 3**		
Teta	0.4037	0.3893
SE	0.0179	0.0178
Teta 3×750	0.4031	0.4003
SE	0.0103	0.0103
chi^2:	0.0481	2.2963

Teta Sel+Ctrl 6×750 0.4017
chi^2: 2.3826
chi^2(ctrl+sel)-chi^2(sel)-chi^2(ctrl): 0.0382
----

markers 1-4 (*net-pk*)

	Control	Hyperoxia
**Line 1**		
Teta	0.4506	0.3797

SE	0.0182	0.0177
**Line 2**		
Teta	0.4394	0.4091
SE	0.0181	0.0179
**Line 3**		
Teta	0.4299	0.3747
SE	0.0180	0.0177

Teta 3×750	0.4404	0.3878
SE	0.0105	0.0103
chi^2:	0.6096	2.1842

Teta Sel+Ctrl 6×750 0.4136
chi^2: 15.6774
chi^2(ctrl+sel)-chi^2(sel)-chi^2(ctrl): 12.8836
----

markers 1-5 (*net-cn*)

	Control	Hyperoxia
Line 1		
Teta	0.4666	0.3891
SE	0.0182	0.0178
Line 2		
Teta	0.4637	0.4076
SE	0.0182	0.0179
Line 3		
Teta	0.4379	0.3933
SE	0.0181	0.0178

Teta 3X750	0.4564	0.3968
SE	0.0105	0.0103
chi^2:	1.4496	0.6031

Teta Sel+Ctrl 6X750 0.4261
chi^2: 18.4278
chi^2(ctrl+sel)-chi^2(sel)-chi^2(ctrl): 16.3750
----

markers 2-3 (*dp-b*)

	Control	Hyperoxia
**Line 1**		
Teta	0.2959	0.3470
SE	0.0167	0.0173
**Line 2**		
Teta	0.3077	0.3735
SE	0.0168	0.0176
**Line 3**		
Teta	0.3074	0.3613
SE	0.0168	0.0175

Teta 3×750	0.3048	0.3616

SE	0.0097	0.0101
chi^2:	0.4230	1.0433

Teta Sel+Ctrl 6×750 0.3320
chi^2: 17.8869
chi^2(ctrl+sel)-chi^2(sel)-chi^2(ctrl): 16.4207

---

markers 2-4 (*dp-pk*)

	**Control**	**Hyperoxia**
**Line 1**		
Teta	0.3569	0.3617
SE	0.0175	0.0175
**Line 2**		
Teta	0.3515	0.3739
SE	0.0174	0.0176
**Line 3**		
Teta	0.3413	0.3813
SE	0.0173	0.0177
Teta 3×750	0.3502	0.3733
SE	0.0101	0.0102
chi^2:	0.4391	0.4913

Teta Sel+Ctrl 6×750 0.3616
chi^2: 3.5358
chi^2(ctrl+sel)-chi^2(sel)-chi^2(ctrl): 2.6054

---

markers 2-5 (*dp-cn*)

	**Control**	**Hyperoxia**
**Line 1**		
Teta	0.3756	0.3800
SE	0.0177	0.0177
**Line 2**		
Teta	0.3731	0.3751
SE	0.0177	0.0176
**Line 3**		
Teta	0.3546	0.4052
SE	0.0175	0.0179
Teta 3×750	0.3679	0.3874
SE	0.0102	0.0103
chi^2:	0.8782	1.5331

Teta Sel+Ctrl 6×750 0.3776
chi^2: 4.2356
chi^2(ctrl+sel)-chi^2(sel)-chi^2(ctrl): 1.8243

---

markers 3-4 (*b-pk*)

	**Control**	**Hyperoxia**

**Line 1**		
Teta	0.0741	0.0647
SE	0.0096	0.0090
**Line 2**		
Teta	0.0506	0.0438
SE	0.0080	0.0074
**Line 3**		
Teta	0.0675	0.0739
SE	0.0091	0.0095
Teta 3×750	0.0665	0.0665
SE	0.0053	0.0052
chi^2:	2.6276	2.7263

Teta Sel+Ctrl 6×750 0.0665
chi^2: 5.3540
chi^2(ctrl+sel)-chi^2(sel)-chi^2(ctrl): 0.0000

<u>markers 3-5 (*b-cn*)</u>

	**Control**	**Hyperoxia**
**Line 1**		
Teta	0.0985	0.0847
SE	0.0109	0.0101
**Line 2**		
Teta	0.0734	0.0612
SE	0.0095	0.0087
**Line 3**		
Teta	0.0867	0.0969
SE	0.0059	0.0056
Teta 3×750	0.0883	0.0842
SE	0.0060	0.0058
chi^2:	1.6092	5.5664

Teta Sel+Ctrl 6×750 0.0862
chi^2: 7.4211
chi^2(ctrl+sel)-chi^2(sel)-chi^2(ctrl): 0.2455

<u>markers 4-5 (*pk-cn*)</u>

	**Control**	**Hyperoxia**
**Line 1**		
Teta	0.0236	0.0193
SE	0.0055	0.0050
**Line 2**		
Teta	0.0222	0.0144
SE	0.0054	0.0044
**Line 3**		
Teta	0.0093	0.0197
SE	0.0035	0.0051
Teta 3×750	0.0219	0.0195

	Control	Hypoxia
SE	0.0031	0.0029
chi^2:	0.7104	2.2003

Teta Sel+Ctrl 6×750 0.0206
chi^2: 3.2347
chi^2(ctrl+sel)-chi^2(sel)-chi^2(ctrl): 0.3240

2R Chromosome

markers 1-2 (*cn-kn*)

	Control	Hypoxia
**Line 1**		
Teta	0.1027	0.0934
SE	0.0111	0.0106
**Line 2**		
Teta	0.0901	0.1039
SE	0.0104	0.0111
**Line 3**		
Teta	0.0984	0.1046
SE	0.0109	0.0111
Teta 3×750	0.0990	0.1038
SE	0.0063	0.0064
chi^2:	0.3665	0.4583

Teta Sel+Ctrl 6×750 0.1014
chi^2: 1.1162
chi^2(ctrl+sel)-chi^2(sel)-chi^2(ctrl): 0.2914

---

markers 1-3 (*cn-c*)

	Control	Hypoxia
**Line 1**		
Teta	0.1255	0.1104
SE	0.0121	0.0114
**Line 2**		
Teta	0.1131	0.1199
SE	0.0115	0.0119
**Line 3**		
Teta	0.1143	0.1206
SE	0.0116	0.0119
Teta 3×750	0.1189	0.1203
SE	0.0068	0.0069
chi^2:	0.6029	0.3092

Teta Sel+Ctrl 6×750 0.1196
chi^2: 0.9340
chi^2(ctrl+sel)-chi^2(sel)-chi^2(ctrl): 0.0219

---

markers 1-4 (*cn-px*)

	Control	Hypoxia
**Line 1**		
Teta	0.3593	0.3087
SE	0.0175	0.0168
**Line 2**		
Teta	0.3574	0.3180
SE	0.0174	0.0170
**Line 3**		
Teta	0.3120	0.3449
SE	0.0169	0.0173

Teta 3×750	0.3442	0.3246
SE	0.0100	0.0099
chi^2:	4.6587	2.2276

Teta Sel+Ctrl 6×750 0.3342
chi^2: 8.8448
chi^2(ctrl+sel)-chi^2(sel)-chi^2(ctrl): 1.9585

<u>markers 1-5 (*cn-sp*)</u>

	Control	Hypoxia
**Line 1**		
Teta	0.4170	0.3590
SE	0.0180	0.0174
**Line 2**		
Teta	0.4226	0.3652
SE	0.0180	0.0176
**Line 3**		
Teta	0.3619	0.4048
SE	0.0175	0.0179

Teta 3X750	0.4006	0.3769
SE	0.0103	0.0102
chi^2:	7.2142	3.7358

Teta Sel+Ctrl 6X750 0.3887
chi^2: 13.6155
chi^2(ctrl+sel)-chi^2(sel)-chi^2(ctrl): 2.6655

<u>markers 2-3 (*kn-c*)</u>

	Control	Hypoxia
**Line 1**		
Teta	0.0252	0.0139
SE	0.0057	0.0043
**Line 2**		
Teta	0.0192	0.0185
SE	0.0050	0.0049
**Line 3**		
Teta	0.0153	0.0153

	Control	Hypoxia
SE	0.0045	0.0045
Teta 3×750	0.0202	0.0173
SE	0.0030	0.0027
chi^2:	1.6868	0.1569

Teta Sel+Ctrl 6×750 0.0186
chi^2: 2.3604
chi^2(ctrl+sel)-chi^2(sel)-chi^2(ctrl): 0.5167

---

markers 2-4 (*kn-px*)

	**Control**	**Hypoxia**
**Line 1**		
Teta	0.2770	0.2356
SE	0.0163	0.0155
**Line 2**		
Teta	0.2865	0.2306
SE	0.0165	0.0154
**Line 3**		
Teta	0.2280	0.2465
SE	0.0153	0.0157
Teta 3×750	0.2647	0.2377
SE	0.0093	0.0090
chi^2:	7.3115	0.5456

Teta Sel+Ctrl 6×750 0.2507
chi^2: 12.2311
chi^2(ctrl+sel)-chi^2(sel)-chi^2(ctrl): 4.3740

---

markers 2-5 (*kn-sp*)

	**Control**	**Hypoxia**
**Line 1**		
Teta	0.3343	0.2973
SE	0.0172	0.0167
**Line 2**		
Teta	0.3542	0.2872
SE	0.0174	0.0165
**Line 3**		
Teta	0.3007	0.3172
SE	0.0167	0.0170
Teta 3×750	0.3299	0.3006
SE	0.0099	0.0097
chi^2:	5.2121	1.5884

Teta Sel+Ctrl 6×750 0.3149
chi^2: 11.2726
chi^2(ctrl+sel)-chi^2(sel)-chi^2(ctrl): 4.4721

---

markers 3-4 (*c-px)*

	Control	Hypoxia
**Line 1**		
Teta	0.2544	0.2202
SE	0.0159	0.0151
**Line 2**		
Teta	0.2687	0.2146
SE	0.0162	0.0150
**Line 3**		
Teta	0.2158	0.2306
SE	0.0150	0.0154
Teta 3×750	0.2467	0.2221
SE	0.0091	0.0088
chi^2:	5.8828	0.5585

Teta Sel+Ctrl 6×750 0.2339
chi^2: 10.2356
chi^2(ctrl+sel)-chi^2(sel)-chi^2(ctrl): 3.7942

---

markers 3-5 (*c-sp*)

	Control	Hypoxia
**Line 1**		
Teta	0.3118	0.2825
SE	0.0169	0.0164
**Line 2**		
Teta	0.3362	0.2713
SE	0.0172	0.0162
**Line 3**		
Teta	0.2904	0.3012
SE	0.0166	0.0167
Teta 3×750	0.3127	0.2850
SE	0.0098	0.0095
chi^2:	3.8035	1.6106

Teta Sel+Ctrl 6×750 0.2985
chi^2: 9.5353
chi^2(ctrl+sel)-chi^2(sel)-chi^2(ctrl): 4.1211

---

markers 4 -5 (*px-sp*)

	Control	Hypoxia
**Line 1**		
Teta	0.0701	0.0650
SE	0.0093	0.0090
**Line 2**		
Teta	0.0768	0.0532
SE	0.0097	0.0082
**Line 3**		
Teta	0.0779	0.0839

SE	0.0098	0.0101

Teta 3×750	0.0758	0.0701
SE	0.0056	0.0054
chi^2:	0.5054	2.7309

Teta Sel+Ctrl 6×750 0.0728
chi^2: 3.7834
chi^2(ctrl+sel)-chi^2(sel)-chi^2(ctrl): 0.5471

----

markers 1-2 (*cn-kn*)

	Control	Hyperoxia
**Line 1**		
Teta	0.1027	0.1006
SE	0.0111	0.0109
**Line 2**		
Teta	0.0901	0.0980
SE	0.0104	0.0108
**Line 3**		
Teta	0.0984	0.0923
SE	0.0109	0.0106
Teta 3×750	0.0990	0.1001
SE	0.0063	0.0063
chi^2:	0.3665	0.8918

Teta Sel+Ctrl 6×750 0.0996
chi^2: 1.2747
chi^2(ctrl+sel)-chi^2(sel)-chi^2(ctrl): 0.0165

----

markers 1-3 (*cn-c*)

	Control	Hyperoxia
**Line 1**		
Teta	0.1255	0.1326
SE	0.0121	0.0124
**Line 2**		
Teta	0.1131	0.1176
SE	0.0115	0.0117
**Line 3**		
Teta	0.1143	0.1184
SE	0.0116	0.0118
Teta 3×750	0.1189	0.1246
SE	0.0068	0.0070
chi^2:	0.6029	0.9528

Teta Sel+Ctrl 6×750 0.1217
chi^2: 1.8935
chi^2(ctrl+sel)-chi^2(sel)-chi^2(ctrl): 0.3379

----

markers 1-4 (*cn-px*)

	Control	Hyperoxia
**Line 1**		
Teta	0.3593	0.3618
SE	0.0175	0.0175
**Line 2**		
Teta	0.3574	0.3064
SE	0.0174	0.0168
**Line 3**		
Teta	0.3120	0.3157
SE	0.0169	0.0169
Teta 3×750	0.3442	0.3290
SE	0.0100	0.0099
chi^2:	4.6587	5.5068

Teta Sel+Ctrl 6×750 0.3365
chi^2: 11.3376
chi^2(ctrl+sel)-chi^2(sel)-chi^2(ctrl): 1.1721

---

markers 1-5 (*cn-sp*)

	Control	Hyperoxia
Line 1		
Teta	0.4170	0.3972
SE	0.0180	0.0178
Line 2		
Teta	0.4226	0.3595
SE	0.0180	0.0174
Line 3		
Teta	0.3619	0.3599
SE	0.0175	0.0175
Teta 3X750	0.4006	0.3735
SE	0.0103	0.0102
chi^2:	7.2142	2.9555

Teta Sel+Ctrl 6X750 0.3869
chi^2: 13.6832
chi^2(ctrl+sel)-chi^2(sel)-chi^2(ctrl): 3.5135

---

markers 2-3 (*kn-c*)

	Control	Hyperoxia
**Line 1**		
Teta	0.0252	0.0210
SE	0.0057	0.0052
**Line 2**		
Teta	0.0192	0.0195
SE	0.0050	0.0050
**Line 3**		
Teta	0.0153	0.0252

SE	0.0045	0.0057
Teta 3×750	0.0202	0.0240
SE	0.0030	0.0032
chi^2:	1.6868	1.0232

Teta Sel+Ctrl 6×750 0.0219
chi^2: 3.4789
chi^2(ctrl+sel)-chi^2(sel)-chi^2(ctrl): 0.7689

---

markers 2-4 (*kn-px*)

	Control	Hyperoxia
**Line 1**		
Teta	0.2770	0.2785
SE	0.0163	0.0164
**Line 2**		
Teta	0.2865	0.2351
SE	0.0165	0.0154
**Line 3**		
Teta	0.2280	0.2392
SE	0.0153	0.0155
Teta 3×750	0.2647	0.2535
SE	0.0093	0.0092
chi^2:	7.3115	3.4485

Teta Sel+Ctrl 6×750 0.2590
chi^2: 11.4928
chi^2(ctrl+sel)-chi^2(sel)-chi^2(ctrl): 0.7328

---

markers 2-5 (*kn-sp*)

	Control	Hyperoxia
**Line 1**		
Teta	0.3343	0.3384
SE	0.0172	0.0173
**Line 2**		
Teta	0.3542	0.3230
SE	0.0174	0.0170
**Line 3**		
Teta	0.3007	0.2929
SE	0.0167	0.0166
Teta 3×750	0.3299	0.3189
SE	0.0099	0.0098
chi^2:	5.2121	3.8791

Teta Sel+Ctrl 6×750 0.3243
chi^2: 9.7082
chi^2(ctrl+sel)-chi^2(sel)-chi^2(ctrl): 0.6170

---

markers 3-4 (*c-px)*

	Control	Hyperoxia
**Line 1**		
Teta	0.2544	0.2594
SE	0.0159	0.0160
**Line 2**		
Teta	0.2687	0.2184
SE	0.0162	0.0150
**Line 3**		
Teta	0.2158	0.2175
SE	0.0150	0.0150
Teta 3×750	0.2467	0.2342
SE	0.0091	0.0089
chi^2:	5.8828	3.7641

Teta Sel+Ctrl 6×750 0.2403
chi^2: 10.5980
chi^2(ctrl+sel)-chi^2(sel)-chi^2(ctrl): 0.9511

---

markers 3-5 (*c-sp*)

	Control	Hyperoxia
**Line 1**		
Teta	0.3118	0.3190
SE	0.0169	0.0170
**Line 2**		
Teta	0.3362	0.3060
SE	0.0172	0.0168
**Line 3**		
Teta	0.2904	0.2740
SE	0.0166	0.0163
Teta 3×750	0.3127	0.3006
SE	0.0098	0.0097
chi^2:	3.8035	4.0968

Teta Sel+Ctrl 6×750 0.3066
chi^2: 8.6784
chi^2(ctrl+sel)-chi^2(sel)-chi^2(ctrl): 0.7781

---

markers 4-5 (*px-sp*)

	Control	Hyperoxia
**Line 1**		
Teta	0.0701	0.0756
SE	0.0093	0.0097
**Line 2**		
Teta	0.0768	0.0879
SE	0.0097	0.0103
**Line 3**		
Teta	0.0779	0.0670
SE	0.0098	0.0091

Teta 3×750	0.0758	0.0771
SE	0.0056	0.0056
chi^2:	0.5054	1.8243

Teta Sel+Ctrl 6×750 0.0764
chi^2: 2.3561
chi^2(ctrl+sel)-chi^2(sel)-chi^2(ctrl): 0.0264

3rd Chromosome

markers 1–2 (*ru-h*)

	Control	Hypoxia
**Line 1**		
Teta	0.1319	0.1662
SE	0.0124	0.0136
**Line 2**		
Teta	0.1570	0.1354
SE	0.0133	0.0125
**Line 3**		
Teta	0.1583	0.1432
SE	0.0133	0.0128

Teta 3×750	0.1489	0.1480
SE	0.0075	0.0075
chi^2:	2.9414	2.8981

Teta Sel+Ctrl 6×750 0.1484
chi^2: 5.8460
chi^2(ctrl+sel)-chi^2(sel)-chi^2(ctrl): 0.0066
----

markers 1–3 (*ru-th*)

	Control	Hypoxia
**Line 1**		
Teta	0.2199	0.2718
SE	0.0151	0.0162
**Line 2**		
Teta	0.2591	0.2335
SE	0.0160	0.0154
**Line 3**		
Teta	0.2753	0.2186
SE	0.0163	0.0151

Teta 3×750	0.2505	0.2404
SE	0.0091	0.0090
chi^2:	6.8581	5.9928

Teta Sel+Ctrl 6×750 0.2454
chi^2: 13.4634

chi^2(ctrl+sel)-chi^2(sel)-chi^2(ctrl): 0.6124
----

markers 1–4 (*ru-cu*)

	Control	Hypoxia
**Line 1**		
Teta	0.2650	0.3372
SE	0.0161	0.0173
**Line 2**		
Teta	0.3203	0.2868
SE	0.0170	0.0165
**Line 3**		
Teta	0.3176	0.2704
SE	0.0170	0.0162
Teta 3×750	0.3004	0.2972
SE	0.0096	0.0096
chi^2:	7.3908	8.3884

Teta Sel+Ctrl 6×750 0.2988
chi^2: 15.8343
chi^2(ctrl+sel)-chi^2(sel)-chi^2(ctrl): 0.0551
----

markers 1–5 (*ru-sr*)

	Control	Hypoxia
**Line 1**		
Teta	0.3131	0.3987
SE	0.0169	0.0179
**Line 2**		
Teta	0.3821	0.3453
SE	0.0177	0.0173
**Line 3**		
Teta	0.3766	0.3163
SE	0.0177	0.0169
Teta 3×750	0.3564	0.3538
SE	0.0101	0.0101
chi^2:	10.0546	10.4039

Teta Sel+Ctrl 6×750 0.3551
chi^2: 20.4914
chi^2(ctrl+sel)-chi^2(sel)-chi^2(ctrl): 0.0330
----

markers 1–6 (*ru-e*)

	Control	Hypoxia
**Line 1**		
Teta	0.3475	0.4480
SE	0.0174	0.0182
**Line 2**		
Teta	0.4260	0.3962

SE	0.0180	0.0178
**Line 3**		
Teta	0.4207	0.3800
SE	0.0180	0.0176

Teta 3X750	0.3974	0.4093
SE	0.0103	0.0103
chi^2:	12.4547	7.0202

Teta Sel+Ctrl 6X750 0.4033
chi^2: 20.1352
chi^2(ctrl+sel)-chi^2(sel)-chi^2(ctrl): 0.6603
----

markers 2–3 (*h-th*)

	**Control**	**Hypoxia**
**Line 1**		
Teta	0.0980	0.1505
SE	0.0109	0.0131
**Line 2**		
Teta	0.1296	0.1231
SE	0.0123	0.0120
**Line 3**		
Teta	0.1296	0.1194
SE	0.0123	0.0118

Teta 3×750	0.1182	0.1309
SE	0.0068	0.0071
chi^2:	5.2701	3.3458

Teta Sel+Ctrl 6×750 0.1243
chi^2: 10.2897
chi^2(ctrl+sel)-chi^2(sel)-chi^2(ctrl): 1.6738
----

markers 2–4 (*h-cu*)

	**Control**	**Hypoxia**
**Line 1**		
Teta	0.1421	0.2157
SE	0.0127	0.0150
**Line 2**		
Teta	0.1955	0.1763
SE	0.0145	0.0139
**Line 3**		
Teta	0.1744	0.1699
SE	0.0138	0.0137

Teta 3×750	0.1701	0.1875
SE	0.0079	0.0082
chi^2:	7.8366	5.2613

Teta Sel+Ctrl 6×750 0.1785

chi^2: 15.4046
chi^2(ctrl+sel)-chi^2(sel)-chi^2(ctrl): 2.3067

<u>markers 2–5 (***h-sr***)</u>

	Control	Hypoxia
**Line 1**		
Teta	0.1903	0.2960
SE	0.0143	0.0167
**Line 2**		
Teta	0.2648	0.2494
SE	0.0161	0.0157
**Line 3**		
Teta	0.2335	0.2308
SE	0.0154	0.0153
Teta 3×750	0.2282	0.2608
SE	0.0088	0.0092
chi^2:	12.1848	6.9623

Teta Sel+Ctrl 6×750 0.2437
chi^2: 25.6663
chi^2(ctrl+sel)-chi^2(sel)-chi^2(ctrl): 6.5192

<u>markers 2–6 (***h-e***)</u>

	Control	Hypoxia
**Line 1**		
Teta	0.2302	0.3506
SE	0.0153	0.0174
**Line 2**		
Teta	0.3081	0.3110
SE	0.0168	0.0168
**Line 3**		
Teta	0.2802	0.2953
SE	0.0164	0.0165
Teta 3×750	0.2721	0.3215
SE	0.0094	0.0098
chi^2:	12.2939	4.4132

Teta Sel+Ctrl 6×750 0.2956
chi^2: 29.9459
chi^2(ctrl+sel)-chi^2(sel)-chi^2(ctrl): 13.2388

<u>markers 3–4 (***th-cu***)</u>

	Control	Hypoxia
**Line 1**		
Teta	0.0431	0.0839
SE	0.0074	0.0101
**Line 2**		
Teta	0.0659	0.0546

	Control	Hypoxia
SE	0.0090	0.0083
**Line 3**		
Teta	0.0474	0.0616
SE	0.0078	0.0088
Teta 3×750	0.0524	0.0658
SE	0.0047	0.0052
chi^2:	4.5159	4.7165

Teta Sel+Ctrl 6×750 0.0584
chi^2: 12.8256
chi^2(ctrl+sel)-chi^2(sel)-chi^2(ctrl): 3.5932
----

markers 3–5 (*th-sr*)

	**Control**	**Hypoxia**
**Line 1**		
Teta	0.0951	0.1769
SE	0.0107	0.0139
**Line 2**		
Teta	0.1341	0.1338
SE	0.0124	0.0124
**Line 3**		
Teta	0.1057	0.1376
SE	0.0066	0.0074
Teta 3×750	0.1114	0.1509
SE	0.0066	0.0075
chi^2:	6.8944	5.0975

Sel+Ctrl 6×750 0.1286
chi^2: 27.4551
chi^2(ctrl+sel)-chi^2(sel)-chi^2(ctrl): 15.4632
----

markers 3–6 (*th-e*)

	**Control**	**Hypoxia**
**Line 1**		
Teta	0.1351	0.2344
SE	0.0125	0.0155
**Line 2**		
Teta	0.1815	0.1949
SE	0.0140	0.0144
**Line 3**		
Teta	0.1525	0.2035
SE	0.0131	0.0146
Teta 3×750	0.1568	0.2144
SE	0.0077	0.0086
chi^2:	7.6134	2.7501

Teta Sel+Ctrl 6×750 0.1821
chi^2: 35.2383

chi^2(ctrl+sel)-chi^2(sel)-chi^2(ctrl): 24.8748
----

markers 4–5 (*cu-sr*)

	Control	Hypoxia
**Line 1**		
Teta	0.0522	0.0979
SE	0.0081	0.0108
**Line 2**		
Teta	0.0671	0.0779
SE	0.0091	0.0098
**Line 3**		
Teta	0.0572	0.0787
SE	0.0085	0.0098
Teta 3×750	0.0598	0.0879
SE	0.0050	0.0060
chi^2:	1.6950	1.3967

Teta Sel+Ctrl 6×750 0.0714
chi^2: 16.1732
chi^2(ctrl+sel)-chi^2(sel)-chi^2(ctrl): 13.0815
----

markers 4–6 (*cu-e*)

	Control	Hypoxia
**Line 1**		
Teta	0.0932	0.1581
SE	0.0106	0.0133
**Line 2**		
Teta	0.1132	0.1434
SE	0.0115	0.0128
**Line 3**		
Teta	0.1045	0.1438
SE	0.0112	0.0127
Teta 3×750	0.1049	0.1528
SE	0.0065	0.0076
chi^2:	2.5463	0.4204

Teta Sel+Ctrl 6×750 0.1250
chi^2: 26.0724
chi^2(ctrl+sel)-chi^2(sel)-chi^2(ctrl): 23.1056
----

markers 5–6 (*sr-e*)

	Control	Hypoxia
**Line 1**		
Teta	0.0384	0.0671
SE	0.0070	0.0091
**Line 2**		
Teta	0.0451	0.0676
SE	0.0076	0.0092

Line 3

	Control	Hyperoxia
Teta	0.0464	0.0707
SE	0.0077	0.0093
Teta 3×750	0.0450	0.0706
SE	0.0044	0.0054
chi^2:	0.8354	0.2781

Teta Sel+Ctrl 6×750 0.0551
chi^2: 14.6640
chi^2(ctrl+sel)-chi^2(sel)-chi^2(ctrl): 13.5505

----

markers 1–2 (*ru-h*)

	Control	Hyperoxia
**Line 1**		
Teta	0.1319	0.1504
SE	0.0124	0.0131
**Line 2**		
Teta	0.1570	0.1394
SE	0.0133	0.0126
**Line 3**		
Teta	0.1583	0.1381
SE	0.0133	0.0126
Teta 3×750	0.1489	0.1439
SE	0.0075	0.0074
chi^2:	2.9414	0.4038

Teta Sel+Ctrl 6×750 0.1463
chi^2: 3.5685
chi^2(ctrl+sel)-chi^2(sel)-chi^2(ctrl): 0.2233

----

markers 1–3 (*ru-th*)

	Control	Hyperoxia
**Line 1**		
Teta	0.2199	0.2492
SE	0.0151	0.0158
**Line 2**		
Teta	0.2591	0.2361
SE	0.0160	0.0155
**Line 3**		
Teta	0.2753	0.2553
SE	0.0163	0.0159
Teta 3×750	0.2505	0.2474
SE	0.0091	0.0091
chi^2:	6.8581	0.7261

Teta Sel+Ctrl 6×750 0.2489
chi^2: 7.6407
chi^2(ctrl+sel)-chi^2(sel)-chi^2(ctrl): 0.0565

----

markers 1–4 (*ru-cu*)

	Control	Hyperoxia
**Line 1**		
Teta	0.2650	0.2959
SE	0.0161	0.0167
**Line 2**		
Teta	0.3203	0.2792
SE	0.0170	0.0164
**Line 3**		
Teta	0.3176	0.3113
SE	0.0170	0.0169
Teta 3×750	0.3004	0.2957
SE	0.0096	0.0096
chi^2:	7.3908	1.8456

Teta Sel+Ctrl 6×750 0.2980
chi^2: 9.3581
chi^2(ctrl+sel)-chi^2(sel)-chi^2(ctrl): 0.1216
----

markers 1–5 (*ru-sr*)

	Control	Hyperoxia
**Line 1**		
Teta	0.3131	0.3573
SE	0.0169	0.0175
**Line 2**		
Teta	0.3821	0.3420
SE	0.0177	0.0173
**Line 3**		
Teta	0.3766	0.3758
SE	0.0177	0.0177
Teta 3×750	0.3564	0.3584
SE	0.0101	0.0101
chi^2:	10.0546	1.8181

Teta Sel+Ctrl 6×750 0.3574
chi^2: 11.8929
chi^2(ctrl+sel)-chi^2(sel)-chi^2(ctrl): 0.0203
----

markers 1–6 (*ru-e*)

	Control	Hyperoxia
Line 1		
Teta	0.3475	0.3971
SE	0.0174	0.0179
Line 2		
Teta	0.4260	0.3877
SE	0.0180	0.0178

Line 3		
Teta	0.4207	0.4198
SE	0.0180	0.0180

Teta 3X750	0.3974	0.4016
SE	0.0103	0.0103
chi^2:	12.4547	1.6836

Teta Sel+Ctrl 6X750 0.3995
chi^2: 14.2224
chi^2(ctrl+sel)-chi^2(sel)-chi^2(ctrl): 0.0841
----

markers 2–3 (*h-th*)

	**Control**	**Hyperoxia**
**Line 1**		
Teta	0.0980	0.1149
SE	0.0109	0.0116
**Line 2**		
Teta	0.1296	0.1089
SE	0.0123	0.0114
**Line 3**		
Teta	0.1296	0.1225
SE	0.0123	0.0120
Teta 3×750	0.1182	0.1166
SE	0.0068	0.0068
chi^2:	5.2701	0.7887

Teta Sel+Ctrl 6×750 0.1174
chi^2: 6.0857
chi^2(ctrl+sel)-chi^2(sel)-chi^2(ctrl): 0.0270
----

markers 2–4 (*h-cu*)

	**Control**	**Hyperoxia**
**Line 1**		
Teta	0.1421	0.1656
SE	0.0127	0.013
**Line 2**		
Teta	0.1955	0.1543
SE	0.0145	0.0132
**Line 3**		
Teta	0.1744	0.1801
SE	0.0138	0.0140
Teta 3×750	0.1701	0.1674
SE	0.0079	0.0079
chi^2:	7.8366	2.1038

Teta Sel+Ctrl 6×750 0.1688
chi^2: 10.0016

chi^2(ctrl+sel)-chi^2(sel)-chi^2(ctrl): 0.0612

----

markers 2–5 (*h-sr*)

	Control	Hyperoxia
**Line 1**		
Teta	0.1903	0.2272
SE	0.0143	0.0153
**Line 2**		
Teta	0.2648	0.2225
SE	0.0161	0.0152
**Line 3**		
Teta	0.2335	0.2478
SE	0.0154	0.0157
Teta 3×750	0.2282	0.2329
SE	0.0088	0.0089
chi^2:	12.1848	1.6382

Teta Sel+Ctrl 6×750 0.2305
chi^2: 13.9688
chi^2(ctrl+sel)-chi^2(sel)-chi^2(ctrl): 0.1458

----

markers 2–6 (*h-e*)

	Control	Hyperoxia
**Line 1**		
Teta	0.2302	0.2752
SE	0.0153	0.0163
**Line 2**		
Teta	0.3081	0.2677
SE	0.0168	0.0162
**Line 3**		
Teta	0.2802	0.2919
SE	0.0164	0.0166
Teta 3×750	0.2721	0.2789
SE	0.0094	0.0095
chi^2:	12.2939	1.2395

Teta Sel+Ctrl 6×750 0.2755
chi^2: 13.7891
chi^2(ctrl+sel)-chi^2(sel)-chi^2(ctrl): 0.2556

----

markers 3–4 (*th-cu*)

	Control	Hyperoxia
**Line 1**		
Teta	0.0431	0.0542
SE	0.0074	0.0083
**Line 2**		

	Control	Hyperoxia
Teta	0.0659	0.0478
SE	0.0090	0.0078
**Line 3**		
Teta	0.0474	0.0602
SE	0.0078	0.0087
Teta 3×750	0.0524	0.0548
SE	0.0047	0.0048
chi^2:	4.5159	1.8188

Teta Sel+Ctrl 6×750 0.0536
chi^2: 6.4615
chi^2(ctrl+sel)-chi^2(sel)-chi^2(ctrl): 0.1268

---

markers 3–5 (*th-sr*)

	**Control**	**Hyperoxia**
**Line 1**		
Teta	0.0951	0.1182
SE	0.0107	0.0118
**Line 2**		
Teta	0.1341	0.1159
SE	0.0124	0.0117
**Line 3**		
Teta	0.1057	0.1348
SE	0.0112	0.0125
Teta 3×750	0.1114	0.1230
SE	0.0066	0.0069
chi^2:	6.8944	1.5717

Teta Sel+Ctrl 6×750 0.1170
chi^2: 9.9308
chi^2(ctrl+sel)-chi^2(sel)-chi^2(ctrl): 1.4647

---

markers 3–6 (*th-e*)

	**Control**	**Hyperoxia**
**Line 1**		
Teta	0.1351	0.1635
SE	0.0125	0.0135
**Line 2**		
Teta	0.1815	0.1608
SE	0.0140	0.0134
**Line 3**		
Teta	0.1525	0.1830
SE	0.0131	0.0141
Teta 3×750	0.1568	0.1697
SE	0.0077	0.0079
chi^2:	7.6134	1.7798

Teta Sel+Ctrl 6×750	0.1631	
chi^2:	10.7619	

chi^2(ctrl+sel)-chi^2(sel)-chi^2(ctrl): 1.3687

----

markers 4–5 (*cu-sr*)

	Control	Hyperoxia
**Line 1**		
Teta	0.0522	0.0638
SE	0.0081	0.0089
**Line 2**		
Teta	0.0671	0.0679
SE	0.0091	0.0092
**Line 3**		
Teta	0.0572	0.0716
SE	0.0085	0.0094
Teta 3×750	0.0598	0.0679
SE	0.0050	0.0053
chi^2:	1.6950	0.3792

Teta Sel+Ctrl 6×750	0.0636
chi^2:	3.3015

chi^2(ctrl+sel)-chi^2(sel)-chi^2(ctrl): 1.2273

----

markers 4–6 (*cu-e)*

	Control	Hyperoxia
**Line 1**		
Teta	0.0932	0.1091
SE	0.0106	0.0114
**Line 2**		
Teta	0.1132	0.1126
SE	0.0115	0.0115
**Line 3**		
Teta	0.1045	0.1206
SE	0.0112	0.0119
Teta 3×750	0.1049	0.1145
SE	0.0065	0.0067
chi^2:	2.5463	0.5442

Teta Sel+Ctrl 6×750	0.1095
chi^2:	4.1521

chi^2(ctrl+sel)-chi^2(sel)-chi^2(ctrl): 1.0616

----

markers 5–6 (*sr-e*)

	Control	Hyperoxia

**Line 1**		
Teta	0.0384	0.0478
SE	0.0070	0.0078
**Line 2**		
Teta	0.0451	0.0443
SE	0.0076	0.0075
**Line 3**		
Teta	0.0464	0.0475
SE	0.0077	0.0078
Teta 3×750	0.0450	0.0475
SE	0.0044	0.0045
chi^2:	0.8354	0.1392

Teta Sel+Ctrl 6×750 0.0462
chi^2: 1.1346
chi^2(ctrl+sel)-chi^2(sel)-chi^2(ctrl): 0.1600
