# Supplementary material for: Experimental evolution of recombination and crossover interference in Drosophila caused by directional selection for stress-related traits
Source: BMC Biol. 2015 Nov 27;13:101. doi: 10.1186/s12915-015-0206-5 (PMC4661966; doi:10.1186/s12915-015-0206-5)
Supplement: Additional file 4: — Estimates of the coefficients of coincidence for adjacent intervals per replicate and entire variants (selection and control). (PDF 2958 kb) [file 12915_2015_206_MOESM4_ESM.pdf]

# Additional file 4

For each experiment (desiccation, hypoxia, hyperoxia), corresponding ML-estimates of the vectors of parameters [r1,r2,c] for each replicate line of control and selection variants are shown. For the entire Control variants and Selection variants, vectors of weighted estimates Teta [r1,r2,c] and corresponding vectors SE [r1,r2,c] are provided. In addition, chi-square for between-replicates heterogeneity of either Control or Selection are provided for the vectors [r1,r2,c] (statistics Chi2) and for coefficients of coincidence (statistics C Chi2) as described in Materials and Methods. In the same way, the total heterogeneity of all replicates taken over Control+Selection was calculated for the vector [r1,r2,c] (statistics Chi2) and for coefficients of coincidence (statistics C Chi2). Finally, chi-square for Control vs. Selection difference for the entire vector [r1,r2,c] is calculated as chi^2(ctrl+sel)-chi^2(sel)-chi^2(ctrl) and for the confident of coincidence as C chi^2(ctrl + sel) - chi^2(ctrl) - chi^2(sel) (see Materials and Methods).

We also employed the standard and more direct ML approach allowing for each line, in both selection and control, to have its own $r1_k$ and $r2_k$. Namely, to test for significance of the differences of *c* values in selection and control for earch pair of intervals, we performed log-likelihood ratio test to compare the model with 3×2+3×2=12 line-specific r-values plus $c_{control}$ plus $c_{selection}$ (in total 14 parameters) versus corresponding model with 12 line-specific r-values plus $c_{global}$ (13 parameters). Thus, $H_0$ and $H_1$ hypotheses are specified by 14 and 13 parameters. The (doubled) log-likelihood ratio test statistics for significance of the difference in c between control and selection is asymptotically distributed as $\chi^2$ with df=1 (present in the tables as: ML ratio (df=14 –13=1). To facilitate the comparison of the c values in the model with 14 parameters (where $c_{control}$ and $c_{selection}$ are line-independent) with those in the full model with 18 parameters (with line-specific c-values), we repeated the corresponding outputs at the end of each marker combination, and marked by asterisk.

**Note: non-nested design was used in all three selection experiments, so that pair-wise comparisons of control vs. selection replicates is impossible.**

## Desiccation experiment

### X chromosome

**markers 1-2-3 (*y-cv-v*)**

| | CONTROL | | | DESICCATION | | |
|---|---|---|---|---|---|---|
| **Lines (ML estimates)** | | | | | | |
| Line1 [r1,r2,c]: | 0.1010 | 0.1867 | 0.6066 | 0.1248 | 0.2733 | 1.0612 |
| Line2 [r1,r2,c]: | 0.1076 | 0.1981 | 0.6701 | 0.1143 | 0.2733 | 0.7927 |
| Line3 [r1,r2,c]: | 0.1000 | 0.2067 | 0.4148 | 0.0876 | 0.2476 | 0.9657 |
| | | | | | | |
| Bailey test for 3 × 750 | | | | | | |
| Teta [r1,r2,c]: | 0.1029 | 0.1968 | 0.5433 | 0.1069 | 0.2645 | 0.9320 |
| SE [r1,r2,c]: | 0.0054 | 0.0071 | 0.0842 | 0.0055 | 0.0079 | 0.0825 |
| Chi2: | | 3.5811 | | | 13.1682 | |
| C Chi2: | | 1.8230 | | | 2.0867 | |

Bailey for 6 × 750:

| | |
|---|---|
| Teta: | 0.1042 0.2273 0.7449 |
| Chi2: | 68.5291 |
| C chi2: | 14.7913 |

chi^2(ctrl + sel) - chi^2(ctrl) - chi^2(sel): 51.7797
C chi^2(ctrl + sel) - chi^2(ctrl) - chi^2(sel): **10.8816**

**ML test Ctrl vs. Selection (analysis of totals)**

| | | |
|---|---|---|
| ML est [r1,r2,c] | 0.1029 0.1971 0.5636 | 0.1089 0.2648 0.9470 |
| ML SE [r1,r2,c] | 0.0054 0.0071 0.0857 | 0.0056 0.0079 0.0836 |

**ML test Ctrl vs. Selection (r1 and r2 are line-specific)**

c - control or selection specific

| | | | | | | |
|---|---|---|---|---|---|---|
| Line1[r1,r2,c]: | 0.1009 | 0.1866 | 0.5635 | 0.1247 | 0.2732 | 0.9412 |
| Line2[r1,r2,c]: | 0.1075 | 0.1979 | 0.5635 | 0.1143 | 0.2734 | 0.9412 |
| Line3[r1,r2,c]: | 0.1002 | 0.2070 | 0.5635 | 0.0876 | 0.2476 | 0.9412 |

c-global

| | | | | | | |
|---|---|---|---|---|---|---|
| Line1[r1,r2,c]: | 0.1010 | 0.1868 | 0.7925 | 0.1244 | 0.2726 | 0.7925 |
| Line2[r1,r2,c]: | 0.1077 | 0.1982 | 0.7925 | 0.1143 | 0.2733 | 0.7925 |
| Line3[r1,r2,c]: | 0.1002 | 0.2071 | 0.7925 | 0.0875 | 0.2474 | 0.7925 |

ML ratio (df=14 –13=1): 9.3896

***ML test Ctrl vs. Selection (r1, r2 and c are all line-specific)**

| | | | | | | |
|---|---|---|---|---|---|---|
| Line1[r1,r2,c]: | 0.1010 | 0.1867 | 0.6066 | 0.1248 | 0.2733 | 1.0612 |
| Line2[r1,r2,c]: | 0.1076 | 0.1981 | 0.6701 | 0.1143 | 0.2733 | 0.7927 |
| Line3[r1,r2,c]: | 0.1000 | 0.2067 | 0.4148 | 0.0876 | 0.2476 | 0.9657 |

ML ratio (df=18 –13=5): 13.1399

ML ratio for heterogeneity (control+selection; df = 5–1=4): 13.1399 – 9.3896

……………………………………………………………………………………………………

## markers 1-2-4 (*y-cv-f*)

| | CONTROL | DESICCATION |
|---|---|---|
| **Lines (ML estimates)** | | |
| Line1 [r1,r2,c]: | 0.1009 0.3505 0.9421 | 0.1248 0.3648 2.0509 |
| Line2 [r1,r2,c]: | 0.1077 0.3527 0.9785 | 0.1143 0.3600 1.7130 |
| Line3 [r1,r2,c]: | 0.1000 0.3648 0.7833 | 0.0876 0.3219 1.7895 |
| | | |
| Bailey test for 3 × 750 | | |
| Teta [r1,r2,c]: | 0.1029 0.3560 0.8970 | 0.1069 0.3491 1.9127 |
| SE [r1,r2,c]: | 0.0054 0.0085 0.0690 | 0.0055 0.0084 0.0708 |
| Chi2: | 2.5169 | 19.9406 |

| | | |
|---|---|---|
| C Chi2: | 1.5665 | 5.6349 |

Bailey for 6 × 750:

| | |
|---|---|
| Teta: | 0.1075 0.3622 1.4031 |
| Chi2: | 130.2446 |
| C chi2: | 112.6332 |

**ML test Control vs. selection**

chi^2(ctrl + sel) - chi^2(ctrl) - chi^2(sel): 107.7871
C chi^2(ctrl + sel) - chi^2(ctrl) - chi^2(sel): **105.4318**

**ML test Ctrl Vs. Selection**

| | | |
|---|---|---|
| ML est [r1,r2,c] | 0.1029 0.3560 0.9017 | 0.1089 0.3489 1.8802 |
| ML SE [r1,r2,c] | 0.0054 0.0085 0.0692 | 0.0056 0.0085 0.0727 |

**ML test Ctrl vs. Selection (r1 and r2 are line-specific)**

c - control or selection specific

| | | | | | | |
|---|---|---|---|---|---|---|
| Line1[r1,r2,c]: | 0.1009 | 0.3504 | 0.9014 | 0.1277 | 0.3734 | 1.8746 |
| Line2[r1,r2,c]: | 0.1077 | 0.3525 | 0.9014 | 0.1123 | 0.3538 | 1.8746 |
| Line3[r1,r2,c]: | 0.1001 | 0.3650 | 0.9014 | 0.0871 | 0.3201 | 1.8746 |

c-global

| | | | | | | |
|---|---|---|---|---|---|---|
| Line1[r1,r2,c]: | 0.0995 | 0.3455 | 1.4072 | 0.1285 | 0.3756 | 1.4072 |
| Line2[r1,r2,c]: | 0.1062 | 0.3477 | 1.4072 | 0.1157 | 0.3643 | 1.4072 |
| Line3[r1,r2,c]: | 0.0980 | 0.3574 | 1.4072 | 0.0884 | 0.3248 | 1.4072 |

ML ratio (df=14 –13=1): 90.2680

***ML test Ctrl vs. Selection (r1, r2 and c are all line-specific)**

| | | | | | | |
|---|---|---|---|---|---|---|
| Line1[r1,r2,c]: | 0.1009 | 0.3505 | 0.9421 | 0.1248 | 0.3648 | 2.0509 |
| Line2[r1,r2,c]: | 0.1077 | 0.3527 | 0.9785 | 0.1143 | 0.3600 | 1.7130 |
| Line3[r1,r2,c]: | 0.1000 | 0.3648 | 0.7833 | 0.0876 | 0.3219 | 1.7895 |

ML ratio (df=18 –13=5): 96.5072

ML ratio for heterogeneity (control+selection; df = 5–1=4): 96.5072 –90.2680
……………………………………………………………………………………………………

## markers 1-3-4 (*y-v-f*)

| | **CONTROL** | **DESICCATION** |
|---|---|---|
| **Lines (ML estimates)** | | |
| Line1 [r1,r2,c]: | 0.2648 0.2095 0.8069 | 0.3257 0.3429 1.6374 |
| Line2 [r1,r2,c]: | 0.2774 0.2231 0.9243 | 0.3381 0.3095 1.5016 |
| Line3 [r1,r2,c]: | 0.2895 0.2248 0.8196 | 0.2933 0.3124 1.6214 |

Bailey test for 3 × 750

| | | | | | | |
|---|---|---|---|---|---|---|
| Teta [r1,r2,c]: | 0.2770 | 0.2190 | 0.8497 | 0.3183 | 0.3219 | 1.5925 |
| SE [r1,r2,c]: | 0.0080 | 0.0074 | 0.0521 | 0.0083 | 0.0083 | 0.0405 |
| Chi2: | | 3.6007 | | | 11.9225 | |
| C Chi2: | | 0.9973 | | | 2.6439 | |

Bailey for 6 × 750:

| | |
|---|---|
| Teta: | 0.2969 0.2703 1.3912 |
| Chi2: | 269.3792 |
| C chi2: | 138.3540 |

**ML test Control vs. selection**

chi^2(ctrl + sel) - chi^2(ctrl) - chi^2(sel): 253.8560

C chi^2(ctrl + sel) - chi^2(ctrl) - chi^2(sel): **134.7128**

**ML test Ctrl Vs. Selection**

| | | | | | | |
|---|---|---|---|---|---|---|
| ML est [r1,r2,c] | 0.2772 | 0.2191 | 0.8521 | 0.3190 | 0.3216 | 1.5873 |
| ML SE [r1,r2,c] | 0.0080 | 0.0074 | 0.0523 | 0.0083 | 0.0083 | 0.0407 |

**ML test Ctrl vs. Selection (r1 and r2 are line-specific)**

c - control or selection specific

| | | | | | | |
|---|---|---|---|---|---|---|
| Line1[r1,r2,c]: | 0.2649 | 0.2096 | 0.8514 | 0.3291 | 0.3464 | 1.5872 |
| Line2[r1,r2,c]: | 0.2771 | 0.2228 | 0.8514 | 0.3329 | 0.3048 | 1.5872 |
| Line3[r1,r2,c]: | 0.2897 | 0.2249 | 0.8514 | 0.2949 | 0.3140 | 1.5872 |

c-global

| | | | | | | |
|---|---|---|---|---|---|---|
| Line1[r1,r2,c]: | 0.2589 | 0.2049 | 1.3612 | 0.3369 | 0.3546 | 1.3612 |
| Line2[r1,r2,c]: | 0.2718 | 0.2185 | 1.3612 | 0.3432 | 0.3142 | 1.3612 |
| Line3[r1,r2,c]: | 0.2818 | 0.2188 | 1.3612 | 0.3001 | 0.3196 | 1.3612 |

ML ratio (df=14 –13=1): 115.2041

***ML test Ctrl vs. Selection (r1, r2 and c are all line-specific)**

| | | | | | | |
|---|---|---|---|---|---|---|
| Line1[r1,r2,c]: | 0.2648 | 0.2095 | 0.8069 | 0.3257 | 0.3429 | 1.6374 |
| Line2[r1,r2,c]: | 0.2774 | 0.2231 | 0.9243 | 0.3381 | 0.3095 | 1.5016 |
| Line3[r1,r2,c]: | 0.2895 | 0.2248 | 0.8196 | 0.2933 | 0.3124 | 1.6214 |

ML ratio (df=18 –13=5): 118.5054

ML ratio for heterogeneity (control+selection; df = 5–1=4): 118.5054 – 115.2041

…………………………………………………………………………………………………

## markers 2-3-4 (*cv-v-f*)

| | CONTROL | | | DESICCATION | | |
|---|---|---|---|---|---|---|
| **Lines (ML estimates)** | | | | | | |
| Line1 [r1,r2,c]: | 0.1867 | 0.2095 | 0.5844 | 0.2733 | 0.3429 | 1.3415 |

| | | |
|---|---|---|
| Line2 [r1,r2,c]: | 0.1983 0.2231 0.7759 | 0.2733 0.3095 1.3171 |
| Line3 [r1,r2,c]: | 0.2067 0.2248 0.7176 | 0.2476 0.3124 1.5391 |

Bailey test for 3 × 750

| | | |
|---|---|---|
| Teta [r1,r2,c]: | 0.1969 0.2189 0.6920 | 0.2649 0.3223 1.3886 |
| SE [r1,r2,c]: | 0.0071 0.0074 0.0604 | 0.0078 0.0083 0.0451 |
| Chi2: | 3.9923 | 8.7126 |
| C Chi2: | 1.7289 | 4.5763 |

Bailey for 6 × 750:

Teta: 0.2250 0.2645 1.1943

Chi2: 233.5559

C chi2: 95.0932

**ML test Control vs. selection**

chi^2(ctrl + sel) - chi^2(ctrl) - chi^2(sel): 220.8510

C chi^2(ctrl + sel) - chi^2(ctrl) - chi^2(sel): **88.7880**

**ML test Ctrl Vs. Selection**

| | | |
|---|---|---|
| ML est [r1,r2,c] | 0.1972 0.2191 0.6982 | 0.2648 0.3216 1.3945 |
| ML SE [r1,r2,c] | 0.0071 0.0074 0.0608 | 0.0079 0.0083 0.0453 |

**ML test Ctrl vs. Selection (r1 and r2 are line-specific)**

c - control or selection specific

| | | | | | | |
|---|---|---|---|---|---|---|
| Line1[r1,r2,c]: | 0.1870 | 0.2099 | 0.6981 | 0.2720 | 0.3412 | 1.3913 |
| Line2[r1,r2,c]: | 0.1980 | 0.2227 | 0.6981 | 0.2717 | 0.3076 | 1.3913 |
| Line3[r1,r2,c]: | 0.2066 | 0.2247 | 0.6981 | 0.2504 | 0.3158 | 1.3913 |

c-global

| | | | | | | |
|---|---|---|---|---|---|---|
| Line1[r1,r2,c]: | 0.1852 | 0.2078 | 1.1989 | 0.2751 | 0.3451 | 1.1989 |
| Line2[r1,r2,c]: | 0.1970 | 0.2216 | 1.1989 | 0.2746 | 0.3109 | 1.1989 |
| Line3[r1,r2,c]: | 0.2050 | 0.2230 | 1.1989 | 0.2505 | 0.3160 | 1.1989 |

ML ratio (df=14 –13=1): 72.7467

***ML test Ctrl vs. Selection (r1, r2 and c are all line-specific)**

| | | | | | | |
|---|---|---|---|---|---|---|
| Line1[r1,r2,c]: | 0.1867 | 0.2095 | 0.5844 | 0.2733 | 0.3429 | 1.3415 |
| Line2[r1,r2,c]: | 0.1983 | 0.2231 | 0.7759 | 0.2733 | 0.3095 | 1.3171 |
| Line3[r1,r2,c]: | 0.2067 | 0.2248 | 0.7176 | 0.2476 | 0.3124 | 1.5391 |

ML ratio (df=18 –13=5): 78.8423

ML ratio for heterogeneity (control+selection; df = 5–1=4): 78.8423 – 72.7467

------------------------------------------------------------------------------------------------------------

## Desiccation/2L chromosome

**markers 1-2-3 (*net-dp-b*)**

| | CONTROL | | | DESICCATION | | |
|---|---|---|---|---|---|---|
| **Lines (ML estimates)** | | | | | | |
| Line1 [r1,r2,c]: | 0.1187 | 0.3040 | 0.4435 | 0.1640 | 0.2987 | 0.7078 |
| Line2 [r1,r2,c]: | 0.1053 | 0.2933 | 0.3021 | 0.1773 | 0.2880 | 0.9660 |
| Line3 [r1,r2,c]: | 0.0987 | 0.2840 | 0.2855 | 0.1667 | 0.2947 | 0.7602 |

Bailey test for 3 × 750

| | | | | | | |
|---|---|---|---|---|---|---|
| teta [r1,r2,c]: | 0.1069 | 0.2936 | 0.3393 | 0.1694 | 0.2938 | 0.8055 |
| SE [r1,r2,c]: | 0.0065 | 0.0096 | 0.0638 | 0.0079 | 0.0096 | 0.0683 |
| chi2: | | 3.6677 | | | 3.2918 | |
| C chi2: | | 1.1831 | | | 2.5847 | |

Bailey for 6 × 750:

| | | | |
|---|---|---|---|
| teta: | 0.1322 | 0.2924 | 0.5570 |
| chi2: | 67.9476 | | |
| C chi2: | 28.6520 | | |

**ML test Control vs. selection**

chi^2(ctrl + sel) - chi^2(ctrl) - chi^2(sel): 60.9881

**C** chi^2(ctrl + sel) - chi^2(ctrl) - chi^2(sel): **24.8841**

**ML test Control vs. selection**

| | | | | | | |
|---|---|---|---|---|---|---|
| ML est [r1,r2,c] | 0.1076 | 0.2938 | 0.3516 | 0.1693 | 0.2938 | 0.8130 |
| ML SE [r1,r2,c] | 0.0065 | 0.0096 | 0.0651 | 0.0079 | 0.0096 | 0.0687 |

**ML test Ctrl vs. Selection (r1 and r2 are line-specific)**

c - control or selection specific

| | | | | | | |
|---|---|---|---|---|---|---|
| Line1[r1,r2,c]: | 0.1184 | 0.3032 | 0.3514 | 0.1642 | 0.2991 | 0.8134 |
| Line2[r1,r2,c]: | 0.1055 | 0.2937 | 0.3514 | 0.1769 | 0.2874 | 0.8134 |
| Line3[r1,r2,c]: | 0.0988 | 0.2844 | 0.3514 | 0.1668 | 0.2949 | 0.8134 |

c-global

| | | | | | | |
|---|---|---|---|---|---|---|
| Line1[r1,r2,c]: | 0.1191 | 0.3051 | 0.6387 | 0.1637 | 0.2982 | 0.6387 |
| Line2[r1,r2,c]: | 0.1059 | 0.2948 | 0.6387 | 0.1758 | 0.2856 | 0.6387 |
| Line3[r1,r2,c]: | 0.0991 | 0.2853 | 0.6387 | 0.1662 | 0.2938 | 0.6387 |

ML ratio (df=14 –13=1): 21.1257

***ML test Ctrl vs. Selection (r1, r2 and c are all line-specific)**

| | | | | | | |
|---|---|---|---|---|---|---|
| Line1[r1,r2,c]: | 0.1187 | 0.3040 | 0.4435 | 0.1640 | 0.2987 | 0.7078 |
| Line2[r1,r2,c]: | 0.1053 | 0.2933 | 0.3021 | 0.1773 | 0.2880 | 0.9660 |
| Line3[r1,r2,c]: | 0.0987 | 0.2840 | 0.2855 | 0.1667 | 0.2947 | 0.7602 |

ML ratio (df=18 –13=5): 24.9968

ML ratio for heterogeneity (control+selection; df = 5–1=4): 24.9968 – 21.1257
-----------------------------------------------------------------------------------------------------------------

## markers 1-2-4 (*net-dp-pk*)

| | CONTROL | DESICCATION |
|---|---|---|
| **Lines (ML estimates)** | | |
| Line1 [r1,r2,c]: | 0.1187 0.3453 0.4230 | 0.1640 0.3413 0.6907 |
| Line2 [r1,r2,c]: | 0.1053 0.3533 0.3224 | 0.1773 0.3387 0.8659 |
| Line3 [r1,r2,c]: | 0.0987 0.3373 0.3205 | 0.1667 0.3453 0.6486 |
| Bailey test for 3 × 750 | | |
| Teta [r1,r2,c]: | 0.1070 0.3453 0.3535 | 0.1693 0.3418 0.7315 |
| SE [r1,r2,c]: | 0.0065 0.0100 0.0594 | 0.0079 0.0100 0.0600 |
| Chi2: | 2.7239 | 2.9832 |
| C Chi2: | 0.6389 | 2.4189 |

Bailey for 6 × 750:
Teta: 0.1322 0.3426 0.5394
Chi2: 61.3390
C chi2: 23.1082

**ML test Control vs. selection**
chi^2(ctrl + sel) - chi^2(ctrl) - chi^2(sel): 55.6320
C chi^2(ctrl + sel) - chi^2(ctrl) - chi^2(sel): **20.0504**

**ML test Ctrl Vs. Selection**

| | | |
|---|---|---|
| ML est [r1,r2,c] | 0.1076 0.3453 0.3590 | 0.1693 0.3418 0.7372 |
| ML SE [r1,r2,c] | 0.0065 0.0100 0.0598 | 0.0079 0.0100 0.0604 |

**ML test Ctrl vs. Selection (r1 and r2 are line-specific)**

| | | | | | | |
|---|---|---|---|---|---|---|
| c - control or selection specific | | | | | | |
| Line1[r1,r2,c]: | 0.1184 | 0.3446 | 0.3590 | 0.1642 | 0.3417 | 0.7375 |
| Line2[r1,r2,c]: | 0.1054 | 0.3537 | 0.3590 | 0.1768 | 0.3376 | 0.7375 |
| Line3[r1,r2,c]: | 0.0988 | 0.3377 | 0.3590 | 0.1670 | 0.3461 | 0.7375 |
| c-global | | | | | | |
| Line1[r1,r2,c]: | 0.1191 | 0.3467 | 0.5931 | 0.1635 | 0.3402 | 0.5931 |
| Line2[r1,r2,c]: | 0.1059 | 0.3553 | 0.5931 | 0.1757 | 0.3355 | 0.5931 |
| Line3[r1,r2,c]: | 0.0992 | 0.3390 | 0.5931 | 0.1664 | 0.3447 | 0.5931 |

ML ratio (df=14 –13=1): 17.9270

***ML test Ctrl vs. Selection (r1, r2 and c are all line-specific)**

| | | | | | | |
|---|---|---|---|---|---|---|
| Line1[r1,r2,c]: | 0.1187 | 0.3453 | 0.4230 | 0.1640 | 0.3413 | 0.6907 |

| | | | | | | |
|---|---|---|---|---|---|---|
| Line2[r1,r2,c]: | 0.1053 | 0.3533 | 0.3224 | 0.1773 | 0.3387 | 0.8659 |
| Line3[r1,r2,c]: | 0.0987 | 0.3373 | 0.3205 | 0.1667 | 0.3453 | 0.6486 |

ML ratio (df=18 –13=5): 21.0421

ML ratio for heterogeneity (control+selection; df = 5–1=4): 21.0421 – 17.9270
..........................................................................................................

**markers 1-2-5 (*net-dp-cn*)**

| | CONTROL | | | DESICCATION | | |
|---|---|---|---|---|---|---|
| **Lines (ML estimates)** | | | | | | |
| Line1 [r1,r2,c]: | 0.1187 | 0.3640 | 0.4013 | 0.1640 | 0.3627 | 0.6725 |
| Line2 [r1,r2,c]: | 0.1053 | 0.3760 | 0.3030 | 0.1773 | 0.3560 | 0.8237 |
| Line3 [r1,r2,c]: | 0.0987 | 0.3587 | 0.3391 | 0.1667 | 0.3627 | 0.6176 |
| | | | | | | |
| Bailey test for 3 × 750 | | | | | | |
| Teta [r1,r2,c]: | 0.1070 | 0.3662 | 0.3453 | 0.1693 | 0.3604 | 0.7011 |
| SE [r1,r2,c]: | 0.0065 | 0.0102 | 0.0569 | 0.0079 | 0.0101 | 0.0573 |
| Chi2: | | 2.7082 | | | 2.8652 | |
| C Chi2: | | 0.5285 | | | 2.2941 | |

Bailey for 6 × 750:

| | | | |
|---|---|---|---|
| Teta: | 0.1322 | 0.3625 | 0.5207 |
| Chi2: | 60.3986 | | |
| C chi2: | 22.2763 | | |

**ML test Control vs. selection**

chi^2(ctrl + sel) - chi^2(ctrl) - chi^2(sel): 54.8252
C chi^2(ctrl + sel) - chi^2(ctrl) - chi^2(sel): **19.4537**

**ML test Ctrl Vs. Selection**

| | | | | | | |
|---|---|---|---|---|---|---|
| ML est [r1,r2,c] | 0.1076 | 0.3662 | 0.3498 | 0.1693 | 0.3604 | 0.7063 |
| ML SE [r1,r2,c] | 0.0065 | 0.0102 | 0.0572 | 0.0079 | 0.0101 | 0.0575 |

**ML test Ctrl vs. Selection (r1 and r2 are line-specific)**

c - control or selection specific

| | | | | | | |
|---|---|---|---|---|---|---|
| Line1[r1,r2,c]: | 0.1185 | 0.3634 | 0.3498 | 0.1641 | 0.3630 | 0.7066 |
| Line2[r1,r2,c]: | 0.1055 | 0.3765 | 0.3498 | 0.1767 | 0.3548 | 0.7066 |
| Line3[r1,r2,c]: | 0.0987 | 0.3588 | 0.3498 | 0.1671 | 0.3636 | 0.7066 |

c-global

| | | | | | | |
|---|---|---|---|---|---|---|
| Line1[r1,r2,c]: | 0.1192 | 0.3656 | 0.5698 | 0.1634 | 0.3613 | 0.5698 |
| Line2[r1,r2,c]: | 0.1060 | 0.3784 | 0.5698 | 0.1756 | 0.3526 | 0.5698 |
| Line3[r1,r2,c]: | 0.0991 | 0.3604 | 0.5698 | 0.1664 | 0.3620 | 0.5698 |

ML ratio (df=14 –13=1): 17.5438

***ML test Ctrl vs. Selection (r1, r2 and c are all line-specific)**

| | | | | | | |
|---|---|---|---|---|---|---|
| Line1[r1,r2,c]: | 0.1187 | 0.3640 | 0.4013 | 0.1640 | 0.3627 | 0.6725 |
| Line2[r1,r2,c]: | 0.1053 | 0.3760 | 0.3030 | 0.1773 | 0.3560 | 0.8237 |
| Line3[r1,r2,c]: | 0.0987 | 0.3587 | 0.3391 | 0.1667 | 0.3627 | 0.6176 |

ML ratio (df=18 –13=5): 20.3889

ML ratio for heterogeneity (control+selection; df = 5–1=4): 20.3889 – 17.5438
………………………………………………………………………………………………………

## markers 1-3-4 (*net-b-pk*)

| | CONTROL | | | DESICCATION | | |
|---|---|---|---|---|---|---|
| **Lines (ML estimates)** | | | | | | |
| Line1 [r1,r2,c]: | 0.3907 | 0.0467 | 0.2194 | 0.3933 | 0.0560 | 0.4842 |
| Line2 [r1,r2,c]: | 0.3800 | 0.0627 | 0.1680 | 0.3667 | 0.0507 | 0.1436 |
| Line3 [r1,r2,c]: | 0.3667 | 0.0560 | 0.1948 | 0.3867 | 0.0587 | 0.1763 |
| Bailey test for 3 × 750 | | | | | | |
| Teta [r1,r2,c]: | 0.3793 | 0.0544 | 0.1894 | 0.3819 | 0.0548 | 0.2160 |
| SE [r1,r2,c]: | 0.0102 | 0.0048 | 0.0608 | 0.0102 | 0.0048 | 0.0630 |
| Chi2: | | 2.8233 | | | 5.6465 | |
| C Chi2: | | 0.1178 | | | 3.8553 | |

Bailey for 6 × 750:

| | | | |
|---|---|---|---|
| Teta: | 0.3806 | 0.0546 | 0.2022 |
| Chi2: | 8.5973 | | |
| C chi2: | 4.0654 | | |

**ML test Control vs. selection**
chi^2(ctrl + sel) - chi^2(ctrl) - chi^2(sel): 0.1275
C chi^2(ctrl + sel) - chi^2(ctrl) - chi^2(sel): **0.0923**

**ML test Ctrl Vs. Selection**

| | | | | | | |
|---|---|---|---|---|---|---|
| ML est [r1,r2,c] | 0.3791 | 0.0551 | 0.1915 | 0.3822 | 0.0551 | 0.2743 |
| ML SE [r1,r2,c] | 0.0102 | 0.0048 | 0.0610 | 0.0102 | 0.0048 | 0.0713 |

**ML test Ctrl vs. Selection (r1 and r2 are line-specific)**

c - control or selection specific

| | | | | | | |
|---|---|---|---|---|---|---|
| Line1[r1,r2,c]: | 0.3905 | 0.0466 | 0.1918 | 0.3918 | 0.0558 | 0.2743 |
| Line2[r1,r2,c]: | 0.3802 | 0.0627 | 0.1918 | 0.3674 | 0.0508 | 0.2743 |
| Line3[r1,r2,c]: | 0.3666 | 0.0560 | 0.1918 | 0.3874 | 0.0588 | 0.2743 |

c-global

| | | | | | | |
|---|---|---|---|---|---|---|
| Line1[r1,r2,c]: | 0.3907 | 0.0467 | 0.2332 | 0.3914 | 0.0557 | 0.2332 |

| | | | | | | |
|---|---|---|---|---|---|---|
| Line2[r1,r2,c]: | 0.3805 | 0.0627 | 0.2332 | 0.3672 | 0.0507 | 0.2332 |
| Line3[r1,r2,c]: | 0.3669 | 0.0560 | 0.2332 | 0.3871 | 0.0587 | 0.2332 |

ML ratio (df=14 –13=1): 0.7729

**<u>*ML test Ctrl vs. Selection (r1, r2 and c are all line-specific)</u>**

| | | | | | | |
|---|---|---|---|---|---|---|
| Line1[r1,r2,c]: | 0.3907 | 0.0467 | 0.2194 | 0.3933 | 0.0560 | 0.4842 |
| Line2[r1,r2,c]: | 0.3800 | 0.0627 | 0.1680 | 0.3667 | 0.0507 | 0.1436 |
| Line3[r1,r2,c]: | 0.3667 | 0.0560 | 0.1948 | 0.3867 | 0.0587 | 0.1763 |

ML ratio (df=18 –13=5): 5.374

ML ratio for heterogeneity (control+selection; df = 5–1=4): 5.374 – 0.7729
…………………………………………………………………………………………………………

## markers 1-3-5 (*net-b-cn*)

| | CONTROL | | | DESICCATION | | |
|---|---|---|---|---|---|---|
| **Lines (ML estimates)** | | | | | | |
| Line1 [r1,r2,c]: | 0.3907 | 0.0653 | 0.1567 | 0.3933 | 0.0800 | 0.4237 |
| Line2 [r1,r2,c]: | 0.3800 | 0.0853 | 0.1234 | 0.3667 | 0.0680 | 0.1070 |
| Line3 [r1,r2,c]: | 0.3667 | 0.0773 | 0.1881 | 0.3867 | 0.0760 | 0.1361 |
| Bailey test for 3 × 750 | | | | | | |
| Teta [r1,r2,c]: | 0.3793 | 0.0752 | 0.1497 | 0.3816 | 0.0742 | 0.1704 |
| SE [r1,r2,c]: | 0.0102 | 0.0056 | 0.0463 | 0.0102 | 0.0055 | 0.0484 |
| Chi2: | | 3.3596 | | | 7.6068 | |
| C Chi2: | | 0.3355 | | | 5.4475 | |

Bailey for 6 × 750:

| | | | |
|---|---|---|---|
| Teta: | 0.3804 | 0.0747 | 0.1596 |
| Chi2: | 11.0948 | | |
| C chi2: | 5.8787 | | |

**<u>ML test Control vs. selection</u>**

chi^2(ctrl + sel) - chi^2(ctrl) - chi^2(sel): 0.1284
C chi^2(ctrl + sel) - chi^2(ctrl) - chi^2(sel): **<u>0.0957</u>**

**<u>ML test Ctrl Vs. Selection</u>**

| | | | | | | |
|---|---|---|---|---|---|---|
| ML est [r1,r2,c] | 0.3791 | 0.0760 | 0.1543 | 0.3822 | 0.0747 | 0.2336 |
| ML SE [r1,r2,c] | 0.0102 | 0.0056 | 0.0470 | 0.0102 | 0.0055 | 0.0569 |

**<u>ML test Ctrl vs. Selection (r1 and r2 are line-specific)</u>**

c - control or selection specific

| | | | | | | |
|---|---|---|---|---|---|---|
| Line1[r1,r2,c]: | 0.3906 | 0.0653 | 0.1544 | 0.3913 | 0.0796 | 0.2335 |
| Line2[r1,r2,c]: | 0.3803 | 0.0854 | 0.1544 | 0.3677 | 0.0682 | 0.2335 |
| Line3[r1,r2,c]: | 0.3663 | 0.0773 | 0.1544 | 0.3876 | 0.0762 | 0.2335 |

c-global

| | | | | | | |
|---|---|---|---|---|---|---|
| Line1[r1,r2,c]: | 0.3910 | 0.0654 | 0.1938 | 0.3908 | 0.0795 | 0.1938 |
| Line2[r1,r2,c]: | 0.3808 | 0.0855 | 0.1938 | 0.3674 | 0.0681 | 0.1938 |
| Line3[r1,r2,c]: | 0.3667 | 0.0773 | 0.1938 | 0.3873 | 0.0761 | 0.1938 |

ML ratio (df=14 –13=1): 1.1538

**<u>*ML test Ctrl vs. Selection (r1, r2 and c are all line-specific)</u>**

| | | | | | | |
|---|---|---|---|---|---|---|
| Line1[r1,r2,c]: | 0.3907 | 0.0653 | 0.1567 | 0.3933 | 0.0800 | 0.4237 |
| Line2[r1,r2,c]: | 0.3800 | 0.0853 | 0.1234 | 0.3667 | 0.0680 | 0.1070 |
| Line3[r1,r2,c]: | 0.3667 | 0.0773 | 0.1881 | 0.3867 | 0.0760 | 0.1361 |

ML ratio (df=18 –13=5): 7.8346

ML ratio for heterogeneity (control+selection; df = 5–1=4): 7.8346 – 1.1538

………………………………………………………………………………………………………

## markers 2-3-4 (*dp-b-pk*)

**CONTROL** **DESICCATION**

**<u>Lines (ML estimates)</u>**

| | | | | | | |
|---|---|---|---|---|---|---|
| Line1 [r1,r2,c]: | 0.3040 | 0.0467 | 0.1880 | 0.2986 | 0.0560 | 0.3987 |
| Line2 [r1,r2,c]: | 0.2933 | 0.0627 | 0.0725 | 0.2881 | 0.0507 | 0.0000 |
| Line3 [r1,r2,c]: | 0.2840 | 0.0560 | 0.0838 | 0.2947 | 0.0587 | 0.2314 |

Bailey test for 3 × 750

| | | | | | | |
|---|---|---|---|---|---|---|
| Teta [r1,r2,c]: | 0.2939 | 0.0543 | 0.0939 | 0.2929 | 0.0547 | 0.0000 |
| SE [r1,r2,c]: | 0.0096 | 0.0048 | 0.0498 | 0.0096 | 0.0048 | 0.0003 |
| Chi2: | | 3.1395 | | | 9.7915 | |
| C Chi2: | | 0.6421 | | | 9.1213 | |

Bailey for 6 × 750:

| | | | |
|---|---|---|---|
| Teta: | 0.2932 | 0.0545 | 0.0000 |
| Chi2: | 16.4884 | | |
| C chi2: | 13.3277 | | |

**<u>ML test Control vs. selection</u>**

chi^2(ctrl + sel) - chi^2(ctrl) - chi^2(sel): 3.5575

C chi^2(ctrl + sel) - chi^2(ctrl) - chi^2(sel): **<u>3.5644</u>**

**<u>ML test Ctrl Vs. Selection</u>**

| | | | | | | |
|---|---|---|---|---|---|---|
| ML est [r1,r2,c] | 0.2938 | 0.0551 | 0.1098 | 0.2938 | 0.0551 | 0.2196 |
| ML SE [r1,r2,c] | 0.0096 | 0.0048 | 0.0538 | 0.0096 | 0.0048 | 0.0745 |

**<u>ML test Ctrl vs. Selection (r1 and r2 are line-specific)</u>**

c - control or selection specific

| | | | | | | |
|---|---|---|---|---|---|---|
| Line1[r1,r2,c]: | 0.3037 | 0.0466 | 0.1100 | 0.2979 | 0.0559 | 0.2197 |
| Line2[r1,r2,c]: | 0.2935 | 0.0627 | 0.1100 | 0.2888 | 0.0508 | 0.2197 |
| Line3[r1,r2,c]: | 0.2841 | 0.0560 | 0.1100 | 0.2946 | 0.0587 | 0.2197 |

c-global

| | | | | | | |
|---|---|---|---|---|---|---|
| Line1[r1,r2,c]: | 0.3039 | 0.0466 | 0.1647 | 0.2976 | 0.0558 | 0.1647 |
| Line2[r1,r2,c]: | 0.2938 | 0.0628 | 0.1647 | 0.2886 | 0.0508 | 0.1647 |
| Line3[r1,r2,c]: | 0.2843 | 0.0561 | 0.1647 | 0.2944 | 0.0586 | 0.1647 |

ML ratio (df=14 –13=1): 1.4367

***ML test Ctrl vs. Selection (r1, r2 and c are all line-specific)**

| | | | | | | |
|---|---|---|---|---|---|---|
| Line1[r1,r2,c]: | 0.3040 | 0.0467 | 0.1880 | 0.2986 | 0.0560 | 0.3987 |
| Line2[r1,r2,c]: | 0.2933 | 0.0627 | 0.0725 | 0.2881 | 0.0507 | 0.0000 |
| Line3[r1,r2,c]: | 0.2840 | 0.0560 | 0.0838 | 0.2947 | 0.0587 | 0.2314 |

ML ratio (df=18 –13=5): 8.8791

ML ratio for heterogeneity (control+selection; df = 5–1=4): 8.8791 – 1.4367

……………………………………………………………………………………………………………

## markers 2-3-5 (*dp-b-cn*)

| | CONTROL | | | DESICCATION | | |
|---|---|---|---|---|---|---|
| **Lines (ML estimates)** | | | | | | |
| Line1 [r1,r2,c]: | 0.3040 | 0.0653 | 0.1343 | 0.2987 | 0.0800 | 0.3348 |
| Line2 [r1,r2,c]: | 0.2933 | 0.0853 | 0.0533 | 0.2882 | 0.0680 | 0.0000 |
| Line3 [r1,r2,c]: | 0.2840 | 0.0773 | 0.0607 | 0.2947 | 0.0760 | 0.1786 |
| Bailey test for 3 × 750 | | | | | | |
| Teta [r1,r2,c]: | 0.2939 | 0.0751 | 0.0684 | 0.2927 | 0.0740 | 0.0000 |
| SE [r1,r2,c]: | 0.0096 | 0.0056 | 0.0364 | 0.0096 | 0.0055 | 0.0003 |
| Chi2: | | 3.3869 | | | 11.1433 | |
| C Chi2: | | 0.6071 | | | 10.1339 | |

Bailey for 6 × 750:

| | | | |
|---|---|---|---|
| Teta: | 0.2931 | 0.0745 | 0.0000 |
| Chi2: | 18.0853 | | |
| C chi2: | 14.2908 | | |

**ML test Control vs. selection**

chi^2(ctrl + sel) - chi^2(ctrl) - chi^2(sel): 3.5550

C chi^2(ctrl + sel) - chi^2(ctrl) - chi^2(sel): **3.5497**

**ML test Ctrl Vs. Selection**

| | | | | | | |
|---|---|---|---|---|---|---|
| ML est [r1,r2,c] | 0.2938 | 0.0760 | 0.0796 | 0.2938 | 0.0747 | 0.1824 |
| ML SE [r1,r2,c] | 0.0096 | 0.0056 | 0.0392 | 0.0096 | 0.0055 | 0.0586 |

**ML test Ctrl vs. Selection (r1 and r2 are line-specific)**

c - control or selection specific

| | | | | | | |
|---|---|---|---|---|---|---|
| Line1[r1,r2,c]: | 0.3037 | 0.0653 | 0.0798 | 0.2977 | 0.0797 | 0.1824 |
| Line2[r1,r2,c]: | 0.2935 | 0.0854 | 0.0798 | 0.2889 | 0.0682 | 0.1824 |
| Line3[r1,r2,c]: | 0.2841 | 0.0774 | 0.0798 | 0.2947 | 0.0760 | 0.1824 |

c-global

| | | | | | | |
|---|---|---|---|---|---|---|
| Line1[r1,r2,c]: | 0.3040 | 0.0653 | 0.1307 | 0.2973 | 0.0796 | 0.1307 |
| Line2[r1,r2,c]: | 0.2939 | 0.0855 | 0.1307 | 0.2887 | 0.0682 | 0.1307 |
| Line3[r1,r2,c]: | 0.2844 | 0.0774 | 0.130 | 0.2944 | 0.0759 | 0.1307 |

ML ratio (df=14 –13=1): 2.1623

***ML test Ctrl vs. Selection (r1, r2 and c are all line-specific)**

| | | | | | | |
|---|---|---|---|---|---|---|
| Line1[r1,r2,c]: | 0.3040 | 0.0653 | 0.1343 | 0.2987 | 0.0800 | 0.3348 |
| Line2[r1,r2,c]: | 0.2933 | 0.0853 | 0.0533 | 0.2882 | 0.0680 | 0.0000 |
| Line3[r1,r2,c]: | 0.2840 | 0.0773 | 0.0607 | 0.2947 | 0.0760 | 0.1786 |

ML ratio (df=18 –13=5): 10.4707

ML ratio for heterogeneity (control+selection; df = 5–1=4): 10.4707 – 2.1623
……………………………………………………………………………………………………

## markers 1-4-5 (*net-pk-cn*)

| | CONTROL | | | DESICCATION | | |
|---|---|---|---|---|---|---|
| **Lines (ML estimates)** | | | | | | |
| Line1 [r1,r2,c]: | 0.4294 | 0.0187 | 0.0000 | 0.4280 | 0.0240 | 0.2600 |
| Line2 [r1,r2,c]: | 0.4348 | 0.0227 | 0.0000 | 0.4120 | 0.0173 | 0.0000 |
| Line3 [r1,r2,c]: | 0.4147 | 0.0213 | 0.1508 | 0.4374 | 0.0173 | 0.0000 |
| Bailey test for 3 × 750 | | | | | | |
| Teta [r1,r2,c]: | 0.4261 | 0.0208 | 0.0000 | 0.4255 | 0.0191 | 0.0000 |
| SE [r1,r2,c]: | 0.0104 | 0.0030 | 0.0006 | 0.0104 | 0.0029 | 0.0002 |
| Chi2: | | 2.1157 | | | 4.3428 | |
| C Chi2: | | 1.0729 | | | 2.2755 | |

Bailey for 6 × 750:

| | | | |
|---|---|---|---|
| Teta: | 0.4258 | 0.0199 | 0.0000 |
| Chi2: | 6.6298 | | |
| C chi2: | 3.3486 | | |

**ML test Control vs. selection**
chi^2(ctrl + sel) - chi^2(ctrl) - chi^2(sel): 0.1713
C chi^2(ctrl + sel) - chi^2(ctrl) - chi^2(sel): **0.0001**

**ML test Ctrl Vs. Selection**

| | | |
|---|---|---|
| ML est [r1,r2,c] | 0.4262 0.0209 0.0499 | 0.4258 0.0196 0.1068 |
| ML SE [r1,r2,c] | 0.0104 0.0030 0.0493 | 0.0104 0.0029 0.0737 |

**ML test Ctrl vs. Selection (r1 and r2 are line-specific)**

c - control or selection specific

| | | | | | | |
|---|---|---|---|---|---|---|
| Line1[r1,r2,c]: | 0.4295 | 0.0187 | 0.0499 | 0.4274 | 0.0240 | 0.1067 |
| Line2[r1,r2,c]: | 0.4349 | 0.0227 | 0.0499 | 0.4123 | 0.0173 | 0.1067 |
| Line3[r1,r2,c]: | 0.4143 | 0.0213 | 0.0499 | 0.4377 | 0.0173 | 0.1067 |

c-global

| | | | | | | |
|---|---|---|---|---|---|---|
| Line1[r1,r2,c]: | 0.4296 | 0.0187 | 0.0773 | 0.4272 | 0.0240 | 0.0773 |
| Line2[r1,r2,c]: | 0.4350 | 0.0227 | 0.0773 | 0.4122 | 0.0173 | 0.0773 |
| Line3[r1,r2,c]: | 0.4144 | 0.0213 | 0.0773 | 0.4376 | 0.0173 | 0.0773 |

ML ratio (df=14 –13=1): 0.4259

***ML test Ctrl vs. Selection (r1, r2 and c are all line-specific)**

| | | | | | | |
|---|---|---|---|---|---|---|
| Line1[r1,r2,c]: | 0.4294 | 0.0187 | 0.0000 | 0.4280 | 0.0240 | 0.2600 |
| Line2[r1,r2,c]: | 0.4348 | 0.0227 | 0.0000 | 0.4120 | 0.0173 | 0.0000 |
| Line3[r1,r2,c]: | 0.4147 | 0.0213 | 0.1508 | 0.4374 | 0.0173 | 0.0000 |

ML ratio (df=18 –13=5): 6.3868

ML ratio for heterogeneity (control+selection; df = 5–1=4): 6.3868 – 0.4259

……………………………………………………………………………………………………

## Desiccation/2R chromosome

**markers 1-2-4 (*cn-kn-px*)**

| | CONTROL | DESICCATION |
|---|---|---|
| **Lines (ML estimates)** | | |
| Line1 [r1,r2,c]: | 0.1347 0.2600 0.4950 | 0.2133 0.3373 0.8893 |
| Line2 [r1,r2,c]: | 0.1053 0.2600 0.2434 | 0.1760 0.2840 0.8803 |
| Line3 [r1,r2,c]: | 0.1267 0.2613 0.3625 | 0.1840 0.3040 0.9296 |
| Bailey test for 3 × 750 | | |
| Teta [r1,r2,c]: | 0.1210 0.2603 0.3524 | 0.1902 0.3077 0.8976 |
| SE [r1,r2,c]: | 0.0069 0.0093 0.0647 | 0.0083 0.0097 0.0629 |
| Chi2: | 5.7465 | 9.1510 |
| C Chi2: | 2.4607 | 0.1062 |

Bailey for 6 × 750:

Teta: 0.1483 0.2812 0.6353

Chi2: 106.2365

C chi2: 39.2665

**ML test Control vs. selection**
chi^2(ctrl + sel) - chi^2(ctrl) - chi^2(sel): 91.3391
C chi^2(ctrl + sel) - chi^2(ctrl) - chi^2(sel): **36.6996**

**ML test Ctrl Vs. Selection**

| | | | | | | |
|---|---|---|---|---|---|---|
| ML est [r1,r2,c] | 0.1222 | 0.2604 | 0.3770 | 0.1911 | 0.3084 | 0.9048 |
| ML SE [r1,r2,c] | 0.0069 | 0.0093 | 0.0670 | 0.0083 | 0.0097 | 0.0635 |

**ML test Ctrl vs. Selection (r1 and r2 are line-specific)**

c - control or selection specific

| | | | | | | |
|---|---|---|---|---|---|---|
| Line1[r1,r2,c]: | 0.1343 | 0.2592 | 0.3775 | 0.2134 | 0.3374 | 0.8993 |
| Line2[r1,r2,c]: | 0.1056 | 0.2607 | 0.3775 | 0.1760 | 0.2840 | 0.8993 |
| Line3[r1,r2,c]: | 0.1267 | 0.2614 | 0.3775 | 0.1839 | 0.3039 | 0.8993 |

c-global

| | | | | | | |
|---|---|---|---|---|---|---|
| Line1[r1,r2,c]: | 0.1351 | 0.2608 | 0.7303 | 0.2123 | 0.3356 | 0.7303 |
| Line2[r1,r2,c]: | 0.1058 | 0.2613 | 0.7303 | 0.1755 | 0.2831 | 0.7303 |
| Line3[r1,r2,c]: | 0.1272 | 0.2625 | 0.7303 | 0.1832 | 0.3026 | 0.7303 |

ML ratio (df=14 –13=1): 26.9292

***ML test Ctrl vs. Selection (r1, r2 and c are all line-specific)**

| | | | | | | |
|---|---|---|---|---|---|---|
| Line1[r1,r2,c]: | 0.1347 | 0.2600 | 0.4950 | 0.2133 | 0.3373 | 0.8893 |
| Line2[r1,r2,c]: | 0.1053 | 0.2600 | 0.2434 | 0.1760 | 0.2840 | 0.8803 |
| Line3[r1,r2,c]: | 0.1267 | 0.2613 | 0.3625 | 0.1840 | 0.3040 | 0.9296 |

ML ratio (df=18 –13=5): 29.3799

ML ratio for heterogeneity (control+selection; df = 5–1=4): 29.3799 – 26.9292
……………………………………………………………………………………………………

## markers 1-2-5 (*cn-kn-sp*)

| | CONTROL | | | DESICCATION | | |
|---|---|---|---|---|---|---|
| **Lines (ML estimates)** | | | | | | |
| Line1 [r1,r2,c]: | 0.1347 | 0.3307 | 0.3892 | 0.2133 | 0.3880 | 0.8215 |
| Line2 [r1,r2,c]: | 0.1053 | 0.3187 | 0.3178 | 0.1760 | 0.3493 | 0.8024 |
| Line3 [r1,r2,c]: | 0.1267 | 0.3107 | 0.3727 | 0.1840 | 0.3600 | 0.8052 |
| Bailey test for 3 × 750 | | | | | | |
| Teta [r1,r2,c]: | 0.1211 | 0.3201 | 0.3604 | 0.1901 | 0.3656 | 0.8096 |
| SE [r1,r2,c]: | 0.0069 | 0.0098 | 0.0585 | 0.0083 | 0.0101 | 0.0546 |
| Chi2: | 4.5559 | | | 6.6584 | | |
| C Chi2: | 0.2702 | | | 0.0265 | | |

Bailey for 6 × 750:

| | | | |
|---|---|---|---|
| Teta: | 0.1484 | 0.3407 | 0.6003 |
| Chi2: | 95.4251 | | |
| C chi2: | 32.0217 | | |

**ML test Control vs. selection**

chi^2(ctrl + sel) - chi^2(ctrl) - chi^2(sel): 84.2107
C chi^2(ctrl + sel) - chi^2(ctrl) - chi^2(sel): **31.7250**

**ML test Ctrl Vs. Selection**

| | | | | | | |
|---|---|---|---|---|---|---|
| ML est [r1,r2,c] | 0.1222 | 0.3200 | 0.3636 | 0.1911 | 0.3658 | 0.8138 |
| ML SE [r1,r2,c] | 0.0069 | 0.0098 | 0.0588 | 0.0083 | 0.0102 | 0.0549 |

**ML test Ctrl vs. Selection (r1 and r2 are line-specific)**

c - control or selection specific

| | | | | | | |
|---|---|---|---|---|---|---|
| Line1[r1,r2,c]: | 0.1345 | 0.3304 | 0.3635 | 0.2133 | 0.3879 | 0.8111 |
| Line2[r1,r2,c]: | 0.1055 | 0.3190 | 0.3635 | 0.1760 | 0.3494 | 0.8111 |
| Line3[r1,r2,c]: | 0.1266 | 0.3106 | 0.3635 | 0.1840 | 0.3600 | 0.8111 |

c-global

| | | | | | | |
|---|---|---|---|---|---|---|
| Line1[r1,r2,c]: | 0.1355 | 0.3326 | 0.6609 | 0.2118 | 0.3852 | 0.6609 |
| Line2[r1,r2,c]: | 0.1059 | 0.3204 | 0.6609 | 0.1752 | 0.3478 | 0.6609 |
| Line3[r1,r2,c]: | 0.1273 | 0.3123 | 0.6609 | 0.1831 | 0.3582 | 0.6609 |

ML ratio (df=14 –13=1): 26.7244

***ML test Ctrl vs. Selection (r1, r2 and c are all line-specific)**

| | | | | | | |
|---|---|---|---|---|---|---|
| Line1[r1,r2,c]: | 0.1347 | 0.3307 | 0.3892 | 0.2133 | 0.3880 | 0.8215 |
| Line2[r1,r2,c]: | 0.1053 | 0.3187 | 0.3178 | 0.1760 | 0.3493 | 0.8024 |
| Line3[r1,r2,c]: | 0.1267 | 0.3107 | 0.3727 | 0.1840 | 0.3600 | 0.8052 |

ML ratio (df=18 –13=5): 27.0089

ML ratio for heterogeneity (control+selection; df = 5–1=4): 27.0089 – 26.7244

…………………………………………………………………………………………………

## markers 1-3-4 (*cn-c-px*)

| | CONTROL | | | DESICCATION | | |
|---|---|---|---|---|---|---|
| **Lines (ML estimates)** | | | | | | |
| Line1 [r1,r2,c]: | 0.1680 | 0.2373 | 0.5685 | 0.2280 | 0.3227 | 0.8699 |
| Line2 [r1,r2,c]: | 0.1200 | 0.2453 | 0.2264 | 0.1973 | 0.2627 | 0.8489 |
| Line3 [r1,r2,c]: | 0.1467 | 0.2413 | 0.3390 | 0.1987 | 0.2867 | 0.8897 |

Bailey test for 3 × 750

| | | |
|---|---|---|
| Teta [r1,r2,c]: | 0.1426 0.2409 0.3521 | 0.2074 0.2895 0.8686 |
| SE [r1,r2,c]: | 0.0074 0.0090 0.0614 | 0.0085 0.0095 0.0618 |
| Chi2: | 11.7669 | 9.6860 |
| C Chi2: | 4.9019 | 0.0631 |

Bailey for 6 × 750:

| | |
|---|---|
| Teta: | 0.1692 0.2624 0.6108 |
| Chi2: | 104.2492 |
| C chi2: | 40.3484 |

**ML test Control vs. selection**

chi^2(ctrl + sel) - chi^2(ctrl) - chi^2(sel): 82.7962

C chi^2(ctrl + sel) - chi^2(ctrl) - chi^2(sel): **35.3834**

**ML test Ctrl Vs. Selection**

| | | |
|---|---|---|
| ML est [r1,r2,c] | 0.1449 0.2413 0.3940 | 0.2080 0.2907 0.8748 |
| ML SE [r1,r2,c] | 0.0074 0.0090 0.0651 | 0.0086 0.0096 0.0623 |

**ML test Ctrl vs. Selection (r1 and r2 are line-specific)**

c - control or selection specific

| | | | | | | |
|---|---|---|---|---|---|---|
| Line1[r1,r2,c]: | 0.1672 | 0.2362 | 0.3958 | 0.2280 | 0.3227 | 0.8703 |
| Line2[r1,r2,c]: | 0.1204 | 0.2462 | 0.3958 | 0.1974 | 0.2627 | 0.8703 |
| Line3[r1,r2,c]: | 0.1469 | 0.2417 | 0.3958 | 0.1986 | 0.2866 | 0.8703 |

c-global

| | | | | | | |
|---|---|---|---|---|---|---|
| Line1[r1,r2,c]: | 0.1684 | 0.2379 | 0.7088 | 0.2268 | 0.3209 | 0.7088 |
| Line2[r1,r2,c]: | 0.1207 | 0.2467 | 0.7088 | 0.1967 | 0.2619 | 0.7088 |
| Line3[r1,r2,c]: | 0.1474 | 0.2426 | 0.7088 | 0.1978 | 0.2854 | 0.7088 |

ML ratio (df=14 –13=1): 24.0400

***ML test Ctrl vs. Selection (r1, r2 and c are all line-specific)**

| | | | | | | |
|---|---|---|---|---|---|---|
| Line1[r1,r2,c]: | 0.1680 | 0.2373 | 0.5685 | 0.2280 | 0.3227 | 0.8699 |
| Line2[r1,r2,c]: | 0.1200 | 0.2453 | 0.2264 | 0.1973 | 0.2627 | 0.8489 |
| Line3[r1,r2,c]: | 0.1467 | 0.2413 | 0.3390 | 0.1987 | 0.2867 | 0.8897 |

ML ratio (df=18 –13=5): 28.965

ML ratio for heterogeneity (control+selection; df = 5–1=4): 28.965 – 24.0400

……………………………………………………………………………………………………

## markers 1-3-5 (*cn-c-sp*)

| | CONTROL | DESICCATION |
|---|---|---|
| **Lines (ML estimates)** | | |
| Line1 [r1,r2,c]: | 0.1680 0.3080 0.4381 | 0.2280 0.3733 0.7989 |

| | | | | | | |
|---|---|---|---|---|---|---|
| Line2 [r1,r2,c]: | 0.1200 | 0.3040 | 0.2924 | 0.1973 | 0.3280 | 0.7622 |
| Line3 [r1,r2,c]: | 0.1467 | 0.2907 | 0.3440 | 0.1987 | 0.3427 | 0.7638 |

Bailey test for 3 × 750

| | | | | | | |
|---|---|---|---|---|---|---|
| Teta [r1,r2,c]: | 0.1425 | 0.3010 | 0.3578 | 0.2073 | 0.3476 | 0.7762 |
| SE [r1,r2,c]: | 0.0074 | 0.0097 | 0.0551 | 0.0085 | 0.0100 | 0.0532 |
| Chi2: | | 9.0034 | | | 6.7239 | |
| C Chi2: | | 1.2107 | | | 0.1104 | |

Bailey for 6 × 750:

| | | | |
|---|---|---|---|
| Teta: | 0.1692 | 0.3220 | 0.5738 |
| Chi2: | 90.7953 | | |
| C chi2: | 31.2916 | | |

**ML test Control vs. selection**

chi^2(ctrl + sel) - chi^2(ctrl) - chi^2(sel): 75.0680

C chi^2(ctrl + sel) - chi^2(ctrl) - chi^2(sel): **29.9705**

**ML test Ctrl Vs. Selection**

| | | | | | | |
|---|---|---|---|---|---|---|
| ML est [r1,r2,c] | 0.1449 | 0.3009 | 0.3670 | 0.2080 | 0.3480 | 0.7798 |
| ML SE [r1,r2,c] | 0.0074 | 0.0097 | 0.0558 | 0.0086 | 0.0100 | 0.0535 |

**ML test Ctrl vs. Selection (r1 and r2 are line-specific)**

c - control or selection specific

| | | | | | | |
|---|---|---|---|---|---|---|
| Line1[r1,r2,c]: | 0.1675 | 0.3071 | 0.3675 | 0.2278 | 0.3731 | 0.7777 |
| Line2[r1,r2,c]: | 0.1202 | 0.3046 | 0.3675 | 0.1974 | 0.3281 | 0.7777 |
| Line3[r1,r2,c]: | 0.1468 | 0.2909 | 0.3675 | 0.1987 | 0.3428 | 0.7777 |

c-global

| | | | | | | |
|---|---|---|---|---|---|---|
| Line1[r1,r2,c]: | 0.1689 | 0.3096 | 0.6324 | 0.2262 | 0.3703 | 0.6324 |
| Line2[r1,r2,c]: | 0.1207 | 0.3059 | 0.6324 | 0.1964 | 0.3265 | 0.6324 |
| Line3[r1,r2,c]: | 0.1476 | 0.2925 | 0.6324 | 0.1977 | 0.3410 | 0.6324 |

ML ratio (df=14 –13=1): 24.8422

***ML test Ctrl vs. Selection (r1, r2 and c are all line-specific)**

| | | | | | | |
|---|---|---|---|---|---|---|
| Line1[r1,r2,c]: | 0.1680 | 0.3080 | 0.4381 | 0.2280 | 0.3733 | 0.7989 |
| Line2[r1,r2,c]: | 0.1200 | 0.3040 | 0.2924 | 0.1973 | 0.3280 | 0.7622 |
| Line3[r1,r2,c]: | 0.1467 | 0.2907 | 0.3440 | 0.1987 | 0.3427 | 0.7638 |

ML ratio (df=18 –13=5): 26.1520

ML ratio for heterogeneity (control+selection; df = 5–1=4): 26.1520 – 24.8422

……………………………………………………………………………………………………

## markers 1-4-5 (*cn-px-sp*)

**CONTROL** **DESICCATION**

**Lines (ML estimates)**

| | | |
|---|---|---|
| Line1 [r1,r2,c]: | 0.3602 0.0707 0.0000 | 0.4227 0.0560 0.2817 |
| Line2 [r1,r2,c]: | 0.3520 0.0587 0.1937 | 0.3720 0.0653 0.2194 |
| Line3 [r1,r2,c]: | 0.3640 0.0547 0.2680 | 0.3840 0.0560 0.0620 |

Bailey test for 3 × 750

| | | |
|---|---|---|
| Teta [r1,r2,c]: | 0.3583 0.0605 0.0000 | 0.3921 0.0587 0.1325 |
| SE [r1,r2,c]: | 0.0101 0.0050 0.0005 | 0.0103 0.0050 0.0481 |
| Chi2: | 9.9202 | 8.3310 |
| C Chi2: | 7.8015 | 3.6620 |

Bailey for 6 × 750:

| | |
|---|---|
| Teta: | 0.3744 0.0596 0.0000 |
| Chi2: | 31.0006 |
| C chi2: | 19.0988 |

**ML test Control vs. selection**

chi^2(ctrl + sel) - chi^2(ctrl) - chi^2(sel): 12.7494
C chi^2(ctrl + sel) - chi^2(ctrl) - chi^2(sel): **7.6353**

**ML test Ctrl Vs. Selection**

| | | |
|---|---|---|
| ML est [r1,r2,c] | 0.3587 0.0613 0.1414 | 0.3929 0.0591 0.1914 |
| ML SE [r1,r2,c] | 0.0101 0.0051 0.0518 | 0.0103 0.0050 0.0578 |

**ML test Ctrl vs. Selection (r1 and r2 are line-specific)**

c - control or selection specific

| | | | | | | |
|---|---|---|---|---|---|---|
| Line1[r1,r2,c]: | 0.3612 | 0.0709 | 0.1413 | 0.4219 | 0.0559 | 0.1922 |
| Line2[r1,r2,c]: | 0.3517 | 0.0586 | 0.1413 | 0.3718 | 0.0653 | 0.1922 |
| Line3[r1,r2,c]: | 0.3632 | 0.0545 | 0.1413 | 0.3850 | 0.0561 | 0.1922 |

c-global

| | | | | | | |
|---|---|---|---|---|---|---|
| Line1[r1,r2,c]: | 0.3614 | 0.0709 | 0.1674 | 0.4216 | 0.0559 | 0.1674 |
| Line2[r1,r2,c]: | 0.3518 | 0.0586 | 0.1674 | 0.3716 | 0.0653 | 0.1674 |
| Line3[r1,r2,c]: | 0.3633 | 0.0546 | 0.1674 | 0.3848 | 0.0561 | 0.1674 |

ML ratio (df=14 –13=1): 0.4273

***ML test Ctrl vs. Selection (r1, r2 and c are all line-specific)**

| | | | | | | |
|---|---|---|---|---|---|---|
| Line1[r1,r2,c]: | 0.3602 | 0.0707 | 0.0000 | 0.4227 | 0.0560 | 0.2817 |
| Line2[r1,r2,c]: | 0.3520 | 0.0587 | 0.1937 | 0.3720 | 0.0653 | 0.2194 |
| Line3[r1,r2,c]: | 0.3640 | 0.0547 | 0.2680 | 0.3840 | 0.0560 | 0.0620 |

ML ratio (df=18 –13=5): 10.6073

ML ratio for heterogeneity (control+selection; df = 5–1=4): 10.6073 – 0.4273

……………………………………………………………………………………………………

**<u>markers 2-4-5 (*kn-px-sp*)</u>**

| | CONTROL | | | DESICCATION | | |
|---|---|---|---|---|---|---|
| **<u>Lines (ML estimates)</u>** | | | | | | |
| Line1 [r1,r2,c]: | 0.2602 | 0.0707 | 0.0000 | 0.3373 | 0.0560 | 0.1412 |
| Line2 [r1,r2,c]: | 0.2601 | 0.0587 | 0.0000 | 0.2842 | 0.0653 | 0.0000 |
| Line3 [r1,r2,c]: | 0.2613 | 0.0547 | 0.1867 | 0.3042 | 0.0560 | 0.0000 |
| | | | | | | |
| Bailey test for 3 × 750 | | | | | | |
| Teta [r1,r2,c]: | 0.2606 | 0.0606 | 0.0000 | 0.3075 | 0.0588 | 0.0000 |
| SE [r1,r2,c]: | 0.0092 | 0.0050 | 0.0002 | 0.0097 | 0.0050 | 0.0004 |
| Chi2: | | 3.9022 | | | 7.3841 | |
| C Chi2: | | 2.1309 | | | 2.1244 | |

Bailey for 6 × 750:

| | | | |
|---|---|---|---|
| Teta: | 0.2829 | 0.0598 | 0.0000 |
| Chi2: | 23.5790 | | |
| C chi2: | 4.2554 | | |

**<u>ML test Control vs. selection</u>**

chi^2(ctrl + sel) - chi^2(ctrl) - chi^2(sel): 12.2927

C chi^2(ctrl + sel) - chi^2(ctrl) - chi^2(sel): **<u>0.0001</u>**

**<u>ML test Ctrl Vs. Selection</u>**

| | | | | | | |
|---|---|---|---|---|---|---|
| ML est [r1,r2,c] | 0.2604 | 0.0613 | 0.0556 | 0.3084 | 0.0591 | 0.0488 |
| ML SE [r1,r2,c] | 0.0093 | 0.0051 | 0.0390 | 0.0097 | 0.0050 | 0.0342 |

**<u>ML test Ctrl vs. Selection (r1 and r2 are line-specific)</u>**

| | | | | | | |
|---|---|---|---|---|---|---|
| c - control or selection specific | | | | | | |
| Line1[r1,r2,c]: | 0.2603 | 0.0707 | 0.0556 | 0.3368 | 0.0559 | 0.0491 |
| Line2[r1,r2,c]: | 0.2602 | 0.0587 | 0.0556 | 0.2842 | 0.0654 | 0.0491 |
| Line3[r1,r2,c]: | 0.2609 | 0.0546 | 0.0556 | 0.3042 | 0.0560 | 0.0491 |
| | | | | | | |
| c-global | | | | | | |
| Line1[r1,r2,c]: | 0.2602 | 0.0707 | 0.0521 | 0.3368 | 0.0559 | 0.0521 |
| Line2[r1,r2,c]: | 0.2602 | 0.0587 | 0.0521 | 0.2843 | 0.0654 | 0.0521 |
| Line3[r1,r2,c]: | 0.2609 | 0.0546 | 0.0521 | 0.3043 | 0.0560 | 0.0521 |

ML ratio (df=14 –13=1): 0.0163

**<u>*ML test Ctrl vs. Selection (r1, r2 and c are all line-specific)</u>**

| | | | | | | |
|---|---|---|---|---|---|---|
| Line1[r1,r2,c]: | 0.2602 | 0.0707 | 0.0000 | 0.3373 | 0.0560 | 0.1412 |
| Line2[r1,r2,c]: | 0.2601 | 0.0587 | 0.0000 | 0.2842 | 0.0653 | 0.0000 |
| Line3[r1,r2,c]: | 0.2613 | 0.0547 | 0.1867 | 0.3042 | 0.0560 | 0.0000 |

ML ratio (df=18 –13=5): 9.2560

ML ratio for heterogeneity (control+selection; df = 5–1=4): 9.2560 – 0.0163

……………………………………………………………………………………………………

**markers 3-4-5 (*c-px-sp*)**

| | CONTROL | | | DESICCATION | | |
|---|---|---|---|---|---|---|
| **Lines (ML estimates)** | | | | | | |
| Line1 [r1,r2,c]: | 0.2374 | 0.0707 | 0.0000 | 0.3227 | 0.0560 | 0.1476 |
| Line2 [r1,r2,c]: | 0.2454 | 0.0587 | 0.0000 | 0.2628 | 0.0653 | 0.0000 |
| Line3 [r1,r2,c]: | 0.2413 | 0.0547 | 0.2021 | 0.2868 | 0.0560 | 0.0000 |
| Bailey test for 3 × 750 | | | | | | |
| Teta [r1,r2,c]: | 0.2415 | 0.0606 | 0.0000 | 0.2894 | 0.0588 | 0.0000 |
| SE [r1,r2,c]: | 0.0090 | 0.0050 | 0.0002 | 0.0095 | 0.0050 | 0.0003 |
| Chi2: | | 3.9437 | | | 8.8669 | |
| C Chi2: | | 2.1324 | | | 2.1249 | |

Bailey for 6 × 750:

| | | | |
|---|---|---|---|
| Teta: | 0.2641 | 0.0599 | 0.0000 |
| Chi2: | 26.1933 | | |
| C chi2: | 4.2573 | | |

**ML test Control vs. selection**

chi^2(ctrl + sel) - chi^2(ctrl) - chi^2(sel): 13.3827

C chi^2(ctrl + sel) - chi^2(ctrl) - chi^2(sel): **0.0000**

**ML test Ctrl Vs. Selection**

| | | | | | | |
|---|---|---|---|---|---|---|
| ML est [r1,r2,c] | 0.2413 | 0.0613 | 0.0601 | 0.2907 | 0.0591 | 0.0517 |
| ML SE [r1,r2,c] | 0.0090 | 0.0051 | 0.0421 | 0.0096 | 0.0050 | 0.0362 |

**ML test Ctrl vs. Selection (r1 and r2 are line-specific)**

| | | | | | | |
|---|---|---|---|---|---|---|
| c - control or selection specific | | | | | | |
| Line1[r1,r2,c]: | 0.2376 | 0.0707 | 0.0601 | 0.3221 | 0.0559 | 0.0521 |
| Line2[r1,r2,c]: | 0.2455 | 0.0587 | 0.0601 | 0.2629 | 0.0654 | 0.0521 |
| Line3[r1,r2,c]: | 0.2409 | 0.0546 | 0.0601 | 0.2869 | 0.0560 | 0.0521 |
| c-global | | | | | | |
| Line1[r1,r2,c]: | 0.2375 | 0.0707 | 0.0558 | 0.3222 | 0.0559 | 0.0558 |
| Line2[r1,r2,c]: | 0.2455 | 0.0587 | 0.0558 | 0.2629 | 0.0654 | 0.0558 |
| Line3[r1,r2,c]: | 0.2409 | 0.0546 | 0.0558 | 0.2869 | 0.0560 | 0.0558 |

ML ratio (df=14 –13=1): 0.0208

***ML test Ctrl vs. Selection (r1, r2 and c are all line-specific)**

| | | | | | | |
|---|---|---|---|---|---|---|
| Line1[r1,r2,c]: | 0.2374 | 0.0707 | 0.0000 | 0.3227 | 0.0560 | 0.1476 |

| | | | | | | |
|---|---|---|---|---|---|---|
| Line2[r1,r2,c]: | 0.2454 | 0.0587 | 0.0000 | 0.2628 | 0.0653 | 0.0000 |
| Line3[r1,r2,c]: | 0.2413 | 0.0547 | 0.2021 | 0.2868 | 0.0560 | 0.0000 |

ML ratio (df=18 –13=5): 9.2083

ML ratio for heterogeneity (control+selection; df = 5–1=4): 9.2083 – 0.0208
……………………………………………………………………………………………………

# <u>3 chromosome</u>

## <u>markers 1-2-3 (*ru-h-th*)</u>

| | CONTROL | | | DESICCATION | | |
|---|---|---|---|---|---|---|
| **<u>Lines (ML estimates)</u>** | | | | | | |
| Line1 [r1,r2,c]: | 0.1973 | 0.1307 | 0.7757 | 0.2027 | 0.2267 | 1.1320 |
| Line2 [r1,r2,c]: | 0.1947 | 0.1427 | 0.8642 | 0.2147 | 0.1907 | 1.1727 |
| Line3 [r1,r2,c]: | 0.1733 | 0.1613 | 0.8106 | 0.2053 | 0.2187 | 1.0097 |
| Bailey test for 3 × 750 | | | | | | |
| Teta [r1,r2,c]: | 0.1880 | 0.1440 | 0.8166 | 0.2077 | 0.2112 | 1.1001 |
| SE [r1,r2,c]: | 0.0082 | 0.0074 | 0.0995 | 0.0085 | 0.0086 | 0.0814 |
| Chi2: | | 4.5686 | | | 4.4819 | |
| C Chi2: | | 0.1317 | | | 0.7293 | |

Bailey for 6 × 750:

| | | | |
|---|---|---|---|
| Teta: | 0.1965 | 0.1724 | 0.9933 |
| Chi2: | 51.7661 | | |
| C chi2: | 5.7360 | | |

**<u>ML test Control vs. selection</u>**

chi^2(ctrl + sel) - chi^2(ctrl) - chi^2(sel): 42.7156
C chi^2(ctrl + sel) - chi^2(ctrl) - chi^2(sel): **<u>4.8750</u>**

**<u>ML test Ctrl Vs. Selection</u>**

| | | | | | | |
|---|---|---|---|---|---|---|
| ML est [r1,r2,c] | 0.1884 | 0.1449 | 0.8139 | 0.2076 | 0.2120 | 1.1010 |
| ML SE [r1,r2,c] | 0.0082 | 0.0074 | 0.0992 | 0.0085 | 0.0086 | 0.0814 |

**<u>ML test Ctrl vs. Selection (r1 and r2 are line-specific)</u>**

| | | | | | | |
|---|---|---|---|---|---|---|
| c - control or selection specific | | | | | | |
| Line1[r1,r2,c]: | 0.1974 | 0.1307 | 0.8178 | 0.2027 | 0.2267 | 1.1025 |
| Line2[r1,r2,c]: | 0.1946 | 0.1426 | 0.8178 | 0.2148 | 0.1908 | 1.1025 |
| Line3[r1,r2,c]: | 0.1733 | 0.1613 | 0.8178 | 0.2052 | 0.2185 | 1.1025 |
| c-global | | | | | | |
| Line1[r1,r2,c]: | 0.1973 | 0.1307 | 1.0010 | 0.2027 | 0.2267 | 1.0010 |
| Line2[r1,r2,c]: | 0.1947 | 0.1427 | 1.0010 | 0.2147 | 0.1907 | 1.0010 |
| Line3[r1,r2,c]: | 0.1733 | 0.1613 | 1.0010 | 0.2053 | 0.2187 | 1.0010 |

ML ratio (df=14 –13=1): 4.6836

***ML test Ctrl vs. Selection (r1, r2 and c are all line-specific)**

| | | | | | | |
|---|---|---|---|---|---|---|
| Line1[r1,r2,c]: | 0.1973 | 0.1307 | 0.7757 | 0.2027 | 0.2267 | 1.1320 |
| Line2[r1,r2,c]: | 0.1947 | 0.1427 | 0.8642 | 0.2147 | 0.1907 | 1.1727 |
| Line3[r1,r2,c]: | 0.1733 | 0.1613 | 0.8106 | 0.2053 | 0.2187 | 1.0097 |

ML ratio (df=18 –13=5): 5.5364
ML ratio for heterogeneity (control+selection; df = 5–1=4): 5.5364 – 4.6836
……………………………………………………………………………………………………

## markers 1-2-4 (*ru-h-cu*)

| | CONTROL | | | DESICCATION | | |
|---|---|---|---|---|---|---|
| **Lines (ML estimates)** | | | | | | |
| Line1 [r1,r2,c]: | 0.1973 | 0.1867 | 0.5430 | 0.2027 | 0.2560 | 0.9766 |
| Line2 [r1,r2,c]: | 0.1947 | 0.1987 | 0.6895 | 0.2147 | 0.2373 | 1.1253 |
| Line3 [r1,r2,c]: | 0.1733 | 0.2133 | 0.5769 | 0.2053 | 0.2480 | 0.8902 |
| Bailey test for 3 × 750 | | | | | | |
| Teta [r1,r2,c]: | 0.1880 | 0.1991 | 0.5999 | 0.2078 | 0.2470 | 0.9924 |
| SE [r1,r2,c]: | 0.0082 | 0.0084 | 0.0740 | 0.0085 | 0.0091 | 0.0717 |
| Chi2: | | 3.7995 | | | 2.8699 | |
| C Chi2: | | 0.6994 | | | 1.7923 | |

Bailey for 6 × 750:
Teta: 0.1968 0.2211 0.8070
Chi2: 38.8385
C chi2: 17.0368

**ML test Control vs. selection**
chi^2(ctrl + sel) - chi^2(ctrl) - chi^2(sel): 32.1691
C chi^2(ctrl + sel) - chi^2(ctrl) - chi^2(sel): **14.5451**

**ML test Ctrl Vs. Selection**

| | | | | | | |
|---|---|---|---|---|---|---|
| ML est [r1,r2,c] | 0.1884 | 0.1996 | 0.6027 | 0.2076 | 0.2471 | 0.9965 |
| ML SE [r1,r2,c] | 0.0082 | 0.0084 | 0.0741 | 0.0085 | 0.0091 | 0.0718 |

**ML test Ctrl vs. Selection (r1 and r2 are line-specific)**

| | | | | | | |
|---|---|---|---|---|---|---|
| c - control or selection specific | | | | | | |
| Line1[r1,r2,c]: | 0.1976 | 0.1869 | 0.6047 | 0.2027 | 0.2560 | 0.9971 |
| Line2[r1,r2,c]: | 0.1943 | 0.1983 | 0.6047 | 0.2147 | 0.2373 | 0.9971 |
| Line3[r1,r2,c]: | 0.1734 | 0.2134 | 0.6047 | 0.2053 | 0.2480 | 0.9971 |
| c-global | | | | | | |
| Line1[r1,r2,c]: | 0.1978 | 0.1871 | 0.8380 | 0.2023 | 0.2555 | 0.8380 |
| Line2[r1,r2,c]: | 0.1949 | 0.1989 | 0.8380 | 0.2139 | 0.2365 | 0.8380 |

| | | | | | | |
|---|---|---|---|---|---|---|
| Line3[r1,r2,c]: | 0.1737 | 0.2138 | 0.8380 | 0.2052 | 0.2478 | 0.8380 |

ML ratio (df=14 –13=1): 13.6274

**<u>*ML test Ctrl vs. Selection (r1, r2 and c are all line-specific)</u>**

| | | | | | | |
|---|---|---|---|---|---|---|
| Line1[r1,r2,c]: | 0.1973 | 0.1867 | 0.5430 | 0.2027 | 0.2560 | 0.9766 |
| Line2[r1,r2,c]: | 0.1947 | 0.1987 | 0.6895 | 0.2147 | 0.2373 | 1.1253 |
| Line3[r1,r2,c]: | 0.1733 | 0.2133 | 0.5769 | 0.2053 | 0.2480 | 0.8902 |

ML ratio (df=18 –13=5): 16.1433
ML ratio for heterogeneity (control+selection; df = 5–1=4): 16.1433 – 13.6274
……………………………………………………………………………………………………

**<u>markers 1-2-5 (*ru-h-sr*)</u>**

| | CONTROL | | | DESICCATION | | |
|---|---|---|---|---|---|---|
| **<u>Lines (ML estimates)</u>** | | | | | | |
| Line1 [r1,r2,c]: | 0.1973 | 0.2573 | 0.4726 | 0.2027 | 0.2787 | 0.7083 |
| Line2 [r1,r2,c]: | 0.1947 | 0.2773 | 0.6174 | 0.2147 | 0.2720 | 0.9362 |
| Line3 [r1,r2,c]: | 0.1733 | 0.2707 | 0.3979 | 0.2053 | 0.2893 | 0.6284 |
| Bailey test for 3 × 750 | | | | | | |
| Teta [r1,r2,c]: | 0.1879 | 0.2681 | 0.4898 | 0.2080 | 0.2800 | 0.7446 |
| SE [r1,r2,c]: | 0.0082 | 0.0093 | 0.0575 | 0.0085 | 0.0095 | 0.0610 |
| Chi2: | | 4.7077 | | | 5.3564 | |
| C Chi2: | | 2.4457 | | | 4.3971 | |

Bailey for 6 × 750:

| | | | |
|---|---|---|---|
| Teta: | 0.1974 | 0.2738 | 0.6106 |
| Chi2: | 22.6993 | | |
| C chi2: | 16.1428 | | |

**<u>ML test Control vs. selection</u>**
chi^2(ctrl + sel) - chi^2(ctrl) - chi^2(sel): 12.6352
C chi^2(ctrl + sel) - chi^2(ctrl) - chi^2(sel): **<u>9.2999</u>**

**<u>ML test Ctrl Vs. Selection</u>**

| | | | | | | |
|---|---|---|---|---|---|---|
| ML est [r1,r2,c] | 0.1884 | 0.2684 | 0.5008 | 0.2076 | 0.2800 | 0.7571 |
| ML SE [r1,r2,c] | 0.0082 | 0.0093 | 0.0583 | 0.0085 | 0.0095 | 0.0615 |

**<u>ML test Ctrl vs. Selection (r1 and r2 are line-specific)</u>**

c - control or selection specific

| | | | | | | |
|---|---|---|---|---|---|---|
| Line1[r1,r2,c]: | 0.1975 | 0.2576 | 0.5017 | 0.2029 | 0.2789 | 0.7569 |
| Line2[r1,r2,c]: | 0.1939 | 0.2762 | 0.5017 | 0.2138 | 0.2709 | 0.7569 |
| Line3[r1,r2,c]: | 0.1739 | 0.2715 | 0.5017 | 0.2059 | 0.2902 | 0.7569 |

c-global

| | | | | | | |
|---|---|---|---|---|---|---|
| Line1[r1,r2,c]: | 0.1982 | 0.2584 | 0.6396 | 0.2023 | 0.2781 | 0.6396 |
| Line2[r1,r2,c]: | 0.1948 | 0.2775 | 0.6396 | 0.2128 | 0.2696 | 0.6396 |
| Line3[r1,r2,c]: | 0.1743 | 0.2722 | 0.6396 | 0.2054 | 0.2894 | 0.6396 |

ML ratio (df=14 –13=1): 8.8601

***ML test Ctrl vs. Selection (r1, r2 and c are all line-specific)**

| | | | | | | |
|---|---|---|---|---|---|---|
| Line1[r1,r2,c]: | 0.1973 | 0.2573 | 0.4726 | 0.2027 | 0.2787 | 0.7083 |
| Line2[r1,r2,c]: | 0.1947 | 0.2773 | 0.6174 | 0.2147 | 0.2720 | 0.9362 |
| Line3[r1,r2,c]: | 0.2147 | 0.2720 | 0.9362 | 0.2053 | 0.2893 | 0.6284 |

ML ratio (df=18 –13=5): 15.8022

ML ratio for heterogeneity (control+selection; df = 5–1=4): 15.8022 – 8.8601
……………………………………………………………………………………………………………

## markers 1-2-6 (*ru-h-e*)

| | CONTROL | | | DESICCATION | | |
|---|---|---|---|---|---|---|
| **Lines (ML estimates)** | | | | | | |
| Line1 [r1,r2,c]: | 0.1973 | 0.3120 | 0.4115 | 0.2027 | 0.3133 | 0.6089 |
| Line2 [r1,r2,c]: | 0.1947 | 0.3053 | 0.4711 | 0.2147 | 0.3187 | 0.7991 |
| Line3 [r1,r2,c]: | 0.1733 | 0.3187 | 0.3379 | 0.2053 | 0.3280 | 0.5939 |
| Bailey test for 3 × 750 | | | | | | |
| Teta [r1,r2,c]: | 0.1880 | 0.3119 | 0.4028 | 0.2076 | 0.3200 | 0.6624 |
| SE [r1,r2,c]: | 0.0082 | 0.0098 | 0.0491 | 0.0085 | 0.0098 | 0.0541 |
| Chi2: | | 3.0659 | | | 3.6044 | |
| C Chi2: | | 1.2221 | | | 2.8918 | |

Bailey for 6 × 750:

| | | | |
|---|---|---|---|
| Teta: | 0.1973 | 0.3156 | 0.5205 |
| Chi2: | 21.8933 | | |
| C chi2: | 16.7941 | | |

**ML test Control vs. selection**
chi^2(ctrl + sel) - chi^2(ctrl) - chi^2(sel): 15.2230
C chi^2(ctrl + sel) - chi^2(ctrl) - chi^2(sel): **12.6801**

**ML test Ctrl Vs. Selection**

| | | | | | | |
|---|---|---|---|---|---|---|
| ML est [r1,r2,c] | 0.1884 | 0.3120 | 0.4082 | 0.2076 | 0.3200 | 0.6692 |
| ML SE [r1,r2,c] | 0.0082 | 0.0098 | 0.0494 | 0.0085 | 0.0098 | 0.0544 |

**ML test Ctrl vs. Selection (r1 and r2 are line-specific)**

c - control or selection specific

| | | | | | | |
|---|---|---|---|---|---|---|
| Line1[r1,r2,c]: | 0.1973 | 0.3120 | 0.4086 | 0.2030 | 0.3139 | 0.6693 |

| | | | | | | |
|---|---|---|---|---|---|---|
| Line2[r1,r2,c]: | 0.1941 | 0.3045 | 0.4086 | 0.2137 | 0.3173 | 0.6693 |
| Line3[r1,r2,c]: | 0.1738 | 0.3196 | 0.4086 | 0.2059 | 0.3288 | 0.6693 |

c-global

| | | | | | | |
|---|---|---|---|---|---|---|
| Line1[r1,r2,c]: | 0.1984 | 0.3137 | 0.5480 | 0.2022 | 0.3126 | 0.5480 |
| Line2[r1,r2,c]: | 0.1952 | 0.3062 | 0.5480 | 0.2123 | 0.3152 | 0.5480 |
| Line3[r1,r2,c]: | 0.1746 | 0.3210 | 0.5480 | 0.2049 | 0.3274 | 0.5480 |

ML ratio (df=14 –13=1): 12.2332

***ML test Ctrl vs. Selection (r1, r2 and c are all line-specific)**

| | | | | | | |
|---|---|---|---|---|---|---|
| Line1[r1,r2,c]: | 0.1973 | 0.3120 | 0.4115 | 0.2027 | 0.3133 | 0.6089 |
| Line2[r1,r2,c]: | 0.1947 | 0.3053 | 0.4711 | 0.2147 | 0.3187 | 0.7991 |
| Line3[r1,r2,c]: | 0.1733 | 0.3187 | 0.3379 | 0.2053 | 0.3280 | 0.5939 |

ML ratio (df=18 –13=5): 16.3775

ML ratio for heterogeneity (control+selection; df = 5–1=4):
……………………………………………………………………………………………………………

**markers 1-3-4 (*ru-th-cu*)**

| | CONTROL | | | DESICCATION | | |
|---|---|---|---|---|---|---|
| **Lines (ML estimates)** | | | | | | |
| Line1 [r1,r2,c]: | 0.2881 | 0.0560 | 0.0000 | 0.3253 | 0.0480 | 0.5125 |
| Line2 [r1,r2,c]: | 0.2893 | 0.0560 | 0.1646 | 0.3093 | 0.0573 | 0.8273 |
| Line3 [r1,r2,c]: | 0.2893 | 0.0573 | 0.0804 | 0.3333 | 0.0427 | 0.4690 |
| Bailey test for 3 × 750 | | | | | | |
| Teta [r1,r2,c]: | 0.2886 | 0.0564 | 0.0000 | 0.3224 | 0.0488 | 0.5875 |
| SE [r1,r2,c]: | 0.0096 | 0.0049 | 0.0005 | 0.0098 | 0.0045 | 0.1128 |
| Chi2: | | 3.1730 | | | 4.5092 | |
| C Chi2: | | 3.1566 | | | 1.8570 | |

Bailey for 6 × 750:

| | | | |
|---|---|---|---|
| Teta: | 0.3045 | 0.0519 | 0.0000 |
| Chi2: | 41.2541 | | |
| C chi2: | 32.2523 | | |

**ML test Control vs. selection**
chi^2(ctrl + sel) - chi^2(ctrl) - chi^2(sel): 33.5718
C chi^2(ctrl + sel) - chi^2(ctrl) - chi^2(sel): **27.2387**

**ML test Ctrl Vs. Selection**

| | | | | | | |
|---|---|---|---|---|---|---|
| ML est [r1,r2,c] | 0.2889 | 0.0564 | 0.0818 | 0.3227 | 0.0493 | 0.6142 |
| ML SE [r1,r2,c] | 0.0096 | 0.0049 | 0.0465 | 0.0099 | 0.0046 | 0.1152 |

**ML test Ctrl vs. Selection (r1 and r2 are line-specific)**

c - control or selection specific

| | | | | | | |
|---|---|---|---|---|---|---|
| Line1[r1,r2,c]: | 0.2884 | 0.0561 | 0.0818 | 0.3256 | 0.0480 | 0.6160 |
| Line2[r1,r2,c]: | 0.2890 | 0.0559 | 0.0818 | 0.3088 | 0.0572 | 0.6160 |
| Line3[r1,r2,c]: | 0.2893 | 0.0573 | 0.0818 | 0.3337 | 0.0427 | 0.6160 |

c-global

| | | | | | | |
|---|---|---|---|---|---|---|
| Line1[r1,r2,c]: | 0.2892 | 0.0562 | 0.3464 | 0.3247 | 0.0479 | 0.3464 |
| Line2[r1,r2,c]: | 0.2900 | 0.0561 | 0.3464 | 0.3074 | 0.0570 | 0.3464 |
| Line3[r1,r2,c]: | 0.2903 | 0.0575 | 0.3464 | 0.3329 | 0.0426 | 0.3464 |

ML ratio (df=14 –13=1): 18.987

**<u>*ML test Ctrl vs. Selection (r1, r2 and c are all line-specific)</u>**

| | | | | | | |
|---|---|---|---|---|---|---|
| Line1[r1,r2,c]: | 0.2881 | 0.0560 | 0.0000 | 0.3253 | 0.0480 | 0.5125 |
| Line2[r1,r2,c]: | 0.2893 | 0.0560 | 0.1646 | 0.3093 | 0.0573 | 0.8273 |
| Line3[r1,r2,c]: | 0.2893 | 0.0573 | 0.0804 | 0.3333 | 0.0427 | 0.4690 |

ML ratio (df=18 –13=5): 23.7573

ML ratio for heterogeneity (control+selection; df = 5–1=4): 23.7573 – 18.987
…………………………………………………………………………………………………

## <u>markers 1-3-5 (*ru-th-sr*)</u>

| | CONTROL | | | DESICCATION | | |
|---|---|---|---|---|---|---|
| **<u>Lines (ML estimates)</u>** | | | | | | |
| Line1 [r1,r2,c]: | 0.2880 | 0.1320 | 0.1754 | 0.3253 | 0.1213 | 0.5742 |
| Line2 [r1,r2,c]: | 0.2893 | 0.1427 | 0.3230 | 0.3093 | 0.1667 | 0.9569 |
| Line3 [r1,r2,c]: | 0.2893 | 0.1360 | 0.2372 | 0.3333 | 0.1293 | 0.4948 |
| Bailey test for 3 × 750 | | | | | | |
| Teta [r1,r2,c]: | 0.2887 | 0.1367 | 0.2342 | 0.3220 | 0.1376 | 0.6620 |
| SE [r1,r2,c]: | 0.0096 | 0.0072 | 0.0484 | 0.0098 | 0.0072 | 0.0670 |
| Chi2: | | 1.8281 | | | 16.5728 | |
| C Chi2: | | 1.5008 | | | 8.8751 | |

Bailey for 6 × 750:

| | | | |
|---|---|---|---|
| Teta: | 0.3047 | 0.1366 | 0.3816 |
| Chi2: | 50.1033 | | |
| C chi2: | 37.1730 | | |

**<u>ML test Control vs. selection</u>**
chi^2(ctrl + sel) - chi^2(ctrl) - chi^2(sel): 31.7024
C chi^2(ctrl + sel) - chi^2(ctrl) - chi^2(sel): **<u>26.7971</u>**

**<u>ML test Ctrl Vs. Selection</u>**

| | | | | | | |
|---|---|---|---|---|---|---|
| ML est [r1,r2,c] | 0.2889 | 0.1369 | 0.2473 | 0.3227 | 0.1391 | 0.6931 |

| | | |
|---|---|---|
| ML SE [r1,r2,c] | 0.0096 0.0072 0.0497 | 0.0099 0.0073 0.0689 |

**<u>ML test Ctrl vs. Selection (r1 and r2 are line-specific)</u>**

c - control or selection specific

| | | | | | | |
|---|---|---|---|---|---|---|
| Line1[r1,r2,c]: | 0.2887 | 0.1323 | 0.2473 | 0.3260 | 0.1216 | 0.6969 |
| Line2[r1,r2,c]: | 0.2886 | 0.1423 | 0.2473 | 0.3075 | 0.1657 | 0.6969 |
| Line3[r1,r2,c]: | 0.2894 | 0.1360 | 0.2473 | 0.3346 | 0.1298 | 0.6969 |

c-global

| | | | | | | |
|---|---|---|---|---|---|---|
| Line1[r1,r2,c]: | 0.2902 | 0.1330 | 0.4879 | 0.3246 | 0.1211 | 0.4879 |
| Line2[r1,r2,c]: | 0.2906 | 0.1433 | 0.4879 | 0.3044 | 0.1640 | 0.4879 |
| Line3[r1,r2,c]: | 0.2912 | 0.1369 | 0.4879 | 0.3346 | 0.1298 | 0.6969 |

ML ratio (df=14 –13=1): 26.2444

**<u>*ML test Ctrl vs. Selection (r1, r2 and c are all line-specific)</u>**

| | | | | | | |
|---|---|---|---|---|---|---|
| Line1[r1,r2,c]: | 0.2880 | 0.1320 | 0.1754 | 0.3253 | 0.1213 | 0.5742 |
| Line2[r1,r2,c]: | 0.2893 | 0.1427 | 0.3230 | 0.3093 | 0.1667 | 0.9569 |
| Line3[r1,r2,c]: | 0.2893 | 0.1360 | 0.2372 | 0.3333 | 0.1293 | 0.4948 |

ML ratio (df=18 –13=5): 36.7821

ML ratio for heterogeneity (control+selection; df = 5–1=4): 36.7821 – 26.2444
……………………………………………………………………………………………………………

## <u>markers 1-3-6 (*ru-th-e*)</u>

| | CONTROL | DESICCATION |
|---|---|---|
| **<u>Lines (ML estimates)</u>** | | |
| Line1 [r1,r2,c]: | 0.2880 0.1867 0.1488 | 0.3253 0.1720 0.5242 |
| Line2 [r1,r2,c]: | 0.2893 0.1867 0.2962 | 0.3093 0.2213 0.7790 |
| Line3 [r1,r2,c]: | 0.2893 0.1813 0.1525 | 0.3333 0.1947 0.5753 |
| Bailey test for 3 × 750 | | |
| Teta [r1,r2,c]: | 0.2887 0.1847 0.1825 | 0.3222 0.1948 0.6233 |
| SE [r1,r2,c]: | 0.0096 0.0082 0.0369 | 0.0098 0.0083 0.0546 |
| Chi2: | 2.7418 | 10.3750 |
| C Chi2: | 2.6788 | 3.9496 |

Bailey for 6 × 750:

| | |
|---|---|
| Teta: | 0.3039 0.1886 0.3215 |
| Chi2: | 63.1170 |
| C chi2: | 51.6895 |

**<u>ML test Control vs. selection</u>**

chi^2(ctrl + sel) - chi^2(ctrl) - chi^2(sel): 50.0003
C chi^2(ctrl + sel) - chi^2(ctrl) - chi^2(sel): **45.0611**

**ML test Ctrl Vs. Selection**

| | | | | | | |
|---|---|---|---|---|---|---|
| ML est [r1,r2,c] | 0.2889 | 0.1849 | 0.1997 | 0.3227 | 0.1960 | 0.6325 |
| ML SE [r1,r2,c] | 0.0096 | 0.0082 | 0.0387 | 0.0099 | 0.0084 | 0.0551 |

**ML test Ctrl vs. Selection (r1 and r2 are line-specific)**

c - control or selection specific

| | | | | | | |
|---|---|---|---|---|---|---|
| Line1[r1,r2,c]: | 0.2887 | 0.1871 | 0.1997 | 0.3264 | 0.1726 | 0.6346 |
| Line2[r1,r2,c]: | 0.2880 | 0.1858 | 0.1997 | 0.3077 | 0.2202 | 0.6346 |
| Line3[r1,r2,c]: | 0.2900 | 0.1817 | 0.1997 | 0.3340 | 0.1951 | 0.6346 |

c-global

| | | | | | | |
|---|---|---|---|---|---|---|
| Line1[r1,r2,c]: | 0.2912 | 0.1888 | 0.4374 | 0.3242 | 0.1714 | 0.4374 |
| Line2[r1,r2,c]: | 0.2909 | 0.1877 | 0.4374 | 0.3041 | 0.2176 | 0.4374 |
| Line3[r1,r2,c]: | 0.2924 | 0.1833 | 0.4374 | 0.3311 | 0.1934 | 0.4374 |

ML ratio (df=14 –13=1): 38.4561

***ML test Ctrl vs. Selection (r1, r2 and c are all line-specific)**

| | | | | | | |
|---|---|---|---|---|---|---|
| Line1[r1,r2,c]: | 0.2880 | 0.1867 | 0.1488 | 0.3253 | 0.1720 | 0.5242 |
| Line2[r1,r2,c]: | 0.2893 | 0.1867 | 0.2962 | 0.3093 | 0.2213 | 0.7790 |
| Line3[r1,r2,c]: | 0.2893 | 0.1813 | 0.1525 | 0.3333 | 0.1947 | 0.5753 |

ML ratio (df=18 –13=5): 45.489

ML ratio for heterogeneity (control+selection; df = 5–1=4): 45.489 5– 38.4561
……………………………………………………………………………………………………

**markers 2-3-4 (*h-th-cu*)**

| | CONTROL | | | DESICCATION | | |
|---|---|---|---|---|---|---|
| **Lines (ML estimates)** | | | | | | |
| Line1 [r1,r2,c]: | 0.1307 | 0.0560 | 0.0000 | 0.2266 | 0.0480 | 0.8586 |
| Line2 [r1,r2,c]: | 0.1427 | 0.0560 | 0.0000 | 0.1907 | 0.0573 | 0.4880 |
| Line3 [r1,r2,c]: | 0.1613 | 0.0573 | 0.2885 | 0.2186 | 0.0426 | 0.7152 |
| Bailey test for 3 × 750 | | | | | | |
| Teta [r1,r2,c]: | 0.1439 | 0.0564 | 0.0000 | 0.2113 | 0.0485 | 0.6568 |
| SE [r1,r2,c]: | 0.0074 | 0.0049 | 0.0003 | 0.0086 | 0.0045 | 0.1518 |
| Chi2: | | 4.9883 | | | 5.8811 | |
| C Chi2: | | 2.1394 | | | 1.0876 | |

Bailey for 6 × 750:
Teta: 0.1725 0.0517 0.0000

| | |
|---|---|
| Chi2: | 65.1207 |
| C chi2: | 21.9106 |

**ML test Control vs. selection**

chi^2(ctrl + sel) - chi^2(ctrl) - chi^2(sel): 54.2512

C chi^2(ctrl + sel) - chi^2(ctrl) - chi^2(sel): **18.6836**

**ML test Ctrl Vs. Selection**

| | | | | | | |
|---|---|---|---|---|---|---|
| ML est [r1,r2,c] | 0.1449 | 0.0564 | 0.1087 | 0.2120 | 0.0493 | 0.6797 |
| ML SE [r1,r2,c] | 0.0074 | 0.0049 | 0.0760 | 0.0086 | 0.0046 | 0.1544 |

**ML test Ctrl vs. Selection (r1 and r2 are line-specific)**

c - control or selection specific

| | | | | | | |
|---|---|---|---|---|---|---|
| Line1[r1,r2,c]: | 0.1308 | 0.0560 | 0.1086 | 0.2265 | 0.0480 | 0.6867 |
| Line2[r1,r2,c]: | 0.1428 | 0.0560 | 0.1086 | 0.1908 | 0.0574 | 0.6867 |
| Line3[r1,r2,c]: | 0.1611 | 0.0572 | 0.1086 | 0.2186 | 0.0427 | 0.6867 |

c-global

| | | | | | | |
|---|---|---|---|---|---|---|
| Line1[r1,r2,c]: | 0.1309 | 0.0561 | 0.4357 | 0.2260 | 0.0479 | 0.4357 |
| Line2[r1,r2,c]: | 0.1430 | 0.0561 | 0.4357 | 0.1906 | 0.0573 | 0.4357 |
| Line3[r1,r2,c]: | 0.1615 | 0.0574 | 0.4357 | 0.2183 | 0.0426 | 0.4357 |

ML ratio (df=14 –13=1): 10.2009

***ML test Ctrl vs. Selection (r1, r2 and c are all line-specific)**

| | | | | | | |
|---|---|---|---|---|---|---|
| Line1[r1,r2,c]: | 0.1307 | 0.0560 | 0.0000 | 0.2266 | 0.0480 | 0.8586 |
| Line2[r1,r2,c]: | 0.1427 | 0.0560 | 0.0000 | 0.1907 | 0.0573 | 0.4880 |
| Line3[r1,r2,c]: | 0.1613 | 0.0573 | 0.2885 | 0.2186 | 0.0426 | 0.7152 |

ML ratio (df=18 –13=5): 15.2177

ML ratio for heterogeneity (control+selection; df = 5–1=4): 15.2177 – 10.2009

……………………………………………………………………………………………………………

## markers 2-3-5 (*h-th-sr*)

| | CONTROL | | | DESICCATION | | |
|---|---|---|---|---|---|---|
| **Lines (ML estimates)** | | | | | | |
| Line1 [r1,r2,c]: | 0.1307 | 0.1320 | 0.1546 | 0.2267 | 0.1213 | 1.2605 |
| Line2 [r1,r2,c]: | 0.1427 | 0.1427 | 0.1965 | 0.1907 | 0.1667 | 1.3427 |
| Line3 [r1,r2,c]: | 0.1613 | 0.1360 | 0.6077 | 0.2187 | 0.1293 | 1.0372 |
| Bailey test for 3 × 750 | | | | | | |
| Teta [r1,r2,c]: | 0.1439 | 0.1364 | 0.2453 | 0.2108 | 0.1372 | 1.2126 |
| SE [r1,r2,c]: | 0.0074 | 0.0072 | 0.0702 | 0.0086 | 0.0072 | 0.1071 |
| Chi2: | | 8.4169 | | | 12.3840 | |
| C Chi2: | | 5.2448 | | | 1.5081 | |

Bailey for 6 × 750:

| | |
|---|---|
| Teta: | 0.1732 0.1353 0.5441 |
| Chi2: | 113.6660 |
| C chi2: | 63.6696 |

**ML test Control vs. selection**

chi^2(ctrl + sel) - chi^2(ctrl) - chi^2(sel): 92.8651
C chi^2(ctrl + sel) - chi^2(ctrl) - chi^2(sel): **56.9167**

**ML test Ctrl Vs. Selection**

| | | |
|---|---|---|
| ML est [r1,r2,c] | 0.1449 0.1369 0.3361 | 0.2120 0.1391 1.2056 |
| ML SE [r1,r2,c] | 0.0074 0.0072 0.0825 | 0.0086 0.0073 0.1067 |

**ML test Ctrl vs. Selection (r1 and r2 are line-specific)**

c - control or selection specific

| | | | | | | |
|---|---|---|---|---|---|---|
| Line1[r1,r2,c]: | 0.1310 | 0.1323 | 0.3367 | 0.2268 | 0.1214 | 1.2186 |
| Line2[r1,r2,c]: | 0.1430 | 0.1430 | 0.3367 | 0.1909 | 0.1669 | 1.2186 |
| Line3[r1,r2,c]: | 0.1606 | 0.1354 | 0.3367 | 0.2183 | 0.1291 | 1.2186 |

c-global

| | | | | | | |
|---|---|---|---|---|---|---|
| Line1[r1,r2,c]: | 0.1309 | 0.1323 | 0.8771 | 0.2263 | 0.1211 | 0.8771 |
| Line2[r1,r2,c]: | 0.1430 | 0.1430 | 0.8771 | 0.1902 | 0.1662 | 0.8771 |
| Line3[r1,r2,c]: | 0.1615 | 0.1361 | 0.8771 | 0.2185 | 0.1292 | 0.8771 |

ML ratio (df=14 –13=1): 35.2670

***ML test Ctrl vs. Selection (r1, r2 and c are all line-specific)**

| | | | | | | |
|---|---|---|---|---|---|---|
| Line1[r1,r2,c]: | 0.1307 | 0.1320 | 0.1546 | 0.2267 | 0.1213 | 1.2605 |
| Line2[r1,r2,c]: | 0.1427 | 0.1427 | 0.1965 | 0.1907 | 0.1667 | 1.3427 |
| Line3[r1,r2,c]: | 0.1613 | 0.1360 | 0.6077 | 0.2187 | 0.1293 | 1.0372 |

ML ratio (df=18 –13=5): 42.9086

ML ratio for heterogeneity (control+selection; df = 5–1=4): 42.9086 – 35.2670

………………………………………………………………………………………………

## markers 2-3-6 (*h-th-e*)

| | CONTROL | DESICCATION |
|---|---|---|
| **Lines (ML estimates)** | | |
| Line1 [r1,r2,c]: | 0.1307 0.1867 0.1093 | 0.2267 0.1720 1.0944 |
| Line2 [r1,r2,c]: | 0.1427 0.1867 0.4506 | 0.1907 0.2213 1.1058 |
| Line3 [r1,r2,c]: | 0.1613 0.1813 0.4102 | 0.2187 0.1947 1.0023 |

Bailey test for 3 × 750

| | | |
|---|---|---|
| Teta [r1,r2,c]: | 0.1438 0.1845 0.2361 | 0.2111 0.1945 1.0655 |
| SE [r1,r2,c]: | 0.0074 0.0082 0.0589 | 0.0086 0.0083 0.0840 |
| Chi2: | 9.7921 | 9.6569 |
| C Chi2: | 7.0921 | 0.3159 |

Bailey for 6 × 750:

| | |
|---|---|
| Teta: | 0.1726 0.1877 0.5153 |
| Chi2: | 121.0554 |
| C chi2: | 72.7857 |

**ML test Control vs. selection**

chi^2(ctrl + sel) - chi^2(ctrl) - chi^2(sel): 101.6065

C chi^2(ctrl + sel) - chi^2(ctrl) - chi^2(sel): **65.3777**

**ML test Ctrl Vs. Selection**

| | | |
|---|---|---|
| ML est [r1,r2,c] | 0.1449 0.1849 0.3318 | 0.2120 0.1960 1.0590 |
| ML SE [r1,r2,c] | 0.0074 0.0082 0.0699 | 0.0086 0.0084 0.0836 |

**ML test Ctrl vs. Selection (r1 and r2 are line-specific)**

c - control or selection specific

| | | | | | | |
|---|---|---|---|---|---|---|
| Line1[r1,r2,c]: | 0.1312 | 0.1874 | 0.3326 | 0.2267 | 0.1720 | 1.0665 |
| Line2[r1,r2,c]: | 0.1423 | 0.1862 | 0.3326 | 0.1907 | 0.2214 | 1.0665 |
| Line3[r1,r2,c]: | 0.1611 | 0.1810 | 0.3326 | 0.2186 | 0.1946 | 1.0665 |

c-global

| | | | | | | |
|---|---|---|---|---|---|---|
| Line1[r1,r2,c]: | 0.1313 | 0.1875 | 0.7908 | 0.2259 | 0.1714 | 0.7908 |
| Line2[r1,r2,c]: | 0.1430 | 0.1871 | 0.7908 | 0.1899 | 0.2204 | 0.7908 |
| Line3[r1,r2,c]: | 0.1618 | 0.1819 | 0.7908 | 0.2181 | 0.1941 | 0.7908 |

ML ratio (df=14 –13=1): 37.6210

***ML test Ctrl vs. Selection (r1, r2 and c are all line-specific)**

| | | | | | | |
|---|---|---|---|---|---|---|
| Line1[r1,r2,c]: | 0.1307 | 0.1867 | 0.1093 | 0.2267 | 0.1720 | 1.0944 |
| Line2[r1,r2,c]: | 0.1427 | 0.1867 | 0.4506 | 0.1907 | 0.2213 | 1.1058 |
| Line3[r1,r2,c]: | 0.1613 | 0.1813 | 0.4102 | 0.2187 | 0.1947 | 1.0023 |

ML ratio (df=18 –13=5): 43.2619

ML ratio for heterogeneity (control+selection; df = 5–1=4): 43.2619 5– 37.6210

………………………………………………………………………………………………………

## markers 1-4-5 (*ru-cu-sr*)

| | CONTROL | DESICCATION |
|---|---|---|
| **Lines (ML estimates)** | | |
| Line1 [r1,r2,c]: | 0.3440 0.0760 0.2550 | 0.3573 0.0787 0.6166 |
| Line2 [r1,r2,c]: | 0.3400 0.0867 0.3620 | 0.3373 0.1173 0.9769 |

| | | |
|---|---|---|
| Line3 [r1,r2,c]: | 0.3440 0.0840 0.3691 | 0.3627 0.0867 0.4666 |

Bailey test for 3 × 750

| | | |
|---|---|---|
| Teta [r1,r2,c]: | 0.3426 0.0820 0.3237 | 0.3520 0.0923 0.6707 |
| SE [r1,r2,c]: | 0.0100 0.0058 0.0661 | 0.0101 0.0061 0.0786 |
| Chi2: | 1.2835 | 15.4601 |
| C Chi2: | 0.6606 | 7.6188 |

Bailey for 6 × 750:

| | |
|---|---|
| Teta: | 0.3470 0.0868 0.4681 |
| Chi2: | 29.9530 |
| C chi2: | 19.7423 |

**<u>ML test Control vs. selection</u>**

chi^2(ctrl + sel) - chi^2(ctrl) - chi^2(sel): 13.2094
C chi^2(ctrl + sel) - chi^2(ctrl) - chi^2(sel): **<u>11.4629</u>**

**<u>ML test Ctrl Vs. Selection</u>**

| | | |
|---|---|---|
| ML est [r1,r2,c] | 0.3427 0.0822 0.3313 | 0.3524 0.0942 0.7093 |
| ML SE [r1,r2,c] | 0.0100 0.0058 0.0669 | 0.0101 0.0062 0.0811 |

**<u>ML test Ctrl vs. Selection (r1 and r2 are line-specific)</u>**

c - control or selection specific

| | | | | | | |
|---|---|---|---|---|---|---|
| Line1[r1,r2,c]: | 0.3445 | 0.0761 | 0.3314 | 0.3577 | 0.0787 | 0.7132 |
| Line2[r1,r2,c]: | 0.3398 | 0.0866 | 0.3314 | 0.3359 | 0.1168 | 0.7132 |
| Line3[r1,r2,c]: | 0.3437 | 0.0839 | 0.3314 | 0.3638 | 0.0869 | 0.7132 |

c-global

| | | | | | | |
|---|---|---|---|---|---|---|
| Line1[r1,r2,c]: | 0.3455 | 0.0763 | 0.5386 | 0.3569 | 0.0786 | 0.5386 |
| Line2[r1,r2,c]: | 0.3411 | 0.0869 | 0.5386 | 0.3338 | 0.1161 | 0.5386 |
| Line3[r1,r2,c]: | 0.3450 | 0.0842 | 0.5386 | 0.3632 | 0.0868 | 0.5386 |

ML ratio (df=14 –13=1): 12.4849

**<u>*ML test Ctrl vs. Selection (r1, r2 and c are all line-specific)</u>**

| | | | | | | |
|---|---|---|---|---|---|---|
| Line1[r1,r2,c]: | 0.3440 | 0.0760 | 0.2550 | 0.3573 | 0.0787 | 0.6166 |
| Line2[r1,r2,c]: | 0.3400 | 0.0867 | 0.3620 | 0.3373 | 0.1173 | 0.9769 |
| Line3[r1,r2,c]: | 0.3440 | 0.0840 | 0.3691 | 0.3627 | 0.0867 | 0.4666 |

ML ratio (df=18 –13=5): 20.610

ML ratio for heterogeneity (control+selection; df = 5–1=4): 20.610 – 12.4849

……………………………………………………………………………………………………

**<u>markers 1-4-6 (*ru-cu-e*)</u>**

| | CONTROL | DESICCATION |
|---|---|---|

**Lines (ML estimates)**

| | | |
|---|---|---|
| Line1 [r1,r2,c]: | 0.3440 0.1307 0.1780 | 0.3573 0.1293 0.5193 |
| Line2 [r1,r2,c]: | 0.3400 0.1307 0.3001 | 0.3373 0.1747 0.7468 |
| Line3 [r1,r2,c]: | 0.3440 0.1320 0.2349 | 0.3627 0.1520 0.5563 |

Bailey test for 3 × 750

| | | |
|---|---|---|
| Teta [r1,r2,c]: | 0.3426 0.1311 0.2282 | 0.3521 0.1504 0.6057 |
| SE [r1,r2,c]: | 0.0100 0.0071 0.0446 | 0.0101 0.0075 0.0592 |
| Chi2: | 1.2854 | 9.6330 |
| C Chi2: | 1.2118 | 2.7724 |

Bailey for 6 × 750:

| | |
|---|---|
| Teta: | 0.3464 0.1399 0.3653 |
| Chi2: | 40.1715 |
| C chi2: | 30.1535 |

**ML test Control vs. selection**

chi^2(ctrl + sel) - chi^2(ctrl) - chi^2(sel): 29.2531
C chi^2(ctrl + sel) - chi^2(ctrl) - chi^2(sel): **26.1693**

**ML test Ctrl Vs. Selection**

| | | |
|---|---|---|
| ML est [r1,r2,c] | 0.3427 0.1311 0.2374 | 0.3524 0.1520 0.6139 |
| ML SE [r1,r2,c] | 0.0100 0.0071 0.0455 | 0.0101 0.0076 0.0596 |

**ML test Ctrl vs. Selection (r1 and r2 are line-specific)**

c - control or selection specific

| | | | | | | |
|---|---|---|---|---|---|---|
| Line1[r1,r2,c]: | 0.3448 | 0.1310 | 0.2374 | 0.3582 | 0.1296 | 0.6160 |
| Line2[r1,r2,c]: | 0.3392 | 0.1303 | 0.2374 | 0.3359 | 0.1739 | 0.6160 |
| Line3[r1,r2,c]: | 0.3440 | 0.1320 | 0.2374 | 0.3633 | 0.1523 | 0.6160 |

c-global

| | | | | | | |
|---|---|---|---|---|---|---|
| Line1[r1,r2,c]: | 0.3469 | 0.1318 | 0.4444 | 0.3564 | 0.1290 | 0.4444 |
| Line2[r1,r2,c]: | 0.3415 | 0.1313 | 0.4444 | 0.3331 | 0.1725 | 0.4444 |
| Line3[r1,r2,c]: | 0.3463 | 0.1329 | 0.4444 | 0.3610 | 0.1513 | 0.4444 |

ML ratio (df=14 –13=1): 23.8279

***ML test Ctrl vs. Selection (r1, r2 and c are all line-specific)**

| | | | | | | |
|---|---|---|---|---|---|---|
| Line1[r1,r2,c]: | 0.3440 | 0.1307 | 0.1780 | 0.3573 | 0.1293 | 0.5193 |
| Line2[r1,r2,c]: | 0.3400 | 0.1307 | 0.3001 | 0.3373 | 0.1747 | 0.7468 |
| Line3[r1,r2,c]: | 0.3440 | 0.1320 | 0.2349 | 0.3627 | 0.1520 | 0.5563 |

ML ratio (df=18 –13=5): 27.8520

ML ratio for heterogeneity (control+selection; df = 5–1=4): 27.8520 – 23.8279

**------------------------------------------------------------------------------------------------------------------------**

**markers 2-4-5 (*h-cu-sr*)**

| | CONTROL | | | DESICCATION | | |
|---|---|---|---|---|---|---|
| <u>**Lines (ML estimates)**</u> | | | | | | |
| Line1 [r1,r2,c]: | 0.1867 | 0.0760 | 0.1880 | 0.2560 | 0.0787 | 1.3907 |
| Line2 [r1,r2,c]: | 0.1987 | 0.0867 | 0.2323 | 0.2373 | 0.1173 | 1.4843 |
| Line3 [r1,r2,c]: | 0.2133 | 0.0840 | 0.7443 | 0.2480 | 0.0867 | 1.0546 |
| Bailey test for 3 × 750 | | | | | | |
| Teta [r1,r2,c]: | 0.1990 | 0.0818 | 0.2968 | 0.2467 | 0.0920 | 1.3156 |
| SE [r1,r2,c]: | 0.0084 | 0.0058 | 0.0838 | 0.0091 | 0.0061 | 0.1224 |
| Chi2: | | 7.8905 | | | 10.3607 | |
| C Chi2: | | 5.5622 | | | 2.3821 | |

Bailey for 6 × 750:

| | | | |
|---|---|---|---|
| Teta: | 0.2218 | 0.0860 | 0.6320 |
| Chi2: | 83.0018 | | |
| C chi2: | 54.9405 | | |

<u>**ML test Control vs. selection**</u>

chi^2(ctrl + sel) - chi^2(ctrl) - chi^2(sel): 64.7505

C chi^2(ctrl + sel) - chi^2(ctrl) - chi^2(sel): <u>**46.9962**</u>

<u>**ML test Ctrl Vs. Selection**</u>

| | | | | | | |
|---|---|---|---|---|---|---|
| ML est [r1,r2,c] | 0.1996 | 0.0822 | 0.4064 | 0.2471 | 0.0942 | 1.3170 |
| ML SE [r1,r2,c] | 0.0084 | 0.0058 | 0.0987 | 0.0091 | 0.0062 | 0.1229 |

<u>**ML test Ctrl vs. Selection (r1 and r2 are line-specific)**</u>

c - control or selection specific

| | | | | | | |
|---|---|---|---|---|---|---|
| Line1[r1,r2,c]: | 0.1870 | 0.0762 | 0.4067 | 0.2562 | 0.0787 | 1.3258 |
| Line2[r1,r2,c]: | 0.1991 | 0.0868 | 0.4067 | 0.2379 | 0.1176 | 1.3258 |
| Line3[r1,r2,c]: | 0.2125 | 0.0837 | 0.4067 | 0.2472 | 0.0864 | 1.3258 |

c-global

| | | | | | | |
|---|---|---|---|---|---|---|
| Line1[r1,r2,c]: | 0.1868 | 0.0760 | 0.9605 | 0.2559 | 0.0786 | 0.9605 |
| Line2[r1,r2,c]: | 0.1988 | 0.0867 | 0.9605 | 0.2372 | 0.1172 | 0.9605 |
| Line3[r1,r2,c]: | 0.2134 | 0.0840 | 0.9605 | 0.2480 | 0.0867 | 0.9605 |

ML ratio (df=14 –13=1): 28.7860

<u>***ML test Ctrl vs. Selection (r1, r2 and c are all line-specific)**</u>

| | | | | | | |
|---|---|---|---|---|---|---|
| Line1[r1,r2,c]: | 0.1867 | 0.0760 | 0.1880 | 0.2560 | 0.0787 | 1.3907 |
| Line2[r1,r2,c]: | 0.1987 | 0.0867 | 0.2323 | 0.2373 | 0.1173 | 1.4843 |
| Line3[r1,r2,c]: | 0.2133 | 0.0840 | 0.7443 | 0.2480 | 0.0867 | 1.0546 |

ML ratio (df=18 –13=5): 37.5947

ML ratio for heterogeneity (control+selection; df = 5–1=4): 37.5947– 28.7860
……………………………………………………………………………………………………

**<u>markers 2-4-6 (*h-cu-e*)</u>**

| | CONTROL | | | DESICCATION | | |
|---|---|---|---|---|---|---|
| **<u>Lines (ML estimates)</u>** | | | | | | |
| Line1 [r1,r2,c]: | 0.1867 | 0.1307 | 0.1093 | 0.2560 | 0.1293 | 1.0873 |
| Line2 [r1,r2,c]: | 0.1987 | 0.1307 | 0.4623 | 0.2373 | 0.1747 | 1.1257 |
| Line3 [r1,r2,c]: | 0.2133 | 0.1320 | 0.4735 | 0.2480 | 0.1520 | 0.9550 |
| Bailey test for 3 × 750 | | | | | | |
| Teta [r1,r2,c]: | 0.1988 | 0.1307 | 0.2430 | 0.2469 | 0.1502 | 1.0549 |
| SE [r1,r2,c]: | 0.0084 | 0.0071 | 0.0601 | 0.0091 | 0.0075 | 0.0882 |
| Chi2: | | 9.8694 | | | 7.5973 | |
| C Chi2: | | 8.3430 | | | 0.7257 | |

Bailey for 6 × 750:

| | | | |
|---|---|---|---|
| Teta: | 0.2208 | 0.1390 | 0.5057 |
| Chi2: | 94.3612 | | |
| C chi2: | 66.9137 | | |

**<u>ML test Control vs. selection</u>**
chi^2(ctrl + sel) - chi^2(ctrl) - chi^2(sel): 76.8944
C chi^2(ctrl + sel) - chi^2(ctrl) - chi^2(sel): **<u>57.8450</u>**

**<u>ML test Ctrl Vs. Selection</u>**

| | | | | | | |
|---|---|---|---|---|---|---|
| ML est [r1,r2,c] | 0.1996 | 0.1311 | 0.3567 | 0.2471 | 0.1520 | 1.0531 |
| ML SE [r1,r2,c] | 0.0084 | 0.0071 | 0.0730 | 0.0091 | 0.0076 | 0.0881 |

**<u>ML test Ctrl vs. Selection (r1 and r2 are line-specific)</u>**

| | | | | | | |
|---|---|---|---|---|---|---|
| c - control or selection specific | | | | | | |
| Line1[r1,r2,c]: | 0.1875 | 0.1312 | 0.3572 | 0.2560 | 0.1293 | 1.0572 |
| Line2[r1,r2,c]: | 0.1983 | 0.1304 | 0.3572 | 0.2374 | 0.1747 | 1.0572 |
| Line3[r1,r2,c]: | 0.2128 | 0.1317 | 0.3572 | 0.2479 | 0.1519 | 1.0572 |
| c-global | | | | | | |
| Line1[r1,r2,c]: | 0.1875 | 0.1313 | 0.7804 | 0.2552 | 0.1289 | 0.7804 |
| Line2[r1,r2,c]: | 0.1991 | 0.1310 | 0.7804 | 0.2363 | 0.1739 | 0.7804 |
| Line3[r1,r2,c]: | 0.2139 | 0.1323 | 0.7804 | 0.2475 | 0.1517 | 0.7804 |

ML ratio (df=14 –13=1): 32.2257

**<u>*ML test Ctrl vs. Selection (r1, r2 and c are all line-specific)</u>**

| | | | | | | |
|---|---|---|---|---|---|---|
| Line1[r1,r2,c]: | 0.1867 | 0.1307 | 0.1093 | 0.2560 | 0.1293 | 1.0873 |

| | | | | | | |
|---|---|---|---|---|---|---|
| Line2[r1,r2,c]: | 0.1987 | 0.1307 | 0.4623 | 0.2373 | 0.1747 | 1.1257 |
| Line3[r1,r2,c]: | 0.2133 | 0.1320 | 0.4735 | 0.2480 | 0.1520 | 0.9550 |

ML ratio (df=18 –13=5): 39.1532

ML ratio for heterogeneity (control+selection; df = 5–1=4): 39.1532 – 32.2257

…………………………………………………………………………………………………

## markers 3-4-5 (*th-cu-sr*)

| | CONTROL | | | DESICCATION | | |
|---|---|---|---|---|---|---|
| **Lines (ML estimates)** | | | | | | |
| Line1 [r1,r2,c]: | 0.0560 | 0.0760 | 0.0000 | 0.0480 | 0.0786 | 0.7059 |
| Line2 [r1,r2,c]: | 0.0560 | 0.0867 | 0.0000 | 0.0573 | 0.1173 | 0.5956 |
| Line3 [r1,r2,c]: | 0.0573 | 0.0840 | 0.5556 | 0.0427 | 0.0867 | 0.0000 |
| Bailey test for 3 × 750 | | | | | | |
| Teta [r1,r2,c]: | 0.0564 | 0.0819 | 0.0000 | 0.0486 | 0.0916 | 0.0000 |
| SE [r1,r2,c]: | 0.0049 | 0.0058 | 0.0005 | 0.0045 | 0.0061 | 0.0006 |
| Chi2: | | 2.8226 | | | 14.1495 | |
| C Chi2: | | 2.1771 | | | 5.5417 | |

Bailey for 6 × 750:

| | | | |
|---|---|---|---|
| Teta: | 0.0522 | 0.0866 | 0.0000 |
| Chi2: | 19.5524 | | |
| C chi2: | 7.7188 | | |

**ML test Control vs. selection**

chi^2(ctrl + sel) - chi^2(ctrl) - chi^2(sel): 2.5803

C chi^2(ctrl + sel) - chi^2(ctrl) - chi^2(sel): **0.0000**

**ML test Ctrl Vs. Selection**

| | | | | | | |
|---|---|---|---|---|---|---|
| ML est [r1,r2,c] | 0.0564 | 0.0822 | 0.1915 | 0.0493 | 0.0942 | 0.4781 |
| ML SE [r1,r2,c] | 0.0049 | 0.0058 | 0.1336 | 0.0046 | 0.0062 | 0.2063 |

**ML test Ctrl vs. Selection (r1 and r2 are line-specific)**

c - control or selection specific

| | | | | | | |
|---|---|---|---|---|---|---|
| Line1[r1,r2,c]: | 0.0560 | 0.0761 | 0.1932 | 0.0480 | 0.0786 | 0.4710 |
| Line2[r1,r2,c]: | 0.0560 | 0.0867 | 0.1932 | 0.0573 | 0.1173 | 0.4710 |
| Line3[r1,r2,c]: | 0.0573 | 0.0839 | 0.1932 | 0.0427 | 0.0868 | 0.4710 |

c-global

| | | | | | | |
|---|---|---|---|---|---|---|
| Line1[r1,r2,c]: | 0.0561 | 0.0761 | 0.3334 | 0.0479 | 0.0786 | 0.3334 |
| Line2[r1,r2,c]: | 0.0561 | 0.0868 | 0.3334 | 0.0573 | 0.1172 | 0.3334 |
| Line3[r1,r2,c]: | 0.0573 | 0.0839 | 0.3334 | 0.0427 | 0.0867 | 0.3334 |

ML ratio (df=14 –13=1): 1.3397

***ML test Ctrl vs. Selection (r1, r2 and c are all line-specific)**

| | | | | | | |
|---|---|---|---|---|---|---|
| Line1[r1,r2,c]: | 0.0560 | 0.0760 | 0.0000 | 0.0480 | 0.0786 | 0.7059 |
| Line2[r1,r2,c]: | 0.0560 | 0.0867 | 0.0000 | 0.0573 | 0.1173 | 0.5956 |
| Line3[r1,r2,c]: | 0.0573 | 0.0840 | 0.5556 | 0.0427 | 0.0867 | 0.0000 |

ML ratio (df=18 –13=5): 8.8652

ML ratio for heterogeneity (control+selection; df = 5–1=4): 8.8652 – 1.3397
……………………………………………………………………………………………………

**markers 3-4-6 (*th-cu-e*)**

| | CONTROL | | | DESICCATION | | |
|---|---|---|---|---|---|---|
| **Lines (ML estimates)** | | | | | | |
| Line1 [r1,r2,c]: | 0.0560 | 0.1307 | 0.0000 | 0.0480 | 0.1293 | 0.4303 |
| Line2 [r1,r2,c]: | 0.0560 | 0.1307 | 0.0000 | 0.0573 | 0.1747 | 0.5331 |
| Line3 [r1,r2,c]: | 0.0573 | 0.1320 | 0.5290 | 0.0427 | 0.1520 | 0.0000 |
| Bailey test for 3 × 750 | | | | | | |
| Teta [r1,r2,c]: | 0.0564 | 0.1310 | 0.0000 | 0.0486 | 0.1498 | 0.0000 |
| SE [r1,r2,c]: | 0.0049 | 0.0071 | 0.0005 | 0.0045 | 0.0075 | 0.0002 |
| Chi2: | | 3.3539 | | | 14.5849 | |
| C Chi2: | | 3.3353 | | | 6.7371 | |

Bailey for 6 × 750:

| | | | |
|---|---|---|---|
| Teta: | 0.0522 | 0.1399 | 0.0000 |
| Chi2: | 22.3281 | | |
| C chi2: | 10.0725 | | |

**ML test Control vs. selection**

chi^2(ctrl + sel) - chi^2(ctrl) - chi^2(sel): 4.3892

C chi^2(ctrl + sel) - chi^2(ctrl) - chi^2(sel): **0.0000**

**ML test Ctrl Vs. Selection**

| | | | | | | |
|---|---|---|---|---|---|---|
| ML est [r1,r2,c] | 0.0564 | 0.1311 | 0.1801 | 0.0493 | 0.1520 | 0.3556 |
| ML SE [r1,r2,c] | 0.0049 | 0.0071 | 0.1022 | 0.0046 | 0.0076 | 0.1398 |

**ML test Ctrl vs. Selection (r1 and r2 are line-specific)**

c - control or selection specific

| | | | | | | |
|---|---|---|---|---|---|---|
| Line1[r1,r2,c]: | 0.0561 | 0.1308 | 0.1804 | 0.0480 | 0.1293 | 0.3533 |
| Line2[r1,r2,c]: | 0.0561 | 0.1308 | 0.1804 | 0.0573 | 0.1744 | 0.3533 |
| Line3[r1,r2,c]: | 0.0572 | 0.1317 | 0.1804 | 0.0427 | 0.1522 | 0.3533 |

c-global

| | | | | | | |
|---|---|---|---|---|---|---|
| Line1[r1,r2,c]: | 0.0561 | 0.1309 | 0.2679 | 0.0480 | 0.1292 | 0.2679 |

| | | | | | | |
|---|---|---|---|---|---|---|
| Line2[r1,r2,c]: | 0.0561 | 0.1309 | 0.2679 | 0.0572 | 0.1743 | 0.2679 |
| Line3[r1,r2,c]: | 0.0572 | 0.1318 | 0.2679 | 0.0427 | 0.1522 | 0.2679 |

ML ratio (df=14 –13=1): 1.0140

**<u>*ML test Ctrl vs. Selection (r1, r2 and c are all line-specific)</u>**

| | | | | | | |
|---|---|---|---|---|---|---|
| Line1[r1,r2,c]: | 0.0560 | 0.1307 | 0.0000 | 0.0480 | 0.1293 | 0.4303 |
| Line2[r1,r2,c]: | 0.0560 | 0.1307 | 0.0000 | 0.0573 | 0.1747 | 0.5331 |
| Line3[r1,r2,c]: | 0.0573 | 0.1320 | 0.5290 | 0.0427 | 0.1520 | 0.0000 |

ML ratio (df=18 –13=5): 11.979

ML ratio for heterogeneity (control+selection; df = 5–1=4): 11.979 – 1.0140
…………………………………………………………………………………………………

**<u>markers 1-5-6 (*ru-sr-e*)</u>**

| | CONTROL | | | DESICCATION | | |
|---|---|---|---|---|---|---|
| **<u>Lines (ML estimates)</u>** | | | | | | |
| Line1 [r1,r2,c]: | 0.4067 | 0.0547 | 0.0600 | 0.4013 | 0.0533 | 0.3737 |
| Line2 [r1,r2,c]: | 0.4053 | 0.0440 | 0.1495 | 0.3773 | 0.0600 | 0.2944 |
| Line3 [r1,r2,c]: | 0.4067 | 0.0507 | 0.0647 | 0.4200 | 0.0680 | 0.6069 |
| Bailey test for 3 × 750 | | | | | | |
| Teta [r1,r2,c]: | 0.4064 | 0.0494 | 0.0754 | 0.3991 | 0.0597 | 0.4116 |
| SE [r1,r2,c]: | 0.0104 | 0.0046 | 0.0399 | 0.0103 | 0.0050 | 0.0771 |
| Chi2: | | 1.6482 | | | 7.0589 | |
| C Chi2: | | 0.6251 | | | 2.8983 | |

Bailey for 6 × 750:

| | | | |
|---|---|---|---|
| Teta: | 0.4019 | 0.0541 | 0.1463 |
| Chi2: | 26.0772 | | |
| C chi2: | 18.5481 | | |

**<u>ML test Control vs. selection</u>**
chi^2(ctrl + sel) - chi^2(ctrl) - chi^2(sel): 17.3701
C chi^2(ctrl + sel) - chi^2(ctrl) - chi^2(sel): **<u>15.0247</u>**

**<u>ML test Ctrl Vs. Selection</u>**

| | | | | | | |
|---|---|---|---|---|---|---|
| ML est [r1,r2,c] | 0.4062 | 0.0498 | 0.0879 | 0.3996 | 0.0604 | 0.4416 |
| ML SE [r1,r2,c] | 0.0104 | 0.0046 | 0.0430 | 0.0103 | 0.0050 | 0.0804 |

**<u>ML test Ctrl vs. Selection (r1 and r2 are line-specific)</u>**

c - control or selection specific

| | | | | | | |
|---|---|---|---|---|---|---|
| Line1[r1,r2,c]: | 0.4069 | 0.0547 | 0.0879 | 0.4017 | 0.0534 | 0.4423 |
| Line2[r1,r2,c]: | 0.4049 | 0.0439 | 0.0879 | 0.3782 | 0.0601 | 0.4423 |
| Line3[r1,r2,c]: | 0.4068 | 0.0507 | 0.0879 | 0.4186 | 0.0678 | 0.4423 |

c-global

| | | | | | | |
|---|---|---|---|---|---|---|
| Line1[r1,r2,c]: | 0.4083 | 0.0549 | 0.2807 | 0.4007 | 0.0532 | 0.2807 |
| Line2[r1,r2,c]: | 0.4061 | 0.0441 | 0.2807 | 0.3772 | 0.0600 | 0.2807 |
| Line3[r1,r2,c]: | 0.4082 | 0.0508 | 0.2807 | 0.4168 | 0.0675 | 0.2807 |

ML ratio (df=14 –13=1): 14.2228

**<u>*ML test Ctrl vs. Selection (r1, r2 and c are all line-specific)</u>**

| | | | | | | |
|---|---|---|---|---|---|---|
| Line1[r1,r2,c]: | 0.4067 | 0.0547 | 0.0600 | 0.4013 | 0.0533 | 0.3737 |
| Line2[r1,r2,c]: | 0.4053 | 0.0440 | 0.1495 | 0.3773 | 0.0600 | 0.2944 |
| Line3[r1,r2,c]: | 0.4067 | 0.0507 | 0.0647 | 0.4200 | 0.0680 | 0.6069 |

ML ratio (df=18 –13=5): 17.9272

ML ratio for heterogeneity (control+selection; df = 5–1=4): 17.9272 – 14.2228
…………………………………………………………………………………………………………

## markers 2-5-6 (*h-sr-e*)

| | CONTROL | | | DESICCATION | | |
|---|---|---|---|---|---|---|
| **Lines (ML estimates)** | | | | | | |
| Line1 [r1,r2,c]: | 0.2574 | 0.0547 | 0.0000 | 0.2786 | 0.0533 | 0.6284 |
| Line2 [r1,r2,c]: | 0.2773 | 0.0440 | 0.6562 | 0.2720 | 0.0600 | 0.4085 |
| Line3 [r1,r2,c]: | 0.2707 | 0.0507 | 0.0972 | 0.2893 | 0.0680 | 0.7457 |
| Bailey test for 3 × 750 | | | | | | |
| Teta [r1,r2,c]: | 0.2683 | 0.0492 | 0.0000 | 0.2799 | 0.0598 | 0.5749 |
| SE [r1,r2,c]: | 0.0093 | 0.0046 | 0.0004 | 0.0095 | 0.0050 | 0.1093 |
| Chi2: | | 10.1789 | | | 3.7820 | |
| C Chi2: | | 8.6205 | | | 1.8058 | |

Bailey for 6 × 750:

| | | | |
|---|---|---|---|
| Teta: | 0.2732 | 0.0538 | 0.0000 |
| Chi2: | 44.7914 | | |
| C chi2: | 38.1116 | | |

**<u>ML test Control vs. selection</u>**
chi^2(ctrl + sel) - chi^2(ctrl) - chi^2(sel): 30.8305
C chi^2(ctrl + sel) - chi^2(ctrl) - chi^2(sel): **<u>27.6852</u>**

**<u>ML test Ctrl Vs. Selection</u>**

| | | | | | | |
|---|---|---|---|---|---|---|
| ML est [r1,r2,c] | 0.2684 | 0.0498 | 0.2329 | 0.2800 | 0.0604 | 0.6038 |
| ML SE [r1,r2,c] | 0.0093 | 0.0046 | 0.0846 | 0.0095 | 0.0050 | 0.1124 |

**<u>*ML test Ctrl vs. Selection (r1 and r2 are line-specific)</u>**

c - control or selection specific

| | | | | | | |
|---|---|---|---|---|---|---|
| Line1[r1,r2,c]: | 0.2580 | 0.0548 | 0.2335 | 0.2786 | 0.0533 | 0.6040 |
| Line2[r1,r2,c]: | 0.2761 | 0.0438 | 0.2335 | 0.2724 | 0.0601 | 0.6040 |
| Line3[r1,r2,c]: | 0.2711 | 0.0507 | 0.2335 | 0.2889 | 0.0679 | 0.6040 |

c-global

| | | | | | | |
|---|---|---|---|---|---|---|
| Line1[r1,r2,c]: | 0.2584 | 0.0549 | 0.4420 | 0.2782 | 0.0532 | 0.4420 |
| Line2[r1,r2,c]: | 0.2769 | 0.0439 | 0.4420 | 0.2721 | 0.0600 | 0.4420 |
| Line3[r1,r2,c]: | 0.2715 | 0.0508 | 0.4420 | 0.2882 | 0.0677 | 0.4420 |

ML ratio (df=14 –13=1): 6.4781

**ML test Ctrl vs. Selection (r1, r2 and c are all line-specific)**

| | | | | | | |
|---|---|---|---|---|---|---|
| Line1[r1,r2,c]: | 0.2574 | 0.0547 | 0.0000 | 0.2786 | 0.0533 | 0.6284 |
| Line2[r1,r2,c]: | 0.2773 | 0.0440 | 0.6562 | 0.2720 | 0.0600 | 0.4085 |
| Line3[r1,r2,c]: | 0.2707 | 0.0507 | 0.0972 | 0.2893 | 0.0680 | 0.7457 |

ML ratio (df=18 –13=5): 19.6475

ML ratio for heterogeneity (control+selection; df = 5–1=4): 19.6475 – 6.4781

……………………………………………………………………………………………………

**markers 3-5-6 (*th-sr-e*)**

| | CONTROL | | | DESICCATION | | |
|---|---|---|---|---|---|---|
| **Lines (ML estimates)** | | | | | | |
| Line1 [r1,r2,c]: | 0.1320 | 0.0547 | 0.0000 | 0.1213 | 0.0533 | 0.2062 |
| Line2 [r1,r2,c]: | 0.1427 | 0.0440 | 0.0000 | 0.1666 | 0.0600 | 0.2668 |
| Line3 [r1,r2,c]: | 0.1360 | 0.0507 | 0.3877 | 0.1293 | 0.0680 | 0.1516 |
| Bailey test for 3 × 750 | | | | | | |
| Teta [r1,r2,c]: | 0.1367 | 0.0494 | 0.0000 | 0.1370 | 0.0600 | 0.1986 |
| SE [r1,r2,c]: | 0.0072 | 0.0046 | 0.0003 | 0.0072 | 0.0050 | 0.1004 |
| Chi2: | | 3.3776 | | | 8.6053 | |
| C Chi2: | | 2.1613 | | | 0.2400 | |

Bailey for 6 × 750:

| | | | |
|---|---|---|---|
| Teta: | 0.1368 | 0.0542 | 0.0000 |
| Chi2: | 18.3340 | | |
| C chi2: | 6.3654 | | |

**ML test Control vs. selection**

chi^2(ctrl + sel) - chi^2(ctrl) - chi^2(sel): 6.3512

C chi^2(ctrl + sel) - chi^2(ctrl) - chi^2(sel): **3.9641**

**ML test Ctrl Vs. Selection**

| | | | | | | |
|---|---|---|---|---|---|---|
| ML est [r1,r2,c] | 0.1369 | 0.0498 | 0.1305 | 0.1391 | 0.0604 | 0.2114 |
| ML SE [r1,r2,c] | 0.0072 | 0.0046 | 0.0911 | 0.0073 | 0.0050 | 0.1034 |

**ML test Ctrl vs. Selection (r1 and r2 are line-specific)**

c - control or selection specific

| | | | | | | |
|---|---|---|---|---|---|---|
| Line1[r1,r2,c]: | 0.1321 | 0.0547 | 0.1308 | 0.1213 | 0.0533 | 0.2112 |
| Line2[r1,r2,c]: | 0.1428 | 0.0440 | 0.1308 | 0.1666 | 0.0600 | 0.2112 |
| Line3[r1,r2,c]: | 0.1358 | 0.0506 | 0.1308 | 0.1294 | 0.0680 | 0.2112 |

c-global

| | | | | | | |
|---|---|---|---|---|---|---|
| Line1[r1,r2,c]: | 0.1321 | 0.0547 | 0.1755 | 0.1213 | 0.0533 | 0.1755 |
| Line2[r1,r2,c]: | 0.1428 | 0.0440 | 0.1755 | 0.1665 | 0.0599 | 0.1755 |
| Line3[r1,r2,c]: | 0.1358 | 0.0506 | 0.1755 | 0.1294 | 0.0680 | 0.1755 |

ML ratio (df=14 –13=1): 0.3315

**<u>*ML test Ctrl vs. Selection (r1, r2 and c are all line-specific)</u>**

| | | | | | | |
|---|---|---|---|---|---|---|
| Line1[r1,r2,c]: | 0.1320 | 0.0547 | 0.0000 | 0.1213 | 0.0533 | 0.2062 |
| Line2[r1,r2,c]: | 0.1427 | 0.0440 | 0.0000 | 0.1666 | 0.0600 | 0.2668 |
| Line3[r1,r2,c]: | 0.1360 | 0.0507 | 0.3877 | 0.1293 | 0.0680 | 0.1516 |

ML ratio (df=18 –13=5): 5.0062

ML ratio for heterogeneity (control+selection; df = 5–1=4): 5.0062 – 0.3315

……………………………………………………………………………………………………

## markers 4-5-6 (*cu-sr-e*)

| | CONTROL | | | DESICCATION | | |
|---|---|---|---|---|---|---|
| **Lines (ML estimates)** | | | | | | |
| Line1 [r1,r2,c]: | 0.0760 | 0.0547 | 0.0000 | 0.0787 | 0.0533 | 0.3176 |
| Line2 [r1,r2,c]: | 0.0867 | 0.0440 | 0.0000 | 0.1173 | 0.0600 | 0.1895 |
| Line3 [r1,r2,c]: | 0.0840 | 0.0507 | 0.3146 | 0.0867 | 0.0680 | 0.2268 |
| Bailey test for 3 × 750 | | | | | | |
| Teta [r1,r2,c]: | 0.0820 | 0.0494 | 0.0000 | 0.0919 | 0.0601 | 0.2229 |
| SE [r1,r2,c]: | 0.0058 | 0.0046 | 0.0002 | 0.0061 | 0.0050 | 0.1297 |
| Chi2: | | 2.5325 | | | 8.4027 | |
| C Chi2: | | 1.0490 | | | 0.1253 | |

Bailey for 6 × 750:

| | | | |
|---|---|---|---|
| Teta: | 0.0866 | 0.0542 | 0.0000 |
| Chi2: | 17.9580 | | |
| C chi2: | 4.1685 | | |

**<u>ML test Control vs. selection</u>**

chi^2(ctrl + sel) - chi^2(ctrl) - chi^2(sel): 7.0228

C chi^2(ctrl + sel) - chi^2(ctrl) - chi^2(sel): **2.9943**

**<u>ML test Ctrl Vs. Selection</u>**

| | | | | | | |
|---|---|---|---|---|---|---|
| ML est [r1,r2,c] | 0.0822 | 0.0498 | 0.1085 | 0.0942 | 0.0604 | 0.2340 |

| | CONTROL | SELECTION |
|---|---|---|
| ML SE [r1,r2,c] | 0.0058 0.0046 0.1078 | 0.0062 0.0050 0.1326 |

**ML test Ctrl vs. Selection (r1 and r2 are line-specific)**

c - control or selection specific

| | | | | | | |
|---|---|---|---|---|---|---|
| Line1[r1,r2,c]: | 0.0760 | 0.0547 | 0.1092 | 0.0786 | 0.0533 | 0.2333 |
| Line2[r1,r2,c]: | 0.0867 | 0.0440 | 0.1092 | 0.1174 | 0.0600 | 0.2333 |
| Line3[r1,r2,c]: | 0.0839 | 0.0506 | 0.1092 | 0.0867 | 0.0680 | 0.2333 |

c-global

| | | | | | | |
|---|---|---|---|---|---|---|
| Line1[r1,r2,c]: | 0.0760 | 0.0547 | 0.1818 | 0.0786 | 0.0533 | 0.1818 |
| Line2[r1,r2,c]: | 0.0867 | 0.0440 | 0.1818 | 0.1173 | 0.0600 | 0.1818 |
| Line3[r1,r2,c]: | 0.0840 | 0.0506 | 0.1818 | 0.0866 | 0.0680 | 0.1818 |

ML ratio (df=14 –13=1): 0.4958

***ML test Ctrl vs. Selection (r1, r2 and c are all line-specific)**

| | | | | | | |
|---|---|---|---|---|---|---|
| Line1[r1,r2,c]: | 0.0760 | 0.0547 | 0.0000 | 0.0787 | 0.0533 | 0.3176 |
| Line2[r1,r2,c]: | 0.0867 | 0.0440 | 0.0000 | 0.1173 | 0.0600 | 0.1895 |
| Line3[r1,r2,c]: | 0.0840 | 0.0507 | 0.3146 | 0.0867 | 0.0680 | 0.2268 |

ML ratio (df=18 –13=5): 2.7734

ML ratio for heterogeneity (control+selection; df = 5–1=4): 2.7734 – 0.4958

……………………………………………………………………………………………………

# Hypoxia-hyperoxia experiment experiment

## X chromosome

## Control versus hypoxia lines

### markers 1-2-3 (*y-cv-c*)

| | CONTROL | HYPOXIA |
|---|---|---|
| **Lines (ML estimates)** | | |
| Line1 [r1,r2,c]: | 0.1293 0.1960 0.6838 | 0.1760 0.2533 1.3457 |
| Line2 [r1,r2,c]: | 0.1347 0.1920 0.8767 | 0.1522 0.2497 0.7730 |
| Line3 [r1,r2,c]: | 0.1307 0.1813 0.5627 | 0.2013 0.2880 1.1267 |
| | | |
| Bailey test for 3 × 750 | | |
| Teta [r1,r2,c]: | 0.1315 0.1896 0.6958 | 0.1751 0.2630 1.0852 |
| SE [r1,r2,c]: | 0.0071 0.0083 0.0981 | 0.0080 0.0092 0.0761 |

| | | |
|---|---|---|
| Chi2: | 2.3074 | 17.7397 |
| C Chi2: | 1.6732 | 8.4690 |

Bailey for 6 × 750:

| | |
|---|---|
| Teta: | 0.1499 0.2216 0.9503 |
| Chi2: | 82.0395 |
| C chi2: | 19.8169 |

**ML test Control vs. selection**

chi^2(ctrl + sel) - chi^2(ctrl) - chi^2(sel): 61.9924
C chi^2(ctrl + sel) - chi^2(ctrl) - chi^2(sel): **9.6746**

**ML test Ctrl Vs. Selection**

| | | | | | | |
|---|---|---|---|---|---|---|
| ML est [r1,r2,c] | 0.1316 | 0.1898 | 0.7121 | 0.1765 | 0.2637 | 1.1082 |
| ML SE [r1,r2,c] | 0.0071 | 0.0083 | 0.0994 | 0.0080 | 0.0093 | 0.0780 |

**ML test Ctrl vs. Selection (r1 and r2 are line-specific)**

c - control or selection specific

| | | | | | | |
|---|---|---|---|---|---|---|
| Line1[r1,r2,c]: | 0.1294 | 0.1960 | 0.7128 | 0.1763 | 0.2538 | 1.1049 |
| Line2[r1,r2,c]: | 0.1345 | 0.1917 | 0.7128 | 0.1519 | 0.2491 | 1.1049 |
| Line3[r1,r2,c]: | 0.1308 | 0.1816 | 0.7128 | 0.2014 | 0.2881 | 1.1049 |

c-global

| | | | | | | |
|---|---|---|---|---|---|---|
| Line1[r1,r2,c]: | 0.1294 | 0.1962 | 0.8238 | 0.1759 | 0.2532 | 0.9815 |
| Line2[r1,r2,c]: | 0.1346 | 0.1919 | 0.8238 | 0.1522 | 0.2497 | 0.9815 |
| Line3[r1,r2,c]: | 0.1309 | 0.1816 | 0.823 | 0.2013 | 0.2879 | 0.9815 |

ML ratio (df=14 –13=1): 8.8832

***ML test Ctrl vs. Selection (r1, r2 and c are all line-specific)**

| | | | | | | |
|---|---|---|---|---|---|---|
| Line1[r1,r2,c]: | 0.1293 | 0.1960 | 0.6838 | 0.1760 | 0.2533 | 1.3457 |
| Line2[r1,r2,c]: | 0.1347 | 0.1920 | 0.8767 | 0.1522 | 0.2497 | 0.7730 |
| Line3[r1,r2,c]: | 0.1307 | 0.1813 | 0.5627 | 0.2013 | 0.2880 | 1.1 |

ML ratio (df=18 –13=5): 18.4754

ML ratio for heterogeneity (control+selection; df = 5–1=4): 18.4754 – 8.8832

……………………………………………………………………………………………………

## markers 1-2-4 (*y-cv-f*)

| | CONTROL | HYPOXIA |
|---|---|---|
| **Lines (ML estimates)** | | |
| Line1 [r1,r2,c]: | 0.1293 0.3667 1.0965 | 0.1767 0.3976 2.1531 |
| Line2 [r1,r2,c]: | 0.1347 0.3387 1.2571 | 0.1522 0.3858 1.7278 |
| Line3 [r1,r2,c]: | 0.1307 0.3520 0.8117 | 0.2019 0.3663 2.4045 |

| Bailey test for 3 × 750 | | |
|---|---|---|
| Teta [r1,r2,c]: | 0.1320 0.3532 1.0403 | 0.1810 0.3903 2.1502 |
| SE [r1,r2,c]: | 0.0071 0.0100 0.0734 | 0.0079 0.0098 0.0614 |
| Chi2: | 7.6319 | 38.4146 |
| C Chi2: | 6.3338 | 30.4396 |

Bailey for 6 × 750:

| | |
|---|---|
| Teta: | 0.1606 0.3944 1.8525 |
| Chi2: | 257.9640 |
| C chi2: | 214.8462 |

**ML test Control vs. selection**

chi^2(ctrl + sel) - chi^2(ctrl) - chi^2(sel): 211.9175
C chi^2(ctrl + sel) - chi^2(ctrl) - chi^2(sel): **178.0727**

**ML test Ctrl Vs. Selection**

| | | |
|---|---|---|
| ML est [r1,r2,c] | 0.1316 0.3524 1.0544 | 0.1769 0.3832 2.1164 |
| ML SE [r1,r2,c] | 0.0071 0.0101 0.0741 | 0.0080 0.0102 0.0621 |

**ML test Ctrl vs. Selection (r1 and r2 are line-specific)**

c - control or selection specific

| | | | | | | |
|---|---|---|---|---|---|---|
| Line1[r1,r2,c]: | 0.1294 | 0.3667 | 1.0543 | 0.1778 | 0.4000 | 2.1329 |
| Line2[r1,r2,c]: | 0.1348 | 0.3390 | 1.0543 | 0.1397 | 0.3542 | 2.1329 |
| Line3[r1,r2,c]: | 0.1305 | 0.3516 | 1.0543 | 0.2202 | 0.3996 | 2.1329 |

c-global

| | | | | | | |
|---|---|---|---|---|---|---|
| Line1[r1,r2,c]: | 0.1227 | 0.3478 | 1.7057 | 0.1918 | 0.4316 | 1.7841 |
| Line2[r1,r2,c]: | 0.1299 | 0.3268 | 1.7057 | 0.1509 | 0.3827 | 1.7841 |
| Line3[r1,r2,c]: | 0.1218 | 0.3282 | 1.7057 | 0.2342 | 0.4249 | 1.7841 |

ML ratio (df=14 –13=1): 123.5167

***ML test Ctrl vs. Selection (r1, r2 and c are all line-specific)**

| | | | | | | |
|---|---|---|---|---|---|---|
| Line1[r1,r2,c]: | 0.1293 | 0.3667 | 1.0965 | 0.1767 | 0.3976 | 2.1531 |
| Line2[r1,r2,c]: | 0.1347 | 0.3387 | 1.2571 | 0.1522 | 0.3858 | 1.7278 |
| Line3[r1,r2,c]: | 0.1307 | 0.3520 | 0.8117 | 0.2019 | 0.3663 | 2.4045 |

ML ratio (df=18 –13=5): 148.0847

ML ratio for heterogeneity (control+selection; df = 5–1=4): 148.0847 – 123.5167
……………………………………………………………………………………………………

**markers 1-3-4 (*y-v-f*)**

**CONTROL** **HYPOXIA**

**Lines (ML estimates)**

| | | |
|---|---|---|
| Line1 [r1,r2,c]: | 0.2907 0.2267 0.9512 | 0.3106 0.3092 1.8120 |

| | | |
|---|---|---|
| Line2 [r1,r2,c]: | 0.2813 0.2293 1.1779 | 0.3436 0.2888 1.4957 |
| Line3 [r1,r2,c]: | 0.2853 0.2213 0.7812 | 0.3596 0.2754 2.1327 |

Bailey test for 3 × 750

| | | |
|---|---|---|
| Teta [r1,r2,c]: | 0.2864 0.2265 0.9598 | 0.3520 0.2971 1.8249 |
| SE [r1,r2,c]: | 0.0095 0.0088 0.0608 | 0.0096 0.0094 0.0512 |
| Chi2: | 7.2385 | 50.0856 |
| C Chi2: | 6.9869 | 36.8384 |

Bailey for 6 × 750:

| | |
|---|---|
| Teta: | 0.3291 0.2669 1.5869 |
| Chi2: | 271.4775 |
| C chi2: | 185.5989 |

**ML test Control vs. selection**

chi^2(ctrl + sel) - chi^2(ctrl) - chi^2(sel): 214.1534

C chi^2(ctrl + sel) - chi^2(ctrl) - chi^2(sel): **141.7737**

**ML test Ctrl Vs. Selection**

| | | |
|---|---|---|
| ML est [r1,r2,c] | 0.2858 0.2258 0.9712 | 0.3379 0.2911 1.8081 |
| ML SE [r1,r2,c] | 0.0095 0.0088 0.0613 | 0.0100 0.0096 0.0518 |

**ML test Ctrl vs. Selection (r1 and r2 are line-specific)**

c - control or selection specific

| | | | | | | |
|---|---|---|---|---|---|---|
| Line1[r1,r2,c]: | 0.2907 | 0.2267 | 0.9715 | 0.3089 | 0.3076 | 1.8343 |
| Line2[r1,r2,c]: | 0.2811 | 0.2292 | 0.9715 | 0.3183 | 0.2675 | 1.8343 |
| Line3[r1,r2,c]: | 0.2855 | 0.2215 | 0.9715 | 0.3930 | 0.3009 | 1.8343 |

c-global

| | | | | | | |
|---|---|---|---|---|---|---|
| Line1[r1,r2,c]: | 0.2767 | 0.2158 | 1.5519 | 0.3239 | 0.3225 | 1.5519 |
| Line2[r1,r2,c]: | 0.2729 | 0.2225 | 1.5519 | 0.3406 | 0.2862 | 1.5519 |
| Line3[r1,r2,c]: | 0.2691 | 0.2087 | 1.5519 | 0.4039 | 0.3093 | 1.5519 |

ML ratio (df=14 –13=1): 111.1525

***ML test Ctrl vs. Selection (r1, r2 and c are all line-specific)**

| | | | | | | |
|---|---|---|---|---|---|---|
| Line1[r1,r2,c]: | 0.2907 | 0.2267 | 0.9512 | 0.3106 | 0.3092 | 1.8120 |
| Line2[r1,r2,c]: | 0.2813 | 0.2293 | 1.1779 | 0.3436 | 0.2888 | 1.4957 |
| Line3[r1,r2,c]: | 0.2853 | 0.2213 | 0.7812 | 0.3596 | 0.2754 | 2.1327 |

ML ratio (df=18 –13=5): 143.6906

ML ratio for heterogeneity (control+selection; df = 5–1=4): 143.6906 – 111.1525

……………………………………………………………………………………………………

**markers 2-3-4 (*cv-v-f*)**

**CONTROL** **HYPOXIA**

**Lines (ML estimates)**

| | | | | | | |
|---|---|---|---|---|---|---|
| Line1 [r1,r2,c]: | 0.1960 | 0.2267 | 0.6303 | 0.2543 | 0.3092 | 1.0552 |
| Line2 [r1,r2,c]: | 0.1920 | 0.2293 | 0.9387 | 0.2500 | 0.2888 | 1.0556 |
| Line3 [r1,r2,c]: | 0.1813 | 0.2213 | 0.6312 | 0.2888 | 0.2754 | 1.2440 |

Bailey test for 3 × 750

| | | | | | | |
|---|---|---|---|---|---|---|
| Teta [r1,r2,c]: | 0.1897 | 0.2259 | 0.7201 | 0.2645 | 0.2905 | 1.1205 |
| SE [r1,r2,c]: | 0.0083 | 0.0088 | 0.0726 | 0.0093 | 0.0096 | 0.0559 |
| Chi2: | | 4.4341 | | | 8.4802 | |
| C Chi2: | | 3.7040 | | | 2.5554 | |

Bailey for 6 × 750:

| | | | |
|---|---|---|---|
| Teta: | 0.2218 | 0.2542 | 0.9862 |
| Chi2: | 93.2994 | | |
| C chi2: | 25.4346 | | |

**ML test Control vs. selection**

chi^2(ctrl + sel) - chi^2(ctrl) - chi^2(sel): 80.3851

C chi^2(ctrl + sel) - chi^2(ctrl) - chi^2(sel): **19.1752**

**ML test Ctrl Vs. Selection**

| | | | | | | |
|---|---|---|---|---|---|---|
| ML est [r1,r2,c] | 0.1898 | 0.2258 | 0.7365 | 0.2644 | 0.2911 | 1.1179 |
| ML SE [r1,r2,c] | 0.0083 | 0.0088 | 0.0736 | 0.0093 | 0.0096 | 0.0560 |

**ML test Ctrl vs. Selection (r1 and r2 are line-specific)**

c - control or selection specific

| | | | | | | |
|---|---|---|---|---|---|---|
| Line1[r1,r2,c]: | 0.1963 | 0.2271 | 0.7365 | 0.2540 | 0.3088 | 1.1214 |
| Line2[r1,r2,c]: | 0.1914 | 0.2286 | 0.7365 | 0.2497 | 0.2884 | 1.1214 |
| Line3[r1,r2,c]: | 0.1816 | 0.2217 | 0.7365 | 0.2895 | 0.2761 | 1.1214 |

c-global

| | | | | | | |
|---|---|---|---|---|---|---|
| Line1[r1,r2,c]: | 0.1960 | 0.2267 | 0.9992 | 0.2543 | 0.3092 | 0.9992 |
| Line2[r1,r2,c]: | 0.1920 | 0.2293 | 0.9992 | 0.2500 | 0.2887 | 0.9992 |
| Line3[r1,r2,c]: | 0.1813 | 0.2213 | 0.9992 | 0.2887 | 0.2754 | 0.9992 |

ML ratio (df=14 –13=1): 16.0655

***ML test Ctrl vs. Selection (r1, r2 and c are all line-specific)**

| | | | | | | |
|---|---|---|---|---|---|---|
| Line1[r1,r2,c]: | 0.1960 | 0.2267 | 0.6303 | 0.2543 | 0.3092 | 1.0552 |
| Line2[r1,r2,c]: | 0.1920 | 0.2293 | 0.9387 | 0.2500 | 0.2888 | 1.0556 |
| Line3[r1,r2,c]: | 0.1813 | 0.2213 | 0.6312 | 0.2888 | 0.2754 | 1.2440 |

ML ratio (df=18 –13=5): 22.4909

ML ratio for heterogeneity (control+selection; df = 5–1=4): 22.4909 – 16.0655

**…………………………………………………………………………………………………………..**

# Control versus hyperoxia lines

## markers 1-2-3 (*y-cv-c*)

| | CONTROL | HYPEROXIA |
|---|---|---|
| **Lines (ML estimates)** | | |
| Line1 [r1,r2,c]: | 0.1293 0.1960 0.6838 | 0.1773 0.2907 0.8536 |
| Line2 [r1,r2,c]: | 0.1347 0.1920 0.8767 | 0.1493 0.2587 0.7939 |
| Line3 [r1,r2,c]: | 0.1307 0.1813 0.5627 | 0.1547 0.2693 0.9922 |
| | | |
| Bailey test for 3 × 750 | | |
| Teta [r1,r2,c]: | 0.1315 0.1896 0.6958 | 0.1598 0.2727 0.8750 |
| SE [r1,r2,c]: | 0.0071 0.0083 0.0981 | 0.0077 0.0094 0.0756 |
| Chi2: | 2.3074 | 5.6211 |
| C Chi2: | 1.6732 | 1.0768 |

Bailey for 6 × 750:
Teta: 0.1442 0.2257 0.8072
Chi2: 62.5576
C chi2: 4.8769

**ML test Control vs. selection**
chi^2(ctrl + sel) - chi^2(ctrl) - chi^2(sel): 54.6292
C chi^2(ctrl + sel) - chi^2(ctrl) - chi^2(sel): **2.1269**

**ML test Ctrl Vs. Selection**

| | | |
|---|---|---|
| ML est [r1,r2,c] | 0.1316 0.1898 0.7121 | 0.1604 0.2729 0.8831 |
| ML SE [r1,r2,c] | 0.0071 0.0083 0.0994 | 0.0077 0.0094 0.0762 |
| ML est - same C | 0.1316 0.1899 0.8259 | 0.1603 0.2727 0.8259 |

ML chi^2 = **1.7989**

**ML test Ctrl vs. Selection (r1 and r2 are line-specific)**

c - control or selection specific

| | | | | | | |
|---|---|---|---|---|---|---|
| Line1[r1,r2,c]: | 0.1294 | 0.1960 | 0.7128 | 0.1774 | 0.2907 | 0.8798 |
| Line2[r1,r2,c]: | 0.1345 | 0.1917 | 0.7128 | 0.1494 | 0.2588 | 0.8798 |
| Line3[r1,r2,c]: | 0.1308 | 0.1816 | 0.7128 | 0.1545 | 0.2691 | 0.8798 |

c-global

| | | | | | | |
|---|---|---|---|---|---|---|
| Line1[r1,r2,c]: | 0.1294 | 0.1962 | 0.8238 | 0.1773 | 0.2905 | 0.8238 |
| Line2[r1,r2,c]: | 0.1346 | 0.1919 | 0.8238 | 0.1494 | 0.2587 | 0.8238 |
| Line3[r1,r2,c]: | 0.1309 | 0.1816 | 0.8238 | 0.1544 | 0.2688 | 0.8238 |

ML ratio (df=14 –13=1): 1.7242

***ML test Ctrl vs. Selection (r1, r2 and c are all line-specific)**

| | | | | | | |
|---|---|---|---|---|---|---|
| Line1[r1,r2,c]: | 0.1293 | 0.1960 | 0.6838 | 0.1773 | 0.2907 | 0.8536 |

| | | | | | | |
|---|---|---|---|---|---|---|
| Line2[r1,r2,c]: | 0.1347 | 0.1920 | 0.8767 | 0.1493 | 0.2587 | 0.7939 |
| Line3[r1,r2,c]: | 0.1307 | 0.1813 | 0.5627 | 0.1547 | 0.2693 | 0.9922 |

ML ratio (df=18 –13=5): 4.4963

ML ratio for heterogeneity (control+selection; df = 5–1=4): 4.4963 – 1.7242
………………………………………………………………………………………………………

## markers 1-2-4 (*y-cv-f*)

| | CONTROL | | | HYPEROXIA | | |
|---|---|---|---|---|---|---|
| **Lines (ML estimates)** | | | | | | |
| Line1 [r1,r2,c]: | 0.1293 | 0.3667 | 1.0965 | 0.1773 | 0.3680 | 2.3905 |
| Line2 [r1,r2,c]: | 0.1347 | 0.3387 | 1.2571 | 0.1493 | 0.3800 | 1.7152 |
| Line3 [r1,r2,c]: | 0.1307 | 0.3520 | 0.8117 | 0.1547 | 0.3560 | 2.1309 |
| Bailey test for 3 × 750 | | | | | | |
| Teta [r1,r2,c]: | 0.1320 | 0.3532 | 1.0403 | 0.1642 | 0.3779 | 2.1283 |
| SE [r1,r2,c]: | 0.0071 | 0.0100 | 0.0734 | 0.0076 | 0.0098 | 0.0662 |
| Chi2: | | 7.6319 | | | 30.8598 | |
| C Chi2: | | 6.3338 | | | 27.4331 | |

Bailey for 6 × 750:

| | | | |
|---|---|---|---|
| Teta: | 0.1527 | 0.3863 | 1.7485 |
| Chi2: | 204.1173 | | |
| C chi2: | 191.4714 | | |

**ML test Control vs. selection**

chi^2(ctrl + sel) - chi^2(ctrl) - chi^2(sel): 165.6257
C chi^2(ctrl + sel) - chi^2(ctrl) - chi^2(sel): **157.7044**

**ML test Ctrl Vs. Selection**

| | | | | | | |
|---|---|---|---|---|---|---|
| ML est [r1,r2,c] | 0.1316 | 0.3524 | 1.0544 | 0.1604 | 0.3680 | 2.0926 |
| ML SE [r1,r2,c] | 0.0071 | 0.0101 | 0.0741 | 0.0077 | 0.0102 | 0.0673 |

**ML test Ctrl vs. Selection (r1 and r2 are line-specific)**

c - control or selection specific

| | | | | | | |
|---|---|---|---|---|---|---|
| Line1[r1,r2,c]: | 0.1294 | 0.3667 | 1.0543 | 0.1923 | 0.3991 | 2.1120 |
| Line2[r1,r2,c]: | 0.1348 | 0.3390 | 1.0543 | 0.1380 | 0.3513 | 2.1120 |
| Line3[r1,r2,c]: | 0.1305 | 0.3516 | 1.0543 | 0.1553 | 0.3574 | 2.1120 |

c-global

| | | | | | | |
|---|---|---|---|---|---|---|
| Line1[r1,r2,c]: | 0.1227 | 0.3478 | 1.7057 | 0.1997 | 0.4143 | 1.7057 |
| Line2[r1,r2,c]: | 0.1299 | 0.3268 | 1.7057 | 0.1495 | 0.3804 | 1.7057 |
| Line3[r1,r2,c]: | 0.1218 | 0.3282 | 1.7057 | 0.1628 | 0.3747 | 1.7057 |

ML ratio (df=14 –13=1): 110.3310

***ML test Ctrl vs. Selection (r1, r2 and c  are all line-specific)**

| | | | | | | |
|---|---|---|---|---|---|---|
| Line1[r1,r2,c]: | 0.1293 | 0.3667 | 1.0965 | 0.1773 | 0.3680 | 2.3905 |
| Line2[r1,r2,c]: | 0.1347 | 0.3387 | 1.2571 | 0.1493 | 0.3800 | 1.7152 |
| Line3[r1,r2,c]: | 0.1307 | 0.3520 | 0.8117 | 0.1547 | 0.3560 | 2.1309 |

ML ratio (df=18 –13=5): 133.9141

ML ratio for heterogeneity (control+selection; df = 5–1=4): 133.9141 – 110.3310
……………………………………………………………………………………………………

**markers 1-3-4 (*y-v-f*)**

CONTROL HYPEROXIA

**Lines (ML estimates)**

| | CONTROL | | | HYPEROXIA | | |
|---|---|---|---|---|---|---|
| Line1 [r1,r2,c]: | 0.2907 | 0.2267 | 0.9512 | 0.3800 | 0.2827 | 1.9985 |
| Line2 [r1,r2,c]: | 0.2813 | 0.2293 | 1.1779 | 0.3467 | 0.2573 | 1.5096 |
| Line3 [r1,r2,c]: | 0.2853 | 0.2213 | 0.7812 | 0.3413 | 0.2867 | 1.7987 |

Bailey test for 3 × 750

| | | | | | | |
|---|---|---|---|---|---|---|
| Teta [r1,r2,c]: | 0.2864 | 0.2265 | 0.9598 | 0.3636 | 0.2790 | 1.8192 |
| SE [r1,r2,c]: | 0.0095 | 0.0088 | 0.0608 | 0.0099 | 0.0093 | 0.0504 |
| Chi2: | | 7.2385 | | | 34.0648 | |
| C Chi2: | | 6.9869 | | | 20.3333 | |

Bailey for 6 × 750:

| | | | |
|---|---|---|---|
| Teta: | 0.3359 | 0.2563 | 1.5837 |
| Chi2: | 248.3792 | | |
| C chi2: | 161.0763 | | |

**ML test Control vs. selection**

chi^2(ctrl + sel) - chi^2(ctrl) - chi^2(sel): 207.0759
C chi^2(ctrl + sel) - chi^2(ctrl) - chi^2(sel): **133.7561**

**ML test Ctrl Vs. Selection**

| | | | | | | |
|---|---|---|---|---|---|---|
| ML est [r1,r2,c] | 0.2858 | 0.2258 | 0.9712 | 0.3560 | 0.2756 | 1.7850 |
| ML SE [r1,r2,c] | 0.0095 | 0.0088 | 0.0613 | 0.0101 | 0.0094 | 0.0511 |

**ML test Ctrl vs. Selection (r1 and r2 are  line-specific)**

c - control or selection specific

| | | | | | | |
|---|---|---|---|---|---|---|
| Line1[r1,r2,c]: | 0.2907 | 0.2267 | 0.9715 | 0.4038 | 0.3004 | 1.7996 |
| Line2[r1,r2,c]: | 0.2811 | 0.2292 | 0.9715 | 0.3274 | 0.2431 | 1.7996 |
| Line3[r1,r2,c]: | 0.2855 | 0.2215 | 0.9715 | 0.3413 | 0.2866 | 1.7996 |

c-global

| | | | | | | |
|---|---|---|---|---|---|---|
| Line1[r1,r2,c]: | 0.2775 | 0.2164 | 1.5349 | 0.4195 | 0.3120 | 1.5349 |
| Line2[r1,r2,c]: | 0.2735 | 0.2230 | 1.5349 | 0.3455 | 0.2565 | 1.5349 |

| | | | | | | |
|---|---|---|---|---|---|---|
| Line3[r1,r2,c]: | 0.2699 | 0.2094 | 1.5349 | 0.3563 | 0.2992 | 1.5349 |

ML ratio (df=14 –13=1): 104.1546

**<u>*ML test Ctrl vs. Selection (r1, r2 and c are all line-specific)</u>**

| | | | | | | |
|---|---|---|---|---|---|---|
| Line1[r1,r2,c]: | 0.2907 | 0.2267 | 0.9512 | 0.3800 | 0.2827 | 1.9985 |
| Line2[r1,r2,c]: | 0.2813 | 0.2293 | 1.1779 | 0.3467 | 0.2573 | 1.5096 |
| Line3[r1,r2,c]: | 0.2853 | 0.2213 | 0.7812 | 0.3413 | 0.2867 | 1.7987 |

ML ratio (df=18 –13=5): 126.2411

ML ratio for heterogeneity (control+selection; df = 5–1=4): 126.2411 – 104.1546
………………………………………………………………………………………………………

## markers 2-3-4 (*cv-v-f*)

| | CONTROL | | | HYPEROXIA | | |
|---|---|---|---|---|---|---|
| **Lines (ML estimates)** | | | | | | |
| Line1 [r1,r2,c]: | 0.1960 | 0.2267 | 0.6303 | 0.2907 | 0.2827 | 1.2496 |
| Line2 [r1,r2,c]: | 0.1920 | 0.2293 | 0.9387 | 0.2587 | 0.2573 | 1.0216 |
| Line3 [r1,r2,c]: | 0.1813 | 0.2213 | 0.6312 | 0.2693 | 0.2867 | 1.2952 |
| Bailey test for 3 × 750 | | | | | | |
| Teta [r1,r2,c]: | 0.1897 | 0.2259 | 0.7201 | 0.2725 | 0.2754 | 1.2030 |
| SE [r1,r2,c]: | 0.0083 | 0.0088 | 0.0726 | 0.0094 | 0.0094 | 0.0572 |
| Chi2: | | 4.4341 | | | 8.1780 | |
| C Chi2: | | 3.7040 | | | 4.0829 | |

Bailey for 6 × 750:

| | | | |
|---|---|---|---|
| Teta: | 0.2255 | 0.2474 | 1.0386 |
| Chi2: | 99.4680 | | |
| C chi2: | 34.9232 | | |

**<u>ML test Control vs. selection</u>**
chi^2(ctrl + sel) - chi^2(ctrl) - chi^2(sel): 86.8558
C chi^2(ctrl + sel) - chi^2(ctrl) - chi^2(sel): **<u>27.1363</u>**

**<u>ML test Ctrl Vs. Selection</u>**

| | | | | | | |
|---|---|---|---|---|---|---|
| ML est [r1,r2,c] | 0.1898 | 0.2258 | 0.7365 | 0.2729 | 0.2756 | 1.1998 |
| ML SE [r1,r2,c] | 0.0083 | 0.0088 | 0.0736 | 0.0094 | 0.0094 | 0.0576 |

**<u>ML test Ctrl vs. Selection (r1 and r2 are line-specific)</u>**

c - control or selection specific

| | | | | | | |
|---|---|---|---|---|---|---|
| Line1[r1,r2,c]: | 0.1963 | 0.2271 | 0.7366 | 0.2912 | 0.2832 | 1.2020 |
| Line2[r1,r2,c]: | 0.1914 | 0.2286 | 0.7366 | 0.2574 | 0.2561 | 1.2020 |
| Line3[r1,r2,c]: | 0.1816 | 0.2217 | 0.7366 | 0.2702 | 0.2876 | 1.2020 |

c-global

| | | | | | | |
|---|---|---|---|---|---|---|
| Line1[r1,r2,c]: | 0.1957 | 0.2263 | 1.0525 | 0.2912 | 0.2831 | 1.0525 |
| Line2[r1,r2,c]: | 0.1919 | 0.2292 | 1.0525 | 0.2586 | 0.2573 | 1.0525 |
| Line3[r1,r2,c]: | 0.1811 | 0.2210 | 1.0525 | 0.2699 | 0.2872 | 1.0525 |

ML ratio (df=14 –13=1): 22.8291

**<u>*ML test Ctrl vs. Selection (r1, r2 and c are all line-specific)</u>**

| | | | | | | |
|---|---|---|---|---|---|---|
| Line1[r1,r2,c]: | 0.1960 | 0.2267 | 0.6303 | 0.2907 | 0.2827 | 1.2496 |
| Line2[r1,r2,c]: | 0.1920 | 0.2293 | 0.9387 | 0.2907 | 0.2827 | 1.2496 |
| Line3[r1,r2,c]: | 0.1813 | 0.2213 | 0.6312 | 0.2693 | 0.2867 | 1.2952 |

ML ratio (df=18 –13=5): 30.6501

ML ratio for heterogeneity (control+selection; df = 5–1=4): 30.6501 – 22.8291
……………………………………………………………………………………………………

## 2L chromosome

## <u>Control and hypoxia lines</u>

### <u>markers 1-2-3 (*net-dp-b*)</u>

| | CONTROL | | | HYPOXIA | | |
|---|---|---|---|---|---|---|
| **<u>Lines (ML estimates)</u>** | | | | | | |
| Line1 [r1,r2,c]: | 0.1253 | 0.2960 | 0.2875 | 0.1667 | 0.3693 | 1.4296 |
| Line2 [r1,r2,c]: | 0.1067 | 0.3093 | 0.1616 | 0.1600 | 0.3707 | 1.2590 |
| Line3 [r1,r2,c]: | 0.1373 | 0.3093 | 0.5022 | 0.1853 | 0.3760 | 1.2054 |
| | | | | | | |
| Bailey test for 3 × 750 | | | | | | |
| Teta [r1,r2,c]: | 0.1218 | 0.3042 | 0.2784 | 0.1704 | 0.3727 | 1.2922 |
| SE [r1,r2,c]: | 0.0069 | 0.0097 | 0.0529 | 0.0079 | 0.0102 | 0.0621 |
| Chi2: | | 9.8633 | | | 4.0561 | |
| C Chi2: | | 6.3762 | | | 2.4112 | |

Bailey for 6 × 750:
Teta: 0.1417 0.3389 0.7290
Chi2: 221.4883
C chi2: 165.0162

**<u>ML test Control vs. selection</u>**
chi^2(ctrl + sel) - chi^2(ctrl) - chi^2(sel): 207.5689
C chi^2(ctrl + sel) - chi^2(ctrl) - chi^2(sel): **<u>156.2288</u>**

**<u>ML test Ctrl Vs. Selection</u>**

| | | | | | | |
|---|---|---|---|---|---|---|
| ML est [r1,r2,c] | 0.1231 | 0.3049 | 0.3315 | 0.1707 | 0.3720 | 1.2951 |
| ML SE [r1,r2,c] | 0.0069 | 0.0097 | 0.0579 | 0.0079 | 0.0102 | 0.0623 |

**ML test Ctrl vs. Selection (r1 and r2 are line-specific)**

c - control or selection specific

| | | | | | | |
|---|---|---|---|---|---|---|
| Line1[r1,r2,c]: | 0.1255 | 0.2964 | 0.3323 | 0.1676 | 0.3714 | 1.2937 |
| Line2[r1,r2,c]: | 0.1071 | 0.3107 | 0.3323 | 0.1598 | 0.3701 | 1.2937 |
| Line3[r1,r2,c]: | 0.1366 | 0.3076 | 0.3323 | 0.1845 | 0.3744 | 1.2937 |

c-global

| | | | | | | |
|---|---|---|---|---|---|---|
| Line1[r1,r2,c]: | 0.1255 | 0.2963 | 0.9701 | 0.1664 | 0.3687 | 0.9701 |
| Line2[r1,r2,c]: | 0.1068 | 0.3097 | 0.9701 | 0.1598 | 0.3703 | 0.9701 |
| Line3[r1,r2,c]: | 0.1375 | 0.3096 | 0.9701 | 0.1852 | 0.3756 | 0.9701 |

ML ratio (df=14 –13=1): 96.1960

***ML test Ctrl vs. Selection (r1, r2 and c are all line-specific)**

| | | | | | | |
|---|---|---|---|---|---|---|
| Line1[r1,r2,c]: | 0.1253 | 0.2960 | 0.2875 | 0.1667 | 0.3693 | 1.4296 |
| Line2[r1,r2,c]: | 0.1067 | 0.3093 | 0.1616 | 0.1600 | 0.3707 | 1.2590 |
| Line3[r1,r2,c]: | 0.1373 | 0.3093 | 0.5022 | 0.1853 | 0.3760 | 1.2054 |

ML ratio (df=18 –13=5): 104.7038

ML ratio for heterogeneity (control+selection; df = 5–1=4): 104.7038 – 96.1960
……………………………………………………………………………………………………

## markers 1-2-4 (*net-dp-pk*)

| | CONTROL | | | HYPOXIA | | |
|---|---|---|---|---|---|---|
| **Lines (ML estimates)** | | | | | | |
| Line1 [r1,r2,c]: | 0.1253 | 0.3573 | 0.3573 | 0.1667 | 0.3853 | 1.5986 |
| Line2 [r1,r2,c]: | 0.1067 | 0.3520 | 0.2486 | 0.1600 | 0.3760 | 1.5071 |
| Line3 [r1,r2,c]: | 0.1373 | 0.3413 | 0.5120 | 0.1853 | 0.3813 | 1.3961 |
| Bailey test for 3 × 750 | | | | | | |
| Teta [r1,r2,c]: | 0.1220 | 0.3498 | 0.3571 | 0.1703 | 0.3817 | 1.4976 |
| SE [r1,r2,c]: | 0.0069 | 0.0101 | 0.0547 | 0.0079 | 0.0102 | 0.0612 |
| Chi2: | | 7.3222 | | | 3.8415 | |
| C Chi2: | | 3.7141 | | | 2.0229 | |

Bailey for 6 × 750:

| | | | |
|---|---|---|---|
| Teta: | 0.1453 | 0.3729 | 0.8967 |
| Chi2: | 242.9311 | | |
| C chi2: | 204.4727 | | |

**ML test Control vs. selection**
chi^2(ctrl + sel) - chi^2(ctrl) - chi^2(sel): 231.7675
C chi^2(ctrl + sel) - chi^2(ctrl) - chi^2(sel): **198.7356**

**ML test Ctrl Vs. Selection**

| | | | | | | |
|---|---|---|---|---|---|---|
| ML est [r1,r2,c] | 0.1231 | 0.3502 | 0.3814 | 0.1707 | 0.3809 | 1.4973 |
| ML SE [r1,r2,c] | 0.0069 | 0.0101 | 0.0566 | 0.0079 | 0.0102 | 0.0614 |

**ML test Ctrl vs. Selection (r1 and r2 are line-specific)**

c - control or selection specific

| | | | | | | |
|---|---|---|---|---|---|---|
| Line1[r1,r2,c]: | 0.1254 | 0.3576 | 0.3821 | 0.1682 | 0.3888 | 1.4975 |
| Line2[r1,r2,c]: | 0.1071 | 0.3533 | 0.3821 | 0.1601 | 0.3763 | 1.4975 |
| Line3[r1,r2,c]: | 0.1367 | 0.3398 | 0.3821 | 0.1835 | 0.3777 | 1.4975 |

c-global

| | | | | | | |
|---|---|---|---|---|---|---|
| Line1[r1,r2,c]: | 0.1247 | 0.3555 | 1.0839 | 0.1676 | 0.3876 | 1.0839 |
| Line2[r1,r2,c]: | 0.1062 | 0.3503 | 1.0839 | 0.1607 | 0.3776 | 1.0839 |
| Line3[r1,r2,c]: | 0.1368 | 0.3399 | 1.0839 | 0.1861 | 0.3828 | 1.0839 |

ML ratio (df=14 –13=1): 139.8527

***ML test Ctrl vs. Selection (r1, r2 and c are all line-specific)**

| | | | | | | |
|---|---|---|---|---|---|---|
| Line1[r1,r2,c]: | 0.1253 | 0.3573 | 0.3573 | 0.1667 | 0.3853 | 1.5986 |
| Line2[r1,r2,c]: | 0.1067 | 0.3520 | 0.2486 | 0.1600 | 0.3760 | 1.5071 |
| Line3[r1,r2,c]: | 0.1373 | 0.3413 | 0.5120 | 0.1853 | 0.3813 | 1.3961 |

ML ratio (df=18 –13=5): 145.3805

ML ratio for heterogeneity (control+selection; df = 5–1=4): 145.3805 – 139.8527
………………………………………………………………………………………………………

## markers 1-2-5 (*net-dp-cn*)

| | CONTROL | | | HYPOXIA | | |
|---|---|---|---|---|---|---|
| **Lines (ML estimates)** | | | | | | |
| Line1 [r1,r2,c]: | 0.1253 | 0.3760 | 0.3678 | 0.1667 | 0.3893 | 1.6027 |
| Line2 [r1,r2,c]: | 0.1067 | 0.3733 | 0.2009 | 0.1600 | 0.3760 | 1.5514 |
| Line3 [r1,r2,c]: | 0.1373 | 0.3547 | 0.5475 | 0.1853 | 0.3987 | 1.3895 |
| Bailey test for 3 × 750 | | | | | | |
| Teta [r1,r2,c]: | 0.1220 | 0.3672 | 0.3373 | 0.1705 | 0.3889 | 1.5055 |
| SE [r1,r2,c]: | 0.0069 | 0.0102 | 0.0516 | 0.0079 | 0.0103 | 0.0600 |
| Chi2: | | 11.1346 | | | 4.3411 | |
| C Chi2: | | 7.2486 | | | 2.5950 | |

Bailey for 6 × 750:

| | | | |
|---|---|---|---|
| Teta: | 0.1464 | 0.3868 | 0.8644 |
| Chi2: | 270.2017 | | |
| C chi2: | 235.4225 | | |

**ML test Control vs. selection**

chi^2(ctrl + sel) - chi^2(ctrl) - chi^2(sel): 254.7261
C chi^2(ctrl + sel) - chi^2(ctrl) - chi^2(sel): **225.5789**

**ML test Ctrl Vs. Selection**

| | | | | | | |
|---|---|---|---|---|---|---|
| ML est [r1,r2,c] | 0.1231 | 0.3680 | 0.3826 | 0.1707 | 0.3880 | 1.5101 |
| ML SE [r1,r2,c] | 0.0069 | 0.0102 | 0.0551 | 0.0079 | 0.0103 | 0.0603 |

**ML test Ctrl vs. Selection (r1 and r2 are line-specific)**

c - control or selection specific

| | | | | | | |
|---|---|---|---|---|---|---|
| Line1[r1,r2,c]: | 0.1254 | 0.3762 | 0.3835 | 0.1682 | 0.3928 | 1.5068 |
| Line2[r1,r2,c]: | 0.1073 | 0.3754 | 0.3835 | 0.1606 | 0.3774 | 1.5068 |
| Line3[r1,r2,c]: | 0.1365 | 0.3525 | 0.3835 | 0.1830 | 0.3937 | 1.5068 |

c-global

| | | | | | | |
|---|---|---|---|---|---|---|
| Line1[r1,r2,c]: | 0.1247 | 0.3741 | 1.0785 | 0.1676 | 0.3915 | 1.0785 |
| Line2[r1,r2,c]: | 0.1061 | 0.3714 | 1.0785 | 0.1607 | 0.3777 | 1.0785 |
| Line3[r1,r2,c]: | 0.1368 | 0.3533 | 1.0785 | 0.1861 | 0.4002 | 1.0785 |

ML ratio (df=14 –13=1): 150.2249

***ML test Ctrl vs. Selection (r1, r2 and c are all line-specific)**

| | | | | | | |
|---|---|---|---|---|---|---|
| Line1[r1,r2,c]: | 0.1253 | 0.3760 | 0.3678 | 0.1667 | 0.3893 | 1.6027 |
| Line2[r1,r2,c]: | 0.1067 | 0.3733 | 0.2009 | 0.1600 | 0.3760 | 1.5514 |
| Line3[r1,r2,c]: | 0.1373 | 0.3547 | 0.5475 | 0.1853 | 0.3987 | 1.3895 |

ML ratio (df=18 –13=5): 159.3231

ML ratio for heterogeneity (control+selection; df = 5–1=4): 159.3231 – 150.2249
……………………………………………………………………………………………………

## markers 1-3-4 (*net-b-pk*)

| | CONTROL | | | HYPOXIA | | |
|---|---|---|---|---|---|---|
| **Lines (ML estimates)** | | | | | | |
| Line1 [r1,r2,c]: | 0.4000 | 0.0747 | 0.4018 | 0.3600 | 0.0827 | 1.6129 |
| Line2 [r1,r2,c]: | 0.4053 | 0.0560 | 0.4699 | 0.3813 | 0.0773 | 1.7634 |
| Line3 [r1,r2,c]: | 0.4040 | 0.0720 | 0.7792 | 0.3933 | 0.0800 | 1.6525 |
| Bailey test for 3 × 750 | | | | | | |
| Teta [r1,r2,c]: | 0.4033 | 0.0666 | 0.5248 | 0.3783 | 0.0799 | 1.6818 |
| SE [r1,r2,c]: | 0.0103 | 0.0052 | 0.0793 | 0.0102 | 0.0057 | 0.0926 |
| Chi2: | | 6.6372 | | | 2.7373 | |
| C Chi2: | | 4.0178 | | | 0.4848 | |

Bailey for 6 × 750:

| | | | |
|---|---|---|---|
| Teta: | 0.3978 | 0.0748 | 1.0127 |
| Chi2: | 104.5795 | | |

C chi2: 96.4385

**ML test Control vs. selection**
chi^2(ctrl + sel) - chi^2(ctrl) - chi^2(sel): 95.2050
C chi^2(ctrl + sel) - chi^2(ctrl) - chi^2(sel): **91.9359**

**ML test Ctrl Vs. Selection**

| | | | | | | |
|---|---|---|---|---|---|---|
| ML est [r1,r2,c] | 0.4031 | 0.0676 | 0.5549 | 0.3782 | 0.0800 | 1.6745 |
| ML SE [r1,r2,c] | 0.0103 | 0.0053 | 0.0819 | 0.0102 | 0.0057 | 0.0930 |

**ML test Ctrl vs. Selection (r1 and r2 are line-specific)**

c - control or selection specific

| | | | | | | |
|---|---|---|---|---|---|---|
| Line1[r1,r2,c]: | 0.4011 | 0.0749 | 0.5553 | 0.3588 | 0.0824 | 1.6770 |
| Line2[r1,r2,c]: | 0.4058 | 0.0561 | 0.5553 | 0.3832 | 0.0777 | 1.6770 |
| Line3[r1,r2,c]: | 0.4024 | 0.0717 | 0.5553 | 0.3927 | 0.0799 | 1.6770 |

c-global

| | | | | | | |
|---|---|---|---|---|---|---|
| Line1[r1,r2,c]: | 0.3976 | 0.0742 | 1.1376 | .3613 | 0.0830 | 1.1376 |
| Line2[r1,r2,c]: | 0.4037 | 0.0558 | 1.1376 | 0.3832 | 0.0777 | 1.1376 |
| Line3[r1,r2,c]: | 0.4028 | 0.0718 | 1.1376 | 0.3951 | 0.0803 | 1.1376 |

ML ratio (df=14 –13=1): 71.639

***ML test Ctrl vs. Selection (r1, r2 and c are all line-specific)**

| | | | | | | |
|---|---|---|---|---|---|---|
| Line1[r1,r2,c]: | 0.4000 | 0.0747 | 0.4018 | 0.3600 | 0.0827 | 1.6129 |
| Line2[r1,r2,c]: | 0.4053 | 0.0560 | 0.4699 | 0.3813 | 0.0773 | 1.7634 |
| Line3[r1,r2,c]: | 0.4040 | 0.0720 | 0.7792 | 0.3933 | 0.0800 | 1.6525 |

ML ratio (df=18 –13=5): 76.2650

ML ratio for heterogeneity (control+selection; df = 5–1=4): 76.2650 – 71.639
……………………………………………………………………………………………………

## markers 1-3-5 (*net-b-cn*)

| | CONTROL | | | HYPOXIA | | |
|---|---|---|---|---|---|---|
| **Lines (ML estimates)** | | | | | | |
| Line1 [r1,r2,c]: | 0.4000 | 0.0987 | 0.4054 | 0.3600 | 0.1027 | 1.5512 |
| Line2 [r1,r2,c]: | 0.4053 | 0.0800 | 0.3289 | 0.3813 | 0.0960 | 1.7482 |
| Line3 [r1,r2,c]: | 0.4040 | 0.0880 | 0.7501 | 0.3933 | 0.1027 | 1.4528 |
| Bailey test for 3 × 750 | | | | | | |
| Teta [r1,r2,c]: | 0.4033 | 0.0883 | 0.4571 | 0.3790 | 0.1005 | 1.5826 |
| SE [r1,r2,c]: | 0.0103 | 0.0060 | 0.0652 | 0.0102 | 0.0063 | 0.0827 |
| Chi2: | | 8.0340 | | | 4.4582 | |
| C Chi2: | | 6.4509 | | | 2.3896 | |

Bailey for 6 × 750:

| | | | |
|---|---|---|---|
| Teta: | 0.3993 | 0.0970 | 0.8877 |
| Chi2: | 130.7405 | | |
| C chi2: | 126.2274 | | |

**ML test Control vs. selection**

chi^2(ctrl + sel) - chi^2(ctrl) - chi^2(sel): 118.2483

C chi^2(ctrl + sel) - chi^2(ctrl) - chi^2(sel): **117.3869**

**ML test Ctrl Vs. Selection**

| | | | | | | |
|---|---|---|---|---|---|---|
| ML est [r1,r2,c] | 0.4031 | 0.0889 | 0.4961 | 0.3782 | 0.1004 | 1.5793 |
| ML SE [r1,r2,c] | 0.0103 | 0.0060 | 0.0682 | 0.0102 | 0.0063 | 0.0831 |

**ML test Ctrl vs. Selection (r1 and r2 are line-specific)**

c - control or selection specific

| | | | | | | |
|---|---|---|---|---|---|---|
| Line1[r1,r2,c]: | 0.4010 | 0.0989 | 0.4962 | 0.3594 | 0.1025 | 1.5795 |
| Line2[r1,r2,c]: | 0.4068 | 0.0803 | 0.4962 | 0.3851 | 0.0969 | 1.5795 |
| Line3[r1,r2,c]: | 0.4016 | 0.0875 | 0.4962 | 0.3901 | 0.1018 | 1.5795 |

c-global

| | | | | | | |
|---|---|---|---|---|---|---|
| Line1[r1,r2,c]: | 0.3991 | 0.0984 | 1.0462 | 0.3606 | 0.1028 | 1.0462 |
| Line2[r1,r2,c]: | 0.4045 | 0.0798 | 1.0462 | 0.3822 | 0.0962 | 1.0462 |
| Line3[r1,r2,c]: | 0.4036 | 0.0879 | 1.0462 | 0.3939 | 0.1028 | 1.0462 |

ML ratio (df=14 –13=1): 89.8168

***ML test Ctrl vs. Selection (r1, r2 and c are all line-specific)**

| | | | | | | |
|---|---|---|---|---|---|---|
| Line1[r1,r2,c]: | 0.4000 | 0.0987 | 0.4054 | 0.3600 | 0.1027 | 1.5512 |
| Line2[r1,r2,c]: | 0.4053 | 0.0800 | 0.3289 | 0.3813 | 0.0960 | 1.7482 |
| Line3[r1,r2,c]: | 0.4040 | 0.0880 | 0.7501 | 0.3933 | 0.1027 | 1.4528 |

ML ratio (df=18 –13=5): 98.8883

ML ratio for heterogeneity (control+selection; df = 5–1=4): 98.8883 – 89.8168

…………………………………………………………………………………………………………

## markers 2-3-4 (*dp-b-pk*)

| | CONTROL | | | HYPOXIA | | |
|---|---|---|---|---|---|---|
| **Lines (ML estimates)** | | | | | | |
| Line1 [r1,r2,c]: | 0.2960 | 0.0747 | 0.3016 | 0.3693 | 0.0827 | 1.0918 |
| Line2 [r1,r2,c]: | 0.3093 | 0.0560 | 0.3848 | 0.3707 | 0.0773 | 1.2559 |
| Line3 [r1,r2,c]: | 0.3093 | 0.0720 | 0.8981 | 0.3760 | 0.0800 | 1.2412 |
| Bailey test for 3 × 750 | | | | | | |
| Teta [r1,r2,c]: | 0.3050 | 0.0665 | 0.4550 | 0.3722 | 0.0800 | 1.1940 |
| SE [r1,r2,c]: | 0.0097 | 0.0052 | 0.0880 | 0.0102 | 0.0057 | 0.0952 |

| | | |
|---|---|---|
| Chi2: | 9.9352 | 0.8439 |
| C Chi2: | 7.0786 | 0.6180 |

Bailey for 6 × 750:

| | |
|---|---|
| Teta: | 0.3375 0.0718 0.8085 |
| Chi2: | 69.8066 |
| C chi2: | 40.3145 |

**ML test Control vs. selection**

chi^2(ctrl + sel) - chi^2(ctrl) - chi^2(sel): 59.0275

C chi^2(ctrl + sel) - chi^2(ctrl) - chi^2(sel): **32.6179**

**ML test Ctrl Vs. Selection**

| | | |
|---|---|---|
| ML est [r1,r2,c] | 0.3049 0.0676 0.5397 | 0.3720 0.0800 1.1947 |
| ML SE [r1,r2,c] | 0.0097 0.0053 0.0965 | 0.0102 0.0057 0.0953 |

**ML test Ctrl vs. Selection (r1 and r2 are line-specific)**

c - control or selection specific

| | | | | | | |
|---|---|---|---|---|---|---|
| Line1[r1,r2,c]: | 0.2969 | 0.0749 | 0.5412 | 0.3689 | 0.0826 | 1.1950 |
| Line2[r1,r2,c]: | 0.3098 | 0.0561 | 0.5412 | 0.3709 | 0.0774 | 1.1950 |
| Line3[r1,r2,c]: | 0.3079 | 0.0717 | 0.5412 | 0.3762 | 0.0800 | 1.1950 |

c-global

| | | | | | | |
|---|---|---|---|---|---|---|
| Line1[r1,r2,c]: | 0.2963 | 0.0747 | 0.9448 | 0.3692 | 0.0826 | 0.9448 |
| Line2[r1,r2,c]: | 0.3096 | 0.0560 | 0.9448 | 0.3704 | 0.0773 | 0.9448 |
| Line3[r1,r2,c]: | 0.3094 | 0.0720 | 0.9448 | 0.3757 | 0.0799 | 0.9448 |

ML ratio (df=14 –13=1): 20.4499

***ML test Ctrl vs. Selection (r1, r2 and c are all line-specific)**

| | | | | | | |
|---|---|---|---|---|---|---|
| Line1[r1,r2,c]: | 0.2960 | 0.0747 | 0.3016 | 0.3693 | 0.0827 | 1.0918 |
| Line2[r1,r2,c]: | 0.3093 | 0.0560 | 0.3848 | 0.3707 | 0.0773 | 1.2559 |
| Line3[r1,r2,c]: | 0.3093 | 0.0720 | 0.8981 | 0.3760 | 0.0800 | 1.2412 |

ML ratio: 28.7074

ML ratio for heterogeneity (control+selection; df = 5–1=4): 28.7074 – 20.4499

…………………………………………………………………………………………………

## markers 2-3-5 (*dp-b-cn*)

| | CONTROL | HYPOXIA |
|---|---|---|
| **Lines (ML estimates)** | | |
| Line1 [r1,r2,c]: | 0.2960 0.0987 0.3196 | 0.3693 0.1027 1.0901 |
| Line2 [r1,r2,c]: | 0.3093 0.0800 0.3233 | 0.3707 0.0960 1.2740 |

| | | | | | | |
|---|---|---|---|---|---|---|
| Line3 [r1,r2,c]: | 0.3093 | 0.0880 | 0.7837 | 0.3760 | 0.1027 | 1.0362 |

Bailey test for 3 × 750

| | | | | | | |
|---|---|---|---|---|---|---|
| Teta [r1,r2,c]: | 0.3051 | 0.0883 | 0.4158 | 0.3724 | 0.1004 | 1.1267 |
| SE [r1,r2,c]: | 0.0097 | 0.0060 | 0.0742 | 0.0102 | 0.0063 | 0.0833 |
| Chi2: | | 8.1542 | | | 1.7397 | |
| C Chi2: | | 6.3326 | | | 1.4438 | |

Bailey for 6 × 750:

| | | | |
|---|---|---|---|
| Teta: | 0.3378 | 0.0929 | 0.7406 |
| Chi2: | 75.7418 | | |
| C chi2: | 48.5911 | | |

**ML test Control vs. selection**

chi^2(ctrl + sel) - chi^2(ctrl) - chi^2(sel): 65.8479
C chi^2(ctrl + sel) - chi^2(ctrl) - chi^2(sel): **40.8147**

**ML test Ctrl Vs. Selection**

| | | | | | | |
|---|---|---|---|---|---|---|
| ML est [r1,r2,c] | 0.3049 | 0.0889 | 0.4756 | 0.3720 | 0.1004 | 1.1300 |
| ML SE [r1,r2,c] | 0.0097 | 0.0060 | 0.0796 | 0.0102 | 0.0063 | 0.0836 |

**ML test Ctrl vs. Selection (r1 and r2 are line-specific)**

c - control or selection specific

| | | | | | | |
|---|---|---|---|---|---|---|
| Line1[r1,r2,c]: | 0.2969 | 0.0990 | 0.4764 | 0.3692 | 0.1026 | 1.1293 |
| Line2[r1,r2,c]: | 0.3101 | 0.0802 | 0.4764 | 0.3711 | 0.0961 | 1.1293 |
| Line3[r1,r2,c]: | 0.3077 | 0.0875 | 0.4764 | 0.3757 | 0.1026 | 1.1293 |

c-global

| | | | | | | |
|---|---|---|---|---|---|---|
| Line1[r1,r2,c]: | 0.2969 | 0.0990 | 0.8702 | 0.3687 | 0.1025 | 0.8702 |
| Line2[r1,r2,c]: | 0.3101 | 0.0802 | 0.8702 | 0.3695 | 0.0957 | 0.8702 |
| Line3[r1,r2,c]: | 0.3095 | 0.0880 | 0.8702 | 0.3755 | 0.1025 | 0.8702 |

ML ratio (df=14 –13=1): 28.1900

***ML test Ctrl vs. Selection (r1, r2 and c are all line-specific)**

| | | | | | | |
|---|---|---|---|---|---|---|
| Line1[r1,r2,c]: | 0.2960 | 0.0987 | 0.3196 | 0.3693 | 0.1027 | 1.0901 |
| Line2[r1,r2,c]: | 0.3093 | 0.0800 | 0.3233 | 0.3707 | 0.0960 | 1.2740 |
| Line3[r1,r2,c]: | 0.3093 | 0.0880 | 0.7837 | 0.3760 | 0.1027 | 1.0362 |

ML ratio (df=18 –13=5): 36.7716

ML ratio for heterogeneity (control+selection; df = 5–1=4): 36.7716 – 28.1900
……………………………………………………………………………………………………

## markers 1-4-5 (*net-pk-cn*)

**CONTROL** **HYPOXIA**

**Lines (ML estimates)**

| | | |
|---|---|---|
| Line1 [r1,r2,c]: | 0.4507 0.0240 0.3699 | 0.3467 0.0227 1.3608 |
| Line2 [r1,r2,c]: | 0.4401 0.0240 0.0000 | 0.3547 0.0187 1.8153 |
| Line3 [r1,r2,c]: | 0.4307 0.0187 0.6615 | 0.3693 0.0227 0.7958 |

Bailey test for 3 × 750

| | | |
|---|---|---|
| Teta [r1,r2,c]: | 0.4400 0.0219 0.0000 | 0.3582 0.0213 1.2498 |
| SE [r1,r2,c]: | 0.0105 0.0031 0.0004 | 0.0101 0.0030 0.1904 |
| Chi2: | 10.6824 | 6.1749 |
| C Chi2: | 9.3116 | 4.9552 |

Bailey for 6 × 750:

Teta: 0.3991 0.0224 0.0000
Chi2: 89.7517
C chi2: 57.8865

**ML test Control vs. selection**

chi^2(ctrl + sel) - chi^2(ctrl) - chi^2(sel): 72.8944
C chi^2(ctrl + sel) - chi^2(ctrl) - chi^2(sel): **43.6197**

**ML test Ctrl Vs. Selection**

| | | |
|---|---|---|
| ML est [r1,r2,c] | 0.4405 0.0222 0.3180 | 0.3568 0.0213 1.2847 |
| ML SE [r1,r2,c] | 0.0105 0.0031 0.1109 | 0.0101 0.0030 0.1993 |

**ML test Ctrl vs. Selection (r1 and r2 are line-specific)**

c - control or selection specific

| | | | | | | |
|---|---|---|---|---|---|---|
| Line1[r1,r2,c]: | 0.4505 | 0.0240 | 0.3168 | 0.3468 | 0.0227 | 1.2829 |
| Line2[r1,r2,c]: | 0.4412 | 0.0241 | 0.3168 | 0.3553 | 0.0187 | 1.2829 |
| Line3[r1,r2,c]: | 0.4297 | 0.0187 | 0.3168 | 0.3685 | 0.0226 | 1.2829 |

c-global

| | | | | | | |
|---|---|---|---|---|---|---|
| Line1[r1,r2,c]: | 0.4514 | 0.0240 | 0.7184 | 0.3460 | 0.0226 | 0.7184 |
| Line2[r1,r2,c]: | 0.4414 | 0.0241 | 0.7184 | 0.3537 | 0.0186 | 0.7184 |
| Line3[r1,r2,c]: | 0.4307 | 0.0187 | 0.7184 | 0.3692 | 0.0227 | 0.7184 |

ML ratio (df=14 –13=1): 17.6626

***ML test Ctrl vs. Selection (r1, r2 and c are all line-specific)**

| | | | | | | |
|---|---|---|---|---|---|---|
| Line1[r1,r2,c]: | 0.4507 | 0.0240 | 0.3699 | 0.3467 | 0.0227 | 1.3608 |
| Line2[r1,r2,c]: | 0.4401 | 0.0240 | 0.0000 | 0.3547 | 0.0187 | 1.8153 |
| Line3[r1,r2,c]: | 0.4307 | 0.0187 | 0.6615 | 0.3693 | 0.0227 | 0.7958 |

ML ratio (df=18 –13=5): 29.7333

ML ratio for heterogeneity (control+selection; df = 5–1=4): 29.7333 – 17.6626
……………………………………………………………………………………………………

**markers 2-4-5 (*dp-pk-cn*)**

| | CONTROL | | | HYPOXIA | | |
|---|---|---|---|---|---|---|
| **Lines (ML estimates)** | | | | | | |
| Line1 [r1,r2,c]: | 0.3573 | 0.0240 | 0.3116 | 0.3854 | 0.0227 | 1.0661 |
| Line2 [r1,r2,c]: | 0.3520 | 0.0240 | 0.1577 | 0.3759 | 0.0187 | 1.3347 |
| Line3 [r1,r2,c]: | 0.3413 | 0.0187 | 0.4206 | 0.3813 | 0.0227 | 0.3090 |

Bailey test for 3 × 750

| | | | | | | |
|---|---|---|---|---|---|---|
| Teta [r1,r2,c]: | 0.3502 | 0.0219 | 0.2467 | 0.3819 | 0.0212 | 0.6906 |
| SE [r1,r2,c]: | 0.0101 | 0.0031 | 0.1121 | 0.0102 | 0.0030 | 0.1529 |
| Chi2: | | 2.0878 | | | 8.8543 | |
| C Chi2: | | 0.8435 | | | 8.3515 | |

Bailey for 6 × 750:

| | | | |
|---|---|---|---|
| Teta: | 0.3660 | 0.0214 | 0.4043 |
| Chi2: | 21.1763 | | |
| C chi2: | 14.6904 | | |

**ML test Control vs. selection**

chi^2(ctrl + sel) - chi^2(ctrl) - chi^2(sel): 10.2342

C chi^2(ctrl + sel) - chi^2(ctrl) - chi^2(sel): **5.4955**

**ML test Ctrl Vs. Selection**

| | | | | | | |
|---|---|---|---|---|---|---|
| ML est [r1,r2,c] | 0.3503 | 0.0222 | 0.2853 | 0.3808 | 0.0213 | 0.8778 |
| ML SE [r1,r2,c] | 0.0101 | 0.0031 | 0.1206 | 0.0102 | 0.0030 | 0.1770 |

**ML test Ctrl vs. Selection (r1 and r2 are line-specific)**

c - control or selection specific

| | | | | | | |
|---|---|---|---|---|---|---|
| Line1[r1,r2,c]: | 0.3572 | 0.0240 | 0.2860 | 0.3852 | 0.0227 | 0.8768 |
| Line2[r1,r2,c]: | 0.3523 | 0.0240 | 0.2860 | 0.3757 | 0.0187 | 0.8768 |
| Line3[r1,r2,c]: | 0.3411 | 0.0187 | 0.2860 | 0.3817 | 0.0227 | 0.8768 |

c-global

| | | | | | | |
|---|---|---|---|---|---|---|
| Line1[r1,r2,c]: | 0.3578 | 0.0240 | 0.5895 | 0.3844 | 0.0226 | 0.5895 |
| Line2[r1,r2,c]: | 0.3527 | 0.0241 | 0.5895 | 0.3749 | 0.0186 | 0.5895 |
| Line3[r1,r2,c]: | 0.3415 | 0.0187 | 0.5895 | 0.3818 | 0.0227 | 0.5895 |

ML ratio (df=14 –13=1): 7.1584

***ML test Ctrl vs. Selection (r1, r2 and c are all line-specific)**

| | | | | | | |
|---|---|---|---|---|---|---|
| Line1[r1,r2,c]: | 0.3573 | 0.0240 | 0.3116 | 0.3854 | 0.0227 | 1.0661 |
| Line2[r1,r2,c]: | 0.3520 | 0.0240 | 0.1577 | 0.3759 | 0.0187 | 1.3347 |
| Line3[r1,r2,c]: | 0.3413 | 0.0187 | 0.4206 | 0.3813 | 0.0227 | 0.3090 |

ML ratio (df=18 –13=5): 14.4691

ML ratio for heterogeneity (control+selection; df = 5–1=4): 14.4691 – 7.1584
…………………………………………………………………………………………………

## **markers 3-4-5 (*b-pk-cn*)**

| | CONTROL | | | HYPOXIA | | |
|---|---|---|---|---|---|---|
| **Lines (ML estimates)** | | | | | | |
| Line1 [r1,r2,c]: | 0.0747 | 0.0240 | 0.0000 | 0.0826 | 0.0227 | 0.7116 |
| Line2 [r1,r2,c]: | 0.0560 | 0.0240 | 0.0000 | 0.0773 | 0.0187 | 0.0000 |
| Line3 [r1,r2,c]: | 0.0721 | 0.0187 | 0.9901 | 0.0800 | 0.0227 | 0.0000 |
| Bailey test for 3 × 750 | | | | | | |
| Teta [r1,r2,c]: | 0.0666 | 0.0219 | 0.0000 | 0.0799 | 0.0212 | 0.0000 |
| SE [r1,r2,c]: | 0.0053 | 0.0031 | 0.0012 | 0.0057 | 0.0030 | 0.0003 |
| Chi2: | | 4.4443 | | | 1.6362 | |
| C Chi2: | | 1.0954 | | | 1.0798 | |

Bailey for 6 × 750:

| | | | |
|---|---|---|---|
| Teta: | 0.0727 | 0.0216 | 0.0000 |
| Chi2: | 9.0158 | | |
| C chi2: | 2.1752 | | |

**ML test Control vs. selection**

chi^2(ctrl + sel) - chi^2(ctrl) - chi^2(sel): 2.9353
C chi^2(ctrl + sel) - chi^2(ctrl) - chi^2(sel): **0.0000**

**ML test Ctrl Vs. Selection**

| | CONTROL | | | HYPOXIA | | |
|---|---|---|---|---|---|---|
| ML est [r1,r2,c] | 0.0676 | 0.0222 | 0.3012 | 0.0800 | 0.0213 | 0.2624 |
| ML SE [r1,r2,c] | 0.0053 | 0.0031 | 0.2945 | 0.0057 | 0.0030 | 0.2579 |

**ML test Ctrl vs. Selection (r1 and r2 are line-specific)**

c - control or selection specific

| | | | | | | |
|---|---|---|---|---|---|---|
| Line1[r1,r2,c]: | 0.0747 | 0.0240 | 0.2992 | 0.0826 | 0.0226 | 0.2694 |
| Line2[r1,r2,c]: | 0.0560 | 0.0240 | 0.2992 | 0.0773 | 0.0187 | 0.2694 |
| Line3[r1,r2,c]: | 0.0719 | 0.0187 | 0.2992 | 0.0800 | 0.0227 | 0.2694 |

c-global

| | | | | | | |
|---|---|---|---|---|---|---|
| Line1[r1,r2,c]: | 0.0747 | 0.0240 | 0.2811 | 0.0826 | 0.0227 | 0.2811 |
| Line2[r1,r2,c]: | 0.0560 | 0.0240 | 0.2811 | 0.0774 | 0.0187 | 0.2811 |
| Line3[r1,r2,c]: | 0.0720 | 0.0186 | 0.2811 | 0.0800 | 0.0227 | 0.2811 |

ML ratio (df=14 –13=1): 0.0083

***ML test Ctrl vs. Selection (r1, r2 and c are all line-specific)**

| | | | | | | |
|---|---|---|---|---|---|---|
| Line1[r1,r2,c]: | 0.0747 | 0.0240 | 0.0000 | 0.0826 | 0.0227 | 0.7116 |
| Line2[r1,r2,c]: | 0.0560 | 0.0240 | 0.0000 | 0.0773 | 0.0187 | 0.0000 |
| Line3[r1,r2,c]: | .0721 | 0.0187 | 0.9901 | 0.0800 | 0.0227 | 0.0000 |

ML ratio (df=18 –13=5):     4.5463

ML ratio for heterogeneity (control+selection; df = 5–1=4): 4.5463 – 0.0083
……………………………………………………………………………………………………

# Control and hyperoxia lines

## markers 1-2-3 (*net-dp-b*)

| | CONTROL | | | HYPEROXIA | | |
|---|---|---|---|---|---|---|
| **Lines (ML estimates)** | | | | | | |
| Line1 [r1,r2,c]: | 0.1253 | 0.2960 | 0.2875 | 0.1547 | 0.3493 | 1.0611 |
| Line2 [r1,r2,c]: | 0.1067 | 0.3093 | 0.1616 | 0.1440 | 0.3747 | 0.8897 |
| Line3 [r1,r2,c]: | 0.1373 | 0.3093 | 0.5022 | 0.1373 | 0.3613 | 1.1016 |
| Bailey test for 3 × 750 | | | | | | |
| Teta [r1,r2,c]: | 0.1218 | 0.3042 | 0.2784 | 0.1453 | 0.3620 | 1.0096 |
| SE [r1,r2,c]: | 0.0069 | 0.0097 | 0.0529 | 0.0074 | 0.0101 | 0.0679 |
| Chi2: | | 9.8633 | | | 3.8755 | |
| C Chi2: | | 6.3762 | | | 1.8825 | |

Bailey for 6 × 750:

| | | | |
|---|---|---|---|
| Teta: | 0.1314 | 0.3317 | 0.5610 |
| Chi2: | 108.0404 | | |
| C chi2: | 80.6612 | | |

**ML test Control vs. selection**

chi^2(ctrl + sel) - chi^2(ctrl) - chi^2(sel): 94.3015

C chi^2(ctrl + sel) - chi^2(ctrl) - chi^2(sel): **72.4025**

**ML test Ctrl Vs. Selection**

| | | | | | | |
|---|---|---|---|---|---|---|
| ML est [r1,r2,c] | 0.1231 | 0.3049 | 0.3315 | 0.1453 | 0.3618 | 1.0144 |
| ML SE [r1,r2,c] | 0.0069 | 0.0097 | 0.0579 | 0.0074 | 0.0101 | 0.0681 |

**ML test Ctrl vs. Selection (r1 and r2 are line-specific)**

c - control or selection specific

| | | | | | | |
|---|---|---|---|---|---|---|
| Line1[r1,r2,c]: | 0.1255 | 0.2964 | 0.3323 | 0.1547 | 0.3494 | 1.0140 |
| Line2[r1,r2,c]: | 0.1071 | 0.3107 | 0.3323 | 0.1440 | 0.3746 | 1.0140 |
| Line3[r1,r2,c]: | 0.1366 | 0.3076 | 0.3323 | 0.1373 | 0.3614 | 1.0140 |

c-global

| | | | | | | |
|---|---|---|---|---|---|---|
| Line1[r1,r2,c]: | 0.1261 | 0.2978 | 0.7426 | .1536 | 0.3470 | 0.7426 |
| Line2[r1,r2,c]: | 0.1074 | 0.3115 | 0.7426 | 0.1435 | 0.3735 | 0.7426 |
| Line3[r1,r2,c]: | 0.1379 | 0.3105 | 0.7426 | 0.1364 | 0.3588 | 0.7426 |

ML ratio (df=14 –13=1): 49.4600

***ML test Ctrl vs. Selection (r1, r2 and c are all line-specific)**

| | | | | | | |
|---|---|---|---|---|---|---|
| Line1[r1,r2,c]: | 0.1253 | 0.2960 | 0.2875 | 0.1547 | 0.3493 | 1.0611 |
| Line2[r1,r2,c]: | 0.1067 | 0.3093 | 0.1616 | 0.1440 | 0.3747 | 0.8897 |
| Line3[r1,r2,c]: | 0.1373 | 0.3093 | 0.5022 | 0.1373 | 0.3613 | 1.1016 |

ML ratio (df=18 –13=5): 57.4485

ML ratio for heterogeneity (control+selection; df = 5–1=4): 57.4485 – 49.4600
…………………………………………………………………………………………………………

## markers 1-2-4 (*net-dp-pk*)

| | CONTROL | | | HYPEROXIA | | |
|---|---|---|---|---|---|---|
| **Lines (ML estimates)** | | | | | | |
| Line1 [r1,r2,c]: | 0.1253 | 0.3573 | 0.3573 | 0.1547 | 0.3640 | 1.2315 |
| Line2 [r1,r2,c]: | 0.1067 | 0.3520 | 0.2486 | 0.1440 | 0.3747 | 1.0132 |
| Line3 [r1,r2,c]: | 0.1373 | 0.3413 | 0.5120 | 0.1373 | 0.3813 | 1.3748 |
| Bailey test for 3 × 750 | | | | | | |
| Teta [r1,r2,c]: | 0.1220 | 0.3498 | 0.3571 | 0.1453 | 0.3745 | 1.2027 |
| SE [r1,r2,c]: | 0.0069 | 0.0101 | 0.0547 | 0.0074 | 0.0102 | 0.0676 |
| Chi2: | | 7.3222 | | | 6.4403 | |
| C Chi2: | | 3.7141 | | | 4.8497 | |

Bailey for 6 × 750:

| | | | |
|---|---|---|---|
| Teta: | 0.1332 | 0.3643 | 0.7006 |
| Chi2: | 117.5393 | | |
| C chi2: | 103.5705 | | |

**ML test Control vs. selection**

chi^2(ctrl + sel) - chi^2(ctrl) - chi^2(sel): 103.7769

C chi^2(ctrl + sel) - chi^2(ctrl) - chi^2(sel): **95.0066**

**ML test Ctrl Vs. Selection**

| | | | | | | |
|---|---|---|---|---|---|---|
| ML est [r1,r2,c] | 0.1231 | 0.3502 | 0.3814 | 0.1453 | 0.3733 | 1.2041 |
| ML SE [r1,r2,c] | 0.0069 | 0.0101 | 0.0566 | 0.0074 | 0.0102 | 0.0679 |

**ML test Ctrl vs. Selection (r1 and r2 are line-specific)**

c - control or selection specific

| | | | | | | |
|---|---|---|---|---|---|---|
| Line1[r1,r2,c]: | 0.1254 | 0.3576 | 0.3821 | 0.1548 | 0.3642 | 1.2059 |
| Line2[r1,r2,c]: | 0.1071 | 0.3533 | 0.3821 | 0.1433 | 0.3730 | 1.2059 |
| Line3[r1,r2,c]: | 0.1367 | 0.3398 | 0.3821 | 0.1379 | 0.3828 | 1.2059 |

c-global

| | | | | | | |
|---|---|---|---|---|---|---|
| Line1[r1,r2,c]: | 0.1260 | 0.3593 | 0.8501 | 0.1538 | 0.3620 | 0.8501 |
| Line2[r1,r2,c]: | 0.1072 | 0.3539 | 0.8501 | 0.1437 | 0.3738 | 0.8501 |
| Line3[r1,r2,c]: | 0.1379 | 0.3426 | 0.8501 | 0.1364 | 0.3787 | 0.8501 |

ML ratio (df=14 –13=1): 74.9737

***ML test Ctrl vs. Selection (r1, r2 and c are all line-specific)**

| | | | | | | |
|---|---|---|---|---|---|---|
| Line1[r1,r2,c]: | 0.1253 | 0.3573 | 0.3573 | 0.1547 | 0.3640 | 1.2315 |
| Line2[r1,r2,c]: | 0.1067 | 0.3520 | 0.2486 | 0.1440 | 0.3747 | 1.0132 |
| Line3[r1,r2,c]: | 0.1373 | 0.3413 | 0.5120 | 0.1373 | 0.3813 | 1.3748 |

ML ratio (df=18 –13=5): 83.3336

ML ratio for heterogeneity (control+selection; df = 5–1=4): 83.3336 – 74.9737
…………………………………………………………………………………………………………

## markers 1-2-5 (*net-dp-cn*)

| | CONTROL | | | HYPEROXIA | | |
|---|---|---|---|---|---|---|
| **Lines (ML estimates)** | | | | | | |
| Line1 [r1,r2,c]: | 0.1253 | 0.3760 | 0.3678 | 0.1547 | 0.3813 | 1.2434 |
| Line2 [r1,r2,c]: | 0.1067 | 0.3733 | 0.2009 | 0.1440 | 0.3760 | 1.0343 |
| Line3 [r1,r2,c]: | 0.1373 | 0.3547 | 0.5475 | 0.1373 | 0.4053 | 1.3413 |
| Bailey test for 3 × 750 | | | | | | |
| Teta [r1,r2,c]: | 0.1220 | 0.3672 | 0.3373 | 0.1450 | 0.3884 | 1.2125 |
| SE [r1,r2,c]: | 0.0069 | 0.0102 | 0.0516 | 0.0074 | 0.0102 | 0.0653 |
| Chi2: | | 11.1346 | | | 6.6594 | |
| C Chi2: | | 7.2486 | | | 3.7876 | |

Bailey for 6 × 750:

| | | | |
|---|---|---|---|
| Teta: | 0.1333 | 0.3807 | 0.6817 |
| Chi2: | 136.6205 | | |
| C chi2: | 121.3689 | | |

**ML test Control vs. selection**
chi^2(ctrl + sel) - chi^2(ctrl) - chi^2(sel): 118.8265
C chi^2(ctrl + sel) - chi^2(ctrl) - chi^2(sel): **110.3328**

**ML test Ctrl Vs. Selection**

| | | | | | | |
|---|---|---|---|---|---|---|
| ML est [r1,r2,c] | 0.1231 | 0.3680 | 0.3826 | 0.1453 | 0.3876 | 1.2073 |
| ML SE [r1,r2,c] | 0.0069 | 0.0102 | 0.0551 | 0.0074 | 0.0103 | 0.0657 |

**ML test Ctrl vs. Selection (r1 and r2 are line-specific)**

c - control or selection specific

| | | | | | | |
|---|---|---|---|---|---|---|
| Line1[r1,r2,c]: | 0.1254 | 0.3762 | 0.3835 | 0.1548 | 0.3817 | 1.2117 |

| | | | | | | |
|---|---|---|---|---|---|---|
| Line2[r1,r2,c]: | 0.1073 | 0.3754 | 0.3835 | 0.1434 | 0.3744 | 1.2117 |
| Line3[r1,r2,c]: | 0.1365 | 0.3525 | 0.3835 | 0.1378 | 0.4068 | 1.2117 |

c-global

| | | | | | | |
|---|---|---|---|---|---|---|
| Line1[r1,r2,c]: | 0.1260 | 0.3781 | 0.8503 | 0.1537 | 0.3791 | 0.8503 |
| Line2[r1,r2,c]: | 0.1073 | 0.3757 | 0.8503 | 0.1436 | 0.3751 | 0.8503 |
| Line3[r1,r2,c]: | 0.1378 | 0.3560 | 0.8503 | 0.1364 | 0.4025 | 0.8503 |

ML ratio (df=14 –13=1): 80.5029

**<u>*ML test Ctrl vs. Selection (r1, r2 and c are all line-specific)</u>**

| | | | | | | |
|---|---|---|---|---|---|---|
| Line1[r1,r2,c]: | 0.1253 | 0.3760 | 0.3678 | 0.1547 | 0.3813 | 1.2434 |
| Line2[r1,r2,c]: | 0.1067 | 0.3733 | 0.2009 | 0.1440 | 0.3760 | 1.0343 |
| Line3[r1,r2,c]: | 0.1373 | 0.3547 | 0.5475 | 0.1373 | 0.4053 | 1.3413 |

ML ratio (df=18 –13=5): 90.8503

ML ratio for heterogeneity (control+selection; df = 5–1=4): 90.8503 – 80.5029

…………………………………………………………………………………………………………

## markers 1-3-4 (*net-b-pk*)

| | CONTROL | | | HYPEROXIA | | |
|---|---|---|---|---|---|---|
| **Lines (ML estimates)** | | | | | | |
| Line1 [r1,r2,c]: | 0.4000 | 0.0747 | 0.4018 | 0.3893 | 0.0707 | 1.4540 |
| Line2 [r1,r2,c]: | 0.4053 | 0.0560 | 0.4699 | 0.4227 | 0.0560 | 1.4646 |
| Line3 [r1,r2,c]: | 0.4040 | 0.0720 | 0.7792 | 0.3893 | 0.0760 | 1.5321 |
| Bailey test for 3 × 750 | | | | | | |
| Teta [r1,r2,c]: | 0.4033 | 0.0666 | 0.5248 | 0.4000 | 0.0665 | 1.4862 |
| SE [r1,r2,c]: | 0.0103 | 0.0052 | 0.0793 | 0.0103 | 0.0052 | 0.0971 |
| Chi2: | | 6.6372 | | | 5.7033 | |
| C Chi2: | | 4.0178 | | | 0.1343 | |

Bailey for 6 × 750:

| | | | |
|---|---|---|---|
| Teta: | 0.4067 | 0.0673 | 0.9103 |
| Chi2: | 71.1752 | | |
| C chi2: | 63.5300 | | |

**<u>ML test Control vs. selection</u>**
chi^2(ctrl + sel) - chi^2(ctrl) - chi^2(sel): 58.8346
C chi^2(ctrl + sel) - chi^2(ctrl) - chi^2(sel): **<u>59.3779</u>**

**<u>ML test Ctrl Vs. Selection</u>**

| | | |
|---|---|---|
| ML est [r1,r2,c] | 0.4031 0.0676 0.5549 | 0.4004 0.0676 1.4784 |
| ML SE [r1,r2,c] | 0.0103 0.0053 0.0819 | 0.0103 0.0053 0.0969 |

**ML test Ctrl vs. Selection (r1 and r2 are line-specific)**

c - control or selection specific

| | | | | | | |
|---|---|---|---|---|---|---|
| Line1[r1,r2,c]: | 0.4011 | 0.0749 | 0.5553 | 0.3889 | 0.0706 | 1.4851 |
| Line2[r1,r2,c]: | 0.4058 | 0.0561 | 0.5553 | 0.4224 | 0.0560 | 1.4851 |
| Line3[r1,r2,c]: | 0.4024 | 0.0717 | 0.5553 | 0.3900 | 0.0761 | 1.4851 |

c-global

| | | | | | | |
|---|---|---|---|---|---|---|
| Line1[r1,r2,c]: | 0.3998 | 0.0746 | 1.0169 | 0.3895 | 0.0707 | 1.0169 |
| Line2[r1,r2,c]: | 0.4052 | 0.0560 | 1.0169 | 0.4228 | 0.0560 | 1.0169 |
| Line3[r1,r2,c]: | 0.4039 | 0.0720 | 1.0169 | 0.3895 | 0.0760 | 1.0169 |

ML ratio (df=14 –13=1): 48.1979

***ML test Ctrl vs. Selection (r1, r2 and c are all line-specific)**

| | | | | | | |
|---|---|---|---|---|---|---|
| Line1[r1,r2,c]: | 0.4000 | 0.0747 | 0.4018 | 0.3893 | 0.0707 | 1.4540 |
| Line2[r1,r2,c]: | 0.4053 | 0.0560 | 0.4699 | 0.4227 | 0.0560 | 1.4646 |
| Line3[r1,r2,c]: | 0.4040 | 0.0720 | 0.7792 | 0.3893 | 0.0760 | 1.5321 |

ML ratio (df=18 –13=5): 52.4977

ML ratio for heterogeneity (control+selection; df = 5–1=4): 52.4977 – 48.1979
……………………………………………………………………………………………………

**markers 1-3-5 (*net-b-cn*)**

| | CONTROL | HYPEROXIA |
|---|---|---|
| (ML) | | |
| Line1 [r1,r2,c]: | 0.4000 0.0987 0.4054 | 0.3893 0.0907 1.2842 |
| Line2 [r1,r2,c]: | 0.4053 0.0800 0.3289 | 0.4227 0.0680 1.4382 |
| Line3 [r1,r2,c]: | 0.4040 0.0880 0.7501 | 0.3893 0.1000 1.2329 |
| Bailey test for 3 × 750 | | |
| Teta [r1,r2,c]: | 0.4033 0.0883 0.4571 | 0.4009 0.0840 1.3140 |
| SE [r1,r2,c]: | 0.0103 0.0060 0.0652 | 0.0103 0.0058 0.0856 |
| Chi2: | 8.0340 | 9.7319 |
| C Chi2: | 6.4509 | 1.0069 |

Bailey for 6 × 750:

| | |
|---|---|
| Teta: | 0.4067 0.0870 0.7708 |
| Chi2: | 81.4098 |
| C chi2: | 70.8390 |

**ML test Control vs. selection**
chi^2(ctrl + sel) - chi^2(ctrl) - chi^2(sel): 63.6439

C chi^2(ctrl + sel) - chi^2(ctrl) - chi^2(sel): **63.3811**

**ML test Ctrl Vs. Selection**

| | | | | | | |
|---|---|---|---|---|---|---|
| ML est [r1,r2,c] | 0.4031 | 0.0889 | 0.4961 | 0.4004 | 0.0862 | 1.3001 |
| ML SE [r1,r2,c] | 0.0103 | 0.0060 | 0.0682 | 0.0103 | 0.0059 | 0.0857 |

**ML test Ctrl vs. Selection (r1 and r2 are line-specific)**

c - control or selection specific

| | | | | | | |
|---|---|---|---|---|---|---|
| Line1[r1,r2,c]: | 0.4010 | 0.0989 | 0.4962 | 0.3891 | 0.0906 | 1.3101 |
| Line2[r1,r2,c]: | 0.4068 | 0.0803 | 0.4962 | 0.4238 | 0.0682 | 1.3101 |
| Line3[r1,r2,c]: | 0.4016 | 0.0875 | 0.4962 | 0.3885 | 0.0998 | 1.3101 |

c-global

| | | | | | | |
|---|---|---|---|---|---|---|
| Line1[r1,r2,c]: | 0.4014 | 0.0990 | 0.8915 | 0.3884 | 0.0904 | 0.8915 |
| Line2[r1,r2,c]: | 0.4067 | 0.0803 | 0.8915 | 0.4214 | 0.0678 | 0.8915 |
| Line3[r1,r2,c]: | 0.4044 | 0.0881 | 0.8915 | 0.3884 | 0.0998 | 0.8915 |

ML ratio (df=14 –13=1): 50.7184

***ML test Ctrl vs. Selection (r1, r2 and c are all line-specific)**

| | | | | | | |
|---|---|---|---|---|---|---|
| Line1[r1,r2,c]: | 0.4000 | 0.0987 | 0.4054 | 0.3893 | 0.0907 | 1.2842 |
| Line2[r1,r2,c]: | 0.4053 | 0.0800 | 0.3289 | 0.4227 | 0.0680 | 1.4382 |
| Line3[r1,r2,c]: | 0.4040 | 0.0880 | 0.7501 | 0.3893 | 0.1000 | 1.2329 |

ML ratio (df=18 –13=5): 58.5178

ML ratio for heterogeneity (control+selection; df = 5–1=4): 58.5178 – 50.7184

…………………………………………………………………………………………………………

## markers 2-3-4 (*dp-b-pk*)

| | CONTROL | | | HYPEROXIA | | |
|---|---|---|---|---|---|---|
| **Lines (ML estimates)** | | | | | | |
| Line1 [r1,r2,c]: | 0.2960 | 0.0747 | 0.3016 | 0.3493 | 0.0707 | 1.1340 |
| Line2 [r1,r2,c]: | 0.3093 | 0.0560 | 0.3848 | 0.3747 | 0.0560 | 1.3349 |
| Line3 [r1,r2,c]: | 0.3093 | 0.0720 | 0.8981 | 0.3613 | 0.0760 | 1.0196 |
| Bailey test for 3 × 750 | | | | | | |
| Teta [r1,r2,c]: | 0.3050 | 0.0665 | 0.4550 | 0.3623 | 0.0664 | 1.1467 |
| SE [r1,r2,c]: | 0.0097 | 0.0052 | 0.0880 | 0.0101 | 0.0052 | 0.1061 |
| Chi2: | | 9.9352 | | | 5.4132 | |
| C Chi2: | | 7.0786 | | | 1.4526 | |

Bailey for 6 × 750:

| | | | |
|---|---|---|---|
| Teta: | 0.3334 | 0.0658 | 0.7453 |
| Chi2: | 57.3653 | | |

C chi2: 33.7446

**ML test Control vs. selection**
chi^2(ctrl + sel) - chi^2(ctrl) - chi^2(sel): 42.0169
C chi^2(ctrl + sel) - chi^2(ctrl) - chi^2(sel): **25.2133**

**ML test Ctrl Vs. Selection**

| | | | | | | |
|---|---|---|---|---|---|---|
| ML est [r1,r2,c] | 0.3049 | 0.0676 | 0.5397 | 0.3618 | 0.0676 | 1.1457 |
| ML SE [r1,r2,c] | 0.0097 | 0.0053 | 0.0965 | 0.0101 | 0.0053 | 0.1065 |

**ML test Ctrl vs. Selection (r1 and r2 are line-specific)**

c - control or selection specific

| | | | | | | |
|---|---|---|---|---|---|---|
| Line1[r1,r2,c]: | 0.2969 | 0.0749 | 0.5411 | 0.3493 | 0.0707 | 1.1491 |
| Line2[r1,r2,c]: | 0.3098 | 0.0561 | 0.5411 | 0.3751 | 0.0561 | 1.1491 |
| Line3[r1,r2,c]: | 0.3079 | 0.0717 | 0.5411 | 0.3610 | 0.0759 | 1.1491 |

c-global

| | | | | | | |
|---|---|---|---|---|---|---|
| Line1[r1,r2,c]: | 0.2967 | 0.0748 | 0.8815 | 0.3489 | 0.0706 | 0.8815 |
| Line2[r1,r2,c]: | 0.3098 | 0.0561 | 0.8815 | 0.3740 | 0.0559 | 0.8815 |
| Line3[r1,r2,c]: | 0.3093 | 0.0720 | 0.8815 | 0.3611 | 0.0759 | 0.8815 |

ML ratio (df=14 –13=1): 16.4166

***ML test Ctrl vs. Selection (r1, r2 and c are all line-specific)**

| | | | | | | |
|---|---|---|---|---|---|---|
| Line1[r1,r2,c]: | 0.2960 | 0.0747 | 0.3016 | 0.3493 | 0.0707 | 1.1340 |
| Line2[r1,r2,c]: | 0.3093 | 0.0560 | 0.3848 | 0.3747 | 0.0560 | 1.3349 |
| Line3[r1,r2,c]: | 0.3093 | 0.0720 | 0.8981 | 0.3613 | 0.0760 | 1.0196 |

ML ratio (df=18 –13=5): 25.5031

ML ratio for heterogeneity (control+selection; df = 5–1=4): 25.5031 – 16.4166
……………………………………………………………………………………………………

## markers 2-3-5 (*dp-b-cn*)

| | CONTROL | | | HYPEROXIA | | |
|---|---|---|---|---|---|---|
| **Lines (ML estimates)** | | | | | | |
| Line1 [r1,r2,c]: | 0.2960 | 0.0987 | 0.3196 | 0.3493 | 0.0907 | 0.9261 |
| Line2 [r1,r2,c]: | 0.3093 | 0.0800 | 0.3233 | 0.3747 | 0.0680 | 1.3085 |
| Line3 [r1,r2,c]: | 0.3093 | 0.0880 | 0.7837 | 0.3613 | 0.1000 | 0.7749 |
| Bailey test for 3 × 750 | | | | | | |
| Teta [r1,r2,c]: | 0.3051 | 0.0883 | 0.4158 | 0.3639 | 0.0841 | 0.9555 |
| SE [r1,r2,c]: | 0.0097 | 0.0060 | 0.0742 | 0.0101 | 0.0058 | 0.0892 |
| Chi2: | | 8.1542 | | | 12.4719 | |
| C Chi2: | | 6.3326 | | | 5.6215 | |

Bailey for 6 × 750:

| | |
|---|---|
| Teta: | 0.3339 0.0854 0.6411 |
| Chi2: | 59.3508 |
| C chi2: | 33.6837 |

**ML test Control vs. selection**

chi^2(ctrl + sel) - chi^2(ctrl) - chi^2(sel): 38.7247

C chi^2(ctrl + sel) - chi^2(ctrl) - chi^2(sel): **21.7296**

**ML test Ctrl Vs. Selection**

| | | |
|---|---|---|
| ML est [r1,r2,c] | 0.3049 0.0889 0.4756 | 0.3618 0.0862 0.9689 |
| ML SE [r1,r2,c] | 0.0097 0.0060 0.0796 | 0.0101 0.0059 0.0906 |

**ML test Ctrl vs. Selection (r1 and r2 are line-specific)**

c - control or selection specific

| | | | | | | |
|---|---|---|---|---|---|---|
| Line1[r1,r2,c]: | 0.2969 | 0.0990 | 0.4764 | 0.3494 | 0.0907 | 0.9710 |
| Line2[r1,r2,c]: | 0.3101 | 0.0802 | 0.4764 | 0.3745 | 0.0680 | 0.9710 |
| Line3[r1,r2,c]: | 0.3077 | 0.0875 | 0.4764 | 0.3615 | 0.1000 | 0.9710 |

c-global

| | | | | | | |
|---|---|---|---|---|---|---|
| Line1[r1,r2,c]: | 0.2973 | 0.0991 | 0.7476 | 0.3486 | 0.0905 | 0.7476 |
| Line2[r1,r2,c]: | 0.3105 | 0.0803 | 0.7476 | 0.3727 | 0.0676 | 0.7476 |
| Line3[r1,r2,c]: | 0.3092 | 0.0880 | 0.7476 | 0.3612 | 0.1000 | 0.7476 |

ML ratio (df=14 –13=1): 15.7509

***ML test Ctrl vs. Selection (r1, r2 and c are all line-specific)**

| | | | | | | |
|---|---|---|---|---|---|---|
| Line1[r1,r2,c]: | 0.2960 | 0.0987 | 0.3196 | 0.3493 | 0.0907 | 0.9261 |
| Line2[r1,r2,c]: | 0.3093 | 0.0800 | 0.3233 | 0.3747 | 0.0680 | 1.3085 |
| Line3[r1,r2,c]: | 0.3093 | 0.0880 | 0.7837 | 0.3613 | 0.1000 | 0.7749 |

ML ratio (df=18 –13=5): 28.5096

ML ratio for heterogeneity (control+selection; df = 5–1=4): 28.5096 – 15.7509

…………………………………………………………………………………………………

## markers 1-4-5 (*net-pk-cn*)

| | CONTROL | HYPEROXIA |
|---|---|---|
| **Lines (ML estimates)** | | |
| Line1 [r1,r2,c]: | 0.4507 0.0240 0.3699 | 0.3800 0.0227 0.7722 |
| Line2 [r1,r2,c]: | 0.4401 0.0240 0.0000 | 0.4093 0.0147 1.3326 |
| Line3 [r1,r2,c]: | 0.4307 0.0187 0.6615 | 0.3746 0.0240 0.2965 |

| | | |
|---|---|---|
| Bailey test for 3 × 750 | | |
| Teta [r1,r2,c]: | 0.4400 0.0219 0.0000 | 0.3891 0.0193 0.5995 |
| SE [r1,r2,c]: | 0.0105 0.0031 0.0004 | 0.0102 0.0029 0.1483 |
| Chi2: | 10.6824 | 11.2489 |
| C Chi2: | 9.3116 | 6.7867 |

Bailey for 6 × 750:
Teta: 0.4141 0.0208 0.0000
Chi2: 51.2076
C chi2: 32.1779

**<u>ML test Control vs. selection</u>**
chi^2(ctrl + sel) - chi^2(ctrl) - chi^2(sel): 29.2763
C chi^2(ctrl + sel) - chi^2(ctrl) - chi^2(sel): **<u>16.0796</u>**

**<u>ML test Ctrl Vs. Selection</u>**

| | | |
|---|---|---|
| ML est [r1,r2,c] | 0.4405 0.0222 0.3180 | 0.3880 0.0204 0.7298 |
| ML SE [r1,r2,c] | 0.0105 0.0031 0.1109 | 0.0103 0.0030 0.1697 |

**<u>ML test Ctrl vs. Selection (r1 and r2 are line-specific)</u>**

c - control or selection specific

| | | | | | | |
|---|---|---|---|---|---|---|
| Line1[r1,r2,c]: | 0.4504 | 0.0240 | 0.3197 | 0.3799 | 0.0227 | 0.7380 |
| Line2[r1,r2,c]: | 0.4412 | 0.0241 | 0.3197 | 0.4088 | 0.0147 | 0.7380 |
| Line3[r1,r2,c]: | 0.4297 | 0.0186 | 0.3197 | 0.3752 | 0.0240 | 0.7380 |

c-global

| | | | | | | |
|---|---|---|---|---|---|---|
| Line1[r1,r2,c]: | 0.4511 | 0.0240 | 0.4998 | 0.3794 | 0.0226 | 0.4998 |
| Line2[r1,r2,c]: | 0.4415 | 0.0241 | 0.4998 | 0.4080 | 0.0146 | 0.4998 |
| Line3[r1,r2,c]: | 0.4303 | 0.0187 | 0.4998 | 0.3751 | 0.0240 | 0.4998 |

ML ratio (df=14 –13=1): 4.4088

**<u>*ML test Ctrl vs. Selection (r1, r2 and c are all line-specific)</u>**

| | | | | | | |
|---|---|---|---|---|---|---|
| Line1[r1,r2,c]: | 0.4507 | 0.0240 | 0.3699 | 0.3800 | 0.0227 | 0.7722 |
| Line2[r1,r2,c]: | 0.4401 | 0.0240 | 0.0000 | 0.4093 | 0.0147 | 1.3326 |
| Line3[r1,r2,c]: | 0.4307 | 0.0187 | 0.6615 | 0.3746 | 0.0240 | 0.2965 |

ML ratio (df=18 –13=5): 17.9132

ML ratio for heterogeneity (control+selection; df = 5–1=4): 17.9132 – 4.4088
…………………………………………………………………………………………………

## <u>markers 2-4-5 (*dp-pk-cn*)</u>

| | CONTROL | HYPEROXIA |
|---|---|---|
| **<u>Lines (ML estimates)</u>** | | |
| Line1 [r1,r2,c]: | 0.3573 0.0240 0.3116 | 0.3640 0.0227 0.3237 |

| | | |
|---|---|---|
| Line2 [r1,r2,c]: | 0.3520 0.0240 0.1577 | 0.3746 0.0147 1.2109 |
| Line3 [r1,r2,c]: | 0.3413 0.0187 0.4206 | 0.3814 0.0240 0.0000 |

Bailey test for 3 × 750

| | | |
|---|---|---|
| Teta [r1,r2,c]: | 0.3502 0.0219 0.2467 | 0.3743 0.0195 0.0000 |
| SE [r1,r2,c]: | 0.0101 0.0031 0.1121 | 0.0102 0.0029 0.0005 |
| Chi2: | 2.0878 | 14.3418 |
| C Chi2: | 0.8435 | 11.5660 |

Bailey for 6 × 750:

| | |
|---|---|
| Teta: | 0.3619 0.0206 0.0000 |
| Chi2: | 24.4420 |
| C chi2: | 17.2793 |

**ML test Control vs. selection**

chi^2(ctrl + sel) - chi^2(ctrl) - chi^2(sel): 8.0124

C chi^2(ctrl + sel) - chi^2(ctrl) - chi^2(sel): **4.8698**

**ML test Ctrl Vs. Selection**

| | | |
|---|---|---|
| ML est [r1,r2,c] | 0.3503 0.0222 0.2853 | 0.3734 0.0204 0.4080 |
| ML SE [r1,r2,c] | 0.0101 0.0031 0.1206 | 0.0102 0.0030 0.1412 |

**ML test Ctrl vs. Selection (r1 and r2 are line-specific)**

c - control or selection specific

| | | | | | | |
|---|---|---|---|---|---|---|
| Line1[r1,r2,c]: | 0.3572 | 0.0240 | 0.2870 | 0.3642 | 0.0227 | 0.4088 |
| Line2[r1,r2,c]: | 0.3523 | 0.0240 | 0.2870 | 0.3735 | 0.0146 | 0.4088 |
| Line3[r1,r2,c]: | 0.3411 | 0.0187 | 0.2870 | 0.3823 | 0.0241 | 0.4088 |

c-global

| | | | | | | |
|---|---|---|---|---|---|---|
| Line1[r1,r2,c]: | 0.3574 | 0.0240 | 0.3458 | 0.3640 | 0.0226 | 0.3458 |
| Line2[r1,r2,c]: | 0.3524 | 0.0240 | 0.3458 | 0.3733 | 0.0146 | 0.3458 |
| Line3[r1,r2,c]: | 0.3412 | 0.0186 | 0.3458 | 0.3822 | 0.0240 | 0.3458 |

ML ratio (df=14 –13=1): 0.4296

***ML test Ctrl vs. Selection (r1, r2 and c are all line-specific)**

| | | | | | | |
|---|---|---|---|---|---|---|
| Line1[r1,r2,c]: | 0.3573 | 0.0240 | 0.3116 | 0.3640 | 0.0227 | 0.3237 |
| Line2[r1,r2,c]: | 0.3520 | 0.0240 | 0.1577 | 0.3746 | 0.0147 | 1.2109 |
| Line3[r1,r2,c]: | 0.3413 | 0.0187 | 0.4206 | 0.3814 | 0.0240 | 0.0000 |

ML ratio (df=18 –13=5): 13.1559

ML ratio for heterogeneity (control+selection; df = 5–1=4): 13.1559 – 0.4296

……………………………………………………………………………………………

**markers 3-4-5 (*b-pk-cn*)**

| | CONTROL | | | HYPEROXIA | | |
|---|---|---|---|---|---|---|
| **Lines (ML estimates)** | | | | | | |
| Line1 [r1,r2,c]: | 0.0747 | 0.0240 | 0.0000 | 0.0706 | 0.0227 | 0.8365 |
| Line2 [r1,r2,c]: | 0.0560 | 0.0240 | 0.0000 | 0.0560 | 0.0147 | 1.6239 |
| Line3 [r1,r2,c]: | 0.0721 | 0.0187 | 0.9901 | 0.0760 | 0.0240 | 0.0000 |
| Bailey test for 3 × 750 | | | | | | |
| Teta [r1,r2,c]: | 0.0666 | 0.0219 | 0.0000 | 0.0667 | 0.0196 | 0.0000 |
| SE [r1,r2,c]: | 0.0053 | 0.0031 | 0.0012 | 0.0052 | 0.0029 | 0.0008 |
| Chi2: | | 4.4443 | | | 7.1398 | |
| C Chi2: | | 1.0954 | | | 2.2143 | |

Bailey for 6 × 750:

| | | | |
|---|---|---|---|
| Teta: | 0.0667 | 0.0207 | 0.0000 |
| Chi2: | 11.8753 | | |
| C chi2: | 3.3097 | | |

**ML test Control vs. selection**

chi^2(ctrl + sel) - chi^2(ctrl) - chi^2(sel): 0.2912

C chi^2(ctrl + sel) - chi^2(ctrl) - chi^2(sel): **0.0000**

**ML test Ctrl Vs. Selection**

| | | | | | | |
|---|---|---|---|---|---|---|
| ML est [r1,r2,c] | 0.0676 | 0.0222 | 0.3012 | 0.0676 | 0.0204 | 0.6432 |
| ML SE [r1,r2,c] | 0.0053 | 0.0031 | 0.2945 | 0.0053 | 0.0030 | 0.4419 |

**ML test Ctrl vs. Selection (r1 and r2 are line-specific)**

| | | | | | | |
|---|---|---|---|---|---|---|
| c - control or selection specific | | | | | | |
| Line1[r1,r2,c]: | 0.0747 | 0.0240 | 0.3134 | 0.0707 | 0.0227 | 0.6275 |
| Line2[r1,r2,c]: | 0.0560 | 0.0240 | 0.3134 | 0.0560 | 0.0147 | 0.6275 |
| Line3[r1,r2,c]: | 0.0719 | 0.0186 | 0.3134 | 0.0760 | 0.0240 | 0.6275 |
| c-global | | | | | | |
| Line1[r1,r2,c]: | 0.0747 | 0.0240 | 0.4773 | 0.0706 | 0.0227 | 0.4773 |
| Line2[r1,r2,c]: | 0.0560 | 0.0240 | 0.4773 | 0.0560 | 0.0147 | 0.4773 |
| Line3[r1,r2,c]: | 0.0720 | 0.0187 | 0.4773 | 0.0760 | 0.0240 | 0.4773 |

ML ratio (df=14 –13=1): 0.4083

***ML test Ctrl vs. Selection (r1, r2 and c are all line-specific)**

| | | | | | | |
|---|---|---|---|---|---|---|
| Line1[r1,r2,c]: | 0.0747 | 0.0240 | 0.0000 | 0.0706 | 0.0227 | 0.8365 |
| Line2[r1,r2,c]: | 0.0560 | 0.0240 | 0.0000 | 0.0560 | 0.0147 | 1.6239 |
| Line3[r1,r2,c]: | 0.0721 | 0.0187 | 0.9901 | 0.0760 | 0.0240 | 0.0000 |

ML ratio (df=18 –13=5): 5.4556

ML ratio for heterogeneity (control+selection; df = 5–1=4): 5.4556 – 0.4083

……………………………………………………………………………………………………

## 2R chromosome

## <u>Control and hypoxia lines</u>

### markers 1-2-4 (*cn-kn-px*)

| | CONTROL | HYPOXIA |
|---|---|---|
| **<u>Lines (ML estimates)</u>** | | |
| Line1 [r1,r2,c]: | 0.1040 0.2773 0.3698 | 0.0987 0.2360 0.5153 |
| Line2 [r1,r2,c]: | 0.0947 0.2893 0.4381 | 0.1040 0.2307 0.3335 |
| Line3 [r1,r2,c]: | 0.0987 0.2320 0.3495 | 0.1093 0.2467 0.1978 |
| | | |
| Bailey test for 3 × 750 | | |
| Teta [r1,r2,c]: | 0.0991 0.2647 0.3835 | 0.1039 0.2378 0.2973 |
| SE [r1,r2,c]: | 0.0063 0.0093 0.0744 | 0.0064 0.0090 0.0686 |
| Chi2: | 7.9383 | 4.3732 |
| C Chi2: | 0.2462 | 3.1247 |

Bailey for 6 × 750:
Teta: 0.1015 0.2508 0.3366
Chi2: 17.3540
C chi2: 4.1107

**<u>ML test Control vs. selection</u>**
chi^2(ctrl + sel) - chi^2(ctrl) - chi^2(sel): 5.0425
C chi^2(ctrl + sel) - chi^2(ctrl) - chi^2(sel): **<u>0.7399</u>**

**<u>ML test Ctrl Vs. Selection</u>**

| | CONTROL | HYPOXIA |
|---|---|---|
| ML est [r1,r2,c] | 0.0991 0.2662 0.3874 | 0.1040 0.2378 0.3415 |
| ML SE [r1,r2,c] | 0.0063 0.0093 0.0747 | 0.0064 0.0090 0.0735 |

**<u>ML test Ctrl vs. Selection (r1 and r2 are line-specific)</u>**

| | | | | | | |
|---|---|---|---|---|---|---|
| c - control or selection specific | | | | | | |
| Line1[r1,r2,c]: | 0.1040 | 0.2774 | 0.3878 | 0.0984 | 0.2353 | 0.3407 |
| Line2[r1,r2,c]: | 0.0946 | 0.2890 | 0.3878 | 0.1040 | 0.2307 | 0.3407 |
| Line3[r1,r2,c]: | 0.0987 | 0.2321 | 0.3878 | 0.1096 | 0.2474 | 0.3407 |
| c-global | | | | | | |
| Line1[r1,r2,c]: | 0.1040 | 0.2773 | 0.3652 | 0.0984 | 0.2354 | 0.3652 |
| Line2[r1,r2,c]: | 0.0945 | 0.2889 | 0.3652 | 0.1041 | 0.2308 | 0.3652 |
| Line3[r1,r2,c]: | 0.0987 | 0.2321 | 0.3652 | 0.1097 | 0.2475 | 0.3652 |

ML ratio (df=14 –13=1): 0.2018

***ML test Ctrl vs. Selection (r1, r2 and c are all line-specific)**

| | | | | | | |
|---|---|---|---|---|---|---|
| Line1[r1,r2,c]: | 0.1040 | 0.2773 | 0.3698 | 0.0987 | 0.2360 | 0.5153 |
| Line2[r1,r2,c]: | 0.0947 | 0.2893 | 0.4381 | 0.1040 | 0.2307 | 0.3335 |
| Line3[r1,r2,c]: | 0.0987 | 0.2320 | 0.3495 | 0.1093 | 0.2467 | 0.1978 |

ML ratio (df=18 –13=5): 3.6150

ML ratio for heterogeneity (control+selection; df = 5–1=4): 3.6150 – 0.2018

………………………………………………………………………………………………

## markers 1-2-5 (*cn-kn-sp*)

| | CONTROL | | | HYPOXIA | | |
|---|---|---|---|---|---|---|
| **Lines (ML estimates)** | | | | | | |
| Line1 [r1,r2,c]: | 0.1040 | 0.3347 | 0.3065 | 0.0987 | 0.2973 | 0.5908 |
| Line2 [r1,r2,c]: | 0.0947 | 0.3560 | 0.3956 | 0.1040 | 0.2880 | 0.4452 |
| Line3 [r1,r2,c]: | 0.0987 | 0.3013 | 0.6278 | 0.1093 | 0.3173 | 0.3074 |
| Bailey test for 3 × 750 | | | | | | |
| Teta [r1,r2,c]: | 0.0995 | 0.3300 | 0.4027 | 0.1040 | 0.3009 | 0.4127 |
| SE [r1,r2,c]: | 0.0063 | 0.0099 | 0.0668 | 0.0064 | 0.0097 | 0.0694 |
| Chi2: | | 9.1408 | | | 5.1898 | |
| C Chi2: | | 3.3169 | | | 2.6928 | |

Bailey for 6 × 750:

| | | | |
|---|---|---|---|
| Teta: | 0.1018 | 0.3151 | 0.4072 |
| Chi2: | 18.8797 | | |
| C chi2: | 6.0192 | | |

**ML test Control vs. selection**

chi^2(ctrl + sel) - chi^2(ctrl) - chi^2(sel): 4.5491

C chi^2(ctrl + sel) - chi^2(ctrl) - chi^2(sel): **0.0095**

**ML test Ctrl Vs. Selection**

| | | | | | | |
|---|---|---|---|---|---|---|
| ML est [r1,r2,c] | 0.0991 | 0.3307 | 0.4340 | 0.1040 | 0.3009 | 0.4403 |
| ML SE [r1,r2,c] | 0.0063 | 0.0099 | 0.0691 | 0.0064 | 0.0097 | 0.0716 |

**ML test Ctrl vs. Selection (r1 and r2 are line-specific)**

c - control or selection specific

| | | | | | | |
|---|---|---|---|---|---|---|
| Line1[r1,r2,c]: | 0.1043 | 0.3357 | 0.4329 | 0.0984 | 0.2964 | 0.4389 |
| Line2[r1,r2,c]: | 0.0947 | 0.3563 | 0.4329 | 0.1040 | 0.2880 | 0.4389 |
| Line3[r1,r2,c]: | 0.0983 | 0.3001 | 0.4329 | 0.1097 | 0.3183 | 0.4389 |

c-global

| | | | | | | |
|---|---|---|---|---|---|---|
| Line1[r1,r2,c]: | 0.1043 | 0.3357 | 0.4358 | 0.0984 | 0.2964 | 0.4358 |
| Line2[r1,r2,c]: | 0.0948 | 0.3564 | 0.4358 | 0.1040 | 0.2880 | 0.4358 |

| | | | | | | |
|---|---|---|---|---|---|---|
| Line3[r1,r2,c]: | 0.0983 | 0.3002 | 0.4358 | 0.1097 | 0.3183 | 0.4358 |

ML ratio (df=14 –13=1): 0.0036

**<u>*ML test Ctrl vs. Selection (r1, r2 and c are all line-specific)</u>**

| | | | | | | |
|---|---|---|---|---|---|---|
| Line1[r1,r2,c]: | 0.1040 | 0.3347 | 0.3065 | 0.0987 | 0.2973 | 0.5908 |
| Line2[r1,r2,c]: | 0.0947 | 0.3560 | 0.3956 | 0.1040 | 0.2880 | 0.4452 |
| Line3[r1,r2,c]: | 0.0987 | 0.3013 | 0.6278 | 0.1093 | 0.3173 | 0.3074 |

ML ratio (df=18 –13=5): 6.2220

ML ratio for heterogeneity (control+selection; df = 5–1=4): 6.2220– 0.0036

……………………………………………………………………………………………………

## markers 1-3-4 (*cn-c-px*)

| | CONTROL | | | HYPOXIA | | |
|---|---|---|---|---|---|---|
| **Lines (ML estimates)** | | | | | | |
| Line1 [r1,r2,c]: | 0.1267 | 0.2547 | 0.3307 | 0.1160 | 0.2213 | 0.5193 |
| Line2 [r1,r2,c]: | 0.1160 | 0.2707 | 0.4247 | 0.1200 | 0.2147 | 0.3105 |
| Line3 [r1,r2,c]: | 0.1147 | 0.2187 | 0.3722 | 0.1253 | 0.2307 | 0.1845 |

| Bailey test for 3 × 750 | | | | | | |
|---|---|---|---|---|---|---|
| Teta [r1,r2,c]: | 0.1191 | 0.2468 | 0.3706 | 0.1204 | 0.2222 | 0.2828 |
| SE [r1,r2,c]: | 0.0068 | 0.0091 | 0.0693 | 0.0069 | 0.0088 | 0.0644 |
| Chi2: | | 7.0784 | | | 4.8903 | |
| C Chi2: | | 0.3296 | | | 3.7939 | |

| Bailey for 6 × 750: | | | |
|---|---|---|---|
| Teta: | 0.1197 | 0.2340 | 0.3233 |
| Chi2: | 16.5147 | | |
| C chi2: | 5.0069 | | |

**<u>ML test Control vs. selection</u>**

chi^2(ctrl + sel) - chi^2(ctrl) - chi^2(sel): 4.5459

C chi^2(ctrl + sel) - chi^2(ctrl) - chi^2(sel): **<u>0.8834</u>**

**<u>ML test Ctrl Vs. Selection</u>**

| | | | | | | |
|---|---|---|---|---|---|---|
| ML est [r1,r2,c] | 0.1191 | 0.2480 | 0.3761 | 0.1204 | 0.2222 | 0.3321 |
| ML SE [r1,r2,c] | 0.0068 | 0.0091 | 0.0697 | 0.0069 | 0.0088 | 0.0698 |

**<u>ML test Ctrl vs. Selection (r1 and r2 are line-specific)</u>**

c - control or selection specific

| | | | | | | |
|---|---|---|---|---|---|---|
| Line1[r1,r2,c]: | 0.1268 | 0.2549 | 0.3757 | 0.1156 | 0.2205 | 0.3316 |
| Line2[r1,r2,c]: | 0.1159 | 0.2704 | 0.3757 | 0.1200 | 0.2148 | 0.3316 |

| | | | | | | |
|---|---|---|---|---|---|---|
| Line3[r1,r2,c]: | 0.1147 | 0.2187 | 0.3757 | 0.1257 | 0.2314 | 0.3316 |

c-global

| | | | | | | |
|---|---|---|---|---|---|---|
| Line1[r1,r2,c]: | 0.1267 | 0.2548 | 0.3549 | 0.1156 | 0.2206 | 0.3549 |
| Line2[r1,r2,c]: | 0.1158 | 0.2702 | 0.3549 | 0.1201 | 0.2148 | 0.3549 |
| Line3[r1,r2,c]: | 0.1146 | 0.2186 | 0.3549 | 0.1258 | 0.2315 | 0.3549 |

ML ratio (df=14 –13=1): 0.2012

***ML test Ctrl vs. Selection (r1, r2 and c are all line-specific)**

| | | | | | | |
|---|---|---|---|---|---|---|
| Line1[r1,r2,c]: | 0.1267 | 0.2547 | 0.3307 | 0.1160 | 0.2213 | 0.5193 |
| Line2[r1,r2,c]: | 0.1160 | 0.2707 | 0.4247 | 0.1200 | 0.2147 | 0.3105 |
| Line3[r1,r2,c]: | 0.1147 | 0.2187 | 0.3722 | 0.1253 | 0.2307 | 0.1845 |

ML ratio (df=18 –13=5): 4.4534

ML ratio for heterogeneity (control+selection; df = 5–1=4): 4.4534 – 0.2012
……………………………………………………………………………………………………………

**markers 1-3-5 (*cn-c-sp*)**

| | CONTROL | | | HYPOXIA | | |
|---|---|---|---|---|---|---|
| **Lines (ML estimates)** | | | | | | |
| Line1 [r1,r2,c]: | 0.1267 | 0.3120 | 0.2699 | 0.1160 | 0.2827 | 0.5693 |
| Line2 [r1,r2,c]: | 0.1160 | 0.3373 | 0.3748 | 0.1200 | 0.2720 | 0.4085 |
| Line3 [r1,r2,c]: | 0.1147 | 0.2907 | 0.6401 | 0.1253 | 0.3013 | 0.2824 |
| Bailey test for 3 × 750 | | | | | | |
| Teta [r1,r2,c]: | 0.1194 | 0.3130 | 0.3753 | 0.1205 | 0.2852 | 0.3845 |
| SE [r1,r2,c]: | 0.0068 | 0.0098 | 0.0606 | 0.0069 | 0.0095 | 0.0643 |
| Chi2: | | 9.8249 | | | 5.5153 | |
| C Chi2: | | 5.0729 | | | 3.1473 | |

Bailey for 6 × 750:

| | | | |
|---|---|---|---|
| Teta: | 0.1200 | 0.2988 | 0.3792 |
| Chi2: | 19.5806 | | |
| C chi2: | 8.2297 | | |

**ML test Control vs. selection**

chi^2(ctrl + sel) - chi^2(ctrl) - chi^2(sel): 4.2404
C chi^2(ctrl + sel) - chi^2(ctrl) - chi^2(sel**): 0.0096**

**ML test Ctrl Vs. Selection**

| | | | | | | |
|---|---|---|---|---|---|---|
| ML est [r1,r2,c] | 0.1191 | 0.3133 | 0.4168 | 0.1204 | 0.2853 | 0.4138 |
| ML SE [r1,r2,c] | 0.0068 | 0.0098 | 0.0637 | 0.0069 | 0.0095 | 0.0666 |

**ML test Ctrl vs. Selection (r1 and r2 are line-specific)**

c - control or selection specific

| | | | | | | |
|---|---|---|---|---|---|---|
| Line1[r1,r2,c]: | 0.1272 | 0.3133 | 0.4152 | 0.1156 | 0.2816 | 0.4127 |
| Line2[r1,r2,c]: | 0.1161 | 0.3377 | 0.4152 | 0.1200 | 0.2720 | 0.4127 |
| Line3[r1,r2,c]: | 0.1141 | 0.2891 | 0.4152 | 0.1258 | 0.3024 | 0.4127 |

c-global

| | | | | | | |
|---|---|---|---|---|---|---|
| Line1[r1,r2,c]: | 0.1272 | 0.3133 | 0.4141 | 0.1156 | 0.2817 | 0.4141 |
| Line2[r1,r2,c]: | 0.1161 | 0.3377 | 0.4141 | 0.1200 | 0.2720 | 0.4141 |
| Line3[r1,r2,c]: | 0.1141 | 0.2891 | 0.4141 | 0.1258 | 0.3024 | 0.4141 |

ML ratio (df=14 –13=1): 0.0008

**<u>*ML test Ctrl vs. Selection (r1, r2 and c are all line-specific)</u>**

| | | | | | | |
|---|---|---|---|---|---|---|
| Line1[r1,r2,c]: | 0.1267 | 0.3120 | 0.2699 | 0.1160 | 0.2827 | 0.5693 |
| Line2[r1,r2,c]: | 0.1160 | 0.3373 | 0.3748 | 0.1200 | 0.2720 | 0.4085 |
| Line3[r1,r2,c]: | 0.1147 | 0.2907 | 0.6401 | 0.1253 | 0.3013 | 0.2824 |

ML ratio (df=18 –13=5): 8.6179

ML ratio for heterogeneity (control+selection; df = 5–1=4): 8.6179 – 0.0008
…………………………………………………………………………………………………………

## <u>markers 2-3-4 (*kn-c-px*)</u>

| | CONTROL | | | HYPOXIA | | |
|---|---|---|---|---|---|---|
| **<u>Lines (ML estimates)</u>** | | | | | | |
| Line1 [r1,r2,c]: | 0.0253 | 0.2547 | 0.2066 | 0.0173 | 0.2214 | 0.3455 |
| Line2 [r1,r2,c]: | 0.0213 | 0.2707 | 0.2306 | 0.0187 | 0.2146 | 0.3402 |
| Line3 [r1,r2,c]: | 0.0160 | 0.2186 | 0.3786 | 0.0160 | 0.2307 | 0.0000 |
| Bailey test for 3 × 750 | | | | | | |
| Teta [r1,r2,c]: | 0.0202 | 0.2469 | 0.2383 | 0.0173 | 0.2219 | 0.0000 |
| SE [r1,r2,c]: | 0.0030 | 0.0091 | 0.1378 | 0.0027 | 0.0088 | 0.0006 |
| Chi2: | | 8.0691 | | | 2.8958 | |
| C Chi2: | | 0.1759 | | | 2.1954 | |

Bailey for 6 × 750:

| | | | |
|---|---|---|---|
| Teta: | 0.0186 | 0.2339 | 0.0000 |
| Chi2: | 18.5047 | | |
| C chi2: | 5.4195 | | |

**<u>ML test Control vs. selection</u>**

chi^2(ctrl + sel) - chi^2(ctrl) - chi^2(sel): 7.5399

C chi^2(ctrl + sel) - chi^2(ctrl) - chi^2(sel): **<u>3.0483</u>**

**ML test Ctrl Vs. Selection**

| | | | | | | |
|---|---|---|---|---|---|---|
| ML est [r1,r2,c] | 0.0209 | 0.2480 | 0.2571 | 0.0173 | 0.2223 | 0.2350 |
| ML SE [r1,r2,c] | 0.0030 | 0.0091 | 0.1433 | 0.0028 | 0.0088 | 0.1599 |

**ML test Ctrl vs. Selection (r1 and r2 are line-specific)**

c - control or selection specific

| | | | | | | |
|---|---|---|---|---|---|---|
| Line1[r1,r2,c]: | 0.0253 | 0.2547 | 0.2545 | 0.0173 | 0.2213 | 0.2345 |
| Line2[r1,r2,c]: | 0.0213 | 0.2707 | 0.2545 | 0.0187 | 0.2146 | 0.2345 |
| Line3[r1,r2,c]: | 0.0160 | 0.2186 | 0.2545 | 0.0160 | 0.2308 | 0.2345 |

c-global

| | | | | | | |
|---|---|---|---|---|---|---|
| Line1[r1,r2,c]: | 0.0253 | 0.2547 | 0.2469 | 0.0173 | 0.2212 | 0.2469 |
| Line2[r1,r2,c]: | 0.0213 | 0.2707 | 0.2469 | 0.0187 | 0.2146 | 0.2469 |
| Line3[r1,r2,c]: | 0.0160 | 0.2186 | 0.2469 | 0.0160 | 0.2308 | 0.2469 |

ML ratio (df=14 –13=1): 0.0116

***ML test Ctrl vs. Selection (r1, r2 and c are all line-specific)**

| | | | | | | |
|---|---|---|---|---|---|---|
| Line1[r1,r2,c]: | 0.0253 | 0.2547 | 0.2066 | 0.0173 | 0.2214 | 0.3455 |
| Line2[r1,r2,c]: | 0.0213 | 0.2707 | 0.2306 | 0.0187 | 0.2146 | 0.3402 |
| Line3[r1,r2,c]: | 0.0160 | 0.2186 | 0.3786 | 0.0160 | 0.2307 | 0.0000 |

ML ratio (df=18 –13=5): 1.8253

ML ratio for heterogeneity (control+selection; df = 5–1=4): 1.8253 – 0.0116

……………………………………………………………………………………………………

## markers 2-3-5 (*kn-c-sp*)

**Lines (ML estimates)**

| | CONTROL | | | HYPOXIA | | |
|---|---|---|---|---|---|---|
| Line1 [r1,r2,c]: | 0.0253 | 0.3120 | 0.1688 | 0.0173 | 0.2826 | 0.2741 |
| Line2 [r1,r2,c]: | 0.0213 | 0.3373 | 0.1855 | 0.0187 | 0.2720 | 0.2613 |
| Line3 [r1,r2,c]: | 0.0160 | 0.2906 | 0.5724 | 0.0160 | 0.3013 | 0.0000 |

Bailey test for 3 × 750

| | | | | | | |
|---|---|---|---|---|---|---|
| Teta [r1,r2,c]: | 0.0203 | 0.3131 | 0.2130 | 0.0173 | 0.2848 | 0.0000 |
| SE [r1,r2,c]: | 0.0030 | 0.0098 | 0.1148 | 0.0027 | 0.0095 | 0.0004 |
| Chi2: | | 6.7693 | | | 3.9097 | |
| C Chi2: | | 1.0504 | | | 2.1755 | |

Bailey for 6 × 750:

| | | | |
|---|---|---|---|
| Teta: | 0.0187 | 0.2984 | 0.0000 |
| Chi2: | 19.0786 | | |
| C chi2: | 6.7316 | | |

**ML test Control vs. selection**

chi^2(ctrl + sel) - chi^2(ctrl) - chi^2(sel): 8.3996

C chi^2(ctrl + sel) - chi^2(ctrl) - chi^2(sel): **3.5056**

**ML test Ctrl Vs. Selection**

| | | | | | | |
|---|---|---|---|---|---|---|
| ML est [r1,r2,c] | 0.0209 | 0.3133 | 0.2716 | 0.0173 | 0.2853 | 0.1799 |
| ML SE [r1,r2,c] | 0.0030 | 0.0098 | 0.1294 | 0.0028 | 0.0095 | 0.1236 |

**ML test Ctrl vs. Selection (r1 and r2 are line-specific)**

c - control or selection specific

| | | | | | | |
|---|---|---|---|---|---|---|
| Line1[r1,r2,c]: | 0.0254 | 0.3122 | 0.2703 | 0.0173 | 0.2825 | 0.1810 |
| Line2[r1,r2,c]: | 0.0214 | 0.3375 | 0.2703 | 0.0187 | 0.2719 | 0.1810 |
| Line3[r1,r2,c]: | 0.0160 | 0.2903 | 0.2703 | 0.0160 | 0.3015 | 0.1810 |

c-global

| | | | | | | |
|---|---|---|---|---|---|---|
| Line1[r1,r2,c]: | 0.0253 | 0.3121 | 0.2332 | 0.0173 | 0.2826 | 0.2332 |
| Line2[r1,r2,c]: | 0.0213 | 0.3374 | 0.2332 | 0.0187 | 0.2720 | 0.2332 |
| Line3[r1,r2,c]: | 0.0160 | 0.2903 | 0.2332 | 0.0160 | 0.3016 | 0.2332 |

ML ratio (df=14 –13=1): 0.2424

***ML test Ctrl vs. Selection (r1, r2 and c are all line-specific)**

| | | | | | | |
|---|---|---|---|---|---|---|
| Line1[r1,r2,c]: | 0.0253 | 0.3120 | 0.1688 | 0.0173 | 0.2826 | 0.2741 |
| Line2[r1,r2,c]: | 0.0213 | 0.3373 | 0.1855 | 0.0187 | 0.2720 | 0.2613 |
| Line3[r1,r2,c]: | 0.0160 | 0.2906 | 0.5724 | 0.0160 | 0.3013 | 0.0000 |

ML ratio (df=18 –13=5): 3.3450

ML ratio for heterogeneity (control+selection; df = 5–1=4): 3.3450 –0.2424

……………………………………………………………………………………………………

**markers 1-4-5 (*cn-px-sp*)**

| | CONTROL | | | HYPOXIA | | |
|---|---|---|---|---|---|---|
| **Lines (ML estimates)** | | | | | | |
| Line1 [r1,r2,c]: | 0.3600 | 0.0707 | 0.2621 | 0.3107 | 0.0667 | 0.3863 |
| Line2 [r1,r2,c]: | 0.3600 | 0.0773 | 0.2395 | 0.3187 | 0.0627 | 0.4006 |
| Line3 [r1,r2,c]: | 0.3147 | 0.0800 | 0.6356 | 0.3453 | 0.0840 | 0.4137 |
| Bailey test for 3 × 750 | | | | | | |
| Teta [r1,r2,c]: | 0.3441 | 0.0763 | 0.3170 | 0.3248 | 0.0701 | 0.3995 |
| SE [r1,r2,c]: | 0.0100 | 0.0056 | 0.0675 | 0.0099 | 0.0054 | 0.0802 |
| Chi2: | | 10.2052 | | | 5.5982 | |
| C Chi2: | | 4.8468 | | | 0.0212 | |

Bailey for 6 × 750:

Teta: 0.3344 0.0731 0.3507

| | |
|---|---|
| Chi2: | 19.4052 |
| C chi2: | 5.4926 |

**<u>ML test Control vs. selection</u>**

chi^2(ctrl + sel) - chi^2(ctrl) - chi^2(sel): 3.6018
C chi^2(ctrl + sel) - chi^2(ctrl) - chi^2(sel): 0.6245

**<u>ML test Ctrl Vs. Selection</u>**

| | | | | | | |
|---|---|---|---|---|---|---|
| ML est [r1,r2,c] | 0.3449 | 0.0760 | 0.3730 | 0.3249 | 0.0711 | 0.4040 |
| ML SE [r1,r2,c] | 0.0100 | 0.0056 | 0.0729 | 0.0099 | 0.0054 | 0.0807 |

**<u>ML test Ctrl vs. Selection (r1 and r2 are line-specific)</u>**

c - control or selection specific

| | | | | | | |
|---|---|---|---|---|---|---|
| Line1[r1,r2,c]: | 0.3608 | 0.0708 | 0.3723 | 0.3107 | 0.0667 | 0.4019 |
| Line2[r1,r2,c]: | 0.3610 | 0.0775 | 0.3723 | 0.3187 | 0.0627 | 0.4019 |
| Line3[r1,r2,c]: | 0.3131 | 0.0796 | 0.3723 | 0.3452 | 0.0840 | 0.4019 |

c-global

| | | | | | | |
|---|---|---|---|---|---|---|
| Line1[r1,r2,c]: | 0.3608 | 0.0708 | 0.3862 | 0.3107 | 0.0667 | 0.3862 |
| Line2[r1,r2,c]: | 0.3611 | 0.0776 | 0.3862 | 0.3186 | 0.0626 | 0.3862 |
| Line3[r1,r2,c]: | 0.3132 | 0.0796 | 0.3862 | 0.3451 | 0.0839 | 0.3862 |

ML ratio (df=14 –13=1): 0.0750

**<u>*ML test Ctrl vs. Selection (r1, r2 and c are all line-specific)</u>**

| | | | | | | |
|---|---|---|---|---|---|---|
| Line1[r1,r2,c]: | 0.3600 | 0.0707 | 0.2621 | 0.3107 | 0.0667 | 0.3863 |
| Line2[r1,r2,c]: | 0.3600 | 0.0773 | 0.2395 | 0.3187 | 0.0627 | 0.4006 |
| Line3[r1,r2,c]: | 0.3147 | 0.0800 | 0.6356 | 0.3453 | 0.0840 | 0.4137 |

ML ratio (df=18 –13=5): 5.7659

ML ratio for heterogeneity (control+selection; df = 5–1=4): 5.7659 – 0.0750

………………………………………………………………………………………………………

## <u>markers 2-4-5 (*kn-px-sp*)</u>

| | CONTROL | | | HYPOXIA | | |
|---|---|---|---|---|---|---|
| **<u>Lines (ML estimates)</u>** | | | | | | |
| Line1 [r1,r2,c]: | 0.2773 | 0.0707 | 0.3402 | 0.2360 | 0.0667 | 0.1695 |
| Line2 [r1,r2,c]: | 0.2893 | 0.0773 | 0.2384 | 0.2307 | 0.0627 | 0.1845 |
| Line3 [r1,r2,c]: | 0.2320 | 0.0800 | 0.2874 | 0.2467 | 0.0840 | 0.3217 |

Bailey test for 3 × 750

| | | | | | | |
|---|---|---|---|---|---|---|
| Teta [r1,r2,c]: | 0.2647 | 0.0760 | 0.2794 | 0.2377 | 0.0700 | 0.2172 |
| SE [r1,r2,c]: | 0.0093 | 0.0056 | 0.0746 | 0.0090 | 0.0054 | 0.0726 |
| Chi2: | | 8.0576 | | | 4.3413 | |

| | | |
|---|---|---|
| C Chi2: | 0.3155 | 0.8271 |

Bailey for 6 × 750:

| | |
|---|---|
| Teta: | 0.2507 0.0729 0.2470 |
| Chi2: | 18.1042 |
| C chi2: | 1.5084 |

**ML test Control vs. selection**

chi^2(ctrl + sel) - chi^2(ctrl) - chi^2(sel): 5.7052
C chi^2(ctrl + sel) - chi^2(ctrl) - chi^2(sel**): 0.3657**

**ML test Ctrl Vs. Selection**

| | | |
|---|---|---|
| ML est [r1,r2,c] | 0.2662 0.0760 0.2856 | 0.2378 0.0711 0.2366 |
| ML SE [r1,r2,c] | 0.0093 0.0056 0.0751 | 0.0090 0.0054 0.0758 |

**ML test Ctrl vs. Selection (r1 and r2 are line-specific)**

c - control or selection specific

| | | | | | | |
|---|---|---|---|---|---|---|
| Line1[r1,r2,c]: | 0.2771 | 0.0706 | 0.2862 | 0.2362 | 0.0667 | 0.2360 |
| Line2[r1,r2,c]: | 0.2896 | 0.0774 | 0.2862 | 0.2308 | 0.0627 | 0.2360 |
| Line3[r1,r2,c]: | 0.2320 | 0.0800 | 0.2862 | 0.2463 | 0.0839 | 0.2360 |

c-global

| | | | | | | |
|---|---|---|---|---|---|---|
| Line1[r1,r2,c]: | 0.2770 | 0.0706 | 0.2636 | 0.2363 | 0.0667 | 0.2636 |
| Line2[r1,r2,c]: | 0.2895 | 0.0774 | 0.2636 | 0.2309 | 0.0627 | 0.2636 |
| Line3[r1,r2,c]: | 0.2319 | 0.0800 | 0.2636 | 0.2464 | 0.0839 | 0.2636 |

ML ratio (df=14 –13=1): 0.2190

***ML test Ctrl vs. Selection (r1, r2 and c are all line-specific)**

| | | | | | | |
|---|---|---|---|---|---|---|
| Line1[r1,r2,c]: | 0.2773 | 0.0707 | 0.3402 | 0.2360 | 0.0667 | 0.1695 |
| Line2[r1,r2,c]: | 0.2893 | 0.0773 | 0.2384 | 0.2307 | 0.0627 | 0.1845 |
| Line3[r1,r2,c]: | 0.2320 | 0.0800 | 0.2874 | 0.2467 | 0.0840 | 0.3217 |

ML ratio (df=18 –13=5): 1.4168

ML ratio for heterogeneity (control+selection; df = 5–1=4): 1.4168 – 0.2190
…………………………………………………………………………………………………………

## markers 3-4-5 (*c-px-sp*)

| | CONTROL | HYPOXIA |
|---|---|---|
| **Lines (ML estimates)** | | |
| Line1 [r1,r2,c]: | 0.2547 0.0707 0.3705 | 0.2213 0.0667 0.1807 |
| Line2 [r1,r2,c]: | 0.2707 0.0773 0.2548 | 0.2147 0.0627 0.1982 |
| Line3 [r1,r2,c]: | 0.2187 0.0800 0.2287 | 0.2307 0.0840 0.3441 |

Bailey test for 3 × 750

| | | |
|---|---|---|
| Teta [r1,r2,c]: | 0.2467 0.0759 0.2725 | 0.2221 0.0700 0.2325 |
| SE [r1,r2,c]: | 0.0091 0.0056 0.0765 | 0.0088 0.0054 0.0776 |
| Chi2: | 6.8203 | 4.3342 |
| C Chi2: | 0.5388 | 0.8288 |

Bailey for 6 × 750:

| | | | |
|---|---|---|---|
| Teta: | 0.2340 | 0.0729 | 0.2523 |
| Chi2: | 16.0197 | | |
| C chi2: | 1.5052 | | |

**<u>ML test Control vs. selection</u>**

chi^2(ctrl + sel) - chi^2(ctrl) - chi^2(sel): 4.8653

C chi^2(ctrl + sel) - chi^2(ctrl) - chi^2(sel): **<u>0.1376</u>**

**<u>ML test Ctrl Vs. Selection</u>**

| | | |
|---|---|---|
| ML est [r1,r2,c] | 0.2480 0.0760 0.2830 | 0.2222 0.0711 0.2531 |
| ML SE [r1,r2,c] | 0.0091 0.0056 0.0778 | 0.0088 0.0054 0.0811 |

**<u>ML test Ctrl vs. Selection (r1 and r2 are line-specific)</u>**

c - control or selection specific

| | | | | | | |
|---|---|---|---|---|---|---|
| Line1[r1,r2,c]: | 0.2543 | 0.0706 | 0.2836 | 0.2215 | 0.0667 | 0.2525 |
| Line2[r1,r2,c]: | 0.2708 | 0.0774 | 0.2836 | 0.2148 | 0.0627 | 0.2525 |
| Line3[r1,r2,c]: | 0.2188 | 0.0801 | 0.2836 | 0.2303 | 0.0839 | 0.2525 |

c-global

| | | | | | | |
|---|---|---|---|---|---|---|
| Line1[r1,r2,c]: | 0.2543 | 0.0706 | 0.2693 | 0.2216 | 0.0667 | 0.2693 |
| Line2[r1,r2,c]: | 0.2707 | 0.0773 | 0.2693 | 0.2148 | 0.0627 | 0.2693 |
| Line3[r1,r2,c]: | 0.2188 | 0.0800 | 0.2693 | 0.2304 | 0.0839 | 0.2693 |

ML ratio (df=14 –13=1): 0.0763

**<u>*ML test Ctrl vs. Selection (r1, r2 and c are all line-specific)</u>**

| | | | | | | |
|---|---|---|---|---|---|---|
| Line1[r1,r2,c]: | 0.2547 | 0.0707 | 0.3705 | 0.2213 | 0.0667 | 0.1807 |
| Line2[r1,r2,c]: | 0.2707 | 0.0773 | 0.2548 | 0.2147 | 0.0627 | 0.1982 |
| Line3[r1,r2,c]: | 0.2187 | 0.0800 | 0.2287 | 0.2307 | 0.0840 | 0.3441 |

ML ratio (df=18 –13=5): 1.5381

ML ratio for heterogeneity (control+selection; df = 5–1=4): 1.5381 – 0.0763

……………………………………………………………………………………………………

### markers 1-2-4 (*cn-kn-px*)

| | CONTROL | HYPEROXIA |
|---|---|---|
| **<u>Lines (ML estimates)</u>** | | |
| Line1 [r1,r2,c]: | 0.1040 0.2773 0.3698 | 0.1080 0.2787 0.3987 |

| | | |
|---|---|---|
| Line2 [r1,r2,c]: | 0.0947 0.2893 0.4381 | 0.1000 0.2413 0.6630 |
| Line3 [r1,r2,c]: | 0.0987 0.2320 0.3495 | 0.0933 0.2427 0.4121 |

Bailey test for 3 × 750

| | | |
|---|---|---|
| Teta [r1,r2,c]: | 0.0991 0.2647 0.3835 | 0.1004 0.2536 0.4623 |
| SE [r1,r2,c]: | 0.0063 0.0093 0.0744 | 0.0063 0.0092 0.0816 |
| Chi2: | 7.9383 | 6.7203 |
| C Chi2: | 0.2462 | 1.8061 |

Bailey for 6 × 750:

| | |
|---|---|
| Teta: | 0.0998 0.2591 0.4191 |
| Chi2: | 15.9331 |
| C chi2: | 2.5742 |

**ML test Control vs. selection**

chi^2(ctrl + sel) - chi^2(ctrl) - chi^2(sel): 1.2746

C chi^2(ctrl + sel) - chi^2(ctrl) - chi^2(sel): **0.5220**

**ML test Ctrl Vs. Selection**

| | | |
|---|---|---|
| ML est [r1,r2,c] | 0.0991 0.2662 0.3874 | 0.1004 0.2542 0.4873 |
| ML SE [r1,r2,c] | 0.0063 0.0093 0.0747 | 0.0063 0.0092 0.0837 |

**ML test Ctrl vs. Selection (r1 and r2 are line-specific)**

c - control or selection specific

| | | | | | | |
|---|---|---|---|---|---|---|
| Line1[r1,r2,c]: | 0.1040 | 0.2774 | 0.3879 | 0.1082 | 0.2791 | 0.4847 |
| Line2[r1,r2,c]: | 0.0946 | 0.2890 | 0.3879 | 0.0997 | 0.2407 | 0.4847 |
| Line3[r1,r2,c]: | 0.0987 | 0.2321 | 0.3879 | 0.0934 | 0.2429 | 0.4847 |

c-global

| | | | | | | |
|---|---|---|---|---|---|---|
| Line1[r1,r2,c]: | 0.1041 | 0.2777 | 0.4356 | 0.1081 | 0.2789 | 0.4356 |
| Line2[r1,r2,c]: | 0.0947 | 0.2893 | 0.4356 | 0.0996 | 0.2405 | 0.4356 |
| Line3[r1,r2,c]: | 0.0988 | 0.2323 | 0.4356 | 0.0934 | 0.2427 | 0.4356 |

ML ratio (df=14 –13=1): 0.7507

***ML test Ctrl vs. Selection (r1, r2 and c are all line-specific)**

| | | | | | | |
|---|---|---|---|---|---|---|
| Line1[r1,r2,c]: | 0.1040 | 0.2773 | 0.3698 | 0.1080 | 0.2787 | 0.3987 |
| Line2[r1,r2,c]: | 0.0947 | 0.2893 | 0.4381 | 0.1000 | 0.2413 | 0.6630 |
| Line3[r1,r2,c]: | 0.0987 | 0.2320 | 0.3495 | 0.0933 | 0.2427 | 0.4121 |

ML ratio (df=18 –13=5): 2.9803

ML ratio for heterogeneity (control+selection; df = 5–1=4): 2.9803 – 0.7507

…………………………………………………………………………………………………………

**markers 1-2-5 (*cn-kn-sp*)**

**CONTROL** **HYPEROXIA**

**Lines (ML estimates)**

| | | |
|---|---|---|
| Line1 [r1,r2,c]: | 0.1040 0.3347 0.3065 | 0.1080 0.3387 0.6562 |
| Line2 [r1,r2,c]: | 0.0947 0.3560 0.3956 | 0.1000 0.3267 0.9796 |
| Line3 [r1,r2,c]: | 0.0987 0.3013 0.6278 | 0.0933 0.2933 0.4870 |

Bailey test for 3 × 750

| | | |
|---|---|---|
| Teta [r1,r2,c]: | 0.0995 0.3300 0.4027 | 0.1000 0.3192 0.6856 |
| SE [r1,r2,c]: | 0.0063 0.0099 0.0668 | 0.0063 0.0098 0.0813 |
| Chi2: | 9.1408 | 10.5879 |
| C Chi2: | 3.3169 | 5.6325 |

Bailey for 6 × 750:

Teta: 0.0999 0.3247 0.5161

Chi2: 27.7464

C chi2: 16.0536

**ML test Control vs. selection**

chi^2(ctrl + sel) - chi^2(ctrl) - chi^2(sel): 8.0178

C chi^2(ctrl + sel) - chi^2(ctrl) - chi^2(sel): **7.1042**

**ML test Ctrl Vs. Selection**

| | | |
|---|---|---|
| ML est [r1,r2,c] | 0.0991 0.3307 0.4340 | 0.1004 0.3196 0.7200 |
| ML SE [r1,r2,c] | 0.0063 0.0099 0.0691 | 0.0063 0.0098 0.0840 |

**ML test Ctrl vs. Selection (r1 and r2 are line-specific)**

c - control or selection specific

| | | | | | | |
|---|---|---|---|---|---|---|
| Line1[r1,r2,c]: | 0.1043 | 0.3357 | 0.4329 | 0.1081 | 0.3390 | 0.7195 |
| Line2[r1,r2,c]: | 0.0947 | 0.3563 | 0.4329 | 0.0997 | 0.3256 | 0.7195 |
| Line3[r1,r2,c]: | 0.0983 | 0.3001 | 0.4329 | 0.0936 | 0.2940 | 0.7195 |

c-global

| | | | | | | |
|---|---|---|---|---|---|---|
| Line1[r1,r2,c]: | 0.1045 | 0.3364 | 0.5740 | 0.1078 | 0.3381 | 0.5740 |
| Line2[r1,r2,c]: | 0.0950 | 0.3572 | 0.5740 | 0.0993 | 0.3243 | 0.5740 |
| Line3[r1,r2,c]: | 0.0986 | 0.3011 | 0.5740 | 0.0934 | 0.2937 | 0.5740 |

ML ratio (df=14 –13=1): 6.9363

***ML test Ctrl vs. Selection (r1, r2 and c are all line-specific)**

| | | | | | | |
|---|---|---|---|---|---|---|
| Line1[r1,r2,c]: | 0.1040 | 0.3347 | 0.3065 | 0.1080 | 0.3387 | 0.6562 |
| Line2[r1,r2,c]: | 0.0947 | 0.3560 | 0.3956 | 0.1000 | 0.3267 | 0.9796 |
| Line3[r1,r2,c]: | 0.0987 | 0.3013 | 0.6278 | 0.0933 | 0.2933 | 0.4870 |

ML ratio (df=18 –13=5): 16.0716

ML ratio for heterogeneity (control+selection; df = 5–1=4): 16.0716 – 6.9363

……………………………………………………………………………………………………

**markers 1-3-4 (*cn-c-px*)**

| | CONTROL | | | HYPEROXIA | | |
|---|---|---|---|---|---|---|
| **Lines (ML estimates)** | | | | | | |
| Line1 [r1,r2,c]: | 0.1267 | 0.2547 | 0.3307 | 0.1347 | 0.2600 | 0.4570 |
| Line2 [r1,r2,c]: | 0.1160 | 0.2707 | 0.4247 | 0.1200 | 0.2240 | 0.6448 |
| Line3 [r1,r2,c]: | 0.1147 | 0.2187 | 0.3722 | 0.1200 | 0.2213 | 0.4518 |
| Bailey test for 3 × 750 | | | | | | |
| Teta [r1,r2,c]: | 0.1191 | 0.2468 | 0.3706 | 0.1248 | 0.2344 | 0.4990 |
| SE [r1,r2,c]: | 0.0068 | 0.0091 | 0.0693 | 0.0070 | 0.0089 | 0.0785 |
| Chi2: | | 7.0784 | | | 6.4316 | |
| C Chi2: | | 0.3296 | | | 1.0910 | |

Bailey for 6 × 750:

| | | | |
|---|---|---|---|
| Teta: | 0.1220 | 0.2404 | 0.4266 |
| Chi2: | 16.2410 | | |
| C chi2: | 2.9491 | | |

**ML test Control vs. selection**

chi^2(ctrl + sel) - chi^2(ctrl) - chi^2(sel): 2.7310

C chi^2(ctrl + sel) - chi^2(ctrl) - chi^2(sel): **1.5286**

**ML test Ctrl Vs. Selection**

| | | | | | | |
|---|---|---|---|---|---|---|
| ML est [r1,r2,c] | 0.1191 | 0.2480 | 0.3761 | 0.1249 | 0.2351 | 0.5146 |
| ML SE [r1,r2,c] | 0.0068 | 0.0091 | 0.0697 | 0.0070 | 0.0089 | 0.0797 |

**ML test Ctrl vs. Selection (r1 and r2 are line-specific)**

| | | | | | | |
|---|---|---|---|---|---|---|
| c - control or selection specific | | | | | | |
| Line1[r1,r2,c]: | 0.1268 | 0.2549 | 0.3757 | 0.1348 | 0.2603 | 0.5119 |
| Line2[r1,r2,c]: | 0.1159 | 0.2704 | 0.3757 | 0.1197 | 0.2235 | 0.5119 |
| Line3[r1,r2,c]: | 0.1147 | 0.2187 | 0.3757 | 0.1201 | 0.2215 | 0.5119 |
| c-global | | | | | | |
| Line1[r1,r2,c]: | 0.1270 | 0.2553 | 0.4436 | 0.1346 | 0.2599 | 0.4436 |
| Line2[r1,r2,c]: | 0.1160 | 0.2708 | 0.4436 | 0.1196 | 0.2232 | 0.4436 |
| Line3[r1,r2,c]: | 0.1148 | 0.2189 | 0.4436 | 0.1200 | 0.2213 | 0.4436 |

ML ratio (df=14 –13=1): 1.6653

***ML test Ctrl vs. Selection (r1, r2 and c are all line-specific)**

| | | | | | | |
|---|---|---|---|---|---|---|
| Line1[r1,r2,c]: | 0.1267 | 0.2547 | 0.3307 | 0.1347 | 0.2600 | 0.4570 |
| Line2[r1,r2,c]: | 0.1160 | 0.2707 | 0.4247 | 0.1200 | 0.2240 | 0.6448 |
| Line3[r1,r2,c]: | 0.1147 | 0.2187 | 0.3722 | 0.1200 | 0.2213 | 0.4518 |

ML ratio (df=18 –13=5): 3.1626

ML ratio for heterogeneity (control+selection; df = 5–1=4): 3.1626 – 1.6653
……………………………………………………………………………………………………

**markers 1-3-5 (*cn-c-sp*)**

| | CONTROL | | | HYPEROXIA | | |
|---|---|---|---|---|---|---|
| **Lines (ML estimates)** | | | | | | |
| Line1 [r1,r2,c]: | 0.1267 | 0.3120 | 0.2699 | 0.1347 | 0.3200 | 0.6498 |
| Line2 [r1,r2,c]: | 0.1160 | 0.3373 | 0.3748 | 0.1200 | 0.3093 | 0.8980 |
| Line3 [r1,r2,c]: | 0.1147 | 0.2907 | 0.6401 | 0.1200 | 0.2747 | 0.5259 |
| Bailey test for 3 × 750 | | | | | | |
| Teta [r1,r2,c]: | 0.1194 | 0.3130 | 0.3753 | 0.1244 | 0.3009 | 0.6756 |
| SE [r1,r2,c]: | 0.0068 | 0.0098 | 0.0606 | 0.0070 | 0.0096 | 0.0751 |
| Chi2: | | 9.8249 | | | 9.0066 | |
| C Chi2: | | 5.0729 | | | 3.7710 | |

Bailey for 6 × 750:

| | | | |
|---|---|---|---|
| Teta: | 0.1220 | 0.3069 | 0.4933 |
| Chi2: | 29.6637 | | |
| C chi2: | 18.4459 | | |

**ML test Control vs. selection**

chi^2(ctrl + sel) - chi^2(ctrl) - chi^2(sel): 10.8322

C chi^2(ctrl + sel) - chi^2(ctrl) - chi^2(sel): **9.6020**

**ML test Ctrl Vs. Selection**

| | | | | | | |
|---|---|---|---|---|---|---|
| ML est [r1,r2,c] | 0.1191 | 0.3133 | 0.4168 | 0.1249 | 0.3013 | 0.6968 |
| ML SE [r1,r2,c] | 0.0068 | 0.0098 | 0.0637 | 0.0070 | 0.0097 | 0.0766 |

**ML test Ctrl vs. Selection (r1 and r2 are line-specific)**

| | | | | | | |
|---|---|---|---|---|---|---|
| c - control or selection specific | | | | | | |
| Line1[r1,r2,c]: | 0.1272 | 0.3133 | 0.4153 | 0.1348 | 0.3203 | 0.6961 |
| Line2[r1,r2,c]: | 0.1161 | 0.3377 | 0.4153 | 0.1196 | 0.3083 | 0.6961 |
| Line3[r1,r2,c]: | 0.1141 | 0.2891 | 0.4153 | 0.1203 | 0.2753 | 0.6961 |
| c-global | | | | | | |
| Line1[r1,r2,c]: | 0.1275 | 0.3141 | 0.5557 | 0.1343 | 0.3192 | 0.5557 |
| Line2[r1,r2,c]: | 0.1165 | 0.3387 | 0.5557 | 0.1191 | 0.3071 | 0.5557 |
| Line3[r1,r2,c]: | 0.1145 | 0.2902 | 0.5557 | 0.1201 | 0.2748 | 0.5557 |

ML ratio: 7.9238

***ML test Ctrl vs. Selection (r1, r2 and c are all line-specific)**

| | | | | | | |
|---|---|---|---|---|---|---|
| Line1[r1,r2,c]: | 0.1267 | 0.3120 | 0.2699 | 0.1347 | 0.3200 | 0.6498 |
| Line2[r1,r2,c]: | 0.1160 | 0.3373 | 0.3748 | 0.1200 | 0.3093 | 0.8980 |
| Line3[r1,r2,c]: | 0.1147 | 0.2907 | 0.6401 | 0.1200 | 0.2747 | 0.5259 |

ML ratio (df=14 –13=1): 17.1806

ML ratio for heterogeneity (control+selection; df = 5–1=4): 17.1806 – 7.9238

…………………………………………………………………………………………………………

**markers 2-3-4 (*kn-c-px*)**

| | CONTROL | | | HYPEROXIA | | |
|---|---|---|---|---|---|---|
| **Lines (ML estimates)** | | | | | | |
| Line1 [r1,r2,c]: | 0.0253 | 0.2547 | 0.2066 | 0.0267 | 0.2600 | 0.5779 |
| Line2 [r1,r2,c]: | 0.0213 | 0.2707 | 0.2306 | 0.0200 | 0.2240 | 0.2990 |
| Line3 [r1,r2,c]: | 0.0160 | 0.2186 | 0.3786 | 0.0267 | 0.2213 | 0.4524 |
| Bailey test for 3 × 750 | | | | | | |
| Teta [r1,r2,c]: | 0.0202 | 0.2469 | 0.2383 | 0.0240 | 0.2342 | 0.4369 |
| SE [r1,r2,c]: | 0.0030 | 0.0091 | 0.1378 | 0.0032 | 0.0089 | 0.1717 |
| Chi2: | | 8.0691 | | | 5.2986 | |
| C Chi2: | | 0.1759 | | | 0.4477 | |

Bailey for 6 × 750:

| | | | |
|---|---|---|---|
| Teta: | 0.0220 | 0.2404 | 0.3160 |
| Chi2: | 15.8314 | | |
| C chi2: | 1.4226 | | |

**ML test Control vs. selection**

chi^2(ctrl + sel) - chi^2(ctrl) - chi^2(sel): 2.4637

C chi^2(ctrl + sel) - chi^2(ctrl) - chi^2(sel): 0.7991

**ML test Ctrl Vs. Selection**

| | | | | | | |
|---|---|---|---|---|---|---|
| ML est [r1,r2,c] | 0.0209 | 0.2480 | 0.2571 | 0.0245 | 0.2350 | 0.4664 |
| ML SE [r1,r2,c] | 0.0030 | 0.0091 | 0.1433 | 0.0033 | 0.0089 | 0.1780 |

**ML test Ctrl vs. Selection (r1 and r2 are line-specific)**

| | | | | | | |
|---|---|---|---|---|---|---|
| c - control or selection specific | | | | | | |
| Line1[r1,r2,c]: | 0.0253 | 0.2547 | 0.2571 | 0.0266 | 0.2599 | 0.4646 |
| Line2[r1,r2,c]: | 0.0213 | 0.2707 | 0.2571 | 0.0200 | 0.2241 | 0.4646 |
| Line3[r1,r2,c]: | 0.0160 | 0.2186 | 0.2571 | 0.0267 | 0.2213 | 0.4646 |
| c-global | | | | | | |
| Line1[r1,r2,c]: | 0.0254 | 0.2548 | 0.3661 | 0.0266 | 0.2597 | 0.3661 |
| Line2[r1,r2,c]: | 0.0214 | 0.2708 | 0.3661 | 0.0200 | 0.2240 | 0.3661 |
| Line3[r1,r2,c]: | 0.0160 | 0.2186 | 0.3661 | 0.0267 | 0.2212 | 0.3661 |

ML ratio (df=14 –13=1): 0.8417

***ML test Ctrl vs. Selection (r1, r2 and c are all line-specific)**

| | | | | | | |
|---|---|---|---|---|---|---|
| Line1[r1,r2,c]: | 0.0253 | 0.2547 | 0.2066 | 0.0267 | 0.2600 | 0.5779 |
| Line2[r1,r2,c]: | 0.0213 | 0.2707 | 0.2306 | 0.0200 | 0.2240 | 0.2990 |
| Line3[r1,r2,c]: | 0.0160 | 0.2186 | 0.3786 | 0.0267 | 0.2213 | 0.4524 |

ML ratio (df=18 –13=5): 1.4679

ML ratio for heterogeneity (control+selection; df = 5–1=4): 1.4679 – 0.8417
…………………………………………………………………………………………………………

**markers 2-3-5 (*kn-c-sp*)**

| | CONTROL | | | HYPEROXIA | | |
|---|---|---|---|---|---|---|
| **Lines (ML estimates)** | | | | | | |
| Line1 [r1,r2,c]: | 0.0253 | 0.3120 | 0.1688 | 0.0267 | 0.3200 | 0.4690 |
| Line2 [r1,r2,c]: | 0.0213 | 0.3373 | 0.1855 | 0.0200 | 0.3093 | 0.2161 |
| Line3 [r1,r2,c]: | 0.0160 | 0.2906 | 0.5724 | 0.0267 | 0.2746 | 0.5463 |
| Bailey test for 3 × 750 | | | | | | |
| Teta [r1,r2,c]: | 0.0203 | 0.3131 | 0.2130 | 0.0241 | 0.3005 | 0.3722 |
| SE [r1,r2,c]: | 0.0030 | 0.0098 | 0.1148 | 0.0032 | 0.0097 | 0.1394 |
| Chi2: | | 6.7693 | | | 6.1809 | |
| C Chi2: | | 1.0504 | | | 1.0816 | |

Bailey for 6 × 750:

| | | | |
|---|---|---|---|
| Teta: | 0.0220 | 0.3067 | 0.2772 |
| Chi2: | 15.2477 | | |
| C chi2: | 2.9026 | | |

**ML test Control vs. selection**

chi^2(ctrl + sel) - chi^2(ctrl) - chi^2(sel): 2.2975
C chi^2(ctrl + sel) - chi^2(ctrl) - chi^2(sel): **0.7706**

**ML test Ctrl Vs. Selection**

| | | | | | | |
|---|---|---|---|---|---|---|
| ML est [r1,r2,c] | 0.0209 | 0.3133 | 0.2716 | 0.0244 | 0.3013 | 0.4220 |
| ML SE [r1,r2,c] | 0.0030 | 0.0098 | 0.1294 | 0.0033 | 0.0097 | 0.1482 |

**ML test Ctrl vs. Selection (r1 and r2 are line-specific)**

c - control or selection specific

| | | | | | | |
|---|---|---|---|---|---|---|
| Line1[r1,r2,c]: | 0.0254 | 0.3122 | 0.2693 | 0.0267 | 0.3199 | 0.4242 |
| Line2[r1,r2,c]: | 0.0214 | 0.3375 | 0.2693 | 0.0200 | 0.3096 | 0.4242 |
| Line3[r1,r2,c]: | 0.0159 | 0.2903 | 0.2693 | 0.0267 | 0.2745 | 0.4242 |

c-global

| | | | | | | |
|---|---|---|---|---|---|---|
| Line1[r1,r2,c]: | 0.0254 | 0.3123 | 0.3514 | 0.0267 | 0.3197 | 0.3514 |
| Line2[r1,r2,c]: | 0.0214 | 0.3376 | 0.3514 | 0.0200 | 0.3095 | 0.3514 |
| Line3[r1,r2,c]: | 0.0160 | 0.2904 | 0.3514 | 0.0266 | 0.2744 | 0.3514 |

ML ratio (df=14 –13=1): 0.6082

***ML test Ctrl vs. Selection (r1, r2 and c are all line-specific)**

| | | | | | | |
|---|---|---|---|---|---|---|
| Line1[r1,r2,c]: | 0.0253 | 0.3120 | 0.1688 | 0.0267 | 0.3200 | 0.4690 |
| Line2[r1,r2,c]: | 0.0213 | 0.3373 | 0.1855 | 0.0200 | 0.3093 | 0.2161 |
| Line3[r1,r2,c]: | 0.0160 | 0.2906 | 0.5724 | 0.0267 | 0.2746 | 0.5463 |

ML ratio (df=18 –13=5): 2.9730

ML ratio for heterogeneity (control+selection; df = 5–1=4): 2.9730 – 0.6082

……………………………………………………………………………………………………

**markers 1-4-5 (*cn-px-sp*)**

| | CONTROL | | | HYPEROXIA | | |
|---|---|---|---|---|---|---|
| **Lines (ML estimates)** | | | | | | |
| Line1 [r1,r2,c]: | 0.3600 | 0.0707 | 0.2621 | 0.3627 | 0.0760 | 0.7256 |
| Line2 [r1,r2,c]: | 0.3600 | 0.0773 | 0.2395 | 0.3093 | 0.0880 | 0.6367 |
| Line3 [r1,r2,c]: | 0.3147 | 0.0800 | 0.6356 | 0.3173 | 0.0693 | 0.6061 |
| Bailey test for 3 × 750 | | | | | | |
| Teta [r1,r2,c]: | 0.3441 | 0.0763 | 0.3170 | 0.3290 | 0.0770 | 0.6580 |
| SE [r1,r2,c]: | 0.0100 | 0.0056 | 0.0675 | 0.0099 | 0.0056 | 0.0915 |
| Chi2: | | 10.2052 | | | 7.4604 | |
| C Chi2: | | 4.8468 | | | 0.3036 | |

Bailey for 6 × 750:

| | | | |
|---|---|---|---|
| Teta: | 0.3365 | 0.0768 | 0.4363 |
| Chi2: | 28.0748 | | |
| C chi2: | 14.0728 | | |

**ML test Control vs. selection**

chi^2(ctrl + sel) - chi^2(ctrl) - chi^2(sel): 10.4092

C chi^2(ctrl + sel) - chi^2(ctrl) - chi^2(sel): **8.9223**

**ML test Ctrl Vs. Selection**

| | | | | | | |
|---|---|---|---|---|---|---|
| ML est [r1,r2,c] | 0.3449 | 0.0760 | 0.3730 | 0.3298 | 0.0778 | 0.6585 |
| ML SE [r1,r2,c] | 0.0100 | 0.0056 | 0.0729 | 0.0099 | 0.0056 | 0.0917 |

**ML test Ctrl vs. Selection (r1 and r2 are line-specific)**

c - control or selection specific

| | | | | | | |
|---|---|---|---|---|---|---|
| Line1[r1,r2,c]: | 0.3608 | 0.0708 | 0.3723 | 0.3624 | 0.0759 | 0.6609 |
| Line2[r1,r2,c]: | 0.3610 | 0.0775 | 0.3723 | 0.3094 | 0.0880 | 0.6609 |
| Line3[r1,r2,c]: | 0.3131 | 0.0796 | 0.3723 | 0.3175 | 0.0694 | 0.6609 |

c-global

| | | | | | | |
|---|---|---|---|---|---|---|
| Line1[r1,r2,c]: | 0.3614 | 0.0709 | 0.5138 | 0.3613 | 0.0757 | 0.5138 |
| Line2[r1,r2,c]: | 0.3617 | 0.0777 | 0.5138 | 0.3087 | 0.0878 | 0.5138 |
| Line3[r1,r2,c]: | 0.3141 | 0.0798 | 0.5138 | 0.3169 | 0.0692 | 0.5138 |

ML ratio (df=14 –13=1): 6.0470

***ML test Ctrl vs. Selection (r1, r2 and c are all line-specific)**

| | | | | | | |
|---|---|---|---|---|---|---|
| Line1[r1,r2,c]: | 0.3600 | 0.0707 | 0.2621 | 0.3627 | 0.0760 | 0.7256 |
| Line2[r1,r2,c]: | 0.3600 | 0.0773 | 0.2395 | 0.3093 | 0.0880 | 0.6367 |
| Line3[r1,r2,c]: | 0.3147 | 0.0800 | 0.6356 | 0.3173 | 0.0693 | 0.6061 |

ML ratio (df=18 –13=5): 12.0238

ML ratio for heterogeneity (control+selection; df = 5–1=4): 12.0238 – 6.0470

…………………………………………………………………………………………………

**markers 2-4-5 (*kn-px-sp*)**

| | CONTROL | | | HYPEROXIA | | |
|---|---|---|---|---|---|---|
| **Lines (ML estimates)** | | | | | | |
| Line1 [r1,r2,c]: | 0.2773 | 0.0707 | 0.3402 | 0.2787 | 0.0760 | 0.3777 |
| Line2 [r1,r2,c]: | 0.2893 | 0.0773 | 0.2384 | 0.2413 | 0.0880 | 0.0628 |
| Line3 [r1,r2,c]: | 0.2320 | 0.0800 | 0.2874 | 0.2427 | 0.0693 | 0.5549 |
| Bailey test for 3 × 750 | | | | | | |
| Teta [r1,r2,c]: | 0.2647 | 0.0760 | 0.2794 | 0.2536 | 0.0770 | 0.1482 |
| SE [r1,r2,c]: | 0.0093 | 0.0056 | 0.0746 | 0.0092 | 0.0056 | 0.0546 |
| Chi2: | | 8.0576 | | | 14.0721 | |
| C Chi2: | | 0.3155 | | | 9.0106 | |

Bailey for 6 × 750:

| | | | |
|---|---|---|---|
| Teta: | 0.2590 | 0.0765 | 0.1939 |
| Chi2: | 24.8091 | | |
| C chi2: | 11.3654 | | |

**ML test Control vs. selection**

chi^2(ctrl + sel) - chi^2(ctrl) - chi^2(sel): 2.6794

C chi^2(ctrl + sel) - chi^2(ctrl) - chi^2(sel): **2.0392**

**ML test Ctrl Vs. Selection**

| | | | | | | |
|---|---|---|---|---|---|---|
| ML est [r1,r2,c] | 0.2662 | 0.0760 | 0.2856 | 0.2542 | 0.0778 | 0.3147 |
| ML SE [r1,r2,c] | 0.0093 | 0.0056 | 0.0751 | 0.0092 | 0.0056 | 0.0795 |

**ML test Ctrl vs. Selection (r1 and r2 are line-specific)**

c - control or selection specific

| | | | | | | |
|---|---|---|---|---|---|---|
| Line1[r1,r2,c]: | 0.2771 | 0.0706 | 0.2862 | 0.2784 | 0.0759 | 0.3148 |
| Line2[r1,r2,c]: | 0.2896 | 0.0774 | 0.2862 | 0.2423 | 0.0884 | 0.3148 |
| Line3[r1,r2,c]: | 0.2320 | 0.0800 | 0.2862 | 0.2419 | 0.0691 | 0.3148 |

c-global

| | | | | | | |
|---|---|---|---|---|---|---|
| Line1[r1,r2,c]: | 0.2772 | 0.0706 | 0.3004 | 0.2783 | 0.0759 | 0.3004 |
| Line2[r1,r2,c]: | 0.2897 | 0.0774 | 0.3004 | 0.2423 | 0.0883 | 0.3004 |
| Line3[r1,r2,c]: | 0.2320 | 0.0800 | 0.3004 | 0.2419 | 0.0691 | 0.3004 |

ML ratio (df=14 –13=1): 0.0681

***ML test Ctrl vs. Selection (r1, r2 and c are all line-specific)**

| | | | | | | |
|---|---|---|---|---|---|---|
| Line1[r1,r2,c]: | 0.2773 | 0.0707 | 0.3402 | 0.2787 | 0.0760 | 0.3777 |
| Line2[r1,r2,c]: | 0.2893 | 0.0773 | 0.2384 | 0.2413 | 0.0880 | 0.0628 |
| Line3[r1,r2,c]: | 0.2320 | 0.0800 | 0.2874 | 0.2427 | 0.0693 | 0.5549 |

ML ratio (df=18 –13=5): 7.9152

ML ratio for heterogeneity (control+selection; df = 5–1=4): 7.9152 – 0.0681

……………………………………………………………………………………………………

**markers 3-4-5 (*c-px-sp*)**

| | CONTROL | | | HYPEROXIA | | |
|---|---|---|---|---|---|---|
| **Lines (ML estimates)** | | | | | | |
| Line1 [r1,r2,c]: | 0.2547 | 0.0707 | 0.3705 | 0.2600 | 0.0760 | 0.4049 |
| Line2 [r1,r2,c]: | 0.2707 | 0.0773 | 0.2548 | 0.2240 | 0.0880 | 0.0676 |
| Line3 [r1,r2,c]: | 0.2187 | 0.0800 | 0.2287 | 0.2213 | 0.0693 | 0.5216 |
| Bailey test for 3 × 750 | | | | | | |
| Teta [r1,r2,c]: | 0.2467 | 0.0759 | 0.2725 | 0.2344 | 0.0770 | 0.1565 |
| SE [r1,r2,c]: | 0.0091 | 0.0056 | 0.0765 | 0.0089 | 0.0056 | 0.0585 |
| Chi2: | | 6.8203 | | | 13.3025 | |
| C Chi2: | | 0.5388 | | | 7.8563 | |

Bailey for 6 × 750:

| | | | |
|---|---|---|---|
| Teta: | 0.2404 | 0.0765 | 0.1992 |
| Chi2: | 22.4621 | | |
| C chi2: | 9.8557 | | |

**ML test Control vs. selection**

chi^2(ctrl + sel) - chi^2(ctrl) - chi^2(sel): 2.3393

C chi^2(ctrl + sel) - chi^2(ctrl) - chi^2(sel): **1.4606**

**ML test Ctrl Vs. Selection**

| | | | | | | |
|---|---|---|---|---|---|---|
| ML est [r1,r2,c] | 0.2480 | 0.0760 | 0.2830 | 0.2351 | 0.0778 | 0.3160 |
| ML SE [r1,r2,c] | 0.0091 | 0.0056 | 0.0778 | 0.0089 | 0.0056 | 0.0831 |

**ML test Ctrl vs. Selection (r1 and r2 are line-specific)**

c - control or selection specific

| | | | | | | |
|---|---|---|---|---|---|---|
| Line1[r1,r2,c]: | 0.2543 | 0.0706 | 0.2835 | 0.2596 | 0.0759 | 0.3159 |
| Line2[r1,r2,c]: | 0.2708 | 0.0774 | 0.2835 | 0.2248 | 0.0883 | 0.3159 |
| Line3[r1,r2,c]: | 0.2188 | 0.0801 | 0.2835 | 0.2208 | 0.0692 | 0.3159 |

c-global

| | | | | | | |
|---|---|---|---|---|---|---|
| Line1[r1,r2,c]: | 0.2544 | 0.0706 | 0.2996 | 0.2596 | 0.0759 | 0.2996 |
| Line2[r1,r2,c]: | 0.2709 | 0.0774 | 0.2996 | 0.2248 | 0.0883 | 0.2996 |
| Line3[r1,r2,c]: | 0.2189 | 0.0801 | 0.2996 | 0.2208 | 0.0691 | 0.2996 |

ML ratio (df=14 –13=1): 0.0806

***ML test Ctrl vs. Selection (r1, r2 and c are all line-specific)**

| | | | | | | |
|---|---|---|---|---|---|---|
| Line1[r1,r2,c]: | 0.2547 | 0.0707 | 0.3705 | 0.2600 | 0.0760 | 0.4049 |
| Line2[r1,r2,c]: | 0.2707 | 0.0773 | 0.2548 | 0.2240 | 0.0880 | 0.0676 |
| Line3[r1,r2,c]: | 0.2187 | 0.0800 | 0.2287 | 0.2213 | 0.0693 | 0.521 |

ML ratio (df=18 –13=5): 7.0675

ML ratio for heterogeneity (control+selection; df = 5–1=4): 7.0675 – 0.0806

……………………………………………………………………………………………………

# 3 chromosome

**markers 1-2-3 (*ru-h-th*)**

| | CONTROL | | | HYPOXIA | | |
|---|---|---|---|---|---|---|
| **Lines (ML estimates)** | | | | | | |
| Line1 [r1,r2,c]: | 0.1320 | 0.0987 | 0.4095 | 0.1667 | 0.1507 | 0.9026 |
| Line2 [r1,r2,c]: | 0.1587 | 0.1307 | 0.7074 | 0.1360 | 0.1253 | 0.7822 |
| Line3 [r1,r2,c]: | 0.1587 | 0.1307 | 0.3216 | 0.1440 | 0.1200 | 1.3116 |

Bailey test for 3 × 750

| | | | | | | |
|---|---|---|---|---|---|---|
| Teta [r1,r2,c]: | 0.1489 | 0.1182 | 0.4399 | 0.1484 | 0.1311 | 0.9543 |
| SE [r1,r2,c]: | 0.0075 | 0.0068 | 0.0966 | 0.0075 | 0.0071 | 0.1258 |
| Chi2: | | 11.6099 | | | 8.7532 | |
| C Chi2: | | 2.7373 | | | 2.5068 | |

Bailey for 6 × 750:

| | | | | | | |
|---|---|---|---|---|---|---|
| Teta: | 0.1486 | 0.1245 | 0.6315 | | | |
| Chi2: | 32.5328 | | | | | |
| C chi2: | 15.7802 | | | | | |

chi^2(ctrl + sel) - chi^2(ctrl) - chi^2(sel): 12.1696
C chi^2(ctrl + sel) - chi^2(ctrl) - chi^2(sel): **10.5362**

**ML test Ctrl Vs. Selection**

| | | | | | | |
|---|---|---|---|---|---|---|
| ML est [r1,r2,c] | 0.1498 | 0.1200 | 0.4946 | 0.1489 | 0.1320 | 0.9949 |
| ML SE [r1,r2,c] | 0.0075 | 0.0069 | 0.1029 | 0.0075 | 0.0071 | 0.1290 |

**ML test Ctrl vs. Selection (r1 and r2 are line-specific)**

c - control or selection specific

| | | | | | | |
|---|---|---|---|---|---|---|
| Line1[r1,r2,c]: | 0.1321 | 0.0987 | 0.4899 | 0.1667 | 0.1507 | 0.9851 |
| Line2[r1,r2,c]: | 0.1582 | 0.1303 | 0.4899 | 0.1360 | 0.1253 | 0.9851 |
| Line3[r1,r2,c]: | 0.1590 | 0.1309 | 0.4899 | 0.1440 | 0.1200 | 0.9851 |

c-global

| | | | | | | |
|---|---|---|---|---|---|---|
| Line1[r1,r2,c]: | 0.1322 | 0.0988 | 0.7495 | 0.1665 | 0.1505 | 0.7495 |
| Line2[r1,r2,c]: | 0.1587 | 0.1307 | 0.7495 | 0.1360 | 0.1253 | 0.7495 |
| Line3[r1,r2,c]: | 0.1591 | 0.1310 | 0.7495 | 0.1436 | 0.1196 | 0.7495 |

ML ratio (df=14 –13=1): 8.9661

***ML test Ctrl vs. Selection (r1, r2 and c are all line-specific)**

| | | | | | | |
|---|---|---|---|---|---|---|
| Line1[r1,r2,c]: | 0.1320 | 0.0987 | 0.4095 | 0.1667 | 0.1507 | 0.9026 |
| Line2[r1,r2,c]: | 0.1587 | 0.1307 | 0.7074 | 0.1360 | 0.1253 | 0.7822 |
| Line3[r1,r2,c]: | 0.1587 | 0.1307 | 0.3216 | 0.1440 | 0.1200 | 1.3116 |

ML ratio (df=18 –13=5): 14.5199

ML ratio for heterogeneity (control+selection; df = 5–1=4): 14.5199 –8.9661

…………………………………………………………………………………………………………

**markers 1-2-4 (*ru-h-cu*)**

**CONTROL** **HYPOXIA**

**Lines (ML estimates)**

| | | | | | | |
|---|---|---|---|---|---|---|
| Line1 [r1,r2,c]: | 0.1320 | 0.1440 | 0.2806 | 0.1667 | 0.2160 | 0.6296 |
| Line2 [r1,r2,c]: | 0.1587 | 0.1973 | 0.5536 | 0.1360 | 0.1787 | 0.5487 |
| Line3 [r1,r2,c]: | 0.1587 | 0.1760 | 0.2865 | 0.1440 | 0.1720 | 0.9151 |

Bailey test for 3 × 750

| | | | | | | |
|---|---|---|---|---|---|---|
| Teta [r1,r2,c]: | 0.1488 | 0.1701 | 0.3572 | 0.1484 | 0.1877 | 0.6634 |
| SE [r1,r2,c]: | 0.0075 | 0.0079 | 0.0727 | 0.0075 | 0.0082 | 0.0898 |

| | | |
|---|---|---|
| Chi2: | 14.3972 | 11.1657 |
| C Chi2: | 2.7682 | 2.3549 |

Bailey for 6 × 750:

| | |
|---|---|
| Teta: | 0.1484 0.1786 0.4789 |
| Chi2: | 34.7681 |
| C chi2: | 12.2458 |

**ML test Control vs. selection**

chi^2(ctrl + sel) - chi^2(ctrl) - chi^2(sel): 9.2051
C chi^2(ctrl + sel) - chi^2(ctrl) - chi^2(sel): **7.1228**

**ML test Ctrl Vs. Selection**

| | | |
|---|---|---|
| ML est [r1,r2,c] | 0.1498 0.1724 0.3958 | 0.1489 0.1889 0.6953 |
| ML SE [r1,r2,c] | 0.0075 0.0080 0.0770 | 0.0075 0.0083 0.0922 |

**ML test Ctrl vs. Selection (r1 and r2 are line-specific)**

c - control or selection specific

| | | | | | | |
|---|---|---|---|---|---|---|
| Line1[r1,r2,c]: | 0.1322 | 0.1442 | 0.3933 | 0.1668 | 0.2162 | 0.6882 |
| Line2[r1,r2,c]: | 0.1581 | 0.1966 | 0.3933 | 0.1362 | 0.1789 | 0.6882 |
| Line3[r1,r2,c]: | 0.1590 | 0.1764 | 0.3933 | 0.1437 | 0.1716 | 0.6882 |

c-global

| | | | | | | |
|---|---|---|---|---|---|---|
| Line1[r1,r2,c]: | 0.1324 | 0.1444 | 0.5481 | 0.1664 | 0.2156 | 0.5481 |
| Line2[r1,r2,c]: | 0.1586 | 0.1973 | 0.5481 | 0.1360 | 0.1787 | 0.5481 |
| Line3[r1,r2,c]: | 0.1593 | 0.1767 | 0.5481 | 0.1433 | 0.1711 | 0.5481 |

ML ratio (df=14 –13=1): 6.0414

***ML test Ctrl vs. Selection (r1, r2 and c are all line-specific)**

| | | | | | | |
|---|---|---|---|---|---|---|
| Line1[r1,r2,c]: | 0.1320 | 0.1440 | 0.2806 | 0.1667 | 0.2160 | 0.6296 |
| Line2[r1,r2,c]: | 0.1587 | 0.1973 | 0.5536 | 0.1360 | 0.1787 | 0.5487 |
| Line3[r1,r2,c]: | 0.1587 | 0.1760 | 0.2865 | 0.1440 | 0.1720 | 0.9151 |

ML ratio (df=18 –13=5): 11.5026

ML ratio for heterogeneity (control+selection; df = 5–1=4): 11.5026 –6.0414

……………………………………………………………………………………………………………

**markers 1-2-5 (*ru-h-sr*)**

| | CONTROL | HYPOXIA |
|---|---|---|
| **Lines (ML estimates)** | | |
| Line1 [r1,r2,c]: | 0.1320 0.1920 0.2104 | 0.1667 0.2960 0.6486 |

| | | |
|---|---|---|
| Line2 [r1,r2,c]: | 0.1587 0.2667 0.5042 | 0.1360 0.2533 0.6192 |
| Line3 [r1,r2,c]: | 0.1587 0.2347 0.2149 | 0.1440 0.2373 0.8973 |

Bailey test for 3 × 750

| | | |
|---|---|---|
| Teta [r1,r2,c]: | 0.1487 0.2280 0.2833 | 0.1486 0.2612 0.6930 |
| SE [r1,r2,c]: | 0.0075 0.0088 0.0559 | 0.0075 0.0092 0.0752 |
| Chi2: | 21.5049 | 12.9978 |
| C Chi2: | 5.1127 | 2.2052 |

Bailey for 6 × 750:

| | |
|---|---|
| Teta: | 0.1481 0.2438 0.4300 |
| Chi2: | 59.6148 |
| C chi2: | 26.7281 |

**ML test Control vs. selection**

chi^2(ctrl + sel) - chi^2(ctrl) - chi^2(sel): 25.1122

C chi^2(ctrl + sel) - chi^2(ctrl) - chi^2(sel): **19.4102**

**ML test Ctrl Vs. Selection**

| | | |
|---|---|---|
| ML est [r1,r2,c] | 0.1498 0.2311 0.3338 | 0.1489 0.2622 0.7172 |
| ML SE [r1,r2,c] | 0.0075 0.0089 0.0610 | 0.0075 0.0093 0.0765 |

**ML test Ctrl vs. Selection (r1 and r2 are line-specific)**

c - control or selection specific

| | | | | | | |
|---|---|---|---|---|---|---|
| Line1[r1,r2,c]: | 0.1323 | 0.1924 | 0.3322 | 0.1669 | 0.2964 | 0.7098 |
| Line2[r1,r2,c]: | 0.1578 | 0.2652 | 0.3322 | 0.1362 | 0.2536 | 0.7098 |
| Line3[r1,r2,c]: | 0.1592 | 0.2355 | 0.3322 | 0.1436 | 0.2367 | 0.7098 |

c-global

| | | | | | | |
|---|---|---|---|---|---|---|
| Line1[r1,r2,c]: | 0.1326 | 0.1929 | 0.5345 | 0.1661 | 0.2950 | 0.5345 |
| Line2[r1,r2,c]: | 0.1588 | 0.2669 | 0.5345 | 0.1358 | 0.2529 | 0.5345 |
| Line3[r1,r2,c]: | 0.1598 | 0.2363 | 0.5345 | 0.1430 | 0.2356 | 0.5345 |

ML ratio (df=14 –13=1): 14.5883

***ML test Ctrl vs. Selection (r1, r2 and c are all line-specific)**

| | | | | | | |
|---|---|---|---|---|---|---|
| Line1[r1,r2,c]: | 0.1320 | 0.1920 | 0.2104 | 0.1667 | 0.2960 | 0.6486 |
| Line2[r1,r2,c]: | 0.1587 | 0.2667 | 0.5042 | 0.1360 | 0.2533 | 0.6192 |
| Line3[r1,r2,c]: | 0.1587 | 0.2347 | 0.2149 | 0.1440 | 0.2373 | 0.8973 |

ML ratio (df=18 –13=5): 22.4311

ML ratio for heterogeneity (control+selection; df = 5–1=4): 22.4311 – 14.5883

……………………………………………………………………………………………………

**markers 1-2-6 (*ru-h-e*)**

| | CONTROL | HYPOXIA |
|---|---|---|
| **Lines (ML estimates)** | | |
| Line1 [r1,r2,c]: | 0.1320 0.2320 0.2612 | 0.1667 0.3507 0.5932 |
| Line2 [r1,r2,c]: | 0.1587 0.3107 0.4328 | 0.1360 0.3147 0.6231 |
| Line3 [r1,r2,c]: | 0.1587 0.2813 0.2091 | 0.1440 0.3013 0.7067 |

Bailey test for 3 × 750

| | CONTROL | HYPOXIA |
|---|---|---|
| Teta [r1,r2,c]: | 0.1490 0.2723 0.2818 | 0.1484 0.3220 0.6288 |
| SE [r1,r2,c]: | 0.0075 0.0094 0.0511 | 0.0075 0.0098 0.0642 |
| Chi2: | 20.5299 | 8.9717 |
| C Chi2: | 3.4525 | 0.5392 |

Bailey for 6 × 750:
Teta: 0.1480 0.2956 0.4169
Chi2: 59.3508
C chi2: 21.9839

**ML test Control vs. selection**
chi^2(ctrl + sel) - chi^2(ctrl) - chi^2(sel): 29.8492
C chi^2(ctrl + sel) - chi^2(ctrl) - chi^2(sel): **17.9922**

**ML test Ctrl Vs. Selection**

| | CONTROL | HYPOXIA |
|---|---|---|
| ML est [r1,r2,c] | 0.1498 0.2747 0.3133 | 0.1489 0.3222 0.6392 |
| ML SE [r1,r2,c] | 0.0075 0.0094 0.0540 | 0.0075 0.0099 0.0647 |

**ML test Ctrl vs. Selection (r1 and r2 are line-specific)**

c - control or selection specific

| | | | | | | |
|---|---|---|---|---|---|---|
| Line1[r1,r2,c]: | 0.1322 | 0.2323 | 0.3113 | 0.1669 | 0.3511 | 0.6350 |
| Line2[r1,r2,c]: | 0.1579 | 0.3092 | 0.3113 | 0.1360 | 0.3147 | 0.6350 |
| Line3[r1,r2,c]: | 0.1592 | 0.2824 | 0.3113 | 0.1438 | 0.3009 | 0.6350 |

c-global

| | | | | | | |
|---|---|---|---|---|---|---|
| Line1[r1,r2,c]: | 0.1326 | 0.2330 | 0.4876 | 0.1660 | 0.3492 | 0.4876 |
| Line2[r1,r2,c]: | 0.1589 | 0.3112 | 0.4876 | 0.1355 | 0.3135 | 0.4876 |
| Line3[r1,r2,c]: | 0.1600 | 0.2836 | 0.4876 | 0.1431 | 0.2995 | 0.4876 |

ML ratio (df=14 –13=1): 14.3059

***ML test Ctrl vs. Selection (r1, r2 and c are all line-specific)**

| | | | | | | |
|---|---|---|---|---|---|---|
| Line1[r1,r2,c]: | 0.1320 | 0.2320 | 0.2612 | 0.1667 | 0.3507 | 0.5932 |
| Line2[r1,r2,c]: | 0.1587 | 0.3107 | 0.4328 | 0.1360 | 0.3147 | 0.6231 |
| Line3[r1,r2,c]: | 0.1587 | 0.2813 | 0.2091 | 0.1440 | 0.3013 | 0.7067 |

ML ratio (df=18 –13=5): 18.4059

ML ratio for heterogeneity (control+selection; df = 5–1=4): 18.4059 –14.3059

…………………………………………………………………………………………………………

**markers 1-3-4 (*ru-th-cu*)**

| | CONTROL | HYPOXIA |
|---|---|---|
| **Lines (ML estimates)** | | |
| Line1 [r1,r2,c]: | 0.2200 0.0453 0.0000 | 0.2720 0.0840 0.4085 |
| Line2 [r1,r2,c]: | 0.2600 0.0693 0.2219 | 0.2347 0.0560 0.1015 |
| Line3 [r1,r2,c]: | 0.2760 0.0480 0.2013 | 0.2187 0.0627 0.3891 |
| Bailey test for 3 × 750 | | |
| Teta [r1,r2,c]: | 0.2503 0.0523 0.0000 | 0.2403 0.0657 0.2312 |
| SE [r1,r2,c]: | 0.0091 0.0047 0.0005 | 0.0090 0.0052 0.0747 |
| Chi2: | 17.1457 | 15.5122 |
| C Chi2: | 5.3936 | 3.9776 |

Bailey for 6 × 750:
Teta: 0.2449 0.0583 0.0000
Chi2: 46.1720
C chi2: 19.1481

**ML test Control vs. selection**
chi^2(ctrl + sel) - chi^2(ctrl) - chi^2(sel): 13.5141
C chi^2(ctrl + sel) - chi^2(ctrl) - chi^2(sel): **9.7769**

**ML test Ctrl Vs. Selection**

| | CONTROL | HYPOXIA |
|---|---|---|
| ML est [r1,r2,c] | 0.2520 0.0542 0.1626 | 0.2418 0.0676 0.3265 |
| ML SE [r1,r2,c] | 0.0092 0.0048 0.0708 | 0.0090 0.0053 0.0893 |

**ML test Ctrl vs. Selection (r1 and r2 are line-specific)**

| | | | | | | |
|---|---|---|---|---|---|---|
| c - control or selection specific | | | | | | |
| Line1[r1,r2,c]: | 0.2203 | 0.0454 | 0.1621 | 0.2716 | 0.0839 | 0.3228 |
| Line2[r1,r2,c]: | 0.2597 | 0.0693 | 0.1621 | 0.2352 | 0.0561 | 0.3228 |
| Line3[r1,r2,c]: | 0.2759 | 0.0480 | 0.1621 | 0.2185 | 0.0626 | 0.3228 |
| c-global | | | | | | |
| Line1[r1,r2,c]: | 0.2204 | 0.0454 | 0.2501 | 0.2712 | 0.0837 | 0.2501 |
| Line2[r1,r2,c]: | 0.2601 | 0.0694 | 0.2501 | 0.2350 | 0.0561 | 0.2501 |
| Line3[r1,r2,c]: | 0.2761 | 0.0480 | 0.2501 | 0.2183 | 0.0626 | 0.2501 |

ML ratio (df=14 –13=1): 1.9668

**<u>*ML test Ctrl vs. Selection (r1, r2 and c are all line-specific)</u>**

| | | | | | | |
|---|---|---|---|---|---|---|
| Line1[r1,r2,c]: | 0.2200 | 0.0453 | 0.0000 | 0.2720 | 0.0840 | 0.4085 |
| Line2[r1,r2,c]: | 0.2600 | 0.0693 | 0.2219 | 0.2347 | 0.0560 | 0.1015 |
| Line3[r1,r2,c]: | 0.2760 | 0.0480 | 0.2013 | 0.2187 | 0.0627 | 0.3891 |

ML ratio (df=18 –13=5): 7.5857

ML ratio for heterogeneity (control+selection; df = 5–1=4): 7.5857 – 1.9668
…………………………………………………………………………………………………

**<u>markers 1-3-5 (*ru-th-sr*)</u>**

| | CONTROL | | | HYPOXIA | | |
|---|---|---|---|---|---|---|
| **<u>Lines (ML estimates)</u>** | | | | | | |
| Line1 [r1,r2,c]: | 0.2200 | 0.0960 | 0.0631 | 0.2720 | 0.1773 | 0.5252 |
| Line2 [r1,r2,c]: | 0.2600 | 0.1387 | 0.2219 | 0.2347 | 0.1387 | 0.4097 |
| Line3 [r1,r2,c]: | 0.2760 | 0.1067 | 0.0906 | 0.2187 | 0.1413 | 0.6471 |
| Bailey test for 3 × 750 | | | | | | |
| Teta [r1,r2,c]: | 0.2507 | 0.1116 | 0.1056 | 0.2408 | 0.1513 | 0.5082 |
| SE [r1,r2,c]: | 0.0091 | 0.0066 | 0.0394 | 0.0090 | 0.0075 | 0.0695 |
| Chi2: | | 17.3747 | | | 14.1564 | |
| C Chi2: | | 2.3425 | | | 1.6077 | |

Bailey for 6 × 750:

| | | | |
|---|---|---|---|
| Teta: | 0.2446 | 0.1287 | 0.2033 |
| Chi2: | 71.7115 | | |
| C chi2: | 29.8357 | | |

**<u>ML test Control vs. selection</u>**
chi^2(ctrl + sel) - chi^2(ctrl) - chi^2(sel): 40.1805
C chi^2(ctrl + sel) - chi^2(ctrl) - chi^2(sel): **<u>25.8855</u>**

**<u>ML test Ctrl Vs. Selection</u>**

| | | | | | | |
|---|---|---|---|---|---|---|
| ML est [r1,r2,c] | 0.2520 | 0.1138 | 0.1395 | 0.2418 | 0.1524 | 0.5306 |
| ML SE [r1,r2,c] | 0.0092 | 0.0067 | 0.0452 | 0.0090 | 0.0076 | 0.0712 |

**<u>ML test Ctrl vs. Selection (r1 and r2 are line-specific)</u>**

c - control or selection specific

| | | | | | | |
|---|---|---|---|---|---|---|
| Line1[r1,r2,c]: | 0.2203 | 0.0961 | 0.1388 | 0.2720 | 0.1773 | 0.5249 |
| Line2[r1,r2,c]: | 0.2593 | 0.1383 | 0.1388 | 0.2352 | 0.1390 | 0.5249 |
| Line3[r1,r2,c]: | 0.2764 | 0.1068 | 0.1388 | 0.2182 | 0.1410 | 0.5249 |

c-global

| | | | | | | |
|---|---|---|---|---|---|---|
| Line1[r1,r2,c]: | 0.2210 | 0.0964 | 0.3576 | 0.2703 | 0.1762 | 0.3576 |

| | | | | | | |
|---|---|---|---|---|---|---|
| Line2[r1,r2,c]: | 0.2610 | 0.1392 | 0.3576 | 0.2344 | 0.1385 | 0.3576 |
| Line3[r1,r2,c]: | 0.2776 | 0.1073 | 0.3576 | 0.2172 | 0.1404 | 0.3576 |

ML ratio (df=14 –13=1): 19.5690

***ML test Ctrl vs. Selection (r1, r2 and c are all line-specific)**

| | | | | | | |
|---|---|---|---|---|---|---|
| Line1[r1,r2,c]: | 0.2200 | 0.0960 | 0.0631 | 0.2720 | 0.1773 | 0.5252 |
| Line2[r1,r2,c]: | 0.2600 | 0.1387 | 0.2219 | 0.2347 | 0.1387 | 0.4097 |
| Line3[r1,r2,c]: | 0.2760 | 0.1067 | 0.0906 | 0.2187 | 0.1413 | 0.6471 |

ML ratio (df=18 –13=5): 23.6707

ML ratio for heterogeneity (control+selection; df = 5–1=4): 23.6707 – 19.5690
……………………………………………………………………………………………………

## markers 1-3-6 (*ru-th-e*)

| | CONTROL | | | HYPOXIA | | |
|---|---|---|---|---|---|---|
| **Lines (ML estimates)** | | | | | | |
| Line1 [r1,r2,c]: | 0.2200 | 0.1360 | 0.1337 | 0.2720 | 0.2347 | 0.4596 |
| Line2 [r1,r2,c]: | 0.2600 | 0.1880 | 0.2182 | 0.2347 | 0.2000 | 0.3977 |
| Line3 [r1,r2,c]: | 0.2760 | 0.1533 | 0.0945 | 0.2187 | 0.2107 | 0.4920 |
| Bailey test for 3 × 750 | | | | | | |
| Teta [r1,r2,c]: | 0.2510 | 0.1573 | 0.1344 | 0.2406 | 0.2148 | 0.4448 |
| SE [r1,r2,c]: | 0.0091 | 0.0077 | 0.0374 | 0.0090 | 0.0086 | 0.0548 |
| Chi2: | | 18.4370 | | | 10.6581 | |
| C Chi2: | | 1.8848 | | | 0.4693 | |

Bailey for 6 × 750:

| | | | |
|---|---|---|---|
| Teta: | 0.2449 | 0.1822 | 0.2321 |
| Chi2: | 74.0055 | | |
| C chi2: | 24.5428 | | |

**ML test Control vs. selection**
chi^2(ctrl + sel) - chi^2(ctrl) - chi^2(sel): 44.9104
C chi^2(ctrl + sel) - chi^2(ctrl) - chi^2(sel): **22.1887**

**ML test Ctrl Vs. Selection**

| | | | | | | |
|---|---|---|---|---|---|---|
| ML est [r1,r2,c] | 0.2520 | 0.1591 | 0.1552 | 0.2418 | 0.2151 | 0.4529 |
| ML SE [r1,r2,c] | 0.0092 | 0.0077 | 0.0400 | 0.0090 | 0.0087 | 0.0553 |

**ML test Ctrl vs. Selection (r1 and r2 are line-specific)**

c - control or selection specific

| | | | | | | |
|---|---|---|---|---|---|---|
| Line1[r1,r2,c]: | 0.2201 | 0.1361 | 0.1543 | 0.2719 | 0.2346 | 0.4508 |
| Line2[r1,r2,c]: | 0.2593 | 0.1875 | 0.1543 | 0.2351 | 0.2003 | 0.4508 |
| Line3[r1,r2,c]: | 0.2766 | 0.1537 | 0.1543 | 0.2184 | 0.2104 | 0.4508 |

c-global

| | | | | | | |
|---|---|---|---|---|---|---|
| Line1[r1,r2,c]: | 0.2210 | 0.1366 | 0.3228 | 0.2701 | 0.2330 | 0.3228 |
| Line2[r1,r2,c]: | 0.2611 | 0.1888 | 0.3228 | 0.2340 | 0.1994 | 0.3228 |
| Line3[r1,r2,c]: | 0.2781 | 0.1545 | 0.3228 | 0.2173 | 0.2094 | 0.3228 |

ML ratio (df=14 –13=1): 17.6066

***ML test Ctrl vs. Selection (r1, r2 and c are all line-specific)**

| | | | | | | |
|---|---|---|---|---|---|---|
| Line1[r1,r2,c]: | 0.2200 | 0.1360 | 0.1337 | 0.2720 | 0.2347 | 0.4596 |
| Line2[r1,r2,c]: | 0.2600 | 0.1880 | 0.2182 | 0.2347 | 0.2000 | 0.3977 |
| Line3[r1,r2,c]: | 0.2760 | 0.1533 | 0.0945 | 0.2187 | 0.2107 | 0.4920 |

ML ratio (df=18 –13=5): 19.9747

ML ratio for heterogeneity (control+selection; df = 5–1=4): 19.9747 –17.6066
.....................................................................................................

**markers 2-3-4 (*h-th-cu*)**

| | CONTROL | | | HYPOXIA | | |
|---|---|---|---|---|---|---|
| **Lines (ML estimates)** | | | | | | |
| Line1 [r1,r2,c]: | 0.0987 | 0.0453 | 0.0000 | 0.1506 | 0.0840 | 0.7379 |
| Line2 [r1,r2,c]: | 0.1307 | 0.0693 | 0.1472 | 0.1253 | 0.0560 | 0.1899 |
| Line3 [r1,r2,c]: | 0.1307 | 0.0480 | 0.2127 | 0.1200 | 0.0627 | 0.7091 |
| Bailey test for 3 × 750 | | | | | | |
| Teta [r1,r2,c]: | 0.1183 | 0.0524 | 0.0000 | 0.1307 | 0.0657 | 0.4372 |
| SE [r1,r2,c]: | 0.0068 | 0.0047 | 0.0003 | 0.0071 | 0.0052 | 0.1369 |
| Chi2: | | 12.3439 | | | 12.0842 | |
| C Chi2: | | 2.0724 | | | 3.8311 | |

Bailey for 6 × 750:

| | | | |
|---|---|---|---|
| Teta: | 0.1240 | 0.0582 | 0.0000 |
| Chi2: | 39.9496 | | |
| C chi2: | 16.1194 | | |

**ML test Control vs. selection**

chi^2(ctrl + sel) - chi^2(ctrl) - chi^2(sel): 15.5216

C chi^2(ctrl + sel) - chi^2(ctrl) - chi^2(sel): **10.2159**

**ML test Ctrl Vs. Selection**

| | | | | | | |
|---|---|---|---|---|---|---|
| ML est [r1,r2,c] | 0.1200 | 0.0542 | 0.1367 | 0.1320 | 0.0675 | 0.5979 |
| ML SE [r1,r2,c] | 0.0069 | 0.0048 | 0.0954 | 0.0071 | 0.0053 | 0.1621 |

**ML test Ctrl vs. Selection (r1 and r2 are line-specific)**

c - control or selection specific

| | | | | | | |
|---|---|---|---|---|---|---|
| Line1[r1,r2,c]: | 0.0987 | 0.0454 | 0.1348 | 0.1505 | 0.0839 | 0.5909 |
| Line2[r1,r2,c]: | 0.1306 | 0.0693 | 0.1348 | 0.1255 | 0.0561 | 0.5909 |
| Line3[r1,r2,c]: | 0.1306 | 0.0480 | 0.1348 | 0.1199 | 0.0626 | 0.5909 |

c-global

| | | | | | | |
|---|---|---|---|---|---|---|
| Line1[r1,r2,c]: | 0.0988 | 0.0454 | 0.3993 | 0.1502 | 0.0838 | 0.3993 |
| Line2[r1,r2,c]: | 0.1309 | 0.0694 | 0.3993 | 0.1254 | 0.0560 | 0.3993 |
| Line3[r1,r2,c]: | 0.1308 | 0.0480 | 0.3993 | 0.1198 | 0.0626 | 0.3993 |

ML ratio (df=14 –13=1): 5.4997

**<u>*ML test Ctrl vs. Selection (r1, r2 and c are all line-specific)</u>**

| | | | | | | |
|---|---|---|---|---|---|---|
| Line1[r1,r2,c]: | 0.0987 | 0.0453 | 0.0000 | 0.1506 | 0.0840 | 0.7379 |
| Line2[r1,r2,c]: | 0.1307 | 0.0693 | 0.1472 | 0.1253 | 0.0560 | 0.1899 |
| Line3[r1,r2,c]: | 0.1307 | 0.0480 | 0.2127 | 0.1200 | 0.0627 | 0.7091 |

ML ratio (df=18 –13=5): 9.2416

ML ratio for heterogeneity (control+selection; df = 5–1=4): 9.2416 – 5.4997

……………………………………………………………………………………………………

**<u>markers 2-3-5 (*h-th-sr*)</u>**

| | CONTROL | | | HYPOXIA | | |
|---|---|---|---|---|---|---|
| **<u>Lines (ML estimates)</u>** | | | | | | |
| Line1 [r1,r2,c]: | 0.0987 | 0.0960 | 0.1407 | 0.1507 | 0.1773 | 0.5988 |
| Line2 [r1,r2,c]: | 0.1307 | 0.1387 | 0.0736 | 0.1253 | 0.1387 | 0.3069 |
| Line3 [r1,r2,c]: | 0.1307 | 0.1067 | 0.0957 | 0.1200 | 0.1413 | 0.7077 |
| Bailey test for 3 × 750 | | | | | | |
| Teta [r1,r2,c]: | 0.1186 | 0.1118 | 0.0891 | 0.1309 | 0.1510 | 0.4950 |
| SE [r1,r2,c]: | 0.0068 | 0.0066 | 0.0533 | 0.0071 | 0.0075 | 0.0955 |
| Chi2: | | 13.5991 | | | 12.0320 | |
| C Chi2: | | 0.1887 | | | 3.0803 | |

Bailey for 6 × 750:

| | | | |
|---|---|---|---|
| Teta: | 0.1241 | 0.1285 | 0.1853 |
| Chi2: | 56.6158 | | |
| C chi2: | 17.1285 | | |

**<u>ML test Control vs. selection</u>**

chi^2(ctrl + sel) - chi^2(ctrl) - chi^2(sel): 30.9846

C chi^2(ctrl + sel) - chi^2(ctrl) - chi^2(sel): **<u>13.8595</u>**

**<u>ML test Ctrl Vs. Selection</u>**

| | | | | | | |
|---|---|---|---|---|---|---|
| ML est [r1,r2,c] | 0.1200 | 0.1138 | 0.0977 | 0.1320 | 0.1524 | 0.5521 |
| ML SE [r1,r2,c] | 0.0069 | 0.0067 | 0.0557 | 0.0071 | 0.0076 | 0.1015 |

**ML test Ctrl vs. Selection (r1 and r2 are line-specific)**

c - control or selection specific

| | | | | | | |
|---|---|---|---|---|---|---|
| Line1[r1,r2,c]: | 0.0986 | 0.0960 | 0.0962 | 0.1505 | 0.1772 | 0.5466 |
| Line2[r1,r2,c]: | 0.1307 | 0.1387 | 0.0962 | 0.1256 | 0.1390 | 0.5466 |
| Line3[r1,r2,c]: | 0.1307 | 0.1067 | 0.0962 | 0.1198 | 0.1411 | 0.5466 |

c-global

| | | | | | | |
|---|---|---|---|---|---|---|
| Line1[r1,r2,c]: | 0.0988 | 0.0961 | 0.3663 | 0.1500 | 0.1765 | 0.3663 |
| Line2[r1,r2,c]: | 0.1312 | 0.1392 | 0.3663 | 0.1254 | 0.1388 | 0.3663 |
| Line3[r1,r2,c]: | 0.1310 | 0.1069 | 0.3663 | 0.1195 | 0.1408 | 0.3663 |
| | 0.0988 | 0.0961 | 0.3663 | | | |
| ML ratio (df=14 –13=1): | 13.4519 | | | | | |

***ML test Ctrl vs. Selection (r1, r2 and c are all line-specific)**

| | | | | | | |
|---|---|---|---|---|---|---|
| Line1[r1,r2,c]: | 0.0987 | 0.0960 | 0.1407 | 0.1507 | 0.1773 | 0.5988 |
| Line2[r1,r2,c]: | 0.1307 | 0.1387 | 0.0736 | 0.1253 | 0.1387 | 0.3069 |
| Line3[r1,r2,c]: | 0.1307 | 0.1067 | 0.0957 | 0.1200 | 0.1413 | 0.7077 |

ML ratio (df=18 –13=5): 16.2923

ML ratio for heterogeneity (control+selection; df = 5–1=4): 16.2923 – 13.451
……………………………………………………………………………………………………

**markers 2-3-6 (*h-th-e*)**

**CONTROL** **HYPOXIA**

**Lines (ML estimates)**

| | | | | | | |
|---|---|---|---|---|---|---|
| Line1 [r1,r2,c]: | 0.0987 | 0.1360 | 0.0994 | 0.1507 | 0.2347 | 0.4902 |
| Line2 [r1,r2,c]: | 0.1307 | 0.1880 | 0.1628 | 0.1253 | 0.2000 | 0.2128 |
| Line3 [r1,r2,c]: | 0.1307 | 0.1533 | 0.0665 | 0.1200 | 0.2107 | 0.5802 |

Bailey test for 3 × 750

| | | | | | | |
|---|---|---|---|---|---|---|
| Teta [r1,r2,c]: | 0.1185 | 0.1572 | 0.0980 | 0.1308 | 0.2144 | 0.3760 |
| SE [r1,r2,c]: | 0.0068 | 0.0077 | 0.0469 | 0.0071 | 0.0086 | 0.0703 |
| Chi2: | | 15.0993 | | | 11.6659 | |
| C Chi2: | | 0.7319 | | | 5.0981 | |

Bailey for 6 × 750:

| | | | |
|---|---|---|---|
| Teta: | 0.1240 | 0.1820 | 0.1827 |
| Chi2: | 64.6400 | | |
| C chi2: | 16.6718 | | |

**ML test Control vs. selection**

chi^2(ctrl + sel) - chi^2(ctrl) - chi^2(sel): 37.8747
C chi^2(ctrl + sel) - chi^2(ctrl) - chi^2(sel): **10.8418**

**ML test Ctrl Vs. Selection**

| | | | | | | |
|---|---|---|---|---|---|---|
| ML est [r1,r2,c] | 0.1200 | 0.1591 | 0.1164 | 0.1320 | 0.2151 | 0.4383 |
| ML SE [r1,r2,c] | 0.0069 | 0.0077 | 0.0512 | 0.0071 | 0.0087 | 0.0762 |

**ML test Ctrl vs. Selection (r1 and r2 are line-specific)**

c - control or selection specific

| | | | | | | |
|---|---|---|---|---|---|---|
| Line1[r1,r2,c]: | 0.0987 | 0.1360 | 0.1150 | 0.1505 | 0.2344 | 0.4365 |
| Line2[r1,r2,c]: | 0.1305 | 0.1878 | 0.1150 | 0.1258 | 0.2007 | 0.4365 |
| Line3[r1,r2,c]: | 0.1308 | 0.1535 | 0.1150 | 0.1197 | 0.2102 | 0.4365 |

c-global

| | | | | | | |
|---|---|---|---|---|---|---|
| Line1[r1,r2,c]: | 0.0989 | 0.1363 | 0.3084 | 0.1499 | 0.2335 | 0.3084 |
| Line2[r1,r2,c]: | 0.1310 | 0.1885 | 0.3084 | 0.1256 | 0.2004 | 0.3084 |
| Line3[r1,r2,c]: | 0.1311 | 0.1539 | 0.3084 | 0.1194 | 0.2096 | 0.3084 |

ML ratio (df=14 –13=1): 10.9211

***ML test Ctrl vs. Selection (r1, r2 and c are all line-specific)**

| | | | | | | |
|---|---|---|---|---|---|---|
| Line1[r1,r2,c]: | 0.0987 | 0.1360 | 0.0994 | 0.1507 | 0.2347 | 0.4902 |
| Line2[r1,r2,c]: | 0.1307 | 0.1880 | 0.1628 | 0.1253 | 0.2000 | 0.2128 |
| Line3[r1,r2,c]: | 0.1307 | 0.1533 | 0.0665 | 0.1200 | 0.2107 | 0.5802 |

ML ratio (df=18 –13=5): 15.8472

ML ratio for heterogeneity (control+selection; df = 5–1=4): 15.8472 –10.9211

…………………………………………………………………………………………………

**markers 1-4-5 (*ru-cu-sr*)**

| | CONTROL | | | HYPOXIA | | |
|---|---|---|---|---|---|---|
| **Lines (ML estimates)** | | | | | | |
| Line1 [r1,r2,c]: | 0.2653 | 0.0533 | 0.1884 | 0.3373 | 0.0987 | 0.5608 |
| Line2 [r1,r2,c]: | 0.3213 | 0.0693 | 0.1796 | 0.2880 | 0.0827 | 0.5040 |
| Line3 [r1,r2,c]: | 0.3189 | 0.0587 | 0.0000 | 0.2707 | 0.0840 | 0.7623 |
| Bailey test for 3 × 750 | | | | | | |
| Teta [r1,r2,c]: | 0.3002 | 0.0600 | 0.0000 | 0.2975 | 0.0883 | 0.5832 |
| SE [r1,r2,c]: | 0.0096 | 0.0050 | 0.0002 | 0.0096 | 0.0060 | 0.0868 |
| Chi2: | | 15.3686 | | | 11.8461 | |
| C Chi2: | | 5.3744 | | | 1.2757 | |

Bailey for 6 × 750:

| | | | |
|---|---|---|---|
| Teta: | 0.2971 | 0.0713 | 0.0000 |
| Chi2: | 84.9401 | | |
| C chi2: | 52.6065 | | |

**ML test Control vs. selection**

chi^2(ctrl + sel) - chi^2(ctrl) - chi^2(sel): 57.7254

C chi^2(ctrl + sel) - chi^2(ctrl) - chi^2(sel): **45.9564**

**ML test Ctrl Vs. Selection**

| | | | | | | |
|---|---|---|---|---|---|---|
| ML est [r1,r2,c] | 0.3018 | 0.0604 | 0.1218 | 0.2987 | 0.0884 | 0.6057 |
| ML SE [r1,r2,c] | 0.0097 | 0.0050 | 0.0532 | 0.0096 | 0.0060 | 0.0885 |

**ML test Ctrl vs. Selection (r1 and r2 are line-specific)**

c - control or selection specific

| | | | | | | |
|---|---|---|---|---|---|---|
| Line1[r1,r2,c]: | 0.2651 | 0.0533 | 0.1208 | 0.3376 | 0.0987 | 0.6000 |
| Line2[r1,r2,c]: | 0.3209 | 0.0692 | 0.1208 | 0.2883 | 0.0828 | 0.6000 |
| Line3[r1,r2,c]: | 0.3193 | 0.0588 | 0.1208 | 0.2702 | 0.0838 | 0.6000 |

c-global

| | | | | | | |
|---|---|---|---|---|---|---|
| Line1[r1,r2,c]: | 0.2659 | 0.0534 | 0.4056 | 0.3361 | 0.0983 | 0.4056 |
| Line2[r1,r2,c]: | 0.3225 | 0.0696 | 0.4056 | 0.2875 | 0.0825 | 0.4056 |
| Line3[r1,r2,c]: | 0.3204 | 0.0590 | 0.4056 | 0.2691 | 0.0835 | 0.4056 |

ML ratio (df=14 –13=1): 18.8459

***ML test Ctrl vs. Selection (r1, r2 and c are all line-specific)**

| | | | | | | |
|---|---|---|---|---|---|---|
| Line1[r1,r2,c]: | 0.2653 | 0.0533 | 0.1884 | 0.3373 | 0.0987 | 0.5608 |
| Line2[r1,r2,c]: | 0.3213 | 0.0693 | 0.1796 | 0.2880 | 0.0827 | 0.5040 |
| Line3[r1,r2,c]: | 0.3189 | 0.0587 | 0.0000 | 0.2707 | 0.0840 | 0.7623 |

ML ratio (df=18 –13=5): 24.4607

ML ratio for heterogeneity (control+selection; df = 5–1=4): 24.4607–18.8459

……………………………………………………………………………………………………

**markers 1-4-6 (*ru-cu-e*)**

| | CONTROL | | | HYPOXIA | | |
|---|---|---|---|---|---|---|
| **Lines (ML estimates)** | | | | | | |
| Line1 [r1,r2,c]: | 0.2653 | 0.0933 | 0.2154 | 0.3373 | 0.1587 | 0.4484 |
| Line2 [r1,r2,c]: | 0.3213 | 0.1187 | 0.1748 | 0.2880 | 0.1467 | 0.4419 |
| Line3 [r1,r2,c]: | 0.3187 | 0.1053 | 0.0397 | 0.2707 | 0.1533 | 0.4819 |

Bailey test for 3 × 750

| | | | | | | |
|---|---|---|---|---|---|---|
| Teta [r1,r2,c]: | 0.3006 | 0.1053 | 0.0827 | 0.2973 | 0.1532 | 0.4529 |
| SE [r1,r2,c]: | 0.0096 | 0.0065 | 0.0330 | 0.0096 | 0.0076 | 0.0592 |
| Chi2: | | 16.1134 | | | 9.6489 | |
| C Chi2: | | 4.3638 | | | 0.0818 | |

Bailey for 6 × 750:

| | | | |
|---|---|---|---|
| Teta: | 0.2978 | 0.1249 | 0.1700 |
| Chi2: | 77.2894 | | |
| C chi2: | 34.7337 | | |

**ML test Control vs. selection**

chi^2(ctrl + sel) - chi^2(ctrl) - chi^2(sel): 51.5271
C chi^2(ctrl + sel) - chi^2(ctrl) - chi^2(sel): **30.2881**

**ML test Ctrl Vs. Selection**

| | | | | | | |
|---|---|---|---|---|---|---|
| ML est [r1,r2,c] | 0.3018 | 0.1058 | 0.1392 | 0.2987 | 0.1529 | 0.4575 |
| ML SE [r1,r2,c] | 0.0097 | 0.0065 | 0.0427 | 0.0096 | 0.0076 | 0.0594 |

**ML test Ctrl vs. Selection (r1 and r2 are line-specific)**

c - control or selection specific

| | | | | | | |
|---|---|---|---|---|---|---|
| Line1[r1,r2,c]: | 0.2649 | 0.0932 | 0.1380 | 0.3374 | 0.1587 | 0.4564 |
| Line2[r1,r2,c]: | 0.3209 | 0.1185 | 0.1380 | 0.2881 | 0.1467 | 0.4564 |
| Line3[r1,r2,c]: | 0.3196 | 0.1056 | 0.1380 | 0.2705 | 0.1532 | 0.4564 |

c-global

| | | | | | | |
|---|---|---|---|---|---|---|
| Line1[r1,r2,c]: | 0.2659 | 0.0935 | 0.3261 | 0.3355 | 0.1578 | 0.3261 |
| Line2[r1,r2,c]: | 0.3228 | 0.1192 | 0.3261 | 0.2869 | 0.1461 | 0.3261 |
| Line3[r1,r2,c]: | 0.3211 | 0.1061 | 0.3261 | 0.2693 | 0.1526 | 0.3261 |

ML ratio (df=14 –13=1): 17.0464

***ML test Ctrl vs. Selection (r1, r2 and c are all line-specific)**

| | | | | | | |
|---|---|---|---|---|---|---|
| Line1[r1,r2,c]: | 0.2653 | 0.0933 | 0.2154 | 0.3373 | 0.1587 | 0.4484 |
| Line2[r1,r2,c]: | 0.3213 | 0.1187 | 0.1748 | 0.2880 | 0.1467 | 0.4419 |
| Line3[r1,r2,c]: | 0.3187 | 0.1053 | 0.0397 | 0.2707 | 0.1533 | 0.4819 |

ML ratio (df=18 –13=5): 20.7089

ML ratio for heterogeneity (control+selection; df = 5–1=4): 20.7089 – 17.0464
……………………………………………………………………………………………………

**markers 2-4-5 (*h-cu-sr*)**

**CONTROL** **HYPOXIA**

**Lines (ML estimates)**

| | | |
|---|---|---|
| Line1 [r1,r2,c]: | 0.1440 0.0533 0.3474 | 0.2160 0.0987 0.4380 |
| Line2 [r1,r2,c]: | 0.1974 0.0693 0.0000 | 0.1787 0.0827 0.2708 |
| Line3 [r1,r2,c]: | 0.1760 0.0587 0.0000 | 0.1720 0.0840 0.6463 |

Bailey test for 3 × 750

| | | |
|---|---|---|
| Teta [r1,r2,c]: | 0.1704 0.0602 0.0000 | 0.1875 0.0881 0.4053 |
| SE [r1,r2,c]: | 0.0079 0.0050 0.0003 | 0.0082 0.0060 0.0969 |
| Chi2: | 12.6281 | 9.1789 |
| C Chi2: | 2.1506 | 2.0075 |

Bailey for 6 × 750:

Teta: 0.1782 0.0714 0.0000

Chi2: 54.9404

C chi2: 21.8969

**ML test Control vs. selection**

chi^2(ctrl + sel) - chi^2(ctrl) - chi^2(sel): 33.1334

C chi^2(ctrl + sel) - chi^2(ctrl) - chi^2(sel): **17.7387**

**ML test Ctrl Vs. Selection**

| | | |
|---|---|---|
| ML est [r1,r2,c] | 0.1724 0.0604 0.0853 | 0.1889 0.0884 0.4523 |
| ML SE [r1,r2,c] | 0.0080 0.0050 0.0597 | 0.0083 0.0060 0.1025 |

**ML test Ctrl vs. Selection (r1 and r2 are line-specific)**

c - control or selection specific

| | | | | | | |
|---|---|---|---|---|---|---|
| Line1[r1,r2,c]: | 0.1437 | 0.0532 | 0.0838 | 0.2160 | 0.0987 | 0.4484 |
| Line2[r1,r2,c]: | 0.1975 | 0.0694 | 0.0838 | 0.1790 | 0.0828 | 0.4484 |
| Line3[r1,r2,c]: | 0.1761 | 0.0587 | 0.0838 | 0.1717 | 0.0838 | 0.4484 |

c-global

| | | | | | | |
|---|---|---|---|---|---|---|
| Line1[r1,r2,c]: | 0.1440 | 0.0533 | 0.3089 | 0.2155 | 0.0985 | 0.3089 |
| Line2[r1,r2,c]: | 0.1980 | 0.0696 | 0.3089 | 0.1787 | 0.0827 | 0.3089 |
| Line3[r1,r2,c]: | 0.1764 | 0.0588 | 0.3089 | 0.1714 | 0.0837 | 0.3089 |

ML ratio (df=14 –13=1): 8.1645

***ML test Ctrl vs. Selection (r1, r2 and c are all line-specific)**

| | | | | | | |
|---|---|---|---|---|---|---|
| Line1[r1,r2,c]: | 0.1440 | 0.0533 | 0.3474 | 0.2160 | 0.0987 | 0.4380 |
| Line2[r1,r2,c]: | 0.1974 | 0.0693 | 0.0000 | 0.1787 | 0.0827 | 0.2708 |
| Line3[r1,r2,c]: | 0.1760 | 0.0587 | 0.0000 | 0.1720 | 0.0840 | 0.6463 |

ML ratio (df=18 –13=5): 15.9213

ML ratio for heterogeneity (control+selection; df = 5–1=4): 15.9213 – 8.1645

…………………………………………………………………………………………………

**markers 2-4-6 (*h-cu-e*)**

| | CONTROL | | | HYPOXIA | | |
|---|---|---|---|---|---|---|
| **Lines (ML estimates)** | | | | | | |
| Line1 [r1,r2,c]: | 0.1440 | 0.0933 | 0.1984 | 0.2160 | 0.1587 | 0.3501 |
| Line2 [r1,r2,c]: | 0.1973 | 0.1187 | 0.1139 | 0.1787 | 0.1467 | 0.2035 |
| Line3 [r1,r2,c]: | 0.1761 | 0.1054 | 0.0000 | 0.1720 | 0.1533 | 0.4550 |
| Bailey test for 3 × 750 | | | | | | |
| Teta [r1,r2,c]: | 0.1703 | 0.1052 | 0.0000 | 0.1874 | 0.1530 | 0.3076 |
| SE [r1,r2,c]: | 0.0079 | 0.0065 | 0.0002 | 0.0082 | 0.0076 | 0.0646 |
| Chi2: | | 15.9159 | | | 8.4560 | |
| C Chi2: | | 4.1846 | | | 2.3918 | |

Bailey for 6 × 750:

| | | | |
|---|---|---|---|
| Teta: | 0.1781 | 0.1248 | 0.0000 |
| Chi2: | 73.3074 | | |
| C chi2: | 29.5202 | | |

**ML test Control vs. selection**

chi^2(ctrl + sel) - chi^2(ctrl) - chi^2(sel): 48.9356

C chi^2(ctrl + sel) - chi^2(ctrl) - chi^2(sel): **22.9438**

**ML test Ctrl Vs. Selection**

| | | | | | | |
|---|---|---|---|---|---|---|
| ML est [r1,r2,c] | 0.1724 | 0.1058 | 0.0975 | 0.1889 | 0.1529 | 0.3386 |
| ML SE [r1,r2,c] | 0.0080 | 0.0065 | 0.0480 | 0.0083 | 0.0076 | 0.0678 |

**ML test Ctrl vs. Selection (r1 and r2 are line-specific)**

| | | | | | | |
|---|---|---|---|---|---|---|
| c - control or selection specific | | | | | | |
| Line1[r1,r2,c]: | 0.1438 | 0.0932 | 0.0962 | 0.2159 | 0.1586 | 0.3378 |
| Line2[r1,r2,c]: | 0.1973 | 0.1186 | 0.0962 | 0.1791 | 0.1470 | 0.3378 |
| Line3[r1,r2,c]: | 0.1763 | 0.1055 | 0.0962 | 0.1716 | 0.1530 | 0.3378 |
| c-global | | | | | | |
| Line1[r1,r2,c]: | 0.1441 | 0.0934 | 0.2447 | 0.2154 | 0.1582 | 0.2447 |
| Line2[r1,r2,c]: | 0.1978 | 0.1190 | 0.2447 | 0.1788 | 0.1468 | 0.2447 |
| Line3[r1,r2,c]: | 0.1766 | 0.1057 | 0.2447 | 0.1712 | 0.1526 | 0.2447 |

ML ratio (df=14 –13=1): 7.4570

***ML test Ctrl vs. Selection (r1, r2 and c are all line-specific)**

| | | | | | | |
|---|---|---|---|---|---|---|
| Line1[r1,r2,c]: | 0.1440 | 0.0933 | 0.1984 | 0.2160 | 0.1587 | 0.3501 |
| Line2[r1,r2,c]: | 0.1973 | 0.1187 | 0.1139 | 0.1787 | 0.1467 | 0.2035 |
| Line3[r1,r2,c]: | 0.1761 | 0.1054 | 0.0000 | 0.1720 | 0.1533 | 0.4550 |

ML ratio (df=18 –13=5): 13.2867

ML ratio for heterogeneity (control+selection; df = 5–1=4): 13.2867 – 7.4570
……………………………………………………………………………………………………

**markers 1-5-6 (*ru-sr-e*)**

| | CONTROL | HYPOXIA |
|---|---|---|
| **Lines (ML estimates)** | | |
| Line1 [r1,r2,c]: | 0.3133 0.0400 0.2127 | 0.3987 0.0680 0.3443 |
| Line2 [r1,r2,c]: | 0.3827 0.0493 0.1413 | 0.3467 0.0693 0.3883 |
| Line3 [r1,r2,c]: | 0.3773 0.0467 0.0757 | 0.3200 0.0747 0.2232 |
| Bailey test for 3 × 750 | | |
| Teta [r1,r2,c]: | 0.3566 0.0453 0.1145 | 0.3538 0.0706 0.3045 |
| SE [r1,r2,c]: | 0.0101 0.0044 0.0546 | 0.0101 0.0054 0.0683 |
| Chi2: | 12.7391 | 11.3900 |
| C Chi2: | 0.8102 | 1.0879 |

Bailey for 6 × 750:
Teta: 0.3554 0.0553 0.1879
Chi2: 42.0020
C chi2: 6.6138

**ML test Control vs. selection**
chi^2(ctrl + sel) - chi^2(ctrl) - chi^2(sel): 17.8729
C chi^2(ctrl + sel) - chi^2(ctrl) - chi^2(sel): **4.7158**

**ML test Ctrl Vs. Selection**

| | CONTROL | HYPOXIA |
|---|---|---|
| ML est [r1,r2,c] | 0.3578 0.0453 0.1370 | 0.3551 0.0707 0.3188 |
| ML SE [r1,r2,c] | 0.0101 0.0044 0.0595 | 0.0101 0.0054 0.0697 |

**ML test Ctrl vs. Selection (r1 and r2 are line-specific)**

c - control or selection specific

| | | | | | | |
|---|---|---|---|---|---|---|
| Line1[r1,r2,c]: | 0.3131 | 0.0400 | 0.1358 | 0.3985 | 0.0680 | 0.3203 |
| Line2[r1,r2,c]: | 0.3826 | 0.0493 | 0.1358 | 0.3462 | 0.0692 | 0.3203 |
| Line3[r1,r2,c]: | 0.3777 | 0.0467 | 0.1358 | 0.3206 | 0.0748 | 0.3203 |

c-global

| | | | | | | |
|---|---|---|---|---|---|---|
| Line1[r1,r2,c]: | 0.3134 | 0.0400 | 0.2475 | 0.3978 | 0.0678 | 0.2475 |
| Line2[r1,r2,c]: | 0.3833 | 0.0494 | 0.2475 | 0.3457 | 0.0691 | 0.2475 |
| Line3[r1,r2,c]: | 0.3783 | 0.0468 | 0.2475 | 0.3202 | 0.0747 | 0.2475 |

ML ratio (df=14 –13=1): 3.6819

***ML test Ctrl vs. Selection (r1, r2 and c are all line-specific)**

| | | | | | | |
|---|---|---|---|---|---|---|
| Line1[r1,r2,c]: | 0.3133 | 0.0400 | 0.2127 | 0.3987 | 0.0680 | 0.3443 |

| | | | | | | |
|---|---|---|---|---|---|---|
| Line2[r1,r2,c]: | 0.3827 | 0.0493 | 0.1413 | 0.3467 | 0.0693 | 0.3883 |
| Line3[r1,r2,c]: | 0.3773 | 0.0467 | 0.0757 | 0.3200 | 0.0747 | 0.2232 |

ML ratio (df=18 –13=5): 5.491

ML ratio for heterogeneity (control+selection; df = 5–1=4): 5.491 – 3.6819

……………………………………………………………………………………………………

**markers 2-5-6 (*h-sr-e*)**

| | CONTROL | | | HYPOXIA | | |
|---|---|---|---|---|---|---|
| **Lines (ML estimates)** | | | | | | |
| Line1 [r1,r2,c]: | 0.1920 | 0.0400 | 0.0000 | 0.2960 | 0.0680 | 0.3312 |
| Line2 [r1,r2,c]: | 0.2667 | 0.0493 | 0.2028 | 0.2533 | 0.0693 | 0.2277 |
| Line3 [r1,r2,c]: | 0.2347 | 0.0467 | 0.0000 | 0.2373 | 0.0747 | 0.3010 |
| Bailey test for 3 × 750 | | | | | | |
| Teta [r1,r2,c]: | 0.2281 | 0.0451 | 0.0000 | 0.2608 | 0.0707 | 0.2816 |
| SE [r1,r2,c]: | 0.0088 | 0.0044 | 0.0003 | 0.0092 | 0.0054 | 0.0782 |
| Chi2: | | 15.7674 | | | 7.3447 | |
| C Chi2: | | 2.1435 | | | 0.3285 | |

Bailey for 6 × 750:

| | | | |
|---|---|---|---|
| Teta: | 0.2435 | 0.0550 | 0.0000 |
| Chi2: | 57.8110 | | |
| C chi2: | 15.5330 | | |

**ML test Control vs. selection**

chi^2(ctrl + sel) - chi^2(ctrl) - chi^2(sel): 34.6990

C chi^2(ctrl + sel) - chi^2(ctrl) - chi^2(sel): **13.0609**

**ML test Ctrl Vs. Selection**

| | | | | | | |
|---|---|---|---|---|---|---|
| ML est [r1,r2,c] | 0.2311 | 0.0453 | 0.0848 | 0.2622 | 0.0707 | 0.2878 |
| ML SE [r1,r2,c] | 0.0089 | 0.0044 | 0.0593 | 0.0093 | 0.0054 | 0.0789 |

**ML test Ctrl vs. Selection (r1 and r2 are line-specific)**

| | | | | | | |
|---|---|---|---|---|---|---|
| c - control or selection specific | | | | | | |
| Line1[r1,r2,c]: | 0.1921 | 0.0400 | 0.0842 | 0.2958 | 0.0679 | 0.2889 |
| Line2[r1,r2,c]: | 0.2663 | 0.0493 | 0.0842 | 0.2535 | 0.0694 | 0.2889 |
| Line3[r1,r2,c]: | 0.2349 | 0.0467 | 0.0842 | 0.2373 | 0.0746 | 0.2889 |
| c-global | | | | | | |
| Line1[r1,r2,c]: | 0.1923 | 0.0400 | 0.2146 | 0.2954 | 0.0679 | 0.2146 |
| Line2[r1,r2,c]: | 0.2667 | 0.0493 | 0.2146 | 0.2533 | 0.0693 | 0.2146 |
| Line3[r1,r2,c]: | 0.2351 | 0.0468 | 0.2146 | 0.2370 | 0.0746 | 0.2146 |

ML ratio (df=14 –13=1): 3.6306

***ML test Ctrl vs. Selection (r1, r2 and c are all line-specific)**

| | | | | | | |
|---|---|---|---|---|---|---|
| Line1[r1,r2,c]: | 0.1920 | 0.0400 | 0.0000 | 0.2960 | 0.0680 | 0.3312 |
| Line2[r1,r2,c]: | 0.2667 | 0.0493 | 0.2028 | 0.2533 | 0.0693 | 0.2277 |
| Line3[r1,r2,c]: | 0.2347 | 0.0467 | 0.0000 | 0.2373 | 0.0747 | 0.3010 |

ML ratio (df=18 –13=5): 7.5435

ML ratio for heterogeneity (control+selection; df = 5–1=4): 7.5435 – 3.6306
…………………………………………………………………………………………………………

## markers 1–2–3 (*ru-h-th*)

| | CONTROL | | | HYPEROXIA | | |
|---|---|---|---|---|---|---|
| **Lines (ML estimates)** | | | | | | |
| Line1 [r1,r2,c]: | 0.1320 | 0.0987 | 0.4095 | 0.1507 | 0.1173 | 0.5279 |
| Line2 [r1,r2,c]: | 0.1587 | 0.1307 | 0.7074 | 0.1413 | 0.1093 | 0.4315 |
| Line3 [r1,r2,c]: | 0.1587 | 0.1307 | 0.3216 | 0.1400 | 0.1240 | 0.2304 |
| Bailey test for 3 × 750 | | | | | | |
| Teta [r1,r2,c]: | 0.1489 | 0.1182 | 0.4399 | 0.1439 | 0.1166 | 0.3535 |
| SE [r1,r2,c]: | 0.0075 | 0.0068 | 0.0966 | 0.0074 | 0.0068 | 0.0914 |
| Chi2: | | 11.6099 | | | 3.1743 | |
| C Chi2: | | 2.7373 | | | 1.9880 | |

Bailey for 6 × 750:

| | | | |
|---|---|---|---|
| Teta: | 0.1464 | 0.1174 | 0.3943 |
| Chi2: | 15.4616 | | |
| C chi2: | 5.1646 | | |

**ML test Control vs. selection**

chi^2(ctrl + sel) - chi^2(ctrl) - chi^2(sel): 0.6774

C chi^2(ctrl + sel) - chi^2(ctrl) - chi^2(sel): **0.4394**

**ML test Ctrl Vs. Selection**

| | | | | | | |
|---|---|---|---|---|---|---|
| ML est [r1,r2,c] | 0.1498 | 0.1200 | 0.4946 | 0.1440 | 0.1169 | 0.3960 |
| ML SE [r1,r2,c] | 0.0075 | 0.0069 | 0.1029 | 0.0074 | 0.0068 | 0.0968 |

**ML test Ctrl vs. Selection (r1 and r2 are line-specific)**

c - control or selection specific

| | | | | | | |
|---|---|---|---|---|---|---|
| Line1[r1,r2,c]: | 0.1321 | 0.0987 | 0.4900 | 0.1504 | 0.1171 | 0.3961 |
| Line2[r1,r2,c]: | 0.1582 | 0.1303 | 0.4900 | 0.1413 | 0.1093 | 0.3961 |
| Line3[r1,r2,c]: | 0.1590 | 0.1309 | 0.4900 | 0.1403 | 0.1242 | 0.3961 |

c-global

| | | | | | | |
|---|---|---|---|---|---|---|
| Line1[r1,r2,c]: | 0.1320 | 0.0987 | 0.4449 | 0.1505 | 0.1172 | 0.4449 |
| Line2[r1,r2,c]: | 0.1581 | 0.1302 | 0.4449 | 0.1413 | 0.1093 | 0.4449 |
| Line3[r1,r2,c]: | 0.1589 | 0.1309 | 0.4449 | 0.1403 | 0.1243 | 0.4449 |

ML ratio (df=14 –13=1): 0.4439

***ML test Ctrl vs. Selection (r1, r2 and c are all line-specific)**

| | | | | | | |
|---|---|---|---|---|---|---|
| Line1[r1,r2,c]: | 0.1320 | 0.0987 | 0.4095 | 0.1507 | 0.1173 | 0.5279 |
| Line2[r1,r2,c]: | 0.1587 | 0.1307 | 0.7074 | 0.1413 | 0.1093 | 0.4315 |
| Line3[r1,r2,c]: | 0.1587 | 0.1307 | 0.3216 | 0.1400 | 0.1240 | 0.2304 |

ML ratio (df=18 –13=5): 5.1458

ML ratio for heterogeneity (control+selection; df = 5–1=4): 5.1458 – 0.4439
…………………………………………………………………………………………………………

## markers 1-2-4 (*ru-h-cu*)

| | CONTROL | | | HYPEROXIA | | |
|---|---|---|---|---|---|---|
| **Lines (ML estimates)** | | | | | | |
| Line1 [r1,r2,c]: | 0.1320 | 0.1440 | 0.2806 | 0.1507 | 0.1667 | 0.4248 |
| Line2 [r1,r2,c]: | 0.1587 | 0.1973 | 0.5536 | 0.1413 | 0.1547 | 0.3660 |
| Line3 [r1,r2,c]: | 0.1587 | 0.1760 | 0.2865 | 0.1400 | 0.1827 | 0.2086 |
| Bailey test for 3 × 750 | | | | | | |
| Teta [r1,r2,c]: | 0.1488 | 0.1701 | 0.3572 | 0.1441 | 0.1673 | 0.3028 |
| SE [r1,r2,c]: | 0.0075 | 0.0079 | 0.0727 | 0.0074 | 0.0079 | 0.0706 |
| Chi2: | | 14.3972 | | | 4.3897 | |
| C Chi2: | | 2.7682 | | | 1.8466 | |

Bailey for 6 × 750:

| | | | |
|---|---|---|---|
| Teta: | 0.1464 | 0.1687 | 0.3291 |
| Chi2: | 19.3495 | | |
| C chi2: | 4.9187 | | |

**ML test Control vs. selection**

chi^2(ctrl + sel) - chi^2(ctrl) - chi^2(sel): 0.5625
C chi^2(ctrl + sel) - chi^2(ctrl) - chi^2(sel**): 0.3040**

**ML test Ctrl Vs. Selection**

| | | | | | | |
|---|---|---|---|---|---|---|
| ML est [r1,r2,c] | 0.1498 | 0.1724 | 0.3958 | 0.1440 | 0.1680 | 0.3307 |
| ML SE [r1,r2,c] | 0.0075 | 0.0080 | 0.0770 | 0.0074 | 0.0079 | 0.0737 |

**ML test Ctrl vs. Selection (r1 and r2 are line-specific)**

c - control or selection specific

| | | | | | | |
|---|---|---|---|---|---|---|
| Line1[r1,r2,c]: | 0.1322 | 0.1442 | 0.3933 | 0.1504 | 0.1664 | 0.3306 |
| Line2[r1,r2,c]: | 0.1581 | 0.1966 | 0.3933 | 0.1412 | 0.1546 | 0.3306 |
| Line3[r1,r2,c]: | 0.1590 | 0.1764 | 0.3933 | 0.1403 | 0.1831 | 0.3306 |

c-global

| | | | | | | |
|---|---|---|---|---|---|---|
| Line1[r1,r2,c]: | 0.1321 | 0.1442 | 0.3632 | 0.1505 | 0.1665 | 0.3632 |
| Line2[r1,r2,c]: | 0.1580 | 0.1965 | 0.3632 | 0.1413 | 0.1547 | 0.3632 |
| Line3[r1,r2,c]: | 0.1589 | 0.1763 | 0.3632 | 0.1404 | 0.1832 | 0.3632 |

ML ratio (df=14 –13=1): 0.3467

***ML test Ctrl vs. Selection (r1, r2 and c are all line-specific)**

| | | | | | | |
|---|---|---|---|---|---|---|
| Line1[r1,r2,c]: | 0.1320 | 0.1440 | 0.2806 | 0.1507 | 0.1667 | 0.4248 |
| Line2[r1,r2,c]: | 0.1587 | 0.1973 | 0.5536 | 0.1413 | 0.1547 | 0.3660 |
| Line3[r1,r2,c]: | 0.1587 | 0.1760 | 0.2865 | 0.1400 | 0.1827 | 0.2086 |

ML ratio (df=18 –13=5): 4.9914

ML ratio for heterogeneity (control+selection; df = 5–1=4): 4.9914 – 0.3467

…………………………………………………………………………………………………………

## markers 1-2-5 (*ru-h-sr*)

| | CONTROL | | | HYPEROXIA | | |
|---|---|---|---|---|---|---|
| **Lines (ML estimates)** | | | | | | |
| Line1 [r1,r2,c]: | 0.1320 | 0.1920 | 0.2104 | 0.1507 | 0.2280 | 0.3105 |
| Line2 [r1,r2,c]: | 0.1587 | 0.2667 | 0.5042 | 0.1413 | 0.2227 | 0.3389 |
| Line3 [r1,r2,c]: | 0.1587 | 0.2347 | 0.2149 | 0.1400 | 0.2493 | 0.1910 |
| Bailey test for 3 × 750 | | | | | | |
| Teta [r1,r2,c]: | 0.1487 | 0.2280 | 0.2833 | 0.1440 | 0.2329 | 0.2620 |
| SE [r1,r2,c]: | 0.0075 | 0.0088 | 0.0559 | 0.0074 | 0.0089 | 0.0556 |
| Chi2: | | 21.5049 | | | 3.5282 | |
| C Chi2: | | 5.1127 | | | 1.4487 | |

Bailey for 6 × 750:

| | | | |
|---|---|---|---|
| Teta: | 0.1464 | 0.2304 | 0.2726 |
| Chi2: | 25.4060 | | |
| C chi2: | 6.6500 | | |

**ML test Control vs. selection**

chi^2(ctrl + sel) - chi^2(ctrl) - chi^2(sel): 0.3729

C chi^2(ctrl + sel) - chi^2(ctrl) - chi^2(sel): **0.0886**

**ML test Ctrl Vs. Selection**

| | | | | | | |
|---|---|---|---|---|---|---|
| ML est [r1,r2,c] | 0.1498 | 0.2311 | 0.3338 | 0.1440 | 0.2333 | 0.2778 |
| ML SE [r1,r2,c] | 0.0075 | 0.0089 | 0.0610 | 0.0074 | 0.0089 | 0.0572 |

**ML test Ctrl vs. Selection (r1 and r2 are line-specific)**

c - control or selection specific

| | | | | | | |
|---|---|---|---|---|---|---|
| Line1[r1,r2,c]: | 0.1323 | 0.1924 | 0.3322 | 0.1505 | 0.2278 | 0.2778 |
| Line2[r1,r2,c]: | 0.1578 | 0.2652 | 0.3322 | 0.1411 | 0.2223 | 0.2778 |
| Line3[r1,r2,c]: | 0.1592 | 0.2355 | 0.3322 | 0.1403 | 0.2499 | 0.2778 |

c-global

| | | | | | | |
|---|---|---|---|---|---|---|
| Line1[r1,r2,c]: | 0.1322 | 0.1924 | 0.3055 | 0.1506 | 0.2280 | 0.3055 |
| Line2[r1,r2,c]: | 0.1576 | 0.2649 | 0.3055 | 0.1412 | 0.2225 | 0.3055 |
| Line3[r1,r2,c]: | 0.1591 | 0.2353 | 0.3055 | 0.1404 | 0.2501 | 0.3055 |

ML ratio (df=14 –13=1): 0.4244

***ML test Ctrl vs. Selection (r1, r2 and c are all line-specific)**

| | | | | | | |
|---|---|---|---|---|---|---|
| Line1[r1,r2,c]: | 0.1320 | 0.1920 | 0.2104 | 0.1507 | 0.2280 | 0.3105 |
| Line2[r1,r2,c]: | 0.1587 | 0.2667 | 0.5042 | 0.1413 | 0.2227 | 0.3389 |
| Line3[r1,r2,c]: | 0.1587 | 0.2347 | 0.2149 | 0.1400 | 0.2493 | 0.1910 |

ML ratio (df=18 –13=5): 7.2635

ML ratio for heterogeneity (control+selection; df = 5–1=4): 7.2635 – 0.4244
……………………………………………………………………………………………………

## markers 1-2-6 (*ru-h-e*)

| | CONTROL | | | HYPEROXIA | | |
|---|---|---|---|---|---|---|
| **Lines (ML estimates)** | | | | | | |
| Line1 [r1,r2,c]: | 0.1320 | 0.2320 | 0.2612 | 0.1507 | 0.2760 | 0.3527 |
| Line2 [r1,r2,c]: | 0.1587 | 0.3107 | 0.4328 | 0.1413 | 0.2680 | 0.2816 |
| Line3 [r1,r2,c]: | 0.1587 | 0.2813 | 0.2091 | 0.1400 | 0.2933 | 0.1623 |
| Bailey test for 3 × 750 | | | | | | |
| Teta [r1,r2,c]: | 0.1490 | 0.2723 | 0.2818 | 0.1440 | 0.2787 | 0.2411 |
| SE [r1,r2,c]: | 0.0075 | 0.0094 | 0.0511 | 0.0074 | 0.0094 | 0.0485 |
| Chi2: | | 20.5299 | | | 4.4724 | |
| C Chi2: | | 3.4525 | | | 2.7750 | |

Bailey for 6 × 750:

| | | | |
|---|---|---|---|
| Teta: | 0.1465 | 0.2754 | 0.2604 |
| Chi2: | 25.7341 | | |
| C chi2: | 6.6141 | | |

**ML test Control vs. selection**

chi^2(ctrl + sel) - chi^2(ctrl) - chi^2(sel): 0.7317

C chi^2(ctrl + sel) - chi^2(ctrl) - chi^2(sel): **0.3866**

**ML test Ctrl Vs. Selection**

| | | | | | | |
|---|---|---|---|---|---|---|
| ML est [r1,r2,c] | 0.1498 | 0.2747 | 0.3133 | 0.1440 | 0.2791 | 0.2654 |
| ML SE [r1,r2,c] | 0.0075 | 0.0094 | 0.0540 | 0.0074 | 0.0095 | 0.0509 |

**ML test Ctrl vs. Selection (r1 and r2 are line-specific)**

c - control or selection specific

| | | | | | | |
|---|---|---|---|---|---|---|
| Line1[r1,r2,c]: | 0.1322 | 0.2323 | 0.3113 | 0.1502 | 0.2752 | 0.2653 |
| Line2[r1,r2,c]: | 0.1579 | 0.3092 | 0.3113 | 0.1413 | 0.2679 | 0.2653 |
| Line3[r1,r2,c]: | 0.1592 | 0.2824 | 0.3113 | 0.1405 | 0.2944 | 0.2653 |

c-global

| | | | | | | |
|---|---|---|---|---|---|---|
| Line1[r1,r2,c]: | 0.1321 | 0.2322 | 0.2887 | 0.1503 | 0.2754 | 0.2887 |
| Line2[r1,r2,c]: | 0.1577 | 0.3088 | 0.2887 | 0.1414 | 0.2681 | 0.2887 |
| Line3[r1,r2,c]: | 0.1591 | 0.2821 | 0.2887 | 0.1406 | 0.2946 | 0.2887 |

ML ratio (df=14 –13=1): 0.3851

**ML test Ctrl vs. Selection (r1, r2 and c are all line-specific)**

| | | | | | | |
|---|---|---|---|---|---|---|
| Line1[r1,r2,c]: | 0.1320 | 0.2320 | 0.2612 | 0.1507 | 0.2760 | 0.3527 |
| Line2[r1,r2,c]: | 0.1587 | 0.3107 | 0.4328 | 0.1413 | 0.2680 | 0.2816 |
| Line3[r1,r2,c]: | 0.1587 | 0.2813 | 0.2091 | 0.1400 | 0.2933 | 0.1623 |

ML ratio (df=18 –13=5): 6.5033

ML ratio for heterogeneity (control+selection; df = 5–1=4): 6.5033 – 0.3851

……………………………………………………………………………………………………

**markers 1-3-4 (*ru-th-cu*)**

| | CONTROL | | | HYPEROXIA | | |
|---|---|---|---|---|---|---|
| **Lines (ML estimates)** | | | | | | |
| Line1 [r1,r2,c]: | 0.2200 | 0.0453 | 0.0000 | 0.2493 | 0.0547 | 0.2934 |
| Line2 [r1,r2,c]: | 0.2600 | 0.0693 | 0.2219 | 0.2373 | 0.0480 | 0.2341 |
| Line3 [r1,r2,c]: | 0.2760 | 0.0480 | 0.2013 | 0.2560 | 0.0640 | 0.2441 |
| Bailey test for 3 × 750 | | | | | | |
| Teta [r1,r2,c]: | 0.2503 | 0.0523 | 0.0000 | 0.2475 | 0.0549 | 0.2547 |
| SE [r1,r2,c]: | 0.0091 | 0.0047 | 0.0005 | 0.0091 | 0.0048 | 0.0870 |
| Chi2: | | 17.1457 | | | 2.8928 | |
| C Chi2: | | 5.3936 | | | 0.0803 | |

Bailey for 6 × 750:

| | | | |
|---|---|---|---|
| Teta: | 0.2485 | 0.0535 | 0.0000 |
| Chi2: | 28.7889 | | |
| C chi2: | 14.1087 | | |

**ML test Control vs. selection**
chi^2(ctrl + sel) - chi^2(ctrl) - chi^2(sel): 8.7505
C chi^2(ctrl + sel) - chi^2(ctrl) - chi^2(sel): **8.6348**

**ML test Ctrl Vs. Selection**

| | | | | | | |
|---|---|---|---|---|---|---|
| ML est [r1,r2,c] | 0.2520 | 0.0542 | 0.1626 | 0.2476 | 0.0556 | 0.2586 |
| ML SE [r1,r2,c] | 0.0092 | 0.0048 | 0.0708 | 0.0091 | 0.0048 | 0.0877 |

**ML test Ctrl vs. Selection (r1 and r2 are line-specific)**

c - control or selection specific

| | | | | | | |
|---|---|---|---|---|---|---|
| Line1[r1,r2,c]: | 0.2203 | 0.0454 | 0.1621 | 0.2492 | 0.0546 | 0.2576 |
| Line2[r1,r2,c]: | 0.2597 | 0.0693 | 0.1621 | 0.2374 | 0.0480 | 0.2576 |
| Line3[r1,r2,c]: | 0.2759 | 0.0480 | 0.1621 | 0.2560 | 0.0640 | 0.2576 |

c-global

| | | | | | | |
|---|---|---|---|---|---|---|
| Line1[r1,r2,c]: | 0.2204 | 0.0454 | 0.2101 | 0.2491 | 0.0546 | 0.2101 |
| Line2[r1,r2,c]: | 0.2600 | 0.0693 | 0.2101 | 0.2373 | 0.0480 | 0.2101 |
| Line3[r1,r2,c]: | 0.2760 | 0.0480 | 0.2101 | 0.2559 | 0.0640 | 0.2101 |

ML ratio (df=14 –13=1): 0.7272

***ML test Ctrl vs. Selection (r1, r2 and c are all line-specific)**

| | | | | | | |
|---|---|---|---|---|---|---|
| Line1[r1,r2,c]: | 0.2200 | 0.0453 | 0.0000 | 0.2493 | 0.0547 | 0.2934 |
| Line2[r1,r2,c]: | 0.2600 | 0.0693 | 0.2219 | 0.2373 | 0.0480 | 0.2341 |
| Line3[r1,r2,c]: | 0.2760 | 0.0480 | 0.2013 | 0.2560 | 0.0640 | 0.2441 |

ML ratio (df=18 –13=5): 3.6682

ML ratio for heterogeneity (control+selection; df = 5–1=4): 3.6682 – 0.7272
……………………………………………………………………………………………………

**markers 1-3-5 (*ru-th-sr*)**

**CONTROL** **HYPEROXIA**

**Lines (ML estimates)**

| | | | | | | |
|---|---|---|---|---|---|---|
| Line1 [r1,r2,c]: | 0.2200 | 0.0960 | 0.0631 | 0.2493 | 0.1187 | 0.1803 |
| Line2 [r1,r2,c]: | 0.2600 | 0.1387 | 0.2219 | 0.2373 | 0.1160 | 0.1937 |
| Line3 [r1,r2,c]: | 0.2760 | 0.1067 | 0.0906 | 0.2560 | 0.1360 | 0.2298 |

Bailey test for 3 × 750

| | | | | | | |
|---|---|---|---|---|---|---|
| Teta [r1,r2,c]: | 0.2507 | 0.1116 | 0.1056 | 0.2475 | 0.1231 | 0.2006 |
| SE [r1,r2,c]: | 0.0091 | 0.0066 | 0.0394 | 0.0091 | 0.0069 | 0.0518 |
| Chi2: | | 17.3747 | | | 2.8120 | |
| C Chi2: | | 2.3425 | | | 0.1675 | |

Bailey for 6 × 750:
Teta: 0.2490 0.1170 0.1404

| | |
|---|---|
| Chi2: | 23.6818 |
| C chi2: | 4.5987 |

**<u>ML test Control vs. selection</u>**

chi^2(ctrl + sel) - chi^2(ctrl) - chi^2(sel): 3.4951

C chi^2(ctrl + sel) - chi^2(ctrl) - chi^2(sel): **<u>2.0886</u>**

**<u>ML test Ctrl Vs. Selection</u>**

| | | | | | | |
|---|---|---|---|---|---|---|
| ML est [r1,r2,c] | 0.2520 | 0.1138 | 0.1395 | 0.2476 | 0.1236 | 0.2034 |
| ML SE [r1,r2,c] | 0.0092 | 0.0067 | 0.0452 | 0.0091 | 0.0069 | 0.0522 |

**<u>ML test Ctrl vs. Selection (r1 and r2 are line-specific)</u>**

c - control or selection specific

| | | | | | | |
|---|---|---|---|---|---|---|
| Line1[r1,r2,c]: | 0.2203 | 0.0961 | 0.1388 | 0.2495 | 0.1187 | 0.2031 |
| Line2[r1,r2,c]: | 0.2593 | 0.1383 | 0.1388 | 0.2374 | 0.1160 | 0.2031 |
| Line3[r1,r2,c]: | 0.2764 | 0.1068 | 0.1388 | 0.2558 | 0.1359 | 0.2031 |

c-global

| | | | | | | |
|---|---|---|---|---|---|---|
| Line1[r1,r2,c]: | 0.2204 | 0.0962 | 0.1720 | 0.2493 | 0.1186 | 0.1720 |
| Line2[r1,r2,c]: | 0.2596 | 0.1384 | 0.1720 | 0.2372 | 0.1159 | 0.1720 |
| Line3[r1,r2,c]: | 0.2766 | 0.1069 | 0.1720 | 0.2555 | 0.1358 | 0.1720 |

ML ratio (df=14 –13=1): 0.8685

**<u>*ML test Ctrl vs. Selection (r1, r2 and c are all line-specific)</u>**

| | | | | | | |
|---|---|---|---|---|---|---|
| Line1[r1,r2,c]: | 0.2200 | 0.0960 | 0.0631 | 0.2493 | 0.1187 | 0.1803 |
| Line2[r1,r2,c]: | 0.2600 | 0.1387 | 0.2219 | 0.2373 | 0.1160 | 0.1937 |
| Line3[r1,r2,c]: | 0.2760 | 0.1067 | 0.0906 | 0.2560 | 0.1360 | 0.2298 |

ML ratio (df=18 –13=5): 3.5566

ML ratio for heterogeneity (control+selection; df = 5–1=4): 3.5566 – 0.8685

…………………………………………………………………………………………………………

## <u>markers 1-3-6 (*ru-th-e*)</u>

| | CONTROL | | | HYPEROXIA | | |
|---|---|---|---|---|---|---|
| **<u>Lines (ML estimates)</u>** | | | | | | |
| Line1 [r1,r2,c]: | 0.2200 | 0.1360 | 0.1337 | 0.2493 | 0.1640 | 0.1956 |
| Line2 [r1,r2,c]: | 0.2600 | 0.1880 | 0.2182 | 0.2373 | 0.1613 | 0.1393 |
| Line3 [r1,r2,c]: | 0.2760 | 0.1533 | 0.0945 | 0.2560 | 0.1853 | 0.2248 |
| Bailey test for 3 × 750 | | | | | | |
| Teta [r1,r2,c]: | 0.2510 | 0.1573 | 0.1344 | 0.2474 | 0.1697 | 0.1825 |
| SE [r1,r2,c]: | 0.0091 | 0.0077 | 0.0374 | 0.0091 | 0.0079 | 0.0420 |
| Chi2: | | 18.4370 | | | 3.6556 | |
| C Chi2: | | 1.8848 | | | 0.7584 | |

Bailey for 6 × 750:

| | |
|---|---|
| Teta: | 0.2492 0.1632 0.1556 |
| Chi2: | 24.0393 |
| C chi2: | 3.3248 |

**ML test Control vs. selection**

chi^2(ctrl + sel) - chi^2(ctrl) - chi^2(sel): 1.9468
C chi^2(ctrl + sel) - chi^2(ctrl) - chi^2(sel): **0.6815**

**ML test Ctrl Vs. Selection**

| | | | | | | |
|---|---|---|---|---|---|---|
| ML est [r1,r2,c] | 0.2520 | 0.1591 | 0.1552 | 0.2476 | 0.1702 | 0.1898 |
| ML SE [r1,r2,c] | 0.0092 | 0.0077 | 0.0400 | 0.0091 | 0.0079 | 0.0428 |

**ML test Ctrl vs. Selection (r1 and r2 are line-specific)**

c - control or selection specific

| | | | | | | |
|---|---|---|---|---|---|---|
| Line1[r1,r2,c]: | 0.2201 | 0.1361 | 0.1542 | 0.2493 | 0.1640 | 0.1897 |
| Line2[r1,r2,c]: | 0.2592 | 0.1875 | 0.1542 | 0.2377 | 0.1616 | 0.1897 |
| Line3[r1,r2,c]: | 0.2766 | 0.1537 | 0.1542 | 0.2556 | 0.1851 | 0.1897 |

c-global

| | | | | | | |
|---|---|---|---|---|---|---|
| Line1[r1,r2,c]: | 0.2202 | 0.1361 | 0.1724 | 0.2491 | 0.1639 | 0.1724 |
| Line2[r1,r2,c]: | 0.2595 | 0.1876 | 0.1724 | 0.2376 | 0.1615 | 0.1724 |
| Line3[r1,r2,c]: | 0.2768 | 0.1538 | 0.1724 | 0.2554 | 0.1849 | 0.1724 |

ML ratio: 0.3664

***ML test Ctrl vs. Selection (r1, r2 and c are all line-specific)**

| | | | | | | |
|---|---|---|---|---|---|---|
| Line1[r1,r2,c]: | 0.2200 | 0.1360 | 0.1337 | 0.2493 | 0.1640 | 0.1956 |
| Line2[r1,r2,c]: | 0.2600 | 0.1880 | 0.2182 | 0.2373 | 0.1613 | 0.1393 |
| Line3[r1,r2,c]: | 0.2760 | 0.1533 | 0.0945 | 0.2560 | 0.1853 | 0.2248 |

ML ratio (df=18 –13=5): 2.9871

ML ratio for heterogeneity (control+selection; df = 5–1=4): 2.9871 –0.3664

……………………………………………………………………………………………………

## markers 2-3-4 (*h-th-cu*)

**Lines (ML estimates)**

| | CONTROL | | | HYPEROXIA | | |
|---|---|---|---|---|---|---|
| Line1 [r1,r2,c]: | 0.0987 | 0.0453 | 0.0000 | 0.1173 | 0.0547 | 0.4164 |
| Line2 [r1,r2,c]: | 0.1307 | 0.0693 | 0.1472 | 0.1093 | 0.0480 | 0.2545 |
| Line3 [r1,r2,c]: | 0.1307 | 0.0480 | 0.2127 | 0.1240 | 0.0640 | 0.3367 |

Bailey test for 3 × 750

| | | | | | | |
|---|---|---|---|---|---|---|
| Teta [r1,r2,c]: | 0.1183 | 0.0524 | 0.0000 | 0.1167 | 0.0548 | 0.3290 |
| SE [r1,r2,c]: | 0.0068 | 0.0047 | 0.0003 | 0.0068 | 0.0048 | 0.1452 |
| Chi2: | | 12.3439 | | | 2.9477 | |
| C Chi2: | | 2.0724 | | | 0.1856 | |

Bailey for 6 × 750:

| | | | |
|---|---|---|---|
| Teta: | 0.1174 | 0.0536 | 0.0000 |
| Chi2: | 20.5727 | | |
| C chi2: | 7.4172 | | |

**ML test Control vs. selection**

chi^2(ctrl + sel) - chi^2(ctrl) - chi^2(sel): 5.2811

C chi^2(ctrl + sel) - chi^2(ctrl) - chi^2(sel): **5.1591**

**ML test Ctrl Vs. Selection**

| | | | | | | |
|---|---|---|---|---|---|---|
| ML est [r1,r2,c] | 0.1200 | 0.0542 | 0.1367 | 0.1169 | 0.0556 | 0.3423 |
| ML SE [r1,r2,c] | 0.0069 | 0.0048 | 0.0954 | 0.0068 | 0.0048 | 0.1484 |

**ML test Ctrl vs. Selection (r1 and r2 are line-specific)**

c - control or selection specific

| | | | | | | |
|---|---|---|---|---|---|---|
| Line1[r1,r2,c]: | 0.0987 | 0.0454 | 0.1349 | 0.1173 | 0.0546 | 0.3402 |
| Line2[r1,r2,c]: | 0.1306 | 0.0693 | 0.1349 | 0.1094 | 0.0480 | 0.3402 |
| Line3[r1,r2,c]: | 0.1306 | 0.0480 | 0.1349 | 0.1240 | 0.0640 | 0.3402 |

c-global

| | | | | | | |
|---|---|---|---|---|---|---|
| Line1[r1,r2,c]: | 0.0987 | 0.0454 | 0.2369 | 0.1172 | 0.0546 | 0.2369 |
| Line2[r1,r2,c]: | 0.1307 | 0.0694 | 0.2369 | 0.1093 | 0.0480 | 0.2369 |
| Line3[r1,r2,c]: | 0.1307 | 0.0480 | 0.2369 | 0.1239 | 0.0640 | 0.2369 |

ML ratio (df=14 –13=1): 1.4161

***ML test Ctrl vs. Selection (r1, r2 and c are all line-specific)**

| | | | | | | |
|---|---|---|---|---|---|---|
| Line1[r1,r2,c]: | 0.0987 | 0.0453 | 0.0000 | 0.1173 | 0.0547 | 0.4164 |
| Line2[r1,r2,c]: | 0.1307 | 0.0693 | 0.1472 | 0.1093 | 0.0480 | 0.2545 |
| Line3[r1,r2,c]: | 0.1307 | 0.0480 | 0.2127 | 0.1240 | 0.0640 | 0.3367 |

ML ratio (df=18 –13=5): 2.7018

ML ratio for heterogeneity (control+selection; df = 5–1=4): 2.7018 – 1.4161

……………………………………………………………………………………………………

**markers 2-3-5 (*h-th-sr*)**

**CONTROL** **HYPEROXIA**

Bailey test for 3 × 750

| | | | | | | |
|---|---|---|---|---|---|---|
| Teta [r1,r2,c]: | 0.1186 | 0.1118 | 0.0891 | 0.1166 | 0.1229 | 0.1979 |
| SE [r1,r2,c]: | 0.0068 | 0.0066 | 0.0533 | 0.0068 | 0.0069 | 0.0755 |
| Chi2: | | 13.5991 | | | 4.2463 | |
| C Chi2: | | 0.1887 | | | 1.7236 | |

Bailey for 6 × 750:

| | | | |
|---|---|---|---|
| Teta: | 0.1175 | 0.1171 | 0.1253 |
| Chi2: | 20.5648 | | |
| C chi2: | 3.2732 | | |

**ML test Control vs. selection**

chi^2(ctrl + sel) - chi^2(ctrl) - chi^2(sel): 2.7194

C chi^2(ctrl + sel) - chi^2(ctrl) - chi^2(sel): **1.3608**

**ML test Ctrl Vs. Selection**

| | | | | | | |
|---|---|---|---|---|---|---|
| ML est [r1,r2,c] | 0.1200 | 0.1138 | 0.0977 | 0.1169 | 0.1236 | 0.2462 |
| ML SE [r1,r2,c] | 0.0069 | 0.0067 | 0.0557 | 0.0068 | 0.0069 | 0.0843 |

**ML test Ctrl vs. Selection (r1 and r2 are line-specific)**

c - control or selection specific

| | | | | | | |
|---|---|---|---|---|---|---|
| Line1[r1,r2,c]: | 0.0986 | 0.0960 | 0.0962 | 0.1173 | 0.1186 | 0.2456 |
| Line2[r1,r2,c]: | 0.1307 | 0.1387 | 0.0962 | 0.1095 | 0.1162 | 0.2456 |
| Line3[r1,r2,c]: | 0.1307 | 0.1067 | 0.0962 | 0.1239 | 0.1359 | 0.2456 |

c-global

| | | | | | | |
|---|---|---|---|---|---|---|
| Line1[r1,r2,c]: | 0.0987 | 0.0960 | 0.1728 | 0.1172 | 0.1185 | 0.1728 |
| Line2[r1,r2,c]: | 0.1309 | 0.1389 | 0.1728 | 0.1094 | 0.1161 | 0.1728 |
| Line3[r1,r2,c]: | 0.1308 | 0.1068 | 0.1728 | 0.1237 | 0.1357 | 0.1728 |

ML ratio (df=14 –13=1): 2.2389

***ML test Ctrl vs. Selection (r1, r2 and c are all line-specific)**

| | | | | | | |
|---|---|---|---|---|---|---|
| Line1[r1,r2,c]: | 0.0987 | 0.0960 | 0.1407 | 0.1173 | 0.1187 | 0.2873 |
| Line2[r1,r2,c]: | 0.1307 | 0.1387 | 0.0736 | 0.1093 | 0.1160 | 0.1051 |
| Line3[r1,r2,c]: | 0.1307 | 0.1067 | 0.0957 | 0.1240 | 0.1360 | 0.3163 |

ML ratio (df=18 –13=5): 3.8003

ML ratio for heterogeneity (control+selection; df = 5–1=4): 3.8003 – 2.2389

……………………………………………………………………………………………………

**markers 2-3-6 (*h-th-e*)**

**CONTROL** **HYPEROXIA**

**Lines (ML estimates)**

| | | |
|---|---|---|
| Line1 [r1,r2,c]: | 0.0987 0.1360 0.0994 | 0.1173 0.1640 0.1386 |
| Line2 [r1,r2,c]: | 0.1307 0.1880 0.1628 | 0.1093 0.1613 0.0756 |
| Line3 [r1,r2,c]: | 0.1307 0.1533 0.0665 | 0.1240 0.1853 0.3482 |

Bailey test for 3 × 750

| | | |
|---|---|---|
| Teta [r1,r2,c]: | 0.1185 0.1572 0.0980 | 0.1164 0.1695 0.1396 |
| SE [r1,r2,c]: | 0.0068 0.0077 0.0469 | 0.0068 0.0079 0.0540 |
| Chi2: | 15.0993 | 5.8411 |
| C Chi2: | 0.7319 | 3.1569 |

Bailey for 6 × 750:

| | |
|---|---|
| Teta: | 0.1174 0.1631 0.1159 |
| Chi2: | 22.5108 |
| C chi2: | 4.2062 |

**ML test Control vs. selection**

chi^2(ctrl + sel) - chi^2(ctrl) - chi^2(sel): 1.5704

C chi^2(ctrl + sel) - chi^2(ctrl) - chi^2(sel): **0.3174**

**ML test Ctrl Vs. Selection**

| | | |
|---|---|---|
| ML est [r1,r2,c] | 0.1200 0.1591 0.1164 | 0.1169 0.1702 0.2010 |
| ML SE [r1,r2,c] | 0.0069 0.0077 0.0512 | 0.0068 0.0079 0.0650 |

**ML test Ctrl vs. Selection (r1 and r2 are line-specific)**

c - control or selection specific

| | | | | | | |
|---|---|---|---|---|---|---|
| Line1[r1,r2,c]: | 0.0987 | 0.1360 | 0.1151 | 0.1174 | 0.1642 | 0.2009 |
| Line2[r1,r2,c]: | 0.1305 | 0.1878 | 0.1151 | 0.1095 | 0.1616 | 0.2009 |
| Line3[r1,r2,c]: | 0.1308 | 0.1535 | 0.1151 | 0.1236 | 0.1848 | 0.2009 |

c-global

| | | | | | | |
|---|---|---|---|---|---|---|
| Line1[r1,r2,c]: | 0.0987 | 0.1361 | 0.1585 | 0.1174 | 0.1641 | 0.1585 |
| Line2[r1,r2,c]: | 0.1306 | 0.1880 | 0.1585 | 0.1095 | 0.1615 | 0.1585 |
| Line3[r1,r2,c]: | 0.1309 | 0.1536 | 0.1585 | 0.1235 | 0.1846 | 0.1585 |

ML ratio (df=14 –13=1): 1.0934

***ML test Ctrl vs. Selection (r1, r2 and c are all line-specific)**

| | | | | | | |
|---|---|---|---|---|---|---|
| Line1[r1,r2,c]: | 0.0987 | 0.1360 | 0.0994 | 0.1173 | 0.1640 | 0.1386 |
| Line2[r1,r2,c]: | 0.1307 | 0.1880 | 0.1628 | 0.1093 | 0.1613 | 0.0756 |
| Line3[r1,r2,c]: | 0.1307 | 0.1533 | 0.0665 | 0.1240 | 0.1853 | 0.3482 |

ML ratio (df=18 –13=5): 5.2334

ML ratio for heterogeneity (control+selection; df = 5–1=4): 5.2334 – 1.0934

……………………………………………………………………………………………………

**markers 1-4-5 (*ru-cu-sr*)**

| | CONTROL | | | HYPEROXIA | | |
|---|---|---|---|---|---|---|
| **Lines (ML estimates)** | | | | | | |
| Line1 [r1,r2,c]: | 0.2653 | 0.0533 | 0.1884 | 0.2960 | 0.0640 | 0.0704 |
| Line2 [r1,r2,c]: | 0.3213 | 0.0693 | 0.1796 | 0.2800 | 0.0680 | 0.1401 |
| Line3 [r1,r2,c]: | 0.3189 | 0.0587 | 0.0000 | 0.3120 | 0.0720 | 0.1781 |
| Bailey test for 3 × 750 | | | | | | |
| Teta [r1,r2,c]: | 0.3002 | 0.0600 | 0.0000 | 0.2956 | 0.0679 | 0.1144 |
| SE [r1,r2,c]: | 0.0096 | 0.0050 | 0.0002 | 0.0096 | 0.0053 | 0.0490 |
| Chi2: | | 15.3686 | | | 3.2406 | |
| C Chi2: | | 5.3744 | | | 0.8862 | |

Bailey for 6 × 750:

| | | | |
|---|---|---|---|
| Teta: | 0.2976 | 0.0636 | 0.0000 |
| Chi2: | 25.2521 | | |
| C chi2: | 11.7453 | | |

**ML test Control vs. selection**

chi^2(ctrl + sel) - chi^2(ctrl) - chi^2(sel): 6.6430

C chi^2(ctrl + sel) - chi^2(ctrl) - chi^2(sel): **5.4847**

**ML test Ctrl Vs. Selection**

| | | | | | | |
|---|---|---|---|---|---|---|
| ML est [r1,r2,c] | 0.3018 | 0.0604 | 0.1218 | 0.2960 | 0.0680 | 0.1325 |
| ML SE [r1,r2,c] | 0.0097 | 0.0050 | 0.0532 | 0.0096 | 0.0053 | 0.0527 |

**ML test Ctrl vs. Selection (r1 and r2 are line-specific)**

c - control or selection specific

| | | | | | | |
|---|---|---|---|---|---|---|
| Line1[r1,r2,c]: | 0.2651 | 0.0533 | 0.1209 | 0.2963 | 0.0641 | 0.1324 |
| Line2[r1,r2,c]: | 0.3210 | 0.0692 | 0.1209 | 0.2800 | 0.0680 | 0.1324 |
| Line3[r1,r2,c]: | 0.3193 | 0.0588 | 0.1209 | 0.3117 | 0.0719 | 0.1324 |

c-global

| | | | | | | |
|---|---|---|---|---|---|---|
| Line1[r1,r2,c]: | 0.2651 | 0.0533 | 0.1269 | 0.2963 | 0.0641 | 0.1269 |
| Line2[r1,r2,c]: | 0.3210 | 0.0693 | 0.1269 | 0.2799 | 0.0680 | 0.1269 |
| Line3[r1,r2,c]: | 0.3194 | 0.0588 | 0.1269 | 0.3117 | 0.0719 | 0.1269 |

ML ratio (df=14 –13=1): 0.0240

***ML test Ctrl vs. Selection (r1, r2 and c are all line-specific)**

| | | | | | | |
|---|---|---|---|---|---|---|
| Line1[r1,r2,c]: | 0.2653 | 0.0533 | 0.1884 | 0.2960 | 0.0640 | 0.0704 |
| Line2[r1,r2,c]: | 0.3213 | 0.0693 | 0.1796 | 0.2800 | 0.0680 | 0.1401 |

| | | | | | | |
|---|---|---|---|---|---|---|
| Line3[r1,r2,c]: | 0.3189 | 0.0587 | 0.0000 | 0.3120 | 0.0720 | 0.1781 |

ML ratio (df=18 –13=5): 5.0740

ML ratio for heterogeneity (control+selection; df = 5–1=4): 5.0740– 0.0240

…………………………………………………………………………………………………

**<u>markers 1-4-6 (*ru-cu-e*)</u>**

**<u>Lines (ML estimates)</u>**

| | CONTROL | | | HYPEROXIA | | |
|---|---|---|---|---|---|---|
| Line1 [r1,r2,c]: | 0.2653 | 0.0933 | 0.2154 | 0.2960 | 0.1093 | 0.1236 |
| Line2 [r1,r2,c]: | 0.3213 | 0.1187 | 0.1748 | 0.2800 | 0.1133 | 0.0840 |
| Line3 [r1,r2,c]: | 0.3187 | 0.1053 | 0.0397 | 0.3120 | 0.1213 | 0.1761 |

Bailey test for 3 × 750

| | | | | | | |
|---|---|---|---|---|---|---|
| Teta [r1,r2,c]: | 0.3006 | 0.1053 | 0.0827 | 0.2956 | 0.1145 | 0.1198 |
| SE [r1,r2,c]: | 0.0096 | 0.0065 | 0.0330 | 0.0096 | 0.0067 | 0.0385 |
| Chi2: | | 16.1134 | | | 3.5596 | |
| C Chi2: | | 4.3638 | | | 0.9370 | |

Bailey for 6 × 750:

| | | | |
|---|---|---|---|
| Teta: | 0.2981 | 0.1097 | 0.0984 |
| Chi2: | 21.1887 | | |
| C chi2: | 5.8092 | | |

**<u>ML test Control vs. selection</u>**

chi^2(ctrl + sel) - chi^2(ctrl) - chi^2(sel): 1.5157

C chi^2(ctrl + sel) - chi^2(ctrl) - chi^2(sel): **<u>0.5084</u>**

**<u>ML test Ctrl Vs. Selection</u>**

| | | | | | | |
|---|---|---|---|---|---|---|
| ML est [r1,r2,c] | 0.3018 | 0.1058 | 0.1392 | 0.2960 | 0.1147 | 0.1309 |
| ML SE [r1,r2,c] | 0.0097 | 0.0065 | 0.0427 | 0.0096 | 0.0067 | 0.0402 |

**<u>ML test Ctrl vs. Selection (r1 and r2 are line-specific)</u>**

c - control or selection specific

| | | | | | | |
|---|---|---|---|---|---|---|
| Line1[r1,r2,c]: | 0.2649 | 0.0932 | 0.1381 | 0.2961 | 0.1094 | 0.1309 |
| Line2[r1,r2,c]: | 0.3209 | 0.1185 | 0.1381 | 0.2804 | 0.1135 | 0.1309 |
| Line3[r1,r2,c]: | 0.3196 | 0.1057 | 0.1381 | 0.3115 | 0.1211 | 0.1309 |

c-global

| | | | | | | |
|---|---|---|---|---|---|---|
| Line1[r1,r2,c]: | 0.2648 | 0.0932 | 0.1344 | 0.2961 | 0.1094 | 0.1344 |
| Line2[r1,r2,c]: | 0.3209 | 0.1185 | 0.1344 | 0.2804 | 0.1135 | 0.1344 |
| Line3[r1,r2,c]: | 0.3196 | 0.1056 | 0.1344 | 0.3115 | 0.1212 | 0.1344 |

ML ratio (df=14 –13=1): 0.0151

***ML test Ctrl vs. Selection (r1, r2 and c are all line-specific)**

| | | | | | | |
|---|---|---|---|---|---|---|
| Line1[r1,r2,c]: | 0.2653 | 0.0933 | 0.2154 | 0.2960 | 0.1093 | 0.1236 |
| Line2[r1,r2,c]: | 0.3213 | 0.1187 | 0.1748 | 0.2800 | 0.1133 | 0.0840 |
| Line3[r1,r2,c]: | 0.3187 | 0.1053 | 0.0397 | 0.3120 | 0.1213 | 0.1761 |

ML ratio (df=18 –13=5): 4.5173

ML ratio for heterogeneity (control+selection; df = 5–1=4): 4.5173 – 0.0151
…………………………………………………………………………………………………

## markers 2-4-5 (*h-cu-sr*)

| | CONTROL | | | HYPEROXIA | | |
|---|---|---|---|---|---|---|
| **Lines (ML estimates)** | | | | | | |
| Line1 [r1,r2,c]: | 0.1440 | 0.0533 | 0.3474 | 0.1667 | 0.0640 | 0.1250 |
| Line2 [r1,r2,c]: | 0.1974 | 0.0693 | 0.0000 | 0.1547 | 0.0680 | 0.0000 |
| Line3 [r1,r2,c]: | 0.1760 | 0.0587 | 0.0000 | 0.1827 | 0.0720 | 0.2028 |
| Bailey test for 3 × 750 | | | | | | |
| Teta [r1,r2,c]: | 0.1704 | 0.0602 | 0.0000 | 0.1672 | 0.0678 | 0.0000 |
| SE [r1,r2,c]: | 0.0079 | 0.0050 | 0.0003 | 0.0079 | 0.0053 | 0.0003 |
| Chi2: | | 12.6281 | | | 5.6817 | |
| C Chi2: | | 2.1506 | | | 3.1448 | |

Bailey for 6 × 750:

| | | | |
|---|---|---|---|
| Teta: | 0.1688 | 0.0638 | 0.0000 |
| Chi2: | 19.4203 | | |
| C chi2: | 5.2954 | | |

**ML test Control vs. selection**

chi^2(ctrl + sel) - chi^2(ctrl) - chi^2(sel): 1.1105

C chi^2(ctrl + sel) - chi^2(ctrl) - chi^2(sel): **0.0000**

**ML test Ctrl Vs. Selection**

| | | | | | | |
|---|---|---|---|---|---|---|
| ML est [r1,r2,c] | 0.1724 | 0.0604 | 0.0853 | 0.1680 | 0.0680 | 0.1167 |
| ML SE [r1,r2,c] | 0.0080 | 0.0050 | 0.0597 | 0.0079 | 0.0053 | 0.0664 |

**ML test Ctrl vs. Selection (r1 and r2 are line-specific)**

c - control or selection specific

| | | | | | | |
|---|---|---|---|---|---|---|
| Line1[r1,r2,c]: | 0.1437 | 0.0532 | 0.0838 | 0.1666 | 0.0640 | 0.1166 |
| Line2[r1,r2,c]: | 0.1975 | 0.0694 | 0.0838 | 0.1548 | 0.0681 | 0.1166 |
| Line3[r1,r2,c]: | 0.1761 | 0.0587 | 0.0838 | 0.1825 | 0.0719 | 0.1166 |

c-global

| | | | | | | |
|---|---|---|---|---|---|---|
| Line1[r1,r2,c]: | 0.1437 | 0.0532 | 0.1006 | 0.1666 | 0.0640 | 0.1006 |

| | | | | | | |
|---|---|---|---|---|---|---|
| Line2[r1,r2,c]: | 0.1976 | 0.0694 | 0.1006 | 0.1548 | 0.0681 | 0.1006 |
| Line3[r1,r2,c]: | 0.1762 | 0.0587 | 0.1006 | 0.1824 | 0.0719 | 0.1006 |

ML ratio (df=14 –13=1): 0.1366

***ML test Ctrl vs. Selection (r1, r2 and c are all line-specific)**

| | | | | | | |
|---|---|---|---|---|---|---|
| Line1[r1,r2,c]: | 0.1440 | 0.0533 | 0.3474 | 0.1667 | 0.0640 | 0.1250 |
| Line2[r1,r2,c]: | 0.1974 | 0.0693 | 0.0000 | 0.1547 | 0.0680 | 0.0000 |
| Line3[r1,r2,c]: | 0.1760 | 0.0587 | 0.0000 | 0.1827 | 0.0720 | 0.2028 |

ML ratio (df=18 –13=5): 8.3264

ML ratio for heterogeneity (control+selection; df = 5–1=4): 8.3264 – 0.1366
……………………………………………………………………………………………………

## markers 2-4-6 (*h-cu-e*)

| | CONTROL | | | HYPEROXIA | | |
|---|---|---|---|---|---|---|
| **Lines (ML estimates)** | | | | | | |
| Line1 [r1,r2,c]: | 0.1440 | 0.0933 | 0.1984 | 0.1667 | 0.1094 | 0.0000 |
| Line2 [r1,r2,c]: | 0.1973 | 0.1187 | 0.1139 | 0.1547 | 0.1134 | 0.0000 |
| Line3 [r1,r2,c]: | 0.1761 | 0.1054 | 0.0000 | 0.1827 | 0.1213 | 0.2406 |
| Bailey test for 3 × 750 | | | | | | |
| Teta [r1,r2,c]: | 0.1703 | 0.1052 | 0.0000 | 0.1671 | 0.1143 | 0.0000 |
| SE [r1,r2,c]: | 0.0079 | 0.0065 | 0.0002 | 0.0079 | 0.0067 | 0.0002 |
| Chi2: | | 15.9159 | | | 7.0310 | |
| C Chi2: | | 4.1846 | | | 4.3343 | |

Bailey for 6 × 750:

| | | | |
|---|---|---|---|
| Teta: | 0.1687 | 0.1096 | 0.0000 |
| Chi2: | 23.9077 | | |
| C chi2: | 8.5190 | | |

**ML test Control vs. selection**
chi^2(ctrl + sel) - chi^2(ctrl) - chi^2(sel): 0.9608
C chi^2(ctrl + sel) - chi^2(ctrl) - chi^2(sel): **0.0000**

**ML test Ctrl Vs. Selection**

| | | | | | | |
|---|---|---|---|---|---|---|
| ML est [r1,r2,c] | 0.1724 | 0.1058 | 0.0975 | 0.1680 | 0.1147 | 0.0923 |
| ML SE [r1,r2,c] | 0.0080 | 0.0065 | 0.0480 | 0.0079 | 0.0067 | 0.0455 |

**ML test Ctrl vs. Selection (r1 and r2 are line-specific)**

c - control or selection specific

| | | | | | | |
|---|---|---|---|---|---|---|
| Line1[r1,r2,c]: | 0.1438 | 0.0932 | 0.0962 | 0.1669 | 0.1095 | 0.0923 |

| | | | | | | |
|---|---|---|---|---|---|---|
| Line2[r1,r2,c]: | 0.1973 | 0.1186 | 0.0962 | 0.1549 | 0.1135 | 0.0923 |
| Line3[r1,r2,c]: | 0.1763 | 0.1055 | 0.0962 | 0.1821 | 0.1210 | 0.0923 |

c-global

| | | | | | | |
|---|---|---|---|---|---|---|
| Line1[r1,r2,c]: | 0.1438 | 0.0932 | 0.0941 | 0.1669 | 0.1095 | 0.0941 |
| Line2[r1,r2,c]: | 0.1972 | 0.1186 | 0.0941 | 0.1549 | 0.1135 | 0.0941 |
| Line3[r1,r2,c]: | 0.1763 | 0.1055 | 0.0941 | 0.1821 | 0.1210 | 0.0941 |

ML ratio (df=14 –13=1): 0.0036

**<u>*ML test Ctrl vs. Selection (r1, r2 and c are all line-specific)</u>**

| | | | | | | |
|---|---|---|---|---|---|---|
| Line1[r1,r2,c]: | 0.1440 | 0.0933 | 0.1984 | 0.1667 | 0.1094 | 0.0000 |
| Line2[r1,r2,c]: | 0.1973 | 0.1187 | 0.1139 | 0.1547 | 0.1134 | 0.0000 |
| Line3[r1,r2,c]: | 0.1761 | 0.1054 | 0.0000 | 0.1827 | 0.1213 | 0.2406 |

ML ratio (df=18 –13=5): 11.5088

ML ratio for heterogeneity (control+selection; df = 5–1=4): 11.5088 – 0.0036

……………………………………………………………………………………………………

## <u>markers 1-5-6 (*ru-sr-e*)</u>

**CONTROL** **HYPEROXIA**

**<u>Lines (ML estimates)</u>**

| | CONTROL | | | HYPEROXIA | | |
|---|---|---|---|---|---|---|
| Line1 [r1,r2,c]: | 0.3133 | 0.0400 | 0.2127 | 0.3573 | 0.0480 | 0.2332 |
| Line2 [r1,r2,c]: | 0.3827 | 0.0493 | 0.1413 | 0.3428 | 0.0453 | 0.0000 |
| Line3 [r1,r2,c]: | 0.3773 | 0.0467 | 0.0757 | 0.3760 | 0.0493 | 0.1438 |

Bailey test for 3 × 750

| | | | | | | |
|---|---|---|---|---|---|---|
| Teta [r1,r2,c]: | 0.3566 | 0.0453 | 0.1145 | 0.3578 | 0.0474 | 0.0000 |
| SE [r1,r2,c]: | 0.0101 | 0.0044 | 0.0546 | 0.0101 | 0.0045 | 0.0001 |
| Chi2: | | 12.7391 | | | 7.4840 | |
| C Chi2: | | 0.8102 | | | 5.4636 | |

Bailey for 6 × 750:

| | | | |
|---|---|---|---|
| Teta: | 0.3569 | 0.0463 | 0.0000 |
| Chi2: | 24.7723 | | |
| C chi2: | 10.8034 | | |

**<u>ML test Control vs. selection</u>**

chi^2(ctrl + sel) - chi^2(ctrl) - chi^2(sel): 4.5492

C chi^2(ctrl + sel) - chi^2(ctrl) - chi^2(sel): **<u>4.5296</u>**

**<u>ML test Ctrl Vs. Selection</u>**

| | | | | | | |
|---|---|---|---|---|---|---|
| ML est [r1,r2,c] | 0.3578 | 0.0453 | 0.1370 | 0.3587 | 0.0476 | 0.1303 |
| ML SE [r1,r2,c] | 0.0101 | 0.0044 | 0.0595 | 0.0101 | 0.0045 | 0.0567 |

**<u>ML test Ctrl vs. Selection (r1 and r2 are line-specific)</u>**

c - control or selection specific

| | | | | | | |
|---|---|---|---|---|---|---|
| Line1[r1,r2,c]: | 0.3131 | 0.0400 | 0.1358 | 0.3568 | 0.0479 | 0.1303 |
| Line2[r1,r2,c]: | 0.3826 | 0.0493 | 0.1358 | 0.3433 | 0.0454 | 0.1303 |
| Line3[r1,r2,c]: | 0.3777 | 0.0467 | 0.1358 | 0.3759 | 0.0493 | 0.1303 |

c-global

| | | | | | | |
|---|---|---|---|---|---|---|
| Line1[r1,r2,c]: | 0.3130 | 0.0400 | 0.1330 | 0.3568 | 0.0479 | 0.1330 |
| Line2[r1,r2,c]: | 0.3826 | 0.0493 | 0.1330 | 0.3433 | 0.0454 | 0.1330 |
| Line3[r1,r2,c]: | 0.3777 | 0.0467 | 0.1330 | 0.3759 | 0.0493 | 0.1330 |

ML ratio (df=14 –13=1): 0.0046

**<u>*ML test Ctrl vs. Selection (r1, r2 and c are all line-specific)</u>**

| | | | | | | |
|---|---|---|---|---|---|---|
| Line1[r1,r2,c]: | 0.3133 | 0.0400 | 0.2127 | 0.3573 | 0.0480 | 0.2332 |
| Line2[r1,r2,c]: | 0.3827 | 0.0493 | 0.1413 | 0.3428 | 0.0453 | 0.0000 |
| Line3[r1,r2,c]: | 0.3773 | 0.0467 | 0.0757 | 0.3760 | 0.0493 | 0.1438 |

ML ratio (df=18 –13=5): 4.8630

ML ratio for heterogeneity (control+selection; df = 5–1=4): 4.8630 – 0.0046
……………………………………………………………………………………………………

**<u>markers 2-5-6 (*h-sr-e*)</u>**

| | CONTROL | | | HYPEROXIA | | |
|---|---|---|---|---|---|---|
| **<u>Lines (ML estimates)</u>** | | | | | | |
| Line1 [r1,r2,c]: | 0.1920 | 0.0400 | 0.0000 | 0.2280 | 0.0480 | 0.0000 |
| Line2 [r1,r2,c]: | 0.2667 | 0.0493 | 0.2028 | 0.2227 | 0.0453 | 0.0000 |
| Line3 [r1,r2,c]: | 0.2347 | 0.0467 | 0.0000 | 0.2493 | 0.0493 | 0.2169 |
| Bailey test for 3 × 750 | | | | | | |
| Teta [r1,r2,c]: | 0.2281 | 0.0451 | 0.0000 | 0.2328 | 0.0474 | 0.0000 |
| SE [r1,r2,c]: | 0.0088 | 0.0044 | 0.0003 | 0.0089 | 0.0045 | 0.0002 |
| Chi2: | | 15.7674 | | | 3.9434 | |
| C Chi2: | | 2.1435 | | | 2.1446 | |

Bailey for 6 × 750:

| | | | |
|---|---|---|---|
| Teta: | 0.2304 | 0.0462 | 0.0000 |
| Chi2: | 20.0305 | | |
| C chi2: | 4.2881 | | |

**<u>ML test Control vs. selection</u>**
chi^2(ctrl + sel) - chi^2(ctrl) - chi^2(sel): 0.3197
C chi^2(ctrl + sel) - chi^2(ctrl) - chi^2(sel): **<u>0.0000</u>**

**<u>ML test Ctrl Vs. Selection</u>**

| | | | | | | |
|---|---|---|---|---|---|---|
| ML est [r1,r2,c] | 0.2311 | 0.0453 | 0.0848 | 0.2333 | 0.0476 | 0.0801 |
| ML SE [r1,r2,c] | 0.0089 | 0.0044 | 0.0593 | 0.0089 | 0.0045 | 0.0560 |

**ML test Ctrl vs. Selection (r1 and r2 are  line-specific)**

c - control or selection specific

| | | | | | | |
|---|---|---|---|---|---|---|
| Line1[r1,r2,c]: | 0.1921 | 0.0400 | 0.0841 | 0.2282 | 0.0480 | 0.0801 |
| Line2[r1,r2,c]: | 0.2663 | 0.0493 | 0.0841 | 0.2228 | 0.0454 | 0.0801 |
| Line3[r1,r2,c]: | 0.2349 | 0.0467 | 0.0841 | 0.2489 | 0.0493 | 0.0801 |

c-global

| | | | | | | |
|---|---|---|---|---|---|---|
| Line1[r1,r2,c]: | 0.1921 | 0.0400 | 0.0821 | 0.2282 | 0.0480 | 0.0821 |
| Line2[r1,r2,c]: | 0.2663 | 0.0493 | 0.0821 | 0.2228 | 0.0454 | 0.0821 |
| Line3[r1,r2,c]: | 0.2349 | 0.0467 | 0.0821 | 0.2490 | 0.0493 | 0.0821 |

ML ratio (df=14 –13=1): 0.0025

***ML test Ctrl vs. Selection (r1, r2 and c  are all line-specific)**

| | | | | | | |
|---|---|---|---|---|---|---|
| Line1[r1,r2,c]: | 0.1920 | 0.0400 | 0.0000 | 0.2280 | 0.0480 | 0.0000 |
| Line2[r1,r2,c]: | 0.2667 | 0.0493 | 0.2028 | 0.2227 | 0.0453 | 0.0000 |
| Line3[r1,r2,c]: | 0.2347 | 0.0467 | 0.0000 | 0.2493 | 0.0493 | 0.2169 |

ML ratio (df=18 –13=5): 7.6778

ML ratio for heterogeneity (control+selection; df = 5–1=4): 7.6778 – 0.0025
…………………………………………………………………………………………………………
