# Supplementary material for: Experimental evolution of recombination and crossover interference in Drosophila caused by directional selection for stress-related traits
Source: BMC Biol. 2015 Nov 27;13:101. doi: 10.1186/s12915-015-0206-5 (PMC4661966; doi:10.1186/s12915-015-0206-5)
Supplement: Additional file 5: — Estimates of the coefficients of coincidence for non-adjacent intervals per replicate and entire variants (selection and control). (PDF 2103 kb) [file 12915_2015_206_MOESM5_ESM.pdf]

Additional file 5

Non-adjacent intervals

For each experiment (desiccation, hypoxia, hyperoxia), corresponding ML-estimates of the vectors of parameters [r1,r2,c] for each replicate line of control and selection variants are shown. For the entire Control variants and Selection variants, vectors of weighted estimates Teta [r1,r2,c] and corresponding vectors SE [r1,r2,c] are provided. In addition, chi-square for between-replicates heterogeneity of either Control or Selection are provided for the vectors [r1,r2,c] (statistics Chi2) and for coefficients of coincidence (statistics C Chi2) as described in Materials and Methods. In the same way, the total heterogeneity of all replicates taken over Control+Selection was calculated for the vector [r1,r2,c] (statistics Chi2) and for coefficients of coincidence (statistics C Chi2). Finally, chi-square for Control vs. Selection difference for the entire vector [r1,r2,c] is calculated as chi^2(ctrl+sel)-chi^2(sel)-chi^2(ctrl) and for the confident of coincidence as C chi^2(ctrl + sel) - chi^2(ctrl) - chi^2(sel) (see Materials and Methods).

We also employed the standard and more direct ML approach allowing for each line, in both selection and control, to have its own $r1_k$ and $r2_k$. Namely, to test for significance of the differences of c values in selection and control for earch pair of intervals, we performed log-likelihood ratio test to compare the model with 3×2+3×2=12 line-specific r-values plus $c_{control}$ plus $c_{selection}$ (in total 14 parameters) versus corresponding model with 12 line-specific r-values plus c_{global} (13 parameters). Thus, H_0 and H_1 hypotheses are specified by 14 and 13 parameters. The (doubled) log-likelihood ratio test statistics for significance of the difference in c between control and selection is asymptotically distributed as χ^2 with df=1 (present in the tables as: ML ratio (df=14 −13=1). To facilitate the comparison of the c values in the model with 14 parameters (where $c_{control}$ and $c_{selection}$ are line-independent) with those in the full model with 18 parameters (with line-specific c-values), we repeated the corresponding outputs at the end of each marker combination, and marked by asterisk.

(A) Desiccation experiment

X chromosome

markers 1-2, 3-4 (*y-cv, v-f*)

	CONTROL	DESICCATION
**Lines (ML estimates)**		
Line1 [r1,r2,c]:	0.1010 0.2095 1.1258	0.1248 0.3429 1.3805
Line2 [r1,r2,c]:	0.1077 0.2231 0.9521	0.1143 0.3095 1.2923
Line3 [r1,r2,c]:	0.1000 0.2248 0.8898	0.0876 0.3124 1.0786
Bailey test for 3 × 1050		
Teta [r1,r2,c]:	0.1029 0.2190 0.9781	0.1067 0.3207 1.2832

SE [r1,r2,c]:	0.0054 0.0074 0.0985	0.0055 0.0083 0.0766
Chi2:	2.1852	14.2926
C Chi2:	0.9433	2.5334

Bailey for 6 × 1050:

Teta:	0.1037 0.2641 1.1878
Chi2:	107.6205
C chi2:	9.2316

chi^2(ctrl + sel) - chi^2(ctrl) - chi^2(sel): 91.1427
C chi^2(ctrl + sel) - chi^2(ctrl) - chi^2(sel): **5.7549**

ML test Ctrl Vs. Selection

ML est [r1,r2,c]	0.1029 0.2191 0.9861	0.1089 0.3216 1.2783
ML SE [r1,r2,c]	0.0054 0.0074 0.0989	0.0056 0.0083 0.0776

ML test Ctrl vs. Selection (r1 and r2 are line-specific)

c - control or selection specific

Line1[r1,r2,c]:	0.1009	0.2095	0.9848	0.1251	0.3437	1.2777
Line2[r1,r2,c]:	0.1077	0.2231	0.9848	0.1143	0.3096	1.2777
Line3[r1,r2,c]:	0.1000	0.2248	0.9848	0.0874	0.3115	1.2777

c-global

Line1[r1,r2,c]:	0.1009	0.2095	1.1760	0.1251	0.3439	1.1760
Line2[r1,r2,c]:	0.1076	0.2227	1.1760	0.1144	0.3099	1.1760
Line3[r1,r2,c]:	0.0998	0.2244	1.1760	0.0875	0.3121	1.1760

ML ratio (df=14 – 13=1): 5.2465

***ML test Ctrl vs. Selection (r1, r2 and c are all line-specific)**

Line1[r1,r2,c]:	0.1010	0.2095	1.1258	0.1248	0.3429	1.3805
Line2[r1,r2,c]:	0.1077	0.2231	0.9521	0.1143	0.3095	1.2923
Line3[r1,r2,c]:	0.1000	0.2248	0.8898	0.0876	0.3124	1.0786

ML ratio (df=18 – 13=5): 8.6481

ML ratio for heterogeneity (control+selection; df = 5–1=4): 8.6481 – 5.2465

……………………………………………………………………………………………………

markers 1-2, 3-4 (*net-dp*, *b-pk*)

	CONTROL	DESICCATION
**Lines (ML estimates)**		
Line1 [r1,r2,c]:	0.1187 0.0467 0.2413	0.1640 0.0560 0.4358
Line2 [r1,r2,c]:	0.1053 0.0627 0.4034	0.1773 0.0507 0.2971
Line3 [r1,r2,c]:	0.0987 0.0560 0.4833	0.1667 0.0587 0.2728

Bailey test for 3 × 750						
Teta [r1,r2,c]:	0.1070	0.0544	0.3492	0.1692	0.0549	0.3215
SE [r1,r2,c]:	0.0065	0.0048	0.1574	0.0079	0.0048	0.1191
Chi2:		3.7792			1.2697	
C Chi2:		0.4144			0.3111	

Bailey for 6 × 750:

Teta:	0.1322	0.0550	0.3276
Chi2:	42.1596		
C chi2:	0.7478		

chi^2(ctrl + sel) - chi^2(ctrl) - chi^2(sel): 37.1107
C chi^2(ctrl + sel) - chi^2(ctrl) - chi^2(sel): **0.0222**

ML test Ctrl Vs. Selection

ML est [r1,r2,c]	0.1076	0.0551	0.3760	0.1693	0.0551	0.3333
ML SE [r1,r2,c]	0.0065	0.0048	0.1627	0.0079	0.0048	0.1211

ML test Ctrl vs. Selection (r1 and r2 are line-specific)

c - control or selection specific

Line1[r1,r2,c]:	0.1187	0.0467	0.3791	0.1639	0.0560	0.3337
Line2[r1,r2,c]:	0.1053	0.0627	0.3791	0.1774	0.0507	0.3337
Line3[r1,r2,c]:	0.0986	0.0560	0.3791	0.1667	0.0587	0.3337

c-global

Line1[r1,r2,c]:	0.1187	0.0467	0.3504	0.1639	0.0560	0.3504
Line2[r1,r2,c]:	0.1053	0.0626	0.3504	0.1774	0.0507	0.3504
Line3[r1,r2,c]:	0.0986	0.0560	0.3504	0.1668	0.0587	0.3504

ML ratio (df=14 – 13=1): 0.0462

***ML test Ctrl vs. Selection (r1, r2 and c are all line-specific)**

Line1[r1,r2,c]:	0.1187	0.0467	0.2413	0.1640	0.0560	0.4358
Line2[r1,r2,c]:	0.1053	0.0627	0.4034	0.1773	0.0507	0.2971
Line3[r1,r2,c]:	0.0987	0.0560	0.4833	0.1667	0.0587	0.2728

ML ratio (df=18 – 13=5): 0.7622

ML ratio for heterogeneity (control+selection; df = 5–1=4): 0.7622 – 0.0462

……………………………………………………………………………………………..

markers 1-2, 3-5 (*net-dp*, *b-cn*)

CONTROL **DESICCATION**

Lines (ML estimates)

Line1 [r1,r2,c]:	0.1187 0.0653 0.1720	0.1640 0.0800 0.4065
Line2 [r1,r2,c]:	0.1053 0.0853 0.2968	0.1773 0.0680 0.2212
Line3 [r1,r2,c]:	0.0986 0.0773 0.5256	0.1667 0.0760 0.2105

Bailey test for 3 × 750

Teta [r1,r2,c]:	0.1070 0.0753 0.2735	0.1691 0.0744 0.2591
SE [r1,r2,c]:	0.0065 0.0056 0.1187	0.0079 0.0055 0.0921
Chi2:	4.7682	2.0041
C Chi2:	1.1348	0.7592

Bailey for 6 × 750:
Teta: 0.1321 0.0753 0.2612
Chi2: 43.7823
C chi2: 1.9061

chi^2(ctrl + sel) - chi^2(ctrl) - chi^2(sel): 37.0099
C chi^2(ctrl + sel) - chi^2(ctrl) - chi^2(sel): **0.0122**

ML test Ctrl Vs. Selection

ML est [r1,r2,c]	0.1075 0.0760 0.3262	0.1693 0.0747 0.2813
ML SE [r1,r2,c]	0.0065 0.0056 0.1291	0.0079 0.0055 0.0959

ML test Ctrl vs. Selection (r1 and r2 are line-specific)

c - control or selection specific

Line1[r1,r2,c]:	0.1188	0.0654	0.3280	0.1638	0.0799	0.2818
Line2[r1,r2,c]:	0.1054	0.0853	0.3280	0.1774	0.0680	0.2818
Line3[r1,r2,c]:	0.0986	0.0772	0.3280	0.1668	0.0760	0.2818

c-global

Line1[r1,r2,c]:	0.1187	0.0654	0.2994	0.1638	0.0799	0.2994
Line2[r1,r2,c]:	0.1053	0.0853	0.2994	0.1775	0.0680	0.2994
Line3[r1,r2,c]:	0.0985	0.0772	0.2994	0.1668	0.0761	0.2994

ML ratio (df=14 – 13=1): 0.0837

***ML test Ctrl vs. Selection (r1, r2 and c are all line-specific)**

Line1[r1,r2,c]:	0.1187	0.0653	0.1720	0.1640	0.0800	0.4065
Line2[r1,r2,c]:	0.1053	0.0853	0.2968	0.1773	0.0680	0.2212
Line3[r1,r2,c]:	0.0986	0.0773	0.5256	0.1667	0.0760	0.2105

ML ratio (df=18 – 13=5): 2.1222

ML ratio for heterogeneity (control+selection; df = 5–1=4): 2.1222 – 0.0837

…………………………………………………………………………………………..

markers 1-2, 4-5 (*net-dp, pk-cn*)

	CONTROL	DESICCATION
**Lines (ML estimates)**		
Line1 [r1,r2,c]:	0.1187 0.0187 0.0000	0.1640 0.0240 0.3412
Line2 [r1,r2,c]:	0.1053 0.0227 0.0000	0.1773 0.0173 0.0000
Line3 [r1,r2,c]:	0.0987 0.0213 0.6340	0.1667 0.0173 0.0000
Bailey test for 3 × 750		
Teta [r1,r2,c]:	0.1070 0.0208 0.0000	0.1691 0.0191 0.0000
SE [r1,r2,c]:	0.0065 0.0030 0.0001	0.0079 0.0029 0.0003
Chi2:	2.9364	2.6101
C Chi2:	1.0825	1.0782

Bailey for 6 × 750:
Teta: 0.1321 0.0201 0.0000
Chi2: 42.3529
C chi2: 2.1607

chi^2(ctrl + sel) - chi^2(ctrl) - chi^2(sel): 36.8065
C chi^2(ctrl + sel) - chi^2(ctrl) - chi^2(sel): **0.0000**

**ML test Ctrl Vs. Selection**		
ML est [r1,r2,c]	0.1075 0.0209 0.1989	0.1693 0.0196 0.1338
ML SE [r1,r2,c]	0.0065 0.0030 0.1958	0.0079 0.0029 0.1323

ML test Ctrl vs. Selection (r1 and r2 are line-specific)

c - control or selection specific						
Line1[r1,r2,c]:	0.1187	0.0187	0.2040	0.1639	0.0240	0.1345
Line2[r1,r2,c]:	0.1053	0.0227	0.2040	0.1774	0.0173	0.1345
Line3[r1,r2,c]:	0.0986	0.0213	0.2040	0.1667	0.0173	0.1345
c-global						
Line1[r1,r2,c]:	0.1187	0.0187	0.1623	0.1639	0.0240	0.1623
Line2[r1,r2,c]:	0.1054	0.0227	0.1623	0.1774	0.0174	0.1623
Line3[r1,r2,c]:	0.0986	0.0213	0.1623	0.1667	0.0173	0.1623

ML ratio (df=14 – 13=1): 0.0761

***ML test Ctrl vs. Selection (r1, r2 and c are all line-specific)**

Line1[r1,r2,c]:	0.1187	0.0187	0.0000	0.1640	0.0240	0.3412
Line2[r1,r2,c]:	0.1053	0.0227	0.0000	0.1773	0.0173	0.0000
Line3[r1,r2,c]:	0.0987	0.0213	0.6340	0.1667	0.0173	0.0000

ML ratio (df=18 – 13=5): 4.3355

ML ratio for heterogeneity (control+selection; df = 5–1=4): 4.3355 – 0.0761

……………………………………………………………………………………………..

markers 1-3, 4-5 (*net-b*, *pk-cn*)

	CONTROL			DESICCATION		
**Lines (ML estimates)**						
Line1 [r1,r2,c]:	0.3907	0.0187	0.0000	0.3933	0.0240	0.2822
Line2 [r1,r2,c]:	0.3801	0.0227	0.0000	0.3667	0.0173	0.0000
Line3 [r1,r2,c]:	0.3667	0.0213	0.1702	0.3867	0.0173	0.0000
Bailey test for 3 × 750						
Teta [r1,r2,c]:	0.3789	0.0208	0.0000	0.3818	0.0191	0.0000
SE [r1,r2,c]:	0.0102	0.0030	0.0003	0.0102	0.0029	0.0004
Chi2:		2.2887			4.6255	
C Chi2:		1.0713			2.2699	

Bailey for 6 × 750:

Teta:	0.3804	0.0199	0.0000
Chi2:	7.1076		
C chi2:	3.3412		

chi^2(ctrl + sel) - chi^2(ctrl) - chi^2(sel): 0.1934
C chi^2(ctrl + sel) - chi^2(ctrl) - chi^2(sel): **0.0000**

**ML test Ctrl Vs. Selection**						
ML est [r1,r2,c]	0.3791	0.0209	0.0560	0.3822	0.0195	0.1189
ML SE [r1,r2,c]	0.0102	0.0030	0.0554	0.0102	0.0029	0.0820

ML test Ctrl vs. Selection (r1 and r2 are line-specific)

c - control or selection specific						
Line1[r1,r2,c]:	0.3908	0.0187	0.0562	0.3928	0.0240	0.1188
Line2[r1,r2,c]:	0.3802	0.0227	0.0562	0.3669	0.0174	0.1188
Line3[r1,r2,c]:	0.3663	0.0213	0.0562	0.3869	0.0173	0.1188
c-global						
Line1[r1,r2,c]:	0.3909	0.0187	0.0863	0.3926	0.0240	0.0863
Line2[r1,r2,c]:	0.3803	0.0227	0.0863	0.3669	0.0173	0.0863
Line3[r1,r2,c]:	0.3664	0.0213	0.0863	0.3869	0.0173	0.0863

ML ratio (df=14 – 13=1): 0.4122

***ML test Ctrl vs. Selection (r1, r2 and c are all line-specific)**

Line1[r1,r2,c]:	0.3907	0.0187	0.0000	0.3933	0.0240	0.2822
Line2[r1,r2,c]:	0.3801	0.0227	0.0000	0.3667	0.0173	0.0000
Line3[r1,r2,c]:	0.3667	0.0213	0.1702	0.3867	0.0173	0.0000

ML ratio (df=18 – 13=5): 6.2963

ML ratio for heterogeneity (control+selection; df = 5–1=4): 6.2963 – 0.4122

……………………………………………………………………………………………….

2R chromosome

markers 1-2, 3-4 (*cn-kn*, *c-px*)

	CONTROL			Desiccation		
**Lines (ML estimates)**						
Line1 [r1,r2,c]:	0.1347	0.2373	0.5423	0.2133	0.3227	0.9298
Line2 [r1,r2,c]:	0.1053	0.2453	0.2580	0.1760	0.2627	0.9518
Line3 [r1,r2,c]:	0.1267	0.2413	0.3926	0.1840	0.2867	0.9606
Bailey test for 3 × 750						
Teta [r1,r2,c]:	0.1211	0.2411	0.3784	0.1902	0.2896	0.9433
SE [r1,r2,c]:	0.0069	0.0090	0.0696	0.0083	0.0095	0.0663
Chi2:		5.9866			10.4868	
C Chi2:		2.7068			0.0432	

Bailey for 6 × 750:
Teta: 0.1484 0.2623 0.6789
Chi2: 107.0146
C chi2: 37.5126

chi^2(ctrl + sel) - chi^2(ctrl) - chi^2(sel): 90.5413
C chi^2(ctrl + sel) - chi^2(ctrl) - chi^2(sel): **34.7625**

**ML test Ctrl Vs. Selection**						
ML est [r1,r2,c]	0.1222	0.2413	0.4068	0.1911	0.2907	0.9521
ML SE [r1,r2,c]	0.0069	0.0090	0.0722	0.0083	0.0096	0.0670

ML test Ctrl vs. Selection (r1 and r2 are line-specific)

c - control or selection specific						
Line1[r1,r2,c]:	0.1343	0.2366	0.4078	0.2134	0.3227	0.9449
Line2[r1,r2,c]:	0.1056	0.2460	0.4078	0.1760	0.2627	0.9449
Line3[r1,r2,c]:	0.1267	0.2414	0.4078	0.1840	0.2866	0.9449
c-global						
Line1[r1,r2,c]:	0.1350	0.2379	0.7742	0.2125	0.3214	0.7742
Line2[r1,r2,c]:	0.1058	0.2463	0.7742	0.1755	0.2620	0.7742
Line3[r1,r2,c]:	0.1271	0.2422	0.7742	0.1834	0.2857	0.7742

ML ratio (df=14 – 13=1): 25.1032

***ML test Ctrl vs. Selection (r1, r2 and c are all line-specific)**

Line1[r1,r2,c]:	0.1347	0.2373	0.5423	0.2133	0.3227	0.9298
Line2[r1,r2,c]:	0.1053	0.2453	0.2580	0.1760	0.2627	0.9518
Line3[r1,r2,c]:	0.1267	0.2413	0.3926	0.1840	0.2867	0.9606

ML ratio (df=18 – 13=5): 27.7275

ML ratio for heterogeneity (control+selection; df = 5–1=4): 27.7275 – 25.1032

…………………………………………………………………………………………………..

markers 1-2, 3-5 (*cn-kn*, *c-sp*)

	CONTROL			DESICCATION		
**Lines (ML estimates)**						
Line1 [r1,r2,c]:	0.1347	0.3080	0.4179	0.2133	0.3733	0.8538
Line2 [r1,r2,c]:	0.1053	0.3040	0.3331	0.1760	0.3280	0.8546
Line3 [r1,r2,c]:	0.1267	0.2907	0.3984	0.1840	0.3427	0.8247
Bailey test for 3 × 750						
Teta [r1,r2,c]:	0.1211	0.3009	0.3834	0.1902	0.3476	0.8435
SE [r1,r2,c]:	0.0069	0.0097	0.0621	0.0083	0.0100	0.0570
Chi2:		4.3066			7.6007	
C Chi2:		0.3411			0.0577	

Bailey for 6 × 750:

Teta:	0.1484	0.3220	0.6339
Chi2:	95.2816		
C chi2:	30.3314		

chi^2(ctrl + sel) - chi^2(ctrl) - chi^2(sel): 83.3743
C chi^2(ctrl + sel) - chi^2(ctrl) - chi^2(sel): **29.9326**

ML test Ctrl Vs. Selection

ML est [r1,r2,c]	0.1222	0.3009	0.3867	0.1911	0.3480	0.8487
ML SE [r1,r2,c]	0.0069	0.0097	0.0624	0.0083	0.0100	0.0574

ML test Ctrl vs. Selection (r1 and r2 are line-specific)

c - control or selection specific

Line1[r1,r2,c]:	0.1345	0.3077	0.3870	0.2133	0.3733	0.8450
Line2[r1,r2,c]:	0.1055	0.3044	0.3870	0.1760	0.3280	0.8450
Line3[r1,r2,c]:	0.1266	0.2906	0.3870	0.1841	0.3428	0.8450

c-global

Line1[r1,r2,c]:	0.1353	0.3095	0.6935	0.2120	0.3710	0.6935
Line2[r1,r2,c]:	0.1058	0.3055	0.6935	0.1752	0.3266	0.6935
Line3[r1,r2,c]:	0.1273	0.2920	0.6935	0.1833	0.3413	0.6935

ML ratio (df=14 – 13=1): 25.1859

<u>*ML test Ctrl vs. Selection (r1, r2 and c are all line-specific)</u>

Line1[r1,r2,c]:	0.1347	0.3080	0.4179	0.2133	0.3733	0.8538
Line2[r1,r2,c]:	0.1053	0.3040	0.3331	0.1760	0.3280	0.8546
Line3[r1,r2,c]:	0.1267	0.2907	0.3984	0.1840	0.3427	0.8247

ML ratio (df=18 – 13=5): 25.5683

ML ratio for heterogeneity (control+selection; df = 5–1=4): 25.5683 – 25.1859

…………………………………………………………………………………………………..

<u>markers 1-2, 4-5 (*cn-kn*, *px-sp*)</u>

	CONTROL			DESICCATION		
**<u>Lines (ML estimates)</u>**						
Line1 [r1,r2,c]:	0.1347	0.0707	0.0000	0.2133	0.0560	0.5578
Line2 [r1,r2,c]:	0.1053	0.0587	0.6491	0.1760	0.0653	0.4641
Line3 [r1,r2,c]:	0.1267	0.0547	0.3855	0.1840	0.0560	0.1294
Bailey test for 3 × 750						
Teta [r1,r2,c]:	0.1212	0.0609	0.0000	0.1900	0.0587	0.2782
SE [r1,r2,c]:	0.0069	0.0050	0.0003	0.0083	0.0050	0.0993
Chi2:		10.9302			7.7607	
C Chi2:		5.5163			3.5672	

Bailey for 6 × 750:

Teta:	0.1492	0.0600	0.0000
Chi2:	67.0398		
C chi2:	16.9403		

chi^2(ctrl + sel) - chi^2(ctrl) - chi^2(sel): 48.3488
C chi^2(ctrl + sel) - chi^2(ctrl) - chi^2(sel): **7.8568**

<u>ML test Ctrl Vs. Selection</u>

ML est [r1,r2,c]	0.1222	0.0613	0.2965	0.1911	0.0591	0.3934
ML SE [r1,r2,c]	0.0069	0.0051	0.1289	0.0083	0.0050	0.1181

<u>ML test Ctrl vs. Selection (r1 and r2 are line-specific)</u>

c - control or selection specific

Line1[r1,r2,c]:	0.1349	0.0708	0.2932	0.2131	0.0559	0.3958
Line2[r1,r2,c]:	0.1052	0.0586	0.2932	0.1759	0.0653	0.3958

Line3[r1,r2,c]:	0.1266	0.0546	0.2932	0.1843	0.0561	0.3958

c-global

Line1[r1,r2,c]:	0.1350	0.0708	0.3550	0.2130	0.0559	0.3550
Line2[r1,r2,c]:	0.1052	0.0586	0.3550	0.1758	0.0653	0.3550
Line3[r1,r2,c]:	0.1266	0.0547	0.3550	0.1843	0.0561	0.3550

ML ratio (df=14 – 13=1): 0.3344

<u>*ML test Ctrl vs. Selection (r1, r2 and c are all line-specific)</u>

Line1[r1,r2,c]:	0.1347	0.0707	0.0000	0.2133	0.0560	0.5578
Line2[r1,r2,c]:	0.1053	0.0587	0.6491	0.1760	0.0653	0.4641
Line3[r1,r2,c]:	0.1267	0.0547	0.3855	0.1840	0.0560	0.1294

ML ratio (df=18 – 13=5): 9.1050

ML ratio for heterogeneity (control+selection; df = 5–1=4): 9.1050 – 0.3344

……………………………………………………………………………………………………..

<u>markers 1-3, 4-5 (*cn-c*, *px-sp*)</u>

	CONTROL			DESICCATION		
**<u>Lines (ML estimates)</u>**						
Line1 [r1,r2,c]:	0.1680	0.0707	0.0000	0.2280	0.0560	0.5224
Line2 [r1,r2,c]:	0.1200	0.0586	0.5676	0.1973	0.0653	0.4139
Line3 [r1,r2,c]:	0.1467	0.0547	0.3330	0.1987	0.0560	0.1199
Bailey test for 3 × 750						
Teta [r1,r2,c]:	0.1428	0.0611	0.0000	0.2072	0.0587	0.2569
SE [r1,r2,c]:	0.0074	0.0050	0.0007	0.0085	0.0050	0.0914
Chi2:		15.0687			6.7459	
C Chi2:		5.4806			3.5146	

Bailey for 6 × 750:

Teta:	0.1700	0.0600	0.0000
Chi2:	61.8988		
C chi2:	16.8859		

chi^2(ctrl + sel) - chi^2(ctrl) - chi^2(sel): 40.0843
C chi^2(ctrl + sel) - chi^2(ctrl) - chi^2(sel): **7.8906**

<u>ML test Ctrl Vs. Selection</u>

ML est [r1,r2,c]	0.1449	0.0613	0.2501	0.2080	0.0591	0.3615
ML SE [r1,r2,c]	0.0074	0.0051	0.1089	0.0086	0.0050	0.1086

<u>ML test Ctrl vs. Selection (r1 and r2 are line-specific)</u>

c - control or selection specific

Line1[r1,r2,c]:	0.1684	0.0708	0.2459	0.2277	0.0559	0.3632
Line2[r1,r2,c]:	0.1198	0.0586	0.2459	0.1972	0.0653	0.3632
Line3[r1,r2,c]:	0.1466	0.0546	0.2459	0.1990	0.0561	0.3632

c-global

Line1[r1,r2,c]:	0.1685	0.0709	0.3141	0.2275	0.0559	0.3141
Line2[r1,r2,c]:	0.1198	0.0586	0.3141	0.1971	0.0653	0.3141
Line3[r1,r2,c]:	0.1466	0.0547	0.3141	0.1990	0.0561	0.3141

ML ratio (df=14 – 13=1): 0.5679

<u>*ML test Ctrl vs. Selection (r1, r2 and c are all line-specific)</u>

Line1[r1,r2,c]:	0.1680	0.0707	0.0000	0.2280	0.0560	0.5224
Line2[r1,r2,c]:	0.1200	0.0586	0.5676	0.1973	0.0653	0.4139
Line3[r1,r2,c]:	0.1467	0.0547	0.3330	0.1987	0.0560	0.1199

ML ratio (df=18 – 13=5): 9.7068

ML ratio for heterogeneity (control+selection; df = 5–1=4): 9.7068 –0.5679

…………………………………………………………………………………………………..

3rd chromosome

markers 1-2, 3-4 (*ru-h*, *th-cu*)

	CONTROL			DESICCATION		
**Lines (ML estimates)**						
Line1 [r1,r2,c]:	0.1974	0.0560	0.0000	0.2027	0.0480	0.4111
Line2 [r1,r2,c]:	0.1946	0.0560	0.2447	0.2146	0.0573	0.9757
Line3 [r1,r2,c]:	0.1733	0.0573	0.1342	0.2053	0.0427	0.6096
Bailey test for 3 × 750						
Teta [r1,r2,c]:	0.1878	0.0564	0.0000	0.2074	0.0486	0.6266
SE [r1,r2,c]:	0.0082	0.0049	0.0002	0.0085	0.0045	0.1488
Chi2:		4.9273			4.5331	
C Chi2:		3.1684			2.4683	

Bailey for 6 × 750:

Teta: 0.1972 0.0520 0.0000

Chi2: 30.9609

C chi2: 23.2985

chi^2(ctrl + sel) - chi^2(ctrl) - chi^2(sel): 21.5005

C chi^2(ctrl + sel) - chi^2(ctrl) - chi^2(sel): **17.6617**

ML test Ctrl Vs. Selection

ML est [r1,r2,c]	0.1884	0.0564	0.1253	0.2075	0.0493	0.6944
ML SE [r1,r2,c]	0.0082	0.0049	0.0712	0.0085	0.0046	0.1577

ML test Ctrl vs. Selection (r1 and r2 are line-specific)

c - control or selection specific

Line1[r1,r2,c]:	0.1976	0.0561	0.1256	0.2029	0.0480	0.6955
Line2[r1,r2,c]:	0.1944	0.0559	0.1256	0.2144	0.0573	0.6955
Line3[r1,r2,c]:	0.1733	0.0573	0.1256	0.2054	0.0427	0.6955

c-global

Line1[r1,r2,c]:	0.1979	0.0562	0.4056	0.2027	0.0480	0.4056
Line2[r1,r2,c]:	0.1949	0.0561	0.4056	0.2137	0.0571	0.4056
Line3[r1,r2,c]:	0.1736	0.0574	0.4056	0.2051	0.0426	0.4056

ML ratio (df=14 – 13=1): 11.3436

***ML test Ctrl vs. Selection (r1, r2 and c are all line-specific)**

Line1[r1,r2,c]:	0.1974	0.0560	0.0000	0.2027	0.0480	0.4111
Line2[r1,r2,c]:	0.1946	0.0560	0.2447	0.2146	0.0573	0.9757
Line3[r1,r2,c]:	0.1733	0.0573	0.1342	0.2053	0.0427	0.6096

ML ratio (df=18 – 13=5): 16.6349

ML ratio for heterogeneity (control+selection; df = 5–1=4): 16.6349 –11.3436

……………………………………………………………………………………………..

markers 1-2, 3-5 (*ru-h*, *th-sr*)

	CONTROL			DESICCATION		
**Lines (ML estimates)**						
Line1 [r1,r2,c]:	0.1973	0.1320	0.2559	0.2027	0.1213	0.5964
Line2 [r1,r2,c]:	0.1947	0.1427	0.4321	0.2147	0.1667	1.2298
Line3 [r1,r2,c]:	0.1733	0.1360	0.3959	0.2053	0.1293	0.9037
Bailey test for 3 × 750						
Teta [r1,r2,c]:	0.1879	0.1369	0.3449	0.2069	0.1371	0.9125
SE [r1,r2,c]:	0.0082	0.0072	0.0723	0.0085	0.0072	0.0973
Chi2:		3.3710			14.8568	
C Chi2:		1.2269			7.5196	

Bailey for 6 × 750:
Teta: 0.1974 0.1368 0.5485
Chi2: 42.5312

C chi2: 30.1410

chi^2(ctrl + sel) - chi^2(ctrl) - chi^2(sel): 24.3034
C chi^2(ctrl + sel) - chi^2(ctrl) - chi^2(sel): **21.3945**

ML test Ctrl Vs. Selection

ML est [r1,r2,c]	0.1884	0.1369	0.3618	0.2076	0.1391	0.9543
ML SE [r1,r2,c]	0.0082	0.0072	0.0741	0.0085	0.0073	0.1010

ML test Ctrl vs. Selection (r1 and r2 are line-specific)

c - control or selection specific

Line1[r1,r2,c]:	0.1977	0.1323	0.3618	0.2028	0.1214	0.9583
Line2[r1,r2,c]:	0.1944	0.1425	0.3618	0.2145	0.1666	0.9583
Line3[r1,r2,c]:	0.1732	0.1359	0.3618	0.2053	0.1293	0.9583

c-global

Line1[r1,r2,c]:	0.1982	0.1326	0.6787	0.2028	0.1214	0.6787
Line2[r1,r2,c]:	0.1952	0.1431	0.6787	0.2129	0.1653	0.6787
Line3[r1,r2,c]:	0.1738	0.1364	0.6787	0.2048	0.1290	0.6787

ML ratio (df=14 – 13=1): 21.5564

***ML test Ctrl vs. Selection (r1, r2 and c are all line-specific)**

Line1[r1,r2,c]:	0.1973	0.1320	0.2559	0.2027	0.1213	0.5964
Line2[r1,r2,c]:	0.1947	0.1427	0.4321	0.2147	0.1667	1.2298
Line3[r1,r2,c]:	0.1733	0.1360	0.3959	0.2053	0.1293	0.9037

ML ratio (df=18 – 13=5): 29.6394

ML ratio for heterogeneity (control+selection; df = 5–1=4): 29.6394 – 21.5564

…………………………………………………………………………………………………..

markers 1-2, 3-6 (*ru-h*, *th-e*)

	CONTROL			DESICCATION		
**Lines (ML estimates)**						
Line1 [r1,r2,c]:	0.1973	0.1867	0.2172	0.2027	0.1720	0.5355
Line2 [r1,r2,c]:	0.1947	0.1867	0.4770	0.2147	0.2213	1.0383
Line3 [r1,r2,c]:	0.1733	0.1813	0.2969	0.2053	0.1947	0.8006
Bailey test for 3 × 750						
Teta [r1,r2,c]:	0.1879	0.1849	0.3014	0.2069	0.1945	0.7893
SE [r1,r2,c]:	0.0082	0.0082	0.0579	0.0085	0.0083	0.0763
Chi2:		5.1729			13.5380	
C Chi2:		3.1569			7.4889	

Bailey for 6 × 750:

Teta:	0.1969 0.1893 0.4809
Chi2:	47.5727
C chi2:	36.1781

chi^2(ctrl + sel) - chi^2(ctrl) - chi^2(sel): 28.8618
C chi^2(ctrl + sel) - chi^2(ctrl) - chi^2(sel): **25.5322**

ML test Ctrl Vs. Selection

ML est [r1,r2,c]	0.1884 0.1849 0.3317	0.2076 0.1960 0.8194
ML SE [r1,r2,c]	0.0082 0.0082 0.0608	0.0085 0.0084 0.0786

ML test Ctrl vs. Selection (r1 and r2 are line-specific)

c - control or selection specific

Line1[r1,r2,c]:	0.1980	0.1873	0.3314	0.2032	0.1724	0.8226
Line2[r1,r2,c]:	0.1939	0.1859	0.3314	0.2141	0.2207	0.8226
Line3[r1,r2,c]:	0.1735	0.1815	0.3314	0.2054	0.1947	0.8226

c-global

Line1[r1,r2,c]:	0.1988	0.1880	0.5981	0.2029	0.1722	0.5981
Line2[r1,r2,c]:	0.1951	0.1871	0.5981	0.2123	0.2189	0.5981
Line3[r1,r2,c]:	0.1742	0.1822	0.5981	0.2045	0.1938	0.5981

ML ratio (df=14 – 13=1): 23.0247

***ML test Ctrl vs. Selection (r1, r2 and c are all line-specific)**

Line1[r1,r2,c]:	0.1973	0.1867	0.2172	0.2027	0.1720	0.5355
Line2[r1,r2,c]:	0.1947	0.1867	0.4770	0.2147	0.2213	1.0383
Line3[r1,r2,c]:	0.1733	0.1813	0.2969	0.2053	0.1947	0.8006

ML ratio (df=18 – 13=5): 33.2755

ML ratio for heterogeneity (control+selection; df = 5–1=4): 33.2755 – 23.0247

………………………………………………………………………………………………………

markers 1-2, 4-5 (*ru-h*, *cu-sr*)

	CONTROL	DESICCATION
**Lines (ML estimates)**		
Line1 [r1,r2,c]:	0.1973 0.0760 0.4447	0.2027 0.0787 0.8367
Line2 [r1,r2,c]:	0.1947 0.0867 0.5534	0.2147 0.1173 1.3764
Line3 [r1,r2,c]:	0.1734 0.0840 0.5494	0.2053 0.0867 1.0494
Bailey test for 3 × 750		
Teta [r1,r2,c]:	0.1879 0.0820 0.5121	0.2069 0.0919 1.1168
SE [r1,r2,c]:	0.0082 0.0058 0.1125	0.0085 0.0061 0.1301

Chi2:	2.5397	10.2618
C Chi2:	0.2094	3.0673

Bailey for 6 × 750:

Teta:	0.1972 0.0866 0.7745
Chi2:	29.1481
C chi2:	15.3082

chi^2(ctrl + sel) - chi^2(ctrl) - chi^2(sel): 16.3466
C chi^2(ctrl + sel) - chi^2(ctrl) - chi^2(sel): **12.0314**

ML test Ctrl Vs. Selection

ML est [r1,r2,c]	0.1884 0.0822 0.5164	0.2075 0.0942 1.1362
ML SE [r1,r2,c]	0.0082 0.0058 0.1129	0.0085 0.0062 0.1330

ML test Ctrl vs. Selection (r1 and r2 are line-specific)

c - control or selection specific

Line1[r1,r2,c]:	0.1974	0.0760	0.5169	0.2025	0.0786	1.1380
Line2[r1,r2,c]:	0.1946	0.0866	0.5169	0.2149	0.1175	1.1380
Line3[r1,r2,c]:	0.1733	0.0840	0.5169	0.2053	0.0866	1.1380

c-global

Line1[r1,r2,c]:	0.1975	0.0761	0.8688	0.2027	0.0787	0.8688
Line2[r1,r2,c]:	0.1948	0.0867	0.8688	0.2142	0.1171	0.8688
Line3[r1,r2,c]:	0.1735	0.0841	0.8688	0.2052	0.0866	0.8688

ML ratio (df=14 – 13=1): 11.8343

***ML test Ctrl vs. Selection (r1, r2 and c are all line-specific)**

Line1[r1,r2,c]:	0.1973	0.0760	0.4447	0.2027	0.0787	0.8367
Line2[r1,r2,c]:	0.1947	0.0867	0.5534	0.2147	0.1173	1.3764
Line3[r1,r2,c]:	0.1734	0.0840	0.5494	0.2053	0.0867	1.0494

ML ratio (df=18 – 13=5): 15.0020

ML ratio for heterogeneity (control+selection; df = 5–1=4): 15.0020 –11.8343

…………………………………………………………………………………………………..

markers 1-2, 4-6 (*ru-h*, *cu-e*)

Lines (ML estimates)

	**CONTROL**	**DESICCATION**
Line1 [r1,r2,c]:	0.1973 0.1307 0.3103	0.2027 0.1293 0.6613
Line2 [r1,r2,c]:	0.1947 0.1307 0.5766	0.2147 0.1747 1.1379
Line3 [r1,r2,c]:	0.1733 0.1320 0.3497	0.2053 0.1520 0.8544

Bailey test for 3 × 750

Teta [r1,r2,c]:	0.1879	0.1311	0.3886	0.2070	0.1502	0.8944
SE [r1,r2,c]:	0.0082	0.0071	0.0778	0.0085	0.0075	0.0926
Chi2:		3.6854			10.9288	
C Chi2:		1.9488			4.5169	

Bailey for 6 × 750:

Teta:	0.1969	0.1399	0.6002
Chi2:	38.0364		
C chi2:	23.6658		

chi^2(ctrl + sel) - chi^2(ctrl) - chi^2(sel): 23.4221
C chi^2(ctrl + sel) - chi^2(ctrl) - chi^2(sel): **17.2000**

ML test Ctrl Vs. Selection

ML est [r1,r2,c]	0.1884	0.1311	0.4137	0.2076	0.1520	0.9157
ML SE [r1,r2,c]	0.0082	0.0071	0.0803	0.0085	0.0076	0.0947

ML test Ctrl vs. Selection (r1 and r2 are line-specific)

c - control or selection specific

Line1[r1,r2,c]:	0.1977	0.1309	0.4139	0.2028	0.1294	0.9180
Line2[r1,r2,c]:	0.1941	0.1303	0.4139	0.2144	0.1745	0.9180
Line3[r1,r2,c]:	0.1735	0.1321	0.4139	0.2054	0.1520	0.9180

c-global

Line1[r1,r2,c]:	0.1981	0.1312	0.7009	0.2027	0.1294	0.7009
Line2[r1,r2,c]:	0.1949	0.1308	0.7009	0.2132	0.1735	0.7009
Line3[r1,r2,c]:	0.1739	0.1324	0.7009	0.2049	0.1517	0.7009

ML ratio (df=14 – 13=1): 15.3538

***ML test Ctrl vs. Selection (r1, r2 and c are all line-specific)**

Line1[r1,r2,c]:	0.1973	0.1307	0.3103	0.2027	0.1293	0.6613
Line2[r1,r2,c]:	0.1947	0.1307	0.5766	0.2147	0.1747	1.1379
Line3[r1,r2,c]:	0.1733	0.1320	0.3497	0.2053	0.1520	0.8544

ML ratio (df=18 – 13=5): 21.8502

ML ratio for heterogeneity (control+selection; df = 5–1=4): 21.8502 –15.3538

…………………………………………………………………………………………………..

markers 1-2, 5-6 (*ru-h*, *sr-e*)

CONTROL **DESICCATION**

Lines (ML estimates)

Line1 [r1,r2,c]:	0.1973 0.0547 0.1236	0.2027 0.0533 0.3702
Line2 [r1,r2,c]:	0.1947 0.0440 0.6225	0.2147 0.0600 0.6216
Line3 [r1,r2,c]:	0.1733 0.0507 0.0000	0.2053 0.0680 0.5732

Bailey test for 3 × 750

Teta [r1,r2,c]:	0.1880 0.0493 0.0000	0.2074 0.0599 0.5110
SE [r1,r2,c]:	0.0082 0.0046 0.0006	0.0085 0.0050 0.1245
Chi2:	8.4225	2.5611
C Chi2:	5.7241	0.7934

Bailey for 6 × 750:
Teta: 0.1969 0.0539 0.0000
Chi2: 33.0496
C chi2: 23.3827

chi^2(ctrl + sel) - chi^2(ctrl) - chi^2(sel): 22.0661
C chi^2(ctrl + sel) - chi^2(ctrl) - chi^2(sel): **16.8653**

ML test Ctrl Vs. Selection

ML est [r1,r2,c]	0.1884 0.0498 0.2369	0.2076 0.0604 0.5313
ML SE [r1,r2,c]	0.0082 0.0046 0.1029	0.0085 0.0050 0.1271

ML test Ctrl vs. Selection (r1 and r2 are line-specific)

c - control or selection specific

Line1[r1,r2,c]:	0.1975	0.0547	0.2370	0.2029	0.0534	0.5317
Line2[r1,r2,c]:	0.1941	0.0439	0.2370	0.2145	0.0600	0.5317
Line3[r1,r2,c]:	0.1736	0.0507	0.2370	0.2053	0.0680	0.5317

c-global

Line1[r1,r2,c]:	0.1977	0.0548	0.4067	0.2027	0.0533	0.4067
Line2[r1,r2,c]:	0.1944	0.0439	0.4067	0.2143	0.0599	0.4067
Line3[r1,r2,c]:	0.1737	0.0508	0.4067	0.2050	0.0679	0.4067

ML ratio (df=14 – 13=1): 3.0421

***ML test Ctrl vs. Selection (r1, r2 and c are all line-specific)**

Line1[r1,r2,c]:	0.1973	0.0547	0.1236	0.2027	0.0533	0.3702
Line2[r1,r2,c]:	0.1947	0.0440	0.6225	0.2147	0.0600	0.6216
Line3[r1,r2,c]:	0.1733	0.0507	0.0000	0.2053	0.0680	0.5732

ML ratio (df=18 – 13=5): 10.5478

ML ratio for heterogeneity (control+selection; df = 5–1=4): 10.5478 –3.0421

……………………………………………………………………………………………………..

markers 1-3, 4-5 (*ru-th, cu-sr*)

	CONTROL			DESICCATION		
**Lines (ML estimates)**						
Line1 [r1,r2,c]:	0.2880	0.0760	0.3046	0.3253	0.0787	0.6773
Line2 [r1,r2,c]:	0.2893	0.0867	0.4254	0.3093	0.1173	0.9551
Line3 [r1,r2,c]:	0.2893	0.0840	0.3292	0.3333	0.0867	0.5077
Bailey test for 3 × 750						
Teta [r1,r2,c]:	0.2889	0.0820	0.3493	0.3222	0.0922	0.7019
SE [r1,r2,c]:	0.0096	0.0058	0.0752	0.0098	0.0061	0.0849
Chi2:		1.0873			12.7065	
C Chi2:		0.4534			5.0034	

Bailey for 6 × 750:

Teta:	0.3048	0.0865	0.5054
Chi2:	30.7741		
C chi2:	15.1485		

chi^2(ctrl + sel) - chi^2(ctrl) - chi^2(sel): 16.9803

C chi^2(ctrl + sel) - chi^2(ctrl) - chi^2(sel): **9.6918**

**ML test Ctrl Vs. Selection**						
ML est [r1,r2,c]	0.2889	0.0822	0.3555	0.3227	0.0942	0.7309
ML SE [r1,r2,c]	0.0096	0.0058	0.0759	0.0099	0.0062	0.0868

ML test Ctrl vs. Selection (r1 and r2 are line-specific)

c - control or selection specific						
Line1[r1,r2,c]:	0.2882	0.0761	0.3555	0.3255	0.0787	0.7347
Line2[r1,r2,c]:	0.2890	0.0865	0.3555	0.3084	0.1170	0.7347
Line3[r1,r2,c]:	0.2895	0.0840	0.3555	0.3342	0.0869	0.7347
c-global						
Line1[r1,r2,c]:	0.2889	0.0762	0.5710	0.3248	0.0785	0.5710
Line2[r1,r2,c]:	0.2899	0.0868	0.5710	0.3069	0.1164	0.5710
Line3[r1,r2,c]:	0.2903	0.0843	0.5710	0.3337	0.0868	0.5710

ML ratio (df=14 – 13=1): 10.0934

***ML test Ctrl vs. Selection (r1, r2 and c are all line-specific)**

Line1[r1,r2,c]:	0.2880	0.0760	0.3046	0.3253	0.0787	0.6773
Line2[r1,r2,c]:	0.2893	0.0867	0.4254	0.3093	0.1173	0.9551
Line3[r1,r2,c]:	0.2893	0.0840	0.3292	0.3333	0.0867	0.5077

ML ratio (df=18 – 13=5): 15.4592

ML ratio for heterogeneity (control+selection; df = 5–1=4): 15.4592 –10.0934

……………………………………………………………………………………………………..

markers 1-3, 4-6 (*ru-th*, *cu-e*)

	CONTROL			DESICCATION		
**Lines (ML estimates)**						
Line1 [r1,r2,c]:	0.2880	0.1307	0.2126	0.3253	0.1293	0.5704
Line2 [r1,r2,c]:	0.2893	0.1307	0.3527	0.3093	0.1747	0.7650
Line3 [r1,r2,c]:	0.2893	0.1320	0.2444	0.3333	0.1520	0.6053

Bailey test for 3 × 750

Teta [r1,r2,c]:	0.2888	0.1311	0.2594	0.3223	0.1503	0.6471
SE [r1,r2,c]:	0.0096	0.0071	0.0518	0.0099	0.0075	0.0641
Chi2:		1.1906			8.5387	
C Chi2:		1.1804			1.7271	

Bailey for 6 × 750:

Teta:	0.3044	0.1396	0.4133
Chi2:	40.9745		
C chi2:	25.2206		

chi^2(ctrl + sel) - chi^2(ctrl) - chi^2(sel): 31.2452
C chi^2(ctrl + sel) - chi^2(ctrl) - chi^2(sel): **22.3131**

ML test Ctrl Vs. Selection

ML est [r1,r2,c]	0.2889	0.1311	0.2699	0.3227	0.1520	0.6525
ML SE [r1,r2,c]	0.0096	0.0071	0.0528	0.0099	0.0076	0.0643

ML test Ctrl vs. Selection (r1 and r2 are line-specific)

c - control or selection specific

Line1[r1,r2,c]:	0.2885	0.1309	0.2698	0.3259	0.1296	0.6546
Line2[r1,r2,c]:	0.2886	0.1303	0.2698	0.3084	0.1741	0.6546
Line3[r1,r2,c]:	0.2896	0.1321	0.2698	0.3337	0.1522	0.6546

c-global

Line1[r1,r2,c]:	0.2899	0.1315	0.4897	0.3246	0.1290	0.4897
Line2[r1,r2,c]:	0.2903	0.1311	0.4897	0.3063	0.1729	0.4897
Line3[r1,r2,c]:	0.2911	0.1328	0.4897	0.3320	0.1514	0.4897

ML ratio (df=14 – 13=1): 19.6098

***ML test Ctrl vs. Selection (r1, r2 and c are all line-specific)**

Line1[r1,r2,c]:	0.2880	0.1307	0.2126	0.3253	0.1293	0.5704
Line2[r1,r2,c]:	0.2893	0.1307	0.3527	0.3093	0.1747	0.7650
Line3[r1,r2,c]:	0.2893	0.1320	0.2444	0.3333	0.1520	0.6053

ML ratio (df=18 – 13=5): 22.6138

ML ratio for heterogeneity (control+selection; df = 5–1=4): 22.6138 – 19.6098

……………………………………………………………………………………………………..

markers 1-3, 5-6 (*ru-th*, *sr-e*)

	CONTROL			DESICCATION		
**Lines (ML estimates)**						
Line1 [r1,r2,c]:	0.2880	0.0547	0.0847	0.3253	0.0533	0.5381
Line2 [r1,r2,c]:	0.2893	0.0440	0.2094	0.3093	0.0600	0.5028
Line3 [r1,r2,c]:	0.2893	0.0507	0.0910	0.3333	0.0680	0.7059
Bailey test for 3 × 750						
Teta [r1,r2,c]:	0.2890	0.0494	0.1063	0.3225	0.0598	0.5831
SE [r1,r2,c]:	0.0096	0.0046	0.0561	0.0099	0.0050	0.1012
Chi2:		1.5878			3.2142	
C Chi2:		0.6193			0.7837	

Bailey for 6 × 750:
Teta: 0.3046 0.0538 0.2187
Chi2: 30.1786
C chi2: 18.3874

chi^2(ctrl + sel) - chi^2(ctrl) - chi^2(sel): 25.3767
C chi^2(ctrl + sel) - chi^2(ctrl) - chi^2(sel): **16.9844**

**ML test Ctrl Vs. Selection**						
ML est [r1,r2,c]	0.2889	0.0498	0.1236	0.3227	0.0604	0.5924
ML SE [r1,r2,c]	0.0096	0.0046	0.0605	0.0099	0.0050	0.1023

ML test Ctrl vs. Selection (r1 and r2 are line-specific)

c - control or selection specific

Line1[r1,r2,c]:	0.2882	0.0547	0.1236	0.3255	0.0534	0.5928
Line2[r1,r2,c]:	0.2890	0.0440	0.1236	0.3096	0.0600	0.5928
Line3[r1,r2,c]:	0.2895	0.0507	0.1236	0.3329	0.0679	0.5928

c-global

Line1[r1,r2,c]:	0.2890	0.0549	0.3959	0.3248	0.0532	0.3959
Line2[r1,r2,c]:	0.2898	0.0441	0.3959	0.3089	0.0599	0.3959
Line3[r1,r2,c]:	0.2902	0.0508	0.3959	0.3317	0.0677	0.3959

ML ratio (df=14 – 13=1): 13.7506

***ML test Ctrl vs. Selection (r1, r2 and c are all line-specific)**

Line1[r1,r2,c]:	0.2880	0.0547	0.0847	0.3253	0.0533	0.5381
Line2[r1,r2,c]:	0.2893	0.0440	0.2094	0.3093	0.0600	0.5028
Line3[r1,r2,c]:	0.2893	0.0507	0.0910	0.3333	0.0680	0.7059

ML ratio (df=18 – 13=5): 15.3237

ML ratio for heterogeneity (control+selection; df = 5–1=4): 15.3237 –13.7506

…………………………………………………………………………………………………..

markers 1-4, 5-6 (*ru-cu*, *sr-e*)

	CONTROL			DESICCATION		
**Lines (ML estimates)**						
Line1 [r1,r2,c]:	0.3440	0.0547	0.0709	0.3573	0.0533	0.4898
Line2 [r1,r2,c]:	0.3400	0.0440	0.1783	0.3373	0.0600	0.3953
Line3 [r1,r2,c]:	0.3442	0.0507	0.0000	0.3627	0.0680	0.6488
Bailey test for 3 × 750						
Teta [r1,r2,c]:	0.3425	0.0494	0.0000	0.3522	0.0598	0.5058
SE [r1,r2,c]:	0.0100	0.0046	0.0001	0.0101	0.0050	0.0907
Chi2:		4.2627			3.8990	
C Chi2:		3.1846			1.3725	

Bailey for 6 × 750:

Teta:	0.3459	0.0538	0.0000
Chi2:	41.9518		
C chi2:	35.6874		

chi^2(ctrl + sel) - chi^2(ctrl) - chi^2(sel): 33.7901
C chi^2(ctrl + sel) - chi^2(ctrl) - chi^2(sel): **31.1303**

ML test Ctrl Vs. Selection

ML est [r1,r2,c]	0.3427	0.0498	0.0782	0.3524	0.0605	0.5216
ML SE [r1,r2,c]	0.0100	0.0046	0.0444	0.0101	0.0050	0.0924

ML test Ctrl vs. Selection (r1 and r2 are line-specific)

c - control or selection specific						
Line1[r1,r2,c]:	0.3440	0.0547	0.0781	0.3575	0.0533	0.5222
Line2[r1,r2,c]:	0.3395	0.0439	0.0781	0.3379	0.0601	0.5222
Line3[r1,r2,c]:	0.3444	0.0507	0.0781	0.3620	0.0679	0.5222
c-global						
Line1[r1,r2,c]:	0.3453	0.0549	0.3254	0.3565	0.0532	0.3254
Line2[r1,r2,c]:	0.3406	0.0441	0.3254	0.3370	0.0599	0.3254
Line3[r1,r2,c]:	0.3455	0.0509	0.3254	0.3604	0.0676	0.3254

ML ratio (df=14 – 13=1): 17.2147

***ML test Ctrl vs. Selection (r1, r2 and c are all line-specific)**

Line1[r1,r2,c]:	0.3440	0.0547	0.0709	0.3573	0.0533	0.4898
Line2[r1,r2,c]:	0.3400	0.0440	0.1783	0.3373	0.0600	0.3953
Line3[r1,r2,c]:	0.3442	0.0507	0.0000	0.3627	0.0680	0.6488

ML ratio (df=18 – 13=5): 21.7628

ML ratio for heterogeneity (control+selection; df = 5–1=4): 21.7628 –17.2147

…………………………………………………………………………………………………..

markers 2-3, 4-5 (*h-th*, *cu-sr*)

	CONTROL			DESICCATION		
**Lines (ML estimates)**						
Line1 [r1,r2,c]:	0.1307	0.0760	0.2687	0.2267	0.0787	1.7203
Line2 [r1,r2,c]:	0.1427	0.0867	0.3237	0.1907	0.1173	1.7883
Line3 [r1,r2,c]:	0.1613	0.0840	0.7876	0.2187	0.0867	1.1964
Bailey test for 3 × 750						
Teta [r1,r2,c]:	0.1440	0.0818	0.3996	0.2110	0.0922	1.5582
SE [r1,r2,c]:	0.0074	0.0058	0.1145	0.0086	0.0061	0.1428
Chi2:		6.6097			15.1247	
C Chi2:		3.0733			3.6229	

Bailey for 6 × 750:
Teta: 0.1735 0.0855 0.8782
Chi2: 101.0585
C chi2: 46.8078

chi^2(ctrl + sel) - chi^2(ctrl) - chi^2(sel): 79.3241
C chi^2(ctrl + sel) - chi^2(ctrl) - chi^2(sel): **40.1115**

ML test Ctrl Vs. Selection

ML est [r1,r2,c]	0.1449	0.0822	0.4851	0.2120	0.0942	1.5578
ML SE [r1,r2,c]	0.0074	0.0058	0.1269	0.0086	0.0062	0.1429

ML test Ctrl vs. Selection (r1 and r2 are line-specific)

c - control or selection specific						
Line1[r1,r2,c]:	0.1308	0.0761	0.4853	0.2272	0.0789	1.5779
Line2[r1,r2,c]:	0.1428	0.0868	0.4853	0.1915	0.1179	1.5779
Line3[r1,r2,c]:	0.1609	0.0838	0.4853	0.2171	0.0860	1.5779
c-global						
Line1[r1,r2,c]:	0.1304	0.0758	1.1953	0.2273	0.0789	1.1953
Line2[r1,r2,c]:	0.1423	0.0864	1.1953	0.1914	0.1178	1.1953
Line3[r1,r2,c]:	0.1611	0.0839	1.1953	0.2187	0.0867	1.1953

ML ratio (df=14 – 13=1): 26.0518

<u>*ML test Ctrl vs. Selection (r1, r2 and c are all line-specific)</u>

Line1[r1,r2,c]:	0.1307	0.0760	0.2687	0.2267	0.0787	1.7203
Line2[r1,r2,c]:	0.1427	0.0867	0.3237	0.1907	0.1173	1.7883
Line3[r1,r2,c]:	0.1613	0.0840	0.7876	0.2187	0.0867	1.1964

ML ratio (df=18 – 13=5): 32.9051

ML ratio for heterogeneity (control+selection; df = 5–1=4): 32.9051 – 26.0518

…………………………………………………………………………………………………..

<u>markers 2-3, 4-6 (*h-th*, *cu-e*)</u>

	CONTROL			DESICCATION		
Bailey test for 3 × 750						
Teta [r1,r2,c]:	0.1439	0.1308	0.3388	0.2112	0.1503	1.2322
SE [r1,r2,c]:	0.0074	0.0071	0.0828	0.0086	0.0075	0.1020
Chi2:		9.7696			11.2430	
C Chi2:		7.0133			1.2654	

Bailey for 6 × 750:
Teta: 0.1727 0.1387 0.7059
Chi2: 107.3593
C chi2: 54.5571

chi^2(ctrl + sel) - chi^2(ctrl) - chi^2(sel): 86.3467
C chi^2(ctrl + sel) - chi^2(ctrl) - chi^2(sel): **46.2784**

<u>ML test Ctrl Vs. Selection</u>

ML est [r1,r2,c]	0.1449	0.1311	0.4679	0.2120	0.1520	1.2275
ML SE [r1,r2,c]	0.0074	0.0071	0.0976	0.0086	0.0076	0.1017

<u>ML test Ctrl vs. Selection (r1 and r2 are line-specific)</u>

c - control or selection specific

Line1[r1,r2,c]:	0.1311	0.1311	0.4686	0.2269	0.1294	1.2373
Line2[r1,r2,c]:	0.1424	0.1304	0.4686	0.1909	0.1749	1.2373
Line3[r1,r2,c]:	0.1611	0.1318	0.4686	0.2182	0.1517	1.2373

c-global

Line1[r1,r2,c]:	0.1307	0.1307	0.9689	0.2266	0.1293	0.9689
Line2[r1,r2,c]:	0.1427	0.1307	0.9689	0.1906	0.1746	0.9689
Line3[r1,r2,c]:	0.1614	0.1320	0.9689	0.2186	0.1520	0.9689

ML ratio (df=14 – 13=1): 24.7715

<u>*ML test Ctrl vs. Selection (r1, r2 and c are all line-specific)</u>

Line1[r1,r2,c]:	0.1307	0.1307	0.1562	0.2267	0.1293	1.3190
Line2[r1,r2,c]:	0.1427	0.1307	0.6438	0.1907	0.1747	1.3212
Line3[r1,r2,c]:	0.1613	0.1320	0.5635	0.2187	0.1520	1.0831

ML ratio (df=18 – 13=5): 31.2743

ML ratio for heterogeneity (control+selection; df = 5–1=4): 31.2743 – 26.0518

……………………………………………………………………………………………………..

<u>markers 2-3, 5-6 (*h-th*, *sr-e*)</u>

	CONTROL			DESICCATION		
Bailey test for 3 × 750						
Teta [r1,r2,c]:	0.1445	0.0495	0.0000	0.2112	0.0598	0.7795
SE [r1,r2,c]:	0.0074	0.0046	0.0002	0.0086	0.0050	0.1455
Chi2:		12.4223			5.7104	
C Chi2:		8.7736			1.0352	

Bailey for 6 × 750:

Teta:	0.1725	0.0539	0.0000
Chi2:	83.8880		
C chi2:	38.4059		

chi^2(ctrl + sel) - chi^2(ctrl) - chi^2(sel): 65.7554
C chi^2(ctrl + sel) - chi^2(ctrl) - chi^2(sel): **28.5971**

<u>ML test Ctrl Vs. Selection</u>

ML est [r1,r2,c]	0.1449	0.0498	0.4313	0.2120	0.0604	0.7979
ML SE [r1,r2,c]	0.0074	0.0046	0.1560	0.0086	0.0050	0.1477

<u>ML test Ctrl vs. Selection (r1 and r2 are line-specific)</u>

c - control or selection specific

Line1[r1,r2,c]:	0.1309	0.0548	0.4326	0.2266	0.0533	0.8007
Line2[r1,r2,c]:	0.1422	0.0439	0.4326	0.1908	0.0600	0.8007
Line3[r1,r2,c]:	0.1616	0.0507	0.4326	0.2186	0.0680	0.8007

c-global

Line1[r1,r2,c]:	0.1309	0.0548	0.6723	0.2264	0.0533	0.6723
Line2[r1,r2,c]:	0.1425	0.0439	0.6723	0.1907	0.0600	0.6723
Line3[r1,r2,c]:	0.1616	0.0507	0.6723	0.2184	0.0679	0.6723

ML ratio (df=14 – 13=1): 2.6426

***ML test Ctrl vs. Selection (r1, r2 and c are all line-specific)**

Line1[r1,r2,c]:	0.1307	0.0547	0.0000	0.2267	0.0533	0.8828
Line2[r1,r2,c]:	0.1427	0.0440	1.2726	0.1907	0.0600	0.5829
Line3[r1,r2,c]:	0.1613	0.0507	0.1631	0.2186	0.0680	0.8965

ML ratio (df=18 – 13=5): 15.6184

ML ratio for heterogeneity (control+selection; df = 5–1=4): 15.6184 – 2.6426

…………………………………………………………………………………………………..

markers 2-4, 5-6 (*h-cu*, *sr-e*)

	CONTROL			DESICCATION		
**Lines (ML estimates)**						
Line1 [r1,r2,c]:	0.1867	0.0547	0.0000	0.2560	0.0533	0.7817
Line2 [r1,r2,c]:	0.1986	0.0440	0.9167	0.2373	0.0600	0.5620
Line3 [r1,r2,c]:	0.2134	0.0507	0.0000	0.2480	0.0680	0.7911
Bailey test for 3 × 750						
Teta [r1,r2,c]:	0.1995	0.0494	0.0000	0.2470	0.0598	0.7032
SE [r1,r2,c]:	0.0084	0.0046	0.0002	0.0091	0.0050	0.1280
Chi2:		10.2241			2.7998	
C Chi2:		7.6610			0.7233	

Bailey for 6 × 750:

Teta:	0.2208	0.0538	0.0000
Chi2:	60.3520		
C chi2:	38.5452		

chi^2(ctrl + sel) - chi^2(ctrl) - chi^2(sel): 47.3281
C chi^2(ctrl + sel) - chi^2(ctrl) - chi^2(sel): **30.1608**

ML test Ctrl Vs. Selection

ML est [r1,r2,c]	0.1996	0.0498	0.2683	0.2471	0.0604	0.7142
ML SE [r1,r2,c]	0.0084	0.0046	0.1058	0.0091	0.0050	0.1292

ML test Ctrl vs. Selection (r1 and r2 are line-specific)

c - control or selection specific

Line1[r1,r2,c]:	0.1871	0.0548	0.2683	0.2559	0.0533	0.7157
Line2[r1,r2,c]:	0.1978	0.0438	0.2683	0.2375	0.0600	0.7157
Line3[r1,r2,c]:	0.2138	0.0508	0.2683	0.2479	0.0680	0.7157

c-global

Line1[r1,r2,c]:	0.1872	0.0548	0.5402	0.2555	0.0532	0.5402
Line2[r1,r2,c]:	0.1983	0.0439	0.5402	0.2373	0.0600	0.5402

Line3[r1,r2,c]:	0.2140	0.0508	0.5402	0.2474	0.0678	0.5402

ML ratio (df=14 – 13=1): 6.3543

<u>*ML test Ctrl vs. Selection (r1, r2 and c are all line-specific)</u>

Line1[r1,r2,c]:	0.1867	0.0547	0.0000	0.2560	0.0533	0.7817
Line2[r1,r2,c]:	0.1986	0.0440	0.9167	0.2373	0.0600	0.5620
Line3[r1,r2,c]:	0.2134	0.0507	0.0000	0.2480	0.0680	0.7911

ML ratio (df=18 – 13=5): 22.7918

ML ratio for heterogeneity (control+selection; df = 5–1=4): 22.7918 – 6.3543

…………………………………………………………………………………………………..

markers 3-4, 5-6 (*th-cu*, *sr-e*)

	CONTROL			DESICCATION		
**Lines (ML estimates)**						
Line1 [r1,r2,c]:	0.0560	0.0547	0.0000	0.0480	0.0533	0.0000
Line2 [r1,r2,c]:	0.0560	0.0440	0.0000	0.0574	0.0600	0.3938
Line3 [r1,r2,c]:	0.0573	0.0507	0.4629	0.0427	0.0680	0.0000
Bailey test for 3 × 750						
Teta [r1,r2,c]:	0.0564	0.0494	0.0000	0.0487	0.0598	0.0000
SE [r1,r2,c]:	0.0049	0.0046	0.0003	0.0045	0.0050	0.0003
Chi2:		2.0277			4.1223	
C Chi2:		1.0617			1.0659	

Bailey for 6 × 750:

Teta:	0.0523	0.0541	0.0000
Chi2:	9.7326		
C chi2:	2.1276		

chi^2(ctrl + sel) - chi^2(ctrl) - chi^2(sel): 3.5827
C chi^2(ctrl + sel) - chi^2(ctrl) - chi^2(sel): **0.0000**

<u>ML test Ctrl Vs. Selection</u>

ML est [r1,r2,c]	0.0564	0.0498	0.1584	0.0493	0.0604	0.1493
ML SE [r1,r2,c]	0.0049	0.0046	0.1569	0.0046	0.0050	0.1479

<u>ML test Ctrl vs. Selection (r1 and r2 are line-specific)</u>

c - control or selection specific

Line1[r1,r2,c]:	0.0560	0.0547	0.1600	0.0480	0.0533	0.1500
Line2[r1,r2,c]:	0.0560	0.0440	0.1600	0.0573	0.0599	0.1500
Line3[r1,r2,c]:	0.0573	0.0506	0.1600	0.0427	0.0680	0.1500

c-global

Line1[r1,r2,c]:	0.0560	0.0547	0.1541	0.0480	0.0533	0.1541
Line2[r1,r2,c]:	0.0560	0.0440	0.1541	0.0573	0.0600	0.1541
Line3[r1,r2,c]:	0.0573	0.0506	0.1541	0.0427	0.0680	0.1541

ML ratio (df=14 – 13=1): 0.0013

<u>*ML test Ctrl vs. Selection (r1, r2 and c are all line-specific)</u>

Line1[r1,r2,c]:	0.0560	0.0547	0.0000	0.0480	0.0533	0.0000
Line2[r1,r2,c]:	0.0560	0.0440	0.0000	0.0574	0.0600	0.3938
Line3[r1,r2,c]:	0.0573	0.0507	0.4629	0.0427	0.0680	0.0000

ML ratio (df=18 – 13=5): 4.0949

ML ratio for heterogeneity (control+selection; df = 5–1=4): 4.0949 – 0.0013

……………………………………………………………………………………………………..

(B) Hypoxia and Hyperoxia experiment

X chromosome

markers 1-2, 3-4 (*y-cv*, *v-f*)

	CONTROL			HYPOXIA		
**Lines (ML estimates)**						
Line1 [r1,r2,c]:	0.1293	0.2267	1.1825	0.1767	0.3092	1.8619
Line2 [r1,r2,c]:	0.1347	0.2293	1.2089	0.1524	0.2888	1.7619
Line3 [r1,r2,c]:	0.1307	0.2213	0.8298	0.2019	0.2754	2.0199
Bailey test for 3 × 750						
Teta [r1,r2,c]:	0.1316	0.2260	1.0607	0.1759	0.2897	1.9046
SE [r1,r2,c]:	0.0071	0.0088	0.1015	0.0080	0.0095	0.0791
Chi2:		3.2939			12.5799	
C Chi2:		3.0537			1.9403	

Bailey for 6 × 750:

Teta:	0.1511	0.2576	1.6701
Chi2:	109.3154		
C chi2:	49.9453		

chi^2(ctrl + sel) - chi^2(ctrl) - chi^2(sel): 93.4416
C chi^2(ctrl + sel) - chi^2(ctrl) - chi^2(sel): **44.9513**

ML test Ctrl Vs. Selection

ML est [r1,r2,c]	0.1316	0.2258	1.0774	0.1770	0.2911	1.8862
ML SE [r1,r2,c]	0.0071	0.0088	0.1025	0.0080	0.0096	0.0793

ML test Ctrl vs. Selection (r1 and r2 are line-specific)

c - control or selection specific

Line1[r1,r2,c]:	0.1294	0.2267	1.0781	0.1760	0.3080	1.8918
Line2[r1,r2,c]:	0.1347	0.2294	1.0781	0.1505	0.2852	1.8918
Line3[r1,r2,c]:	0.1306	0.2211	1.0781	0.2053	0.2800	1.8918

c-global

Line1[r1,r2,c]:	0.1273	0.2231	1.6400	0.1802	0.3154	1.6400
Line2[r1,r2,c]:	0.1325	0.2257	1.6400	0.1536	0.2910	1.6400
Line3[r1,r2,c]:	0.1271	0.2154	1.6400	0.2087	0.2848	1.6400

ML ratio (df=14 – 13=1): 36.2925

***ML test Ctrl vs. Selection (r1, r2 and c are all line-specific)**

Line1[r1,r2,c]:	0.1293	0.2267	1.1825	0.1767	0.3092	1.8619
Line2[r1,r2,c]:	0.1347	0.2293	1.2089	0.1524	0.2888	1.7619
Line3[r1,r2,c]:	0.1307	0.2213	0.8298	0.2019	0.2754	2.0199

ML ratio (df=18 – 13=5): 40.8866

ML ratio for heterogeneity (control+selection; df = 5–1=4): 40.8866 – 36.2925

……………………………………………………………………………………………………

markers 1-2, 3-4 (*y-cv*, *v-f*)

	CONTROL			HYPEROXIA		
**Lines (ML estimates)**						
Line1 [r1,r2,c]:	0.1293	0.2267	1.1825	0.1773	0.2827	2.2344
Line2 [r1,r2,c]:	0.1347	0.2293	1.2089	0.1493	0.2573	1.7348
Line3 [r1,r2,c]:	0.1307	0.2213	0.8298	0.1547	0.2867	1.7141
Bailey test for 3 × 750						
Teta [r1,r2,c]:	0.1316	0.2260	1.0607	0.1612	0.2773	1.9453
SE [r1,r2,c]:	0.0071	0.0088	0.1015	0.0077	0.0093	0.0872
Chi2:		3.2939			15.7385	
C Chi2:		3.0537			9.1774	

Bailey for 6 × 750:
Teta: 0.1457 0.2536 1.6333

Chi2:	95.2360	
C chi2:	57.2264	

chi^2(ctrl + sel) - chi^2(ctrl) - chi^2(sel): 76.2036
C chi^2(ctrl + sel) - chi^2(ctrl) - chi^2(sel): **44.9953**

ML test Ctrl Vs. Selection

ML est [r1,r2,c]	0.1316	0.2258	1.0774	0.1604	0.2755	1.9201
ML SE [r1,r2,c]	0.0071	0.0088	0.1025	0.0077	0.0094	0.0883

ML test Ctrl vs. Selection (r1 and r2 are line-specific)

c - control or selection specific

Line1[r1,r2,c]:	0.1294	0.2267	1.0782	0.1844	0.2939	1.9298
Line2[r1,r2,c]:	0.1347	0.2294	1.0782	0.1470	0.2533	1.9298
Line3[r1,r2,c]:	0.1305	0.2211	1.0782	0.1513	0.2805	1.9298

c-global

Line1[r1,r2,c]:	0.1274	0.2233	1.6259	0.1859	0.2963	1.6259
Line2[r1,r2,c]:	0.1326	0.2259	1.6259	0.1502	0.2587	1.6259
Line3[r1,r2,c]:	0.1273	0.2156	1.6259	0.1555	0.2883	1.6259

ML ratio (df=14 – 13=1): 37.0231

***ML test Ctrl vs. Selection (r1, r2 and c are all line-specific)**

Line1[r1,r2,c]:	0.1293	0.2267	1.1825	0.1773	0.2827	2.2344
Line2[r1,r2,c]:	0.1347	0.2293	1.2089	0.1493	0.2573	1.7348
Line3[r1,r2,c]:	0.1307	0.2213	0.8298	0.1547	0.2867	1.7141

ML ratio (df=18 – 13=5): 47.9558

ML ratio for heterogeneity (control+selection; df = 5–1=4): 47.9558 – 37.0231

……

2L chromosome

markers 1-2, 3-4 (*net-dp*, *b-pk*)

	CONTROL			HYPOXIA		
Bailey test for 3 × 750						
Teta [r1,r2,c]:	0.1221	0.0667	0.4559	0.1703	0.0801	1.5452
SE [r1,r2,c]:	0.0069	0.0052	0.1467	0.0079	0.0057	0.1812
Chi2:		9.2711			3.1703	
C Chi2:		2.9328			1.2850	

Bailey for 6 × 750:

Teta:	0.1431	0.0722	0.9050
Chi2:	59.2040		
C chi2:	26.0444		

chi^2(ctrl + sel) - chi^2(ctrl) - chi^2(sel): 46.7625
C chi^2(ctrl + sel) - chi^2(ctrl) - chi^2(sel): **21.8266**

ML test Ctrl Vs. Selection

ML est [r1,r2,c]	0.1231	0.0675	0.5878	0.1707	0.0800	1.5628
ML SE [r1,r2,c]	0.0069	0.0053	0.1671	0.0079	0.0057	0.1824

ML test Ctrl vs. Selection (r1 and r2 are line-specific)

c - control or selection specific

Line1[r1,r2,c]:	0.1252	0.0746	0.5819	0.1672	0.0829	1.5596
Line2[r1,r2,c]:	0.1066	0.0560	0.5819	0.1599	0.0773	1.5596
Line3[r1,r2,c]:	0.1375	0.0721	0.5819	0.1848	0.0798	1.5596

c-global

Line1[r1,r2,c]:	0.1252	0.0746	1.2055	0.1671	0.0829	1.2055
Line2[r1,r2,c]:	0.1066	0.0560	1.2055	0.1602	0.0774	1.2055
Line3[r1,r2,c]:	0.1370	0.0718	1.2055	0.1854	0.0800	1.2055

ML ratio (df=14 – 13=1): 13.5168

***ML test Ctrl vs. Selection (r1, r2 and c are all line-specific)**

Line1[r1,r2,c]:	0.1253	0.0746	0.8562	0.1667	0.0827	1.8399
Line2[r1,r2,c]:	0.1066	0.0560	0.6714	0.1600	0.0773	1.5085
Line3[r1,r2,c]:	0.1373	0.0720	0.2697	0.1853	0.0800	1.3498

ML ratio (df=18 – 13=5): 17.5017

ML ratio for heterogeneity (control+selection; df = 5–1=4): 17.5017 – 13.5168

…………………………………………………………………………………………………..

markers 1-2, 3-5 (*net-dp, b-cn*)

	CONTROL			HYPOXIA		
**Lines (ML estimates)**						
Line1 [r1,r2,c]:	0.1253	0.0987	0.7563	0.1666	0.1027	1.7149
Line2 [r1,r2,c]:	0.1067	0.0800	0.6259	0.1600	0.0960	1.5612
Line3 [r1,r2,c]:	0.1373	0.0880	0.4416	0.1853	0.1027	1.4014
Bailey test for 3 × 750						
Teta [r1,r2,c]:	0.1219	0.0884	0.5777	0.1702	0.1005	1.5450
SE [r1,r2,c]:	0.0069	0.0060	0.1428	0.0079	0.0063	0.1593
Chi2:		6.0720			2.6015	

C Chi2:	0.9177	0.6864

Bailey for 6 × 750:

Teta:	0.1431 0.0933 1.0280
Chi2:	53.0526
C chi2:	22.0707

chi^2(ctrl + sel) - chi^2(ctrl) - chi^2(sel): 44.3791
C chi^2(ctrl + sel) - chi^2(ctrl) - chi^2(sel): **20.4666**

ML test Ctrl Vs. Selection

ML est [r1,r2,c]	0.1231	0.0889	0.6092	0.1707	0.1004	1.5557
ML SE [r1,r2,c]	0.0069	0.0060	0.1469	0.0079	0.0063	0.1600

ML test Ctrl vs. Selection (r1 and r2 are line-specific)

c - control or selection specific

Line1[r1,r2,c]:	0.1252	0.0986	0.6060	0.1671	0.1029	1.5515
Line2[r1,r2,c]:	0.1067	0.0800	0.6060	0.1600	0.0960	1.5515
Line3[r1,r2,c]:	0.1375	0.0881	0.6060	0.1849	0.1024	1.5515

c-global

Line1[r1,r2,c]:	0.1251	0.0985	1.2005	0.1671	0.1029	1.2005
Line2[r1,r2,c]:	0.1065	0.0799	1.2005	0.1602	0.0961	1.2005
Line3[r1,r2,c]:	0.1370	0.0878	1.2005	0.1855	0.1028	1.2005

ML ratio (df=14 – 13=1): 16.5003

***ML test Ctrl vs. Selection (r1, r2 and c are all line-specific)**

Line1[r1,r2,c]:	0.1253	0.0987	0.7563	0.1666	0.1027	1.7149
Line2[r1,r2,c]:	0.1067	0.0800	0.6259	0.1600	0.0960	1.5612
Line3[r1,r2,c]:	0.1373	0.0880	0.4416	0.1853	0.1027	1.4014

ML ratio (df=18 – 13=5): 18.0635

ML ratio for heterogeneity (control+selection; df = 5–1=4): 18.0635 –16.5003

…………………………………………………………………………………………………..

markers 1-2, 4-5 (*net-dp*, *pk-cn*)

	CONTROL			HYPOXIA		
**Lines (ML estimates)**						
Line1 [r1,r2,c]:	0.1253	0.0240	0.4463	0.1667	0.0227	1.0560
Line2 [r1,r2,c]:	0.1067	0.0240	0.5220	0.1600	0.0187	1.7887
Line3 [r1,r2,c]:	0.1373	0.0187	1.0356	0.1853	0.0227	1.5860

Bailey test for 3 × 750

Teta [r1,r2,c]:	0.1221	0.0219	0.5852	0.1703	0.0212	1.4138

SE [r1,r2,c]:	0.0069 0.0031 0.2940	0.0079 0.0030 0.3527
Chi2:	4.6768	2.8954
C Chi2:	0.5679	0.7669

Bailey for 6 × 750:
Teta: 0.1430 0.0213 0.9368
Chi2: 32.1457
C chi2: 4.5851

chi^2(ctrl + sel) - chi^2(ctrl) - chi^2(sel): 24.5735
C chi^2(ctrl + sel) - chi^2(ctrl) - chi^2(sel): **3.2503**

ML test Ctrl Vs. Selection

ML est [r1,r2,c]	0.1230 0.0222 0.6467	0.1706 0.0213 1.4696
ML SE [r1,r2,c]	0.0069 0.0031 0.3086	0.0079 0.0030 0.3615

ML test Ctrl vs. Selection (r1 and r2 are line-specific)

c - control or selection specific

Line1[r1,r2,c]:	0.1253	0.0240	0.6717	0.1665	0.0226	1.4598
Line2[r1,r2,c]:	0.1067	0.0240	0.6717	0.1601	0.0187	1.4598
Line3[r1,r2,c]:	0.1373	0.0187	0.6717	0.1854	0.0227	1.4598

c-global

Line1[r1,r2,c]:	0.1253	0.0240	1.1447	0.1667	0.0227	1.1447
Line2[r1,r2,c]:	0.1066	0.0240	1.1447	0.1601	0.0187	1.1447
Line3[r1,r2,c]:	0.1373	0.0187	1.1447	0.1854	0.0227	1.1447

ML ratio (df=14 – 13=1): 2.5887

***ML test Ctrl vs. Selection (r1, r2 and c are all line-specific)**

Line1[r1,r2,c]:	0.1253	0.0240	0.4463	0.1667	0.0227	1.0560
Line2[r1,r2,c]:	0.1067	0.0240	0.5220	0.1600	0.0187	1.7887
Line3[r1,r2,c]:	0.1373	0.0187	1.0356	0.1853	0.0227	1.586

ML ratio (df=18 – 13=5): 3.9746

ML ratio for heterogeneity (control+selection; df = 5–1=4): 3.9746 – 2.5887

……………………………………………………………………………………………………..

markers 1-3, 4-5 (*net-b*, *pk-cn*)

	CONTROL	HYPOXIA
**Lines (ML estimates)**		
Line1 [r1,r2,c]:	0.4000 0.0240 0.4174	0.3601 0.0227 1.1395
Line2 [r1,r2,c]:	0.4054 0.0240 0.0000	0.3814 0.0187 1.6856
Line3 [r1,r2,c]:	0.4040 0.0187 0.8850	0.3933 0.0227 0.7486

Bailey test for 3 × 750		
Teta [r1,r2,c]:	0.4030 0.0219 0.0000	0.3794 0.0212 1.1332
SE [r1,r2,c]:	0.0103 0.0031 0.0006	0.0102 0.0030 0.1789
Chi2:	12.3684	7.0088
C Chi2:	11.5895	4.6664

Bailey for 6 × 750:

Teta:	0.3920 0.0217 0.0000
Chi2:	61.7919
C chi2:	56.6059

chi^2(ctrl + sel) - chi^2(ctrl) - chi^2(sel): 42.4147
C chi^2(ctrl + sel) - chi^2(ctrl) - chi^2(sel): **40.3501**

<u>ML test Ctrl Vs. Selection</u>

ML est [r1,r2,c]	0.4031 0.0222 0.3980	0.3782 0.0213 1.1563
ML SE [r1,r2,c]	0.0103 0.0031 0.1280	0.0102 0.0030 0.1872

<u>ML test Ctrl vs. Selection (r1 and r2 are line-specific)</u>

c - control or selection specific

Line1[r1,r2,c]:	0.3999	0.0240	0.3965	0.3600	0.0227	1.1575
Line2[r1,r2,c]:	0.4065	0.0241	0.3965	0.3817	0.0187	1.1575
Line3[r1,r2,c]:	0.4029	0.0186	0.3965	0.3929	0.0226	1.1575

c-global

Line1[r1,r2,c]:	0.4004	0.0240	0.7512	0.3595	0.0227	0.7512
Line2[r1,r2,c]:	0.4064	0.0241	0.7512	0.3804	0.0186	0.7512
Line3[r1,r2,c]:	0.4038	0.0187	0.7512	0.3933	0.0227	0.7512

ML ratio (df=14 – 13=1): 10.7154

<u>*ML test Ctrl vs. Selection (r1, r2 and c are all line-specific)</u>

Line1[r1,r2,c]:	0.4000	0.0240	0.4174	0.3601	0.0227	1.1395
Line2[r1,r2,c]:	0.4054	0.0240	0.0000	0.3814	0.0187	1.6856
Line3[r1,r2,c]:	0.4040	0.0187	0.8850	0.3933	0.0227	0.7486

ML ratio (df=18 – 13=5): 24.5555

ML ratio for heterogeneity (control+selection; df = 5–1=4): 24.5555 –10.7154

……………………………………………………………………………………………..

<u>markers 2-3, 4-5 (*dp-b*, *pk-cn*)</u>

CONTROL **HYPOXIA**

<u>Lines (ML estimates)</u>

Line1 [r1,r2,c]:	0.2960 0.0240 0.3746	0.3693 0.0227 0.9567
Line2 [r1,r2,c]:	0.3094 0.0240 0.1795	0.3706 0.0187 1.3507
Line3 [r1,r2,c]:	0.3093 0.0187 0.6939	0.3760 0.0227 0.3135

Bailey test for 3 × 750

Teta [r1,r2,c]:	0.3050 0.0219 0.3068	0.3729 0.0212 0.6684
SE [r1,r2,c]:	0.0097 0.0031 0.1322	0.0102 0.0030 0.1551
Chi2:	2.9487	7.9579
C Chi2:	1.8177	7.4577

Bailey for 6 × 750:

Teta: 0.3374 0.0214 0.4628

Chi2: 37.2160

C chi2: 12.4450

chi^2(ctrl + sel) - chi^2(ctrl) - chi^2(sel): 26.3093

C chi^2(ctrl + sel) - chi^2(ctrl) - chi^2(sel): **3.1696**

ML test Ctrl Vs. Selection

ML est [r1,r2,c]	0.3049 0.0222 0.3939	0.3720 0.0213 0.8368
ML SE [r1,r2,c]	0.0097 0.0031 0.1500	0.0102 0.0030 0.1779

ML test Ctrl vs. Selection (r1 and r2 are line-specific)

c - control or selection specific

Line1[r1,r2,c]:	0.2960	0.0240	0.3965	0.3692	0.0227	0.8385
Line2[r1,r2,c]:	0.3097	0.0240	0.3965	0.3703	0.0187	0.8385
Line3[r1,r2,c]:	0.3089	0.0186	0.3965	0.3764	0.0227	0.8385

c-global

Line1[r1,r2,c]:	0.2962	0.0240	0.6423	0.3689	0.0226	0.6423
Line2[r1,r2,c]:	0.3098	0.0240	0.6423	0.3698	0.0186	0.6423
Line3[r1,r2,c]:	0.3093	0.0187	0.6423	0.3765	0.0227	0.6423

ML ratio (df=14 – 13=1): 3.4171

***ML test Ctrl vs. Selection (r1, r2 and c are all line-specific)**

Line1[r1,r2,c]:	0.2960	0.0240	0.3746	0.3693	0.0227	0.9567
Line2[r1,r2,c]:	0.3094	0.0240	0.1795	0.3706	0.0187	1.3507
Line3[r1,r2,c]:	0.3093	0.0187	0.6939	0.3760	0.0227	0.3135

ML ratio (df=18 – 13=5): 11.3668

ML ratio for heterogeneity (control+selection; df = 5–1=4): 11.3668 – 3.4171

…………………………………………………………………………………………………..

markers 1-2, 3-4 (*net-dp*, *b-pk*)

	CONTROL			HYPEROXIA		
**Lines (ML estimates)**						
Line1 [r1,r2,c]:	0.1253	0.0746	0.8562	0.1547	0.0707	1.3422
Line2 [r1,r2,c]:	0.1066	0.0560	0.6714	0.1440	0.0560	1.1577
Line3 [r1,r2,c]:	0.1373	0.0720	0.2697	0.1373	0.0760	1.6592
Bailey test for 3 × 750						
Teta [r1,r2,c]:	0.1221	0.0667	0.4559	0.1448	0.0666	1.3877
SE [r1,r2,c]:	0.0069	0.0052	0.1467	0.0074	0.0052	0.2131
Chi2:		9.2711			4.5981	
C Chi2:		2.9328			0.8795	

Bailey for 6 × 750:

Teta:	0.1329	0.0665	0.7597
Chi2:	32.0550		
C chi2:	16.6625		

chi^2(ctrl + sel) - chi^2(ctrl) - chi^2(sel): 18.1858
C chi^2(ctrl + sel) - chi^2(ctrl) - chi^2(sel): **12.8501**

**ML test Ctrl Vs. Selection**						
ML est [r1,r2,c]	0.1231	0.0675	0.5878	0.1453	0.0675	1.4032
ML SE [r1,r2,c]	0.0069	0.0053	0.1671	0.0074	0.0053	0.2148

ML test Ctrl vs. Selection (r1 and r2 are line-specific)

c - control or selection specific						
Line1[r1,r2,c]:	0.1252	0.0746	0.5819	0.1546	0.0706	1.4058
Line2[r1,r2,c]:	0.1066	0.0560	0.5819	0.1438	0.0559	1.4058
Line3[r1,r2,c]:	0.1375	0.0721	0.5819	0.1375	0.0761	1.4058

c-global						
Line1[r1,r2,c]:	0.1253	0.0747	1.0294	0.1547	0.0707	1.0294
Line2[r1,r2,c]:	0.1067	0.0560	1.0294	0.1440	0.0560	1.0294
Line3[r1,r2,c]:	0.1373	0.0720	1.0294	0.1374	0.0760	1.0294

ML ratio (df=14 – 13=1): 8.8109

***ML test Ctrl vs. Selection (r1, r2 and c are all line-specific)**

Line1[r1,r2,c]:	0.1253	0.0746	0.8562	0.1547	0.0707	1.3422
Line2[r1,r2,c]:	0.1066	0.0560	0.6714	0.1440	0.0560	1.1577
Line3[r1,r2,c]:	0.1373	0.0720	0.2697	0.1373	0.0760	1.6592

ML ratio (df=18 – 13=5): 12.3877

ML ratio for heterogeneity (control+selection; df = 5–1=4): 12.3877–8.8109

……………………………………………………………………………………………..

markers 1-2, 3-5 (*net-dp, b-cn*)

	CONTROL			HYPEROXIA		
Bailey test for 3 × 750						
Teta [r1,r2,c]:	0.1219	0.0884	0.5777	0.1451	0.0842	1.4481
SE [r1,r2,c]:	0.0069	0.0060	0.1428	0.0074	0.0058	0.1892
Chi2:		6.0720			6.8698	
C Chi2:		0.9177			0.3747	

Bailey for 6 × 750:
Teta: 0.1332 0.0860 0.8982
Chi2: 32.1201
C chi2: 14.7170

chi^2(ctrl + sel) - chi^2(ctrl) - chi^2(sel): 19.1783
C chi^2(ctrl + sel) - chi^2(ctrl) - chi^2(sel): **13.4246**

ML test Ctrl Vs. Selection

ML est [r1,r2,c]	0.1231	0.0889	0.6092	0.1453	0.0862	1.4550
ML SE [r1,r2,c]	0.0069	0.0060	0.1469	0.0074	0.0059	0.1898

ML test Ctrl vs. Selection (r1 and r2 are line-specific)

c - control or selection specific						
Line1[r1,r2,c]:	0.1252	0.0986	0.6061	0.1545	0.0906	1.4537
Line2[r1,r2,c]:	0.1067	0.0800	0.6061	0.1442	0.0681	1.4537
Line3[r1,r2,c]:	0.1375	0.0881	0.6061	0.1373	0.1000	1.4537
c-global						
Line1[r1,r2,c]:	0.1253	0.0986	1.0626	0.1547	0.0907	1.0626
Line2[r1,r2,c]:	0.1066	0.0800	1.0626	0.1441	0.0680	1.0626
Line3[r1,r2,c]:	0.1372	0.0879	1.0626	0.1374	0.1000	1.0626

ML ratio (df=14 – 13=1): 11.9468

***ML test Ctrl vs. Selection (r1, r2 and c are all line-specific)**

Line1[r1,r2,c]:	0.1253	0.0987	0.7563	0.1547	0.0907	1.3317
Line2[r1,r2,c]:	0.1067	0.0800	0.6259	0.1440	0.0680	1.6325
Line3[r1,r2,c]:	0.1373	0.0880	0.4416	0.1374	0.1000	1.4555

ML ratio (df=18 – 13=5): 13.2137

ML ratio for heterogeneity (control+selection; df = 5–1=4): 13.2137–11.9468

……………………………………………………………………………………………..

markers 1-2, 4-5 (*net-dp, pk-cn*)

	CONTROL			HYPEROXIA		
**Lines (ML estimates)**						
Line1 [r1,r2,c]:	0.1253	0.0240	0.4463	0.1547	0.0227	1.9024
Line2 [r1,r2,c]:	0.1067	0.0240	0.5220	0.1441	0.0147	3.1474
Line3 [r1,r2,c]:	0.1373	0.0187	1.0356	0.1373	0.0240	0.8091
Bailey test for 3 × 750						
Teta [r1,r2,c]:	0.1221	0.0219	0.5852	0.1463	0.0195	1.4952
SE [r1,r2,c]:	0.0069	0.0031	0.2940	0.0074	0.0029	0.3928
Chi2:		4.6768			7.5485	
C Chi2:		0.5679			4.5793	

Bailey for 6 × 750:

Teta:	0.1336	0.0205	0.9208
Chi2:	21.8818		
C chi2:	8.5838		

chi^2(ctrl + sel) - chi^2(ctrl) - chi^2(sel): 9.6565
C chi^2(ctrl + sel) - chi^2(ctrl) - chi^2(sel): **3.4366**

**ML test Ctrl Vs. Selection**						
ML est [r1,r2,c]	0.1230	0.0222	0.6467	0.1453	0.0204	1.7981
ML SE [r1,r2,c]	0.0069	0.0031	0.3086	0.0074	0.0030	0.4389

ML test Ctrl vs. Selection (r1 and r2 are line-specific)

c - control or selection specific						
Line1[r1,r2,c]:	0.1254	0.0240	0.6720	0.1547	0.0227	1.8229
Line2[r1,r2,c]:	0.1067	0.0240	0.6720	0.1445	0.0148	1.8229
Line3[r1,r2,c]:	0.1373	0.0187	0.6720	0.1368	0.0239	1.8229
c-global						
Line1[r1,r2,c]:	0.1252	0.0240	1.2836	0.1548	0.0227	1.2836
Line2[r1,r2,c]:	0.1066	0.0240	1.2836	0.1442	0.0147	1.2836
Line3[r1,r2,c]:	0.1373	0.0187	1.2836	0.1373	0.0240	1.2836

ML ratio (df=14 – 13=1): 4.2557

***ML test Ctrl vs. Selection (r1, r2 and c are all line-specific)**

Line1[r1,r2,c]:	0.1253	0.0240	0.4463	0.1547	0.0227	1.9024
Line2[r1,r2,c]:	0.1067	0.0240	0.5220	0.1441	0.0147	3.1474
Line3[r1,r2,c]:	0.1373	0.0187	1.0356	0.1373	0.0240	0.8091

ML ratio (df=18 – 13=5): 9.1348

ML ratio for heterogeneity (control+selection; df = 5–1=4): 9.1348 –4.2557

…………………………………………………………………………………………………..

markers 1-3, 4-5 (*net-b, pk-cn*)

	CONTROL			HYPEROXIA		
**Lines (ML estimates)**						
Line1 [r1,r2,c]:	0.4000	0.0240	0.4174	0.3894	0.0227	0.6033
Line2 [r1,r2,c]:	0.4054	0.0240	0.0000	0.4227	0.0147	1.0726
Line3 [r1,r2,c]:	0.4040	0.0187	0.8850	0.3893	0.0240	0.2855
Bailey test for 3 × 750						
Teta [r1,r2,c]:	0.4030	0.0219	0.0000	0.4011	0.0194	0.5055
SE [r1,r2,c]:	0.0103	0.0031	0.0006	0.0103	0.0029	0.1407
Chi2:		12.3684			8.4817	
C Chi2:		11.5895			4.0772	

Bailey for 6 × 750:
Teta: 0.4018 0.0205 0.0000
Chi2: 34.1704
C chi2: 28.4280

chi^2(ctrl + sel) - chi^2(ctrl) - chi^2(sel): 13.3202
C chi^2(ctrl + sel) - chi^2(ctrl) - chi^2(sel): **12.7614**

**ML test Ctrl Vs. Selection**						
ML est [r1,r2,c]	0.4031	0.0222	0.3980	0.4004	0.0204	0.5989
ML SE [r1,r2,c]	0.0103	0.0031	0.1280	0.0103	0.0030	0.1561

ML test Ctrl vs. Selection (r1 and r2 are line-specific)

c - control or selection specific						
Line1[r1,r2,c]:	0.3999	0.0240	0.3971	0.3893	0.0227	0.6074
Line2[r1,r2,c]:	0.4065	0.0241	0.3971	0.4220	0.0147	0.6074
Line3[r1,r2,c]:	0.4029	0.0186	0.3971	0.3899	0.0240	0.6074
c-global						
Line1[r1,r2,c]:	0.4002	0.0240	0.4971	0.3891	0.0227	0.4971
Line2[r1,r2,c]:	0.4066	0.0241	0.4971	0.4217	0.0147	0.4971
Line3[r1,r2,c]:	0.4033	0.0186	0.4971	0.3898	0.0240	0.4971

ML ratio (df=14 – 13=1): 1.0559

***ML test Ctrl vs. Selection (r1, r2 and c are all line-specific)**

Line1[r1,r2,c]:	0.4000	0.0240	0.4174	0.3894	0.0227	0.6033
Line2[r1,r2,c]:	0.4054	0.0240	0.0000	0.4227	0.0147	1.0726
Line3[r1,r2,c]:	0.4040	0.0187	0.8850	0.3893	0.0240	0.2855

ML ratio (df=18 – 13=5): 14.5572

ML ratio for heterogeneity (control+selection; df = 5–1=4): 14.5572 –1.0559

……………………………………………………………………………………………………..

<u>markers 2-3, 4-5 (*dp-b*, *pk-cn*)</u>

	CONTROL			HYPEROXIA		
**<u>Lines (ML estimates)</u>**						
Line1 [r1,r2,c]:	0.2960	0.0240	0.3746	0.3493	0.0227	0.5052
Line2 [r1,r2,c]:	0.3094	0.0240	0.1795	0.3747	0.0147	0.9674
Line3 [r1,r2,c]:	0.3093	0.0187	0.6939	0.3614	0.0240	0.0000
Bailey test for 3 × 750						
Teta [r1,r2,c]:	0.3050	0.0219	0.3068	0.3621	0.0194	0.0000
SE [r1,r2,c]:	0.0097	0.0031	0.1322	0.0101	0.0029	0.0004
Chi2:		2.9487			13.2919	
C Chi2:		1.8177			10.0382	

Bailey for 6 × 750:

Teta:	0.3321	0.0206	0.0000
Chi2:	38.5976		
C chi2:	17.2551		

chi^2(ctrl + sel) - chi^2(ctrl) - chi^2(sel): 22.3570

C chi^2(ctrl + sel) - chi^2(ctrl) - chi^2(sel): **5.3993**

<u>ML test Ctrl Vs. Selection</u>

ML est [r1,r2,c]	0.3049	0.0222	0.3939	0.3618	0.0204	0.4207
ML SE [r1,r2,c]	0.0097	0.0031	0.1500	0.0101	0.0030	0.1456

<u>ML test Ctrl vs. Selection (r1 and r2 are line-specific)</u>

c - control or selection specific

Line1[r1,r2,c]:	0.2960	0.0240	0.3974	0.3492	0.0227	0.4233
Line2[r1,r2,c]:	0.3097	0.0240	0.3974	0.3739	0.0147	0.4233
Line3[r1,r2,c]:	0.3090	0.0187	0.3974	0.3623	0.0241	0.4233

c-global

Line1[r1,r2,c]:	0.2960	0.0240	0.4118	0.3491	0.0227	0.4118
Line2[r1,r2,c]:	0.3097	0.0240	0.4118	0.3738	0.0146	0.4118
Line3[r1,r2,c]:	0.3090	0.0187	0.4118	0.3622	0.0241	0.4118

ML ratio (df=14 – 13=1): 0.0188

***ML test Ctrl vs. Selection (r1, r2 and c are all line-specific)**

Line1[r1,r2,c]:	0.2960	0.0240	0.3746	0.3493	0.0227	0.5052
Line2[r1,r2,c]:	0.3094	0.0240	0.1795	0.3747	0.0147	0.9674
Line3[r1,r2,c]:	0.3093	0.0187	0.6939	0.3614	0.0240	0.0000

ML ratio (df=18 – 13=5): 10.7664

ML ratio for heterogeneity (control+selection; df = 5–1=4): 10.7664 – 0.0188

……………………………………………………………………………………………………..

2R chromosome

markers 1-2, 3-4 (*cn-kn*, *c-px*)

	CONTROL			HYPOXIA		
**Lines (ML estimates)**						
Line1 [r1,r2,c]:	0.1040	0.2547	0.3524	0.0987	0.2213	0.5495
Line2 [r1,r2,c]:	0.0947	0.2707	0.4683	0.1040	0.2147	0.2986
Line3 [r1,r2,c]:	0.0987	0.2187	0.3708	0.1093	0.2307	0.2115
Bailey test for 3 × 750						
Teta [r1,r2,c]:	0.0990	0.2467	0.3919	0.1039	0.2222	0.3012
SE [r1,r2,c]:	0.0063	0.0091	0.0781	0.0064	0.0088	0.0716
Chi2:		6.6387			4.2220	
C Chi2:		0.4133			3.0093	

Bailey for 6 × 750:
Teta: 0.1014 0.2340 0.3425
Chi2: 15.3679
C chi2: 4.1640

chi^2(ctrl + sel) - chi^2(ctrl) - chi^2(sel): 4.5071
C chi^2(ctrl + sel) - chi^2(ctrl) - chi^2(sel): **0.7414**

**ML test Ctrl Vs. Selection**						
ML est [r1,r2,c]	0.0991	0.2480	0.3978	0.1040	0.2222	0.3462
ML SE [r1,r2,c]	0.0063	0.0091	0.0786	0.0064	0.0088	0.0767

ML test Ctrl vs. Selection (r1 and r2 are line-specific)

c - control or selection specific

Line1[r1,r2,c]:	0.1041	0.2549	0.3983	0.0983	0.2206	0.3454
Line2[r1,r2,c]:	0.0945	0.2703	0.3983	0.1041	0.2148	0.3454
Line3[r1,r2,c]:	0.0987	0.2188	0.3983	0.1096	0.2312	0.3454

c-global

Line1[r1,r2,c]:	0.1040	0.2548	0.3728	0.0984	0.2207	0.3728
Line2[r1,r2,c]:	0.0945	0.2702	0.3728	0.1041	0.2149	0.3728
Line3[r1,r2,c]:	0.0987	0.2187	0.3728	0.1097	0.2313	0.3728

ML ratio (df=14 – 13=1): 0.2310

***ML test Ctrl vs. Selection (r1, r2 and c are all line-specific)**

Line1[r1,r2,c]:	0.1040	0.2547	0.3524	0.0987	0.2213	0.5495
Line2[r1,r2,c]:	0.0947	0.2707	0.4683	0.1040	0.2147	0.2986
Line3[r1,r2,c]:	0.0987	0.2187	0.3708	0.1093	0.2307	0.2115

ML ratio (df=18 – 13=5): 3.9845

ML ratio for heterogeneity (control+selection; df = 5–1=4): 3.9845 –0.2310

…………………………………………………………………………………………………..

markers 1-2, 3-5 (*cn-kn*, *c-sp*)

	CONTROL			HYPOXIA		
**Lines (ML estimates)**						
Line1 [r1,r2,c]:	0.1040	0.3120	0.2876	0.0987	0.2827	0.6215
Line2 [r1,r2,c]:	0.0947	0.3373	0.4175	0.1040	0.2720	0.4242
Line3 [r1,r2,c]:	0.0987	0.2907	0.6509	0.1093	0.3013	0.3238
Bailey test for 3 × 750						
Teta [r1,r2,c]:	0.0993	0.3128	0.4049	0.1040	0.2853	0.4216
SE [r1,r2,c]:	0.0063	0.0098	0.0689	0.0064	0.0095	0.0722
Chi2:		8.2547			5.0495	
C Chi2:		4.0060			2.6055	

Bailey for 6 × 750:

Teta:	0.1017	0.2987	0.4126
Chi2:	17.5365		
C chi2:	6.6390		

chi^2(ctrl + sel) - chi^2(ctrl) - chi^2(sel): 4.2323
C chi^2(ctrl + sel) - chi^2(ctrl) - chi^2(sel): **0.0274**

ML test Ctrl Vs. Selection

ML est [r1,r2,c]	0.0991	0.3133	0.4437	0.1040	0.2853	0.4493
ML SE [r1,r2,c]	0.0063	0.0098	0.0720	0.0064	0.0095	0.0745

ML test Ctrl vs. Selection (r1 and r2 are line-specific)

c - control or selection specific

Line1[r1,r2,c]:	0.1044	0.3131	0.4429	0.0983	0.2818	0.4480
Line2[r1,r2,c]:	0.0947	0.3375	0.4429	0.1040	0.2721	0.4480
Line3[r1,r2,c]:	0.0983	0.2895	0.4429	0.1096	0.3022	0.4480

c-global

Line1[r1,r2,c]:	0.1044	0.3131	0.4453	0.0983	0.2817	0.4453
Line2[r1,r2,c]:	0.0947	0.3375	0.4453	0.1040	0.2721	0.4453
Line3[r1,r2,c]:	0.0983	0.2895	0.4453	0.1096	0.3022	0.4453

ML ratio (df=14 – 13=1): 0.0023

***ML test Ctrl vs. Selection (r1, r2 and c are all line-specific)**

Line1[r1,r2,c]:	0.1040	0.3120	0.2876	0.0987	0.2827	0.6215
Line2[r1,r2,c]:	0.0947	0.3373	0.4175	0.1040	0.2720	0.4242
Line3[r1,r2,c]:	0.0987	0.2907	0.6509	0.1093	0.3013	0.3238

ML ratio (df=18 – 13=5): 6.8578

ML ratio for heterogeneity (control+selection; df = 5–1=4): 6.8578 – 0.0023

………………………………………………………………………………………………..

markers 1-2, 4-5 (*cn-kn*, *px-sp*)

	CONTROL			HYPOXIA		
**Lines (ML estimates)**						
Line1 [r1,r2,c]:	0.1040	0.0707	0.0000	0.0987	0.0667	0.8114
Line2 [r1,r2,c]:	0.0947	0.0773	0.1822	0.1040	0.0627	0.8206
Line3 [r1,r2,c]:	0.0987	0.0800	1.3545	0.1093	0.0840	0.5850
Bailey test for 3 × 750						
Teta [r1,r2,c]:	0.0989	0.0759	0.0000	0.1040	0.0701	0.7011
SE [r1,r2,c]:	0.0063	0.0056	0.0001	0.0064	0.0054	0.1920
Chi2:		12.2218			3.6459	
C Chi2:		11.3653			0.3621	

Bailey for 6 × 750:

Teta:	0.1013	0.0728	0.0000
Chi2:	30.0595		
C chi2:	25.1391		

chi^2(ctrl + sel) - chi^2(ctrl) - chi^2(sel): 14.1918

C chi^2(ctrl + sel) - chi^2(ctrl) - chi^2(sel): **13.4117**

ML test Ctrl Vs. Selection

ML est [r1,r2,c]	0.0991	0.0760	0.5308	0.1040	0.0711	0.7214
ML SE [r1,r2,c]	0.0063	0.0056	0.1686	0.0064	0.0054	0.1949

ML test Ctrl vs. Selection (r1 and r2 are line-specific)

c - control or selection specific

Line1[r1,r2,c]:	0.1042	0.0708	0.5339	0.0986	0.0667	0.7177
Line2[r1,r2,c]:	0.0948	0.0774	0.5339	0.1040	0.0627	0.7177
Line3[r1,r2,c]:	0.0983	0.0797	0.5339	0.1094	0.0840	0.7177

c-global

Line1[r1,r2,c]:	0.1042	0.0708	0.6246	0.0986	0.0666	0.6246
Line2[r1,r2,c]:	0.0948	0.0774	0.6246	0.1039	0.0626	0.6246
Line3[r1,r2,c]:	0.0984	0.0798	0.6246	0.1093	0.0840	0.6246

ML ratio (df=14 – 13=1): 0.5154

***ML test Ctrl vs. Selection (r1, r2 and c are all line-specific)**

Line1[r1,r2,c]:	0.1040	0.0707	0.0000	0.0987	0.0667	0.8114
Line2[r1,r2,c]:	0.0947	0.0773	0.1822	0.1040	0.0627	0.8206
Line3[r1,r2,c]:	0.0987	0.0800	1.3545	0.1093	0.0840	0.5850

ML ratio (df=18 – 13=5): 14.8762

ML ratio for heterogeneity (control+selection; df = 5–1=4): 14.8762 – 0.5154

……………………………………………………………………………………………………..

markers 1-3, 4-5 (*cn-c*, *px-sp*)

	CONTROL			HYPOXIA		
**Lines (ML estimates)**						
Line1 [r1,r2,c]:	0.1267	0.0707	0.0000	0.1160	0.0667	0.6905
Line2 [r1,r2,c]:	0.1160	0.0773	0.1486	0.1200	0.0627	0.7091
Line3 [r1,r2,c]:	0.1147	0.0800	1.3084	0.1253	0.0840	0.5073
Bailey test for 3 × 750						
Teta [r1,r2,c]:	0.1188	0.0761	0.0000	0.1205	0.0701	0.6051
SE [r1,r2,c]:	0.0068	0.0056	0.0005	0.0069	0.0054	0.1664
Chi2:		13.8471			3.4816	
C Chi2:		12.8032			0.3352	

Bailey for 6 × 750:

Teta: 0.1196 0.0729 0.0000

Chi2:	31.1899
C chi2:	26.4386

chi^2(ctrl + sel) - chi^2(ctrl) - chi^2(sel): 13.8612
C chi^2(ctrl + sel) - chi^2(ctrl) - chi^2(sel): **13.3002**

ML test Ctrl Vs. Selection

ML est [r1,r2,c]	0.1191 0.0760 0.4909	0.1204 0.0711 0.6230
ML SE [r1,r2,c]	0.0068 0.0056 0.1476	0.0069 0.0054 0.1689

ML test Ctrl vs. Selection (r1 and r2 are line-specific)

c - control or selection specific

Line1[r1,r2,c]:	0.1270	0.0708	0.4925	0.1160	0.0666	0.6202
Line2[r1,r2,c]:	0.1162	0.0775	0.4925	0.1200	0.0626	0.6202
Line3[r1,r2,c]:	0.1142	0.0797	0.4925	0.1254	0.0840	0.6202

c-global

Line1[r1,r2,c]:	0.1270	0.0708	0.5540	0.1159	0.0666	0.5540
Line2[r1,r2,c]:	0.1162	0.0775	0.5540	0.1199	0.0626	0.5540
Line3[r1,r2,c]:	0.1143	0.0797	0.5540	0.1254	0.0840	0.5540

ML ratio (df=14 – 13=1): 0.3284

***ML test Ctrl vs. Selection (r1, r2 and c are all line-specific)**

Line1[r1,r2,c]:	0.1267	0.0707	0.0000	0.1160	0.0667	0.6905
Line2[r1,r2,c]:	0.1160	0.0773	0.1486	0.1200	0.0627	0.7091
Line3[r1,r2,c]:	0.1147	0.0800	1.3084	0.1253	0.0840	0.5073

ML ratio (df=18 – 13=5): 17.3918

ML ratio for heterogeneity (control+selection; df = 5–1=4): 17.3918 –0.3284

…………………………………………………………………………………………………..

markers 1-2, 3-4 (*cn-kn*, *c-px*)

	CONTROL	HYPEROXIA
**Lines (ML estimates)**		
Line1 [r1,r2,c]:	0.1040 0.2547 0.3524	0.1080 0.2600 0.4273
Line2 [r1,r2,c]:	0.0947 0.2707 0.4683	0.1000 0.2240 0.7143
Line3 [r1,r2,c]:	0.0987 0.2187 0.3708	0.0933 0.2213 0.4518
Bailey test for 3 × 750		
Teta [r1,r2,c]:	0.0990 0.2467 0.3919	0.1004 0.2344 0.4998
SE [r1,r2,c]:	0.0063 0.0091 0.0781	0.0063 0.0089 0.0881

Chi2:	6.6387	6.9988
C Chi2:	0.4133	1.7971

Bailey for 6 × 750:

Teta:	0.0998 0.2404 0.4392
Chi2:	15.4754
C chi2:	3.0750

chi^2(ctrl + sel) - chi^2(ctrl) - chi^2(sel): 1.8379
C chi^2(ctrl + sel) - chi^2(ctrl) - chi^2(sel): **0.8646**

<u>ML test Ctrl Vs. Selection</u>

ML est [r1,r2,c]	0.0991 0.2480 0.3978	0.1004 0.2351 0.5270
ML SE [r1,r2,c]	0.0063 0.0091 0.0786	0.0063 0.0089 0.0904

<u>ML test Ctrl vs. Selection (r1 and r2 are line-specific)</u>

c - control or selection specific

Line1[r1,r2,c]:	0.1041	0.2549	0.3984	0.1082	0.2604	0.5238
Line2[r1,r2,c]:	0.0945	0.2703	0.3984	0.0998	0.2234	0.5238
Line3[r1,r2,c]:	0.0987	0.2188	0.3984	0.0934	0.2215	0.5238

c-global

Line1[r1,r2,c]:	0.1042	0.2551	0.4597	0.1081	0.2601	0.4597
Line2[r1,r2,c]:	0.0946	0.2706	0.4597	0.0996	0.2232	0.4597
Line3[r1,r2,c]:	0.0988	0.2189	0.4597	0.0933	0.2214	0.4597

ML ratio (df=14 – 13=1): 1.1072

<u>*ML test Ctrl vs. Selection (r1, r2 and c are all line-specific)</u>

Line1[r1,r2,c]:	0.1040	0.2547	0.3524	0.1080	0.2600	0.4273
Line2[r1,r2,c]:	0.0947	0.2707	0.4683	0.1000	0.2240	0.7143
Line3[r1,r2,c]:	0.0987	0.2187	0.3708	0.0933	0.2213	0.4518

ML ratio (df=18 – 13=5): 3.4976

ML ratio for heterogeneity (control+selection; df = 5–1=4): 3.4976 – 1.1072

……………………………………………………………………………………………………..

<u>markers 1-2, 3-5 (*cn-kn*, *c-sp*)</u>

	CONTROL	HYPEROXIA
Bailey test for 3 × 750		
Teta [r1,r2,c]:	0.0993 0.3128 0.4049	0.1000 0.3009 0.7287
SE [r1,r2,c]:	0.0063 0.0098 0.0689	0.0063 0.0096 0.0861
Chi2:	8.2547	10.6472

C Chi2:	4.0060	5.4861

Bailey for 6 × 750:
Teta: 0.0998 0.3069 0.5305
Chi2: 28.4686
C chi2: 17.9937

chi^2(ctrl + sel) - chi^2(ctrl) - chi^2(sel): 9.5668
C chi^2(ctrl + sel) - chi^2(ctrl) - chi^2(sel): **8.5015**

ML test Ctrl Vs. Selection

ML est [r1,r2,c]	0.0991 0.3133 0.4437	0.1004 0.3013 0.7636
ML SE [r1,r2,c]	0.0063 0.0098 0.0720	0.0063 0.0097 0.0889

ML test Ctrl vs. Selection (r1 and r2 are line-specific)

c - control or selection specific

Line1[r1,r2,c]:	0.1044	0.3131	0.4430	0.1081	0.3203	0.7629
Line2[r1,r2,c]:	0.0947	0.3375	0.4430	0.0997	0.3085	0.7629
Line3[r1,r2,c]:	0.0983	0.2895	0.4430	0.0935	0.2752	0.7629

c-global

Line1[r1,r2,c]:	0.1046	0.3137	0.6002	0.1078	0.3194	0.6002
Line2[r1,r2,c]:	0.0950	0.3384	0.6002	0.0993	0.3072	0.6002
Line3[r1,r2,c]:	0.0986	0.2904	0.6002	0.0934	0.2750	0.6002

ML ratio (df=14 – 13=1): 7.8139

***ML test Ctrl vs. Selection (r1, r2 and c are all line-specific)**

Line1[r1,r2,c]:	0.1040	0.3120	0.2876	0.1080	0.3200	0.6944
Line2[r1,r2,c]:	0.0947	0.3373	0.4175	0.1000	0.3093	1.0345
Line3[r1,r2,c]:	0.0987	0.2907	0.6509	0.0933	0.2747	0.5201

ML ratio (df=18 – 13=5): 17.4074

ML ratio for heterogeneity (control+selection; df = 5–1=4): 17.4074 – 7.8139

---

markers 1-2, 4-5 (*cn-kn*, *px-sp*)

	CONTROL	HYPEROXIA
**Lines (ML estimates)**		
Line1 [r1,r2,c]:	0.1040 0.0707 0.0000	0.1080 0.0760 1.4600
Line2 [r1,r2,c]:	0.0947 0.0773 0.1822	0.1000 0.0880 1.8172
Line3 [r1,r2,c]:	0.0987 0.0800 1.3545	0.0933 0.0694 0.6190
Bailey test for 3 × 750		
Teta [r1,r2,c]:	0.0989 0.0759 0.0000	0.1000 0.0772 1.1870

SE [r1,r2,c]:	0.0063 0.0056 0.0001	0.0063 0.0056 0.2264
Chi2:	12.2218	8.1453
C Chi2:	11.3653	5.2935

Bailey for 6 × 750:

Teta:	0.0997 0.0767 0.0000
Chi2:	47.9188
C chi2:	43.8007

chi^2(ctrl + sel) - chi^2(ctrl) - chi^2(sel): 27.5516
C chi^2(ctrl + sel) - chi^2(ctrl) - chi^2(sel): **27.1419**

ML test Ctrl Vs. Selection

ML est [r1,r2,c]	0.0991 0.0760 0.5308	0.1004 0.0778 1.3645
ML SE [r1,r2,c]	0.0063 0.0056 0.1686	0.0063 0.0056 0.2453

ML test Ctrl vs. Selection (r1 and r2 are line-specific)

c - control or selection specific

Line1[r1,r2,c]:	0.1042	0.0708	0.5333	0.1080	0.0760	1.3681
Line2[r1,r2,c]:	0.0948	0.0775	0.5333	0.1002	0.0882	1.3681
Line3[r1,r2,c]:	0.0984	0.0797	0.5333	0.0931	0.0692	1.3681

c-global

Line1[r1,r2,c]:	0.1040	0.0707	0.9607	0.1080	0.0760	0.9607
Line2[r1,r2,c]:	0.0947	0.0774	0.9607	0.1000	0.0880	0.9607
Line3[r1,r2,c]:	0.0987	0.0800	0.9607	0.0933	0.0693	0.9607

ML ratio (df=14 – 13=1): 7.7802

***ML test Ctrl vs. Selection (r1, r2 and c are all line-specific)**

Line1[r1,r2,c]:	0.1040	0.0707	0.0000	0.1080	0.0760	1.4600
Line2[r1,r2,c]:	0.0947	0.0773	0.1822	0.1000	0.0880	1.8172
Line3[r1,r2,c]:	0.0987	0.0800	1.3545	0.0933	0.0694	0.6190

ML ratio (df=18 – 13=5): 26.0458

ML ratio for heterogeneity (control+selection; df = 5–1=4): 26.0458 – 7.7802

……………………………………………………………………………………………..

markers 1-3, 4-5 (*cn-c*, *px-sp*)

	CONTROL	HYPEROXIA
**Lines (ML estimates)**		
Line1 [r1,r2,c]:	0.1267 0.0707 0.0000	0.1347 0.0760 1.1741
Line2 [r1,r2,c]:	0.1160 0.0773 0.1486	0.1200 0.0880 1.5162
Line3 [r1,r2,c]:	0.1147 0.0800 1.3084	0.1200 0.0693 0.6420

Bailey test for 3 × 750

Teta [r1,r2,c]:	0.1188	0.0761	0.0000	0.1244	0.0772	1.0514
SE [r1,r2,c]:	0.0068	0.0056	0.0005	0.0070	0.0056	0.1930
Chi2:		13.8471			6.4256	
C Chi2:		12.8032			3.5566	

Bailey for 6 × 750:

Teta:	0.1217	0.0766	0.0000
Chi2:	50.3407		
C chi2:	45.8481		

chi^2(ctrl + sel) - chi^2(ctrl) - chi^2(sel): 30.0680
C chi^2(ctrl + sel) - chi^2(ctrl) - chi^2(sel): **29.4882**

ML test Ctrl Vs. Selection

ML est [r1,r2,c]	0.1191	0.0760	0.4909	0.1249	0.0778	1.1444
ML SE [r1,r2,c]	0.0068	0.0056	0.1476	0.0070	0.0056	0.2024

ML test Ctrl vs. Selection (r1 and r2 are line-specific)

c - control or selection specific

Line1[r1,r2,c]:	0.1270	0.0708	0.4918	0.1347	0.0760	1.1479
Line2[r1,r2,c]:	0.1162	0.0775	0.4918	0.1201	0.0881	1.1479
Line3[r1,r2,c]:	0.1142	0.0797	0.4918	0.1199	0.0693	1.1479

c-global

Line1[r1,r2,c]:	0.1269	0.0708	0.8326	0.1346	0.0759	0.8326
Line2[r1,r2,c]:	0.1161	0.0774	0.8326	0.1198	0.0879	0.8326
Line3[r1,r2,c]:	0.1146	0.0799	0.8326	0.1200	0.0693	0.8326

ML ratio (df=14 – 13=1): 6.6988

***ML test Ctrl vs. Selection (r1, r2 and c are all line-specific)**

Line1[r1,r2,c]:	0.1267	0.0707	0.0000	0.1347	0.0760	1.1741
Line2[r1,r2,c]:	0.1160	0.0773	0.1486	0.1200	0.0880	1.5162
Line3[r1,r2,c]:	0.1147	0.0800	1.3084	0.1200	0.0693	0.6420

ML ratio (df=18 – 13=5): 26.5747

ML ratio for heterogeneity (control+selection; df = 5–1=4): 26.5747 – 6.6988

…………………………………………………………………………………………………..

3rd chromosome

markers 1-2, 3-4 (*ru-h, th-cu*)

CONTROL **HYPOXIA**

Lines (ML estimates)

Line1 [r1,r2,c]:	0.1320	0.0453	0.0000	0.1667	0.0840	0.1905
Line2 [r1,r2,c]:	0.1587	0.0693	0.2424	0.1360	0.0560	0.0000
Line3 [r1,r2,c]:	0.1587	0.0480	0.1751	0.1440	0.0627	0.0000

Bailey test for 3 × 750

Teta [r1,r2,c]:	0.1488	0.0524	0.0000	0.1481	0.0657	0.0000
SE [r1,r2,c]:	0.0075	0.0047	0.0006	0.0075	0.0052	0.0003
Chi2:		10.9555			10.4359	
C Chi2:		3.1601			2.1050	

Bailey for 6 × 750:

Teta: 0.1486 0.0583 0.0000

Chi2: 25.0092

C chi2: 5.2652

chi^2(ctrl + sel) - chi^2(ctrl) - chi^2(sel): 3.6178

C chi^2(ctrl + sel) - chi^2(ctrl) - chi^2(sel): **0.0000**

ML test Ctrl Vs. Selection

ML est [r1,r2,c]	0.1498	0.0542	0.1642	0.1489	0.0676	0.0884
ML SE [r1,r2,c]	0.0075	0.0048	0.0931	0.0075	0.0053	0.0619

ML test Ctrl vs. Selection (r1 and r2 are line-specific)

c - control or selection specific

Line1[r1,r2,c]:	0.1321	0.0454	0.1629	0.1664	0.0839	0.0873
Line2[r1,r2,c]:	0.1585	0.0693	0.1629	0.1361	0.0560	0.0873
Line3[r1,r2,c]:	0.1586	0.0480	0.1629	0.1441	0.0627	0.0873

c-global

Line1[r1,r2,c]:	0.1321	0.0454	0.1208	0.1665	0.0839	0.1208
Line2[r1,r2,c]:	0.1585	0.0692	0.1208	0.1361	0.0560	0.1208
Line3[r1,r2,c]:	0.1586	0.0480	0.1208	0.1441	0.0627	0.1208

ML ratio (df=14 – 13=1): 0.4935

***ML test Ctrl vs. Selection (r1, r2 and c are all line-specific)**

Line1[r1,r2,c]:	0.1320	0.0453	0.0000	0.1667	0.0840	0.1905
Line2[r1,r2,c]:	0.1587	0.0693	0.2424	0.1360	0.0560	0.0000
Line3[r1,r2,c]:	0.1587	0.0480	0.1751	0.1440	0.0627	0.0000

ML ratio (df=18 – 13=5): 5.4569

ML ratio for heterogeneity (control+selection; df = 5–1=4): 5.4569 – 0.4935

…………………………………………………………………………………………………..

<u>markers 1-2, 3-5 (*ru-h, th-sr*)</u>

	CONTROL			HYPOXIA		
**<u>Lines (ML estimates)</u>**						
Line1 [r1,r2,c]:	0.1320	0.0960	0.0000	0.1667	0.1773	0.4060
Line2 [r1,r2,c]:	0.1587	0.1387	0.3030	0.1360	0.1387	0.4242
Line3 [r1,r2,c]:	0.1587	0.1067	0.0788	0.1440	0.1413	0.3931
Bailey test for 3 × 750						
Teta [r1,r2,c]:	0.1486	0.1112	0.0000	0.1482	0.1511	0.4036
SE [r1,r2,c]:	0.0075	0.0066	0.0006	0.0075	0.0075	0.0830
Chi2:		16.9457			9.0134	
C Chi2:		6.5417			0.0212	

Bailey for 6 × 750:

Teta:	0.1479	0.1281	0.0000
Chi2:	64.7911		
C chi2:	30.6375		

chi^2(ctrl + sel) - chi^2(ctrl) - chi^2(sel): 38.8319
C chi^2(ctrl + sel) - chi^2(ctrl) - chi^2(sel): **24.0745**

**<u>ML test Ctrl Vs. Selection</u>**						
ML est [r1,r2,c]	0.1498	0.1138	0.1565	0.1489	0.1524	0.4112
ML SE [r1,r2,c]	0.0075	0.0067	0.0625	0.0075	0.0076	0.0839

<u>ML test Ctrl vs. Selection (r1 and r2 are line-specific)</u>

c - control or selection specific						
Line1[r1,r2,c]:	0.1322	0.0962	0.1556	0.1667	0.1773	0.4071
Line2[r1,r2,c]:	0.1582	0.1383	0.1556	0.1360	0.1386	0.4071
Line3[r1,r2,c]:	0.1588	0.1068	0.1556	0.1440	0.1414	0.4071
c-global						
Line1[r1,r2,c]:	0.1324	0.0963	0.3003	0.1663	0.1769	0.3003
Line2[r1,r2,c]:	0.1587	0.1387	0.3003	0.1358	0.1384	0.3003
Line3[r1,r2,c]:	0.1591	0.1070	0.3003	0.1438	0.1411	0.3003

ML ratio (df=14 – 13=1): 5.4783

<u>*ML test Ctrl vs. Selection (r1, r2 and c are all line-specific)</u>

Line1[r1,r2,c]:	0.1320	0.0960	0.0000	0.1667	0.1773	0.4060
Line2[r1,r2,c]:	0.1587	0.1387	0.3030	0.1360	0.1387	0.4242
Line3[r1,r2,c]:	0.1587	0.1067	0.0788	0.1440	0.1413	0.3931

ML ratio (df=18 – 13=5): 11.0409

ML ratio for heterogeneity (control+selection; df = 5–1=4): 11.0409 – 5.4783

……………………………………………………………………………………………..

markers 1-2, 3-6 (*ru-h, th-e*)

	CONTROL			HYPOXIA		
**Lines (ML estimates)**						
Line1 [r1,r2,c]:	0.1320	0.1360	0.1485	0.1667	0.2347	0.3750
Line2 [r1,r2,c]:	0.1587	0.1880	0.2235	0.1360	0.2000	0.4902
Line3 [r1,r2,c]:	0.1587	0.1533	0.1096	0.1440	0.2107	0.3516
Bailey test for 3 × 750						
Teta [r1,r2,c]:	0.1492	0.1570	0.1507	0.1483	0.2148	0.3908
SE [r1,r2,c]:	0.0075	0.0077	0.0516	0.0075	0.0086	0.0680
Chi2:		12.6054			7.3057	
C Chi2:		0.8709			0.6307	

Bailey for 6 × 750:

Teta:	0.1485	0.1822	0.2372
Chi2:	52.3008		
C chi2:	9.5115		

chi^2(ctrl + sel) - chi^2(ctrl) - chi^2(sel): 32.3896
C chi^2(ctrl + sel) - chi^2(ctrl) - chi^2(sel): **8.0099**

**ML test Ctrl Vs. Selection**						
ML est [r1,r2,c]	0.1498	0.1591	0.1678	0.1489	0.2151	0.4024
ML SE [r1,r2,c]	0.0075	0.0077	0.0544	0.0075	0.0087	0.0689

ML test Ctrl vs. Selection (r1 and r2 are line-specific)

c - control or selection specific						
Line1[r1,r2,c]:	0.1320	0.1360	0.1665	0.1668	0.2348	0.3996
Line2[r1,r2,c]:	0.1584	0.1877	0.1665	0.1358	0.1997	0.3996
Line3[r1,r2,c]:	0.1589	0.1535	0.1665	0.1441	0.2109	0.3996
c-global						
Line1[r1,r2,c]:	0.1323	0.1363	0.3012	0.1663	0.2341	0.3012
Line2[r1,r2,c]:	0.1590	0.1883	0.3012	0.1355	0.1992	0.3012
Line3[r1,r2,c]:	0.1592	0.1539	0.3012	0.1438	0.2104	0.3012

ML ratio (df=14 – 13=1): 6.6529

***ML test Ctrl vs. Selection (r1, r2 and c are all line-specific)**

Line1[r1,r2,c]:	0.1320	0.1360	0.1485	0.1667	0.2347	0.3750
Line2[r1,r2,c]:	0.1587	0.1880	0.2235	0.1360	0.2000	0.4902

Line3[r1,r2,c]:	0.1587	0.1533	0.1096	0.1440	0.2107	0.3516

ML ratio (df=18 – 13=5): 8.1975

ML ratio for heterogeneity (control+selection; df = 5–1=4): 8.1975 –6.6529

…………………………………………………………………………………………..

markers 1-2, 4-5 (*ru-h*, *cu-sr*)

	CONTROL			HYPOXIA		
**Lines (ML estimates)**						
Line1 [r1,r2,c]:	0.1320	0.0533	0.0000	0.1667	0.0987	0.5678
Line2 [r1,r2,c]:	0.1587	0.0693	0.3638	0.1360	0.0827	0.7117
Line3 [r1,r2,c]:	0.1587	0.0587	0.0000	0.1440	0.0840	0.6617
Bailey test for 3 × 750						
Teta [r1,r2,c]:	0.1487	0.0597	0.0000	0.1481	0.0881	0.6288
SE [r1,r2,c]:	0.0075	0.0050	0.0001	0.0075	0.0060	0.1341
Chi2:		8.1453			4.7745	
C Chi2:		3.2784			0.2086	

Bailey for 6 × 750:

Teta:	0.1478	0.0713	0.0000
Chi2:	47.8972		
C chi2:	25.6452		

chi^2(ctrl + sel) - chi^2(ctrl) - chi^2(sel): 34.9774
C chi^2(ctrl + sel) - chi^2(ctrl) - chi^2(sel): **22.1582**

ML test Ctrl Vs. Selection

ML est [r1,r2,c]	0.1498	0.0604	0.1474	0.1489	0.0884	0.6412
ML SE [r1,r2,c]	0.0075	0.0050	0.0837	0.0075	0.0060	0.1356

ML test Ctrl vs. Selection (r1 and r2 are line-specific)

c - control or selection specific

Line1[r1,r2,c]:	0.1321	0.0534	0.1467	0.1667	0.0987	0.6359
Line2[r1,r2,c]:	0.1583	0.0692	0.1467	0.1359	0.0826	0.6359
Line3[r1,r2,c]:	0.1589	0.0587	0.1467	0.1440	0.0840	0.6359

c-global

Line1[r1,r2,c]:	0.1322	0.0534	0.4386	0.1664	0.0985	0.4386
Line2[r1,r2,c]:	0.1587	0.0694	0.4386	0.1357	0.0825	0.4386
Line3[r1,r2,c]:	0.1591	0.0588	0.4386	0.1438	0.0839	0.4386

ML ratio (df=14 – 13=1): 8.4271

***ML test Ctrl vs. Selection (r1, r2 and c are all line-specific)**

Line1[r1,r2,c]:	0.1320	0.0533	0.0000	0.1667	0.0987	0.5678
Line2[r1,r2,c]:	0.1587	0.0693	0.3638	0.1360	0.0827	0.7117
Line3[r1,r2,c]:	0.1587	0.0587	0.0000	0.1440	0.0840	0.6617

ML ratio (df=18 – 13=5): 14.2516

ML ratio for heterogeneity (control+selection; df = 5–1=4): 14.2516 – 8.4271

………………………………………………………………………………………………..

markers 1-2, 4-6 (*ru-h*, *cu-e*)

	CONTROL			HYPOXIA		
**Lines (ML estimates)**						
Line1 [r1,r2,c]:	0.1320	0.0933	0.2165	0.1667	0.1587	0.4538
Line2 [r1,r2,c]:	0.1587	0.1187	0.2125	0.1360	0.1467	0.6684
Line3 [r1,r2,c]:	0.1587	0.1053	0.0798	0.1440	0.1533	0.4831
Bailey test for 3 × 750						
Teta [r1,r2,c]:	0.1490	0.1050	0.1351	0.1481	0.1531	0.5113
SE [r1,r2,c]:	0.0075	0.0065	0.0601	0.0075	0.0076	0.0917
Chi2:		7.2984			4.4938	
C Chi2:		1.2136			0.8802	

Bailey for 6 × 750:

Teta:	0.1482	0.1251	0.2476
Chi2:	46.2665		
C chi2:	13.9313		

chi^2(ctrl + sel) - chi^2(ctrl) - chi^2(sel): 34.4743
C chi^2(ctrl + sel) - chi^2(ctrl) - chi^2(sel): **11.8375**

ML test Ctrl Vs. Selection

ML est [r1,r2,c]	0.1498	0.1058	0.1683	0.1489	0.1529	0.5272
ML SE [r1,r2,c]	0.0075	0.0065	0.0672	0.0075	0.0076	0.0931

ML test Ctrl vs. Selection (r1 and r2 are line-specific)

c - control or selection specific

Line1[r1,r2,c]:	0.1319	0.0933	0.1671	0.1668	0.1588	0.5247
Line2[r1,r2,c]:	0.1585	0.1186	0.1671	0.1358	0.1464	0.5247
Line3[r1,r2,c]:	0.1589	0.1055	0.1671	0.1441	0.1534	0.5247

c-global

Line1[r1,r2,c]:	0.1322	0.0935	0.3794	0.1664	0.1584	0.3794
Line2[r1,r2,c]:	0.1590	0.1189	0.3794	0.1355	0.1461	0.3794
Line3[r1,r2,c]:	0.1592	0.1057	0.3794	0.1438	0.1531	0.3794

ML ratio (df=14 – 13=1): 8.8207

***ML test Ctrl vs. Selection (r1, r2 and c are all line-specific)**

Line1[r1,r2,c]:	0.1320	0.0933	0.2165	0.1667	0.1587	0.4538
Line2[r1,r2,c]:	0.1587	0.1187	0.2125	0.1360	0.1467	0.6684
Line3[r1,r2,c]:	0.1587	0.1053	0.0798	0.1440	0.1533	0.4831

ML ratio (df=18 – 13=5): 10.8047

ML ratio for heterogeneity (control+selection; df = 5–1=4): 10.8047 – 8.8207

---

markers 1-2, 5-6 (*ru-h, sr-e*)

	CONTROL			HYPOXIA		
**Lines (ML estimates)**						
Line1 [r1,r2,c]:	0.1321	0.0400	0.5034	0.1667	0.0680	0.2354
Line2 [r1,r2,c]:	0.1587	0.0493	0.0000	0.1360	0.0693	0.5654
Line3 [r1,r2,c]:	0.1587	0.0467	0.1802	0.1440	0.0747	0.2480
Bailey test for 3 × 750						
Teta [r1,r2,c]:	0.1489	0.0451	0.0000	0.1480	0.0707	0.2931
SE [r1,r2,c]:	0.0075	0.0044	0.0003	0.0075	0.0054	0.1073
Chi2:		7.2154			4.4091	
C Chi2:		3.2233			1.2465	

Bailey for 6 × 750:
Teta: 0.1485 0.0552 0.0000
Chi2: 32.5401
C chi2: 11.9850

chi^2(ctrl + sel) - chi^2(ctrl) - chi^2(sel): 20.9156
C chi^2(ctrl + sel) - chi^2(ctrl) - chi^2(sel): **7.5152**

ML test Ctrl Vs. Selection

ML est [r1,r2,c]	0.1498	0.0453	0.1963	0.1489	0.0707	0.3380
ML SE [r1,r2,c]	0.0075	0.0044	0.1111	0.0075	0.0054	0.1149

ML test Ctrl vs. Selection (r1 and r2 are line-specific)

c - control or selection specific

Line1[r1,r2,c]:	0.1318	0.0399	0.1945	0.1668	0.0681	0.3379

Line2[r1,r2,c]:	0.1589	0.0494	0.1945	0.1358	0.0692	0.3379
Line3[r1,r2,c]:	0.1587	0.0467	0.1945	0.1441	0.0747	0.3379

c-global

Line1[r1,r2,c]:	0.1319	0.0400	0.2813	0.1667	0.0680	0.2813
Line2[r1,r2,c]:	0.1589	0.0494	0.2813	0.1357	0.0692	0.2813
Line3[r1,r2,c]:	0.1588	0.0467	0.2813	0.1440	0.0747	0.2813

ML ratio (df=14 – 13=1): 0.7613

***ML test Ctrl vs. Selection (r1, r2 and c are all line-specific)**

Line1[r1,r2,c]:	0.1321	0.0400	0.5034	0.1667	0.0680	0.2354
Line2[r1,r2,c]:	0.1587	0.0493	0.0000	0.1360	0.0693	0.5654
Line3[r1,r2,c]:	0.1587	0.0467	0.1802	0.1440	0.0747	0.2480

ML ratio (df=18 – 13=5): 6.0589

ML ratio for heterogeneity (control+selection; df = 5–1=4): 6.0589 – 0.7613

…………………………………………………………………………………………………..

markers 1-3, 4-5 (*ru-th*, *cu-sr*)

	CONTROL			HYPOXIA		
**Lines (ML estimates)**						
Line1 [r1,r2,c]:	0.2200	0.0533	0.1136	0.2720	0.0987	0.5962
Line2 [r1,r2,c]:	0.2600	0.0693	0.2219	0.2347	0.0827	0.6186
Line3 [r1,r2,c]:	0.2761	0.0587	0.0000	0.2187	0.0840	0.7986
Bailey test for 3 × 750						
Teta [r1,r2,c]:	0.2502	0.0599	0.0000	0.2406	0.0882	0.6470
SE [r1,r2,c]:	0.0091	0.0050	0.0002	0.0090	0.0060	0.1027
Chi2:		13.4107			8.5557	
C Chi2:		4.2817			0.6521	

Bailey for 6 × 750:

Teta:	0.2440	0.0714	0.0000
Chi2:	74.6854		
C chi2:	45.1622		

chi^2(ctrl + sel) - chi^2(ctrl) - chi^2(sel): 52.7190
C chi^2(ctrl + sel) - chi^2(ctrl) - chi^2(sel): **40.2283**

ML test Ctrl Vs. Selection

ML est [r1,r2,c]	0.2520	0.0604	0.1167	0.2418	0.0885	0.6652

ML SE [r1,r2,c]	0.0092 0.0050 0.0572	0.0090 0.0060 0.1042

ML test Ctrl vs. Selection (r1 and r2 are line-specific)

c - control or selection specific

Line1[r1,r2,c]:	0.2200	0.0533	0.1160	0.2722	0.0987	0.6589
Line2[r1,r2,c]:	0.2595	0.0692	0.1160	0.2347	0.0827	0.6589
Line3[r1,r2,c]:	0.2765	0.0588	0.1160	0.2184	0.0839	0.6589

c-global

Line1[r1,r2,c]:	0.2205	0.0535	0.4347	0.2712	0.0984	0.4347
Line2[r1,r2,c]:	0.2607	0.0695	0.4347	0.2341	0.0825	0.4347
Line3[r1,r2,c]:	0.2773	0.0589	0.4347	0.2177	0.0836	0.4347

ML ratio (df=14 – 13=1): 18.4946

***ML test Ctrl vs. Selection (r1, r2 and c are all line-specific)**

Line1[r1,r2,c]:	0.2200	0.0533	0.1136	0.2720	0.0987	0.5962
Line2[r1,r2,c]:	0.2600	0.0693	0.2219	0.2347	0.0827	0.6186
Line3[r1,r2,c]:	0.2761	0.0587	0.0000	0.2187	0.0840	0.7986

ML ratio (df=18 – 13=5): 23.1427

ML ratio for heterogeneity (control+selection; df = 5–1=4): 23.1427 – 18.4946

…………………………………………………………………………………………………..

markers 1-3, 4-6 (*ru-th*, *cu-e*)

	CONTROL	HYPOXIA
**Lines (ML estimates)**		
Line1 [r1,r2,c]:	0.2200 0.0933 0.1948	0.2720 0.1587 0.4634
Line2 [r1,r2,c]:	0.2600 0.1187 0.2161	0.2347 0.1467 0.5036
Line3 [r1,r2,c]:	0.2760 0.1053 0.0459	0.2187 0.1533 0.5170
Bailey test for 3 × 750		
Teta [r1,r2,c]:	0.2505 0.1052 0.0922	0.2405 0.1531 0.4884
SE [r1,r2,c]:	0.0091 0.0065 0.0382	0.0090 0.0076 0.0688
Chi2:	14.3552	7.0376
C Chi2:	3.7330	0.1188

Bailey for 6 × 750:

Teta:	0.2445 0.1251 0.1848
Chi2:	68.6610
C chi2:	29.4245

chi^2(ctrl + sel) - chi^2(ctrl) - chi^2(sel): 47.2682
C chi^2(ctrl + sel) - chi^2(ctrl) - chi^2(sel): **25.5726**

ML test Ctrl Vs. Selection

ML est [r1,r2,c]	0.2520 0.1058 0.1501	0.2418 0.1529 0.4930
ML SE [r1,r2,c]	0.0092 0.0065 0.0486	0.0090 0.0076 0.0691

ML test Ctrl vs. Selection (r1 and r2 are line-specific)

c - control or selection specific

Line1[r1,r2,c]:	0.2198	0.0933	0.1490	0.2722	0.1588	0.4916
Line2[r1,r2,c]:	0.2595	0.1184	0.1490	0.2346	0.1466	0.4916
Line3[r1,r2,c]:	0.2767	0.1056	0.1490	0.2185	0.1532	0.4916

c-global

Line1[r1,r2,c]:	0.2205	0.0935	0.3489	0.2710	0.1581	0.3489
Line2[r1,r2,c]:	0.2608	0.1190	0.3489	0.2338	0.1461	0.3489
Line3[r1,r2,c]:	0.2778	0.1060	0.3489	0.2177	0.1527	0.3489

ML ratio (df=14 – 13=1): 14.9878

***ML test Ctrl vs. Selection (r1, r2 and c are all line-specific)**

Line1[r1,r2,c]:	0.2200	0.0933	0.1948	0.2720	0.1587	0.4634
Line2[r1,r2,c]:	0.2600	0.1187	0.2161	0.2347	0.1467	0.5036
Line3[r1,r2,c]:	0.2760	0.1053	0.0459	0.2187	0.1533	0.5170

ML ratio (df=18 – 13=5): 18.2094

ML ratio for heterogeneity (control+selection; df = 5–1=4): 18.2094 – 14.9878

…………………………………………………………………………………………………..

markers 1-3, 5-6 (*ru-th*, *sr-e*)

	CONTROL	HYPOXIA
**Lines (ML estimates)**		
Line1 [r1,r2,c]:	0.2200 0.0400 0.3033	0.2720 0.0680 0.3605
Line2 [r1,r2,c]:	0.2600 0.0493 0.2079	0.2347 0.0693 0.3277
Line3 [r1,r2,c]:	0.2760 0.0467 0.1035	0.2187 0.0747 0.3267
Bailey test for 3 × 750		
Teta [r1,r2,c]:	0.2506 0.0452 0.1602	0.2404 0.0706 0.3378
SE [r1,r2,c]:	0.0091 0.0044 0.0767	0.0090 0.0054 0.0888
Chi2:	9.1372	6.1189
C Chi2:	0.9081	0.0319

Bailey for 6 × 750:

Teta:	0.2457 0.0553 0.2355
Chi2:	31.0441
C chi2:	3.2169

chi^2(ctrl + sel) - chi^2(ctrl) - chi^2(sel): 15.7879
C chi^2(ctrl + sel) - chi^2(ctrl) - chi^2(sel): **2.2768**

ML test Ctrl Vs. Selection

ML est [r1,r2,c]	0.2520	0.0453	0.1946	0.2418	0.0707	0.3383
ML SE [r1,r2,c]	0.0092	0.0044	0.0844	0.0090	0.0054	0.0886

ML test Ctrl vs. Selection (r1 and r2 are line-specific)

c - control or selection specific

Line1[r1,r2,c]:	0.2198	0.0400	0.1928	0.2719	0.0680	0.3392
Line2[r1,r2,c]:	0.2600	0.0493	0.1928	0.2347	0.0693	0.3392
Line3[r1,r2,c]:	0.2763	0.0467	0.1928	0.2187	0.0747	0.3392

c-global

Line1[r1,r2,c]:	0.2200	0.0400	0.2803	0.2717	0.0679	0.2803
Line2[r1,r2,c]:	0.2602	0.0494	0.2803	0.2345	0.0693	0.2803
Line3[r1,r2,c]:	0.2765	0.0467	0.2803	0.2185	0.0746	0.2803

ML ratio (df=14 – 13=1): 1.3564

***ML test Ctrl vs. Selection (r1, r2 and c are all line-specific)**

Line1[r1,r2,c]:	0.2200	0.0400	0.3033	0.2720	0.0680	0.3605
Line2[r1,r2,c]:	0.2600	0.0493	0.2079	0.2347	0.0693	0.3277
Line3[r1,r2,c]:	0.2760	0.0467	0.1035	0.2187	0.0747	0.3267

ML ratio (df=18 – 13=5): 2.2848

ML ratio for heterogeneity (control+selection; df = 5–1=4):

…………………………………………………………………………………………………..

markers 1-4, 5-6 (*ru-cu*, *sr-e*)

Lines (ML estimates)

	CONTROL			HYPOXIA		
Line1 [r1,r2,c]:	0.2653	0.0400	0.2514	0.3373	0.0680	0.3487
Line2 [r1,r2,c]:	0.3213	0.0493	0.1682	0.2880	0.0693	0.3339
Line3 [r1,r2,c]:	0.3187	0.0467	0.0897	0.2707	0.0747	0.2640

Bailey test for 3 × 750

Teta [r1,r2,c]:	0.3006 0.0452 0.1363	0.2972 0.0706 0.3119
SE [r1,r2,c]:	0.0096 0.0044 0.0647	0.0096 0.0054 0.0762
Chi2:	9.7573	8.6490
C Chi2:	0.8123	0.2489

Bailey for 6 × 750:

Teta:	0.2992 0.0552 0.2091
Chi2:	34.7595
C chi2:	4.1389

chi^2(ctrl + sel) - chi^2(ctrl) - chi^2(sel): 16.3532
C chi^2(ctrl + sel) - chi^2(ctrl) - chi^2(sel): **3.0778**

ML test Ctrl Vs. Selection

ML est [r1,r2,c]	0.3018 0.0453 0.1625	0.2987 0.0707 0.3159
ML SE [r1,r2,c]	0.0097 0.0044 0.0705	0.0096 0.0054 0.0765

ML test Ctrl vs. Selection (r1 and r2 are line-specific)

c - control or selection specific

Line1[r1,r2,c]:	0.2651	0.0400	0.1610	0.3371	0.0680	0.3171
Line2[r1,r2,c]:	0.3213	0.0493	0.1610	0.2879	0.0693	0.3171
Line3[r1,r2,c]:	0.3190	0.0467	0.1610	0.2709	0.0747	0.3171

c-global

Line1[r1,r2,c]:	0.2653	0.0400	0.2552	0.3367	0.0679	0.2552
Line2[r1,r2,c]:	0.3217	0.0494	0.2552	0.2876	0.0692	0.2552
Line3[r1,r2,c]:	0.3193	0.0468	0.2552	0.2706	0.0747	0.2552

ML ratio (df=14 – 13=1): 2.0883

***ML test Ctrl vs. Selection (r1, r2 and c are all line-specific)**

Line1[r1,r2,c]:	0.2653	0.0400	0.2514	0.3373	0.0680	0.3487
Line2[r1,r2,c]:	0.3213	0.0493	0.1682	0.2880	0.0693	0.3339
Line3[r1,r2,c]:	0.3187	0.0467	0.0897	0.2707	0.0747	0.2640

ML ratio (df=18 – 13=5): 3.1366

ML ratio for heterogeneity (control+selection; df = 5−1=4): 3.1366 −2.0883

…………………………………………………………………………………………………..

markers 2-3, 4-5 (*h-th*, *cu-sr*)

	CONTROL	HYPOXIA
**Lines (ML estimates)**		
Line1 [r1,r2,c]:	0.0987 0.0533 0.2530	0.1507 0.0987 0.4485
Line2 [r1,r2,c]:	0.1307 0.0693 0.0000	0.1253 0.0827 0.3863

Line3 [r1,r2,c]:	0.1307 0.0587 0.0000	0.1200 0.0840 0.6621

Bailey test for 3 × 750

Teta [r1,r2,c]:	0.1183 0.0600 0.0000	0.1310 0.0881 0.4693
SE [r1,r2,c]:	0.0068 0.0050 0.0002	0.0071 0.0060 0.1259
Chi2:	8.4617	5.7378
C Chi2:	1.0389	0.6525

Bailey for 6 × 750:

Teta: 0.1241 0.0714 0.0000
Chi2: 43.0983
C chi2: 15.7188

chi^2(ctrl + sel) - chi^2(ctrl) - chi^2(sel): 28.8988
C chi^2(ctrl + sel) - chi^2(ctrl) - chi^2(sel): **14.0275**

<u>ML test Ctrl Vs. Selection</u>

ML est [r1,r2,c]	0.1200 0.0604 0.0613	0.1320 0.0884 0.4949
ML SE [r1,r2,c]	0.0069 0.0050 0.0609	0.0071 0.0060 0.1296

<u>ML test Ctrl vs. Selection (r1 and r2 are line-specific)</u>

c - control or selection specific

Line1[r1,r2,c]:	0.0986	0.0533	0.0605	0.1507	0.0987	0.4905
Line2[r1,r2,c]:	0.1307	0.0694	0.0605	0.1254	0.0827	0.4905
Line3[r1,r2,c]:	0.1307	0.0587	0.0605	0.1199	0.0839	0.4905

c-global

Line1[r1,r2,c]:	0.0987	0.0533	0.3269	0.1505	0.0985	0.3269
Line2[r1,r2,c]:	0.1309	0.0695	0.3269	0.1253	0.0826	0.3269
Line3[r1,r2,c]:	0.1309	0.0588	0.3269	0.1197	0.0838	0.3269

ML ratio (df=14 – 13=1): 7.7163

<u>*ML test Ctrl vs. Selection (r1, r2 and c are all line-specific)</u>

Line1[r1,r2,c]:	0.0987	0.0533	0.2530	0.1507	0.0987	0.4485
Line2[r1,r2,c]:	0.1307	0.0693	0.0000	0.1253	0.0827	0.3863
Line3[r1,r2,c]:	0.1307	0.0587	0.0000	0.1200	0.0840	0.6621

ML ratio (df=18 – 13=5): 11.3177

ML ratio for heterogeneity (control+selection; df = 5–1=4): 11.3177 – 7.7163

…………………………………………………………………………………………………..

<u>markers 2-3, 4-6 (*h-th*, *cu-e*)</u>

CONTROL **HYPOXIA**

Lines (ML estimates)

Line1 [r1,r2,c]:	0.0987	0.0933	0.1447	0.1507	0.1587	0.3347
Line2 [r1,r2,c]:	0.1307	0.1187	0.1720	0.1253	0.1467	0.2176
Line3 [r1,r2,c]:	0.1307	0.1053	0.0000	0.1200	0.1533	0.5072

Bailey test for 3 × 750

Teta [r1,r2,c]:	0.1182	0.1051	0.0000	0.1309	0.1530	0.3182
SE [r1,r2,c]:	0.0068	0.0065	0.0001	0.0071	0.0076	0.0793
Chi2:		11.6341			5.8492	
C Chi2:		3.1241			1.8296	

Bailey for 6 × 750:

Teta:	0.1241	0.1248	0.0000
Chi2:	59.1277		
C chi2:	21.1804		

chi^2(ctrl + sel) - chi^2(ctrl) - chi^2(sel): 41.6444
C chi^2(ctrl + sel) - chi^2(ctrl) - chi^2(sel): **16.2267**

ML test Ctrl Vs. Selection

ML est [r1,r2,c]	0.1200	0.1058	0.1050	0.1320	0.1529	0.3524
ML SE [r1,r2,c]	0.0069	0.0065	0.0599	0.0071	0.0076	0.0835

ML test Ctrl vs. Selection (r1 and r2 are line-specific)

c - control or selection specific

Line1[r1,r2,c]:	0.0986	0.0933	0.1039	0.1507	0.1587	0.3514
Line2[r1,r2,c]:	0.1305	0.1185	0.1039	0.1256	0.1469	0.3514
Line3[r1,r2,c]:	0.1308	0.1055	0.1039	0.1197	0.1530	0.3514

c-global

Line1[r1,r2,c]:	0.0987	0.0934	0.2564	0.1504	0.1584	0.2564
Line2[r1,r2,c]:	0.1308	0.1188	0.2564	0.1254	0.1467	0.2564
Line3[r1,r2,c]:	0.1310	0.1056	0.2564	0.1196	0.1528	0.2564

ML ratio (df=14 – 13=1): 5.1198

***ML test Ctrl vs. Selection (r1, r2 and c are all line-specific)**

Line1[r1,r2,c]:	0.0987	0.0933	0.1447	0.1507	0.1587	0.3347
Line2[r1,r2,c]:	0.1307	0.1187	0.1720	0.1253	0.1467	0.2176
Line3[r1,r2,c]:	0.1307	0.1053	0.0000	0.1200	0.1533	0.5072

ML ratio (df=18 – 13=5): 9.6886

ML ratio for heterogeneity (control+selection; df = 5–1=4): 9.6886 – 5.1198

……………………………………………………………………………………………………..

markers 2-3, 5-6 (*h-th*, *sr-e*)

	CONTROL			HYPOXIA		
**Lines (ML estimates)**						
Line1 [r1,r2,c]:	0.0987	0.0400	0.0000	0.1507	0.0680	0.3906
Line2 [r1,r2,c]:	0.1307	0.0493	0.4145	0.1253	0.0693	0.0000
Line3 [r1,r2,c]:	0.1307	0.0467	0.0000	0.1200	0.0747	0.5957
Bailey test for 3 × 750						
Teta [r1,r2,c]:	0.1181	0.0450	0.0000	0.1306	0.0705	0.0000
SE [r1,r2,c]:	0.0068	0.0044	0.0005	0.0071	0.0054	0.0006
Chi2:		8.5148			11.3073	
C Chi2:		2.1665			7.8098	

Bailey for 6 × 750:

Teta:	0.1242	0.0551	0.0000
Chi2:	35.6916		
C chi2:	**9.9763**		

chi^2(ctrl + sel) - chi^2(ctrl) - chi^2(sel): 15.8696
C chi^2(ctrl + sel) - chi^2(ctrl) - chi^2(sel): **0.0000**

ML test Ctrl Vs. Selection

ML est [r1,r2,c]	0.1200	0.0453	0.1634	0.1320	0.0707	0.3334
ML SE [r1,r2,c]	0.0069	0.0044	0.1140	0.0071	0.0054	0.1216

ML test Ctrl vs. Selection (r1 and r2 are line-specific)

c - control or selection specific						
Line1[r1,r2,c]:	0.0987	0.0400	0.1621	0.1506	0.0680	0.3348
Line2[r1,r2,c]:	0.1305	0.0493	0.1621	0.1256	0.0695	0.3348
Line3[r1,r2,c]:	0.1308	0.0467	0.1621	0.1198	0.0745	0.3348
c-global						
Line1[r1,r2,c]:	0.0987	0.0400	0.2711	0.1505	0.0679	0.2711
Line2[r1,r2,c]:	0.1306	0.0493	0.2711	0.1256	0.0695	0.2711
Line3[r1,r2,c]:	0.1308	0.0467	0.2711	0.1197	0.0745	0.2711

ML ratio (df=14 – 13=1): 0.9697

***ML test Ctrl vs. Selection (r1, r2 and c are all line-specific)**

Line1[r1,r2,c]:	0.0987	0.0400	0.0000	0.1507	0.0680	0.3906
Line2[r1,r2,c]:	0.1307	0.0493	0.4145	0.1253	0.0693	0.0000
Line3[r1,r2,c]:	0.1307	0.0467	0.0000	0.1200	0.0747	0.5957

ML ratio (df=18 – 13=5): 10.6094

ML ratio for heterogeneity (control+selection; df = 5–1=4): 10.6094 – 0.9697

…………………………………………………………………………………………………..

markers 2-4, 5-6 (*h-cu, sr-e*)

	CONTROL			HYPOXIA		
**Lines (ML estimates)**						
Line1 [r1,r2,c]:	0.1440	0.0400	0.0000	0.2160	0.0680	0.3632
Line2 [r1,r2,c]:	0.1973	0.0493	0.2741	0.1787	0.0693	0.1076
Line3 [r1,r2,c]:	0.1760	0.0467	0.0000	0.1720	0.0747	0.4155
Bailey test for 3 × 750						
Teta [r1,r2,c]:	0.1700	0.0450	0.0000	0.1873	0.0706	0.2190
SE [r1,r2,c]:	0.0079	0.0044	0.0002	0.0082	0.0054	0.0821
Chi2:		11.1839			8.1705	
C Chi2:		2.1495			2.8048	

Bailey for 6 × 750:

Teta:	0.1784	0.0550	0.0000
Chi2:	43.2045		
C chi2:	12.1150		

chi^2(ctrl + sel) - chi^2(ctrl) - chi^2(sel): 23.8502
C chi^2(ctrl + sel) - chi^2(ctrl) - chi^2(sel): **7.1607**

ML test Ctrl Vs. Selection

ML est [r1,r2,c]	0.1724	0.0453	0.1136	0.1889	0.0707	0.2996
ML SE [r1,r2,c]	0.0080	0.0044	0.0794	0.0083	0.0054	0.0958

ML test Ctrl vs. Selection (r1 and r2 are line-specific)

c - control or selection specific

Line1[r1,r2,c]:	0.1441	0.0400	0.1128	0.2158	0.0679	0.3009
Line2[r1,r2,c]:	0.1970	0.0493	0.1128	0.1790	0.0695	0.3009
Line3[r1,r2,c]:	0.1761	0.0467	0.1128	0.1718	0.0746	0.3009

c-global

Line1[r1,r2,c]:	0.1441	0.0400	0.2314	0.2157	0.0679	0.2314
Line2[r1,r2,c]:	0.1973	0.0493	0.2314	0.1789	0.0694	0.2314
Line3[r1,r2,c]:	0.1763	0.0467	0.2314	0.1717	0.0745	0.2314

ML ratio (df=14 – 13=1): 2.0019

***ML test Ctrl vs. Selection (r1, r2 and c are all line-specific)**

Line1[r1,r2,c]:	0.1440	0.0400	0.0000	0.2160	0.0680	0.3632
Line2[r1,r2,c]:	0.1973	0.0493	0.2741	0.1787	0.0693	0.1076
Line3[r1,r2,c]:	0.1760	0.0467	0.0000	0.1720	0.0747	0.4155

ML ratio (df=18 – 13=5): 7.8253

ML ratio for heterogeneity (control+selection; df = 5–1=4): 7.8253 – 2.0019

……………………………………………………………………………………………………..

markers 1-2, 3-4 (*ru-h*, *th-cu*)

	CONTROL			HYPEROXIA		
**Lines (ML estimates)**						
Line1 [r1,r2,c]:	0.1320	0.0453	0.0000	0.1507	0.0547	0.1617
Line2 [r1,r2,c]:	0.1587	0.0693	0.2424	0.1413	0.0480	0.1968
Line3 [r1,r2,c]:	0.1587	0.0480	0.1751	0.1400	0.0640	0.1488
Bailey test for 3 × 750						
Teta [r1,r2,c]:	0.1488	0.0524	0.0000	0.1440	0.0548	0.1645
SE [r1,r2,c]:	0.0075	0.0047	0.0006	0.0074	0.0048	0.0941
Chi2:		10.9555			2.2496	
C Chi2:		3.1601			0.0398	

Bailey for 6 × 750:

Teta:	0.1463	0.0536	0.0000
Chi2:	16.5906		
C chi2:	6.2682		

chi^2(ctrl + sel) - chi^2(ctrl) - chi^2(sel): 3.3855
C chi^2(ctrl + sel) - chi^2(ctrl) - chi^2(sel): **3.0683**

**ML test Ctrl Vs. Selection**						
ML est [r1,r2,c]	0.1498	0.0542	0.1642	0.1440	0.0555	0.1667
ML SE [r1,r2,c]	0.0075	0.0048	0.0931	0.0074	0.0048	0.0946

ML test Ctrl vs. Selection (r1 and r2 are line-specific)

c - control or selection specific						
Line1[r1,r2,c]:	0.1321	0.0454	0.1629	0.1507	0.0547	0.1668
Line2[r1,r2,c]:	0.1585	0.0693	0.1629	0.1413	0.0480	0.1668
Line3[r1,r2,c]:	0.1586	0.0480	0.1629	0.1400	0.0640	0.1668
c-global						
Line1[r1,r2,c]:	0.1321	0.0454	0.1646	0.1507	0.0547	0.1646
Line2[r1,r2,c]:	0.1585	0.0693	0.1646	0.1413	0.0480	0.1646
Line3[r1,r2,c]:	0.1587	0.0480	0.1646	0.1400	0.0640	0.1646

ML ratio (df=14 – 13=1): 0.0009

***ML test Ctrl vs. Selection (r1, r2 and c are all line-specific)**

Line1[r1,r2,c]:	0.1320	0.0453	0.0000	0.1507	0.0547	0.1617
Line2[r1,r2,c]:	0.1587	0.0693	0.2424	0.1413	0.0480	0.1968
Line3[r1,r2,c]:	0.1587	0.0480	0.1751	0.1400	0.0640	0.1488

ML ratio (df=18 – 13=5): 1.8225

ML ratio for heterogeneity (control+selection; df = 5–1=4): 1.8225 – 0.0009

…………………………………………………………………………………………………..

markers 1-2, 3-5 (*ru-h, th-sr*)

	CONTROL			HYPEROXIA		
**Lines (ML estimates)**						
Line1 [r1,r2,c]:	0.1320	0.0960	0.0000	0.1507	0.1187	0.0746
Line2 [r1,r2,c]:	0.1587	0.1387	0.3030	0.1413	0.1160	0.2440
Line3 [r1,r2,c]:	0.1587	0.1067	0.0788	0.1400	0.1360	0.2801
Bailey test for 3 × 750						
Teta [r1,r2,c]:	0.1486	0.1112	0.0000	0.1438	0.1230	0.1444
SE [r1,r2,c]:	0.0075	0.0066	0.0006	0.0074	0.0069	0.0584
Chi2:		16.9457			4.3487	
C Chi2:		6.5417			2.4527	

Bailey for 6 × 750:
Teta: 0.1460 0.1167 0.0000
Chi2: 28.9731
C chi2: **15.1245**

chi^2(ctrl + sel) - chi^2(ctrl) - chi^2(sel): 7.6787
C chi^2(ctrl + sel) - chi^2(ctrl) - chi^2(sel): **6.1301**

ML test Ctrl Vs. Selection

ML est [r1,r2,c]	0.1498	0.1138	0.1565	0.1440	0.1236	0.1998
ML SE [r1,r2,c]	0.0075	0.0067	0.0625	0.0074	0.0069	0.0687

ML test Ctrl vs. Selection (r1 and r2 are line-specific)

c - control or selection specific

Line1[r1,r2,c]:	0.1322	0.0962	0.1557	0.1509	0.1189	0.2001
Line2[r1,r2,c]:	0.1582	0.1383	0.1557	0.1412	0.1159	0.2001
Line3[r1,r2,c]:	0.1588	0.1068	0.1557	0.1398	0.1358	0.2001

c-global

Line1[r1,r2,c]:	0.1323	0.0962	0.1783	0.1509	0.1189	0.1783
Line2[r1,r2,c]:	0.1583	0.1383	0.1783	0.1412	0.1159	0.1783
Line3[r1,r2,c]:	0.1589	0.1068	0.1783	0.1398	0.1358	0.1783

ML ratio (df=14 – 13=1): 0.2297

***ML test Ctrl vs. Selection (r1, r2 and c are all line-specific)**

Line1[r1,r2,c]:	0.1320	0.0960	0.0000	0.1507	0.1187	0.0746
Line2[r1,r2,c]:	0.1587	0.1387	0.3030	0.1413	0.1160	0.2440
Line3[r1,r2,c]:	0.1587	0.1067	0.0788	0.1400	0.1360	0.2801

ML ratio (df=18 – 13=5): 7.7782

ML ratio for heterogeneity (control+selection; df = 5–1=4): 7.7782 – 0.2297

……………………………………………………………………………………………..

markers 1-2, 3-6 (*ru-h*, *th-e*)

	CONTROL			HYPEROXIA		
**Lines (ML estimates)**						
Line1 [r1,r2,c]:	0.1320	0.1360	0.1485	0.1507	0.1640	0.2158
Line2 [r1,r2,c]:	0.1587	0.1880	0.2235	0.1413	0.1613	0.1754
Line3 [r1,r2,c]:	0.1587	0.1533	0.1096	0.1400	0.1853	0.2055
Bailey test for 3 × 750						
Teta [r1,r2,c]:	0.1492	0.1570	0.1507	0.1439	0.1697	0.1981
SE [r1,r2,c]:	0.0075	0.0077	0.0516	0.0074	0.0079	0.0580
Chi2:		12.6054			2.1748	
C Chi2:		0.8709			0.0879	

Bailey for 6 × 750:

Teta:	0.1465	0.1631	0.1716
Chi2:	16.5691		
C chi2:	1.3089		

chi^2(ctrl + sel) - chi^2(ctrl) - chi^2(sel): 1.7889
C chi^2(ctrl + sel) - chi^2(ctrl) - chi^2(sel): **0.3501**

ML test Ctrl Vs. Selection

ML est [r1,r2,c]	0.1498	0.1591	0.1678	0.1440	0.1702	0.1994
ML SE [r1,r2,c]	0.0075	0.0077	0.0544	0.0074	0.0079	0.0581

ML test Ctrl vs. Selection (r1 and r2 are line-specific)

c - control or selection specific

Line1[r1,r2,c]:	0.1320	0.1360	0.1666	0.1506	0.1639	0.1997
Line2[r1,r2,c]:	0.1584	0.1877	0.1666	0.1414	0.1614	0.1997
Line3[r1,r2,c]:	0.1589	0.1535	0.1666	0.1400	0.1853	0.1997

c-global

Line1[r1,r2,c]:	0.1321	0.1361	0.1833	0.1506	0.1639	0.1833
Line2[r1,r2,c]:	0.1585	0.1878	0.1833	0.1414	0.1614	0.1833
Line3[r1,r2,c]:	0.1589	0.1536	0.1833	0.1399	0.1852	0.1833

ML ratio (df=14 – 13=1): 0.1736

<u>*ML test Ctrl vs. Selection (r1, r2 and c are all line-specific)</u>

Line1[r1,r2,c]:	0.1320	0.1360	0.1485	0.1507	0.1640	0.2158
Line2[r1,r2,c]:	0.1587	0.1880	0.2235	0.1413	0.1613	0.1754
Line3[r1,r2,c]:	0.1587	0.1533	0.1096	0.1400	0.1853	0.2055

ML ratio (df=18 – 13=5): 1.1333

ML ratio for heterogeneity (control+selection; df = 5–1=4): 1.1333 – 0.1736

……………………………………………………………………………………………………..

markers 1-2, 4-5 (*ru-h, cu-sr*)

	CONTROL			HYPEROXIA		
**Lines (ML estimates)**						
Line1 [r1,r2,c]:	0.1320	0.0533	0.0000	0.1507	0.0640	0.0000
Line2 [r1,r2,c]:	0.1587	0.0693	0.3638	0.1413	0.0680	0.2777
Line3 [r1,r2,c]:	0.1587	0.0587	0.0000	0.1400	0.0720	0.3972
Bailey test for 3 × 750						
Teta [r1,r2,c]:	0.1487	0.0597	0.0000	0.1436	0.0678	0.0000
SE [r1,r2,c]:	0.0075	0.0050	0.0001	0.0074	0.0053	0.0003
Chi2:		8.1453			6.1401	
C Chi2:		3.2784			5.4104	

Bailey for 6 × 750:

Teta:	0.1461	0.0635	0.0000
Chi2:	15.6664		
C chi2:	8.6888		

chi^2(ctrl + sel) - chi^2(ctrl) - chi^2(sel): 1.3810
C chi^2(ctrl + sel) - chi^2(ctrl) - chi^2(sel): **0.0000**

<u>ML test Ctrl Vs. Selection</u>

ML est [r1,r2,c]	0.1498	0.0604	0.1474	0.1440	0.0680	0.2268

ML SE [r1,r2,c]	0.0075 0.0050 0.0837	0.0074 0.0053 0.0989

ML test Ctrl vs. Selection (r1 and r2 are line-specific)

c - control or selection specific

Line1[r1,r2,c]:	0.1321	0.0534	0.1466	0.1509	0.0641	0.2273
Line2[r1,r2,c]:	0.1583	0.0692	0.1466	0.1413	0.0680	0.2273
Line3[r1,r2,c]:	0.1589	0.0587	0.1466	0.1398	0.0719	0.2273

c-global

Line1[r1,r2,c]:	0.1321	0.0534	0.1884	0.1509	0.0641	0.1884
Line2[r1,r2,c]:	0.1584	0.0692	0.1884	0.1412	0.0679	0.1884
Line3[r1,r2,c]:	0.1589	0.0587	0.1884	0.1397	0.0719	0.1884

ML ratio (df=14 – 13=1): 0.3863

***ML test Ctrl vs. Selection (r1, r2 and c are all line-specific)**

Line1[r1,r2,c]:	0.1320	0.0533	0.0000	0.1507	0.0640	0.0000
Line2[r1,r2,c]:	0.1587	0.0693	0.3638	0.1413	0.0680	0.2777
Line3[r1,r2,c]:	0.1587	0.0587	0.0000	0.1400	0.0720	0.3972

ML ratio (df=18 – 13=5): 10.2852

ML ratio for heterogeneity (control+selection; df = 5–1=4): 10.2852 – 0.3863

……………………………………………………………………………………………………..

markers 1-2, 4-6 (*ru-h*, *cu-e*)

	CONTROL	HYPEROXIA
**Lines (ML estimates)**		
Line1 [r1,r2,c]:	0.1320 0.0933 0.2165	0.1507 0.1093 0.2428
Line2 [r1,r2,c]:	0.1587 0.1187 0.2125	0.1413 0.1133 0.1665
Line3 [r1,r2,c]:	0.1587 0.1053 0.0798	0.1400 0.1213 0.2355
Bailey test for 3 × 750		
Teta [r1,r2,c]:	0.1490 0.1050 0.1351	0.1439 0.1145 0.2098
SE [r1,r2,c]:	0.0075 0.0065 0.0601	0.0074 0.0067 0.0730
Chi2:	7.2984	1.1032
C Chi2:	1.2136	0.2393

Bailey for 6 × 750:

Teta:	0.1464 0.1096 0.1653
Chi2:	10.1754
C chi2:	2.0633

chi^2(ctrl + sel) - chi^2(ctrl) - chi^2(sel): 1.7739
C chi^2(ctrl + sel) - chi^2(ctrl) - chi^2(sel): **0.6104**

ML test Ctrl Vs. Selection

ML est [r1,r2,c]	0.1498	0.1058	0.1683	0.1440	0.1147	0.2153
ML SE [r1,r2,c]	0.0075	0.0065	0.0672	0.0074	0.0067	0.0739

ML test Ctrl vs. Selection (r1 and r2 are line-specific)

c - control or selection specific

Line1[r1,r2,c]:	0.1319	0.0933	0.1672	0.1506	0.1093	0.2156
Line2[r1,r2,c]:	0.1585	0.1186	0.1672	0.1414	0.1134	0.2156
Line3[r1,r2,c]:	0.1589	0.1055	0.1672	0.1400	0.1213	0.2156

c-global

Line1[r1,r2,c]:	0.1320	0.0933	0.1918	0.1506	0.1093	0.1918
Line2[r1,r2,c]:	0.1586	0.1186	0.1918	0.1414	0.1134	0.1918
Line3[r1,r2,c]:	0.1589	0.1055	0.1918	0.1399	0.1213	0.1918

ML ratio (df=14 – 13=1): 0.2370

***ML test Ctrl vs. Selection (r1, r2 and c are all line-specific)**

Line1[r1,r2,c]:	0.1320	0.0933	0.2165	0.1507	0.1093	0.2428
Line2[r1,r2,c]:	0.1587	0.1187	0.2125	0.1413	0.1133	0.1665
Line3[r1,r2,c]:	0.1587	0.1053	0.0798	0.1400	0.1213	0.2355

ML ratio (df=18 – 13=5): 1.4938

ML ratio for heterogeneity (control+selection; df = 5–1=4): 1.4938 – 0.2370

----

markers 1-2, 5-6 (*ru-h*, *sr-e*)

	CONTROL			HYPEROXIA		
**Lines (ML estimates)**						
Line1 [r1,r2,c]:	0.1321	0.0400	0.5034	0.1506	0.0480	0.5542
Line2 [r1,r2,c]:	0.1587	0.0493	0.0000	0.1413	0.0453	0.0000
Line3 [r1,r2,c]:	0.1587	0.0467	0.1802	0.1400	0.0493	0.0000
Bailey test for 3 × 750						
Teta [r1,r2,c]:	0.1489	0.0451	0.0000	0.1438	0.0474	0.0000
SE [r1,r2,c]:	0.0075	0.0044	0.0003	0.0074	0.0045	0.0005
Chi2:		7.2154			3.8928	
C Chi2:		3.2233			3.3763	

Bailey for 6 × 750:
Teta: 0.1463 0.0462 0.0000

Chi2:	11.4574
C chi2:	6.5996

chi^2(ctrl + sel) - chi^2(ctrl) - chi^2(sel): 0.3493
C chi^2(ctrl + sel) - chi^2(ctrl) - chi^2(sel): **0.0000**

<u>ML test Ctrl Vs. Selection</u>

ML est [r1,r2,c]	0.1498	0.0453	0.1963	0.1440	0.0476	0.1947
ML SE [r1,r2,c]	0.0075	0.0044	0.1111	0.0074	0.0045	0.1103

<u>ML test Ctrl vs. Selection (r1 and r2 are line-specific)</u>

c - control or selection specific

Line1[r1,r2,c]:	0.1318	0.0399	0.1944	0.1503	0.0479	0.1949
Line2[r1,r2,c]:	0.1589	0.0494	0.1944	0.1415	0.0454	0.1949
Line3[r1,r2,c]:	0.1587	0.0467	0.1944	0.1402	0.0494	0.1949

c-global

Line1[r1,r2,c]:	0.1318	0.0399	0.1947	0.1503	0.0479	0.1947
Line2[r1,r2,c]:	0.1589	0.0494	0.1947	0.1415	0.0454	0.1947
Line3[r1,r2,c]:	0.1587	0.0467	0.1947	0.1402	0.0494	0.1947

ML ratio (df=14 – 13=1): 0.0000

<u>*ML test Ctrl vs. Selection (r1, r2 and c are all line-specific)</u>

Line1[r1,r2,c]:	0.1321	0.0400	0.5034	0.1506	0.0480	0.5542
Line2[r1,r2,c]:	0.1587	0.0493	0.0000	0.1413	0.0453	0.0000
Line3[r1,r2,c]:	0.1587	0.0467	0.1802	0.1400	0.0493	0.0000

ML ratio (df=18 – 13=5): 10.2556

ML ratio for heterogeneity (control+selection; df = 5–1=4): 10.2556 – 0.0000

……………………………………………………………………………………………………..

<u>markers 1-3, 4-5 (*ru-th*, *cu-sr*)</u>

<u>Lines (ML estimates)</u>

	CONTROL			HYPEROXIA		
Line1 [r1,r2,c]:	0.2200	0.0533	0.1136	0.2493	0.0640	0.0836
Line2 [r1,r2,c]:	0.2600	0.0693	0.2219	0.2373	0.0680	0.1652
Line3 [r1,r2,c]:	0.2761	0.0587	0.0000	0.2560	0.0720	0.2170

Bailey test for 3 × 750

Teta [r1,r2,c]:	0.2502	0.0599	0.0000	0.2473	0.0678	0.1363

SE [r1,r2,c]:	0.0091 0.0050 0.0002	0.0091 0.0053 0.0584
Chi2:	13.4107	2.0625
C Chi2:	4.2817	0.9230

Bailey for 6 × 750:
Teta: 0.2486 0.0636 0.0000
Chi2: 22.0778
C chi2: 10.6637

chi^2(ctrl + sel) - chi^2(ctrl) - chi^2(sel): 6.6046
C chi^2(ctrl + sel) - chi^2(ctrl) - chi^2(sel): **5.4590**

ML test Ctrl Vs. Selection

ML est [r1,r2,c]	0.2520 0.0604 0.1167	0.2476 0.0680 0.1585
ML SE [r1,r2,c]	0.0092 0.0050 0.0572	0.0091 0.0053 0.0630

ML test Ctrl vs. Selection (r1 and r2 are line-specific)

c - control or selection specific

Line1[r1,r2,c]:	0.2200	0.0533	0.1162	0.2496	0.0641	0.1584
Line2[r1,r2,c]:	0.2595	0.0692	0.1162	0.2373	0.0680	0.1584
Line3[r1,r2,c]:	0.2765	0.0588	0.1162	0.2558	0.0719	0.1584

c-global

Line1[r1,r2,c]:	0.2201	0.0533	0.1382	0.2495	0.0640	0.1382
Line2[r1,r2,c]:	0.2597	0.0692	0.1382	0.2372	0.0680	0.1382
Line3[r1,r2,c]:	0.2766	0.0588	0.1382	0.2557	0.0719	0.1382

ML ratio (df=14 – 13=1): 0.2473

***ML test Ctrl vs. Selection (r1, r2 and c are all line-specific)**

Line1[r1,r2,c]:	0.2200	0.0533	0.1136	0.2493	0.0640	0.0836
Line2[r1,r2,c]:	0.2600	0.0693	0.2219	0.2373	0.0680	0.1652
Line3[r1,r2,c]:	0.2761	0.0587	0.0000	0.2560	0.0720	0.2170

ML ratio (df=18 – 13=5): 5.0309

ML ratio for heterogeneity (control+selection; df = 5–1=4): 5.0309 – 0.2473

---

markers 1-3, 4-6 (*ru-th*, *cu-e*)

	CONTROL	HYPEROXIA
**Lines (ML estimates)**		
Line1 [r1,r2,c]:	0.2200 0.0933 0.1948	0.2493 0.1093 0.1467
Line2 [r1,r2,c]:	0.2600 0.1187 0.2161	0.2373 0.1133 0.0991

Line3 [r1,r2,c]:	0.2760 0.1053 0.0459	0.2560 0.1213 0.2146

Bailey test for 3 × 750

Teta [r1,r2,c]:	0.2505 0.1052 0.0922	0.2473 0.1144 0.1424
SE [r1,r2,c]:	0.0091 0.0065 0.0382	0.0091 0.0067 0.0458
Chi2:	14.3552	2.3502
C Chi2:	3.7330	1.0189

Bailey for 6 × 750:

Teta: 0.2489 0.1096 0.1128
Chi2: 18.3708
C chi2: 5.4363

chi^2(ctrl + sel) - chi^2(ctrl) - chi^2(sel): 1.6655
C chi^2(ctrl + sel) - chi^2(ctrl) - chi^2(sel): **0.6844**

ML test Ctrl Vs. Selection

ML est [r1,r2,c]	0.2520 0.1058 0.1501	0.2476 0.1147 0.1566
ML SE [r1,r2,c]	0.0092 0.0065 0.0486	0.0091 0.0067 0.0480

ML test Ctrl vs. Selection (r1 and r2 are line-specific)

c - control or selection specific

Line1[r1,r2,c]:	0.2198	0.0932	0.1490	0.2494	0.1094	0.1565
Line2[r1,r2,c]:	0.2595	0.1184	0.1490	0.2377	0.1135	0.1565
Line3[r1,r2,c]:	0.2768	0.1056	0.1490	0.2556	0.1211	0.1565

c-global

Line1[r1,r2,c]:	0.2198	0.0933	0.1529	0.2494	0.1093	0.1529
Line2[r1,r2,c]:	0.2595	0.1185	0.1529	0.2376	0.1135	0.1529
Line3[r1,r2,c]:	0.2768	0.1056	0.1529	0.2556	0.1211	0.1529

ML ratio (df=14 – 13=1): 0.0123

***ML test Ctrl vs. Selection (r1, r2 and c are all line-specific)**

Line1[r1,r2,c]:	0.2200	0.0933	0.1948	0.2493	0.1093	0.1467
Line2[r1,r2,c]:	0.2600	0.1187	0.2161	0.2373	0.1133	0.0991
Line3[r1,r2,c]:	0.2760	0.1053	0.0459	0.2560	0.1213	0.2146

ML ratio (df=18 – 13=5): 4.1289

ML ratio for heterogeneity (control+selection; df = 5–1=4): 4.1289 – 0.0123

…………………………………………………………………………………………………..

markers 1-3, 5-6 (*ru-th*, *sr-e*)

CONTROL **HYPEROXIA**

Lines (ML estimates)

Line1 [r1,r2,c]:	0.2200 0.0400 0.3033	0.2493 0.0480 0.4460
Line2 [r1,r2,c]:	0.2600 0.0493 0.2079	0.2374 0.0453 0.0000
Line3 [r1,r2,c]:	0.2760 0.0467 0.1035	0.2560 0.0493 0.2111

Bailey test for 3 × 750

Teta [r1,r2,c]:	0.2506 0.0452 0.1602	0.2469 0.0474 0.0000
SE [r1,r2,c]:	0.0091 0.0044 0.0767	0.0091 0.0045 0.0001
Chi2:	9.1372	7.6384
C Chi2:	0.9081	6.7634

Bailey for 6 × 750:

Teta: 0.2485 0.0462 0.0000
Chi2: 21.3087
C chi2: 12.1190

chi^2(ctrl + sel) - chi^2(ctrl) - chi^2(sel): 4.5331
C chi^2(ctrl + sel) - chi^2(ctrl) - chi^2(sel): **4.4475**

ML test Ctrl Vs. Selection

ML est [r1,r2,c]	0.2520 0.0453 0.1946	0.2475 0.0476 0.2265
ML SE [r1,r2,c]	0.0092 0.0044 0.0844	0.0091 0.0045 0.0893

ML test Ctrl vs. Selection (r1 and r2 are line-specific)

c - control or selection specific

Line1[r1,r2,c]:	0.2198	0.0400	0.1928	0.2488	0.0479	0.2265
Line2[r1,r2,c]:	0.2600	0.0493	0.1928	0.2378	0.0454	0.2265
Line3[r1,r2,c]:	0.2763	0.0467	0.1928	0.2560	0.0493	0.2265

c-global

Line1[r1,r2,c]:	0.2198	0.0400	0.2099	0.2487	0.0479	0.2099
Line2[r1,r2,c]:	0.2600	0.0493	0.2099	0.2378	0.0454	0.2099
Line3[r1,r2,c]:	0.2763	0.0467	0.2099	0.2560	0.0493	0.2099

ML ratio (df=14 – 13=1): 0.0758

***ML test Ctrl vs. Selection (r1, r2 and c are all line-specific)**

Line1[r1,r2,c]:	0.2200	0.0400	0.3033	0.2493	0.0480	0.4460
Line2[r1,r2,c]:	0.2600	0.0493	0.2079	0.2374	0.0453	0.0000
Line3[r1,r2,c]:	0.2760	0.0467	0.1035	0.2560	0.0493	0.2111

ML ratio (df=18 – 13=5): 6.3872

ML ratio for heterogeneity (control+selection; df = 5–1=4): 6.3872 – 0.0758

……………………………………………………………………………………………………..

markers 1-4, 5-6 (*ru-cu*, *sr-e*)

	CONTROL			HYPEROXIA		
**Lines (ML estimates)**						
Line1 [r1,r2,c]:	0.2653	0.0400	0.2514	0.2960	0.0480	0.3756
Line2 [r1,r2,c]:	0.3213	0.0493	0.1682	0.2801	0.0453	0.0000
Line3 [r1,r2,c]:	0.3187	0.0467	0.0897	0.3120	0.0493	0.1732

Bailey test for 3 × 750

	CONTROL			HYPEROXIA		
Teta [r1,r2,c]:	0.3006	0.0452	0.1363	0.2951	0.0474	0.0000
SE [r1,r2,c]:	0.0096	0.0044	0.0647	0.0096	0.0045	0.0003
Chi2:		9.7573			8.7810	
C Chi2:		0.8123			6.7474	

Bailey for 6 × 750:
Teta: 0.2976 0.0462 0.0000
Chi2: 23.2071
C chi2: 12.0935

chi^2(ctrl + sel) - chi^2(ctrl) - chi^2(sel): 4.6688
C chi^2(ctrl + sel) - chi^2(ctrl) - chi^2(sel): **4.5339**

ML test Ctrl Vs. Selection

	CONTROL			HYPEROXIA		
ML est [r1,r2,c]	0.3018	0.0453	0.1625	0.2960	0.0476	0.1894
ML SE [r1,r2,c]	0.0097	0.0044	0.0705	0.0096	0.0045	0.0747

ML test Ctrl vs. Selection (r1 and r2 are line-specific)

c - control or selection specific

Line1[r1,r2,c]:	0.2651	0.0400	0.1610	0.2953	0.0479	0.1895
Line2[r1,r2,c]:	0.3213	0.0493	0.1610	0.2806	0.0454	0.1895
Line3[r1,r2,c]:	0.3190	0.0467	0.1610	0.3121	0.0493	0.1895

c-global

Line1[r1,r2,c]:	0.2651	0.0400	0.1754	0.2953	0.0479	0.1754
Line2[r1,r2,c]:	0.3214	0.0493	0.1754	0.2805	0.0454	0.1754
Line3[r1,r2,c]:	0.3190	0.0467	0.1754	0.3120	0.0493	0.1754

ML ratio (df=14 – 13=1): 0.0766

***ML test Ctrl vs. Selection (r1, r2 and c are all line-specific)**

Line1[r1,r2,c]:	0.2653	0.0400	0.2514	0.2960	0.0480	0.3756
Line2[r1,r2,c]:	0.3213	0.0493	0.1682	0.2801	0.0453	0.0000
Line3[r1,r2,c]:	0.3187	0.0467	0.0897	0.3120	0.0493	0.1732

ML ratio (df=18 – 13=5): 6.2827

ML ratio for heterogeneity (control+selection; df = 5–1=4): 6.2827 – 0.0766

----

markers 2-3, 4-5 (*h-th*, *cu-sr*)

	CONTROL			HYPEROXIA		
**Lines (ML estimates)**						
Line1 [r1,r2,c]:	0.0987	0.0533	0.2530	0.1173	0.0640	0.1777
Line2 [r1,r2,c]:	0.1307	0.0693	0.0000	0.1093	0.0680	0.0000
Line3 [r1,r2,c]:	0.1307	0.0587	0.0000	0.1240	0.0720	0.2987
Bailey test for 3 × 750						
Teta [r1,r2,c]:	0.1183	0.0600	0.0000	0.1165	0.0677	0.0000
SE [r1,r2,c]:	0.0068	0.0050	0.0002	0.0068	0.0053	0.0005
Chi2:		8.4617			4.3392	
C Chi2:		1.0389			3.1629	

Bailey for 6 × 750:
Teta: 0.1174 0.0637 0.0000
Chi2: 13.9357
C chi2: 4.2018

chi^2(ctrl + sel) - chi^2(ctrl) - chi^2(sel): 1.1348
C chi^2(ctrl + sel) - chi^2(ctrl) - chi^2(sel): **0.0001**

**ML test Ctrl Vs. Selection**	CONTROL			HYPEROXIA		
ML est [r1,r2,c]	0.1200	0.0604	0.0613	0.1169	0.0680	0.1677
ML SE [r1,r2,c]	0.0069	0.0050	0.0609	0.0068	0.0053	0.0953

ML test Ctrl vs. Selection (r1 and r2 are line-specific)

	CONTROL			HYPEROXIA		
c - control or selection specific						
Line1[r1,r2,c]:	0.0986	0.0533	0.0605	0.1173	0.0640	0.1678
Line2[r1,r2,c]:	0.1307	0.0694	0.0605	0.1094	0.0681	0.1678
Line3[r1,r2,c]:	0.1307	0.0587	0.0605	0.1239	0.0719	0.1678
c-global						
Line1[r1,r2,c]:	0.0986	0.0533	0.1165	0.1173	0.0640	0.1165
Line2[r1,r2,c]:	0.1308	0.0694	0.1165	0.1094	0.0680	0.1165
Line3[r1,r2,c]:	0.1308	0.0587	0.1165	0.1238	0.0719	0.1165

ML ratio (df=14 – 13=1): 0.9139

***ML test Ctrl vs. Selection (r1, r2 and c are all line-specific)**

	CONTROL			HYPEROXIA		
Line1[r1,r2,c]:	0.0987	0.0533	0.2530	0.1173	0.0640	0.1777
Line2[r1,r2,c]:	0.1307	0.0693	0.0000	0.1093	0.0680	0.0000
Line3[r1,r2,c]:	0.1307	0.0587	0.0000	0.1240	0.0720	0.2987

ML ratio (df=18 – 13=5): 6.2881

ML ratio for heterogeneity (control+selection; df = 5–1=4): 6.2881 – 0.9139

……………………………………………………………………………………………..

markers 2-3, 4-6 (*h-th*, *cu-e*)

	CONTROL			HYPEROXIA		
**Lines (ML estimates)**						
Line1 [r1,r2,c]:	0.0987	0.0933	0.1447	0.1173	0.1093	0.0000
Line2 [r1,r2,c]:	0.1307	0.1187	0.1720	0.1093	0.1133	0.0000
Line3 [r1,r2,c]:	0.1307	0.1053	0.0000	0.1240	0.1213	0.3545
Bailey test for 3 × 750						
Teta [r1,r2,c]:	0.1182	0.1051	0.0000	0.1164	0.1143	0.0000
SE [r1,r2,c]:	0.0068	0.0065	0.0001	0.0068	0.0067	0.0001
Chi2:		11.6341			5.7084	
C Chi2:		3.1241			4.3918	

Bailey for 6 × 750:
Teta: 0.1173 0.1095 0.0000
Chi2: 18.3247
C chi2: 7.5159

chi^2(ctrl + sel) - chi^2(ctrl) - chi^2(sel): 0.9821
C chi^2(ctrl + sel) - chi^2(ctrl) - chi^2(sel): **0.0000**

ML test Ctrl Vs. Selection

	CONTROL			HYPEROXIA		
ML est [r1,r2,c]	0.1200	0.1058	0.1050	0.1169	0.1147	0.1326
ML SE [r1,r2,c]	0.0069	0.0065	0.0599	0.0068	0.0067	0.0653

ML test Ctrl vs. Selection (r1 and r2 are line-specific)

c - control or selection specific						
Line1[r1,r2,c]:	0.0986	0.0933	0.1039	0.1175	0.1095	0.1327
Line2[r1,r2,c]:	0.1305	0.1185	0.1039	0.1095	0.1135	0.1327
Line3[r1,r2,c]:	0.1308	0.1055	0.1039	0.1236	0.1210	0.1327
c-global						
Line1[r1,r2,c]:	0.0986	0.0933	0.1187	0.1175	0.1095	0.1187
Line2[r1,r2,c]:	0.1306	0.1186	0.1187	0.1095	0.1135	0.1187
Line3[r1,r2,c]:	0.1309	0.1055	0.1187	0.1236	0.1209	0.118

ML ratio (df=14 – 13=1): 0.1062

***ML test Ctrl vs. Selection (r1, r2 and c are all line-specific)**

Line1[r1,r2,c]:	0.0987	0.0933	0.1447	0.1173	0.1093	0.0000
Line2[r1,r2,c]:	0.1307	0.1187	0.1720	0.1093	0.1133	0.0000
Line3[r1,r2,c]:	0.1307	0.1053	0.0000	0.1240	0.1213	0.3545

ML ratio (df=18 – 13=5): 10.9314

ML ratio for heterogeneity (control+selection; df = 5–1=4): 10.9314 – 0.1062

……………………………………………………………………………………………………..

markers 2-3, 5-6 (*h-th*, *sr-e*)

	CONTROL			HYPEROXIA		
**Lines (ML estimates)**						
Line1 [r1,r2,c]:	0.0987	0.0400	0.0000	0.1173	0.0480	0.2369
Line2 [r1,r2,c]:	0.1307	0.0493	0.4145	0.1093	0.0453	0.0000
Line3 [r1,r2,c]:	0.1307	0.0467	0.0000	0.1240	0.0493	0.4369
Bailey test for 3 × 750						
Teta [r1,r2,c]:	0.1181	0.0450	0.0000	0.1165	0.0474	0.0000
SE [r1,r2,c]:	0.0068	0.0044	0.0005	0.0068	0.0045	0.0003
Chi2:		8.5148			4.1548	
C Chi2:		2.1665			3.2116	

Bailey for 6 × 750:

Teta:	0.1173	0.0462	0.0000
Chi2:	12.8403		
C chi2:	5.3781		

chi^2(ctrl + sel) - chi^2(ctrl) - chi^2(sel): 0.1708
C chi^2(ctrl + sel) - chi^2(ctrl) - chi^2(sel): **0.0000**

ML test Ctrl Vs. Selection

ML est [r1,r2,c]	0.1200	0.0453	0.1634	0.1169	0.0476	0.2400
ML SE [r1,r2,c]	0.0069	0.0044	0.1140	0.0068	0.0045	0.1357

ML test Ctrl vs. Selection (r1 and r2 are line-specific)

c - control or selection specific

Line1[r1,r2,c]:	0.0987	0.0400	0.1623	0.1173	0.0480	0.2398
Line2[r1,r2,c]:	0.1305	0.0493	0.1623	0.1094	0.0454	0.2398
Line3[r1,r2,c]:	0.1308	0.0467	0.1623	0.1239	0.0493	0.2398

c-global

Line1[r1,r2,c]:	0.0987	0.0400	0.2011	0.1173	0.0480	0.2011
Line2[r1,r2,c]:	0.1305	0.0493	0.2011	0.1094	0.0454	0.2011
Line3[r1,r2,c]:	0.1308	0.0467	0.2011	0.1238	0.0493	0.2011

ML ratio (df=14 – 13=1): 0.1948

***ML test Ctrl vs. Selection (r1, r2 and c are all line-specific)**

Line1[r1,r2,c]:	0.0987	0.0400	0.0000	0.1173	0.0480	0.2369
Line2[r1,r2,c]:	0.1307	0.0493	0.4145	0.1093	0.0453	0.0000
Line3[r1,r2,c]:	0.1307	0.0467	0.0000	0.1240	0.0493	0.4369

ML ratio (df=18 – 13=5): 6.4856

ML ratio for heterogeneity (control+selection; df = 5–1=4): 6.4856 – 0.1948

……………………………………………………………………………………………………..

markers 2-4, 5-6 (*h-cu*, *sr-e*)

	CONTROL			HYPEROXIA		
**Lines (ML estimates)**						
Line1 [r1,r2,c]:	0.1440	0.0400	0.0000	0.1666	0.0480	0.1668
Line2 [r1,r2,c]:	0.1973	0.0493	0.2741	0.1547	0.0453	0.0000
Line3 [r1,r2,c]:	0.1760	0.0467	0.0000	0.1827	0.0493	0.2959
Bailey test for 3 × 750						
Teta [r1,r2,c]:	0.1700	0.0450	0.0000	0.1672	0.0474	0.0000
SE [r1,r2,c]:	0.0079	0.0044	0.0002	0.0079	0.0045	0.0002
Chi2:		11.1839			5.4702	
C Chi2:		2.1495			3.1887	

Bailey for 6 × 750:
Teta: 0.1686 0.0462 0.0000
Chi2: 16.8540
C chi2: 5.3382

chi^2(ctrl + sel) - chi^2(ctrl) - chi^2(sel): 0.1998
C chi^2(ctrl + sel) - chi^2(ctrl) - chi^2(sel): **0.0000**

ML test Ctrl Vs. Selection

ML est [r1,r2,c]	0.1724	0.0453	0.1136	0.1680	0.0476	0.1670
ML SE [r1,r2,c]	0.0080	0.0044	0.0794	0.0079	0.0045	0.0946

ML test Ctrl vs. Selection (r1 and r2 are line-specific)

c - control or selection specific

Line1[r1,r2,c]:	0.1441	0.0400	0.1128	0.1667	0.0480	0.1667

Line2[r1,r2,c]:	0.1970	0.0493	0.1128	0.1548	0.0454	0.1667
Line3[r1,r2,c]:	0.1761	0.0467	0.1128	0.1825	0.0493	0.1667

c-global

Line1[r1,r2,c]:	0.1441	0.0400	0.1400	0.1666	0.0480	0.1400
Line2[r1,r2,c]:	0.1971	0.0493	0.1400	0.1548	0.0454	0.1400
Line3[r1,r2,c]:	0.1762	0.0467	0.1400	0.1824	0.0493	0.1400

ML ratio (df=14 – 13=1): 0.1933

***ML test Ctrl vs. Selection (r1, r2 and c are all line-specific)**

Line1[r1,r2,c]:	0.1440	0.0400	0.0000	0.1666	0.0480	0.1668
Line2[r1,r2,c]:	0.1973	0.0493	0.2741	0.1547	0.0453	0.0000
Line3[r1,r2,c]:	0.1760	0.0467	0.0000	0.1827	0.0493	0.2959

ML ratio (df=18 – 13=5): 6.1936

ML ratio for heterogeneity (control+selection; df = 5–1=4): 6.1936 – 0.1933

…………………………………………………………………………………………………..
