# Supplementary material for: Experimental evolution of recombination and crossover interference in Drosophila caused by directional selection for stress-related traits
Source: BMC Biol. 2015 Nov 27;13:101. doi: 10.1186/s12915-015-0206-5 (PMC4661966; doi:10.1186/s12915-015-0206-5)
Supplement: Additional file 6: — Text S1. Changes in recombination and interference are not necessarily directly coupled.(PDF 199 kb) [file 12915_2015_206_MOESM6_ESM.pdf]

Additional file 6: Text S1

Changes in recombination and interference are not necessarily directly coupled

The performed analysis enabled us to gain some additional support for the conclusion on the effect of selection on interference. Namely, in most analyzed cases, we could see rather low heterogeneity of the $\hat{c}$ values between the three independent replicates within selection and control lines (Additional files 4 and 5). Moreover, as a rule, in cases with significant effect of selection on interference, the ranges of the three $\hat{c}$ values for control lines and selection lines did not overlap. This pattern was typical to all three selection experiments for both adjacent and non-adjacent pairs of intervals (see Additional files 4 and 5, respectively). It was also observed in the rare situations of significant heterogeneity of $\hat{c}$ estimates among the three replicates in either control or selection lines. The results on significant relaxation of positive interference in the *h-th-e* region of chromosome 3 in lines selected for desiccation tolerance can serve as an example of such a situation. The **c** vectors of ML-estimates in control and selection replicates were: $\hat{\mathbf{c}}_{\text{control}}$=(0.1093, 0.4506, 0.4102) and $\hat{\mathbf{c}}_{\text{selection}}$=(1.0944, 1.1058, 1.0023), with χ^2=7.09 (df=2, p<0.05) in control and χ^2=0.32 (ns) in selection, respectively (see Additional file 4). Another illustrative example is provided by the results of the effect of hypoxia-tolerance selection on the *y-cv-f* region, with heterogeneity of both selection lines and control lines. Here we obtained χ^2=6.33 (p<0.05) between control lines and 30.4 (p<2.5E-7) between selection lines. However, despite the significant heterogeneity of the $\hat{c}$ estimates, the ranges of variation of

control lines and selection lines did not overlap: $\hat{\boldsymbol{c}}_{control}$=(1.096, 1.257, 0.812) and $\hat{\boldsymbol{c}}_{selection}$=(2.153, 1.728, 2.405), as in most other cases (Additional file 4).

A usual (conservative) assumption is that the observed changes in interference simply accompany the increase in *rf*. The relationships between changes in *rf* and interference have been discussed for decades and remain a hot topic (Foss et al., 1993; Fujitani et al., 2002; Zhang et al., 2014). In our tests, relaxation of positive interference or appearance of negative interference indeed tended, to some extent, to coincide with increased *rf* in one (Additional file 1: Table S4b) or both (Additional file 1: Table S4c) of the considered pairs of intervals. However, significant changes in interference were also observed in cases where *rf* was not affected by selection (compare also Text tables 1 and 3 with Tables S2 and S3 in Additional file 1). Moreover, no change in interference was observed in a part of interval pairs where one or both intervals displayed a significant increase in *rf* in the selection lines. Therefore, we consider our results as indicative of evolvability of some components controlling crossover interference independently of crossover rate, similar to the conclusions reached in studies of changes in crossover rate and interference upon heat stress and interchromosomal effects (Grell 1978).

Text S1 references

1. Foss E, Lande R, Stahl WF, Steinberg CM: Chiasma Interference as a Function of Genetic Distance. *Genetics* 1993, 133:681–691.

2. Fujitani Y, Mori S, Kobayashi I: A Reaction-Diffusion Model for Interference in Meiotic Crossing Over. *Genetics* 2002, 161:365–372.
3. Zhang L, Liang Z, Hutchinson J, Kleckner N: Crossover Patterning by the Beam-Film Model: Analysis and Implications. *PLoS Genetics* 2014, 10: e1004042.
4. Grell RF: A comparison of heat and inter-chromosomal effects on recombination and interference in *Drosophila melanogaster*. *Genetics* 1978, 89:65–77.
